# Synthesis of 2-oxoglutarate derivatives and their evaluation as cosubstrates and inhibitors of human aspartate/asparagine-β-hydroxylase†

**DOI:** 10.1039/d0sc04301j

**Published:** 2020-12-07

**Authors:** Lennart Brewitz, Yu Nakashima, Christopher J. Schofield

**Affiliations:** Chemistry Research Laboratory, University of Oxford 12 Mansfield Road OX1 3TA Oxford UK christopher.schofield@chem.ox.ac.uk

## Abstract

2-Oxoglutarate (2OG) is involved in biological processes including oxidations catalyzed by 2OG oxygenases for which it is a cosubstrate. Eukaryotic 2OG oxygenases have roles in collagen biosynthesis, lipid metabolism, DNA/RNA modification, transcriptional regulation, and the hypoxic response. Aspartate/asparagine-β-hydroxylase (AspH) is a human 2OG oxygenase catalyzing post-translational hydroxylation of Asp/Asn-residues in epidermal growth factor-like domains (EGFDs) in the endoplasmic reticulum. AspH is of chemical interest, because its Fe(ii) cofactor is complexed by two rather than the typical three residues. AspH is upregulated in hypoxia and is a prognostic marker on the surface of cancer cells. We describe studies on how derivatives of its natural 2OG cosubstrate modulate AspH activity. An efficient synthesis of C3- and/or C4-substituted 2OG derivatives, proceeding *via* cyanosulfur ylid intermediates, is reported. Mass spectrometry-based AspH assays with >30 2OG derivatives reveal that some efficiently inhibit AspH *via* competing with 2OG as evidenced by crystallographic and solution analyses. Other 2OG derivatives can substitute for 2OG enabling substrate hydroxylation. The results show that subtle changes, *e.g.* methyl- to ethyl-substitution, can significantly alter the balance between catalysis and inhibition. 3-Methyl-2OG, a natural product present in human nutrition, was the most efficient alternative cosubstrate identified; crystallographic analyses reveal the binding mode of (*R*)-3-methyl-2OG and other 2OG derivatives to AspH and inform on the balance between turnover and inhibition. The results will enable the use of 2OG derivatives as mechanistic probes for other 2OG utilizing enzymes and suggest 2-oxoacids other than 2OG may be employed by some 2OG oxygenases *in vivo*.

## Abstract 2

Efficient synthesis of 2-oxoglutarate derivatives enables identification of inhibitors and novel cosubstrates of human aspartate/asparagine-β-hydroxylase as revealed by spectroscopic and crystallographic studies.

## Graphical abstract

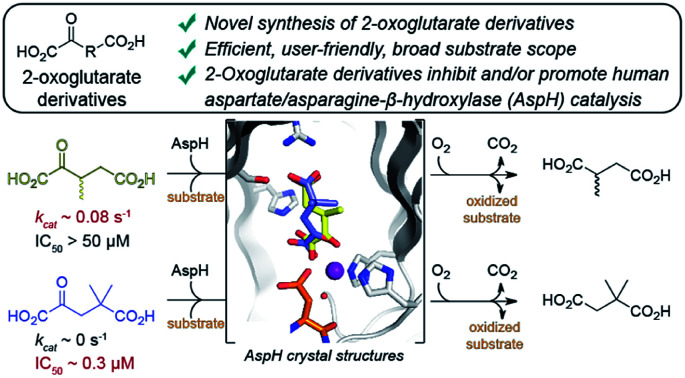

## Introduction

**Fig. 1:**
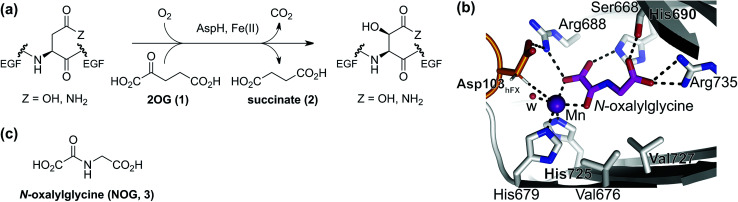
The AspH active site and stoichiometry of its reaction. (a) AspH catalyzes the post-translational hydroxylation of Asn- and Asp-residues in epidermal growth factor (EGF)-like domains; (b) analysis of an AspH:substrate (human Factor X, hFX) crystal structure (PDB ID: 5JQY)^22^ reveals that two AspH residues (His679 and His725) coordinate the active site metal rather than the typical three residues found in other human 2OG dependent hydroxylases. In the crystallographic analyses, Mn substitutes for Fe and NOG (3) for 2OG (1); (c) *N*-oxalylglycine (NOG, 3).

2-Oxoglutarate (2OG, α-ketoglutarate; 1, Fig. 1a) is an integral metabolite in most of biology including prokaryotes, archaea, and eukaryotes;^1^ 2OG is crucially involved in cellular energy homeostasis and small-molecule metabolism^1^ and can act as a signaling molecule linking nitrogen and carbon metabolism.^2^ 2OG is an intermediate of the tricarboxylic acid (TCA) cycle where it is produced by isocitrate dehydrogenase (IDH)-catalyzed decarboxylation of d-isocitrate; 2OG is converted to succinyl-CoA and CO_2_ by the 2OG dehydrogenase complex. Reductions in cellular 2OG coupled with the formation of (*R*)-2-hydroxyglutarate, which occur as a result of IDH mutations,^3^ are associated with changes in epigenetic regulation (*e.g.* DNA and histone methylation status) and certain types of cancer.^3*b*,4^ These effects are proposed to be mediated, at least in part, by modulation of the activities of enzymes that rely on 2OG as a (co-)substrate, other than the 2OG dehydrogenase complex. Such enzymes include aminotransferases (*e.g.* branched-chain aminotransferases, BCATs),^5^ which convert 2OG to glutamate, and 2OG dependent oxygenases.^6^ The latter couple the conversion of 2OG and O_2_ to succinate and CO_2_ with substrate hydroxylation or demethylation *via* hydroxylation.^7^ There are approximately 60–70 assigned human 2OG oxygenases, all studied members of which likely employ Fe(ii) as a cofactor and which have diverse roles, ranging from DNA/RNA modification and damage repair,^8^ histone/chromatin modification,^9^ lipid metabolism,^10^ post-translational modification of proteins with important functions in the extracellular matrix,^7,11^ to hypoxia sensing.^12^

The human 2OG oxygenase aspartate/asparagine-β-hydroxylase (AspH, BAH, HAAH)^13^ is highly unusual amongst human 2OG dependent hydroxylases, because its Fe(ii) cofactor is complexed by only two residues (His679 and His725) rather than by the typical triad of ligands (HXD/E⋯H) found in other human 2OG oxygenases (Fig. 1b).^7,14^ AspH catalyzes the post-translational hydroxylation of specific Asp- and Asn-residues in epidermal growth factor-like domains (EGFDs) of its substrate proteins in the endoplasmic reticulum (Fig. 1a).^15^ AspH is of significant interest from a cancer research perspective, because its levels are upregulated in invasive cancers (*e.g.* hepatocellular carcinoma^16^ and pancreatic cancer^17^) and it is translocated to the cell surface where it can be used as a diagnostic and prognostic marker.^18^ Mouse models^19^ and heritable genetic diseases associated with mutations likely effecting AspH catalysis (*e.g.* Traboulsi syndrome)^20^ suggest that the Notch signaling pathway may be involved in transmitting the effect of AspH on cancer invasiveness. AspH levels are regulated by hypoxia which is a characteristic of many tumor cells.^21^ Thus, AspH appears to be an attractive medicinal chemistry and diagnostic target for certain types of cancer.

Recent studies have provided crystallographic and solution-based evidence that AspH accepts EGFD substrates with an unusual non-canonical disulfide connectivity (*i.e.* Cys 1–2, 3–4, 5–6) rather than the well-characterized canonical disulfide connectivity (*i.e.* Cys 1–3, 2–4, 5–6; ESI Fig. S1†).^22^ High-throughput MS assays were established to monitor the catalytic activity of AspH using stable thioether-linked cyclic peptide substrate analogues mimicking the central non-canonical macrocyclic Cys 3–4 EGFD disulfide (ESI Fig. S1†).^23^ Kinetic studies have revealed that AspH is sensitive towards subtle changes in oxygen availability and thus it is a candidate oxygenase for involvement in hypoxia sensing.^23^

Previous studies have revealed that differences in the cosubstrate binding sites of 2OG oxygenases can be exploited for stereoselective selective inhibition employing 2OG analogues, *e.g. N*-oxalylamino acids.^24^ Analysis of reported AspH crystal structures wherein 2OG is replaced by a close 2OG analogue, *i.e. N*-oxalylglycine (NOG, 3; Fig. 1c),^22^ suggest that the AspH active site is sufficiently spacious to accommodate substituents at the 2OG C3- and/or C4-position, in part due to its unusual Fe(ii)-binding geometry (Fig. 1b). We were therefore interested to test this by exploring how a diverse set of 2OG derivatives interact with AspH.

**Fig. 2:**
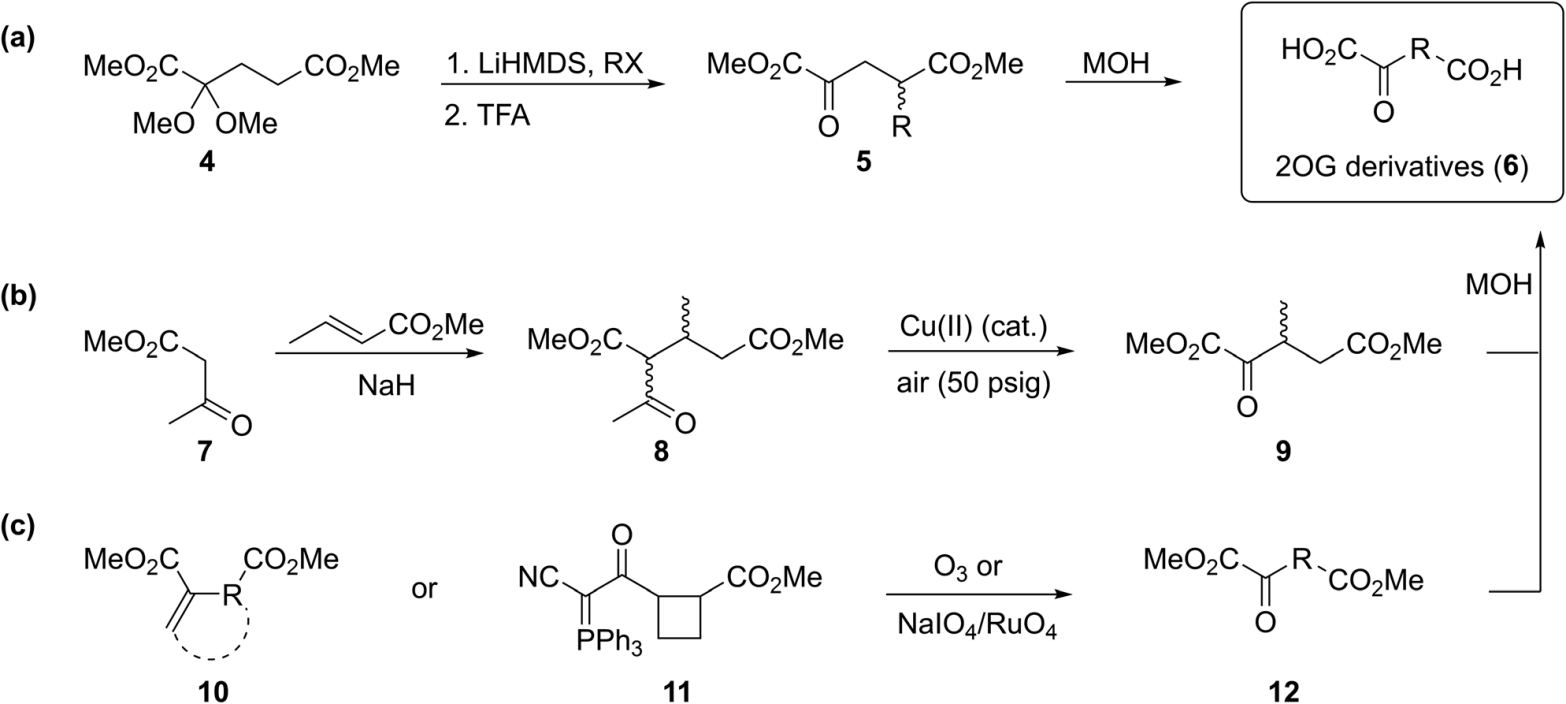
Selected reported strategies for the synthesis of C3- and/or C4-substituted 2OG derivatives. (a) Alkylation reactions,^25^ (b) Michael reactions,^27^ and (b and c) oxidation reactions^28*a*–*c*,29^ have been employed in syntheses of C3/C4-substituted 2OG derivatives (6).

To our knowledge, only a limited number of studies describing how 2OG derivatives bearing substituents at the C3- and/or C4-position modulate the activities of 2OG oxygenases (*i.e.* human JmjC histone *N*^ε^-methyl lysine demethylase 4A, KDM4A;^25^ human factor inhibiting the hypoxia-inducible transcription factor HIF-α, FIH;^24^ and rat γ-butyrobetaine dioxygenase, BBOX^26^) are reported. In part, this likely reflects a lack of a simple synthetic method to access these types of 2OG derivatives. Some prior syntheses of C3/C4-substituted 2OG derivatives have relied *inter alia* on alkylation (Fig. 2a)^25^ and Michael reactions (Fig. 2b)^27^ to access key synthetic intermediates; the corresponding 2OG derivatives (6) were obtained after saponification. Other approaches rely on oxidation reactions using ozone^28^ or sodium periodate^29^ as oxidants to convert Michael acceptors (10) into 2OG derivatives (Fig. 2c). The described syntheses are frequently associated with limited scalability, low overall chemical yields, and/or narrow substrate scopes due to harsh reaction conditions requiring, for example, the use of strong bases and acids,^25,30^ high pressure,^27^ or strong oxidants.^28*a*–*c*,29^

Cyanophosphorous ylids are reported as valuable intermediates for the synthesis of α-keto acids,^31^ however, only one example of a cyanophosphorous ylid (*i.e.*11) being converted into a 2OG derivative is reported (Fig. 2c),^32^ possibly reflecting limitations associated with the conversion of cyanophosphorous ylids into α-keto acids which requires strong oxidants.^31–33^ The use of cyanophosphorous ylids has been largely superseded by the corresponding cyanosulfur ylids, which can be oxidized under milder conditions.^33*a*,34^ We thus envisaged that cyanosulfur ylids could be used for the synthesis of C3/C4-substituted 2OG derivatives.

Here we report the use of cyanosulfur ylids as intermediates that enable the facile synthesis of multiple 2OG derivatives bearing a diverse set of substituents at the C3- and/or C4-positions. The synthetic 2OG derivatives were used to modulate the activity of recombinant human AspH. Kinetic and crystallographic studies were employed to elucidate the mechanisms by which the 2OG derivatives modulate AspH activity and to garner information of the active site requirements of AspH. The results reveal an unexpectedly diverse set of 2OG derivatives can bind at the AspH active site and that subtle differences in the 2OG substitution pattern can cause significant disturbances in the balance between productive catalysis and inhibition.

## Results

### Synthesis of 2OG derivatives

**Scheme 1:**
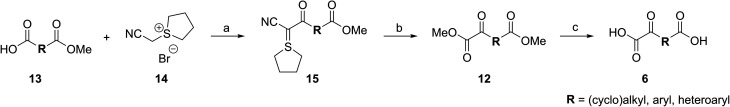
Route and substrate scope for the synthesis of 2OG derivatives. Reagents and conditions: (a) T3P, ^i^Pr_2_NEt, CH_2_Cl_2_, 0 °C to rt, 11–95%; (b) oxone, MeOH/H_2_O, rt, 39–98%; (c) LiOH, MeOH/H_2_O, 0 °C to rt, then: purification by ion exchange chromatography (Dowex® 50XW8), 67% to apparent quantitative.

A versatile and scalable synthetic route to access 2OG derivatives was developed employing cyanosulfur ylids as key intermediates (Scheme 1). The route employs mono-methyl dicarboxylic acid half-esters 13 as starting materials which were either commercially available or synthesized by established reactions, *i.e.* nucleophilic openings of the requisite symmetric cyclic anhydrides, formylation reactions of aryl iodides,^35^ Heck couplings^36^ of aryl iodides with orthogonally protected itaconates,^37^ or Horner–Wadsworth–Emmons (HWE)^38^ reactions (ESI Fig. S2†). In our work, racemic mixtures of mono-methyl dicarboxylic acid half-esters 13 bearing stereogenic carbon atoms at the 2OG C3- or C4-equivalent position were employed.

Cyanosulfur ylids 15, which are the key intermediates in our strategy, were obtained by reaction of mono-methyl dicarboxylic acid half-esters 13 with the reported tetrahydrothiophene bromide salt  14^33*a*^ in yields ranging from 11 to 95%. T3P^39^ was chosen as the coupling reagent because it is suited for use with sterically hindered carboxylic acids, including those bearing substituents at the carboxylate α-position. For some substrates, T3P-derived byproducts interfered with the purification process; however, these were completely removed after the subsequent reaction by chromatography.

The cyanosulfur ylids 15 were converted into the corresponding dimethyl dicarboxylic acid esters 12 using oxone^33*a*^ as a mild oxidation reagent in methanol, in part to avoid ester exchange (Scheme 1). The dimethyl esters 12 were obtained in high purity after column chromatography in yields ranging from 39 to 98%. Lithium hydroxide-mediated saponification of dimethyl dicarboxylic acid esters 12 afforded the desired 2OG derivatives 6 (Scheme 1). The 2OG derivatives were obtained in sufficient purity after removal of excess base by acidic ion exchange chromatography yielding salt-free dicarboxylic acids 6 (ESI†), which are suitable for performing *in vitro* biochemical experiments with AspH. The 2OG derivatives and their synthetic precursors were stable when stored at −20 °C for more than six months.

### Scope of the synthesis

Following its development, the synthetic route was used to synthesize a diverse set of 2OG derivatives bearing aliphatic substituents at the 2OG C3- and/or C4-positions (Table 1, entries 1–21). The aliphatic substituents varied in both length and steric bulk of the carbon chain. Furthermore, 2OG derivatives were synthesized in which the C3/C4 ethylene unit of 2OG was replaced by rings including heteroaromatic rings (Table 1, entries 22 and 23), aromatic rings (Table 1, entries 24–31), and aliphatic bicyclic rings (Table 1, entry 32). A cyclopropane-containing 2-oxoacid that was not based on the glutarate C5 skeleton but on the succinate C4 skeleton (48; Table 1, entry 33), was synthesized.

**Table 1:** Inhibition of AspH by 2OG derivatives

	2OG derivative^a^	IC_50_^b^^,^^c^ [μM]		2OG derivative^a^	IC_50_^b^^,^^c^ [μM]		2OG derivative^a^	IC_50_^b^^,^^c^ [μM]
1	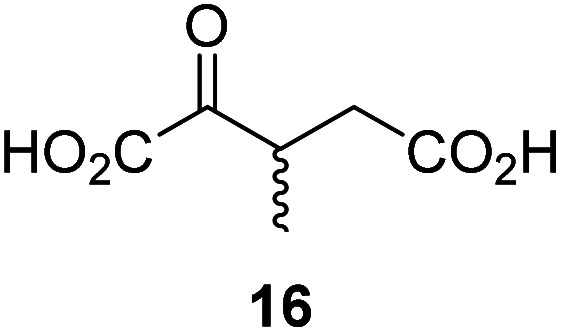	Inactive	12	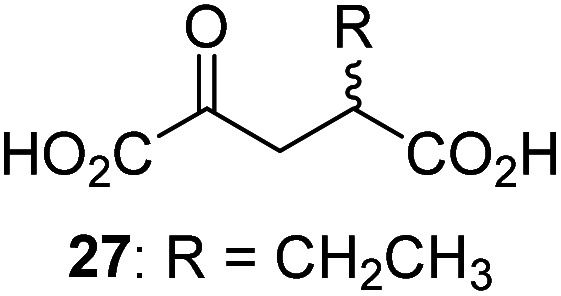	0.61 ± 0.09	23	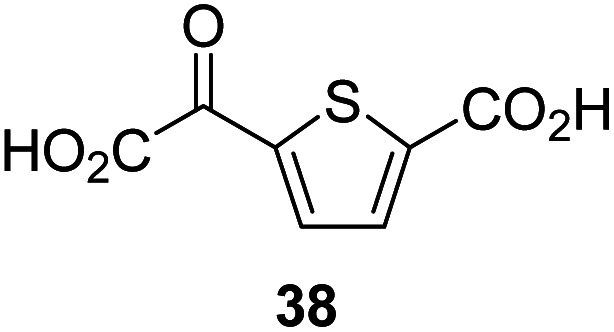	12.9 ± 1.3
2	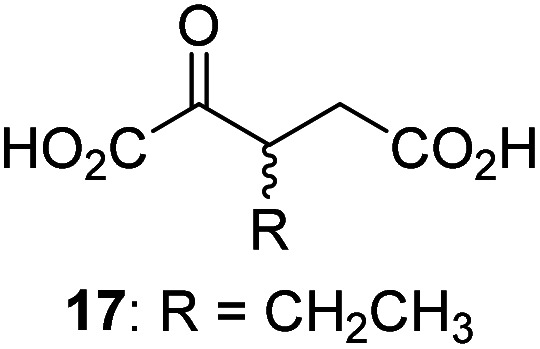	1.2 ± 0.5	13	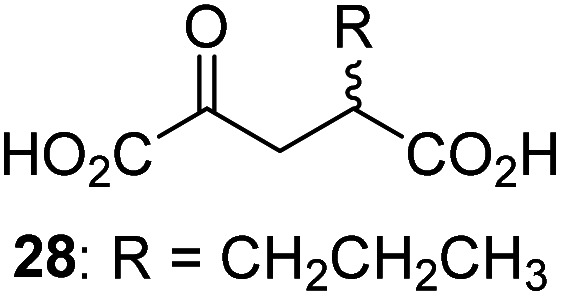	0.47 ± 0.08	24	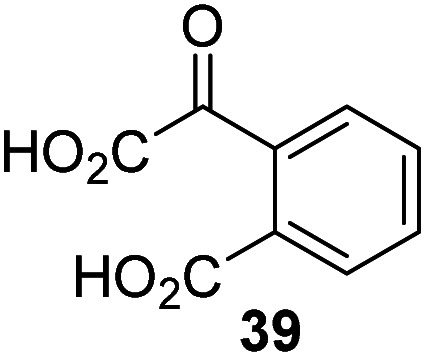	Inactive
3	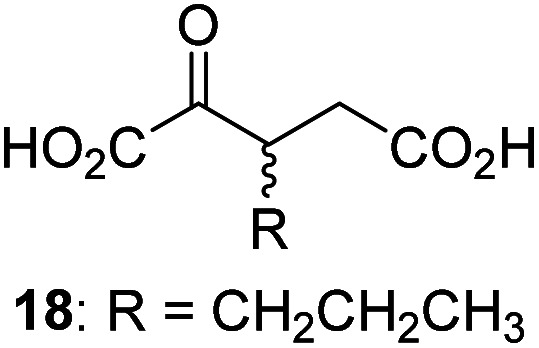	5.7 ± 1.1	14	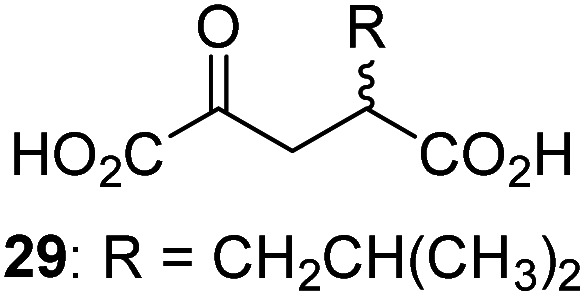	0.51 ± 0.12	25	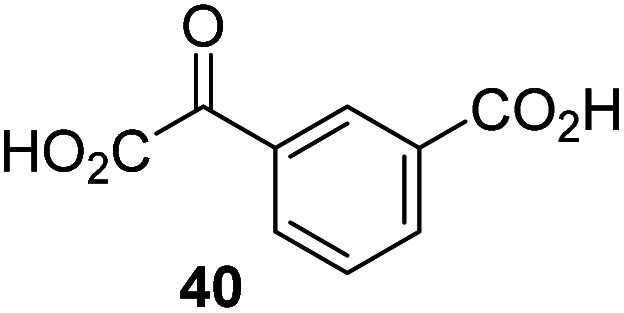	Inactive
4	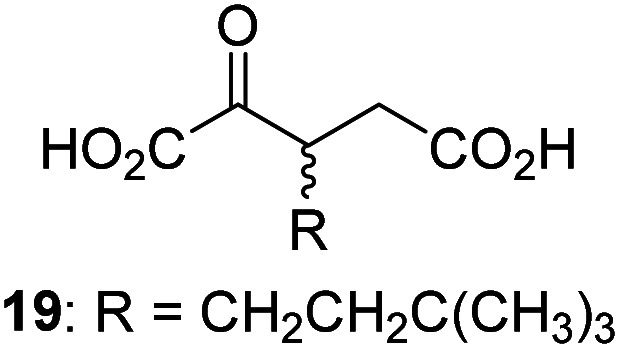	48.2 ± 13.1	15	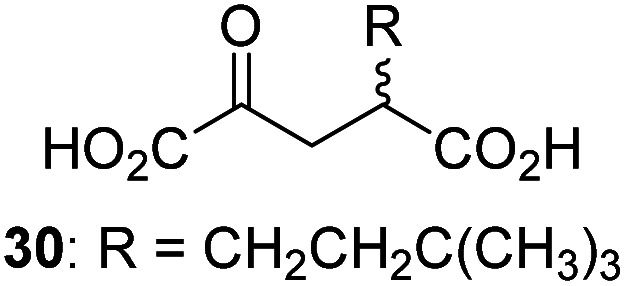	0.70 ± 0.11	26	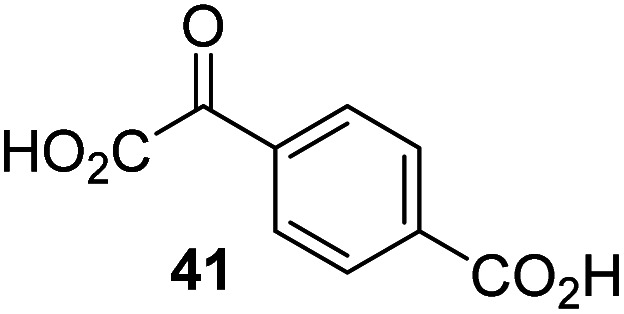	Inactive
5	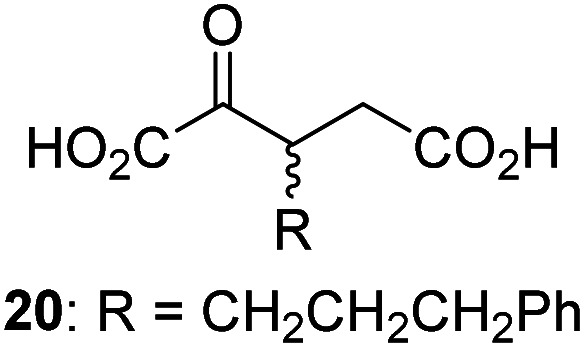	6.8 ± 0.9	16	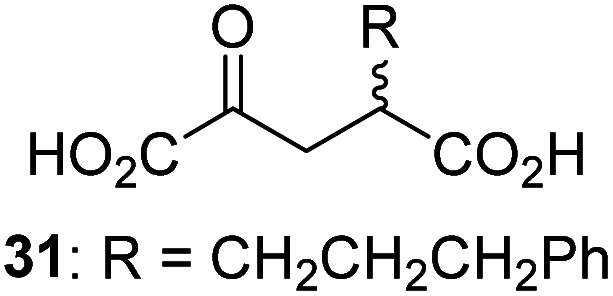	0.25 ± 0.05	27	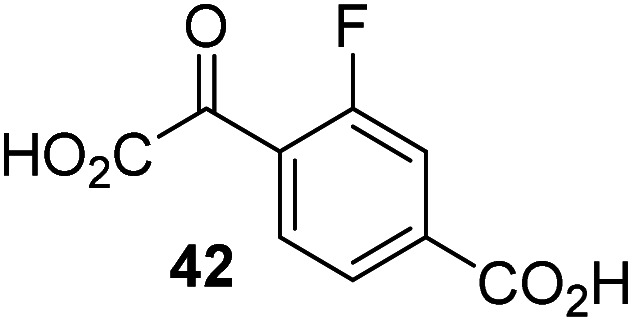	Inactive
6	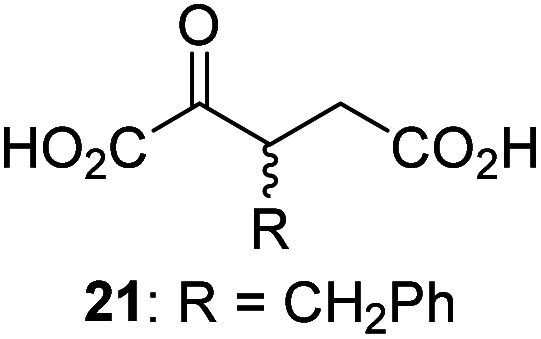	1.6 ± 0.3	17	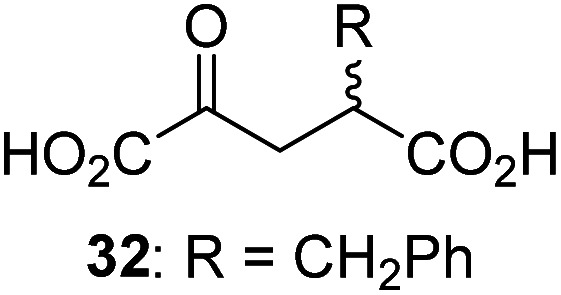	0.43 ± 0.05	28	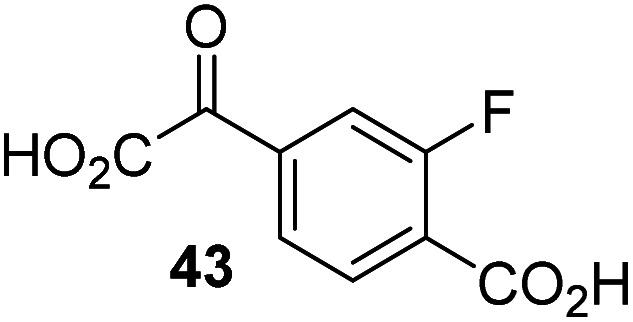	Inactive
7	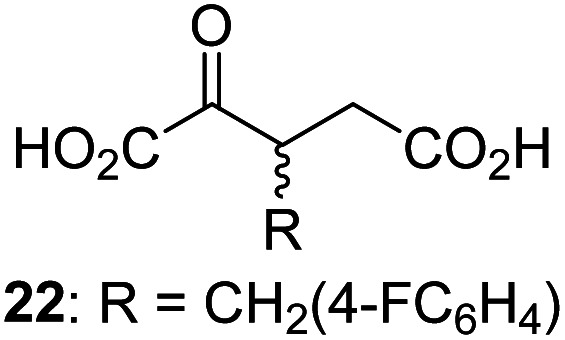	2.6 ± 0.8	18	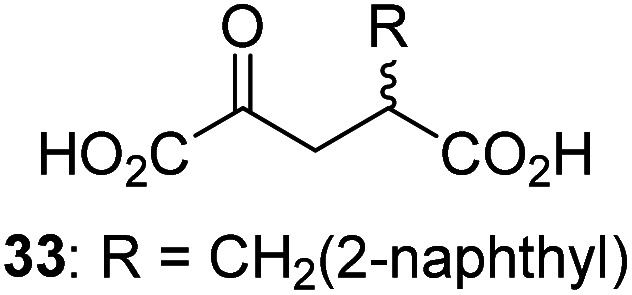	0.17 ± 0.03	29	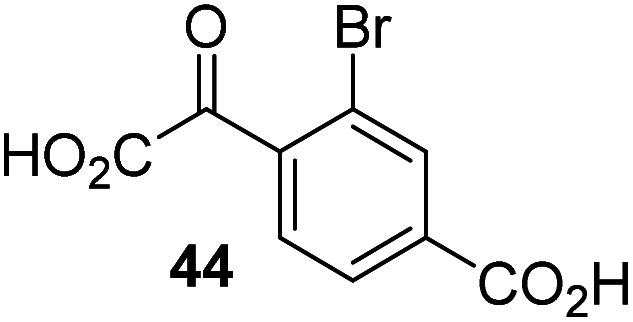	Inactive
8	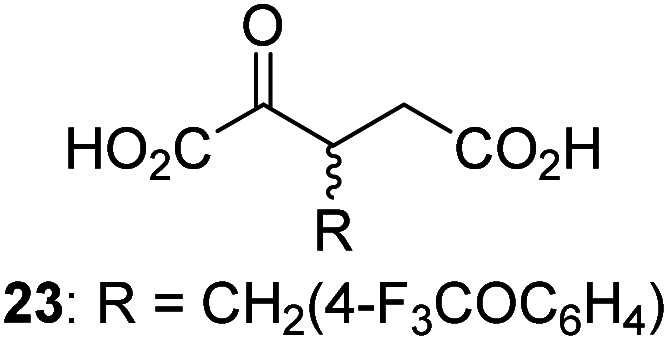	6.3 ± 2.6	19	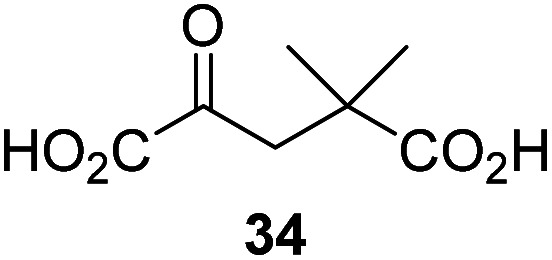	0.3 ± 0.1	30	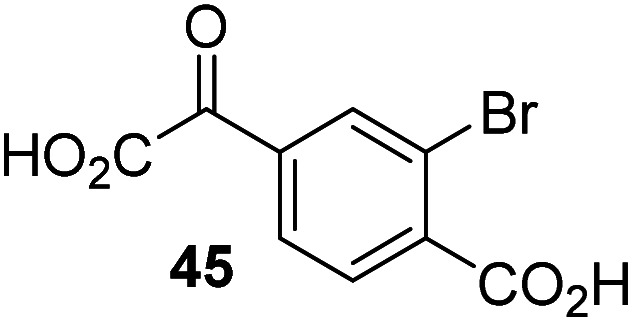	Inactive
9	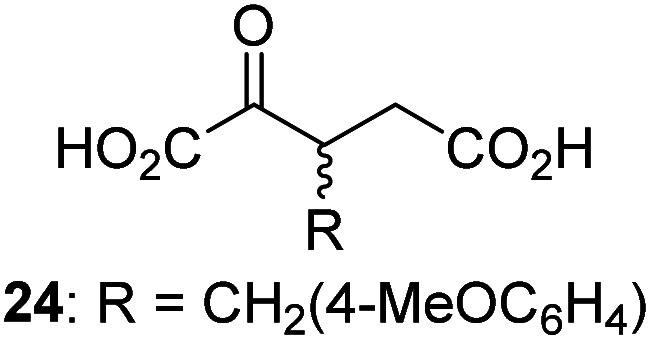	3.6 ± 1.4	20^d^	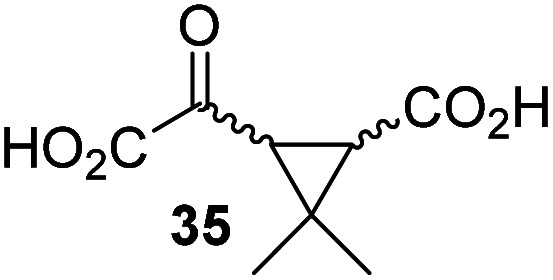	5.2 ± 1.7	31	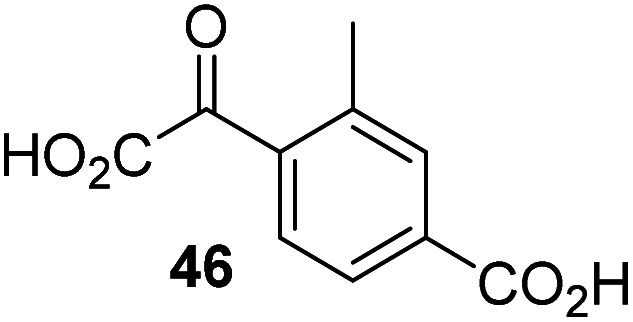	Inactive
10	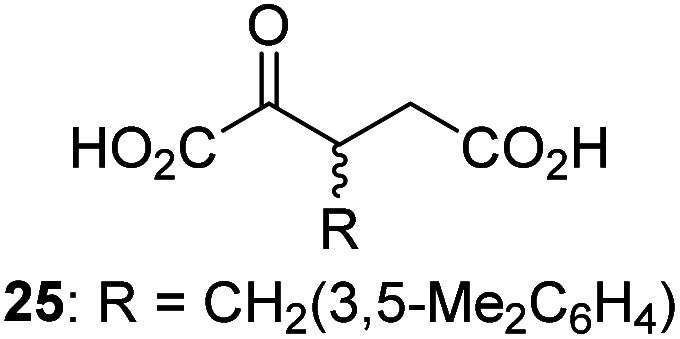	4.7 ± 0.2	21^e^	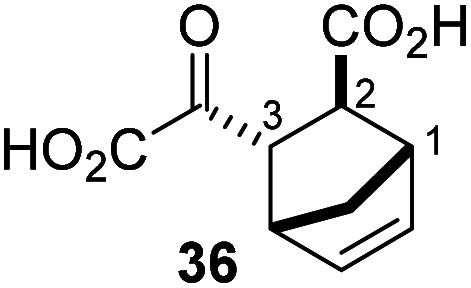	19.3 ± 1.6	32	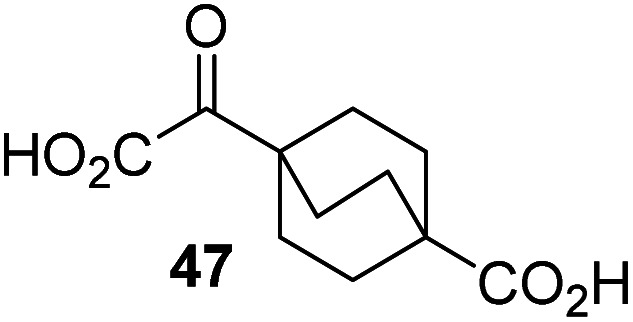	Inactive
11	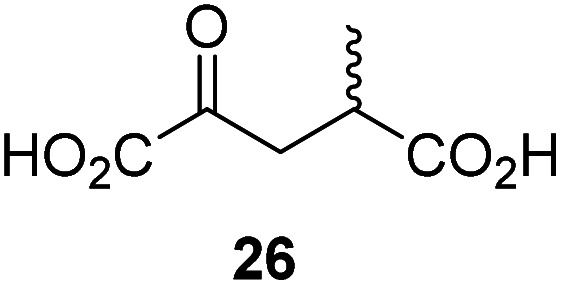	Inactive	22	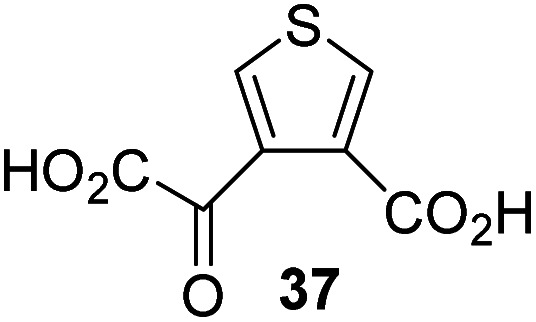	Inactive	33	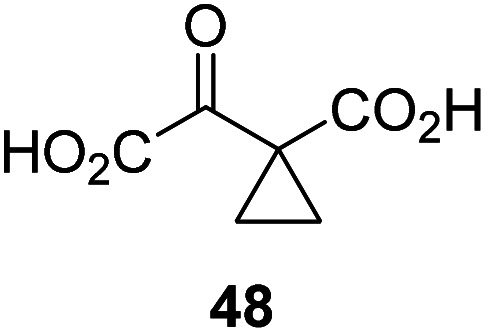	3.3 ± 1.0

a All chiral 2OG derivatives were prepared as racemic mixtures.

b Mean of three independent runs (*n* = 3; mean ± SD). AspH inhibition assays were performed as described in the ESI using 50 nM His_6_-AspH_315–758_ and 1.0 μM hFX-CP_101–119_ (ESI Fig. S1d) as a substrate.

c 2OG derivatives were termed inactive when the IC_50_-values were >50 μM. The AspH inhibition assays were of good quality which high S/N ratios and *Z*′-factors^40^ (>0.5 for each plate) indicate (ESI Fig. S3).

d Mixture of racemic diastereomers, dr (*cis* : *trans*) = 2.5 : 1.

e (±)-(2-*Exo*,3-*endo*)-diastereomer.

The synthesis of 2OG derivatives bearing acid-labile or some oxidation-prone moieties was challenging. For example, during the cyanosulfur ylid oxidation reaction, both ketal and silyl ether alcohol protecting groups were cleaved and nitrogen-containing heteroaromatic rings (*e.g.* pyridines) formed *N*-oxides. Nonetheless, the oxidation conditions were sufficiently mild to tolerate alkenes (36; Table 1, entry 21) and substituted thiophenes (37 and 38; Table 1, entries 22 and 23), which constitutes an advantage compared to many prior syntheses of 2OG derivatives (Fig. 2).

### AspH inhibition studies

We then evaluated the potential of the synthesized racemic 2OG derivatives to inhibit AspH by measuring AspH substrate depletion and product formation (*i.e.* by monitoring a +16 Da mass shift) using an established solid phase extraction coupled to mass spectrometry (SPE-MS) AspH inhibition assay.^41^ Half maximum inhibitory concentrations (IC_50_-values) for all the synthetic 2OG derivatives prepared were determined (Table 1).

3-Methyl-2OG (16) did not inhibit AspH (*i.e.* IC_50_ > 50 μM), while 2OG derivatives bearing longer carbon chains at the C3-position, including 3-ethyl-2OG (17), were efficient inhibitors, under the assay conditions. The inhibitory potency decreased on increasing the length or steric bulk of the carbon chain of the C3-substituent beyond that of an ethyl group, *i.e.* C3–Et (17): IC_50_ ∼ 1.2 μM *vs.* C3–Pr (18): IC_50_ ∼ 5.7 μM, C3–CH_2_Ph (21): IC_50_ ∼ 1.6 μM *vs.* C3–CH_2_CH_2_CH_2_Ph (20): IC_50_ ∼ 6.8 μM, and C3–CH_2_CH_2_CH_2_Ph (20): IC_50_ ∼ 6.8 μM *vs.* C3–CH_2_CH_2_C(CH_3_)_3_ (19): IC_50_ ∼ 48.2 μM. For the C3 benzyl substituted 2OG derivatives 21–25 with differently substituted phenyl rings, the substitution pattern on the phenyl rings appears to not affect the inhibitor potency within experimental error (Table 1, entries 6–10). With the exception of 4-methyl-2OG (26), which did not inhibit AspH (Table 1, entry 11), 2OG derivatives bearing substituents at the 2OG C4-position (Table 1, entries 12–18) were substantially more potent in inhibiting AspH than those bearing substituents at the C3-position: their relative potencies increased by a factor of 2 (for C3/4-Et) to ∼70 (for C3/4-CH_2_CH_2_C(CH_3_)_3_). The IC_50_-values of the C4-substituted 2OG derivatives range between 0.2 and 0.7 μM (Table 1, entries 12–18) and did not appear to depend on length or bulk of the tested C4-substituents. The C4-substituted 2OG derivatives inhibit AspH with comparable efficiency as the broad-spectrum 2OG oxygenase inhibitor NOG (3; IC_50_ ∼ 1.0 μM; ESI Fig. S4†),^41*a*^ but less efficiently than pyridine-2,4-dicarboxylate (2,4-PDCA; IC_50_ ∼ 0.03 μM),^41*a*^ which is another broad-spectrum 2OG oxygenase inhibitor.

4,4-Dimethyl-2OG (34) was an efficient AspH inhibitor (IC_50_ ∼ 0.3 μM; Table 1, entry 19), whilst the more bulky bicyclo[2.2.2]octane-bearing 2OG derivative 47 did not inhibit (Table 1, entry 32). The dimethylcyclopropane-bearing 2OG derivative 35 (2.5 : 1 mixture of racemic *cis*/*trans*-diastereomers) inhibits (IC_50_ ∼ 5.2 μM; Table 1, entry 20), albeit less efficiently than 34. This might reflect the reduced rotational flexibility of 35 around the C3–C4 bond due to the presence of the cyclopropane ring. 2OG derivative 36, which is also C3/C4-disubstituted, but which is significantly more bulky than 35, inhibits AspH (IC_50_ ∼ 19.3 μM), but substantially less efficiently than does 35 (Table 1, entry 21). The thiophene-based 2OG derivative 37 did not inhibit AspH (Table 1, entry 22). By contrast, the regioisomeric thiophene-based 2OG derivative 38 inhibited AspH with moderate efficiency (IC_50_ ∼ 12.9 μM; Table 1, entry 23). This observation might reflect the ability of 38, but likely not of 37 to better chelate Fe(ii), including that at the AspH active site. Several Fe(ii)-chelators have been previously identified to inhibit AspH in inhibitor screens.^41*a*^

The phenyl ring regioisomers 39, 40, and 41, which are 2OG derivatives bearing an aromatic core, did not inhibit AspH (Table 1, entries 24–26), as it was the case for the other tested phenyl ring containing 2OG derivatives 42–46 (Table 1, entries 27–31). The 2OG derivative 48 whose carbon scaffold was not based on glutarate, but derived from succinate, inhibited AspH with moderate efficiency (IC_50_ ∼ 3.3 μM; Table 1, entry 33).

Interestingly, 4-benzyl-2OG (32) inhibits AspH significantly more efficiently (IC_50_ ∼ 0.4 μM; Table 1, entry 17) than its *N*-oxalyl analogue *N*-oxalyl-d-phenylalanine (NOFD, IC_50_ ∼ 15.5 μM),^41*a*^ which is a reported inhibitor of human FIH.^24^ An opposite trend was observed for FIH, for which NOFD was a substantially more efficient inhibitor than 32,^24^ revealing the context dependent effect of the same 2OG substitutions. The structures of these two inhibitors are very similar: the C3 methylene-unit of 4-benzyl-2OG (32) is substituted for an NH-group in NOFD;^24^ however, 32 was prepared as a racemic mixture whereas NOFD was used in enantiopure d-form. To investigate the effect which the NH-group present in NOG and NOFD imposes on AspH inhibition, while excluding possible interference from the stereochemistry of the inhibitors (including with respect of C3-racemisation of the chiral 2-oxoacids), a derivative of 4,4-dimethyl-2OG (34) was thus synthesized in which the C3 methylene-unit was replaced with an NH-group (*N*-oxalyl-α-methylalanine, 49; ESI Fig. S4†). 34 inhibits AspH approximately ten times more efficiently (IC_50_ ∼ 0.3 μM; Table 1, entry 19) than 49 (IC_50_ ∼ 2.9 μM; ESI Fig. S4†). This observation could reflect the higher conformational flexibility of 34 and/or the higher stability of the AspH:34 complex.

### 2OG derivatives compete with 2OG for binding the AspH active site

**Fig. 3:**
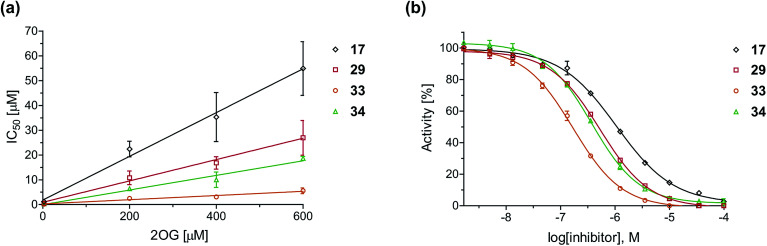
Inhibition of AspH by 2OG derivatives. (a) The AspH IC_50_-values for the 2OG derivatives 17 (black diamonds), 29 (red squares), 33 (orange circles), and 34 (green triangles) depend on the 2OG concentration. AspH inhibition assays were performed in triplicate as described in the ESI† using 3, 200, 400, and 600 μM 2OG. IC_50_-values are summarized in ESI Table S1;† (b) representative dose–response curves used to determine IC_50_-values for the 2OG derivatives 17 (black diamonds), 29 (red squares), 33 (orange circles), and 34 (green triangles) at a 2OG assay concentration of 3 μM 2OG. Three dose–response curves each composed of technical duplicates were independently determined using SPE-MS AspH inhibition assays, performed as described in the ESI† and manifest high *Z*′-factors^40^ and signal-to-noise ratios (ESI Fig. S3†).

To define whether the mechanism by which the 2OG derivatives inhibit AspH involves competition with 2OG for binding to the AspH active site, the effect of altered 2OG concentrations on the IC_50_-values of AspH was investigated. The IC_50_-values of four potent AspH inhibitors (*i.e.*17, 29, 33, and 34) were determined at 2OG assay concentrations of 3, 200, 400, and 600 μM (Fig. 3a; ESI Table S1†). The results reveal an ascending linear dependence of the IC_50_-values on the 2OG assay concentration suggesting that the 2OG derivatives inhibit AspH by competing with 2OG for binding to the active site. This is in agreement with a Hill coefficient^42^ analysis of the AspH inhibition curves which indicates that the 2OG derivatives did not inhibit AspH by forming colloidal aggregates; the Hill coefficients are in the range of the expected ‘ideal’ value −1 (Fig. 3b).^43^

Neither the position nor the size of the C3/C4-substituent of the 2OG derivatives had a detrimental effect on the linear dependence of their AspH IC_50_-values on the 2OG concentration (Fig. 3a). Efficient inhibition of AspH at higher 2OG assay concentrations was observed for 2OG derivatives 33 and 34 (IC_50_ ∼ 5.6 and 18.7 μM at 0.6 mM 2OG assay concentration, respectively; ESI Table S1,† entries 3 and 4).

### Some 2OG derivatives can replace 2OG as an AspH cosubstrate

**Fig. 4:**
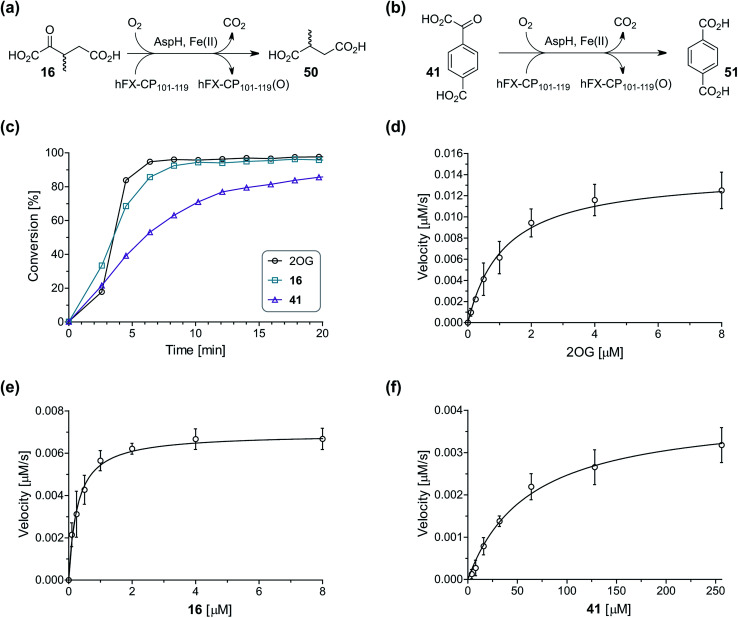
AspH steady-state kinetic parameters for selected 2OG derivatives measuring initial hydroxylation rates of a synthetic thioether linked cyclic peptide AspH substrate. (a) AspH catalyzes the oxidative decarboxylation of 3-methyl-2OG (16) to give 2-methylsuccinate (50); (b) AspH catalyzes the oxidative decarboxylation of 4-carboxyphenylglyoxylic acid (41) to give terephthalate (51); (c) the 2OG derivatives 16 and 41 replace 2OG as an AspH cosubstrate, as monitoring AspH substrate hydroxylation manifests. AspH assays were performed as described in the ESI† using 0.1 μM AspH, 2 μM hFX-CP_101–119_ (ESI Fig. S1d†), 50 μM FAS, 330 μM 2OG or 2OG derivative in 50 mM HEPES (pH 7.5, 20 °C); (d) *K*^app^_m_ of AspH for 2OG; (e) *K*^app^_m_ of AspH for 16; (f) *K*^app^_m_ of AspH for 41. AspH assays were performed as described in the ESI,† data are shown as the mean of three independent runs (*n* = 3; mean ± standard deviation, SD). The results are summarized in Table 2 and the peptide hydroxylation rates are shown in ESI Fig. S6.†

During the assessment of the AspH inhibition data, we observed that in the presence of two 2OG derivatives, *i.e.* 3-methyl-2OG (16) and 4-carboxyphenylglyoxylic acid (41), the extent of AspH-catalyzed substrate hydroxylation appeared to increase. We proposed that these 2OG derivatives could replace 2OG and function as alternative cosubstrates for AspH. AspH substrate hydroxylation was thus investigated in the absence of 2OG: high levels of AspH substrate hydroxylation were observed at elevated concentrations of the 2OG derivatives 16 (>95%) and, somewhat unexpectedly, the phenyl ring derivative 41 (∼80%) in the absence of 2OG, demonstrating that these two 2OG derivatives can replace 2OG as an AspH cosubstrate (Fig. 4a–c). NMR studies revealed that AspH converts the 2OG derivatives 16 and 41 into 2-methylsuccinate (50) and terephthalate (51), respectively, in an analogous manner to which it converts 2OG into succinate (Fig. 4a–c; ESI Fig. S5†).

To investigate whether the other synthesized 2OG derivatives can substitute for 2OG as a cosubstrate for AspH-catalyzed hydroxylation, assays were performed in the absence of 2OG, but in the presence of high concentrations (330 μM) of all the synthetic 2OG derivatives (ESI Table S2†). In addition to the 2OG derivatives 16 and 41, AspH substrate hydroxylation was observed for the 2OG derivatives 26 (∼10%), 38 (∼15%), 42 (∼45%), 43 (∼8%), 44 (∼20%), 45 (∼8%), 46 (∼5%), and 47 (∼8%) after 15 minutes. These results reveal that 3-methyl-2OG (16) is a substantially more efficient alternative AspH cosubstrate than its isomer 4-methyl-2OG (26), an observation in accord with the observation that C3-substituted 2OG derivatives are generally less efficient AspH inhibitors than their corresponding C4-substituted isomers (Table 1).

Increasing the steric bulk of the 2OG derivative, while maintaining the relative arrangement of the two carboxylate groups, decreases the catalytic efficiency of the cosubstrate analogue, as revealed by the comparison of the phenyl-ring 2OG derivative 41 (∼80%) with its bridged bicyclo[2.2.2]octane analogue 47 (∼8%). Derivatives of 41 bearing substituents *ortho* to the ketone (*e.g.* as in 42, ∼45%) seemed to be more efficient AspH cosubstrates than those bearing substituents *meta* to the ketone (*e.g.* as in 43, ∼8%). Increasing the size of the substituents *ortho* to the ketone of 2OG derivative 41 (*i.e. ortho*-F, 42; *ortho*-Br, 44; *ortho* methyl 46) results in a noticeable decrease in the efficiency to replace 2OG as an AspH cosubstrate (*i.e.*42: ∼45%; 44: ∼20%; 46: ∼5%); all derivatives of 41 were significantly less efficient with respect to the parent compound (∼80%).

Kinetic analyses of the two most efficient 2OG substitute AspH cosubstrates identified (*i.e.*16 and 41) were performed. Maximum velocities (*v*^app^_max_) and Michaelis constants (*K*^app^_m_) were determined employing SPE-MS turnover assays, albeit under modified conditions than previously reported for 2OG as a cosubstrate (Fig. 4d–f),^23^ as l-ascorbic acid (LAA), which is commonly added to 2OG oxygenase assays, was included in the assay buffer. The presence of LAA affected the kinetic parameters for 2OG when compared to the previous parameters (Table 2, entry 1). The *v*^app^_max_ (2OG) did not change significantly, being ∼16.8 × 10^−3^ μM s^−1^ in the absence of LAA^23^ and ∼15.0 × 10^−3^ μM s^−1^ in the presence of LAA. The *K*^app^_m_ (2OG)-value in the presence of LAA was approximately double (∼1.3 μM) compared to that in the absence of LAA (∼0.6 μM).^23^ However, this *K*^app^_m_ (2OG)-value is still in the range of those values reported for most other human 2OG oxygenases including for the HIF-α prolyl hydroxylases and FIH (1–25 μM)^44^ and bovine AspH (∼5 μM).^45^

**Table 2:** Steady-state kinetic parameters for AspH. Maximum velocities (*v*^app^_max_), Michaelis constants (*K*^app^_m_), turnover numbers (*k*_cat_), and specificity constants (*k*_cat_/*K*_m_) of His_6_-AspH_315–758_ for 2OG and the 2OG derivatives 16 and 41^a^^,^^b^

	AspH co-substrate	*v* ^app^_max_ [μM s^−1^]	*K* ^app^_m_ [μM]	*k*_cat_ [s^−1^]	*k*_cat_/*K*_m_ [μM^−1^ s^−1^]
1	2OG	15.0 × 10^−3^ ± 0.9 × 10^−3^	1.3 ± 0.3	0.17 ± 0.03	0.13 ± 0.03
2	3-Methyl-2OG (16)	6.9 × 10^−3^ ± 0.2 × 10^−3^	0.27 ± 0.04	0.08 ± 0.01	0.30 ± 0.06
3	4-Carboxyphenylglyoxylic acid (41)	4.0 × 10^−3^ ± 0.3 × 10^−3^	61.9 ± 11.0	0.04 ± 0.01	0.65 × 10^−3^ ± 0.2 × 10^−3^

a Mean of three independent runs (*n* = 3; mean ± SD).

b AspH assays were performed as described in the ESI using 0.1 μM His_6_-AspH_315–758_ and 2.0 μM hFX-CP_101–119_ (ESI Fig. S1d) as a substrate.

Compared to the *K*^app^_m_-value of AspH for 2OG (∼1.3 μM; Table 2, entry 1), the *K*^app^_m_-value for 3-methyl-2OG (16) was about five times lower (∼0.3 μM; Table 2, entry 2), indicating a higher affinity of AspH for 16 compared to 2OG. By contrast, the *K*^app^_m_-value of AspH for the 2OG derivative 41 was ∼47 times higher (∼62 μM; Table 2, entry 3) than that for 2OG, indicating much less efficient binding. All three *K*^app^_m_-values range significantly below reported 2OG concentrations in healthy cells (up to >1 mM),^46^ which however vary substantially, but are in the approximate range of reported physiological 2OG levels in human plasma (9–12 μM 2OG).^47^

Based on the determined concentration of active AspH (90.8 ± 13.7 nM for an original estimated AspH assay concentration of 100 nM AspH),^23^ turnover numbers (catalytic constants, *k*_cat_) and specificity constants (*k*_cat_/*K*_m_) were calculated for the 2OG derivatives (Table 2). Comparison of the *k*_cat_-values reveals that the impact of the 2OG derivatives on *k*_cat_-values is notably smaller than on *K*^app^_m_-values which indicates that efficient AspH-catalyzed substrate hydroxylation is still feasible with the 2OG derivatives (Table 2): for AspH, the *k*_cat_-value for 16 (∼0.08 s^−1^; Table 2, entry 2) was about half the *k*_cat_ for 2OG (∼0.17 s^−1^; Table 2, entry 1), whereas the *k*_cat_ for 41 (∼0.04 s^−1^; Table 2, entry 3) was about a quarter of that for 2OG. Comparison of the *k*_cat_/*K*_m_-values clearly reveals the potential of 2OG derivatives to substitute for 2OG itself, with racemic 16 being of similar efficiency to 2OG. Although 41 is a much less efficient substrate, its conversion reveals the potential for unexpected cosubstrate utilization by 2OG oxygenases.

### Crystallography

The AspH turnover assays indicated that the synthetic 2OG derivatives compete with 2OG for binding to the AspH active site. To investigate the divergent effects of C3/C4-substituted 2OG derivatives on AspH catalysis, *i.e.* AspH inhibition or promoting AspH activity, crystallographic studies were initiated. In the reported AspH crystal structures, the natural AspH cosubstrate 2OG was substituted for a 2OG competing inhibitor (*e.g.* NOG, 2,4-PDCA or l-malate),^22,41*a*^ but an AspH crystal structure complexed with 2OG has not previously been reported.

**Fig. 5:**
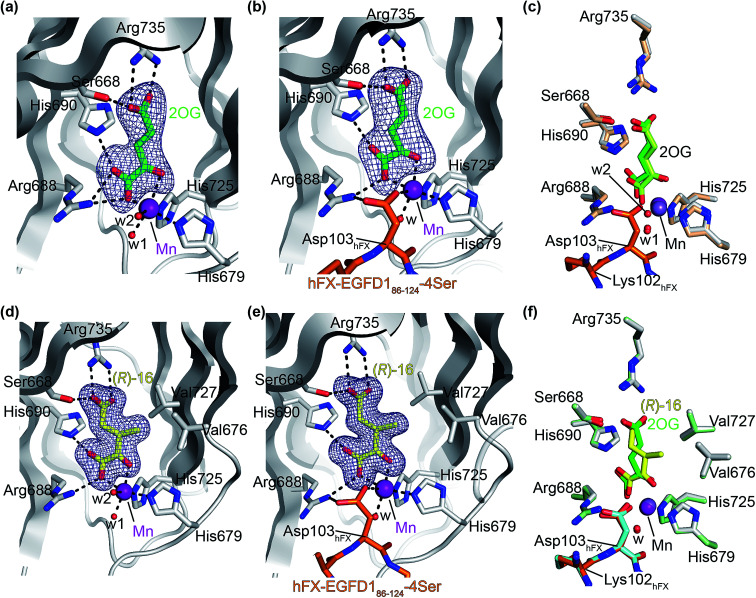
The conformations of 3-methyl-2OG (16) and 2OG differ at the AspH active site. Colors: grey: His_6_-AspH_315–758_; green: carbon-backbone of 2OG; yellow: carbon-backbone of (*R*)-16; violet: Mn; orange: carbon-backbone of the hFX-EGFD1_86–124_-4Ser peptide (ESI Fig. S1c†); red: oxygen; blue: nitrogen; w: water. (a) Representative OMIT electron density map (mF_o_-DF_c_) contoured to 5*σ* around 2OG of the AspH:2OG structure; (b) representative OMIT electron density map (mF_o_-DF_c_) contoured to 5*σ* around 2OG of the AspH:2OG:hFX-EGFD1_86–124_-4Ser structure. The 2OG C5-carboxylate is positioned to salt bridge with the Arg735 side chain (2.5 and 2.9 Å) and to interact with Ser668 (2.5 Å). 2OG is positioned to interact with His690 (2.9 Å) and Arg688 (2.8 Å) through its C1-carboxylate, and with the Mn ion *via* its C1-carboxylate (2.2 Å) and C2-carbonyl (2.3 Å). The Mn ion is also complexed by His679 (2.2 Å), His725 (2.1 Å), a water molecule (2.1 Å), and the C4-carboxylate of the Asp103_hFX_ peptide substrate (2.4 Å); (c) superimposition of views from the AspH:2OG:hFX-EGFD1_86–124_-4Ser structure with one from the AspH:2OG structure (beige: His_6_-AspH_315–758_; lavender: Mn ion; lemon: carbon-backbone of 2OG); (d) representative OMIT electron density map (mF_o_-DF_c_) contoured to 5*σ* around (*R*)-16 of the AspH:16 structure; (e) representative OMIT electron density map (mFo-DFc) contoured to 5*σ* around (*R*)-16 of the AspH:16:hFX-EGFD1_86–124_-4Ser structure; (f) superimposition of views from the AspH:16:hFX-EGFD1_86–124_-4Ser structure with one from the AspH:2OG:hFX-EGFD1_86–124_-4Ser structure (pale green: His_6_-AspH_315–758_; green: carbon-backbone of 2OG; lavender: Mn ion; aquamarine: carbon-backbone of hFX-EGFD1_86–124_-4Ser).

To enable comparisons of how 2OG and the 2OG derivatives bind AspH, AspH was first crystallized in the presence of 2OG with the natural AspH cofactor Fe(ii) being replaced by Mn(ii). AspH crystallized in the absence of substrate in the *P*2_1_2_1_2_1_ space group (AspH:2OG; 2.1 Å resolution), the structure, as were the subsequently described structures, was solved by molecular replacement using a reported AspH structure (PDB ID: 5JZA)^22^ as a search model (ESI Fig. S7†). Clear electron density corresponding to 2OG was observed (Fig. 5a); the C5-carboxylate of 2OG being positioned to form a salt bridge with the side chain of Arg735 (2.4 and 3.0 Å) and to interact with Ser668 (2.6 Å), which is part of an ‘RXS motif’ present in some other 2OG oxygenases.^48^ The C1-carboxylate of 2OG is positioned to interact with Arg688 (2.7 and 2.9 Å) and His690 (3.3 Å). The C1-carboxylate of 2OG and the 2OG C2-carbonyl group complex the Mn ion in a bidentate manner (1.6 and 2.5 Å; Fig. 5a). Two water molecules also coordinate the Mn ion (2.1 and 2.4 Å) along with the two anticipated residues His679 and His725 (2.2 and 2.1 Å; Fig. 5a). Thus, the AspH:2OG structure supports the proposal (based on the AspH structures in complex with the 2OG analogue NOG^22^) that the active site metal, when bound to the natural AspH cosubstrate 2OG, is complexed by only two AspH residues rather than by the typical triad of ligands (HXD/E⋯H) found in other human 2OG hydroxylases.^7,14^ Superimposition of the AspH:2OG structure with the reported AspH:NOG^22^ and AspH:l-malate^22^ structures reveals that AspH adopts similar conformations in all structures and that 2OG binds the AspH active site in a similar manner to NOG (Cα RMSD = 0.21 and 0.21 Å, ESI Fig. S7†).

To investigate the effects of substrate binding in the presence of 2OG, AspH was crystallized in the presence of 2OG, Mn(ii), and the synthetic hFX-EGFD1_86–124_-4Ser substrate^22^ (ESI Fig. S1c†), which mimics the EGFD1 of the reported AspH substrate human coagulation factor X (hFX).^49^ The structure was solved by molecular replacement using a reported structure (PDB ID: 5JTC)^22^ as a search model (*P*2_1_2_1_2_1_ space group; 2.3 Å resolution). The active site region manifested electron density for both 2OG (Fig. 5b) and for the hFX-EGFD1_86–124_-4Ser peptide (ESI Fig. S8†). Substrate binding to AspH affects the relative alignment of the oxygenase and TPR domains, *i.e.* the distance between the Cα atoms of Leu433 on TPR repeat α6 and Pro756 in the AspH C terminal region decreases from ∼20 Å to ∼14 Å upon substrate binding (ESI Fig. S9†). Evidence for an induced fit substrate binding mechanism involving this conformational change has been described when the cosubstrate 2OG was substituted for NOG.^22^ The significant conformational changes in the AspH oxygenase domain triggered by substrate binding (ESI Fig. S10†) do not affect the observed mode of 2OG binding in the active site (Fig. 5c); the binding modes of both 2OG and NOG are very similar, both in the presence or absence of substrate (ESI Fig. S9†). Interestingly, whilst in the 2OG complex structure the substrate residue Asp103_hFX_ was observed in a single conformation whereas in the analogous NOG structure it was observed in two conformations (ESI Fig. S9†).

High resolution crystal structures of AspH complexed with 3-methyl-2OG (16) and Mn(ii), both with and without the hFX-EGFD1_86–124_-4Ser substrate bound, were obtained (AspH:16:hFX-EGFD1_86–124_-4Ser, ESI Fig. S11;† AspH:16, ESI Fig. S12;† 1.5 and 1.8 Å resolution, respectively). Substitution of 2OG for 16 did not trigger significant changes in the AspH conformations (superimposition of the AspH:2OG and the AspH:16 structures: Cα RMSD = 0.38 Å; superimposition of the AspH:2OG:hFX-EGFD1_86–124_-4Ser and the AspH:16:hFX-EGFD1_86–124_-4Ser structures: Cα RMSD = 0.23 Å; ESI Fig. S12 and S13†). A similar change in the relative alignment of the AspH oxygenase and TPR domains upon substrate binding was observed when 2OG was substituted for 16 (ESI Fig. S13†).

Although 3-methyl-2OG (16) was prepared and used as a racemic mixture, the electron density map corresponded to the, at least predominant, presence of the (*R*)-enantiomers at the active site in both the AspH:16 and AspH:16:hFX-EGFD1_86–124_-4Ser structures (Fig. 5d and e; ESI Fig. S11 and S12†). The carboxylate groups of the (*R*)-enantiomer of 16 interact with the same AspH residues as 2OG in both the AspH:16 and AspH:16:hFX-EGFD1_86–124_-4Ser structures. It is positioned to salt bridge with Arg735 (2.6/2.8 and 2.7/2.7 Å, respectively) with its C5-carboxylate and is positioned to interact with Ser668 (2.5 and 2.6 Å, respectively) through its C5-carboxylate, with His690 (2.8 and 2.8 Å, respectively) and Arg688 (2.8 and 2.9 Å, respectively) through its C1-carboxylate, and with Mn(ii) through its C1-carboxylate (2.2 and 2.1 Å, respectively) and C2-carbonyl groups (2.2 and 2.1 Å, respectively). Notably, superimposition of the AspH:16:hFX-EGFD1_86–124_-4Ser and AspH:2OG:hFX-EGFD1_86–124_-4Ser structures (ESI Fig. S12†) reveals that the (*R*)-enantiomer of 16 adopts a different conformation than 2OG at the active site. Whilst the oxalyl-groups of both 2OG and (*R*)-16 bind the metal in an identical manner and the two C5-carboxylate groups are superimposable, the C3- and C4-methylenes adopt different conformations (Fig. 5f). The C3-methyl group of (*R*)-16 faces towards AspH residues Val676 and Val727 (distance of the methyl C-atom of (*R*)-16 to the γ-methyl C-atoms of the Val residues: ∼3.5–4.6 Å; Fig. 5f), which together with Met670 form one face of a hydrophobic pocket, to which the indole ring of Trp625 also contributes (ESI Fig. S16c†).

The crystallographically observed different conformations which (*R*)-16 and 2OG occupy when bound to AspH may reflect the differences in their kinetic parameters. Thus, the hydrophobic interactions that the C3-methyl of (*R*)-16 forms with the side-chains of Val676 and Val727 might increase its binding affinity, in agreement with its *K*^app^_m_-value that is about five time lower than that for 2OG (Table 2). The *k*_cat_-value for (racemic) 16 is about half the *k*_cat_-value for 2OG (Table 2, entries 1 and 2), possibly reflecting the crystallographic evidence that the (*R*)- but not the (*S*)-enantiomer of 16 is a cosubstrate for AspH. It should also be noted that the (*S*)- and (*R*)-enantiomers of 16 will interconvert in aqueous media and in the presence of metal ions (ESI† Sections 4 and 5)^50^ and that, as (*R*)-16 is consumed in assays, this interconversion may become rate-limiting.

**Fig. 6:**
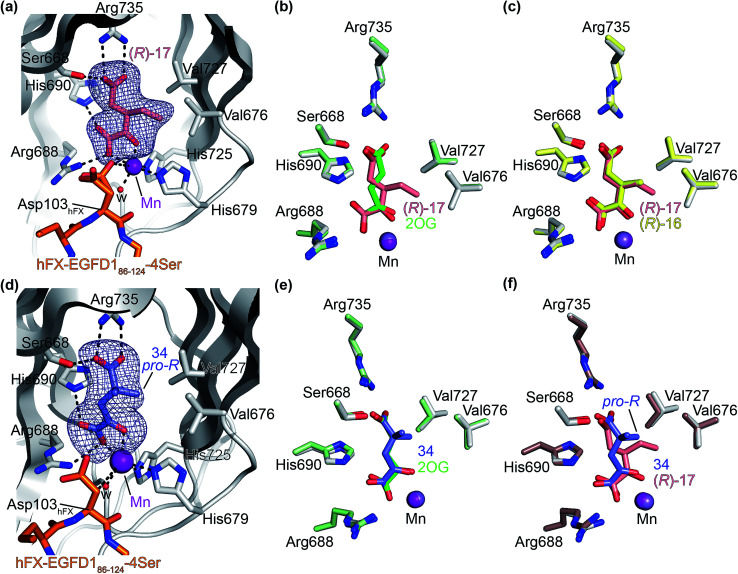
Binding of 3-ethyl-2OG (17) and 4,4-dimethyl-2OG (34) at the AspH active site. Colors: grey: His_6_-AspH_315–758_; salmon: carbon-backbone of (*R*)-17; slate blue: carbon-backbone of 34; violet: Mn; orange: carbon-backbone of the hFX-EGFD1_86–124_-4Ser peptide (ESI Fig. S1c†); red: oxygen; blue: nitrogen; w: water. (a) Representative OMIT electron density map (mF_o_-DF_c_) contoured to 3*σ* around (*R*)-17 of the AspH:17:hFX-EGFD1_86–124_-4Ser structure. (*R*)-17 binds similarly to (*R*)-16 (ESI Fig. S14a†); (b) superimposition of views of the AspH:17:hFX-EGFD1_86–124_-4Ser and the AspH:2OG:hFX-EGFD1_86–124_-4Ser (pale green: His_6_-AspH_315–758_; lavender: Mn ion; green: carbon-backbone of 2OG) structures; (c) superimposition of active sites views of the AspH:17:hFX-EGFD1_86–124_-4Ser and the AspH:16:hFX-EGFD1_86–124_-4Ser (pale yellow: His_6_-AspH_315–758_; lavender: Mn ion; yellow: carbon-backbone of (*R*)-16) structures; (d) representative OMIT electron density map (mF_o_-DF_c_) contoured to 3*σ* around 34 of the AspH:34:hFX-EGFD1_86–124_-4Ser structure. 34 binds similarly to 2OG (ESI Fig. S16a†); (e) superimposition of views of the AspH:34:hFX-EGFD1_86–124_-4Ser and the AspH:2OG:hFX-EGFD1_86–124_-4Ser (pale green: His_6_-AspH_315–758_; lavender: Mn ion; green: carbon-backbone of 2OG) structures; (f) superimposition of active site views of the AspH:34:hFX-EGFD1_86–124_-4Ser and the AspH:17:hFX-EGFD1_86–124_-4Ser (brown: His_6_-AspH_315–758_; lavender: Mn ion; salmon: carbon-backbone of 17) structures.

Next, AspH was crystallized in the presence of the inhibitor 3-ethyl-2OG (17); a crystal structure of AspH complexed with 17, Mn(ii), and the hFX-EGFD1_86–124_-4Ser substrate was obtained to 1.8 Å resolution (AspH:17:hFX-EGFD1_86–124_-4Ser, ESI Fig. S14†). As with 16, the results suggest that 17 competes with 2OG for binding the AspH active site, in agreement with inhibition assays with varied 2OG concentrations (Fig. 3a and ESI Table S1†). As for 16, the observed electron density corresponded to the presence of the (*R*)-enantiomer of 17 (Fig. 6a and ESI Fig. S14†), even though 17 was used as a racemic mixture during crystallization. The (*R*)-enantiomers of 17 and 3-methyl-2OG (16) occupy similar conformations when bound to the AspH active site (Fig. 6c), with their C3-substituents facing towards the side chains of Val676 and Val727. Superimposition of the AspH:17:hFX-EGFD1_86–124_-4Ser, the AspH:2OG:hFX-EGFD1_86–124_-4Ser, and the AspH:16:hFX-EGFD1_86–124_-4Ser structures reveals no significant changes in the AspH and the hFX-EGFD1_86–124_-4Ser substrate conformations (ESI Fig. S15†), and the presence of the different 2OG analogues does not appear to affect the conformations of AspH active site side-chain residues (Fig. 6b and c).

We also crystallized AspH in the presence of 4,4-dimethyl-2OG (34), Mn(ii), and the hFX-EGFD1_86–124_-4Ser substrate (2.3 Å resolution; ESI Fig. S16†). In this AspH:34:hFX-EGFD1_86–124_-4Ser structure, clear electron density for 34 was observed in the AspH active site (Fig. 6d). The *pro-R* methyl group of 34 projects towards one face of the hydrophobic pocket (Val626, Val727, Met670), whereas the *pro-S* methyl group of 34 is in proximity of Met670 and faces towards the indole ring of Trp625 (Fig. 6d and ESI Fig. S16c†).

While the AspH and the hFX-EGFD1_86–124_-4Ser substrate conformations of the AspH:34:hFX-EGFD1_86–124_-4Ser structure do not differ from those of previous structures (ESI Fig. S17†), the conformations of 34, of (*R*)-3-ethyl-2OG (17), and of *N*-oxalyl-α-methylalanine (49), which is a derivative of 34, differ substantially in the crystalline state (Fig. 6f, ESI Fig. S18 and S19†). By contrast, the conformations of 2OG and 34 are very similar (Fig. 6e); we therefore investigated by NMR if 34 is converted into 2,2-dimethylsuccinate, in the presence of substantially higher AspH concentrations compared to the original SPE-MS assay conditions (∼100 times more AspH). The results reveal that 34 indeed undergoes slow AspH-catalyzed oxidative decarboxylation, whereas another AspH inhibitor, 4-benzyl-2OG (32), was apparently not turned over under the same reaction conditions (ESI Fig. S20†).

Thus, the combined crystallographic analyses reveal that the C3/C4-substituted 2OG derivatives bind AspH in the same general manner as 2OG, with near identical binding modes for the oxalyl-groups and the C5-carboxylates. The different conformations for the C3- and C4-methylenes observed, however, do not correlate with catalysis *versus* inhibition (assuming the crystallographic binding modes reflect those in solution). Thus, whilst the C3- and C4-methylenes of (*R*)-16 and (*R*)-17 adopt a very similar conformation, which is distinct from that of 2OG and 34, 16 is a cosubstrate, whereas 17 is an inhibitor.

Hydrophobic interactions made by the C3-methyl and C3-ethyl groups of (*R*)-16 and (*R*)-17, respectively, and of one of the methyl groups of 34 with AspH, are consistent with the tighter binding of 16 as compared with 2OG and judged by *K*^app^_m_ comparison (Table 2). However, the *k*_cat_ for 16 is approximately half that of 2OG whereas 17 and 34 are inhibitors and/or poor substrates, respectively (Table 2); the reason for these differences is uncertain, but it may reflect slower binding of O_2_, or a slower subsequent step during catalysis, *e.g.* reaction of the ferryl intermediate proposed to be present in the 2OG oxygenase catalytic cycle or release of the succinate coproduct (ESI Fig. S21†). Given the reduced *k*_cat_ for 16, it is reasonable to propose that the ‘*k*_cat_’ for the C3-ethyl substituted inhibitor 17 will be reduced even further, potentially approaching zero, potentially as a consequence of particular strong interaction with the hydrophobic pocket.

## Discussion

The use of C3/C4-substituted 2OG derivatives has been a productive strategy to investigate *inter alia* the function, mechanism, and inhibition of aminotransferases^30,51^ and dehydrogenases^51*a*,51*b*,52^ that employ 2OG as a substrate or cosubstrate, but has been employed to a much lesser extent with 2OG oxygenases.^24–26^ By contrast with 2OG derivatives bearing major structural modifications of the 2-oxo-1,5-dicarboxylic acid scaffold,^53^ the synthetic accessibility of C3/C4-substituted 2OG derivatives has been hitherto limited, a factor which might have hampered detailed biochemical studies on their effects on 2OG oxygenases. To address this need, we developed an efficient synthesis of C3/C4-substituted 2OG derivatives relying on the use of cyanosulfur ylids^33*a*,34^ as key intermediates (Scheme 1). Our synthesis compares favorably to reported syntheses of 2OG derivatives (Fig. 2);^25,27–30^ it is scalable, affords the 2OG derivatives and their synthetic precursors in high purity suitable for biochemical applications, and avoids the use of strong bases, acids, and oxidants. The broad substrate scope of the synthesis reflects the mild reaction conditions; for example, 2OG derivatives bearing oxidation-prone olefins and thiophenes were readily synthesized (36–38; Table 1). No special laboratory equipment, such as an ozone generator, is required for the synthesis rendering it particularly user-friendly. We prepared racemic mixtures of C3/C4-substituted 2OG derivatives, however, the same synthetic strategy could be applied for the synthesis of enantiopure C4-substituted 2OG derivatives using enantiopure mono-methyl dicarboxylic acid half-esters 13 as starting materials, which, for example, can be obtained by asymmetric hydrogenation reactions from itaconates.^54^ The C3/C4-substituted 2OG derivatives were obtained as dicarboxylic acids (6, Scheme 1) and dimethyl dicarboxylates (12, Scheme 1). The former are useful for experiments with isolated enzymes as performed in this study, whereas the latter can be used for cell-based and *in vivo* experiments, due to their improved cell-wall penetrating abilities.^55^

Many of the C3/C4-substituted 2OG derivatives synthesized inhibited human AspH by a mechanism involving competition with 2OG for binding the active site as revealed by inhibition assays performed at variable 2OG concentrations and crystallographic studies (Fig. 3 and 6). In general, the C4-substituted 2OG derivatives were more efficient AspH inhibitors than the C3-substituted 2OG derivatives (Table 1). The C4-substituted 2OG derivatives were also more potent AspH inhibitors than the corresponding C4-substituted NOG derivatives (ESI Fig. S4†). NOG and other *N*-oxalyl amino acids are plant natural products and it is proposed that they may act as enzyme inhibitors *in vivo*.^56^ Our observations thus raise the possibility that naturally occurring 2-oxoacids may be biologically relevant modulators of the activities of 2OG oxygenases and related enzymes.

Our results indicate that some 2OG derivatives efficiently inhibit AspH at physiologically relevant 2OG levels which range from 9–12 μM 2OG in human plasma^47^ to >1 mM 2OG in cells.^46^ For example, 2OG derivative 33 inhibits AspH with 0.6 mM 2OG in the assay (IC_50_ ∼ 5.6 μM; ESI Table S1†). 2OG competitive AspH inhibitors, such as 33, 34, or optimized variants of them, might be useful from a therapeutic perspective, because their inhibitory effect is unlikely to be compromised by mutations. Indeed an AspH mutation associated with Traboulsi syndrome occurs in the 2OG binding site (*i.e.* R735W) and is likely inactivating.^20*a*^

At high enzyme concentrations, AspH converts some of the inhibitors (*e.g.*34) slowly into the corresponding succinate derivatives (ESI Fig. S20†), indicating that the hydrophobic interactions of the 2OG C3/C4-substituents with AspH stabilize the AspH:2OG derivative complexes and/or reduce the rotational flexibility of the cosubstrate necessary to enable its oxidative decarboxylation. This observation is interesting because in some contexts, *e.g.* the inhibition of HIF-α prolyl hydroxylases, compounds that do not completely block activity even when present in excess, including poor cosubstrates, may actually be desirable, as they may help avoid overdose.^57^

C3/C4-substituted 2OG derivatives bear the potential to be used as small-molecule probes^55*a*^ in cells or *in vivo* to modulate the catalytic activity of 2OG oxygenases, provided selective interaction can be achieved. In this regard, the 2OG derivatives might, at least partially, be selective AspH inhibitors considering that C4-substituted 2OG derivatives were reported to not inhibit wild-type KDM4A,^25^ which is a 2OG dependent histone demethylase.^58^ Note that, NOG derivatives have been used *in vitro* and in cellular experiments to modulate the catalytic activities of FIH^24^ as well as of the KDM4 ^59^ and ten-eleven-translocation (TET) enzymes^60^ with some selectivity. Our results indicate that C4-substituted 2OG derivatives are more efficient in inhibiting AspH than the corresponding C4-substituted NOG derivatives (ESI Fig. S4†), as opposed to human FIH which also catalyzes the hydroxylation of Asp- and Asn-residues and for which NOG derivatives were more potent inhibitors.^24^ With regard to enzymes other than 2OG oxygenases, the AspH inhibitor 4,4-dimethyl-2OG (34), for example, does not efficiently substitute for 2OG in glutamic oxaloacetic aminotransferase catalyzed transamination reactions suggesting that it might not interfere with the catalytic activities of human aminotransferases.^61^

The lysine metabolite 2-oxoadipate, which is based on a C6 rather than a C5 carbon skeleton as in 2OG, is capable of acting as a relatively poor cosubstrate for procollagen prolyl hydroxylases,^62^ phytanoyl-CoA 2-hydroxylase (PAHX),^63^ and for a bacterial ethylene-forming 2OG dependent enzyme.^64^ However, to our knowledge, C3/C4-substituted 2OG derivatives have so far not been reported to substitute for 2OG as a cosubstrate for wild-type 2OG oxygenases. The observation that several of our synthetic 2OG derivatives efficiently substituted for 2OG as an AspH cosubstrate, with 2OG derivatives 16 and 41 being the most efficient (ESI Table S2†), is therefore of more general interest.

The observation that 4-carboxyphenylglyoxylic acid (41) can promote turnover of a 2OG oxygenase is remarkable because of its cyclic aromatic scaffold. 41 is thus a promising candidate to modulate AspH activity *in vivo*, as it might display selectivity for AspH over other 2OG oxygenases because of its distinctive structure. The oxidative decarboxylation of 41 by AspH is reminiscent of the reaction catalyzed by 4-hydroxyphenyl pyruvate dioxygenase (HPPD),^65^ which is from a different class of Fe(ii)-dependent oxygenases. A regioisomer of 41, 3-carboxyphenylglyoxylic acid (40), is a plant metabolite;^66^ thus, it is possible that 41 or related aromatic compounds may modulate the catalytic activity of 2OG oxygenases *in vivo*.

The *k*_cat_/*K*_m_-value of AspH for 3-methyl-2OG (16) is approximately three times higher than for 2OG, suggesting the feasibility of 2OG derivatives to selectively enhance AspH (or indeed other 2OG oxygenase) catalysis in cells or on the cell surface of cancer cells in the presence of 2OG (Table 2). 16 is of particular interest because it is a reported ingredient of human nutrition (*i.e.* it is present in honey);^67^ thus, 16 might modulate the activity of AspH and potentially other 2OG oxygenases in humans. 2-Methylsuccinate, which is formed by the AspH-catalyzed oxidative decarboxylation from 16 (ESI Fig. S5†), has been detected in human urine^68^ and is *inter alia* used as a biomarker for metabolic diseases as it is a product of other metabolic pathways (*i.e.* isoleucine catabolism);^69^ this, however, does not rule out the possibility that some 2-methylsuccinate might originate from the oxidative decarboxylation of 16 catalyzed by 2OG oxygenases. 16 is also a proposed precursor of 3-methyl glutamate, which is incorporated in natural products such as polytheonamide A and B^70^ and daptomycin.^71^16 is likely biosynthesized in microorganisms through the direct reaction of 2OG and SAM,^72^ suggesting that it might also be biosynthesized by animals in a similar manner. The results thus raise the possibility that 16 or other C3/C4-substituted 2OG derivatives, including their corresponding glutamate derivatives, are human/animal metabolites and/or are bioavailable through nutrition or the gut microbiome.

In comparison with 16, its isomer 4-methyl-2OG (26), which is a reported metabolite in plants^73^ and ingredient of wine,^74^ is substantially less efficient in substituting for 2OG as a cosubstrate (ESI Table S2†), highlighting that the position of the 2OG substituent determines the ability of the 2OG derivatives to serve as an alternative AspH cosubstrate.

AspH was co-crystallized in the presence of 3-methyl-2OG (16) affording structures with the highest resolution reported for AspH so far (1.5 and 1.8 Å; ESI Fig. S11 and S12†). The crystallographic studies revealed that 16 substitutes for, and binds similarly to, 2OG in the AspH active site (Fig. 5). However, the C3- and C4-methylenes of 16 occupy different conformations orienting the 2OG C3-methyl-substituent towards Val676 and Val727, so enabling hydrophobic interactions. Superimposition of 16 and 3-ethyl-2OG (17), which is a potent AspH inhibitor, reveals that both 2OG derivatives adopt a similar conformation when bound to AspH (Fig. 6c). Despite the use of racemic 16 and 17 for crystallizations, in both cases the (3*R*)-enantiomers were observed by crystallography, with the C3-alkyl substituents interacting with one face of a hydrophobic pocket (ESI Fig. S16c†). Thus, subtle changes in the structure of the 2OG derivative can have a pronounced effect on AspH catalysis, resulting in either efficient substrate hydroxylation or efficient AspH inhibition. The exact factors that determine whether a particular 2OG derivative inhibits or enables AspH catalysis are unknown, but might relate to differences in oxygen binding, modulation of steps after oxygen binding or to the stability of the AspH:succinate derivative complexes (ESI Fig. S21†).

The potential of compensating catalytically inactivating (with 2OG as a cosubstrate) mutants of 2OG oxygenases with 2-oxoacids has been exemplified in the case of PAHX.^63^ Our results thus raise to possibility that naturally occurring 2OG derivatives/analogues may modulate the activity of 2OG oxygenases and related enzymes (*e.g.* HPPD^65^), either by acting as inhibitors or by replacing 2OG in catalysis. Indeed, it is possible that some of the 2OG oxygenases without assigned biochemical functions (*e.g.* Jumonji C domain-containing protein 1c, JMJD1C)^75^ or with unusual Fe(ii) binding site geometries (*e.g.* PHD finger protein 2 (PHF2)^76^ and hairless^77^) use 2-oxoacid cosubstrates other than 2OG. Future work will focus on exploring these possibilities using the 2OG derivatives described here and others prepared by the cyanosulfur ylid methodology.

## Conclusions

A user-friendly efficient synthesis of C3/C4-substituted 2OG derivatives based on the use of cyanosulfur ylids was developed and employed to afford a diverse set of 2OG derivatives for detailed biochemical and structural investigations on the cosubstrate selectivity of the human 2OG oxygenase AspH. The overall results reveal that C3/C4-substituted 2OG derivatives can have profound effects on AspH catalysis and, by implication, likely other 2OG oxygenases. Simple alkyl substituents, *e.g.* methyl at the 2OG C3- or C4-postion, enables retention of productive catalysis, likely in a stereoselective manner. By contrast, *e.g.* 2OG C4-dimethylation leads to inhibition/low levels of cosubstrate activity. The use of 2OG derivatives may thus inform on both the catalytic mechanisms and biological roles of AspH and other 2OG oxygenases, and aid in the development of new types of small-molecules that modulate 2OG oxygenase activity.

## Data availability

Crystal structure data for His_6_-AspH_315–758_ complexed to Mn, 2OG or a 2OG derivative (3-methyl-2OG, 16; 3-ethyl-2OG, 17; 4,4-dimethyl-2OG, 34; *N*-oxalyl-α-methylalanine, 49), and, in some cases, substrate peptide (hFX-EGFD1_86–124_-4Ser) are deposited in the protein data bank with PDB accession codes: 6YYU (AspH:2OG), 6YYW (AspH:2OG:hFX-EGFD1_86–124_-4Ser), 6YYV (AspH:16), 6YYX (AspH:16:hFX-EGFD1_86–124_-4Ser), 6YYY (AspH:34:hFX-EGFD1_86–124_-4Ser), 6Z6Q (AspH:17:hFX-EGFD1_86–124_-4Ser), and 6Z6R (AspH:49:hFX-EGFD1_86–124_-4Ser).

## Author contributions

L. B. synthesized the 2OG derivatives and the AspH substrate, produced recombinant human AspH, and performed AspH assays and crystallizations. Y. N. solved and refined the AspH crystal structures. All authors analyzed data. L. B. and C. J. S. wrote the manuscript with help from Y. N.

## Conflicts of interest

There are no conflicts to declare.

## Supplementary Material

SC-012-D0SC04301J-s001
