# Supplementary material for: Synthesis of 2-oxoglutarate derivatives and their evaluation as cosubstrates and inhibitors of human aspartate/asparagine-β-hydroxylase
Source: Chem Sci. 2020 Dec 7;12(4):1327–42. doi: 10.1039/d0sc04301j (PMC8179049; doi:10.1039/d0sc04301j)
Supplement: SC-012-D0SC04301J-s001 [file SC-012-D0SC04301J-s001.pdf]

Electronic Supplementary Material (ESI) for Chemical Science.
This journal is © The Royal Society of Chemistry 2020

Supporting Information

Synthesis of 2-oxoglutarate derivatives and their evaluation as cosubstrates and inhibitors of human aspartate/asparagine-β-hydroxylase

Lennart Brewitz, Yu Nakashima, and Christopher J. Schofield*

Chemistry Research Laboratory, University of Oxford, Oxford, United Kingdom

*christopher.schofield@chem.ox.ac.uk

Table of contents

1. Supporting figures 1-30
2. Supporting tables 31-35
3. General information 36
4. General synthetic procedures 36-38
5. Synthetic procedures and analytical data 39-68
6. Recombinant AspH production and purification 69
7. AspH substrate synthesis 69
8. AspH inhibition assays 69-70
9. Determination of kinetic parameters 70
10. Crystallography and structure solutions 70-71
11. References 71-72
12. ^{1}H and ^{13}C NMR spectra of novel compounds prepared for this study 73-178

1. Supporting figures

Supporting Figure S1. The canonical and the non-canonical EGFD disulfide connectivity patterns and structures of synthetic AspH substrates used in this work. (a) The canonical (Cys 1–3, 2–4, 5–6; green) EGFD disulfide pattern bearing the consensus sequence (orange/red); the Asp/Asn-residue for AspH-catalyzed hydroxylation is in red; (b) The non-canonical (Cys 1–2, 3–4, 5–6; green) EGFD disulfide pattern bearing the consensus sequence (orange/red) for AspH-catalyzed Asp/Asn-residue (red) hydroxylation, as identified as a substrate requirement for isolated AspH;[1] (c) The hFX-EGFD1$_{86\text{-}124}$-4Ser peptide[1] which is based on the sequence of EGFD1 of the confirmed AspH substrate human coagulation Factor X (hFX)[2] and which was used as AspH substrate for the crystallographic analyses in this work. The peptide was synthesized by solid phase peptide synthesis (SPPS) and purified by GL Biochem (Shanghai) Ltd. The AspH hydroxylation site (Asp103$_{hFX}$) is in red; consensus sequence residues are in orange; cystine sulfurs forming the central Cys 3–4 macrocycle are in green; substituted residues are in light blue; numbering is according to the sequence of hFX;[2] (d) Structure of the cyclic thioether peptide hFX-CP$_{101\text{-}119}$ which was used in this work as an AspH substrate for solid phase extraction (SPE) coupled to mass spectrometry (MS) AspH turnover assays, this peptide mimics the non-canonical EGFD disulfide pattern of hFX.[3] The peptide was synthesized and purified as described in Section 7 of the Supporting Information.[1,4] The AspH hydroxylation site (Asp103$_{hFX}$) is in red, consensus sequence residues are in orange, the cysteine sulfur is in green, substituted residues are in light blue; numbering is according to the sequence of hFX.

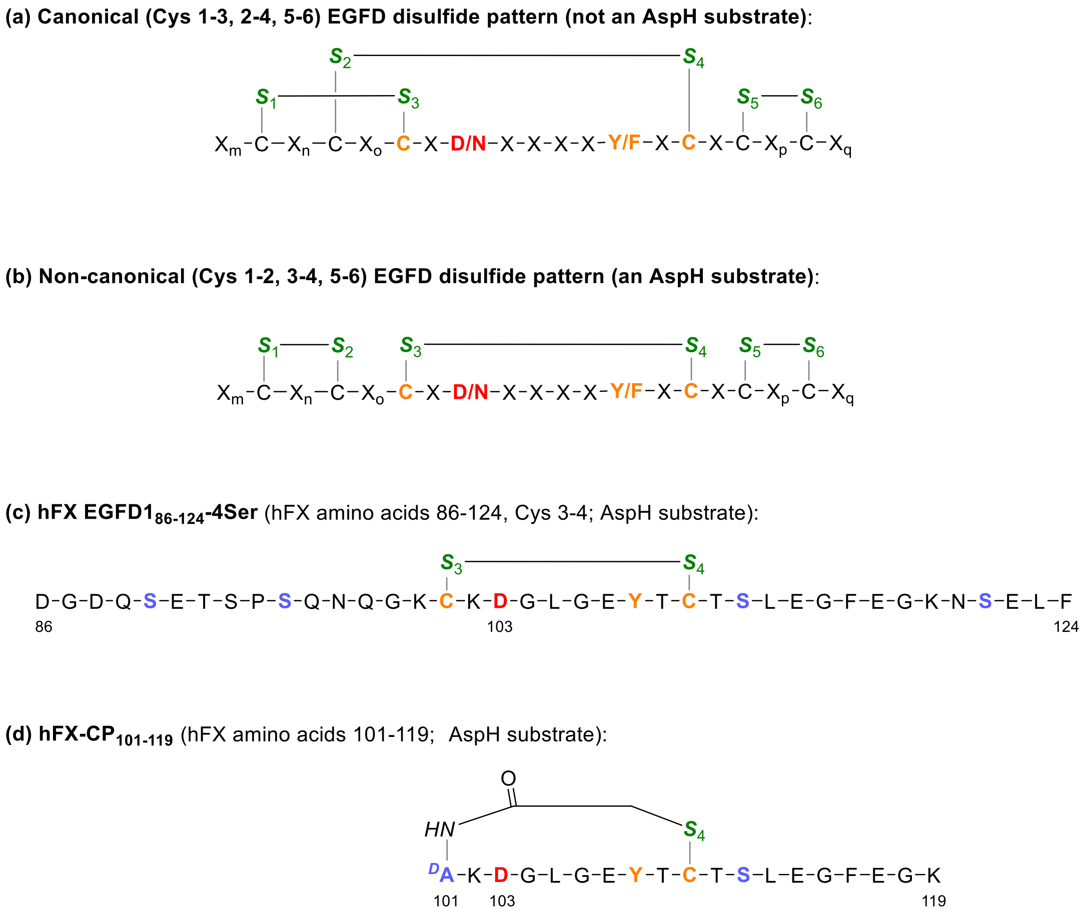

Supporting Figure S2. Syntheses of mono-methyl dicarboxylic acid half-esters (continues on the following page). Mono-methyl dicarboxylic acid half-esters, which are used as synthetic precursors for the synthesis of 2OG derivatives, were synthesized employing the following reactions: (a) nucleophilic openings of symmetric cyclic carboxylic acid anhydrides by methoxide, (b) carboxylation reactions of aryl iodides, (c) Heck reactions[5] of orthogonally protected itaconic acid diesters, and (d) Horner-Wadsworth-Emmons reactions[6] of alkyl phosphonates with alkyl and aryl aldehydes.

(a) Nucleophilic openings of symmetric cyclic carboxylic acid anhydrides by methoxide

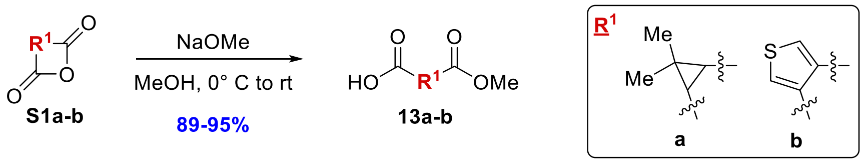

Mono-methyl dicarboxylic acid half-esters **13a** and **13b** were obtained in 89-95% yield by the nucleophilic opening of symmetric cyclic carboxylic acid anhydrides **S1a** and **S1b** using sodium methoxide.

(b) Carboxylation reactions of aryl iodides

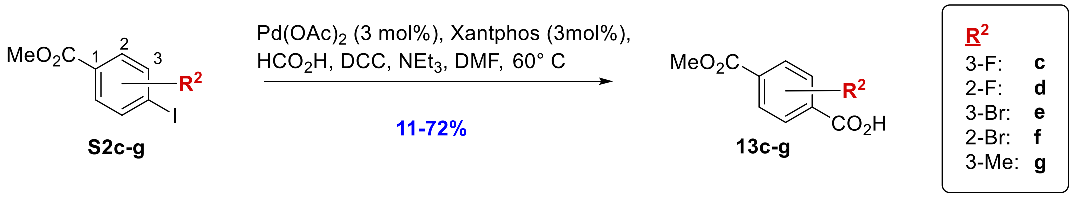

Mono-methyl dicarboxylic acid half-esters **13c-13g** were obtained in in 11-72% yield via Pd-catalyzed carboxylation using aryliodides **S2c-g** as substrates. The reaction relied on the use of formic acid and *N*,*N*-dicyclohexylcarbodiimide (DCC) to generate carbon monoxide according to a modified literature protocol.[7]

(c) Heck reactions of aryliodides with an orthogonally protected itaconate derivative

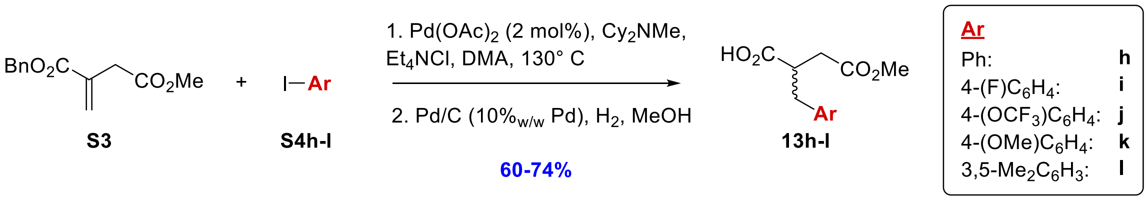

Heck reactions,[5] using the commercial (hetero-)aryliodides **S4h-l** and the orthogonally protected itaconate **S3**[8] as substrates, afforded intermediates for the synthesis of 2OG derivatives bearing benzyl groups at the C3-position. The Heck reaction products were obtained as a single alkene diastereomer, which was tentatively assigned as the (*E*)-isomer based on literature reports (the stereochemistry is not relevant for non-enantioselective reductions).[9] The Heck reactions were scalable (15 mmol scale) by increasing the reaction concentration from 0.25 M[9] to 1.5 M. Simultaneous hydrogenation of the trisubstituted olefin and cleavage of the benzyl ester afforded the corresponding desired mono-methyl dicarboxylic acid half-esters **13h-l** as racemic mixtures, with small amounts of impurities which were separated after the following reaction.

Chiral metal complexes have been reported for the enantioselective reduction of itaconate derivatives which could potentially be applied to obtain enantiopure C3-substituted mono-methyl dicarboxylic acid half-esters.[10]

(d) Horner-Wadsworth-Emmons reactions of phosphonates with alkyl and aryl aldehydes

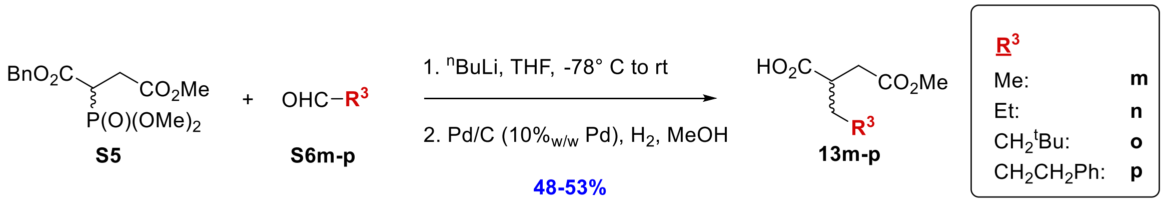

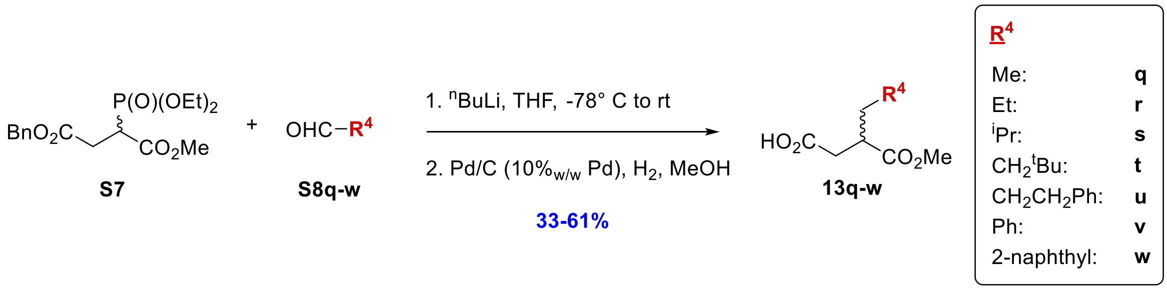

A diverse set of mono-methyl dicarboxylic acid half-esters (**13m-13w**) was synthesized via Horner-Wadsworth-Emmons reaction[6] of phosphonates **S5** and **S7**[11] with commercial aldehydes. Phosphonates **S5** and **S7**[11] were efficiently obtained from commercial materials by alkylation reactions. The desired mono-methyl dicarboxylic acid half-esters **13m-13w** were obtained as racemic mixtures after simultaneous hydrogenation of the trisubstituted olefin and cleavage of the benzyl ester. Chiral metal complexes have been reported for the enantioselective reduction of itaconate derivatives which could potentially be applied to obtain enantiopure C3[10] and C4[12] substituted mono-methyl dicarboxylic acid half-esters.

By contrast with the efficient use of phosphonates for the synthesis of mono-methyl dicarboxylic acid half-esters, an alternative entry to C4-substituted mono-methyl dicarboxylic acid half-esters using the Stobbe condensation[13] was less efficient due to the formation of reaction byproducts resulting in low product yields and time-consuming product purifications, even when applying the reported recrystallization conditions.

Supporting Figure S3. Robustness of the AspH inhibition assays. (a) Z'-factors[14] (circles) and (b) signal-to-noise ratios (S/N, squares) for the AspH inhibition assay plates used to determine IC_{50}-values, 16 compounds were assayed per plate, using DMSO and 2,4-PDCA as negative and positive inhibition controls, respectively; technical duplicates were in adjacent wells. The Z'-factors >0.5 (grey line) indicate a stable and robust assay.[14] Z'-factors and S/N-ratios were determined according to the cited literature using Microsoft Excel software as previously described.[3]

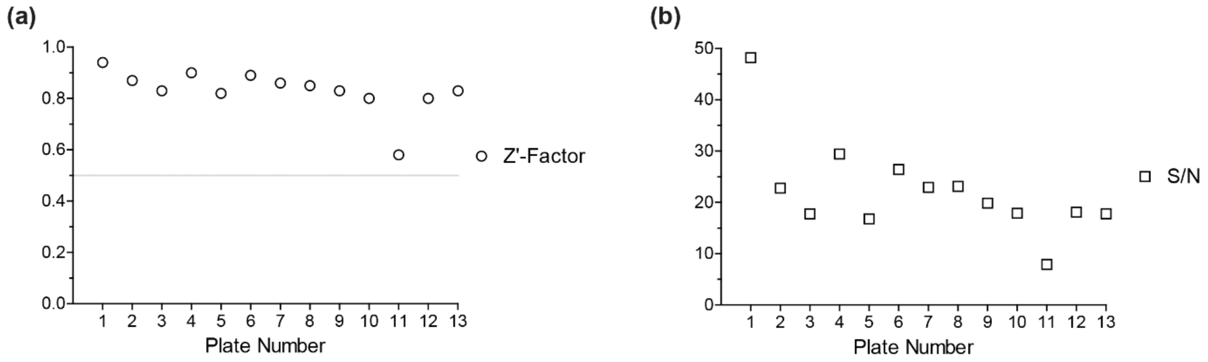

Supporting Figure S4. AspH inhibition by 4,4-dimethyl-2OG (34) compared to the analogous *N*-oxalylglycine derivative. The inhibition of AspH by 4,4-dimethyl-2OG (**34**; Entry 1) compares favourably with AspH inhibition by *N*-oxalylglycine (NOG, **3**; Entry 2) and by the corresponding *N*-oxalylglycine derivative **49** (*N*-oxalyl-α-methylalanine; Entry 3): The IC_{50}-value for **34** is approximately half that of NOG (**3**) and one tenth that of *N*-oxalyl-α-methylalanine (**49**). The reduced inhibitory properties of NOG (**3**) and *N*-oxalyl-α-methylalanine (**49**) might result from an enhanced rotational barrier imposed by the nitrogen lone-pair restricting the conformational space the *N*-oxalyl inhibitors can occupy when binding to AspH.

The graph shows dose-response curves used to determine IC_{50}-values for 4,4-dimethyl-2OG (**34**, red triangles), NOG (**3**, green circles), and *N*-oxalyl-α-methylalanine (**49**, orange squares). For each compound, one representative set of three independently determined dose-response curves each composed of technical duplicates is shown. Note that under prolonged incubation conditions, **34** can support slow hydroxylation (Supporting Figure S20).

Entry	2OG derivative	a)IC_{50} [µM]
1	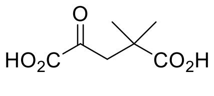 **34**	0.31 ± 0.10
2	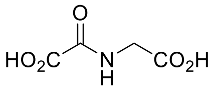 **NOG (3)**	0.99 ± 0.31
3	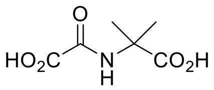 **49**	2.9 ± 1.3

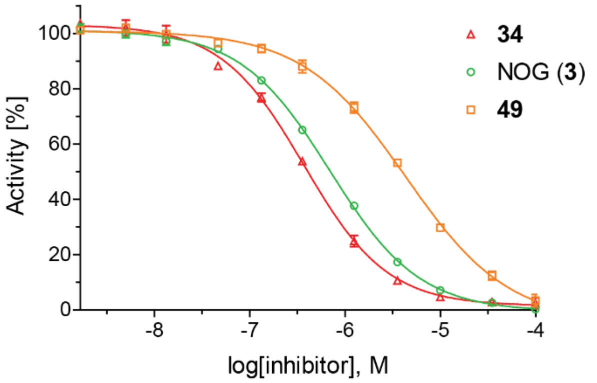

a) Mean of three independent runs (n = 3; mean ± standard deviation, SD). AspH inhibition assays were performed as described in the Supporting Information (Section 8) using 50 nM His_6-$AspH_{315-758}$ and 1.0 µM hFX-$CP_{101-119}$ (Supporting Figure S1d). The AspH inhibition assays were of good quality which high S/N ratios and Z'-factors[14] (>0.5 for each plate) indicate (Supporting Figure S3)

Supporting Figure S5. AspH catalyzes the oxidative decarboxylation of 2OG derivatives 16 and 41 to give 2-methylsuccinate and terephthalate, respectively (continues on the following two pages). The AspH-catalyzed oxidative decarboxylation of 2OG or 2OG derivatives **16** and **41** was monitored using a Bruker AVIII 700 NMR machine equipped with a 5mm ^{1}H(^{13}C/^{15}N) inverse cryoprobe. Assay conditions: 10 µM His$_6$-AspH$_{315-758}$, 150 µM hFX-CP$_{101-119}$ (Supporting Figure S1d), 150 µM 2OG or 2OG derivative, and 50 µM ammonium iron(II) sulfate hexahydrate (FAS, $(NH_4)_2Fe(SO_4)_2{\cdot}6H_2O$) in 50 mM aqueous Tris-$d_{11}$ containing 10%$_{v/v}$ D_2O (pH 7.5, 25° C). The ratio of the hFX-CP$_{101-119}$ peptide and the hydroxylated hFX-CP$_{101-119}$ peptide, which is the product of the AspH-catalyzed reaction, was determined using SPE-MS (Supporting Information, Section 9) after NMR analysis indicated full conversion of the AspH cosubstrate.

(I) Controls using 2OG as an AspH cosubstrate: (a) Close-up (3.0-2.2 ppm) of the ^{1}H NMR spectrum of commercial 2OG in 50 mM aqueous Tris-d_{11} containing 10%$_{v/v}$ D_2O (pH 7.5, 25° C); (b) Close-up (3.0-2.2 ppm) of the ^{1}H NMR spectrum of commercial succinate in 50 mM aqueous Tris-d_{11} containing 10%$_{v/v}$ D_2O (pH 7.5, 25° C); (c) a timecourse NMR experiment (t = 0.5 min, top blue spectrum; t = 5 min, bottom black spectrum) reveals that AspH converts 2OG into succinate within 5 min under the assay conditions, in agreement with reported studies that have identified succinate (and CO_2) as the sole 2OG-derived reaction product during AspH catalysis;[1] (d) Analysis of the final reaction mixture (t = 5 min) by SPE-MS reveals complete hydroxylation (>95% conversion) of the hFX-CP$_{101-119}$ peptide (m/z = 1022.5 corresponds to the +2 charge state of hFX-CP$_{101-119}$; m/z = 1030.5 corresponds to the +2 charge state of hydroxylated hFX-CP$_{101-119}$), indicating that approximately equimolar amounts of succinate and hydroxylated hFX-CP$_{101-119}$ peptide are present in the final reaction mixture. Thus, the AspH-catalyzed oxidative decarboxylation of 2OG to give succinate is highly coupled with AspH-catalyzed substrate hydroxylation.

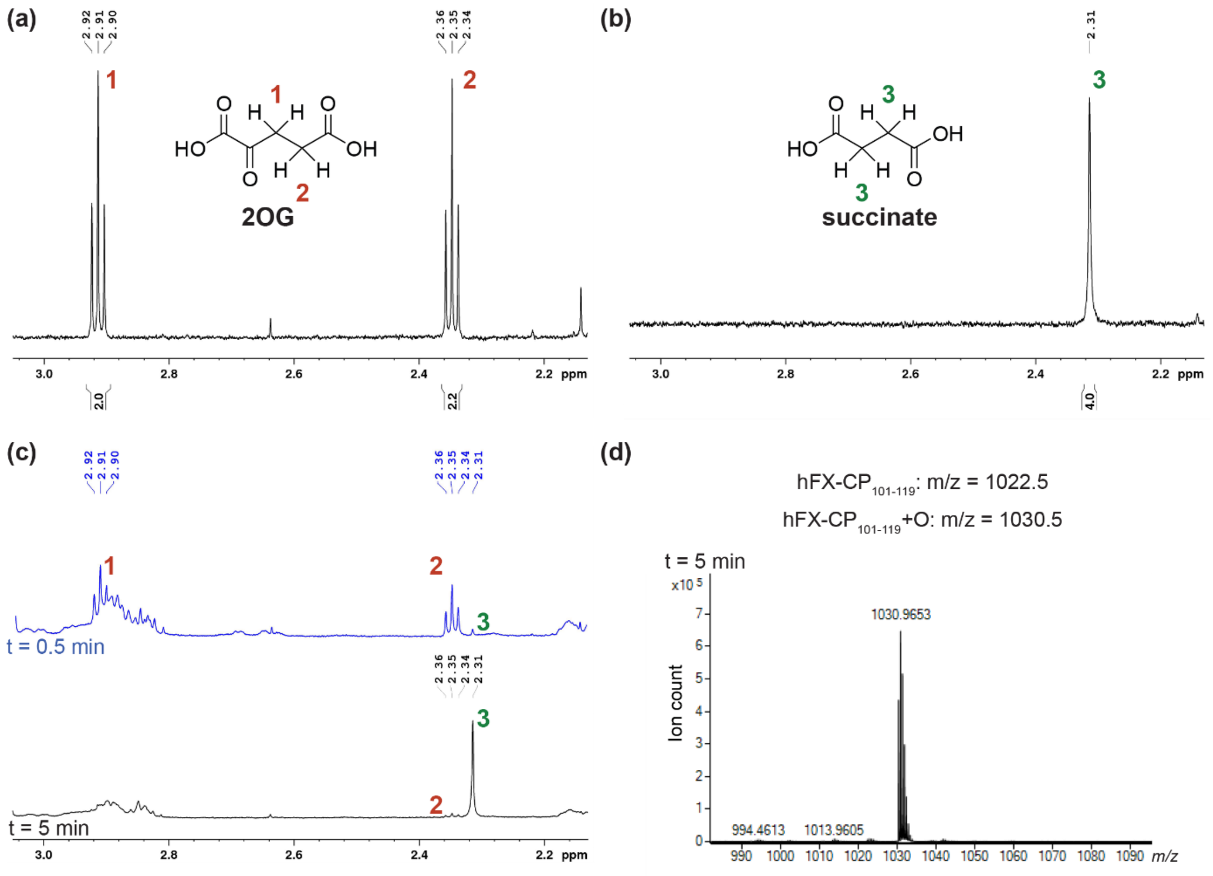

(II) Experiments using 3-methyl-2OG (**16**) as an AspH cosubstrate: (a) Close-up (3.4-0.9 ppm) of the ^{1}H NMR spectrum of synthetic **16** in 50 mM aqueous Tris-d_{11} containing 10%$_{v/v}$ D_2O (pH 7.5, 25° C); (b) Close-up (3.4-0.9 ppm) of the ^{1}H NMR spectrum of commercial 2-methylsuccinate (**50**) in 50 mM aqueous Tris-d_{11} containing 10%$_{v/v}$ D_2O (pH 7.5, 25° C); (c) a timecourse NMR experiment (t = 0.5 min, top blue spectrum; t = 5 min, bottom black spectrum) reveals that AspH converts **16** into 2-methylsuccinate within 5 min under the assay conditions; (d) Analysis of the final reaction mixture (t = 5 min) by SPE-MS reveals complete hydroxylation (>95% conversion) of the hFX-CP$_{101-119}$ peptide (m/z = 1022.5 corresponds to the +2 charge state of hFX-CP$_{101-119}$; m/z = 1030.5 corresponds to the +2 charge state of hydroxylated hFX-CP$_{101-119}$), indicating that approximately equimolar amounts of 2-methylsuccinate (**50**) and hydroxylated hFX-CP$_{101-119}$ peptide are present in the final reaction mixture. Thus, the AspH-catalyzed oxidative decarboxylation of **16** to give 2-methylsuccinate (**50**) is highly coupled with AspH-catalyzed substrate hydroxylation.

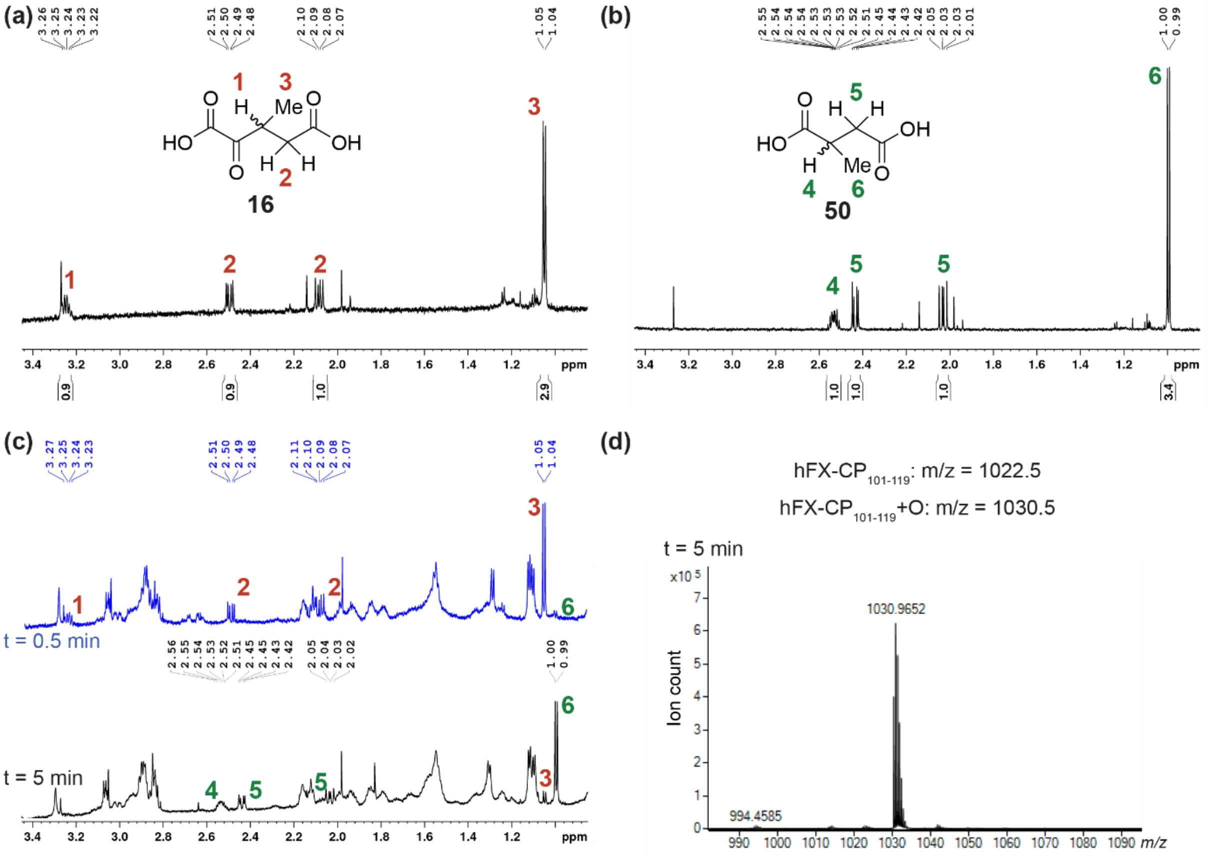

(III) Experiments using the 2OG derivative **41** as an AspH cosubstrate: (a) Close-up (8.6-7.4 ppm) of the ^{1}H NMR spectrum of synthetic **41** in 50 mM aqueous Tris-d_{11} containing 10%$_{v/v}$ D_2O (pH 7.5, 25° C); (b) Close-up (8.6-7.4 ppm) of the ^{1}H NMR spectrum of commercial terephthalate (**51**) in 50 mM aqueous Tris-d_{11} containing 10%$_{v/v}$ D_2O (pH 7.5, 25° C); (c) a timecourse NMR experiment (t = 0.5 min, top blue spectrum; t = 5 min, bottom black spectrum) reveals that AspH converts the 2OG derivative **41** into terephthalate within 5 min under the assay conditions; (d) Analysis of the final reaction mixture (t = 5 min) by SPE-MS reveals complete hydroxylation (>95% conversion) of the hFX-CP$_{101-119}$ peptide (m/z = 1022.5 corresponds to the +2 charge state of hFX-CP$_{101-119}$; m/z = 1030.5 corresponds to the +2 charge state of hydroxylated hFX-CP$_{101-119}$), indicating that approximately equimolar amounts of terephthalate (**51**) and hydroxylated hFX-CP$_{101-119}$ peptide are present in the final reaction mixture. Thus, the AspH-catalyzed oxidative decarboxylation of the 2OG derivative **41** to give terephthalate (**51**) is highly coupled with AspH-catalyzed substrate hydroxylation.

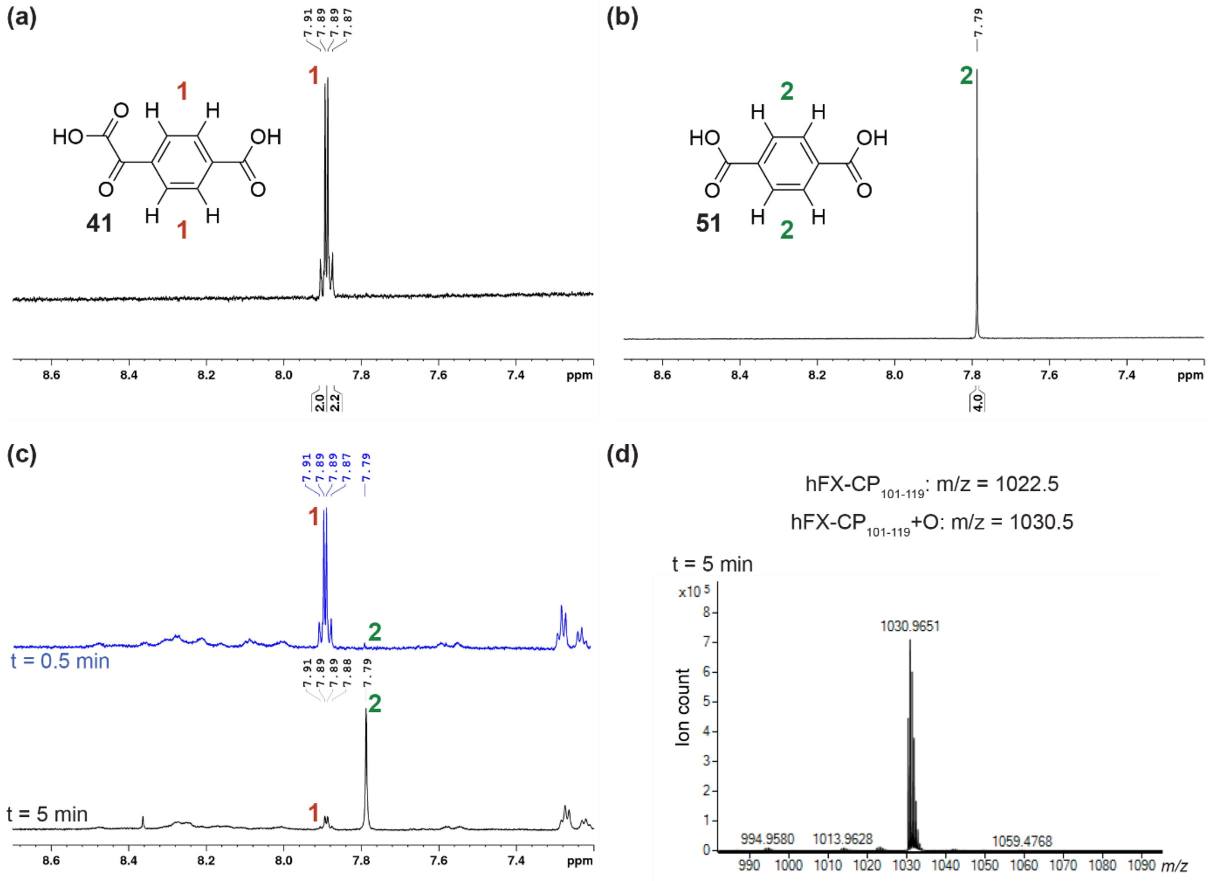

Supporting Figure S6. Initial hydroxylation rates of AspH-catalyzed peptide hydroxylations used to determine kinetic parameters for 2OG derivatives (continues on the following page). Maximum velocities (v_{max}^{app}) and Michaelis constants (K_m^{app}) of AspH were determined in independent triplicates for 2OG and the 2OG derivatives **16** and **41**, monitoring AspH-catalyzed hydroxylation of hFX-CP$_{101-119}$ (Supporting Figure S1d) by solid-phase extraction coupled to mass spectrometry (SPE-MS) as described in Section 9 of the Supporting Information. Conditions: 0.1 µM His$_6$-AspH$_{315-758}$, 2.0 µM hFX-CP$_{101-119}$, 100 µM L-ascorbic acid (LAA), and 20 µM ammonium iron(II) sulfate hexahydrate (FAS, $(NH_4)_2Fe(SO_4)_2{\cdot}6H_2O$) in 50 mM HEPES (pH 7.5, 20° C). Measurement times were normalized to the first sample injection analyzed after the addition of the Enzyme Mixture to the Substrate Mixture (t = 0 s), by which time low levels of hydroxylation were manifest. Data are shown as the mean average of three independent runs (n = 3; mean ± standard deviation, SD).

(a) Time course of the AspH-catalyzed hydroxylation reaction of hFX-CP$_{101-119}$ (Supporting Figure S1d) for the shown 2OG concentrations; and (b) hydroxylation rates used to determine kinetic parameters of AspH for 2OG.

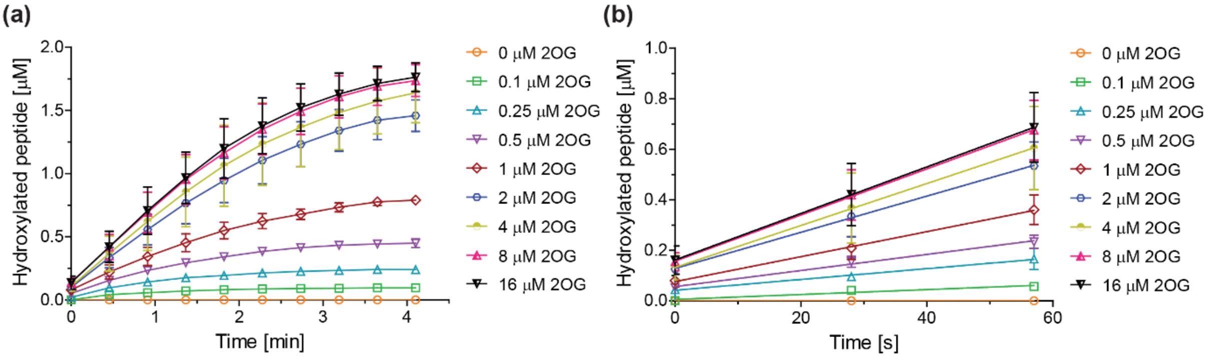

(c) Time course of the AspH-catalyzed hydroxylation reaction of hFX-CP$_{101-119}$ (Supporting Figure S1d) for the shown concentrations of 3-methyl-2OG (**16**); and (d) hydroxylation rates used to determine kinetic parameters of AspH for **16**.

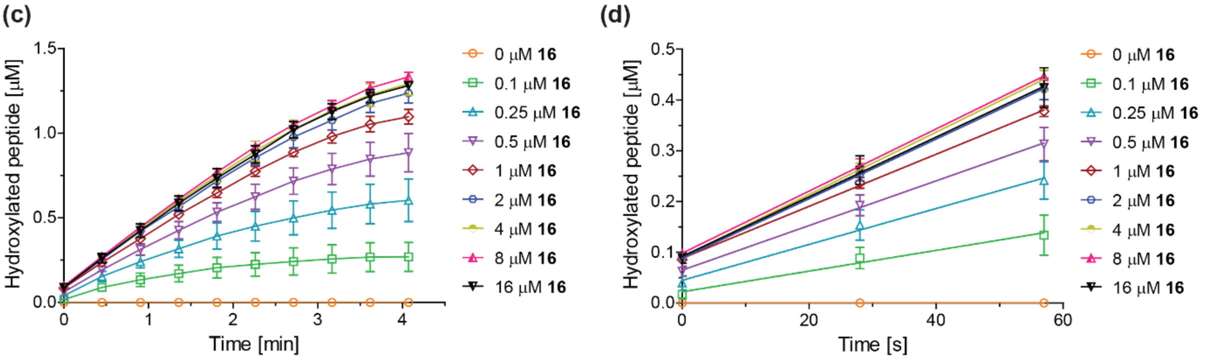

(e) Time course of the AspH-catalyzed hydroxylation reaction of hFX-CP$_{101-119}$ (Supporting Figure S1d) for the shown concentrations of 4-carboxyphenylglyoxylic acid (**41**); and (f) hydroxylation rates used to determine kinetic parameters of AspH for **41**.

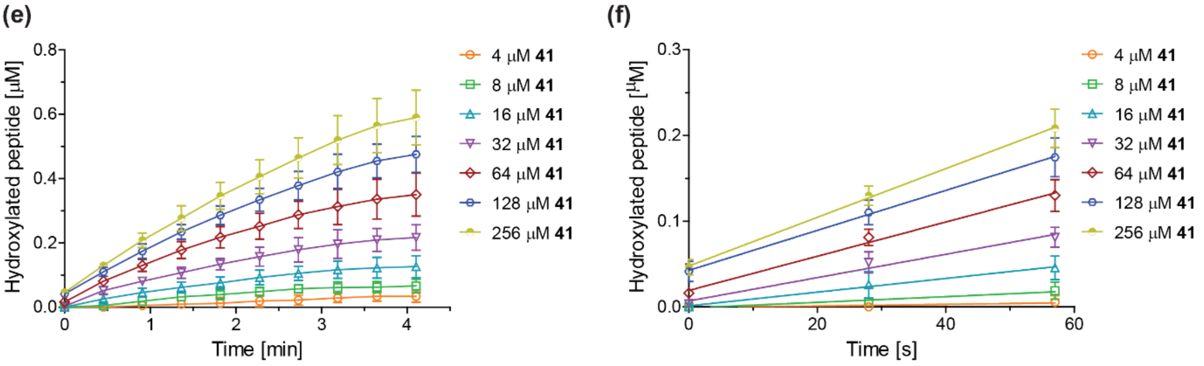

Supporting Figure S7. Views from a crystal structure of AspH complexed with 2OG and Mn (AspH:2OG; PDB ID: 6YYU). Colors: grey: His_6-$AspH_{315-758}$; green: carbon-backbone of 2OG; violet: Mn; red: oxygen; blue: nitrogen. w: water. (a) Overview of the AspH:2OG crystal structure; (b) superimposition of a view from the AspH:2OG structure with one from the reported AspH:NOG structure (AspH: pale pink, carbon-backbone of *N*-oxalylglycine (NOG, **3**): magenta; PDB ID: 5JZA)[1] reveals similar AspH conformations (Cα RMSD = 0.21 Å); (c) superimposition of a view from the AspH:2OG structure with one from the reported AspH:L-malate structure (AspH: pale blue, carbon-backbone of L-malate: cyan; PDB ID: 5JZ6)[1] reveals similar AspH conformations (Cα RMSD = 0.21 Å); (d) superimposition of views from AspH active site residues of the AspH:2OG, AspH:NOG, and AspH:L-malate structures engaged in ligand and metal binding. The comparison reveals that the crystallographically observed conformations of the AspH active site residues do not change when substituting 2OG for NOG or L-malate. 2OG and NOG bind AspH and the active site metal in a similar manner and adopt a similar conformations. The binding of L-malate to the AspH active site metal is distinct from that of 2OG and NOG, because its C1-carboxylate engages in bidentate metal coordination.

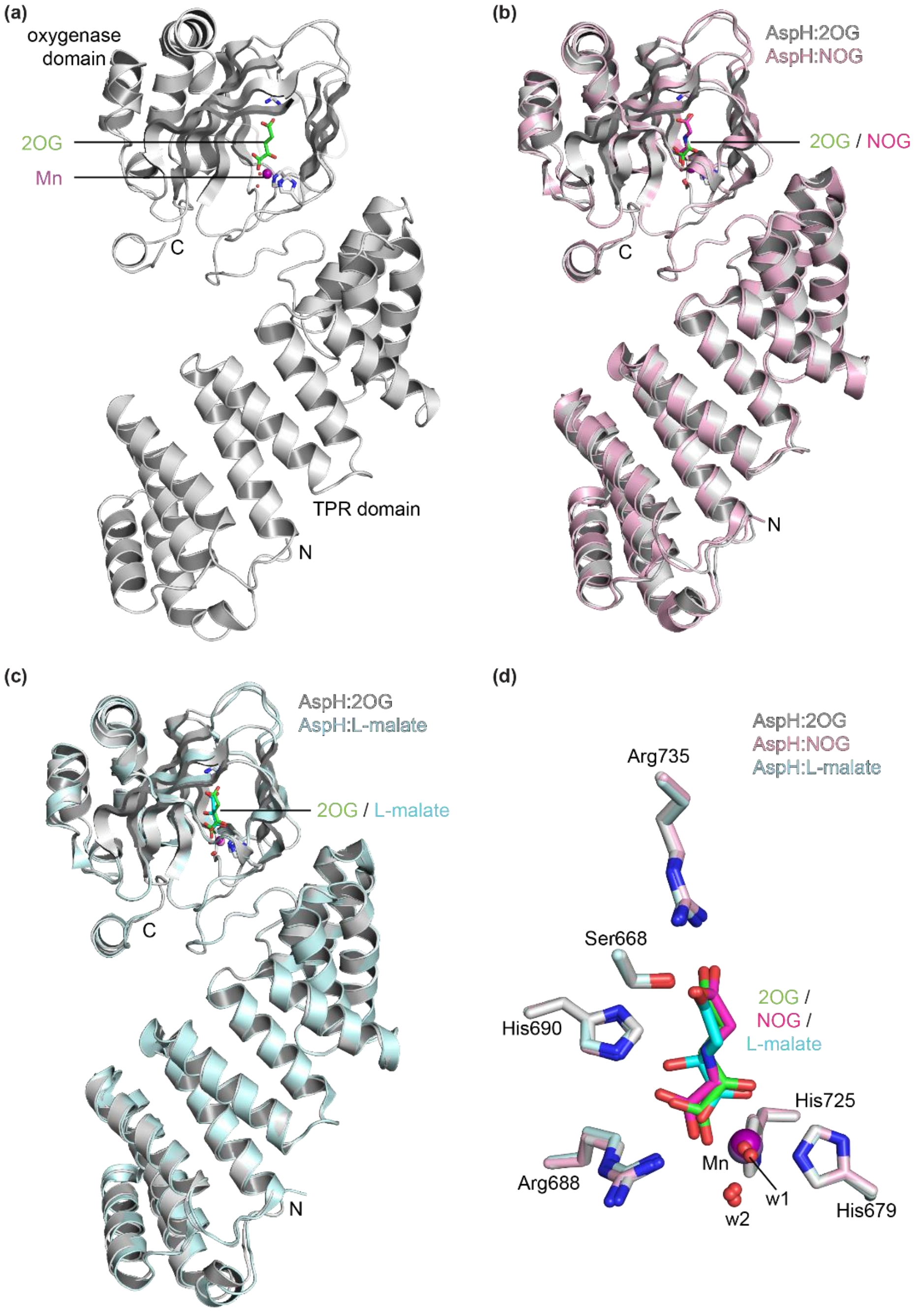

Supporting Figure S8. Views from a crystal structure of AspH complexed with 2OG, Mn, and a synthetic EGFD substrate peptide (AspH:2OG:hFX-EGFD1$_{86-124}$-4Ser; PDB ID: 6YYW). Colors: grey: His$_6$-AspH$_{315-758}$; green: carbon-backbone of 2OG; violet: Mn; orange: carbon-backbone of the hFX-EGFD1$_{86-124}$-4Ser peptide (Supporting Figure S1c); red: oxygen; blue: nitrogen.

(a) Overview of the AspH:2OG:hFX-EGFD1$_{86-124}$-4Ser crystal structure; (b) OMIT electron density map (mF_o-DF_c) contoured to 3σ around the hFX-EGFD1$_{86-124}$-4Ser peptide from the AspH:2OG:hFX-EGFD1$_{86-124}$-4Ser structure reveals electron density for residues Gly99$_{hFX}$ to Thr111$_{hFX}$ including for the disulfide bridged (Cys101$_{hFX}$ and Cys110$_{hFX}$) ten-membered non-canonical EGFD macrocycle. Clear electron density is observed for the side chain of Tyr108$_{hFX}$, an hFX-EGFD1 residue which is part of the AspH-substrate consensus sequence requirement (Supporting Figure S1); the Tyr108$_{hFX}$ side chain interacts with the TPR domain of AspH as revealed in reported AspH:substrate complex structures.[1]

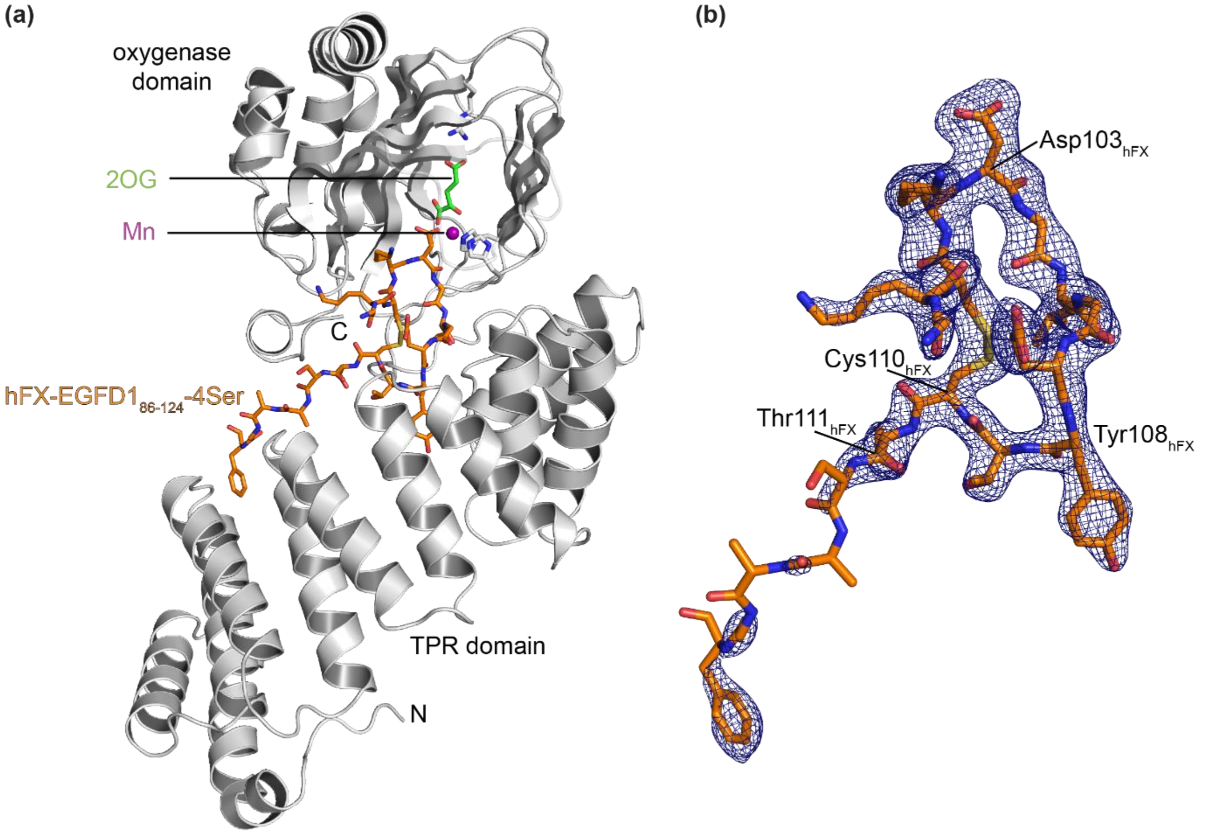

Supporting Figure S9. AspH binds EGFD substrates through an induced fit mechanism in the presence of its natural cosubstrate 2OG. Colors: grey: His_6-$AspH_{315-758}$; green: carbon-backbone of 2OG; violet: Mn; orange: carbon-backbone of the hFX-$EGFD1_{86-124}$-4Ser peptide (Supporting Figure S1c); red: oxygen; blue: nitrogen.

(a) Superimposition of a view from the AspH:2OG:hFX-$EGFD1_{86-124}$-4Ser structure (AspH: grey, carbon-backbone of 2OG: green, carbon-backbone of the hFX-$EGFD1_{86-124}$-4Ser peptide: orange; Supporting Figure S8) with one from the AspH:2OG structure (AspH: pale green, carbon-backbone of 2OG: lemon; Supporting Figure S7) reveals that the distance between the Cα atoms of Leu433 on TPR repeat α6 and Pro756 in the AspH C-terminal region decreases from ~20 Å to ~14 Å upon substrate binding (Cα RMSD = 2.59 Å). Evidence for an induced fit mechanism involving this conformational change has previously been described when AspH was crystallized in the presence of *N*-oxalylglycine (NOG, **3**) rather than 2OG;[1] (b) superimposition of a view from the AspH:2OG:hFX-$EGFD1_{86-124}$-4Ser structure (AspH: grey, carbon-backbone of 2OG: green, carbon-backbone of the hFX-$EGFD1_{86-124}$-4Ser peptide: orange; Supporting Figure S8) with one from the reported AspH:NOG:hFX-$EGFD1_{86-124}$-4Ser structure (AspH: pale pink, carbon-backbone of NOG: magenta, carbon-backbone of the hFX-$EGFD1_{86-124}$-4Ser peptide: aquamarine; PDB ID: 5JQY)[1] reveals similar AspH (Cα RMSD = 0.23 Å) and substrate (Cα RMSD = 0.11 Å) conformations. Note that in the AspH:2OG:hFX-$EGFD1_{86-124}$-4Ser structure, the $Asp103_{hFX}$ side-chain carboxylate was refined in only one conformation compared to the two conformations as observed in the AspH:NOG:hFX-$EGFD1_{86-124}$-4Ser structure.[1]

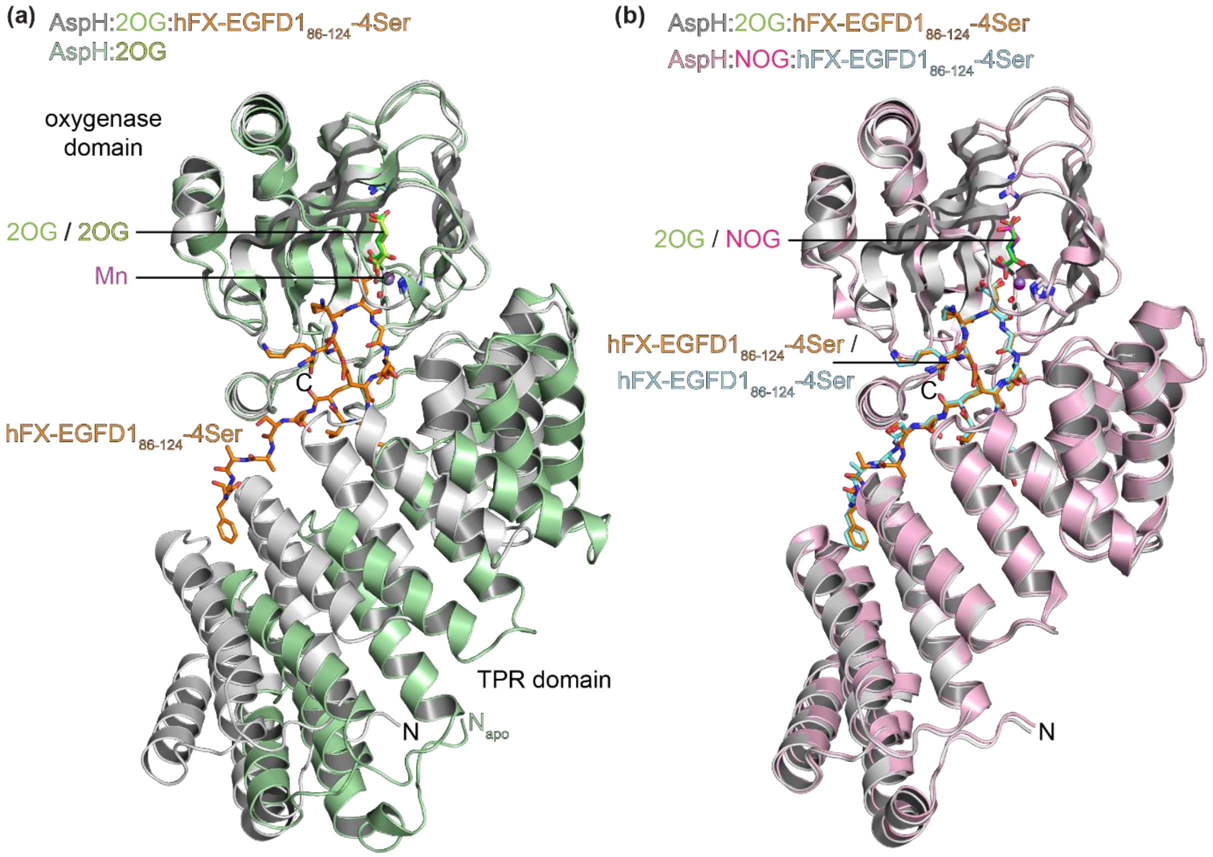

Supporting Figure S10. Conformational changes at the AspH active site of the AspH:2OG complex structure occur on EGFD substrate binding. Colors: violet: Mn; orange: carbon-backbone of the hFX-EGFD1$_{86\text{-}124}$-4Ser peptide (Supporting Figure S1c); red: oxygen; blue: nitrogen.

Superimposition of a view from the active site of the AspH:2OG:hFX-EGFD1$_{86\text{-}124}$-4Ser structure (AspH: grey, carbon-backbone of 2OG: green, carbon-backbone of the hFX-EGFD1$_{86\text{-}124}$-4Ser peptide: orange; Supporting Figure S8) with one from the AspH:2OG structure (AspH: pale green, carbon-backbone of 2OG: lemon; Supporting Figure S7) reveals that the conformations of several (*i.e.* Glu615, Asp616, Glu617, Arg620, Gln627, and Gln632), but not all (*i.e.* Arg686 and Arg688), AspH residues engaged in EGFD substrate binding change on substrate binding (Cα RMSD = 2.59 Å). By contrast, the conformations of those AspH residues directly engaged in 2OG and Fe(II) binding (Ser668, His679, Arg 688, His690, His725, and Arg735; Figure 5) do not alter significantly upon substrate binding. These observations are, in general, in accord with observations made in AspH crystal structures when 2OG was substituted with NOG.[1]

Changes in the conformations of AspH residues occurring on substrate binding include the following: (i) the interaction between Glu617$_{apo}$ and Arg620 (2.5 Å) in the AspH:2OG structure is lost on substrate binding, both Glu617 (2.9, 3.0, and 3.1 Å) and Asp616 (2.7 Å) interact with the substrate in the AspH:2OG:hFX-EGFD1$_{86\text{-}124}$-4Ser structure; (ii) upon substrate binding, the side chain of Glu615 rotates by ~90° to interact with the side chain of Arg620 (2.8 Å) and the main chain amine groups of Glu617 (3.0 Å) and Leu619 (2.7 Å), rather than Lys666 (2.9 Å) as in the substrate unbound state; (iii) Lys666 (2.6 Å) binds to the Asp103$_{hFX}$ carboxylate while Gln627 rotates towards the hFX-EGFD1$_{86\text{-}124}$-4Ser substrate and no longer interacts with Thr629 (2.6 Å) as in the substrate unbound state; (iv) in the presence of EGFD substrate, Gln632 moves to interact with the side chain amine of Lys100$_{hFX}$ (2.9 Å).

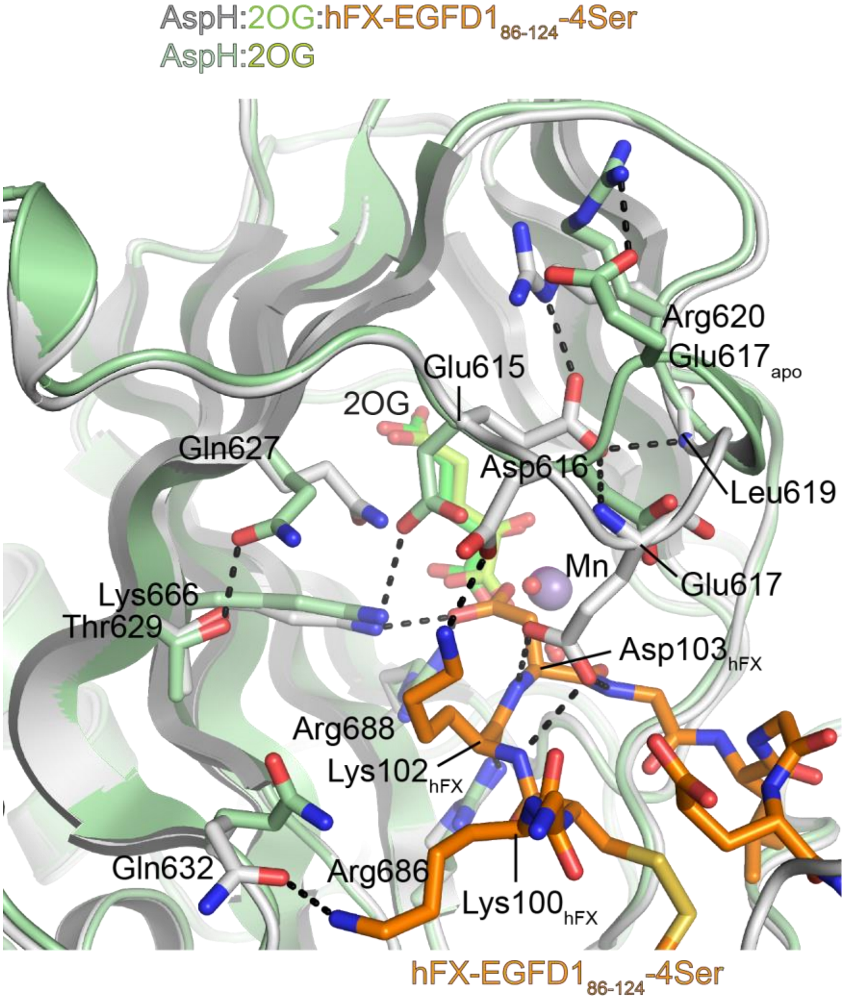

Supporting Figure S11. Views from a crystal structure of AspH complexed with 3-methyl-2OG (16), Mn, and a synthetic EGFD substrate peptide (AspH:16:hFX-EGFD1$_{86\text{-}124}$-4Ser; PDB ID: 6YYX; continues on the following page). Colors: grey: His$_6$-AspH$_{315\text{-}758}$; yellow: carbon-backbone of (*R*)-3-methyl-2OG (**16**); violet: Mn; orange: carbon-backbone of the hFX-EGFD1$_{86\text{-}124}$-4Ser peptide (Supporting Figure S1c); red: oxygen; blue: nitrogen.

(a) Overview of the AspH:**16**:hFX-EGFD1$_{86\text{-}124}$-4Ser crystal structure; (b) OMIT electron density map ($mF_o\text{−}DF_c$) contoured to 3σ around the hFX-EGFD1$_{86\text{-}124}$-4Ser peptide from the AspH:**16**:hFX-EGFD1$_{86\text{-}124}$-4Ser structure reveals electron density for residues Gly99$_{hFX}$ to Phe116$_{hFX}$ including for the disulfide bridged (Cys101$_{hFX}$ and Cys110$_{hFX}$) ten-membered non-canonical EGFD macrocycle. Clear electron density is observed for the side chain of Tyr108$_{hFX}$, an hFX-EGFD1 residue which is part of the AspH-substrate consensus sequence requirement (Supporting Figure S1); the Tyr108$_{hFX}$ side chain interacts with the TPR domain of AspH as revealed in reported AspH:substrate complex structures;[1] (c and d) analyses of the OMIT electron density maps ($mF_o\text{−}DF_c$) contoured to 9σ around (c) (*S*)-**16** (lightbrown) or (d) (*R*)-**16** (yellow), which were both refined in the AspH:**16**:hFX-EGFD1$_{86\text{-}124}$-4Ser structure. The results support the (at least) predominant presence of (*R*)-**16** (d) in the structure as the methyl-group and the C3- and C4-methylenes of (*S*)-**16** (c) do not align with the observed electron density.

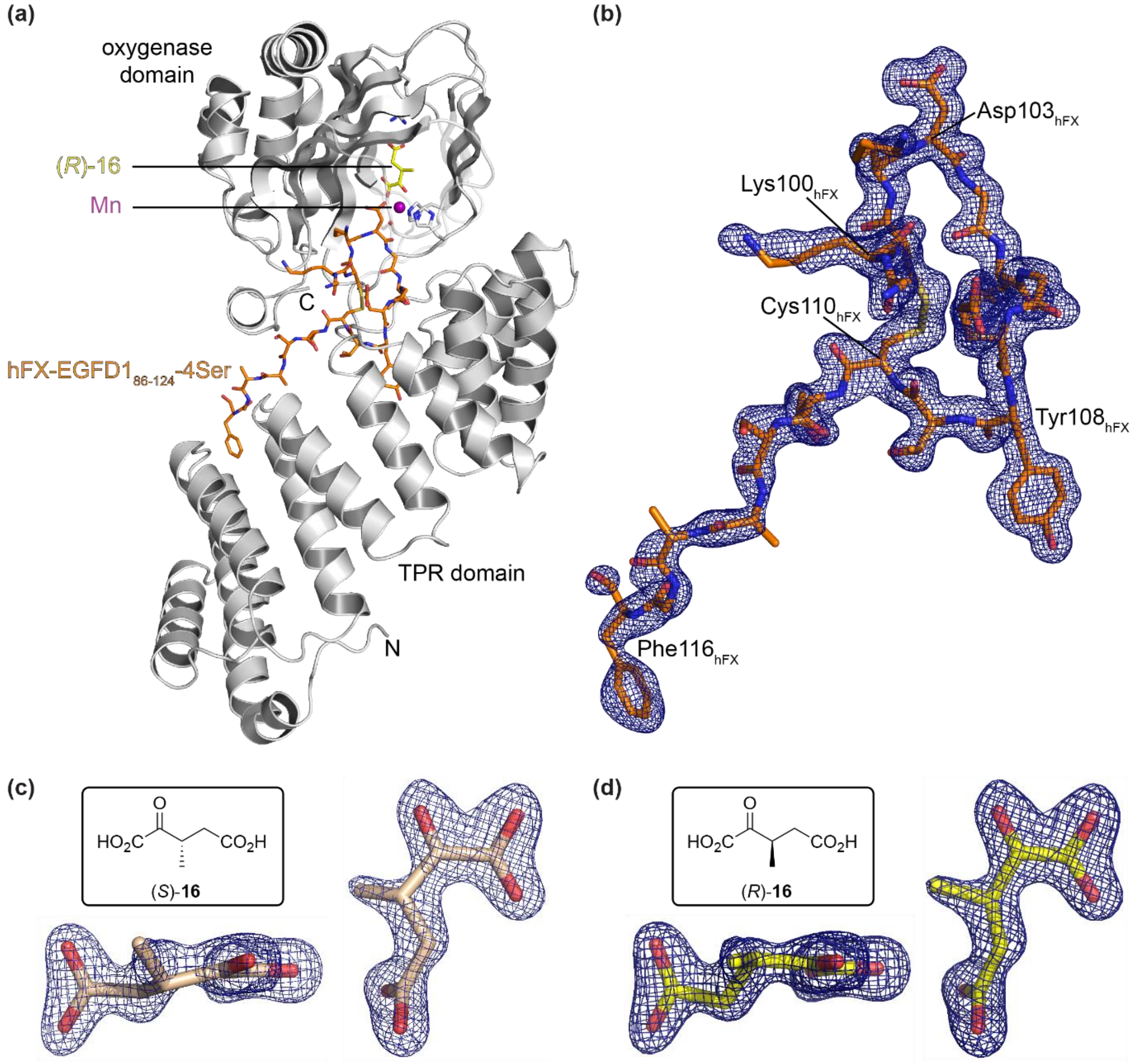

(e and f) B-factor analyses of the OMIT electron density maps ($mF_o{-}DF_c$) contoured to 9σ around either (e) (*S*)-**16** or (f) (*R*)-**16**, which were both refined in the AspH:**16**:hFX-EGFD1$_{86\text{-}124}$-4Ser structure. The results supports the (at least) predominant presence of (*R*)-**16** in the structure as the B-factors for the C3- and C6-carbon atoms of (e) (*S*)-**16** are substantially higher (by ~8 and ~3 Å^2, respectively) than those for (f) (*R*)-**16**.

(e)

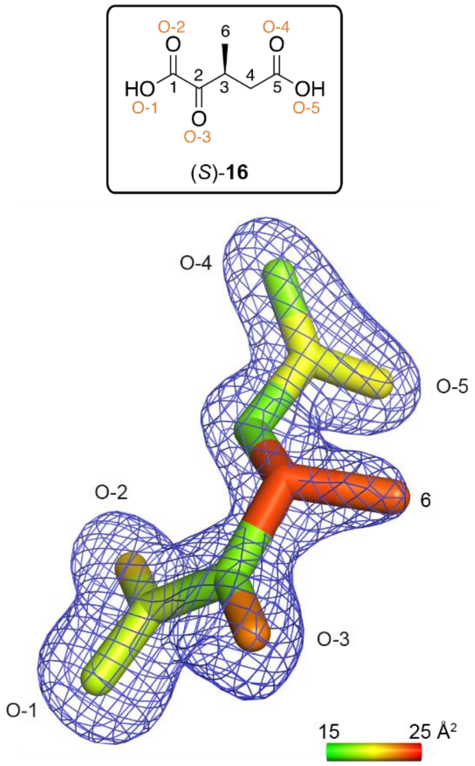

(f)

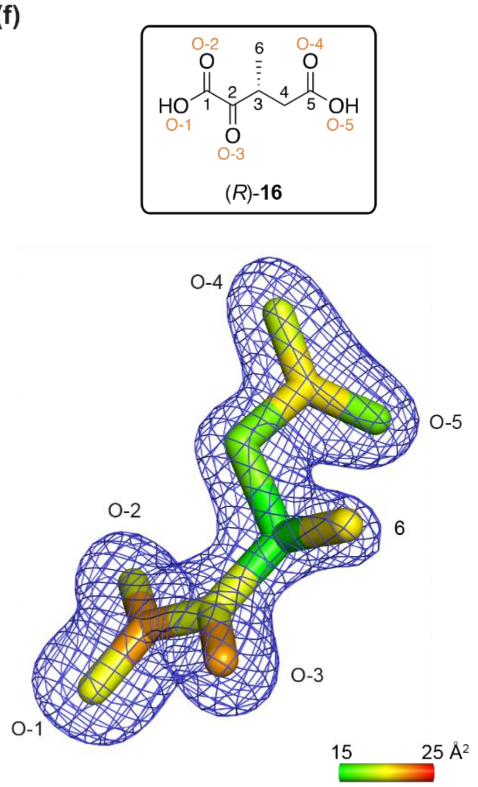

**(*S*)-3-methyl-2OG (16)**	**B-factor** [Å^2]
C1	18.81
C2	17.49
C3	23.73
C4	17.07
C5	19.63
C6	23.33
O-1	18.75
O-2	20.99
O-3	22.47
O-4	17.55
O-5	20.04

**(*R*)-3-methyl-2OG (16)**	**B-factor** [Å^2]
C1	21.90
C2	19.46
C3	15.26
C4	17.27
C5	20.40
C6	20.33
O-1	19.71
O-2	19.49
O-3	21.90
O-4	18.95
O-5	17.38

Supporting Figure S12. Views from a crystal structure of AspH complexed with 3-methyl-2OG (16) and Mn (AspH:16; PDB ID: 6YYV; continues on the following page). Colors: grey: His$_6$-AspH$_{315\text{-}758}$; yellow: carbon-backbone of (*R*)-3-methyl-2OG (**16**); violet: Mn; red: oxygen; blue: nitrogen. w: water.

(a) Overview of the AspH:**16** crystal structure; (b) superimposition of a view from the AspH:**16** structure with one from the AspH:2OG structure (AspH: pale green, carbon-backbone of 2OG: green; Supporting Figure S7) reveals similar AspH conformations (Cα RMSD = 0.38 Å). However, the relative arrangement of TPR repeats α1 to α6 (numbered from the AspH N-terminus)[1] differs slightly in the two structures (*e.g.* the Pro372 Cα atoms in the two structures are ~2.5 Å apart); these differences, however, do not affect the distance between the oxygenase and TPR domains, which together form the substrate binding pocket (Supporting Figures S9 and S13); (c and d) analyses of the OMIT electron density maps ($mF_o{-}DF_c$) contoured to 9σ around (c) (*S*)-**16** (lightbrown) or (d) (*R*)-**16** (yellow), which were both refined in the AspH:**16** structure. The results support the (at least) predominant presence of (*R*)-**16** (d) in the structure as the C3-methylene group of (*S*)-**16** (c) does not align with the observed electron density.

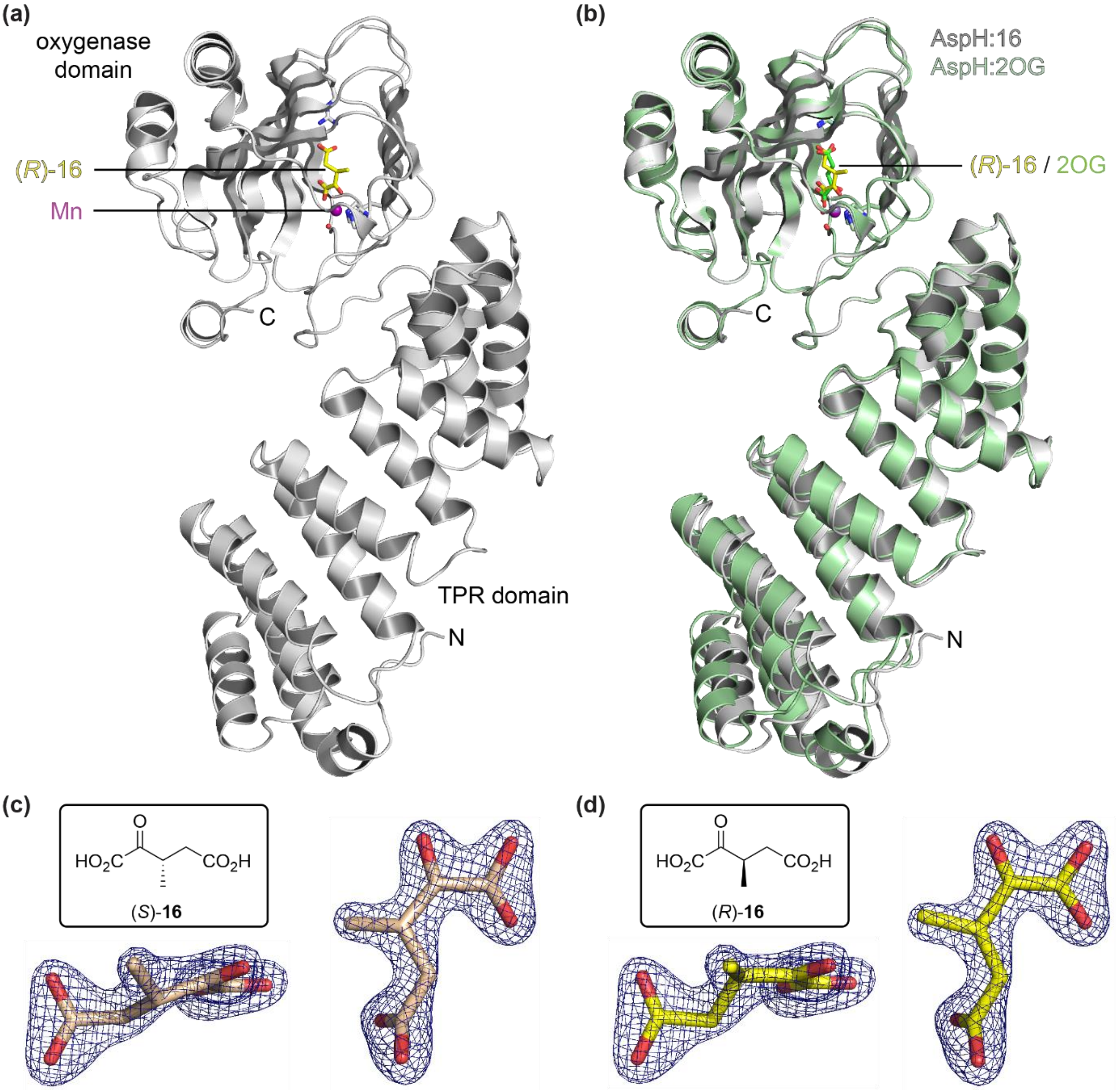

(e and f) B-factor analyses of the OMIT electron density maps (mF_o-DF_c) contoured to 9σ around either (e) (*S*)-**16** or (f) (*R*)-**16**, which were both refined in the AspH:**16** structure. The results support the (at least) predominant presence of (*R*)-**16** in the structure as the B-factor of the C3-carbon atom of (e) (*S*)-**16** is substantially higher (by ~20 Å^2) than the one for (f) (*R*)-**16**.

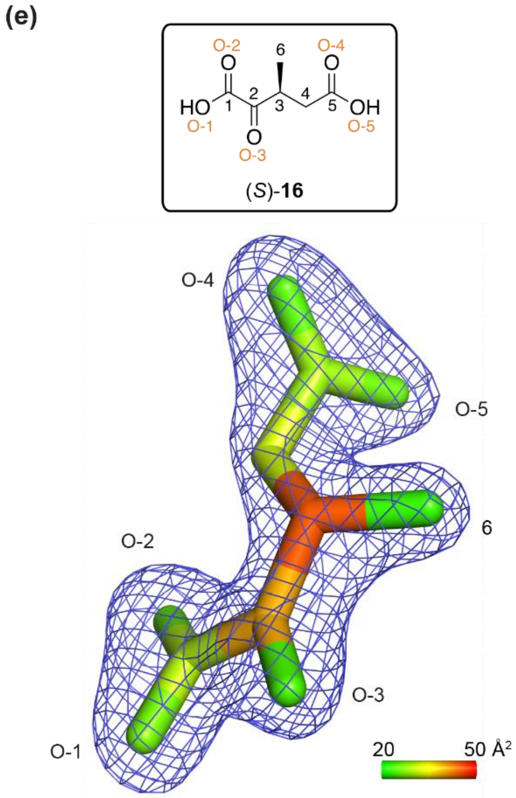

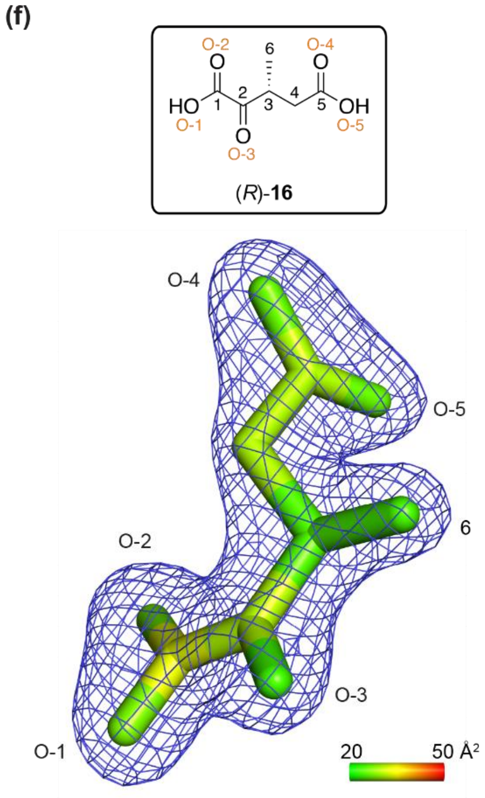

**(*S*)-3-methyl-2OG (16)**	**B-factor** [Å^2]
C1	30.86
C2	39.71
C3	44.81
C4	32.44
C5	29.18
C6	23.13
O-1	26.77
O-2	25.62
O-3	24.50
O-4	24.89
O-5	26.23

**(*R*)-3-methyl-2OG (16)**	**B-factor** [Å^2]
C1	33.81
C2	30.74
C3	24.82
C4	30.45
C5	30.24
C6	29.30
O-1	27.37
O-2	27.13
O-3	23.97
O-4	26.11
O-5	26.34

Supporting Figure S13. Replacing the reported AspH cosubstrate 2OG by 3-methyl-2OG (16) does not cause changes in the conformation of the crystallographically observed AspH active site residues. Colors: red: oxygen; blue: nitrogen.

(a) Superimposition (Cα RMSD = 2.79 Å) of a view from the AspH:**16**:hFX-EGFD1$_{86\text{-}124}$-4Ser structure (AspH: grey, carbon-backbone of (*R*)-**16**: yellow, carbon-backbone of the hFX-EGFD1$_{86\text{-}124}$-4Ser peptide: orange; Supporting Figure S11) with one from the AspH:**16** structure (AspH: pale yellow, carbon-backbone of (*R*)-**16**: brown; Supporting Figure S12) reveals that the distance between the Cα atoms of Leu433 on TPR repeat α6 and Pro756 in the AspH C-terminal region decreases from ~21 Å to ~14 Å upon substrate binding. Evidence for an induced fit mechanism involving this conformational change has been described when AspH was crystallized in the presence of 2OG (Supporting Figure S9) or *N*-oxalylglycine (NOG)[1] rather than **16**; (b) superimposition of a view from the AspH:**16**:hFX-EGFD1$_{86\text{-}124}$-4Ser structure (AspH: grey, carbon-backbone of (*R*)-**16**: yellow, carbon-backbone of the hFX-EGFD1$_{86\text{-}124}$-4Ser peptide: orange; Supporting Figure S11) with one from the AspH:2OG:hFX-EGFD1$_{86\text{-}124}$-4Ser structure (AspH: pale green, carbon-backbone of 2OG: green, carbon-backbone of the hFX-EGFD1$_{86\text{-}124}$-4Ser peptide: aquamarine; Supporting Figure S12) reveals similar AspH (Cα RMSD = 0.23 Å) and substrate (Cα RMSD = 0.19 Å) conformations.

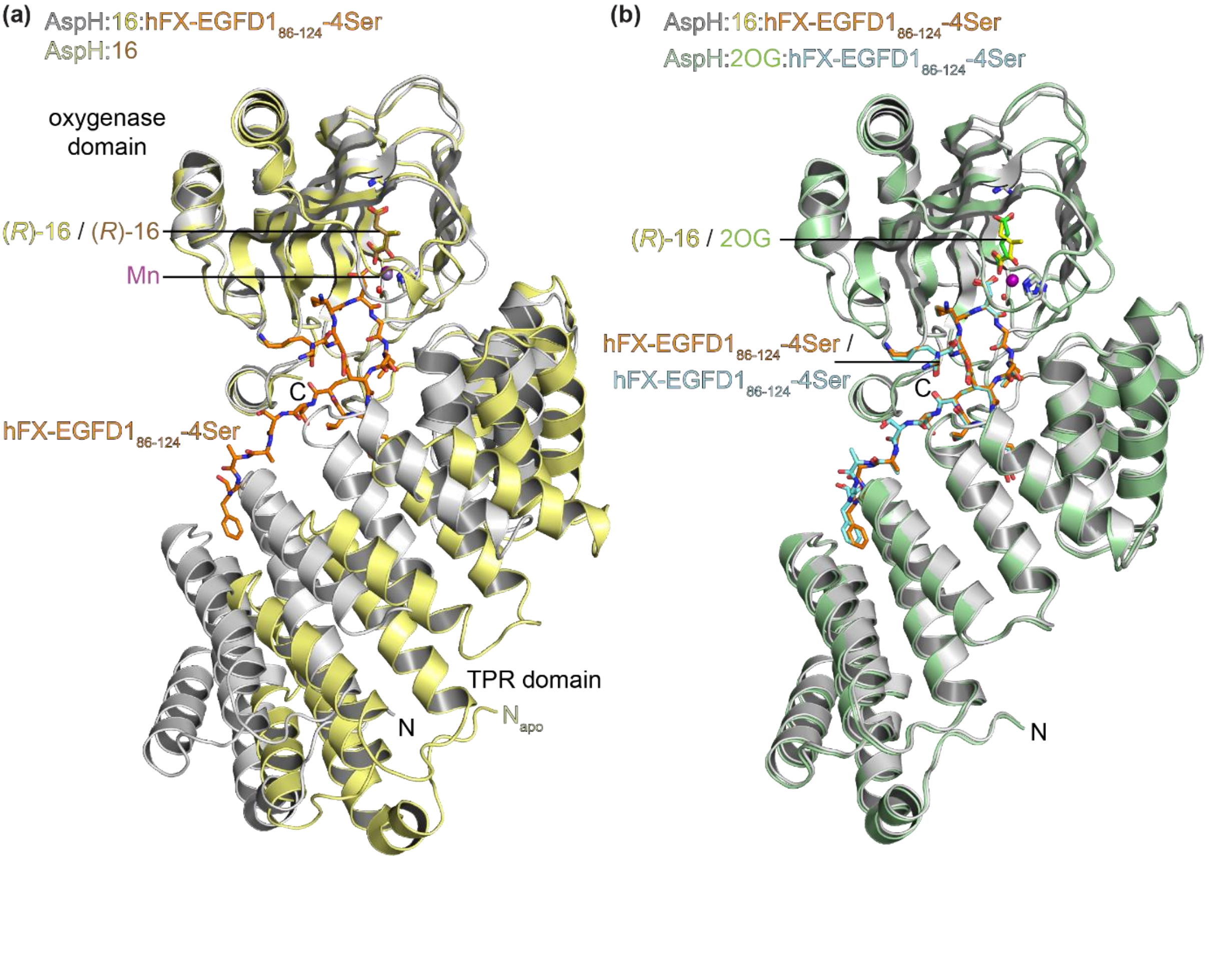

Supporting Figure S14. Views from a crystal structure of AspH complexed with 3-ethyl-2OG (17), Mn, and a synthetic EGFD substrate peptide (AspH:34:hFX-EGFD1$_{86\text{-}124}$-4Ser; PDB ID: 6Z6Q; continues on the following page). Colors: grey: His$_6$-AspH$_{315\text{-}758}$; salmon: carbon-backbone of (*R*)-3-ethyl-2OG (**17**); violet: Mn; orange: carbon-backbone of the hFX-EGFD1$_{86\text{-}124}$-4Ser peptide (Supporting Figure S1c); red: oxygen; blue: nitrogen.

(a) Overview of the AspH:**17**:hFX-EGFD1$_{86\text{-}124}$-4Ser crystal structure. (*R*)-**17** forms a salt bridge to Arg735 (2.8 and 2.9 Å) with its C5-carboxylate and is positioned to interact with Ser668 (2.3 Å) through its C5-carboxylate, with His690 (2.8 Å) and Arg688 (2.6 Å) through its C1-carboxylate, and with the Mn ion via its C1-carboxylate (2.3 Å) and C2-carbonyl (1.9 Å). The Mn ion additionally coordinates His679 (2.3 Å), His725 (2.2 Å), a water molecule (2.1 Å), and is positioned to interact with the side-chain carboxylate of Asp103$_{hFX}$ (2.9 Å); (b) OMIT electron density map (mF_o−DF_c) contoured to 3σ around the hFX-EGFD1$_{86\text{-}124}$-4Ser peptide from the AspH:**17**:hFX-EGFD1$_{86\text{-}124}$-4Ser structure reveals electron density for residues Gly99$_{hFX}$ to Thr111$_{hFX}$ including for the disulfide bridged (Cys101$_{hFX}$ and Cys110$_{hFX}$) ten-membered non-canonical EGFD macrocycle. Clear electron density is observed for the side chain of Tyr108$_{hFX}$, an hFX-EGFD1 residue which is part of the AspH-substrate consensus sequence requirement (Supporting Figure S1); the Tyr108$_{hFX}$ side chain interacts with the TPR domain of AspH as revealed in reported AspH:substrate complex crystal structures.[1] Note that in the AspH:**17**:hFX-EGFD1$_{86\text{-}124}$-4Ser structure, the Asp103$_{hFX}$ side-chain carboxylate adopts two conformations, as observed in AspH:substrate complex structures;[1] (c and d) analyses of the OMIT electron density maps (mF_o−DF_c) contoured to 9σ around (c) (*S*)-**17** (dark yellow) or (d) (*R*)-**17** (salmon), which were both refined in the AspH:**17**:hFX-EGFD1$_{86\text{-}124}$-4Ser structure. The results support the (at least) predominant presence of (*R*)-**17** in the structure as the C3-methylene of (*S*)-**17** orients in a manner that the C3-ethyl substituent does not correlate well with the observed electron density; (e) superimposition of the refined conformations of the (*S*)- and (*R*)-enantiomers of **17** reveal differences in the conformations of the C3-methylene group and the C3-ethyl substituent.

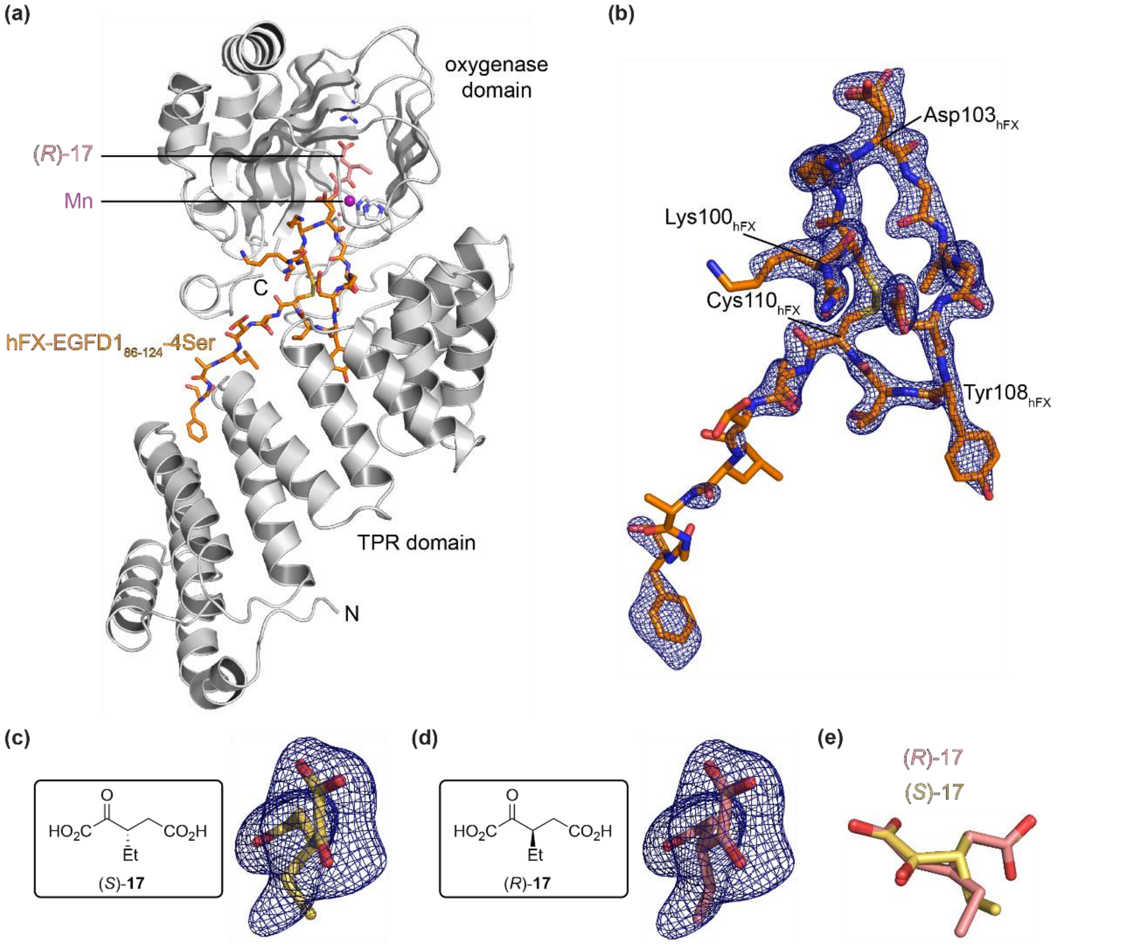

(f and g) B-factor analyses of the OMIT electron density maps ($mF_o{-}DF_c$) contoured to 3σ around either (f) (*S*)-**17** or (g) (*R*)-**17**, which were both refined in the AspH:**17**:hFX-EGFD1$_{86\text{-}124}$-4Ser structure. The results support the (at least) predominant presence of (f) (*R*)-**17** in the structure as the B-factors of carbon atoms C3-C7 and oxygen atoms O4 and O5 of (*S*)-**17** are substantially higher than those for (*R*)-**17**. The most pronounced effect on the B-factors was observed for the C6- and C7-atoms, which are ~8.5 respectively ~9 Å^2 higher for (f) (*S*)-**17** than for (g) (*R*)-**16**.

(f)

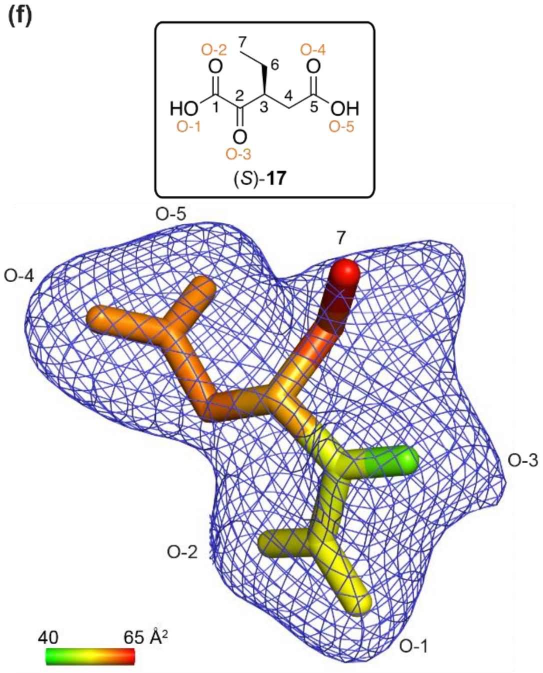

(g)

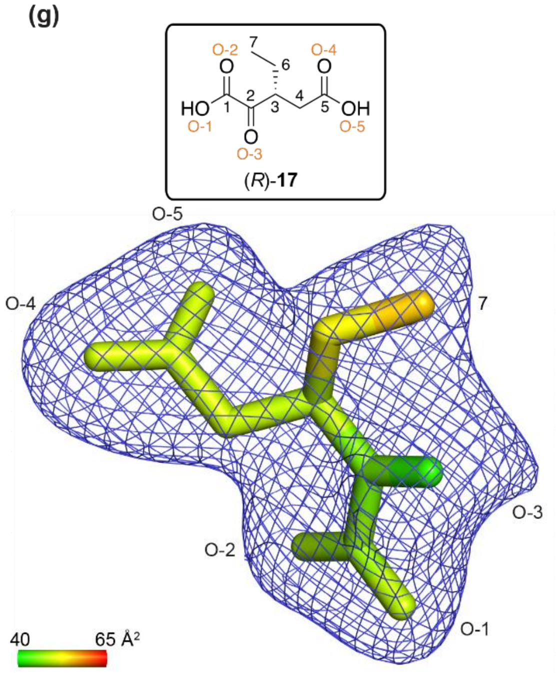

**(*S*)-3-ethyl-2OG (17)**	**B-factor** [Å^2]
C1	52.30
C2	51.24
C3	56.82
C4	59.22
C5	58.68
C6	61.14
C7	64.07
O-1	52.59
O-2	52.56
O-3	44.97
O-4	58.30
O-5	58.25

**(*R*)-3-ethyl-2OG (17)**	**B-factor** [Å^2]
C1	47.75
C2	46.14
C3	49.27
C4	49.97
C5	49.76
C6	52.47
C7	54.83
O-1	49.50
O-2	48.79
O-3	42.41
O-4	50.14
O-5	49.91

Supporting Figure S15. AspH adopts similar conformations when complexed to the AspH inhibitor 3-ethyl-2OG (17), and the AspH cosubstrates 2OG and 3-methyl-2OG (16). Colors: grey: His_6-$AspH_{315-758}$; salmon: carbon-backbone of (*R*)-3-ethyl-2OG (**17**); violet: Mn; orange: carbon-backbone of the hFX-EGFD1_{86-124}-4Ser peptide (Supporting Figure S1c); red: oxygen; blue: nitrogen.

(a) Superimposition of a view from the AspH:**17**:hFX-EGFD1_{86-124}-4Ser structure (Supporting Figure S14) with one from the AspH:2OG:hFX-EGFD1_{86-124}-4Ser structure (AspH: pale green, carbon-backbone of 2OG: green carbon-backbone of the hFX-EGFD1_{86-124}-4Ser peptide: aquamarine; Supporting Figure S8) reveals similar AspH (Cα RMSD = 0.23 Å) and substrate (Cα RMSD = 0.70 Å) conformations. Note that in the AspH:**17**:hFX-EGFD1_{86-124}-4Ser structure, the $Asp103_{hFX}$ side-chain carboxylate adopts two conformations compared to only one conformation observed in the AspH:2OG:hFX-EGFD1_{86-124}-4Ser structure; (b) superimposition of a view from the AspH:**17**:hFX-EGFD1_{86-124}-4Ser structure (Supporting Figure S14) with one from the AspH:**16**:hFX-EGFD1_{86-124}-4Ser structure (AspH: pale yellow, carbon-backbone of (*R*)-**16**: yellow, carbon-backbone of the hFX-EGFD1_{86-124}-4Ser peptide: aquamarine; Supporting Figure S11) reveals similar AspH (Cα RMSD = 0.18 Å) and substrate (Cα RMSD = 0.49 Å) conformations. Note that in the AspH:**17**:hFX-EGFD1_{86-124}-4Ser structure, the $Asp103_{hFX}$ side-chain carboxylate adopts two conformations compared to only one conformation observed in the AspH:**16**:hFX-EGFD1_{86-124}-4Ser structure.

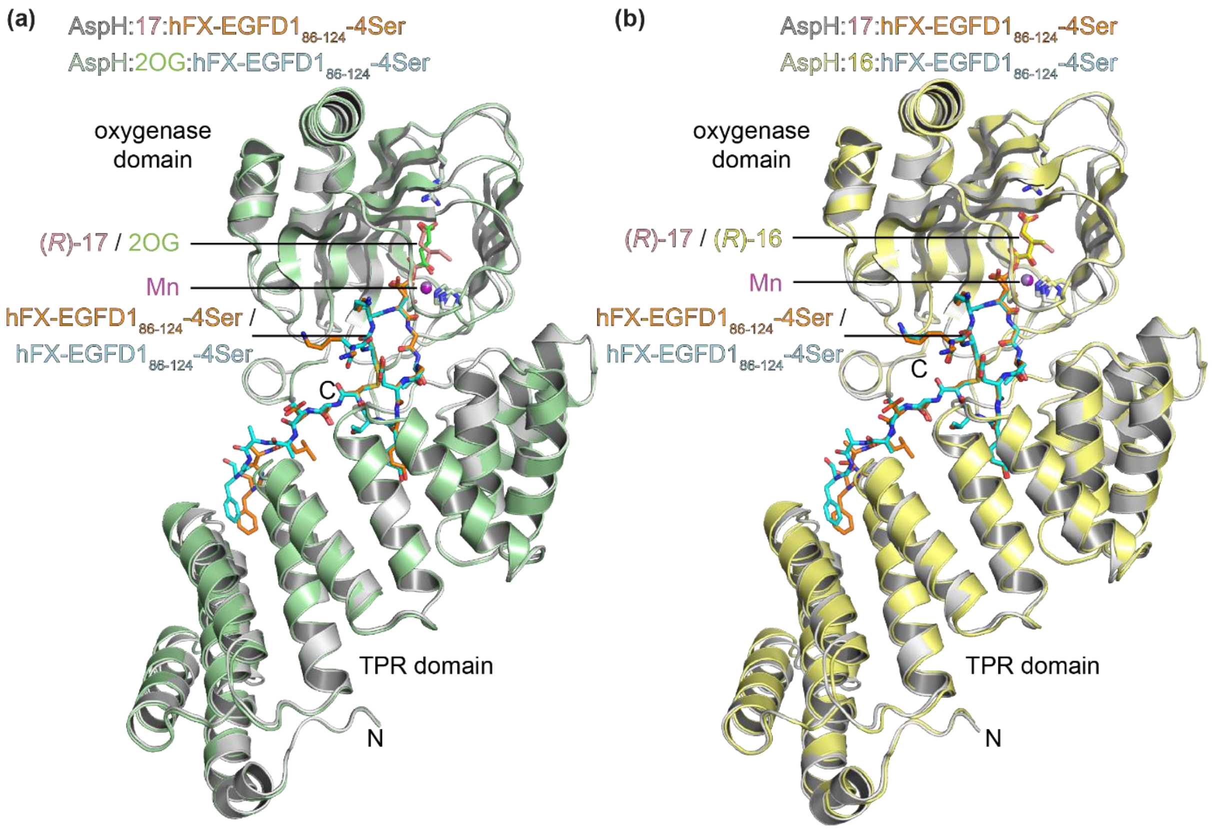

Supporting Figure S16. Views from a crystal structure of AspH complexed with 4,4-dimethyl-2OG (34), Mn, and a synthetic EGFD substrate peptide (AspH:34:hFX-EGFD1$_{86\text{-}124}$-4Ser; PDB ID: 6YYY). Colors: grey: His$_6$-AspH$_{315\text{-}758}$; slate blue: carbon-backbone of 4,4-dimethyl-2OG (**34**); violet: Mn; orange: carbon-backbone of the hFX-EGFD1$_{86\text{-}124}$-4Ser peptide (Supporting Figure S1c); red: oxygen; blue: nitrogen.

(a) Overview of the AspH:**34**:hFX-EGFD1$_{86\text{-}124}$-4Ser crystal structure. 4,4-Dimethyl-2OG (**34**) forms a salt bridge to Arg735 (2.5 and 2.6 Å) with its C5-carboxylate and is positioned to interact with Ser668 (2.6 Å) through its C5-carboxylate, with His690 (3.3 Å) and Arg688 (3.0 Å) through its C1-carboxylate, and with the Mn ion via its C1-carboxylate (2.3 Å) and C2-carbonyl (2.3 Å). The Mn ion additionally coordinates His679 (2.4 Å), His725 (2.3 Å), a water molecule (2.3 Å), and is positioned to interact with the *pro-R* hydrogen at the Asp103$_{hFX}$ β-position (distance Cβ-Mn: 4.0 Å); (b) OMIT electron density map (mF$_o$−DF$_c$) contoured to 3σ around the hFX-EGFD1$_{86\text{-}124}$-4Ser peptide from the AspH:**34**:hFX-EGFD1$_{86\text{-}124}$-4Ser structure reveals electron density for residues Lys100$_{hFX}$ to Phe116$_{hFX}$ including for the disulfide bridged (Cys101$_{hFX}$ and Cys110$_{hFX}$) ten-membered non-canonical EGFD macrocycle. Clear electron density is observed for the side chain of Tyr108$_{hFX}$, an hFX-EGFD1 residue which is part of the AspH-substrate consensus sequence requirement (Supporting Figure S1); the Tyr108$_{hFX}$ side chain interacts with the TPR domain of AspH as revealed in reported AspH:substrate complex structures;[1] (c) Note that the *pro-R* methyl group of **34** faces towards the hydrophobic isopropyl side chains of Val676 and Val727 which, together with the side chain of Met670, form one face of a hydrophobic pocket. By contrast, the *pro-S* methyl group of **34** faces towards the indole ring of Trp625 while still being in proximity to the side chain of Met670.

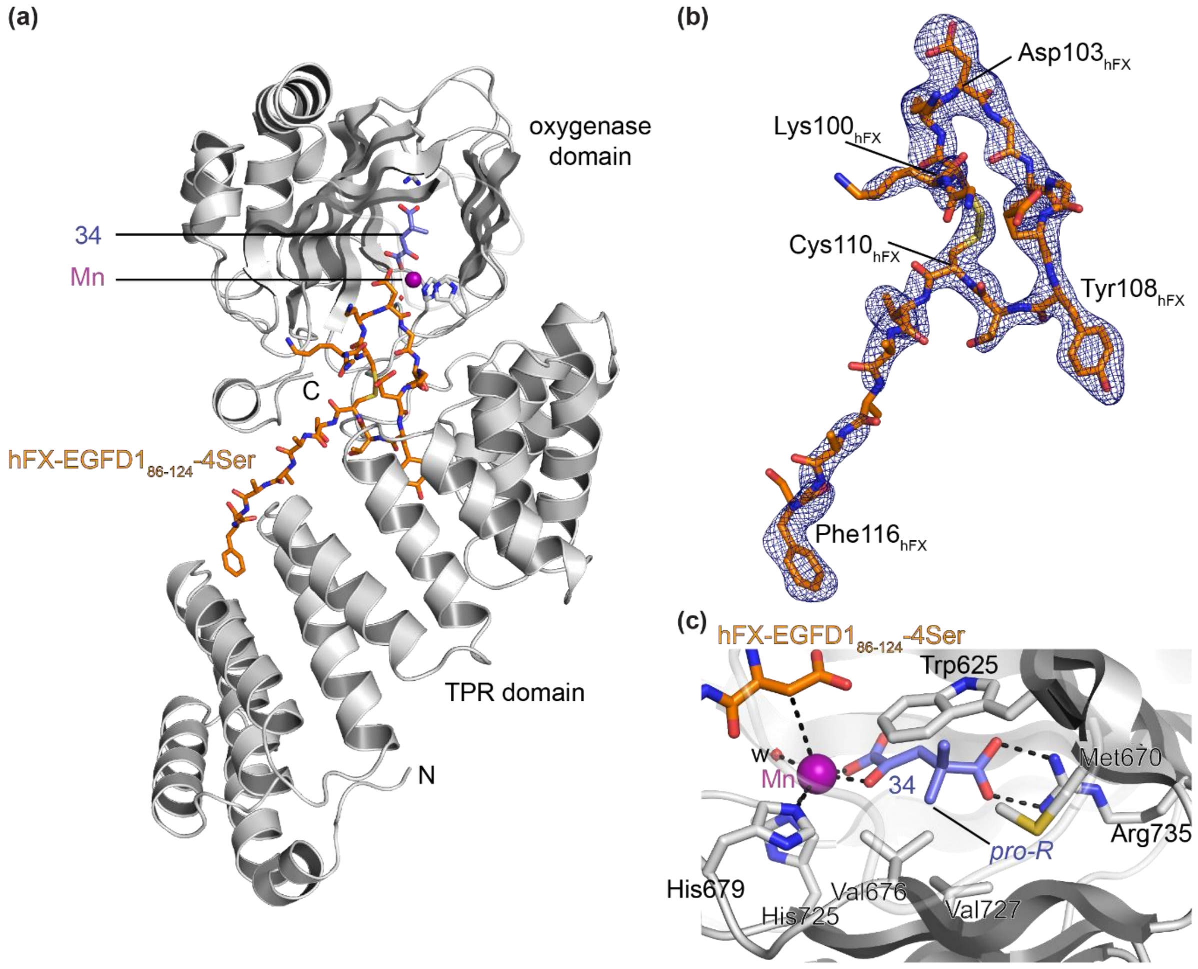

Supporting Figure S17. AspH adopts similar conformations when complexed to the AspH inhibitor 4,4-dimethyl-2OG (34), 2OG, and NOG. Colors: grey: violet: Mn; red: oxygen; blue: nitrogen.

(a) Superimposition of a view from the AspH:**34**:hFX-EGFD1$_{86\text{-}124}$-4Ser structure (AspH: grey, carbon-backbone of 4,4-dimethyl-2OG (**34**): slate blue, carbon-backbone of the hFX-EGFD1$_{86\text{-}124}$-4Ser peptide: orange; Supporting Figure S16) with one from the AspH:2OG:hFX-EGFD1$_{86\text{-}124}$-4Ser structure (AspH: pale green, carbon-backbone of 2OG: green, carbon-backbone of the hFX-EGFD1$_{86\text{-}124}$-4Ser peptide: aquamarine; Supporting Figure S8) reveals similar AspH (Cα RMSD = 0.46 Å) and substrate (Cα RMSD = 0.38 Å) conformations; (b) superimposition of a view from the AspH:**34**:hFX-EGFD1$_{86\text{-}124}$-4Ser structure (AspH: grey, carbon-backbone of 4,4-dimethyl-2OG (**34**): slate blue, carbon-backbone of the hFX-EGFD1$_{86\text{-}124}$-4Ser peptide: orange; Supporting Figure S16) with one from the reported AspH:NOG:hFX-EGFD1$_{86\text{-}124}$-4Ser structure (AspH: pale pink, carbon-backbone of *N*-oxalylglycine (NOG, **3**): magenta, carbon-backbone of the hFX-EGFD1$_{86\text{-}124}$-4Ser peptide: aquamarine; PDB ID: 5JQY)[1] reveals similar AspH (Cα RMSD = 0.53 Å) and substrate (Cα RMSD = 0.53 Å) conformations. Note that in the AspH:**34**:hFX-EGFD1$_{86\text{-}124}$-4Ser structure, the Asp103$_{hFX}$ side-chain carboxylate adopts only one conformation compared to the two conformations observed for it in the reported AspH:NOG:hFX-EGFD1$_{86\text{-}124}$-4Ser structure.[1]

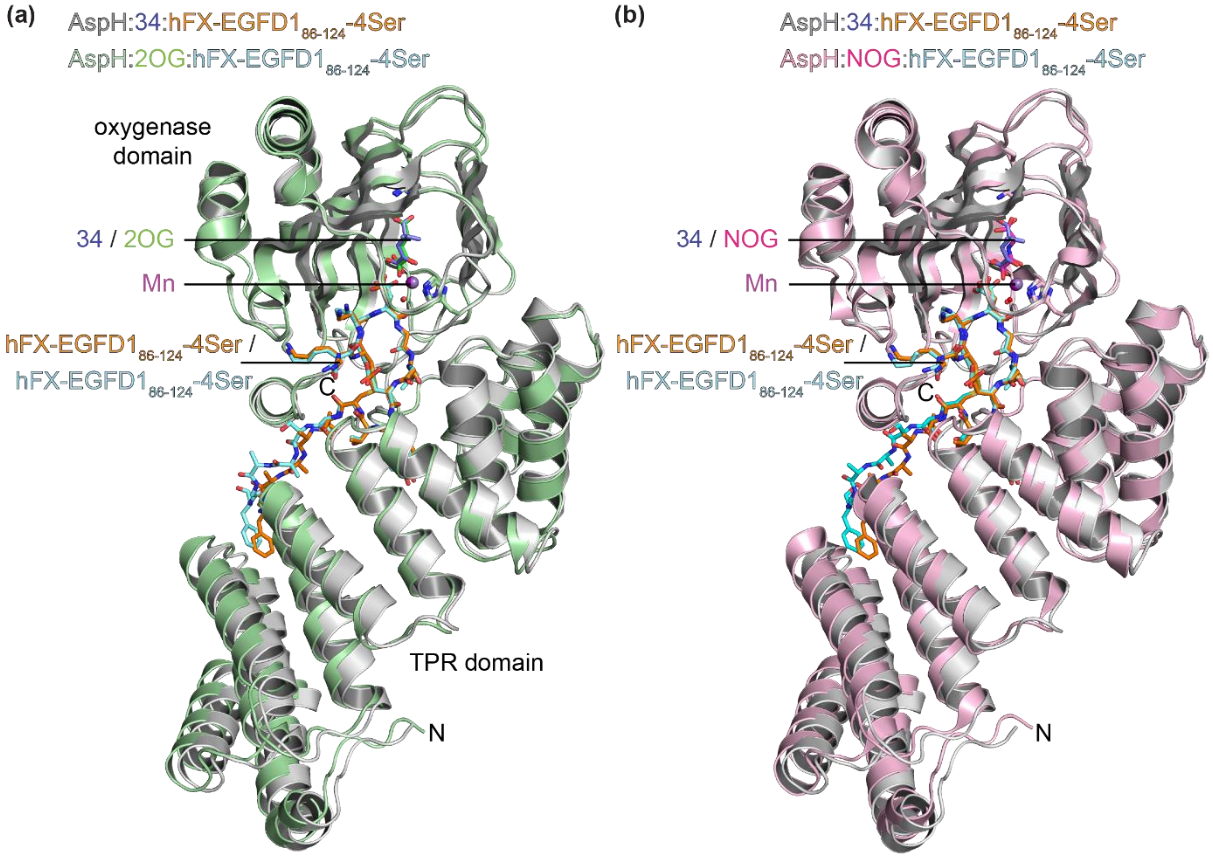

Supporting Figure S18. Views from a crystal structure of AspH complexed with *N*-oxalyl-α-methylalanine (49), Mn, and a synthetic EGFD substrate peptide (AspH:49:hFX-EGFD1$_{86\text{-}124}$-4Ser; PDB ID: 6Z6R). Colors: grey: His$_6$-AspH$_{315\text{-}758}$; olive: carbon-backbone of *N*-oxalyl-α-methylalanine (**49**); violet: Mn; orange: carbon-backbone of the hFX-EGFD1$_{86\text{-}124}$-4Ser peptide (Supporting Figure S1c); red: oxygen; blue: nitrogen.

(a) *N*-Oxalyl-α-methylalanine (**49**); (b) overview of a fold of the AspH:**49**:hFX-EGFD1$_{86\text{-}124}$-4Ser crystal structure; (c) OMIT electron density map (mF_o−DF_c) contoured to 3σ around the hFX-EGFD1$_{86\text{-}124}$-4Ser peptide from the AspH:**49**:hFX-EGFD1$_{86\text{-}124}$-4Ser structure reveals electron density for residues Gly99$_{hFX}$ to Thr111$_{hFX}$ including for the disulfide bridged (Cys101$_{hFX}$ and Cys110$_{hFX}$) ten-membered non-canonical EGFD macrocycle; (d) representative OMIT electron density map (mF_o-DF_c) contoured to 3σ around **49** of the AspH:**49**:hFX-EGFD1$_{86\text{-}124}$-4Ser structure. The C5-carboxylate of **49** is positioned to interact with the side chains of Arg735 (2.7 and 3.0 Å) and of Ser668 (2.8 and 3.2 Å). **49** is positioned to interact with His690 (3.1 Å) and Arg688 (3.2 and 3.2 Å) through its C1-carboxylate, and with the Mn ion via its C1-carboxylate (2.2 Å) and C2-carbonyl (2.2 Å). The Mn ion is also complexed by His679 (2.1 Å), His725 (2.2 Å), and a water molecule (2.0 Å); the *pro-R* hydrogen at the Asp103$_{hFX}$ β-position is positioned close to the Mn ion (Cβ-Mn: 4.6 Å), consistent with hydroxylation at this position during productive catalysis with an appropriate cosubstrate (**49** is an AspH inhibitor).

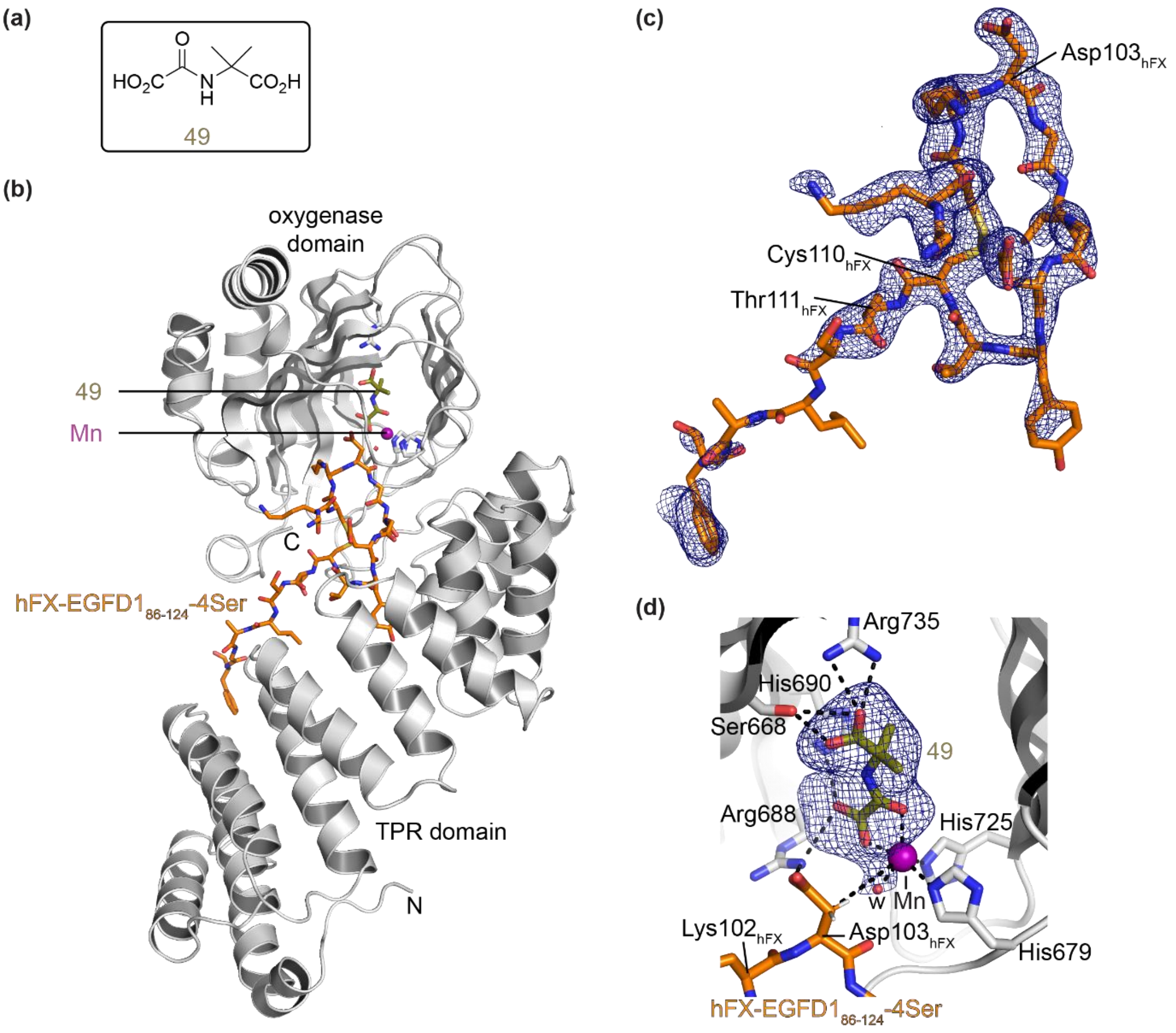

Supporting Figure S19. The AspH inhibitors *N*-oxalyl-α-methylalanine (49) and NOG adopt similar conformations when bound to AspH which are distinct from the conformation of the AspH inhibitor 4,4-dimethyl-2OG (34). Colors: grey: violet: Mn; red: oxygen; blue: nitrogen.

(a) Superimposition of a view from the AspH:**49**:hFX-EGFD1$_{86-124}$-4Ser structure (AspH: grey, carbon-backbone of *N*-oxalyl-α-methylalanine (**49**): olive, carbon-backbone of the hFX-EGFD1$_{86-124}$-4Ser peptide: orange; Supporting Figure S18) with one from the reported AspH:NOG:hFX-EGFD1$_{86-124}$-4Ser structure (AspH: pale pink, carbon-backbone of *N*-oxalylglycine (NOG): magenta, carbon-backbone of the hFX-EGFD1$_{86-124}$-4Ser peptide: brown; PDB ID: 5JQY)[1] reveals similar AspH (Cα RMSD = 0.26 Å) and substrate (Cα RMSD = 0.49 Å) conformations; (b) superimposition of a view from the AspH:**49**:hFX-EGFD1$_{86-124}$-4Ser structure (AspH: grey, carbon-backbone of *N*-oxalyl-α-methylalanine (**49**): olive, carbon-backbone of the hFX-EGFD1$_{86-124}$-4Ser peptide: orange; Supporting Figure S18) with one from the AspH:**34**:hFX-EGFD1$_{86-124}$-4Ser structure (AspH: pale blue, carbon-backbone of 4,4-dimethyl-2OG (**34**): slate blue, carbon-backbone of the hFX-EGFD1$_{86-124}$-4Ser peptide: aquamarine; Supporting Figure S16) similar AspH (Cα RMSD = 0.56 Å) and substrate (Cα RMSD = 0.34 Å) conformations; (c) superimposition of views from AspH active site residues of the AspH:**49**:hFX-EGFD1$_{86-124}$-4Ser structure and the reported AspH:NOG:hFX-EGFD1$_{86-124}$-4Ser structure (AspH: pale pink, carbon-backbone of NOG: magenta, Mn ion: lavender; PDB ID: 5JQY)[1] indicates that the conformations of **49** and NOG are similar; (d) superimposition of views from AspH active site residues of the AspH:**49**:hFX-EGFD1$_{86-124}$-4Ser structure and the AspH:**34**:hFX-EGFD1$_{86-124}$-4Ser structure (AspH: pale blue, carbon-backbone of **34**: slate blue, Mn ion: lavender) indicates that the conformations of **49** and **34** differ substantially, potentially reflecting the reduced rotational freedom of the NH-group resulding in a near linear arrangement of the oxalyl- and the NH-group in **49** resulting in a different relative arrangement of the dimethyl-substituents of **49** and **34**.

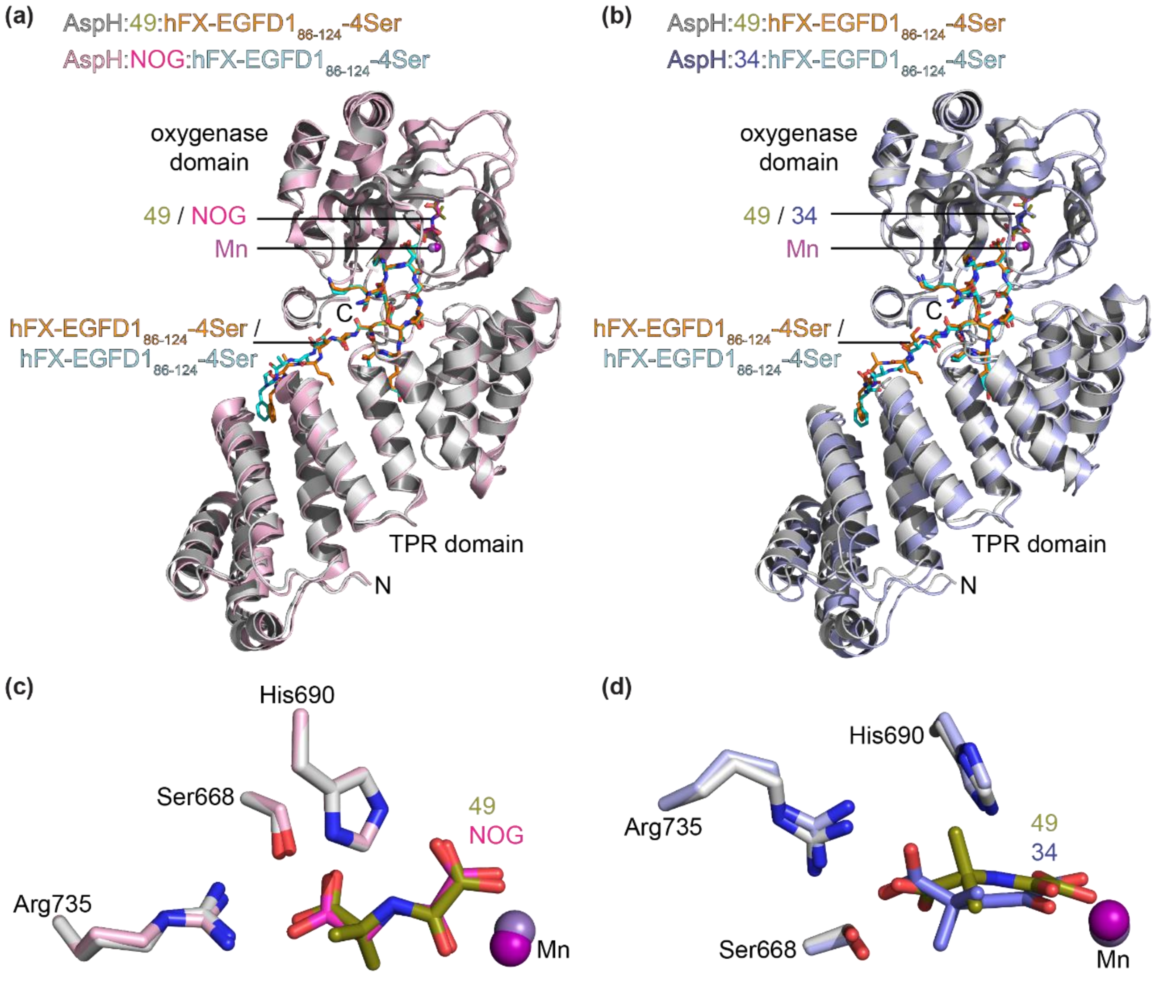

Supporting Figure S20. AspH catalyzes the oxidative decarboxylation of 4,4-dimethyl-2OG (34) to give 2,2-dimethylsuccinate but does not catalyse the oxidative conversion of 2OG derivative 32 (continues on the following page). The AspH-catalyzed oxidative decarboxylation of 2OG derivatives **34** and **32** was monitored using a Bruker AVIII 700 NMR machine equipped with a 5mm $^{1}H(^{13}C/^{15}N)$ inverse cryoprobe. Assay conditions: 10 µM His_6-$AspH_{315-758}$, 150 µM hFX-$CP_{101-119}$ (Supporting Figure S1d), 150 µM 2OG derivative, and 50 µM ammonium iron(II) sulfate hexahydrate (FAS, $(NH_4)_2Fe(SO_4)_2 \cdot 6H_2O$) in 50 mM aqueous Tris-$d_{11}$ containing $10\%_{v/v}$ D_2O (pH 7.5, 25° C). The ratio of the hFX-$CP_{101-119}$ peptide and the hydroxylated hFX-$CP_{101-119}$ peptide, which is the product of the AspH-catalyzed reaction, was determined using SPE-MS (Supporting Information, Section 9) after completion of the NMR experiments.

(I) Experiments using 4,4-dimethyl-2OG (**34**) as an AspH cosubstrate: (a) Close-up (3.0-0.9 ppm) of the ^{1}H NMR spectrum of synthetic **34** in 50 mM aqueous Tris-d_{11} containing $10\%_{v/v}$ D_2O (pH 7.5, 25° C); (b) Close-up (3.0-0.9 ppm) of the ^{1}H NMR spectrum of commercial 2,2-dimethylsuccinate (**52**) in 50 mM aqueous Tris-d_{11} containing $10\%_{v/v}$ D_2O (pH 7.5, 25° C); (c) a timecourse NMR experiment (t = 5 min, top blue spectrum; t = 3 h, bottom black spectrum) reveals that AspH slowly converts **34** into 2,2-dimethylsuccinate (**52**) under the assay conditions. The AspH-catalyzed oxidative decarboxylation of **34** is less efficient than the oxidative decarboxylation reactions of 2OG or 2OG derivatives **16** and **41**, which were complete within 5 min (Supporting Figure S5); (d) Analysis of the final reaction mixture (t = 15 h) by SPE-MS reveals complete hydroxylation (~95% conversion) of the hFX-$CP_{101-119}$ peptide (m/z = 1022.5 corresponds to the +2 charge state of hFX-$CP_{101-119}$; m/z = 1030.5 corresponds to the +2 charge state of hydroxylated hFX-$CP_{101-119}$), indicating that approximately equimolar amounts of 2,2-dimethylsuccinate (**52**) and hydroxylated hFX-$CP_{101-119}$ peptide are present in the final reaction mixture. Thus, the AspH-catalyzed oxidative decarboxylation of **34** to give 2,2-dimethylsuccinate (**52**) is highly coupled with AspH-catalyzed substrate hydroxylation.

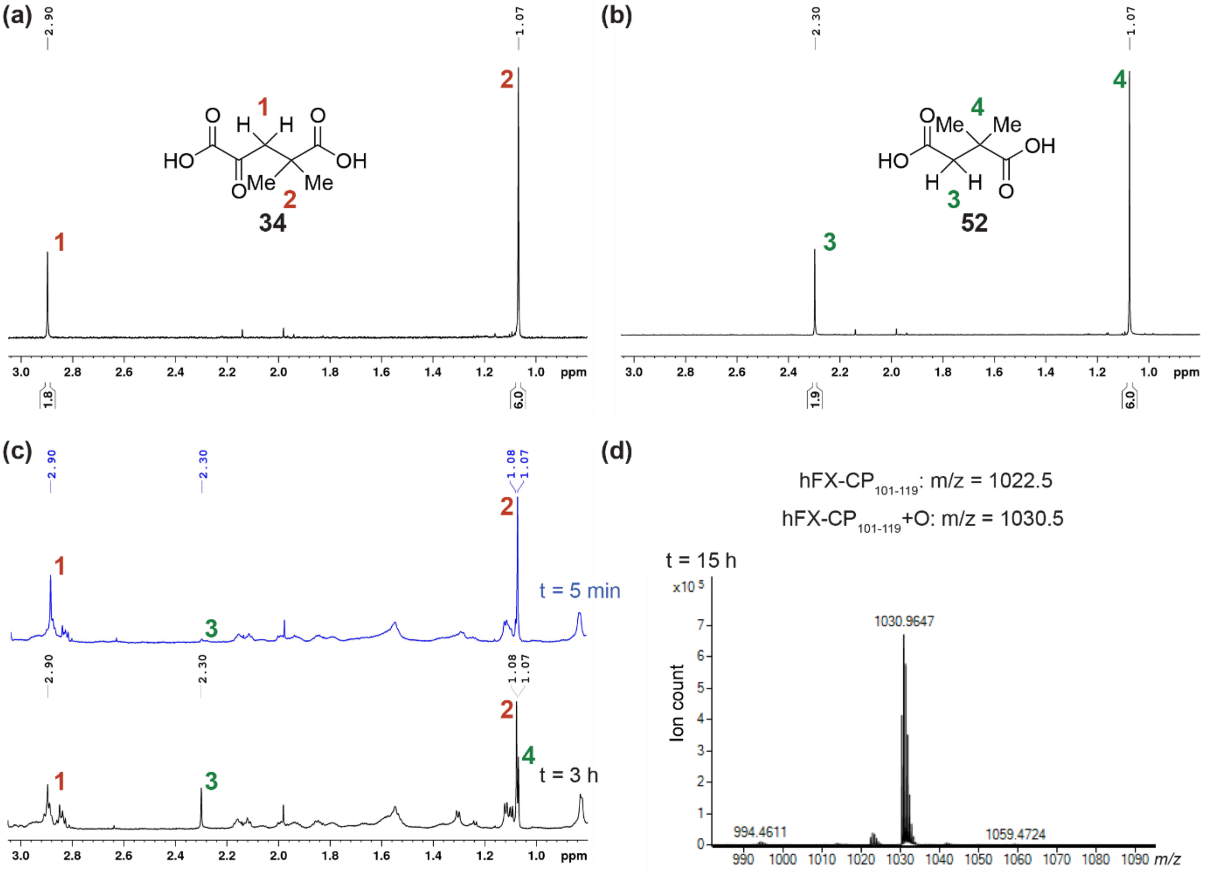

(II) Experiments using the 2OG derivative **32** as an AspH cosubstrate: (a) Close-up (3.0-2.0 ppm) of the ^{1}H NMR spectrum of synthetic **32** in 50 mM aqueous Tris-d_{11} containing 10%$_{v/v}$ D_2O (pH 7.5, 25° C); (b) Close-up (3.0-2.0 ppm) of the ^{1}H NMR spectrum of commercial 2-benzylsuccinate (**53**) in 50 mM aqueous Tris-d_{11} containing 10%$_{v/v}$ D_2O (pH 7.5, 25° C); (c) a timecourse NMR experiment (t = 5 min, top blue spectrum; t = 3 h, bottom black spectrum) reveals that AspH does not convert the 2OG derivative **32** into 2-benzylsuccinate (**53**) under the assay conditions as no new peaks are observed around 2.4 ppm; (d) Analysis of the final reaction mixture (t = 15 h) by SPE-MS reveals negligible amounts of AspH-catalyzed hydroxylation (<5% conversion) of the hFX-CP$_{101-119}$ peptide (m/z = 1022.5 corresponds to the +2 charge state of hFX-CP$_{101-119}$; m/z = 1030.5 corresponds to the +2 charge state of hydroxylated hFX-CP$_{101-119}$). Note the ^{1}H NMR signals of **32** are broad under the assay conditions, possible due to protein binding.

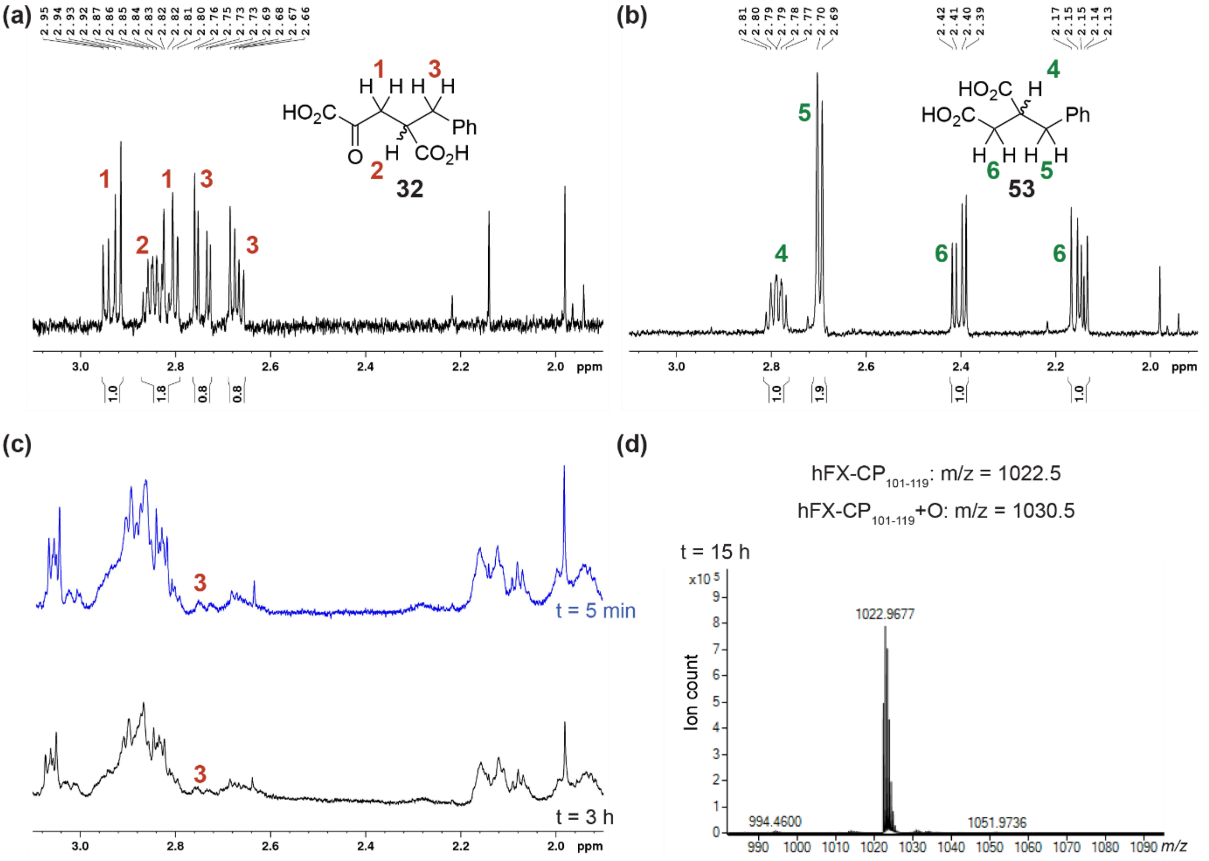

Supporting Figure S21. Proposed outline catalytic cycle for the AspH-catalyzed hydroxylation of Asp- and Asn-residues. Based on both the AspH:2OG and AspH:2OG:hFX-EGFD1$_{86\text{-}124}$-4Ser structures (Supporting Figures S7 and S8) and mechanistic studies with other 2OG oxygenases,[15] an outline catalytic cycle for AspH is proposed. Initially, 2OG coordinates to the AspH Fe(II) cofactor in a bidentate manner through one of its C1-carboxylate oxygen atoms and its C2-ketone oxygen atom (**II**), replacing two ligating water molecules in the Fe(II) complex **I**. The 2OG C1-carboxylate oxygen atom coordinates Fe(II) *trans* to His679 (**II**; Supporting Figure S7). The EGFD substrate then binds AspH with the Asp/Asn-residue undergoing β-hydroxylation displacing the water molecule binding Fe(II) *trans* to His725 (**III**), as observed in the AspH:2OG:hFX-EGFD1$_{86\text{-}124}$-4Ser structure (Supporting Figure S8). Interactions of the Asp/Asn side chain carboxylate with the active site Fe(II) are likely weak, as several different conformations for the Asp/Asn side chain carboxylate are observed in the crystalline state (Supporting Figures S8 and S14).[1] Molecular O_2 binds Fe(II) *trans* to His725 replacing the substrate side chain carboxylate to give the superoxo-Fe(III) complex **IV**. Irreversible oxidative decarboxylation of 2OG occurs, potentially via a peroxyester intermediate,[15b] to liberate CO_2 and form the Fe(IV)-oxo:succinate:substrate complex **V** (*i.e.* the ferryl intermediate). The *pro-R* hydrogen of the substrate EGFD Asn/Asp-side chain is proximate to the Fe(IV)-oxo moiety of complex **V** and is hydroxylated, via direct insertion or a radical mechanism (proceeding via an Fe(III) intermediate), to afford the Fe(II):succinate:hydroxylated substrate complex **VI**. Finally, three water molecules replace the oxidized EGFD substrate and succinate to reform complex **I**. Note, other mechanistic scenarios are feasible and detailed mechanistic studies have to be performed to define the precise AspH catalytic cycle. Note that the role of the water-occupied coordination site *trans* to the 2OG ketone oxygen is not addressed in this mechanistic proposal; it is likely, this is important as it distinguishes AspH from other 2OG oxygenases.[15]

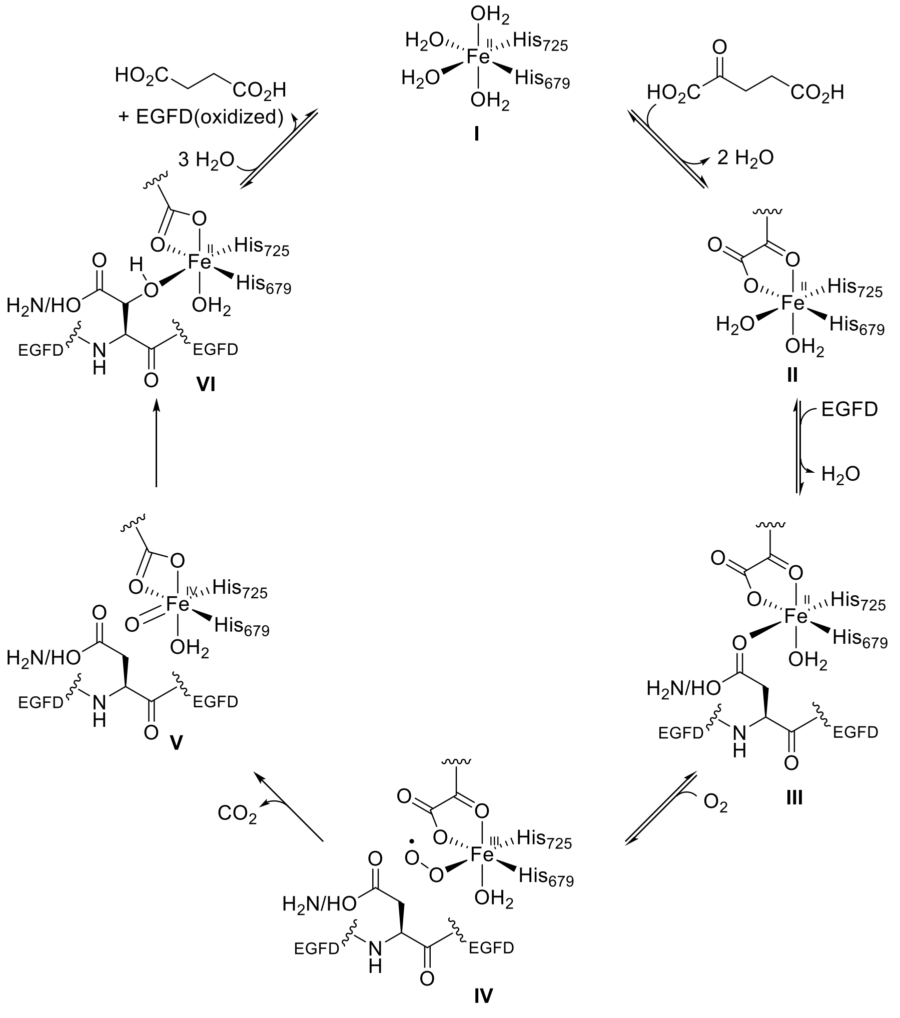

2. Supporting tables

Supporting Table S1. Evidence that the 2OG derivatives bind to AspH by competing with 2OG for binding the active site. AspH inhibition assays were performed as described in Section 8 of the Supporting Information using 50 nM His$_6$-AspH$_{315-758}$ and 1.0 µM hFX-CP$_{101-119}$ (Supporting Figure S1d). The AspH IC$_{50}$-values for the synthetic 2OG derivatives **17**, **29**, **33**, and **34** depend on the 2OG concentration, suggesting that these inhibitors/substrates (Supporting Figure S20) compete with 2OG for binding the AspH active site.

Entry	AspH inhibitor	[a,b] **IC$_{50}$** [µM]	[a,c] **IC$_{50}$** [µM]	[a,d] **IC$_{50}$** [µM]	[a,e] **IC$_{50}$** [µM]
**1**	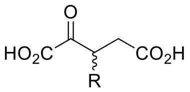 **17**: R = CH_2CH_3	1.2 ± 0.5	22.4 ± 3.2	35.3 ± 9.9	54.9 ± 10.8
**2**	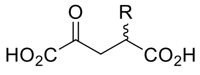 **29**: R = $CH_2CH(CH_3)_2$	0.51 ± 0.12	10.8 ± 2.7	16.8 ± 2.5	26.9 ± 7.0
**3**	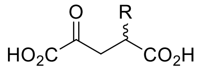 **33**: R = CH_2(2-naphthyl)	0.17 ± 0.03	2.5 ± 0.4	3.1 ± 0.3	5.6 ± 1.2
**4**	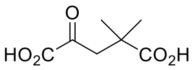 **34**	0.31 ± 0.10	6.4 ± 0.4	10.0 ± 3.1	18.7 ± 0.8

a) Mean of three independent runs (n = 3; mean ± SD); b) at an assay concentration of 3 µM 2OG; c) at an assay concentration of 200 µM 2OG; d) at an assay concentration of 400 µM 2OG; e) at an assay concentration of 600 µM 2OG. The AspH inhibition assays were of good quality which high S/N ratios and Z'-factors[14] (>0.5 for each plate) indicate (Supporting Figure S3).

Supporting Table S2. Relative efficiencies of the synthetic 2OG derivatives in substituting for 2OG as a cosubstrate in AspH-catalyzed hydroxylation reactions of hFX-CP$_{101-119}$ (continues on the following page). AspH assays were performed as described in Section 9 of the Supporting Information using 0.1 µM AspH, 2 µM hFX-CP$_{101-119}$, 50 µM FAS, 330 µM 2OG or 2OG derivative in 50 mM HEPES (pH 7.5, 20° C). 2OG was used as a positive control in the SPE-MS AspH turnover assays (Entry 1). Note that 2OG derivatives **16** and **41** were able to efficiently act as an AspH cosubstrate, *i.e.* can substitute for 2OG (Entries 2 and 27). Reduced (relative to 2OG) substrate turnover was observed when 2OG derivatives **26**, **38**, **42**, **43**, **44**, **45**, **46**, and **47** replaced 2OG (Entries 12, 24, and 28-33, respectively). None of the other 2OG derivatives functioned as cosubstrates within experimental error for the reaction time and reaction conditions investigated.

Entry	[a]2OG derivative	[b]AspH-catalyzed substrate conversion	Entry	[a]2OG derivative	[b]AspH-catalyzed substrate conversion
**1**	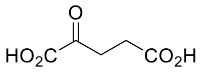<br>**2OG**	>95%	**18**	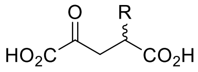<br>**32**: R = CH_2Ph	<1%
**2**	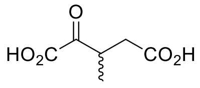<br>**16**	>95%	**19**	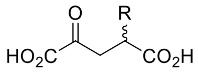<br>**33**: R = CH_2(2-naphthyl)	<1%
**3**	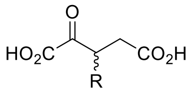<br>**17**: R = CH_2CH_3	<1%	**20**	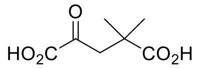<br>**34**	<1%
**4**	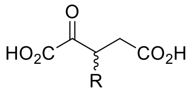<br>**18**: R = $CH_2CH_2CH_3$	<1%	[c]**21**	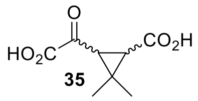<br>**35**	<1%
**5**	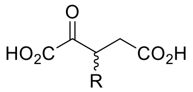<br>**19**: R = $CH_2CH_2C(CH_3)_3$	<1%	[d]**22**	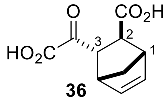<br>**36**	<1%
**6**	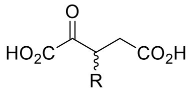<br>**20**: R = $CH_2CH_2CH_2Ph$	<1%	**23**	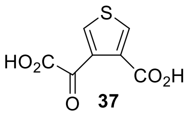<br>**37**	<1%
**7**	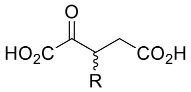<br>**21**: R = CH_2Ph	<1%	**24**	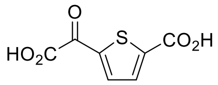<br>**38**	~15%
**8**	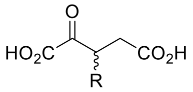<br>**22**: R = $CH_2(4\text{-}FC_6H_4)$	<1%	**25**	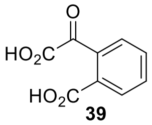<br>**39**	<1%
**9**	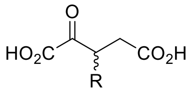<br>**23**: R = $CH_2(4\text{-}F_3COC_6H_4)$	<1%	**26**	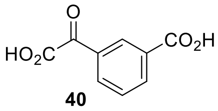<br>**40**	<1%

**10**	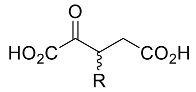 **24**: R = $CH_2(4\text{-}MeOC_6H_4)$	<1%	**27**	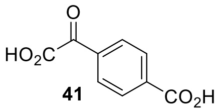 **41**	**~80%**
**11**	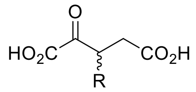 **25**: R = $CH_2(3,5\text{-}Me_2C_6H_4)$	<1%	**28**	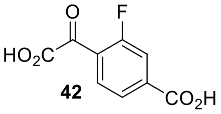 **42**	**~45%**
**12**	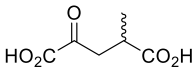 **26**	**~10%**	**29**	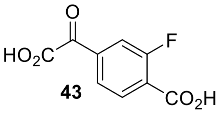 **43**	**~8%**
**13**	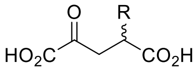 **27**: R = CH_2CH_3	<1%	**30**	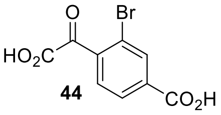 **44**	**~20%**
**14**	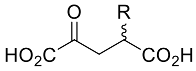 **28**: R = $CH_2CH_2CH_3$	<1%	**31**	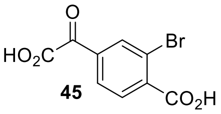 **45**	**~8%**
**15**	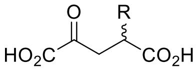 **29**: R = $CH_2CH(CH_3)_2$	<1%	**32**	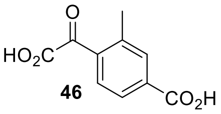 **46**	**~5%**
**16**	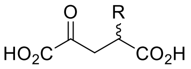 **30**: R = $CH_2CH_2C(CH_3)_3$	<1%	**33**	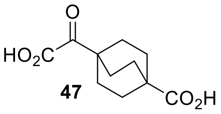 **47**	**~8%**
**17**	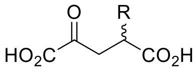 **31**: R = $CH_2CH_2CH_2Ph$	<1%	**34**	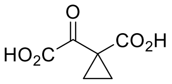 **48**	<1%

a) All chiral 2OG derivatives were prepared as racemic mixtures – as indicated by the crystallographic analyses (Supporting Figures S11, S12, and S14), it is likely that there is stereoselectivity with respect to the ability of enantiomers to inhibit AspH or act as a cosubstrate; b) determined by using SPE-MS monitoring the time-dependent conversion of the AspH substrate hFX-$CP_{101\text{-}119}$ (Supporting Figure S1d). %-Substrate conversion is shown for t = 15 min; 2OG was used as a positive control for all experiments. AspH assays were performed as described in Section 9 of the Supporting Information using 0.1 µM AspH, 2 µM hFX-$CP_{101\text{-}119}$, 50 µM FAS, 330 µM 2OG or 2OG derivative in 50 mM HEPES (pH 7.5, 20º C); c) mixture of racemic diastereomers, dr (*cis*:*trans*) = 2.5:1; d) (±)-(*2-exo,3-endo*)-diastereomer.

Supporting Table S3. Crystallization conditions, data collection, and refinement statistics for the AspH:2OG complexes.[a)]

	$AspH_{315\text{-}758}\cdot Mn^{II}\cdot$ 2OG (AspH:2OG)	$AspH_{315\text{-}758}\cdot Mn^{II}\cdot$2OG·hFX-EGFD$1_{86\text{-}124}$-4Ser (AspH:2OG:hFX-EGFD$1_{86\text{-}124}$-4Ser)
**PDB ID**	6YYU	6YYW
**Crystallization**		
Precipitation conditions	18 mg/mL His_6-$AspH_{315\text{-}758}$, 1 mM $MnCl_2$, 2 mM 2OG, 100 mM HEPES, pH 7.5, 20%$_{w/v}$ PEG4000, 10%$_{v/v}$ 2-propanol	18 mg/mL His_6-$AspH_{315\text{-}758}$, 1 mM $MnCl_2$, 2 mM 2OG, 100 mM Bis Tris propane, pH 7.5, 200 mM sodium acetate trihydrate, 20%$_{w/v}$ PEG3350, 726 μM hFX-EGFD$1_{86\text{-}124}$-4Ser
**Data collection**		
Space group	$P2_12_12_1$	$P2_12_12_1$
Symmetry	orthorhombic	orthorhombic
Cell dimensions:		
a, *b*, *c* (Å)	48.79, 70.00, 170.87	50.44, 90.95, 123.61
α, β, γ (°)	90.00, 90.00, 90.00	90.00, 90.00, 90.00
X-Ray source[b)]	Synchrotron (DLS I03)	Synchrotron (DLS I04)
Resolution (Å)[c)]	85.44-2.11 (2.42-2.11)	29.64-2.27 (2.34-2.27)
R_{merge}	0.185 (1.746)	0.125 (0.863)
$I / \sigma I$	11.0 (1.6)	12.0 (1.8)
CC (1/2)	0.997 (0.509)	0.998 (0.807)
Total number of reflections	269481 (11362)	332506 (13376)
Total number unique reflections	20459 (1024)	26364 (1700)
Completeness (%)[d)]	90.8 (56.2)	97.2 (69.9)
Multiplicity	13.2 (11.1)	12.6 (7.9)
**Refinement**		
R_{work} / R_{free}	0.202 / 0.231	0.187 / 0.225
No. atoms:	6566	7197
B-factors:	64.8	56.9
R.m.s. deviations:		
Bond lengths (Å)	0.017	0.014
Bond angles (°)	1.619	1.442

[a)]Experimental details are described in Section 10 of the Supporting Information; [b)]DLS: Diamond Light Source; [c)]Values in parentheses are for highest-resolution shell; [d)]Ellipsoidal completeness, as defined by autoPROC/STARANISO (Supporting Information, Section 10).

Supporting Table S4. Crystallization conditions, data collection, and refinement statistics for the AspH:2OG derivative complexes.[a)]

	$AspH_{315-758}·Mn^{II}·$(*R*)-**16** (AspH:**16**)	$AspH_{315-758}·Mn^{II}·$(*R*)-**16**·hFX-$EGFD1_{86-124}$-4Ser (AspH:**16**:hFX-$EGFD1_{86-124}$-4Ser)	$AspH_{315-758}·Mn^{II}·$(*R*)-**17**·hFX-$EGFD1_{86-124}$-4Ser (AspH:**17**:hFX-$EGFD1_{86-124}$-4Ser)	$AspH_{315-758}·Mn^{II}·$**34**·hFX-$EGFD1_{86-124}$-4Ser (AspH:**34**:hFX-$EGFD1_{86-124}$-4Ser)	$AspH_{315-758}·Mn^{II}·$**49**·hFX-$EGFD1_{86-124}$-4Ser (AspH:**49**:hFX-$EGFD1_{86-124}$-4Ser)
**PDB ID**	6YYV	6YYX	6Z6Q	6YYY	6Z6R
**Crystallization**					
Precipitation conditions	18 mg/mL His_6-$AspH_{315-758}$, 1 mM $MnCl_2$, 2 mM **16**, 200 mM magnesium acetate tetrahydrate, pH 7.9, 20%$_{w/v}$ PEG3350	18 mg/mL His_6-$AspH_{315-758}$, 1 mM $MnCl_2$, 2 mM **16**, 200 mM Bis Tris propane, pH 6.5, 200 mM sodium/potassium phosphate, 20%$_{w/v}$ PEG3350, 726 µM hFX-$EGFD1_{86-124}$-4Ser	18 mg/mL His_6-$AspH_{315-758}$, 1 mM $MnCl_2$, 2 mM **17**, 100 mM HEPES, pH 7.0, 200 mM ammonium chloride, 20%$_{w/v}$ PEG6000, 726 µM hFX-$EGFD1_{86-124}$-4Ser	18 mg/mL His_6-$AspH_{315-758}$, 1 mM $MnCl_2$, 2 mM **34**, 200 mM sodium bromide, 20%$_{w/v}$ PEG3350, 726 µM hFX-$EGFD1_{86-124}$-4Ser	18 mg/mL His_6-$AspH_{315-758}$, 1 mM $MnCl_2$, 2 mM **49**, 100 mM Bis Tris propane, pH 6.5, 200 mM sodium bromide, 20%$_{w/v}$ PEG3350, 726 µM hFX-$EGFD1_{86-124}$-4Ser
**Data collection**					
Space group	$P2_12_12_1$	$P2_12_12_1$	$P2_12_12_1$	$P2_12_12_1$	$P2_12_12_1$
Symmetry	orthorhombic	orthorhombic	orthorhombic	orthorhombic	orthorhombic
Cell dimensions:					
a, *b*, *c* (Å)	48.80, 70.52, 174.49	50.22, 86.59, 123.27	49.6, 91.3, 122.6	125.82, 50.32, 86.60	49.9, 90.6, 122.4
α, β, γ (°)	90.00, 90.00, 90.00	90.00, 90.00, 90.00	90.00, 90.00, 90.00	90.00, 90.00, 90.00	90.00, 90.00, 90.00
X-Ray source[b)]	Synchrotron (DLS I03)	Synchrotron (DLS I04)	Synchrotron (DLS I03)	Synchrotron (DLS I04)	Synchrotron (DLS I03)
Resolution (Å)[c)]	87.24-1.77 (1.96-1.77)	86.59-1.53 (1.71-1.53)	46.00-1.81 (1.84-1.81)	86.60-2.29 (2.48-2.29)	50.70-2.14 (2.29-2.14)
R_{merge}	0.110 (1.876)	0.068 (1.289)	0.180 (5.809)	1.129 (3.167)	0.175 (2.090)
$I / \sigma I$	16.1 (1.5)	16.6 (1.4)	6.8 (0.1)	4.7 (1.3)	11.1 (1.3)
CC (1/2)	0.999 (0.540)	0.999 (0.665)	0.994 (0.296)	0.816 (0.348)	0.998 (0.493)
Total number of reflections	575914 (26436)	537727 (22432)	672788 (31198)	195505 (11034)	277115 (13568)
Total number unique reflections	43395 (2170)	48664 (2433)	51780 (2579)	15358 (769)	21003 (1051)
Completeness (%)	94.2 (59.6)[d)]	88.7 (51.4)[d)]	100.0 (100.0)	89.6 (77.1)[d)]	67.2 (19.2)[d)]
Multiplicity	13.3 (12.2)	11.0 (9.2)	13.0 (12.1)	12.7 (14.3)	13.2 (12.9)
**Refinement**					
R_{work} / R_{free}	0.173 / 0.208	0.162 / 0.192	0.208 / 0.232	0.209 / 0.244	0.212 / 0.242
No. atoms:	7142	7527	7422	6889	7156
B-factors:	52.7	31.6	54.0	42.9	45.6
R.m.s. deviations:					
Bond lengths (Å)	0.012	0.012	0.011	0.018	0.014
Bond angles (°)	1.150	1.281	0.997	1.594	1.615

[a)]Experimental details are described in Section 10 of the Supporting Information; [b)]DLS: Diamond Light Source; [c)]Values in parentheses are for highest-resolution shell; [d)]Ellipsoidal completeness, as defined by autoPROC/STARANISO (Supporting Information, Section 10).

3. General information

Unless otherwise stated, all reagents were from commercial sources (Sigma-Aldrich, Inc.; Fluorochem Ltd; Tokyo Chemical Industry Ltd.; Alfa Aesar; Manchester Organics) and used as received. To oxidize cyanosulfur ylids, oxone obtained from Alfa Aesar was used according to a literature protocol.[16] Compounds **14**,[16] **S3**,[8] and **S7**[11] were synthesized according to the cited literature. All chiral compounds were prepared as racemates. Anhydrous solvents were from Sigma-Aldrich, Inc. and kept under an atmosphere of nitrogen. Solvents, liquids, and solutions were transferred using nitrogen-flushed stainless steel needles and syringes. All reactions were carried out under an atmosphere of nitrogen unless stated otherwise. Milli-Q® Ultrapure (MQ-grade) water was used for buffers; LCMS grade solvents (Merck) were used for solid phase extraction coupled to mass spectrometry (SPE-MS).
Purifications were performed using an automated Biotage Isolera One purification machine (wavelengths monitored: 254 and 280 nm) equipped with pre-packed Biotage® SNAP KP-Sil or Biotage® SNAP Ultra flash chromatography cartridges. The cartridge size and solvent gradients (in column volumes, CV) used are specified in the individual experimental procedures. HPLC grade solvents (acetone, cyclohexane, dichloromethane, ethyl acetate, methanol; Sigma-Aldrich, Inc.) were used for purifications, reaction work-ups, and extractions.
Thin layer chromatography (TLC) was carried out using Merck silica gel 60 F_{254} TLC plates and visualized under UV light and by using appropriate staining solutions (*e.g.* Hanessian's stain). Melting points (m.p.) were determined using a Stuart SMP-40 automated melting point apparatus. Infrared (IR) spectroscopy was performed using a Bruker Tensor-27 Fourier transform infrared (FT-IR) spectrometer. High-resolution mass spectrometry (HRMS) was performed using electro-spray ionization (ESI) mass spectrometry (MS) in the positive or negative ionization modes employing a Thermo Scientific Exactive mass spectrometer (ThermoFisher Scientific); data are presented as a mass-to-charge ratio (*m/z*).
Nuclear magnetic resonance (NMR) spectroscopy was performed using a Bruker AVANCE AVIIIHD 600 machine equipped with a 5mm BB-F/1H Prodigy N_2 cryoprobe. Chemical shifts for protons are reported in parts per million (ppm) downfield from tetramethylsilane and are referenced to residual protium in the NMR solvent ($CDCl_3$: δ = 7.28 ppm; CD_3OD: δ = 3.31 ppm; DMSO-d_6: δ = 2.49 ppm; D_2O: δ = 4.79 ppm). For ^{13}C NMR, chemical shifts are reported in the scale relative to the NMR solvent (*i.e.* $CDCl_3$: δ = 77.00 ppm; CD_3OD: δ = 49.00 ppm; DMSO-d_6: δ = 39.52 ppm). For ^{19}F NMR, chemical shifts are reported in the scale relative to $CFCl_3$ and for ^{31}P NMR, chemical shifts are reported in the scale relative to 85% H_3PO_4. NMR data are reported as follows: chemical shift, multiplicity (s: singlet, d: doublet, dd: doublet of doublets, t: triplet, q: quartet, m: multiplet, br: broad signal), coupling constant (*J* in Hz; accurate to 0.1 Hz), and integration. The number of C-atoms in brackets indicates overlapping signals in ^{13}C NMR; chemical shift numbers in brackets indicate close signals that can be differentiated taking into account second respectively third decimal numbers.

4. General synthetic procedures

4.1 General Procedure A

To a solution of commercial anhydride **S1a** or **b** (1.0 equiv.) in methanol (1.0 M, HPLC grade) was added sodium methoxide solution (25%$_{w/w}$ in methanol, 1.5 equiv.) under an ambient atmosphere at 0° C. The reaction mixture was slowly warmed to ambient temperature overnight (10 – 12 h), before methanol was removed under reduced pressure. The residue was diluted with water, then extracted three times with dichloromethane (the organic extracts were discarded). The aqueous phase was acidified (pH < 2) by adding aqueous HCl (1.0 M), then extracted three times with ethyl acetate. The combined organic extracts were dried over anhydrous Na_2SO_4, filtered, and evaporated to afford the desired crude mono-methyl dicarboxylic acid half-esters **13a** or **b**, which were used without further purification following General Procedure F.

4.2 General Procedure B

A modified literature procedure was followed:[7] To palladium acetate (0.03 equiv.) and Xantphos[17] (0.03 equiv.) in a pressure tube were sequentially added anhydrous *N*,*N*-dimethylformamide (0.75 M), an aryl iodide (**S1c-g**; 1.0 equiv.), *N*,*N*-dicyclohexylcarbodiimide (DCC, 2.0 equiv), trimethylamine (2.0 equiv.), and formic acid (3.0 equiv.) under ambient atmosphere at ambient temperature. The pressure tube was sealed immediately after the addition of formic acid. *Caution*: Upon addition of formic acid to the reaction mixture, vigorous gas evolution occurs! The pressure tube was placed into a preheated sand bath (60° C), and the reaction mixture stirred overnight (12 – 14 h). *Caution*: The reaction was performed behind an additional safety shield as the internal pressure might cause an explosion! The pressure tube was cooled to ambient temperature and carefully opened taking appropriate safety measurements. The reaction mixture was carefully diluted with saturated aqueous $NaHCO_3$ solution, then extracted three times with ethyl acetate (the organic extracts were discarded). The aqueous phase was acidified (pH < 2) by adding concentrated HCl, then extracted three times with ethyl acetate. The combined organic extracts were dried over anhydrous Na_2SO_4, filtered, and evaporated to afford the desired crude mono-methyl dicarboxylic acid half-ester (**13c-g**), which was used without further purification following General Procedure F.

4.3 General Procedure C

A modified literature procedure was followed:[9] A solution of 1-benzyl-4-methyl itaconate **S3**[8] (1.0 equiv.), aryl iodide **S4h-l** (1.1 equiv.), and *N*,*N*-dicyclohexylmethylamine (1.5 equiv.) in anhydrous *N*,*N*-dimethylacetamide (1.5 M) was added into a sealed 20 mL microwave vial (Biotage) containing palladium acetate (0.02 equiv.) and tetraethylammonium chloride (1.0 equiv.). N_2 gas was bubbled through the reaction mixture for 15 min. The microwave vial was then placed into a preheated sand bath (130° C) and the reaction mixture stirred overnight (12 – 16 h). The reaction mixture was then cooled to ambient temperature and evaporated under reduced pressure. The residue was diluted with saturated aqueous NH_4Cl solution, then extracted three times with ethyl acetate. The combined organic extracts were dried over anhydrous Na_2SO_4, filtered, evaporated, and purified using column chromatography to afford the desired substituted 1-benzyl-4-methylsuccinate as a mixture of diastereomers (typically > 10:1, *E*:*Z*) which was used in the next reaction without further purification following General Procedure E.

4.4 General Procedure D

To a solution of phosphonate **S5** or **S7**[11] (1.0 equiv.) in anhydrous THF (0.24 M) was added dropwise *n*-butyl lithium (2.5 M in hexanes, 1.1 equiv.) at -78° C under an atmosphere of N_2 gas. The reaction mixture was stirred for 30 min at the same temperature; the neat aldehyde **S6** or **S8** (1.1 equiv.) was then added dropwise. The reaction mixture was then slowly warmed to ambient temperature overnight (10 – 12 h), before saturated aqueous NH_4Cl solution was added and the mixture extracted three times with ethyl acetate. The combined organic extracts were dried over anhydrous Na_2SO_4, filtered, evaporated, and purified using column chromatography to afford the desired substituted 1-benzyl-4-methylsuccinate as a mixture of alkene diastereomers (typically >1.3:1) which was used in the next reaction without further purification following General Procedure E.

4.5 General Procedure E

The substituted 1-benzyl-4-methylsuccinate (1.0 equiv.) was dissolved in anhydrous methanol (0.4 M). N_2 gas was bubbled through the solution for 15 min before palladium on charcoal (10%$_{w/w}$ palladium) was added. H_2 gas was bubbled through the black suspension for 15 min and the reaction mixture was stirred overnight (12 – 14 h)

at ambient temperature under an atmosphere of H_2 (1 atm). The resultant black suspension was filtered through Celite®, washed with methanol (*Caution*: Palladium-containing waste was kept as aqueous suspension before appropriate disposal), and evaporated to afford the desired crude mono-methyl dicarboxylic acid half-ester (**13h-w**), which was used in the next reaction without further purification following General Procedure F.

4.6 General Procedure F

To a solution of mono-methyl dicarboxylic acid half-ester **13** (1.0 equiv.) and 1-(cyanomethyl)tetrahydro-1*H*-thiophen-1-ium bromide **14**[16] (1.4 equiv.) in anhydrous dichloromethane (0.2 M) were sequentially added redistilled *N*,*N*-diisopropylethylamine (4.0 equiv.) and 1-propanephosphonic anhydride (T3P, $50\%_{w/w}$ in ethyl acetate, 1.4 equiv.) dropwise at 0° C under an atmosphere of N_2 gas. The reaction mixture was stirred and allowed to slowly warm to ambient temperature overnight (10 – 12 h). The reaction mixture was diluted with saturated aqueous $NaHCO_3$ solution and extracted three times with dichloromethane. The combined organic extracts were dried over anhydrous Na_2SO_4, filtered, and evaporated. The crude residue was purified using column chromatography to afford cyanosulfur ylid **15** which was used in the next reaction following General Procedure G.

4.7 General Procedure G

To a solution of the cyanosulfur ylid **15** (1.0 equiv.) in a 2:1 mixture (0.08 M final concentration) of methanol (HPLC grade) and Milli-Q® Ultrapure water was added Oxone (2.0 equiv., obtained from Alfa Aesar as recommended by Bode *et al.*[16]) under an ambient atmosphere at 0° C. The reaction mixture was stirred vigorously at the same temperature for 5 min, then at ambient temperature for 1 h. It was then filtered and the methanol removed under reduced pressure. The remaining aqueous solution was carefully diluted with saturated aqueous $NaHCO_3$ solution and the mixture extracted three times with dichloromethane; the combined organic extracts were dried over anhydrous Na_2SO_4, filtered, and evaporated. The crude residue was purified using column chromatography to afford dimethyl dicarboxylic acid ester **12** which was used in the next reaction following General Procedure H.

4.8 General Procedure H

To a solution of dimethyl dicarboxylic acid ester **12** (1.0 equiv.) in methanol (0.2 M, HPLC grade) was added an aqueous solution of lithium hydroxide (0.4 M, 2.8 equiv.) under an ambient atmosphere at 0° C. The reaction mixture was allowed to slowly warm to ambient temperature overnight (14 – 18 h). The methanol was then removed under reduced pressure and the remaining aqueous reaction mixture was extracted three times with dichloromethane (the organic extracts were discarded). The aqueous phase was acidified (pH ≈7.0 to 7.7) using Dowex® 50XW8 (H^+-form, mesh 200–400), filtered, and lyophilized to afford the solid dicarboxylic acid **6**. The crude product was sufficiently pure as judged by ^1H and ^{13}C NMR and used without further purification in the biological assays. pKa-values for the 2OG derivatives were not determined, thus, some might have actually been isolated as the corresponding mono- or dilithium salts.

The chemical shift data of the 2OG derivatives are likely pH-dependent. In the NMR spectra of some 2OG derivatives, minor amounts of the enol-tautomers, keto-hydrates or pseudo-acids were observed. Peak-broadening was observed in the ^{13}C NMR of some 2OG derivatives.

5. Synthetic procedures and analytical data

1-Benzyl 4-methyl 2-(dimethoxyphosphoryl)succinate (S5).

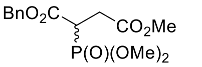

To a suspension of sodium hydride (1.86 g of a 60%$_{w/w}$ dispersion in mineral oil, 46.4 mmol, 1.0 equiv.) in anhydrous THF (120 mL) was added dropwise benzyl 2-(dimethoxyphosphoryl)acetate (12.0 g, 46.4 mmol, 1.0 equiv.) at 0° C. The reaction mixture was stirred for 30 min at the same temperature. Methyl 2-bromoacetate (4.8 mL, 51.0 mmol, 1.1 equiv.) was added dropwise to the reaction mixture, which was then stirred at 0° C for 3 h before being poured onto ice. Saturated aqueous NH_4Cl solution was added and the mixture was extracted three times with ethyl acetate. The combined organic extracts were washed with brine, dried over anhydrous Na_2SO_4, filtered, and evaporated to afford 11.0 g the pure phosphonate **S5** (72%), following column chromatography (100 g Ultra; 100 mL/min; 100% dichloromethane (2 CV), followed by a linear gradient (10 CV): 0%→50% ethyl acetate in dichloromethane).

Clear pale yellow oil; ^{1}H NMR (600 MHz, 300 K, $CDCl_3$): δ = 7.40−7.35 (m, 4H), 7.34−7.31 (m, 1H), 5.28 (d, J = 12.3 Hz, 1H), 5.17 (d, J = 12.3 Hz, 1H), 3.74 (d, J = 11.1 Hz, 3H), 3.72 (d, J = 11.1 Hz, 3H), 3.66 (s, 3H), 3.56 (ddd, J = 24.3, 11.2, 3.6 Hz, 1H), 3.10 (ddd, J = 17.5, 11.2, 7.7 Hz, 1H), 2.84 ppm (ddd, J = 17.5, 9.5, 3.6 Hz, 1H); ^{13}C NMR (150 MHz, 300 K, $CDCl_3$): δ = 171.3 (d, J = 19.1 Hz), 167.8 (d, J = 5.5 Hz), 135.2, 128.4, 128.3, 128.2(5), 67.5, 53.4 (d, J = 4.3 Hz), 53.3(7) (d, J = 4.0 Hz), 52.1, 40.7 (d, J = 132.2 Hz), 31.1 ppm (d, J = 2.4 Hz); ^{31}P NMR (243 MHz, 300 K, $CDCl_3$): δ = 23.6 ppm (m, 1P); IR (film): ṽ = 3032, 3010, 2956, 2853, 1736, 1499, 1456, 1439, 1366, 1322, 1260, 1216, 1158, 1051, 1029 cm^{-1}; HRMS (ESI): m/z calculated for $C_{14}H_{19}O_7PNa$ $[M+Na]^+$: 353.0761, found: 353.0759.

Methyl 5-cyano-3-methyl-4-oxo-5-(tetrahydro-1-λ^4-thiophen-1-ylidene)pentanoate (S9).

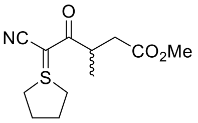

According to General Procedure F, cyanosulfur ylid **S9** (2.98 g, 69%) was obtained from 4-methoxy-2-methyl-4-oxobutanoic acid[18] (2.46 g, 16.8 mmol), following column chromatography (100 g KP-Sil; 50 mL/min; 100% ethyl acetate (7 CV), followed by a linear gradient (7 CV): 0%→100% acetone in ethyl acetate).

Clear orange oil; ^{1}H NMR (600 MHz, 300 K, $CDCl_3$): δ = 3.66 (s, 3H), 3.40−3.35 (m, 4H), 3.35−3.30 (m, 1H), 2.77 (dd, J = 16.2, 8.7 Hz, 1H), 2.66−2.59 (m, 2H), 2.34 (dd, J = 16.2, 5.7 Hz, 1H), 2.11−2.03 (m, 2H), 1.18 ppm (d, J = 7.0 Hz, 3H); ^{13}C NMR (150 MHz, 300 K, $CDCl_3$): δ = 194.1, 172.7, 120.5, 53.1, 51.5, 45.0, 44.4, 38.2, 37.5, 28.5, 28.4(5), 18.0 ppm; IR (film): ṽ = 3458, 2954, 2878, 2168, 1731, 1576, 1459, 1439, 1421, 1377, 1351, 1300, 1274, 1198, 1178, 1156, 1134, 1086, 1070, 1006 cm^{-1}; HRMS (ESI): m/z calculated for $C_{12}H_{17}O_3NSNa$ $[M+Na]^+$: 278.0821, found: 278.0821.

Dimethyl 3-methyl-2-oxopentanedioate (S10).

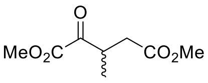

According to General Procedure G, dimethyl dicarboxylate **S10** (1.18 g, 54%) was obtained from cyanosulfur ylid **S9** (2.98 g, 11.7 mmol), following column chromatography (50 g KP-Sil; 50 mL/min; 100% cyclohexane (3 CV), followed by a linear gradient (6 CV): 0%→20% ethyl acetate in cyclohexane). The analytical data are in agreement with those reported.[19]

Clear colorless oil; ^{1}H NMR (600 MHz, 300 K, $CDCl_3$): δ = 3.91 (s, 3H), 3.73−3.66 (m, 1H), 3.68 (s, 3H), 2.77 (dd, J = 17.0, 8.9 Hz, 1H), 2.53 (dd, J = 17.0, 5.4 Hz, 1H), 1.23 ppm (d, J = 7.1 Hz, 3H); ^{13}C NMR (150 MHz, 300 K, $CDCl_3$): δ = 195.8, 172.1, 161.2, 53.0, 51.9, 38.3, 36.8, 15.9 ppm; IR (film): ṽ = 2958, 1728, 1438, 1354, 1262, 1201, 1035, 1004 cm^{-1}; HRMS (ESI): m/z calculated for $C_8H_{12}O_5Na$ $[M+Na]^+$: 211.0588, found: 211.0579.

3-Methyl-2-oxopentanedioic acid (16).

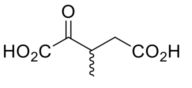

Dicarboxylic acid **16** (159 mg, quant.) was obtained from dimethyl 3-methyl-2-oxopentanedioate **S10** (188 mg, 1.0 mmol) according to General Procedure H. Minor amounts

(~3%) of a byproduct are visible in ^{1}H NMR. Signal broadening required extended measurement times to obtain a high quality ^{13}C NMR spectrum enabling the parallel detection of ^{13}C resonances for the byproduct which can be assigned as the corresponding pseudo-carboxylic acid or the keto-hydrate but likely not the enol-tautomer: ^{13}C NMR (150 MHz, 300 K, D_2O): δ = 182.4, 178.0, 97.2, 38.9, 37.8, 13.9 ppm. Analytical data are shown below for the major 2-oxocarboxylic acid; the chemical shifts in the ^{13}C NMR spectrum are in agreement with those of the reported corresponding dilithium salt.[19b]

White amorphous solid; ^{1}H NMR (600 MHz, 300 K, D_2O): δ = 3.34 (brs, 1H), 2.61 (dd, J = 15.2, 6.2 Hz, 1H), 2.25−2.21 (m, 1H), 1.16 ppm (d, J = 7.1 Hz, 3H); ^{13}C NMR (150 MHz, 300 K, D_2O): δ = 208.9 (br), 180.5, 171.3, 39.9, 39.1, 14.4 ppm; IR (film): ṽ = 3385, 2952, 2917, 2876, 1706, 1571, 1456, 1435, 1405, 1377, 1359, 1165, 1136, 1056, 1038 cm^{-1}; HRMS (ESI): *m/z* calculated for $C_6H_7O_5$ [M−H]$^{-}$: 159.0299, found: 159.0297.

Methyl 5-cyano-3-ethyl-4-oxo-5-(tetrahydro-1-λ^4-thiophen-1-ylidene)pentanoate (S11).

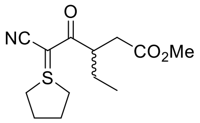

According to General Procedure D, 1-benzyl 4-methyl 2-ethylidenesuccinate (810 mg, 53%) was obtained from 1-benzyl 4-methyl 2-(dimethoxyphosphoryl)succinate **S5** (2.05 g, 6.2 mmol) and acetaldehyde, following column chromatography (50 g KP-Sil; 50 mL/min; 100% cyclohexane (3 CV), followed by a linear gradient (12 CV): 0%→15% ethyl acetate in cyclohexane). The clear colorless oil was a mixture of alkene diastereomers (1.8:1) which was used in the next reaction without further purification. ^{1}H NMR (400 MHz, 300 K, $CDCl_3$): major diastereomer: δ = 7.40−7.31 (m, 5H), 7.14 (q, J = 7.2 Hz, 1H), 5.21 (s, 2H), 3.66 (s, 3H), 3.41 (s, 2H), 1.86 ppm (d, J = 7.2 Hz, 3H); minor diastereomer: δ = 7.40−7.31 (m, 5H), 6.25 (qt, J = 7.3, 1.0 Hz, 1H), 5.22 (s, 2H), 3.60 (s, 3H), 3.30 (t, J = 1.0 Hz, 2H), 2.12 ppm (dt, J = 7.3, 0.9 Hz, 3H).

1-Benzyl 4-methyl 2-ethylidenesuccinate (810 mg, 3.26 mmol) was reduced according to General Procedure E to afford the corresponding crude mono-methyl dicarboxylic acid half-ester (quant.), which was used in the next reaction without further purification. According to General Procedure F, cyanosulfur ylid **S11** (264 mg, 30% over two steps) was obtained, following column chromatography (50 g KP-Sil; 50 mL/min; 100% ethyl acetate (7 CV), followed by a linear gradient (7 CV): 0%→100% acetone in ethyl acetate).

Clear pale yellow oil; ^{1}H NMR (600 MHz, 300 K, $CDCl_3$): δ = 3.66 (s, 3H), 3.38−3.34 (m, 4H), 3.23−3.19 (m, 1H), 2.74 (dd, J = 16.1, 9.5 Hz, 1H), 2.66−2.60 (m, 2H), 2.40 (dd, J = 16.1, 5.0 Hz, 1H), 2.11−2.04 (m, 2H), 1.71−1.64 (m, 1H), 1.56−1.49 (m, 1H), 0.95 ppm (t, J = 7.4 Hz, 3H); ^{13}C NMR (150 MHz, 300 K, $CDCl_3$): δ = 193.5, 172.8, 120.6, 54.4, 51.5, 44.9, 44.8, 44.3, 35.9, 28.5, 28.4(7), 25.9, 11.5 ppm; IR (film): ṽ = 3483, 2963, 2876, 2168, 1733, 1576, 1437, 1356, 1302, 1244, 1155, 1089 cm^{-1}; HRMS (ESI): *m/z* calculated for $C_{13}H_{19}O_3NSNa$ [M+Na]$^{+}$: 292.0978, found: 292.0978.

Dimethyl 3-ethyl-4-oxopentanedioate (S12).

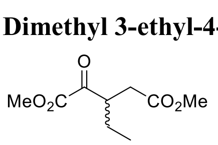

According to General Procedure G, dimethyl dicarboxylate **S12** (106 mg, 53%) was obtained from cyanosulfur ylid **S11** (264 mg, 0.98 mmol), following column chromatography (10 g Ultra; 35 mL/min; 100% cyclohexane (3 CV), followed by a linear gradient (20 CV): 0%→15% ethyl acetate in cyclohexane). The analytical data are in agreement with those reported.[19b]

Clear colorless oil; ^{1}H NMR (600 MHz, 300 K, $CDCl_3$): δ = 3.91 (s, 3H), 3.66 (s, 3H), 3.65−3.62 (m, 1H), 2.85 (dd, J = 17.1, 10.2 Hz, 1H), 2.57 (dd, J = 17.1, 4.5 Hz, 1H), 1.80−1.73 (m, 1H), 1.59−1.52 (m, 1H), 0.95 ppm (t, J = 7.5 Hz, 3H); ^{13}C NMR (150 MHz, 300 K, $CDCl_3$): δ = 196.0, 172.4, 161.4, 53.0, 52.0, 44.3, 34.9, 24.0, 11.1 ppm; IR (film): ṽ = 2959, 2881, 1728, 1438, 1357, 1251, 1202, 1179, 1138, 1053, 975 cm^{-1}; HRMS (ESI): *m/z* calculated for $C_9H_{14}O_5Na$ [M+Na]$^{+}$: 225.0733, found: 225.0735.

3-Ethyl-2-oxopentanedioic acid (17).

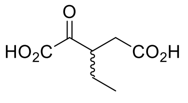

Dicarboxylic acid **17** (34 mg, 78%) was obtained from dimethyl 3-ethyl-4-oxopentanedioate **S12** (50.6 mg, 0.25 mmol) according to General Procedure H. The chemical shifts in the ^{13}C NMR spectrum are in agreement with those of the reported corresponding dilithium salt.[19b]

White amorphous solid; ^1H NMR (600 MHz, 300 K, D_2O): δ = 3.40−3.35 (m, 1H), 2.55 (dd, J = 15.3, 6.8 Hz, 1H), 2.29 (dd, J = 15.3, 7.8 Hz, 1H), 1.76−1.68 (m, 1H), 1.64−1.57 (m, 1H), 0.91 ppm (t, J = 7.5 Hz, 3H); ^{13}C NMR (150 MHz, 300 K, D_2O): δ = 208.9, 180.9, 171.2, 46.5, 36.9, 22.8, 10.4 ppm; IR (film): ṽ = 3377, 2971, 2936, 2880, 1703, 1636, 1571, 1404, 1361, 1133, 1071 cm^{-1}; HRMS (ESI): *m/z* calculated for $C_7H_9O_5$ $[M-H]^-$: 173.0445, found: 173.0452.

Methyl 3-(2-cyano-2-(tetrahydro-1-λ⁴-thiophen-1-ylidene)acetyl)hexanoate (S13).

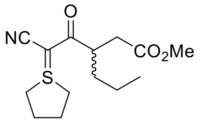

According to General Procedure D, 1-benzyl 4-methyl 2-propylidenesuccinate (821 mg, 52%) was obtained from 1-benzyl 4-methyl 2-(dimethoxyphosphoryl)succinate **S5** (1.98 g, 6.0 mmol) and propionaldehyde, following column chromatography (50 g KP-Sil; 50 mL/min; 100% cyclohexane (3 CV), followed by a linear gradient (10 CV): 0%→12% ethyl acetate in cyclohexane). The clear colorless oil was a mixture of alkene diastereomers (1.7:1) which was used in the next reaction without further purification. ^1H NMR (400 MHz, 300 K, $CDCl_3$): major diastereomer: δ = 7.41−7.31 (m, 5H), 7.03 (t, J = 7.5 Hz, 1H), 5.21 (s, 2H), 3.65 (s, 3H), 3.39 (s, 2H), 2.23 (apparent quint., J = 7.5 Hz, 2H), 1.09 ppm (t, J = 7.5 Hz, 3H); minor diastereomer: δ = 7.41−7.31 (m, 5H), 6.10 (tt, J = 7.3, 1.0 Hz, 1H), 5.21 (s, 2H), 3.60 (s, 3H), 3.30−3.29 (m, 2H), 2.61 (apparent quint., J = 7.5 Hz, 2H), 1.06 ppm (t, J = 7.6 Hz, 3H).

1-Benzyl 4-methyl 2-propylidenesuccinate (821 mg, 3.13 mmol) was reduced according to General Procedure E to afford the corresponding crude mono-methyl dicarboxylic acid half-ester (quant.), which was used in the next reaction without further purification. According to General Procedure F, cyanosulfur ylid **S13** (270 mg, 30% over two steps) was obtained, following column chromatography (50 g KP-Sil; 50 mL/min; 100% ethyl acetate (7 CV) followed by a linear gradient (7 CV): 0%→100% acetone in ethyl acetate).

Clear colorless oil; ^1H NMR (600 MHz, 300 K, $CDCl_3$): δ = 3.66 (s, 3H), 3.42−3.34 (m, 4H), 3.30−3.25 (m, 1H), 2.73 (dd, J = 16.1, 9.5 Hz, 1H), 2.67−2.60 (m, 2H), 2.40 (dd, J = 16.1, 5.0 Hz, 1H), 2.11−2.04 (m, 2H), 1.66−1.62 (m, 1H), 1.45−1.30 (m, 3H), 0.93 ppm (t, J = 7.2 Hz, 3H); ^{13}C NMR (150 MHz, 300 K, $CDCl_3$): δ = 193.7, 172.7, 120.6, 54.4, 51.5, 44.9, 44.3, 43.3, 36.3, 35.1, 28.5, 28.4(7), 20.3, 14.1 ppm; IR (film): ṽ = 3484, 2956, 2873, 2168, 1733, 1577, 1437, 1352, 1288, 1223, 1154 cm^{-1}; HRMS (ESI): *m/z* calculated for $C_{14}H_{22}O_3NS$ $[M+H]^+$: 284.1315, found: 284.1316.

Dimethyl 2-oxo-3-propylpentanedioate (S14).

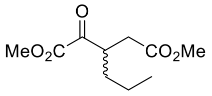

According to General Procedure G, dimethyl dicarboxylate **S14** (147 mg, 72%) was obtained from cyanosulfur ylid **S13** (270 mg, 0.95 mmol), following column chromatography (10 g Ultra; 35 mL/min; 100% cyclohexane (3 CV), followed by a linear gradient (20 CV): 0%→15% ethyl acetate in cyclohexane). The analytical data are in agreement with those reported.[19b]

Clear colorless oil; ^1H NMR (600 MHz, 300 K, $CDCl_3$): δ = 3.91 (s, 3H), 3.73−3.69 (m, 1H), 3.66 (s, 3H), 2.85 (dd, J = 17.1, 10.3 Hz, 1H), 2.57 (dd, J = 17.1, 4.4 Hz, 1H), 1.72−1.66 (m, 1H), 1.47−1.28 (m, 3H), 0.94 ppm (t, J = 7.3 Hz, 3H); ^{13}C NMR (150 MHz, 300 K, $CDCl_3$): δ = 196.2, 172.5, 161.4, 53.0, 52.0, 42.8, 35.4, 33.0, 20.1, 13.9 ppm; IR (film): ṽ = 2960, 2876, 1730, 1438, 1361, 1271, 1205, 1057 cm^{-1}; HRMS (ESI): *m/z* calculated for $C_{10}H_{17}O_5$ $[M+H]^+$: 217.1071, found: 217.1073.

2-Oxo-3-propylpentanedioic acid (18).

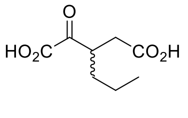

Dicarboxylic acid **18** (39 mg, 83%) was obtained from dimethyl 2-oxo-3-propylpentanedioate **S14** (54.1 mg, 0.25 mmol) according to General Procedure H. The chemical shifts in the ^{13}C NMR spectrum are in agreement with those of the reported corresponding dilithium salt.[19b]

White amorphous solid; ^{1}H NMR (600 MHz, 300 K, D_2O): δ = 3.39 (brs, 1H), 2.56 (dd, J = 15.5, 7.3 Hz, 1H), 2.35−2.32 (m, 1H), 1.69−1.63 (m, 1H), 1.52−1.46 (m, 1H), 1.39−1.26 (m, 2H), 0.91 ppm (t, J = 7.3 Hz, 3H); ^{13}C NMR (150 MHz, 300 K, D_2O): δ = 209.1 (br), 180.6, 171.3, 44.9, 37.1, 31.8, 19.6, 13.4 ppm; IR (film): $\tilde{v}$ = 3412, 2960, 2934, 2873, 1704, 1637, 1577, 1404, 1232, 1082 cm^{-1}; HRMS (ESI): *m/z* calculated for $C_8H_{11}O_5$ [M−H]$^-$: 187.0601, found: 187.0608.

Methyl 3-(2-cyano-2-(tetrahydro-1-λ^4-thiophen-1-ylidene)acetyl)-6,6-dimethylheptanoate (S15).

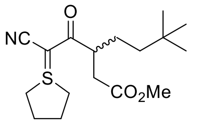

According to General Procedure D, 1-benzyl 4-methyl 2-(3,3-dimethylbutylidene)succinate (778 mg, 48%) was obtained from 1-benzyl 4-methyl 2-(dimethoxyphosphoryl)succinate **S5** (1.75 g, 5.3 mmol) and 3,3-dimethylbutyraldehyde, following column chromatography (25 g Ultra; 50 mL/min; 100% cyclohexane (3 CV), followed by a linear gradient (15 CV): 0%→15% ethyl acetate in cyclohexane). The clear pale yellow oil was a mixture of alkene diastereomers (1.3:1) which was used in the next reaction used without further purification. ^{1}H NMR (400 MHz, 300 K, $CDCl_3$): major diastereomer: δ = 7.41−7.31 (m, 5H), 7.14 (t, J = 7.9 Hz, 1H), 5.23 (s, 2H), 3.63 (s, 3H), 3.40 (s, 2H), 2.12 (d, J = 8.0 Hz, 2H), 0.97 ppm (s, 9H); minor diastereomer: δ = 7.41−7.31 (m, 5H), 6.18 (tt, J = 7.9, 1.0 Hz, 1H), 5.21 (s, 2H), 3.61 (s, 3H), 3.34−3.33 (m, 2H), 2.53 (d, J = 7.8 Hz, 2H), 0.93 ppm (s, 9H).

1-Benzyl 4-methyl 2-(3,3-dimethylbutylidene)succinate (778 mg, 2.56 mmol) was reduced according to General Procedure E to afford the corresponding crude mono-methyl dicarboxylic acid half-ester (quant.), which was used in the next reaction without further purification. According to General Procedure F, cyanosulfur ylid **S15** (207 mg, 25% over two steps) was obtained, following column chromatography (25 g Ultra; 50 mL/min; 100% ethyl acetate (8 CV), followed by a linear gradient (8 CV): 0%→100% acetone in ethyl acetate).

Clear yellow oil; ^{1}H NMR (600 MHz, 300 K, $CDCl_3$): δ = 3.66 (s, 3H), 3.42−3.34 (m, 4H), 3.22−3.17 (m, 1H), 2.74 (dd, J = 16.1, 9.5 Hz, 1H), 2.68−2.60 (m, 2H), 2.41 (dd, J = 16.1, 4.8 Hz, 1H), 2.11−2.04 (m, 2H), 1.65−1.60 (m, 1H), 1.46−1.40 (m, 1H), 1.28−1.18 (m, 2H), 0.90 ppm (s, 9H); ^{13}C NMR (150 MHz, 300 K, $CDCl_3$): δ = 193.7, 172.8, 120.5, 54.2, 51.5, 45.0, 44.3, 44.0, 40.9, 36.2, 30.2, 29.2, 28.5, 28.4(7), 27.9 ppm; IR (film): $\tilde{v}$ = 3481, 2952, 2866, 2169, 1734, 1585, 1438, 1363, 1304, 1243, 1200, 1153, 1087, 1019 cm^{-1}; HRMS (ESI): *m/z* calculated for $C_{17}H_{27}O_3NSNa$ [M+Na]$^+$: 348.1605, found: 348.1604.

Dimethyl 3-(3,3-dimethylbutyl)-2-oxopentanedioate (S16).

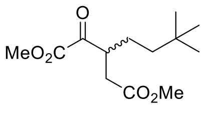

According to General Procedure G, dimethyl dicarboxylate **S16** (65 mg, 39%) was obtained from cyanosulfur ylid **S15** (207 mg, 0.64 mmol), following column chromatography (10 g Ultra; 35 mL/min; 100% cyclohexane (3 CV), followed by a linear gradient (15 CV): 0%→15% ethyl acetate in cyclohexane).

Clear colorless oil; ^{1}H NMR (600 MHz, 300 K, $CDCl_3$): δ = 3.92 (s, 3H), 3.67 (s, 3H), 3.66−3.62 (m, 1H), 2.85 (dd, J = 17.1, 10.2 Hz, 1H), 2.58 (dd, J = 17.1, 4.5 Hz, 1H), 1.72−1.66 (m, 1H), 1.46 (tdd, J = 13.0, 6.9, 4.7 Hz, 1H), 1.25−1.13 (m, 2H), 0.87 ppm (s, 9H); ^{13}C NMR (150 MHz, 300 K, $CDCl_3$): δ = 196.2, 172.4, 161.4, 53.0, 52.0, 43.4, 40.8, 35.4, 30.2, 29.1, 26.1 ppm; IR (film): $\tilde{v}$ = 2956, 2868, 1731, 1468, 1439, 1366, 1274, 1248, 1203, 1180, 1149, 1104, 1050 cm^{-1}; HRMS (ESI): *m/z* calculated for $C_{13}H_{22}O_5Na$ [M+Na]$^+$: 281.1359, found: 281.1359.

3-(3,3-Dimethylbutyl)-2-oxopentanedioic acid (19).

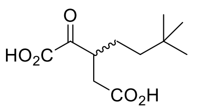

Dicarboxylic acid **19** (45 mg, 78%) was obtained from dimethyl 3-(3,3-dimethylbutyl)-2-oxopentanedioate **S16** (64.6 mg, 0.25 mmol) according to General Procedure H.

White solid, m.p.: >250 °C (decomposition); ^{1}H NMR (600 MHz, 300 K, D_2O): δ = 3.39 (quint., J = 6.6 Hz, 1H), 2.55 (dd, J = 15.3, 6.8 Hz, 1H), 2.29 (dd, J = 15.3, 7.8 Hz, 1H), 1.71−1.65 (m, 1H), 1.62−1.56 (m, 1H), 1.21 (td, J = 12.9, 4.7 Hz, 1H), 1.12 (td, J = 12.9, 4.5 Hz, 1H), 0.87 ppm (s, 9H); ^{13}C NMR (150 MHz, 300 K, D_2O): δ = 209.2, 180.9, 171.1, 45.6, 39.9, 37.2, 29.3, 28.4, 24.7 ppm; IR (film): ṽ = 3243, 2954, 2903, 2867, 1703, 1658, 1601, 1435, 1417, 1398, 1364, 1323, 1247, 1105 cm^{-1}; HRMS (ESI): *m/z* calculated for $C_{11}H_{17}O_5$ [M−H]$^-$: 229.1071, found: 229.1078.

Methyl 3-(2-cyano-2-(tetrahydro-1-λ^4-thiophen-1-ylidene)acetyl)-6-phenylhexanoate (S17).

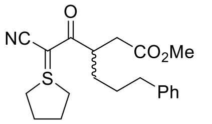

According to General Procedure D, 1-benzyl 4-methyl 2-(3-phenylpropylidene)succinate (1.14 g, 48%) was obtained from 1-benzyl 4-methyl 2-(dimethoxyphosphoryl)succinate **S5** (2.31 g, 7.0 mmol) and hydrocinnamaldehyde, following column chromatography (50 g KP-Sil; 50 mL/min; 100% cyclohexane (3 CV), followed by a linear gradient (12 CV): 0%→15% ethyl acetate in cyclohexane). The clear pale yellow oil was a mixture of alkene diastereomers (1.5:1) which was used in the next reaction without further purification. ^{1}H NMR (400 MHz, 300 K, $CDCl_3$): major diastereomer: δ = 7.41−7.28 (m, 6H), 7.26−7.14 (m, 4H), 7.08 (t, J = 7.5 Hz, 1H), 5.21 (s, 2H), 3.66 (s, 3H), 3.33 (s, 2H), 2.81−2.74 (m, 2H), 2.53 ppm (apparent q, J = 7.7 Hz, 2H); minor diastereomer: δ = 7.41−7.28 (m, 6H), 7.26−7.14 (m, 4H), 6.14 (tt, J = 7.4, 1.0 Hz, 1H), 5.20 (s, 2H), 3.60 (s, 3H), 3.30−3.29 (m, 2H), 2.95−2.89 (m, 2H), 2.81−2.74 ppm (m, 2H).

1-Benzyl 4-methyl 2-(3-phenylpropylidene)succinate (1.14 g, 3.37 mmol) was reduced according to General Procedure E to afford the corresponding crude mono-methyl dicarboxylic acid half-ester (quant.), which was used in the next reaction without further purification. According to General Procedure F, cyanosulfur ylid **S17** (466 mg, 38% over two steps) was obtained, following column chromatography (50 g KP-Sil; 50 mL/min; 100% ethyl acetate (7 CV), followed by a linear gradient (7 CV): 0%→100% acetone in ethyl acetate).

Clear yellow oil; ^{1}H NMR (600 MHz, 300 K, $CDCl_3$): δ = 7.34−7.24 (m, 2H), 7.21−7.17 (m, 3H), 3.65 (s, 3H), 3.40−3.35 (m, 4H), 3.33−3.28 (m, 1H), 2.73 (dd, J = 16.1, 9.4 Hz, 1H), 2.65−2.59 (m, 4H), 2.39 (dd, J = 16.1, 5.1 Hz, 1H), 2.10−2.04 (m, 2H), 1.74−1.64 (m, 3H), 1.55−1.48 ppm (m, 1H); ^{13}C NMR (150 MHz, 300 K, $CDCl_3$): δ = 193.3, 172.6, 142.2, 128.4, 128.3, 125.7, 120.5, 54.6, 51.5, 45.0, 44.3, 43.3, 36.4, 35.8, 32.5, 28.8, 28.5, 28.4(7) ppm; IR (film): ṽ = 3483, 3025, 2946, 2859, 2168, 1733, 1583, 1496, 1439, 1353, 1308, 1199, 1153, 1085 cm^{-1}; HRMS (ESI): *m/z* calculated for $C_{20}H_{25}O_3NSNa$ [M+Na]$^+$: 382.1447, found: 382.1449.

Dimethyl 2-oxo-3-(3-phenylpropyl)pentanedioate (S18).

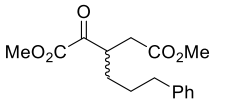

According to General Procedure G, dimethyl dicarboxylate **S18** (190 mg, 50%) was obtained from cyanosulfur ylid **S17** (466 mg, 1.30 mmol), following column chromatography (25 g Ultra; 50 mL/min; 100% cyclohexane (3 CV), followed by a linear gradient (15 CV): 0%→15% ethyl acetate in cyclohexane).

Clear colorless oil; ^{1}H NMR (600 MHz, 300 K, $CDCl_3$): δ = 7.30−7.28 (m, 2H), 7.21−7.19 (m, 1H), 7.16−7.15 (m, 2H), 3.90 (s, 3H), 3.75−3.70 (m, 1H), 3.66 (s, 3H), 2.84 (dd, J = 17.1, 10.2 Hz, 1H), 2.67−2.59 (m, 2H), 2.56 (dd, J = 17.1, 4.5 Hz, 1H), 1.79−1.73 (m, 1H), 1.71−1.60 (m, 2H), 1.54−1.48 ppm (m, 1H); ^{13}C NMR (150 MHz, 300 K, $CDCl_3$): δ = 196.0, 172.3, 161.3, 141.4, 128.4, 128.3, 126.0, 53.0, 52.0, 42.7, 35.5(4), 35.5, 30.4, 28.5 ppm; IR (film): ṽ = 3027, 2953, 2861, 1729, 1603, 1496, 1438, 1357, 1279, 1205, 1105, 1045 cm^{-1}; HRMS (ESI): *m/z* calculated for $C_{16}H_{20}O_5Na$ [M+Na]$^+$: 315.1203, found: 315.1203.

2-Oxo-3-(3-phenylpropyl)pentanedioic acid (20).

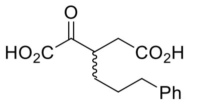

Dicarboxylic acid **20** (46 mg, 70%) was obtained from dimethyl 2-oxo-3-(3-phenylpropyl)pentanedioate **S18** (73.1 mg, 0.25 mmol) according to General Procedure H.
White solid, m.p.: >220 °C (decomposition); White amorphous solid; ^{1}H NMR (600 MHz, 300 K, D_2O): δ = 7.40−7.38 (m, 2H), 7.32 (d, J = 7.2 Hz, 2H), 7.29−7.27 (m, 1H), 3.43 (brs, 1H), 2.67 (t, J = 7.4

Hz, 2H), 2.55 (dd, J = 15.5, 7.2 Hz, 1H), 2.31 (dd, J = 14.9, 6.9 Hz, 1H), 1.75−1.60 (m, 3H), 1.59−1.52 ppm (m, 1H); ^{13}C NMR (150 MHz, 300 K, D_2O): δ = 208.8 (br), 180.5, 171.0, 142.7, 128.6, 128.5(6), 125.9, 44.9, 37.3, 35.0, 29.2, 28.0 ppm; IR (film): ṽ = 3398, 3027, 2928, 2860, 1704, 1637, 1576, 1497, 1404, 1110, 1079 cm^{-1}; HRMS (ESI): *m/z* calculated for $C_{14}H_{15}O_5$ [M−H]$^-$: 263.0914, found: 263.0921.

1-Benzyl 4-methyl (*E*)-2-benzylidenesuccinate (S19).

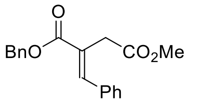

According to General Procedure C, succinate derivative **S19** (2.5 g, 81%) was obtained from 1-benzyl 4-methyl itaconate **S3**[8] (2.34 g, 10.0 mmol) and iodobenzene, following column chromatography (25 g Ultra; 45 mL/min; 100% cyclohexane (3 CV), followed by a linear gradient (8 CV): 0%→10% ethyl acetate in cyclohexane). The reaction product was isolated as a single alkene diastereoisomer, which was tentatively assigned based on the literature[9] as the *E*-isomer.

Clear yellow oil; ^{1}H NMR (400 MHz, 300 K, $CDCl_3$): δ = 7.98 (s, 1H), 7.44−7.35 (m, 10H), 5.30 (s, 2H), 3.68 (s, 3H), 3.59 ppm (s, 2H); ^{13}C NMR (100 MHz, 300 K, $CDCl_3$): δ = 171.5, 167.1, 142.4, 135.9, 134.9, 129.0, 128.9, 128.6, 128.5, 128.2, 128.1, 125.9, 66.9, 52.1, 33.5 ppm; HRMS (ESI): *m/z* calculated for $C_{19}H_{18}O_4Na$ [M+Na]$^+$: 333.1097, found: 333.1096.

Methyl 3-benzyl-5-cyano-4-oxo-5-(tetrahydro-1-λ^4-thiophen-1-ylidene)pentanoate (S20).

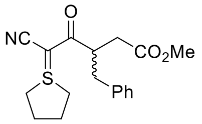

1-Benzyl 4-methyl (*E*)-2-benzylidenesuccinate **S19** (3.67 g, 11.8 mmol) was reduced according to General Procedure E to afford the corresponding crude mono-methyl dicarboxylic acid half-ester **13h** (91%), which was used in the next step without further purification. According to General Procedure F, cyanosulfur ylid **S20** (1.89 g, 48% over two steps) was obtained, following column chromatography (50 g KP-Sil; 50 mL/min; 100% ethyl acetate (8 CV), followed by a linear gradient (7 CV): 0%→100% acetone in ethyl acetate). Some impurities were separated after the next reaction; characteristic analytical data for the major product are given.

Clear orange oil; ^{1}H NMR (600 MHz, 300 K, $CDCl_3$): δ = 7.30−7.25 (m, 4H), 7.22−7.20 (m, 1H), 3.62 (s, 3H), 3.55 (dddd, J = 9.5, 8.7, 6.8, 4.7 Hz, 1H), 3.32−3.27 (m, 3H), 3.20−3.16 (m, 1H), 2.98 (dd, J = 13.4, 6.8 Hz, 1H), 2.77 (dd, J = 16.4, 9.6 Hz, 1H), 2.67 (dd, J = 13.3, 8.6 Hz, 1H), 2.64−2.51 (m, 2H), 2.37 (dd, J = 16.4, 4.6 Hz, 1H), 2.10−2.00 ppm (m, 2H); ^{13}C NMR (150 MHz, 300 K, $CDCl_3$): δ = 192.5, 172.5, 138.8, 129.3, 128.3, 126.3, 120.3, 54.7, 51.5, 45.3, 44.7, 44.6, 38.9, 35.4, 28.4(3), 28.4 ppm; IR (film): ṽ = 3456, 3025, 2981, 2952, 2168, 1733, 1580, 1438, 1354, 1302, 1267, 1202, 1154, 1084 cm^{-1}; HRMS (ESI): *m/z* calculated for $C_{18}H_{21}O_3NSNa$ [M+Na]$^+$: 354.1134, found: 354.1136.

Dimethyl 3-benzyl-2-oxopentanedioate (S21).

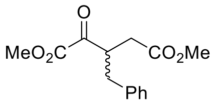

According to General Procedure G, dimethyl dicarboxylate **S21** (854 mg, 57%) was obtained from cyanosulfur ylid **S20** (1.89 g, 5.70 mmol), following column chromatography (50 g KP-Sil; 50 mL/min; 100% cyclohexane (3 CV), followed by a linear gradient (8 CV): 0%→15% ethyl acetate in cyclohexane). The analytical data are in agreement with those reported.[20]

Clear colorless oil; ^{1}H NMR (600 MHz, 300 K, $CDCl_3$): δ = 7.33−7.30 (m, 2H), 7.26−7.24 (m, 1H), 7.20−7.18 (m, 2H), 4.00 (dddd, J = 10.2, 8.8, 5.9, 4.4 Hz, 1H), 3.86 (s, 3H), 3.63 (s, 3H), 3.09 (dd, J = 13.7, 6.0 Hz, 1H), 2.83 (dd, J = 17.3, 10.2 Hz, 1H), 2.65 (dd, J = 13.7, 8.8 Hz, 1H), 2.52 ppm (dd, J = 17.3, 4.4 Hz, 1H); ^{13}C NMR (150 MHz, 300 K, $CDCl_3$): δ = 195.4, 172.2, 161.0, 137.3, 129.1, 128.7, 126.9, 53.0, 52.0, 44.9, 36.8, 34.9 ppm; IR (film): ṽ = 3030, 2955, 1730, 1438, 1358, 1265, 1206, 1105, 1048, 991 cm^{-1}; HRMS (ESI): *m/z* calculated for $C_{14}H_{16}O_5Na$ [M+Na]$^+$: 287.0890, found: 287.0891.

3-Benzyl-2-oxopentanedioic acid (21).

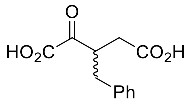

Dicarboxylic acid **21** (236 mg, quant.) was obtained from dimethyl 3-benzyl-2-oxopentanedioate **S21** (264 mg, 1.0 mmol) according to General Procedure H.
White solid, m.p.: >250 °C (decomposition); ^{1}H NMR (600 MHz, 300 K, D_2O): δ = 7.40−7.37 (m, 2H), 7.32−7.29 (m, 3H), 3.80 (pent., J = 7.0 Hz, 1H), 3.06 (dd, J = 13.9, 6.9 Hz, 1H), 2.85 (dd, J = 13.9, 6.8 Hz, 1H), 2.52 (dd, J = 15.8, 7.3 Hz, 1H), 2.27 ppm (dd, J = 15.8, 7.0 Hz, 1H); ^{13}C NMR (150 MHz, 300 K, D_2O): δ = 207.7, 180.2, 170.0, 138.8, 129.3, 128.5, 126.5, 46.7, 37.2, 35.6 ppm; IR (film): ṽ = 3390, 3028, 2981, 1703, 1634, 1572, 1496, 1404, 1361, 1220, 1110, 1075 cm^{-1}; HRMS (ESI): *m/z* calculated for $C_{12}H_{11}O_5$ [M−H]$^{-}$: 235.0612, found: 235.0609.

1-Benzyl 4-methyl (*E*)-2-(4-fluorobenzylidene)succinate (S22).

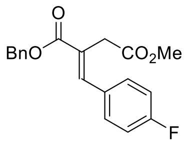

According to General Procedure C, succinate derivative **S22** (3.58 g, 73%) was obtained from 1-benzyl 4-methyl itaconate **S3**[8] (3.51 g, 15.0 mmol) and 4-fluoroiodobenzene, following column chromatography (100 g KP-Sil; 60 mL/min; 100% cyclohexane (3 CV), followed by a linear gradient (9 CV): 0%→7% ethyl acetate in cyclohexane). The reaction product was isolated as a single alkene diastereoisomer, which was tentatively assigned based on the literature[9] as the *E*-isomer.
Clear pale yellow oil; ^{1}H NMR (600 MHz, 300 K, $CDCl_3$): δ = 7.92 (s, 1H), 7.42−7.35 (m, 7H), 7.12−7.09 (m, 2H), 5.29 (s, 2H), 3.69 (s, 3H), 3.55 ppm (s, 2H); ^{13}C NMR (150 MHz, 300 K, $CDCl_3$): δ = 171.5, 167.0, 163.0 (d, J = 249.8 Hz), 141.3, 135.8, 131.0 (d, J = 8.0 Hz), 130.9 (d, J = 3.5 Hz), 128.6, 128.3, 128.2, 125.9, 115.8 (d, J = 21.9 Hz), 67.0, 52.2, 33.5 ppm; ^{19}F NMR (565 MHz, 300 K, $CDCl_3$): δ = −111.4 ppm (m, 1F); IR (film): ṽ = 3067, 3035, 2953, 1736, 1709, 1643, 1601, 1508, 1455, 1436, 1383, 1330, 1265, 1224, 1194, 1175, 1159, 1092, 1012 cm^{-1}; HRMS (ESI): *m/z* calculated for $C_{19}H_{17}O_4FNa$ [M+Na]$^{+}$: 351.1003, found: 351.1004.

Methyl 5-cyano-3-(4-fluorobenzyl)-4-oxo-5-(tetrahydro-1-λ^{4}-thiophen-1-ylidene)pentanoate (S23).

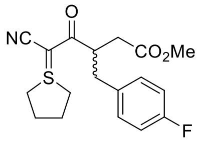

1-Benzyl 4-methyl (*E*)-2-(4-fluorobenzylidene)succinate **S22** (3.58 g, 10.9 mmol) was reduced according to General Procedure E to afford the corresponding crude mono-methyl dicarboxylic acid half-ester **13i** (quant.), which was used in the next reaction without further purification. According to General Procedure F, cyanosulfur ylid **S23** (2.02 g, 58% over two steps) was obtained, following column chromatography (100 g KP-Sil; 60 mL/min; 100% ethyl acetate (8 CV), followed by a linear gradient (7 CV): 0%→100% acetone in ethyl acetate).
Clear pale orange oil; ^{1}H NMR (600 MHz, 300 K, $CDCl_3$): δ = 7.22−7.20 (m, 2H), 6.99−6.96 (m, 2H), 3.63 (s, 3H), 3.54−3.49 (m, 1H), 3.35−3.27 (m, 3H), 3.21−3.16 (m, 1H), 2.94 (dd, J = 13.4, 7.1 Hz, 1H), 2.76 (dd, J = 16.4, 9.4 Hz, 1H), 2.66 (dd, J = 13.4, 8.2 Hz, 1H), 2.62−2.52 (m, 2H), 2.36 (dd, J = 16.4, 4.9 Hz, 1H), 2.08−2.01 ppm (m, 2H); ^{13}C NMR (150 MHz, 300 K, $CDCl_3$): δ = 192.2, 172.4, 161.6 (d, J = 244.3 Hz), 134.5 (d, J = 3.2 Hz), 130.7 (d, J = 7.9 Hz), 120.2, 115.1 (d, J = 21.2 Hz), 54.8, 51.6, 45.3, 44.7, 44.6, 38.0, 35.4, 28.4 (2C) ppm; ^{19}F NMR (565 MHz, 300 K, $CDCl_3$): δ = −116.8 ppm (m, 1F); IR (film): ṽ = 3483, 3010, 2951, 2169, 1733, 1589, 1509, 1439, 1355, 1297, 1268, 1219, 1156, 1095, 995 cm^{-1}; HRMS (ESI): *m/z* calculated for $C_{18}H_{21}O_3FNS$ [M+H]$^{+}$: 350.1221, found: 350.1220.

Dimethyl 3-(4-fluorobenzyl)-2-oxopentanedioate (S24).

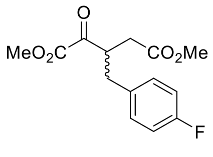

According to General Procedure G, dimethyl dicarboxylate **S24** (978 mg, 60%) was obtained from cyanosulfur ylid **S23** (2.02 g, 5.79 mmol), following column chromatography (50 g KP-Sil; 50 mL/min; 100% cyclohexane (3 CV), followed by a linear gradient (8 CV): 0%→15% ethyl acetate in cyclohexane).
Clear colorless oil; ^{1}H NMR (600 MHz, 300 K, $CDCl_3$): δ = 7.17−7.14 (m, 2H), 7.02−6.98 (m, 2H), 3.97 (dddd, J = 10.1, 8.4, 6.1, 4.5 Hz, 1H), 3.87 (s, 3H), 3.64 (s, 3H), 3.05 (dd, J = 13.8, 6.2 Hz, 1H), 2.82 (dd, J = 17.2, 10.0

Hz, 1H), 2.65 (dd, *J* = 13.8, 8.5 Hz, 1H), 2.51 ppm (dd, *J* = 17.2, 4.5 Hz, 1H); ^{13}C NMR (150 MHz, 300 K, $CDCl_3$): δ = 195.3, 172.1, 161.8 (d, *J* = 245.7 Hz), 161.0, 133.0 (d, *J* = 3.2 Hz), 130.6 (d, *J* = 7.8 Hz), 115.5 (d, *J* = 21.1 Hz), 53.1 (d, *J* = 3.1 Hz), 52.0 (d, *J* = 1.9 Hz), 44.8, 36.0, 34.9 ppm; ^{19}F NMR (565 MHz, 300 K, $CDCl_3$): δ = −115.8 ppm (m, 1F); IR (film): ṽ = 2981, 2957, 1729, 1603, 1510, 1438, 1358, 1265, 1221, 1159, 1089, 1048 cm^{-1}; HRMS (ESI): *m/z* calculated for $C_{14}H_{15}O_5FNa$ [M+Na]$^+$: 305.0796, found: 305.0797.

3-(4-Fluorobenzyl)-2-oxopentanedioic acid (22).

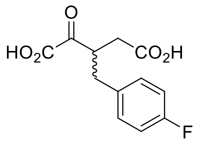

Dicarboxylic acid **22** (46 mg, 90%) was obtained from dimethyl 3-(4-fluorobenzyl)-2-oxopentanedioate **S24** (56.5 mg, 0.2 mmol) according to General Procedure H.

White solid, m.p.: >250 °C (decomposition); ^{1}H NMR (600 MHz, 300 K, D_2O): δ = 7.28−7.25 (m, 2H), 7.11−7.07 (m, 2H), 3.77 (pent., *J* = 7.0 Hz, 1H), 3.03 (dd, *J* = 14.0, 7.1 Hz, 1H), 2.84 (dd, *J* = 14.0, 6.7 Hz, 1H), 2.52 (dd, *J* = 15.8, 7.2 Hz, 1H), 2.27 ppm (dd, *J* = 15.8, 7.2 Hz, 1H); ^{13}C NMR (150 MHz, 300 K, D_2O): δ = 207.7, 180.1, 169.9, 161.4 (d, *J* = 241.2 Hz), 134.4 (d, *J* = 3.0 Hz), 130.8 (d, *J* = 7.8 Hz), 115.0 (d, *J* = 21.1 Hz), 46.7, 37.3, 34.9 ppm; ^{19}F NMR (565 MHz, 300 K, D_2O): δ = −117.5 ppm (m, 1F); IR (film): ṽ = 3392, 2981, 1702, 1636, 1575, 1509, 1404, 1361, 1222, 1159, 1092 cm^{-1}; HRMS (ESI): *m/z* calculated for $C_{12}H_{10}O_5F$ [M−H]$^-$: 253.0518, found: 253.0517.

1-Benzyl 4-methyl (*E*)-2-(4-(trifluoromethoxy)benzylidene)succinate (S25).

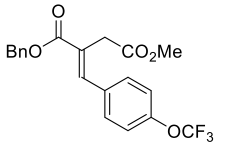

According to General Procedure C, succinate derivative **S25** (3.96 g, 67%) was obtained from 1-benzyl 4-methyl itaconate **S3**[8] (3.51 g, 15.0 mmol) and 1-iodo-4-trifluoromethoxybenzene, following column chromatography (100 g KP-Sil; 60 mL/min; 100% cyclohexane (3 CV), followed by a linear gradient (9 CV): 0%→8% ethyl acetate in cyclohexane). The reaction product was isolated as a single alkene diastereoisomer, which was tentatively assigned based on the literature[9] as the *E*-isomer.

Clear yellow oil; ^{1}H NMR (600 MHz, 300 K, $CDCl_3$): δ = 7.92 (s, 1H), 7.43−7.38 (m, 6H), 7.37−7.35 (m, 1H), 7.26 (brd, *J* = 8.1 Hz, 2H), 5.29 (s, 2H), 3.69 (s, 3H), 3.54 ppm (s, 2H); ^{13}C NMR (150 MHz, 300 K, $CDCl_3$): δ = 171.3, 166.8, 149.4 (m), 140.8, 135.7, 133.4, 130.6, 128.6, 128.3, 128.2, 126.8, 121.0, 120.4 (q, *J* = 257.9 Hz), 67.1 (m), 52.2 (m), 33.5 ppm; ^{19}F NMR (565 MHz, 300 K, $CDCl_3$): δ = −57.8 ppm (s, 3F); IR (film): ṽ = 3068, 3036, 2955, 1737, 1713, 1508, 1437, 1330, 1254, 1211, 1167, 1093, 1017 cm^{-1}; HRMS (ESI): *m/z* calculated for $C_{20}H_{17}O_5F_3Na$ [M+Na]$^+$: 417.0920, found: 417.0920.

Methyl 5-cyano-4-oxo-5-(tetrahydro-1-λ^4-thiophen-1-ylidene)-3-(4-(trifluoromethoxy)benzyl)pentanoate (S26).

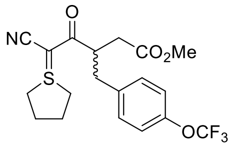

1-Benzyl 4-methyl (*E*)-2-(4-(trifluoromethoxy)benzylidene)succinate **S25** (3.96 g, 10.0 mmol) was reduced according to General Procedure E to afford the corresponding crude mono-methyl dicarboxylic acid half-ester **13j** (99%), which was used in the next reaction without further purification. According to General Procedure F, cyanosulfur ylid **S26** (2.39 g, 58% over two steps) was obtained, following column chromatography (100 g KP-Sil; 60 mL/min; 100% ethyl acetate (8 CV), followed by a linear gradient (7 CV): 0%→100% acetone in ethyl acetate).

Clear pale brown oil; ^{1}H NMR (600 MHz, 300 K, $CDCl_3$): δ = 7.29−7.27 (m, 2H), 7.14 (d, *J* = 7.9 Hz, 2H), 3.64 (s, 3H), 3.57−3.52 (m, 1H), 3.34−3.25 (m, 3H), 3.12−3.08 (m, 1H), 2.95 (dd, *J* = 13.4, 7.5 Hz, 1H), 2.77 (dd, *J* = 16.4, 9.2 Hz, 1H), 2.72 (dd, *J* = 13.4, 8.0 Hz, 1H), 2.60−2.48 (m, 2H), 2.38 (dd, *J* = 16.4, 5.0 Hz, 1H), 2.07−2.00 ppm (m, 2H); ^{13}C NMR (150 MHz, 300 K, $CDCl_3$): δ = 191.9, 172.3, 147.8, 137.7, 130.6, 120.8, 120.5 (q, *J* = 256.5 Hz), 120.1, 55.2, 51.6, 45.1, 44.9, 44.6, 38.1, 35.5, 28.4, 28.3(8) ppm; ^{19}F NMR (565 MHz, 300 K, $CDCl_3$): δ = −57.9 ppm (s, 3F); IR (film): ṽ = 3471, 2953, 2169, 1734, 1589, 1509, 1439, 1356, 1260, 1222, 1199, 1156 cm^{-1}; HRMS (ESI): *m/z* calculated for $C_{19}H_{21}O_3F_3NS$ [M+H]$^+$: 416.1138, found: 416.1137.

Dimethyl 2-oxo-3-(4-(trifluoromethoxy)benzyl)pentanedioate (S27).

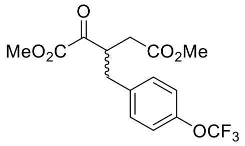

According to General Procedure G, dimethyl dicarboxylate **S27** (1.25 g, 62%) was obtained from cyanosulfur ylid **S26** (2.39 g, 5.74 mmol), following column chromatography (50 g KP-Sil; 50 mL/min; 100% cyclohexane (3 CV), followed by a linear gradient (8 CV): 0%→13% ethyl acetate in cyclohexane).

Clear colorless oil; ^{1}H NMR (600 MHz, 300 K, $CDCl_3$): δ = 7.24−7.21 (m, 2H), 7.14 (d, J = 8.0 Hz, 2H), 3.99 (dddd, J = 9.9, 8.4, 6.3, 4.6 Hz, 1H), 3.86 (s, 3H), 3.64 (s, 3H), 3.08 (dd, J = 13.8, 6.4 Hz, 1H), 2.83 (dd, J = 17.2, 9.9 Hz, 1H), 2.69 (dd, J = 13.8, 8.4 Hz, 1H), 2.38 ppm (dd, J = 17.2, 4.6 Hz, 1H); ^{13}C NMR (150 MHz, 300 K, $CDCl_3$): δ = 195.1, 172.0, 160.9, 148.2, 136.1, 130.5, 121.2, 120.4 (q, J = 257.1 Hz), 53.1, 52.1, 44.7, 36.1, 35.0 ppm; ^{19}F NMR (565 MHz, 300 K, $CDCl_3$): δ = −57.9 ppm (s, 3F); IR (film): ṽ = 3014, 2957, 1732, 1509, 1439, 1361, 1260, 1222, 1201, 1164, 1097, 1048 cm^{-1}; HRMS (ESI): *m/z* calculated for $C_{15}H_{15}O_6$ F_3Na $[M+Na]^+$: 371.0713, found: 371.0712.

2-Oxo-3-(4-(trifluoromethoxy)benzyl)pentanedioic acid (23).

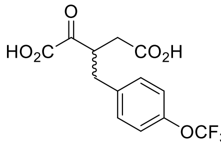

Dicarboxylic acid **23** (300 mg, 94%) was obtained from dimethyl 2-oxo-3-(4-(trifluoromethoxy)benzyl)pentanedioate **S27** (348 mg, 1.0 mmol) according to General Procedure H.

White solid, m.p.: >260 °C (decomposition); ^{1}H NMR (600 MHz, 300 K, D_2O): δ = 7.35−7.34 (m, 2H), 7.29 (d, J = 8.3 Hz, 2H), 3.78 (pent., J = 7.0 Hz, 1H), 3.07 (dd, J = 14.0, 7.0 Hz, 1H), 2.87 (dd, J = 14.0, 6.7 Hz, 1H), 2.53 (dd, J = 15.8, 7.3 Hz, 1H), 2.27 ppm (dd, J = 15.8, 7.0 Hz, 1H); ^{13}C NMR (150 MHz, 300 K, D_2O): δ = 207.5, 180.0, 169.9, 147.5 (q, J = 2.0 Hz), 137.8, 130.7, 121.0, 120.3 (q, J = 255.7 Hz), 46.6, 37.3, 34.9 ppm; ^{19}F NMR (565 MHz, 300 K, D_2O): δ = −57.8 ppm (s, 3F); IR (film): ṽ = 3390, 2981, 2928, 1704, 1637, 1576, 1438, 1360, 1264, 1205, 1101, 1047 cm^{-1}; HRMS (ESI): *m/z* calculated for $C_{13}H_{10}O_6F_3$ $[M-H]^-$: 319.0435, found: 319.0433.

1-Benzyl 4-methyl (*E*)-2-(4-methoxybenzylidene)succinate (S28).

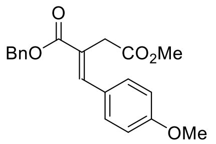

According to General Procedure C, succinate derivative **S28** (3.29 g, 64%) was obtained from 1-benzyl 4-methyl itaconate **S3**[8] (3.51 g, 15.0 mmol) and 4-iodoanisol, following column chromatography (100 g KP-Sil; 60 mL/min; 100% cyclohexane (3 CV), followed by a linear gradient (10 CV): 0%→10% ethyl acetate in cyclohexane). The reaction product was isolated as a single alkene diastereoisomer, which was tentatively assigned based on the literature[9] as the *E*-isomer.

Clear yellow oil; ^{1}H NMR (600 MHz, 300 K, $CDCl_3$): δ = 7.91 (s, 1H), 7.43−7.38 (m, 4H), 7.37−7.33 (m, 3H), 6.95−6.92 (m, 2H), 5.28 (s, 2H), 3.85 (s, 3H), 3.69 (s, 3H), 3.61 ppm (s, 2H); ^{13}C NMR (150 MHz, 300 K, $CDCl_3$): δ = 171.7, 167.4, 160.3, 142.2, 136.0, 130.9, 128.5, 128.2, 128.1, 127.3, 123.8, 114.1, 66.8, 55.3, 52.1, 33.6 ppm; IR (film): ṽ = 3065, 3034, 3000, 2953, 1736, 1704, 1637, 1605, 1511, 1456, 1436, 1382, 1304, 1253, 1170, 1091, 1029 cm^{-1}; HRMS (ESI): *m/z* calculated for $C_{20}H_{20}O_5Na$ $[M+Na]^+$: 363.1203, found: 363.1201.

Methyl 5-cyano-3-(4-methoxybenzyl)-4-oxo-5-(tetrahydro-1-λ^4-thiophen-1-ylidene)pentanoate (S29).

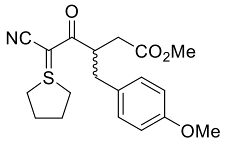

1-Benzyl 4-methyl (*E*)-2-(4-methoxybenzylidene)succinate **S28** (3.29 g, 9.67 mmol) was reduced according to General Procedure E to afford the corresponding crude mono-methyl dicarboxylic acid half-ester **13k** (94%), which was used in the next reaction without further purification. According to General Procedure F, cyanosulfur ylid **S29** (1.78 g, 51% over two steps) was obtained, following column chromatography (100 g KP-Sil; 50 mL/min; 100% ethyl acetate (7 CV), followed by a linear gradient (7 CV): 0%→100% acetone in ethyl acetate).

Clear orange oil; ^{1}H NMR (600 MHz, 300 K, $CDCl_3$): δ = 7.17−7.16 (m, 2H), 6.84−6.82 (m, 2H), 3.80 (s, 3H), 3.62 (s, 3H), 3.51−3.48 (m, 1H), 3.33−3.28 (m, 3H), 3.24−3.20 (m, 1H), 2.93 (dd, J = 13.5, 6.7 Hz, 1H), 2.75 (dd, J = 16.4, 9.7 Hz, 1H), 2.62−2.52 (m, 3H), 2.35 (dd, J = 16.4, 4.5 Hz, 1H), 2.08−2.00 ppm (m, 2H); ^{13}C NMR (150 MHz, 300 K, $CDCl_3$): δ = 192.6, 172.6, 158.2, 130.9, 130.3, 120.4, 113.7, 55.2, 54.5, 51.5, 45.5, 44.6, 44.5(6), 38.0, 35.3, 28.4(3), 28.4 ppm; IR (film): ṽ = 3482, 2981, 2953, 2168, 1733, 1585, 1513, 1440, 1354, 1301, 1247, 1178, 1154, 1033 cm^{-1}; HRMS (ESI): *m/z* calculated for $C_{19}H_{23}O_4NSNa$ $[M+Na]^+$: 384.1240, found: 384.1241.

Dimethyl 3-(4-methoxybenzyl)-2-oxopentanedioate (S30).

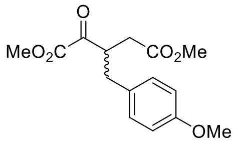

According to General Procedure G, dimethyl dicarboxylate **S30** (855 mg, 59%) was obtained from cyanosulfur ylid **S29** (1.78 g, 4.92 mmol), following column chromatography (50 g KP-Sil; 50 mL/min; 100% cyclohexane (3 CV), followed by a linear gradient (8 CV): 0%→17% ethyl acetate in cyclohexane).

Clear colorless oil; ^{1}H NMR (600 MHz, 300 K, $CDCl_3$): δ = 7.11−7.09 (m, 2H), 6.86−6.84 (m, 2H), 3.96 (dddd, J = 10.2, 8.7, 5.9, 4.4 Hz, 1H), 3.87 (s, 3H), 3.80 (s, 3H), 3.63 (s, 3H), 3.03 (dd, J = 13.7, 6.0 Hz, 1H), 2.81 (dd, J = 17.2, 10.2 Hz, 1H), 2.60 (dd, J = 13.8, 8.7 Hz, 1H), 2.51 ppm (dd, J = 17.3, 4.4 Hz, 1H); ^{13}C NMR (150 MHz, 300 K, $CDCl_3$): δ = 195.6, 172.2, 161.0, 158.6, 130.1, 129.2, 114.1, 55.3, 53.0, 52.0, 45.1, 36.0, 34.8 ppm; IR (film): ṽ = 2997, 2956, 1730, 1613, 1514, 1439, 1357, 1249, 1205, 1179, 1095, 1035 cm^{-1}; HRMS (ESI): *m/z* calculated for $C_{15}H_{18}O_6Na$ $[M+Na]^+$: 317.0996, found: 317.0997.

3-(4-Methoxybenzyl)-2-oxopentanedioic acid (24).

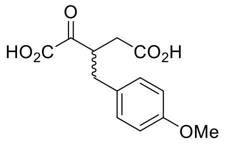

Dicarboxylic acid **24** (232 mg, 87%) was obtained from dimethyl 3-(4-methoxybenzyl)-2-oxopentanedioate **S30** (294 mg, 1.0 mmol) according to General Procedure H.

White solid, m.p.: >260 °C (decomposition); ^{1}H NMR (600 MHz, 300 K, D_2O): δ = 7.24−7.21 (m, 2H), 6.98−6.96 (m, 2H), 3.84 (s, 3H), 3.71 (brs, 1H), 3.00 (dd, J = 14.0, 6.7 Hz, 1H), 2.78 (dd, J = 14.0, 7.1 Hz, 1H), 2.52 (dd, J = 16.1, 7.8 Hz, 1H), 2.27 ppm (dd, J = 16.0, 6.8 Hz, 1H); ^{13}C NMR (150 MHz, 300 K, D_2O): δ = 208.2 (br), 179.8, 170.2 (br), 157.4, 131.3, 130.4, 114.0, 55.4, 46.6, 36.7, 34.7 ppm; IR (film): ṽ = 3275, 2964, 2933, 1702, 1627, 1567, 1513, 1432, 1403, 1358, 1302, 1243, 1177, 1103, 1030 cm^{-1}; HRMS (ESI): *m/z* calculated for $C_{13}H_{13}O_6$ $[M-H]^-$: 265.0718, found: 265.0718.

1-Benzyl 4-methyl (*E*)-2-(3,5-dimethylbenzylidene)succinate (S31).

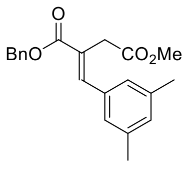

According to General Procedure C, succinate derivative **S31** (3.48 g, 69%) was obtained from 1-benzyl 4-methyl itaconate **S3**[8] (3.51 g, 15.0 mmol) and 3,5-dimethyliodobenzene, following column chromatography (100 g KP-Sil; 60 mL/min; 100% cyclohexane (3 CV), followed by a linear gradient (10 CV): 0%→10% ethyl acetate in cyclohexane). The reaction product was isolated as a single alkene diastereoisomer, which was tentatively assigned based on the literature[9] as the *E*-isomer.

Clear yellow oil; ^{1}H NMR (600 MHz, 300 K, $CDCl_3$): δ = 7.91 (s, 1H), 7.43−7.38 (m, 4H), 7.37−7.34 (m, 1H), 7.00 (s, 1H), 6.97 (s, 2H), 5.28 (s, 2H), 3.68 (s, 3H), 3.59 (s, 2H), 2.33 ppm (s, 6H); ^{13}C NMR (150 MHz, 300 K, $CDCl_3$): δ = 171.6, 167.2, 142.8, 138.2, 136.0, 134.8, 130.7, 128.5, 128.2, 128.1, 126.8, 125.5, 66.8, 52.0, 33.6, 21.3 ppm; IR (film): ṽ = 3064, 3033, 2951, 2919, 1738, 1709, 1640, 1600, 1498, 1455, 1435, 1381, 1329, 1280, 1246, 1192, 1170, 1094, 1011 cm^{-1}; HRMS (ESI): *m/z* calculated for $C_{21}H_{22}O_4Na$ $[M+Na]^+$: 361.1410, found: 361.1416.

Methyl 5-cyano-3-(3,5-dimethylbenzyl)-4-oxo-5-(tetrahydro-1-λ^4-thiophen-1-ylidene)pentanoate (S32).

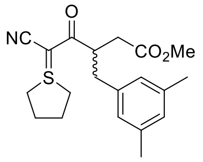

1-Benzyl 4-methyl (*E*)-2-(3,5-dimethylbenzylidene)succinate **S31** (3.48 g, 10.3 mmol) was reduced according to General Procedure E to afford the corresponding crude mono-methyl dicarboxylic acid half-ester **13l** (quant.), which was used in the next reaction without further purification. According to General Procedure F, cyanosulfur ylid **S32** (1.40 g, 38% over two steps) was obtained, following column chromatography (100 g KP-Sil; 60 mL/min; 100% ethyl acetate (8 CV), followed by a linear gradient (7 CV): 0%→100% acetone in ethyl acetate).

Salmon-colored solid, m.p.: 108−109 °C; ^{1}H NMR (600 MHz, 300 K, $CDCl_3$): δ = 6.86 (s, 2H), 6.84 (s, 1H), 3.62 (s, 3H), 3.54−3.49 (m, 1H), 3.36−3.27 (m, 3H), 3.24−3.20 (m, 1H), 2.92 (dd, J = 13.2, 6.4 Hz, 1H), 2.76 (dd, J = 16.5, 10.0 Hz, 1H), 2.63−2.52 (m, 3H), 2.35 (dd, J = 16.5, 4.4 Hz, 1H), 2.29 (s, 6H), 2.08−2.01 ppm (m, 2H); ^{13}C NMR (150 MHz, 300 K, $CDCl_3$): δ = 192.7, 172.6, 138.6, 137.7, 127.9, 127.1, 120.3, 54.4, 51.5, 45.2, 44.6, 44.4, 38.8, 35.2, 28.4(4), 28.4, 21.2 ppm; IR (film): ṽ = 3481, 3008, 2950, 2918, 2168, 1733, 1592, 1438, 1354, 1297, 1260, 1201, 1153 cm^{-1}; HRMS (ESI): *m/z* calculated for $C_{20}H_{25}O_3NSNa$ [M+Na]$^+$: 382.1458, found: 382.1450.

Dimethyl 3-(3,5-dimethylbenzyl)-2-oxopentanedioate (S33).

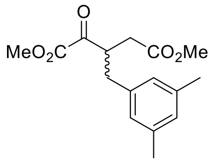

According to General Procedure G, dimethyl dicarboxylate **S33** (662 mg, 58%) was obtained from cyanosulfur ylid **S32** (1.40 g, 3.9 mmol), following column chromatography (50 g KP-Sil; 50 mL/min; 100% cyclohexane (3 CV), followed by a linear gradient (8 CV): 0%→15% ethyl acetate in cyclohexane).

Clear colorless oil; ^{1}H NMR (600 MHz, 300 K, $CDCl_3$): δ = 6.88 (s, 1H), 6.80 (s, 2H), 3.99−3.94 (m, 1H), 3.88 (s, 3H), 3.63 (s, 3H), 3.03 (dd, J = 13.5, 5.6 Hz, 1H), 2.82 (dd, J = 17.3, 10.4 Hz, 1H), 2.53−2.49 (m, 2H), 2.30 ppm (s, 6H); ^{13}C NMR (150 MHz, 300 K, $CDCl_3$): δ = 195.5, 172.3, 161.1, 138.2, 137.2, 128.5, 126.9, 53.0, 51.9, 44.9, 36.6, 34.7, 21.2 ppm; IR (film): ṽ = 3014, 2954, 2920, 1730, 1606, 1438, 1356, 1262, 1204, 1103, 1048, 992 cm^{-1}; HRMS (ESI): *m/z* calculated for $C_{16}H_{20}O_5Na$ [M+Na]$^+$: 315.1203, found: 315.1204.

3-(3,5-Dimethylbenzyl)-2-oxopentanedioic acid (25).

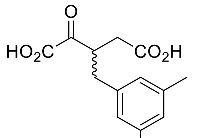

Dicarboxylic acid **25** (119 mg, 90%) was obtained from dimethyl 3-(3,5-dimethylbenzyl)-2-oxopentanedioate **S33** (146 mg, 0.5 mmol) according to General Procedure H.

White solid, m.p.: >270 °C (decomposition); ^{1}H NMR (600 MHz, 300 K, D_2O): δ = 6.99 (s, 1H), 6.94 (s, 2H), 3.71 (brs, 1H), 3.00 (dd, J = 13.8, 6.5 Hz, 1H), 2.73 (dd, J = 13.8, 7.3 Hz, 1H), 2.52 (dd, J = 16.0, 7.9 Hz, 1H), 2.31−2.25 ppm (m, 7H); ^{13}C NMR (150 MHz, 300 K, D_2O): δ = 207.1 (br), 179.8, 170.2 (br), 138.9, 138.7, 127.8, 127.7(6), 126.9, 126.8(5), 46.4, 36.6, 35.3, 20.2 ppm; IR (film): ṽ = 3391, 3011, 2981, 2918, 1700, 1629, 1605, 1569, 1403, 1356, 1216, 1104 cm^{-1}; HRMS (ESI): *m/z* calculated for $C_{14}H_{15}O_5$ [M−H]$^-$: 263.0925, found: 263.0925.

Dimethyl 4-methyl-2-oxopentanedioate (S34).

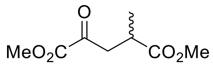

3-(Methoxycarbonyl)but-3-enoic acid[21] (690 mg, 4.8 mmol) was reduced according to General Procedure E to afford the corresponding crude mono-methyl dicarboxylic acid half-ester (quant.), which was used in the next reaction without further purification. According to General Procedure F, methyl 5-cyano-2-methyl-4-oxo-5-(tetrahydro-1-λ^4-thiophen-1-ylidene)pentanoate (730 mg, 60% over two steps) was obtained along with some impurities, following column chromatography (50 g KP-Sil; 50 mL/min; 100% ethyl acetate (7 CV), followed by a linear gradient (8 CV): 0%→100% acetone in ethyl acetate). According to General Procedure G, pure dimethyl dicarboxylate **S34** (300 mg, 56%) was obtained from methyl 5-cyano-2-methyl-4-oxo-5-(tetrahydro-1-λ^4-thiophen-1-ylidene)pentanoate (730 mg, 2.86 mmol), following column chromatography (25 g KP-Sil; 50 mL/min; 100% cyclohexane (3 CV), followed by a linear gradient (10 CV): 0%→25% ethyl acetate in cyclohexane). The analytical data are in agreement with those reported.[20]

Clear colorless oil; ^{1}H NMR (600 MHz, 300 K, $CDCl_3$): δ = 3.90 (s, 3H), 3.70 (s, 3H), 3.34 (dd, J = 18.6, 8.4 Hz, 1H), 3.07−3.01 (m, 1H), 2.90 (dd, J = 18.6, 5.3 Hz, 1H), 1.27 ppm (d, J = 7.2 Hz, 3H); ^{13}C NMR (150 MHz, 300 K, $CDCl_3$): δ = 192.0, 175.3, 161.0, 53.0, 52.1, 42.4, 34.6, 16.9 ppm; IR (film): ṽ = 2957, 1727, 1459, 1437, 1393, 1346, 1295, 1259, 1222, 1172, 1137, 1086, 1055 cm^{-1}; HRMS (ESI): m/z calculated for $C_8H_{13}O_5$ $[M+H]^+$: 189.0757, found: 189.0759.

4-Methyl-2-oxopentanedioic acid (26).

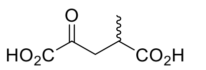

Dicarboxylic acid **26** (81 mg, quant.) was obtained from dimethyl 4-methyl-2-oxopentanedioate **S34** (94 mg, 0.5 mmol) according to General Procedure H. Minor amounts (~3%) of a byproduct are visible in ^{1}H NMR. To obtain a high quality ^{13}C NMR spectrum, extended measurement times were required due to signal broadening leading to the partial formation of the enol tautomer and keto-hydrate. Analytical data are shown below for the major 2-oxocarboxylic acid; the chemical shifts in the ^{13}C NMR spectrum are in agreement with those of the reported corresponding dilithium salt.[22]

White amorphous solid; ^{1}H NMR (600 MHz, 300 K, D_2O): δ = 3.07 (brdd, J = 17.3, 6.6 Hz, 1H), 2.81 (dd, J = 18.0, 6.3 Hz, 1H), 2.76−2.70 (m, 1H), 1.15 ppm (d, J = 7.1 Hz, 3H); ^{13}C NMR (150 MHz, 300 K, D_2O): δ = 205.1 (br), 184.6, 170.3, 43.7, 37.4, 17.3 ppm; IR (film): ṽ = 3366, 2977, 1709, 1567, 1463, 1414, 1351, 1307, 1276, 1142, 1097 cm^{-1}; HRMS (ESI): m/z calculated for $C_6H_7O_5$ $[M-H]^-$: 159.0299, found: 159.0293.

Methyl 5-cyano-2-ethyl-4-oxo-5-(tetrahydro-1-λ⁴-thiophen-1-ylidene)pentanoate (S35).

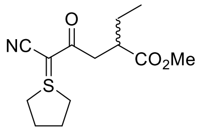

According to General Procedure D, 1-benzyl 4-methyl 3-ethylidenesuccinate (801 mg, 54%) was obtained from 4-benzyl 1-methyl 2-(diethoxyphosphoryl)succinate **S7**[11] (2.15 g, 6.0 mmol) and acetaldehyde, following column chromatography (50 g KP-Sil; 50 mL/min; 100% cyclohexane (3 CV), followed by a linear gradient (12 CV): 0%→15% ethyl acetate in cyclohexane). The clear colorless oil was a mixture of alkene diastereomers (1.5:1) which was used in the next reaction without further purification. ^{1}H NMR (400 MHz, 300 K, $CDCl_3$): major diastereomer: δ = 7.40−7.31 (m, 5H), 7.09 (q, J = 7.2 Hz, 1H), 5.16 (s, 2H), 3.72 (s, 3H), 3.44 (s, 2H), 1.84 ppm (d, J = 7.1 Hz, 3H); minor diastereomer: δ = 7.40−7.31 (m, 5H), 6.22 (qt, J = 7.3, 0.9 Hz, 1H), 5.15 (s, 2H), 3.68 (s, 3H), 3.33 (t, J = 0.9 Hz, 2H), 2.10 ppm (dt, J = 7.2, 0.9 Hz, 3H).

1-Benzyl 4-methyl 3-ethylidenesuccinate (801 mg, 3.2 mmol) was reduced according to General Procedure E to afford the corresponding crude mono-methyl dicarboxylic acid half-ester (quant.), which was used in the next reaction without further purification. According to General Procedure F, cyanosulfur ylid **S35** (723 mg, 84% over two steps) was obtained, following column chromatography (50 g KP-Sil; 50 mL/min; 100% ethyl acetate (7 CV), followed by a linear gradient (7 CV): 0%→100% acetone in ethyl acetate).

Clear pale orange oil; ^{1}H NMR (600 MHz, 300 K, $CDCl_3$): δ = 3.70 (s, 3H), 3.37−3.34 (m, 4H), 2.91 (dd, J = 15.8, 9.2 Hz, 1H), 2.84−2.80 (m, 1H), 2.65 (dd, J = 15.8, 4.9 Hz, 1H), 2.63−2.57 (m, 2H), 2.10−2.03 (m, 2H), 1.70−1.63 (m, 1H), 1.60−1.55 (m, 1H), 0.93 ppm (t, J = 7.4 Hz, 3H); ^{13}C NMR (150 MHz, 300 K, $CDCl_3$): δ = 189.7, 175.9, 120.6, 53.7, 51.5, 44.9, 44.8, 42.8, 40.2, 28.4, 28.3(9), 25.2, 11.6 ppm; IR (film): ṽ = 3474, 2964, 2877, 2168, 1730, 1585, 1437, 1373, 1302, 1268, 1212, 1157, 1090 cm^{-1}; HRMS (ESI): m/z calculated for $C_{13}H_{19}O_3NSNa$ $[M+Na]^+$: 292.0978, found: 292.0978.

Dimethyl 2-ethyl-4-oxopentanedioate (S36).

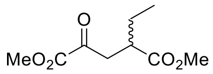

According to General Procedure G, dimethyl dicarboxylate **S36** (233 mg, 43%) was obtained from cyanosulfur ylid **S35** (723 mg, 2.68 mmol), following column chromatography (25 g Ultra; 50 mL/min; 100% cyclohexane (3 CV), followed by a linear gradient (15 CV): 0%→15% ethyl acetate in cyclohexane). The analytical data are in agreement with those reported.[22]

Clear colorless oil; ^{1}H NMR (600 MHz, 300 K, $CDCl_3$): δ = 3.90 (s, 3H), 3.71 (s, 3H), 3.33 (dd, J = 19.5, 10.4

Hz, 1H), 2.94−2.89 (m, 2H), 1.77−1.70 (m, 1H), 1.69−1.62 (m, 1H), 0.96 ppm (t, J = 7.5 Hz, 3H); ^{13}C NMR (150 MHz, 300 K, $CDCl_3$): δ = 192.3, 174.9, 161.0, 53.0, 51.9, 41.2, 40.3, 24.8, 11.3 ppm; IR (film): ṽ = 2969, 1731, 1458, 1437, 1395, 1263, 1215, 1170, 1129, 1061 cm^{-1}; HRMS (ESI): *m/z* calculated for $C_9H_{14}O_5Na$ [M+Na]$^+$: 225.0733, found: 225.0737.

2-Ethyl-4-oxopentanedioic acid (27).

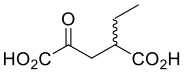

Dicarboxylic acid **27** (79 mg, 91%) was obtained from dimethyl 2-ethyl-4-oxopentanedioate **S36** (101 mg, 0.5 mmol) according to General Procedure H.

White amorphous solid; ^{1}H NMR (600 MHz, 300 K, D_2O): δ = 2.96 (brs, 1H), 2.87−2.84 (m, 1H), 2.67 (brs, 1H), 1.59−1.52 (m, 2H), 0.92 ppm (t, J = 7.4 Hz, 3H); ^{13}C NMR (150 MHz, 300 K, D_2O): δ = 204.9 (br), 183.3, 170.7 (br), 44.1 (br), 41.3 (br), 25.0, 11.0 ppm; IR (film): ṽ = 3371, 2967, 2936, 2878, 1711, 1628, 1570, 1411, 1353, 1274, 1247, 1225, 1213, 1135, 1104, 1073, 1025 cm^{-1}; HRMS (ESI): *m/z* calculated for $C_7H_9O_5$ [M−H]$^-$: 173.0445, found: 173.0452.

Methyl 5-cyano-4-oxo-2-propyl-5-(tetrahydro-1-λ^4-thiophen-1-ylidene)pentanoate (S37).

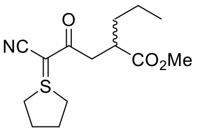

According to General Procedure D, 1-benzyl 4-methyl 3-propylidenesuccinate (958 mg, 61%) was obtained from 4-benzyl 1-methyl 2-(diethoxyphosphoryl)succinate **S7**[11] (2.15 g, 6.0 mmol) and propionaldehyde, following column chromatography (100 g KP-Sil; 60 mL/min; 100% cyclohexane (2 CV), followed by a linear gradient (10 CV): 0%→7% ethyl acetate in cyclohexane). The clear colorless oil was a mixture of alkene diastereomers (1.7:1) which was used in the next reaction without further purification. ^{1}H NMR (400 MHz, 300 K, $CDCl_3$): major diastereomer: δ = 7.40−7.31 (m, 5H), 6.98 (t, J = 7.5 Hz, 1H), 5.15 (s, 2H), 3.72 (s, 3H), 3.42 (s, 2H), 2.21 (apparent quint., J = 7.5 Hz, 2H), 1.07 ppm (t, J = 7.5 Hz, 3H); minor diastereomer: δ = 7.40−7.31 (m, 5H), 6.08 (tt, J = 7.4, 1.0 Hz, 1H), 5.15 (s, 2H), 3.67 (s, 3H), 3.33−3.32 (m, 2H), 2.59 (apparent quint., J = 7.5 Hz, 2H), 1.06 ppm (t, J = 7.6 Hz, 3H). 1-Benzyl 4-methyl 3-propylidenesuccinate (958 mg, 3.65 mmol) was reduced according to General Procedure E to afford the corresponding crude mono-methyl dicarboxylic acid half-ester (quant.), which was used in the next reaction without further purification. According to General Procedure F, cyanosulfur ylid **S37** (864 mg, 84% over two steps) was obtained, following column chromatography (50 g KP-Sil; 50 mL/min; 100% ethyl acetate (7 CV), followed by a linear gradient (7 CV): 0%→100% acetone in ethyl acetate).

Clear pale orange oil; ^{1}H NMR (600 MHz, 300 K, $CDCl_3$): δ = 3.70 (s, 3H), 3.37−3.32 (m, 4H), 2.93−2.86 (m, 2H), 2.65 (dd, J = 14.4, 3.2 Hz, 1H), 2.63−2.58 (m, 2H), 2.09−2.04 (m, 2H), 1.66−1.62 (m, 1H), 1.51−1.45 (m, 1H), 1.37−1.31 (m, 2H), 0.91 ppm (t, J = 7.3 Hz, 3H); ^{13}C NMR (150 MHz, 300 K, $CDCl_3$): δ = 189.7, 176.1, 120.6, 53.6, 51.5, 44.9, 44.8, 41.1, 40.6, 34.3, 28.4, 28.3(9), 20.3, 13.9 ppm; IR (film): ṽ = 3503, 2957, 2873, 2168, 1731, 1584, 1437, 1377, 1306, 1258, 1233, 1202, 1156, 1087 cm^{-1}; HRMS (ESI): *m/z* calculated for $C_{14}H_{21}O_3NSNa$ [M+Na]$^+$: 306.1134, found: 306.1136.

Dimethyl 2-oxo-4-propylpentanedioate (S38).

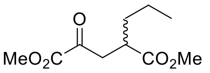

According to General Procedure G, dimethyl dicarboxylate **S38** (344 mg, 52%) was obtained from cyanosulfur ylid **S37** (864 mg, 3.05 mmol), following column chromatography (25 g Ultra; 50 mL/min; 100% cyclohexane (3 CV), followed by a linear gradient (15 CV): 0%→15% ethyl acetate in cyclohexane). The analytical data are in agreement with those reported.[22]

Clear colorless oil; ^{1}H NMR (600 MHz, 300 K, $CDCl_3$): δ = 3.89 (s, 3H), 3.70 (s, 3H), 3.31 (dd, J = 18.2, 9.0 Hz, 1H), 2.99−2.91 (m, 2H), 1.71−1.65 (m, 1H), 1.57−1.51 (m, 1H), 1.39−1.33 (m, 2H), 0.94 ppm (t, J = 7.3 Hz, 3H); ^{13}C NMR (150 MHz, 300 K, $CDCl_3$): δ = 192.3, 175.1, 161.0, 53.0, 51.9, 40.7, 39.7, 33.8, 20.1, 13.8 ppm; IR (film): ṽ = 2958, 2875, 1730, 1437, 1395, 1260, 1205, 1169, 1132, 1107, 1064, 971 cm^{-1}; HRMS (ESI): *m/z* calculated for $C_{10}H_{17}O_5$ [M+H]$^+$: 217.1071, found: 217.1073.

2-Oxo-4-propylpentanedioic acid (28).

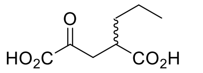

Dicarboxylic acid **28** (73 mg, 78%) was obtained from dimethyl 2-oxo-4-propylpentanedioate **S38** (108 mg, 0.5 mmol) according to General Procedure H.

White amorphous solid; ^{1}H NMR (600 MHz, 300 K, D_2O): δ = 2.92−2.78 (m, 3H), 1.59 (brs, 1H), 1.52−1.46 (m, 1H), 1.38−1.32 (m, 2H), 0.92 ppm (t, J = 7.4 Hz, 3H); ^{13}C NMR (150 MHz, 300 K, D_2O): δ = 183.2, 171.0 (br), 42.0 (br), 41.1 (br), 33.9 (br), 19.9, 13.2 ppm (the ketone ^{13}C-signal was not observed even after prolonged measurement times); IR (film): ṽ = 3455, 2965, 2928, 1757, 1630, 1577, 1407, 1264, 1237, 1209, 1024 cm^{-1}; HRMS (ESI): *m/z* calculated for $C_8H_{11}O_5$ [M−H]$^-$: 187.0601, found: 187.0608.

Methyl 5-cyano-2-isobutyl-4-oxo-5-(tetrahydro-1-λ^4-thiophen-1-ylidene)pentanoate (S39).

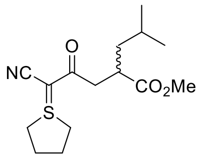

According to General Procedure D, 1-benzyl 4-methyl 3-(2-methylpropylidene)succinate (486 mg, 33%) was obtained from 4-benzyl 1-methyl 2-(diethoxyphosphoryl)succinate **S7**[11] (1.95 g, 5.4 mmol) and isobutyraldehyde, following column chromatography (50 g KP-Sil; 50 mL/min; 100% cyclohexane (2 CV), followed by a linear gradient (12 CV): 0%→12% ethyl acetate in cyclohexane). The clear colorless oil was a mixture of alkene diastereomers (1.4:1) which was used in the next reaction without further purification. Characteristic ^{1}H NMR signals (400 MHz, 300 K, $CDCl_3$): major diastereomer: δ = 5.86 ppm (dt, J = 9.8, 1.0 Hz, 1H); minor diastereomer: δ = 6.79 ppm (d, J = 10.2 Hz, 1H).

1-Benzyl 4-methyl 3-(2-methylpropylidene)succinate (486 mg, 1.76 mmol) was reduced according to General Procedure E to afford the corresponding crude mono-methyl dicarboxylic acid half-ester (quant.), which was used in the next reaction without further purification. According to General Procedure F, cyanosulfur ylid **S39** (260 mg, 50% over two steps) was obtained, following column chromatography (25 g KP-Sil; 50 mL/min; 100% ethyl acetate (7 CV), followed by a linear gradient (7 CV): 0%→100% acetone in ethyl acetate).

Clear pale yellow oil; ^{1}H NMR (600 MHz, 300 K, $CDCl_3$): δ = 3.70 (s, 3H), 3.37−3.31 (m, 4H), 2.96−2.92 (m, 1H), 2.89 (dd, J = 15.3, 9.5 Hz, 1H), 2.64 (dd, J = 15.5, 4.2 Hz, 1H), 2.64−2.57 (m, 2H), 2.09−2.03 (m, 2H), 1.63−1.56 (m, 2H), 1.33−1.27 (m, 1H), 0.93 (d, J = 6.2 Hz, 3H), 0.90 ppm (d, J = 6.2 Hz, 3H); ^{13}C NMR (150 MHz, 300 K, $CDCl_3$): δ = 189.7, 176.4, 120.6, 53.5, 51.6, 44.9, 44.8, 41.4, 41.1, 39.5, 28.4, 28.3(9), 25.9, 22.6, 22.3 ppm; IR (film): ṽ = 3487, 2955, 2871, 2168, 1731, 1587, 1438, 1370, 1309, 1270, 1234, 1157, 1087 cm^{-1}; HRMS (ESI): *m/z* calculated for $C_{15}H_{24}O_3NS$ [M+H]$^+$: 298.1471, found: 298.1472.

Dimethyl 2-isobutyl-4-oxopentanedioate (S40).

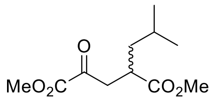

According to General Procedure G, dimethyl dicarboxylate **S40** (98 mg, 49%) was obtained from cyanosulfur ylid **S39** (260 mg, 0.87 mmol), following column chromatography (10 g Ultra; 35 mL/min; 100% cyclohexane (3 CV), followed by a linear gradient (15 CV): 0%→15% ethyl acetate in cyclohexane). The analytical data are in agreement with those reported.[20,22]

Clear colorless oil; ^{1}H NMR (600 MHz, 300 K, $CDCl_3$): δ = 3.89 (s, 3H), 3.70 (s, 3H), 3.31 (dd, J = 18.5, 9.4 Hz, 1H), 3.02−2.98 (m, 1H), 2.94 (dd, J = 18.5, 4.4 Hz, 1H), 1.65−1.60 (m, 2H), 1.38−1.33 (m, 1H), 0.96 (d, J = 5.9 Hz, 3H), 0.91 ppm (d, J = 6.0 Hz, 3H); ^{13}C NMR (150 MHz, 300 K, $CDCl_3$): δ = 192.3, 175.4, 161.0, 53.0, 51.9, 41.1, 40.9, 38.1, 25.8, 22.4, 22.3 ppm; IR (film): ṽ = 2957, 2873, 1732, 1438, 1388, 1261, 1205, 1169, 1142, 1069 cm^{-1}; HRMS (ESI): *m/z* calculated for $C_{11}H_{18}O_5Na$ [M+Na]$^+$: 253.1046, found: 253.1048.

2-Isobutyl-4-oxopentanedioic acid (29).

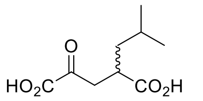

Dicarboxylic acid **29** (34 mg, 67%) was obtained from dimethyl 2-isobutyl-4-oxopentanedioate **S40** (57.6 mg, 0.25 mmol) according to General Procedure H.

White amorphous solid; ^{1}H NMR (600 MHz, 300 K, D_2O): δ = 2.84 (brs, 3H), 1.63−1.58 (m,

1H), 1.57−1.53 (m, 1H), 1.33−1.31 (m, 1H), 0.94 (d, *J* = 6.5 Hz, 3H), 0.90 ppm (d, *J* = 6.5 Hz, 3H); ^{13}C NMR (150 MHz, 300 K, D_2O): *δ* = 183.5, 171.0 (br), 41.9 (br), 41.1 (br), 40.8 (br), 25.6, 21.8 ppm (the ketone ^{13}C-signal was not observed even after prolonged measurement times); IR (film): ṽ = 3421, 2957, 2935, 1757, 1714, 1630, 1577, 1410, 1305, 1240, 1214, 1028 cm^{-1}; HRMS (ESI): *m/z* calculated for $C_9H_{13}O_5$ [M−H]$^-$: 201.0768, found: 201.0763.

Methyl 2-(3-cyano-2-oxo-3-(tetrahydro-1-λ^4-thiophen-1-ylidene)propyl)-5,5-dimethylhexanoate (S41).

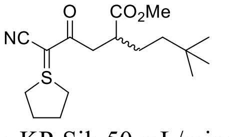

According to General Procedure D, 1-benzyl 4-methyl 3-(3,3-dimethylbutylidene)succinate (1.1 g, 52%) was obtained from 4-benzyl 1-methyl 2-(diethoxyphosphoryl)succinate **S7**[11] (2.5 g, 6.98 mmol) and 3,3-dimethylbutyraldehyde, following column chromatography (50 g KP-Sil; 50 mL/min; 100% cyclohexane (3 CV), followed by a linear gradient (10 CV): 0%→10% ethyl acetate in cyclohexane). The clear pale yellow oil was a mixture of alkene diastereomers (2.4:1) which was used in the next reaction without further purification. ^{1}H NMR (400 MHz, 300 K, $CDCl_3$): major diastereomer: *δ* = 7.39−7.31 (m, 5H), 7.08 (t, *J* = 7.9 Hz, 1H), 5.15 (s, 2H), 3.72 (s, 3H), 3.43 (s, 2H), 2.09 (d, *J* = 7.9 Hz, 2H), 0.95 ppm (s, 9H); minor diastereomer: *δ* = 7.39−7.31 (m, 5H), 6.16 (t, *J* = 7.7 Hz, 1H), 5.15 (s, 2H), 3.66 (s, 3H), 3.37−3.36 (m, 2H), 2.52 (d, *J* = 7.7 Hz, 2H), 0.94 ppm (s, 9H).

1-Benzyl 4-methyl 3-(3,3-dimethylbutylidene)succinate (1.1 g, 3.6 mmol) was reduced according to General Procedure E to afford the corresponding crude mono-methyl dicarboxylic acid half-ester (quant.), which was used in the next reaction without further purification. According to General Procedure F, cyanosulfur ylid **S41** (845 mg, 72% over two steps) was obtained, following column chromatography (50 g KP-Sil; 50 mL/min; 100% ethyl acetate (7 CV), followed by a linear gradient (7 CV): 0%→100% acetone in ethyl acetate).

Pale yellow solid, m.p.: 144−146 °C; ^{1}H NMR (600 MHz, 300 K, $CDCl_3$): *δ* = 3.70 (s, 3H), 3.37−3.33 (m, 4H), 2.92 (dd, *J* = 15.8, 9.4 Hz, 1H), 2.84−2.80 (m, 1H), 2.66 (dd, *J* = 15.9, 4.8 Hz, 1H), 2.64−2.58 (m, 2H), 2.10−2.03 (m, 2H), 1.64−1.59 (m, 1H), 1.53−1.47 (m, 1H), 1.23−1.15 (m, 2H), 0.87 ppm (s, 9H); ^{13}C NMR (150 MHz, 300 K, $CDCl_3$): *δ* = 189.7, 176.1, 120.6, 53.6, 51.6, 44.9, 44.8, 41.9, 41.1, 40.5, 30.2, 29.2, 28.4, 27.2 ppm; IR (film): ṽ = 3524, 2952, 2904, 2868, 2167, 1731, 1593, 1436, 1365, 1310, 1218, 1157, 1089 cm^{-1}; HRMS (ESI): *m/z* calculated for $C_{17}H_{28}O_3NS$ [M+H]$^+$: 326.1784, found: 326.1785.

Dimethyl 2-(3,3-dimethylbutyl)-4-oxopentanedioate (S42).

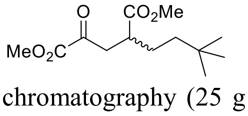

According to General Procedure G, dimethyl dicarboxylate **S42** (410 mg, 61%) was obtained from cyanosulfur ylid **S41** (845 mg, 2.6 mmol), following column chromatography (25 g Ultra; 50 mL/min; 100% cyclohexane (3 CV), followed by a linear gradient (12 CV): 0%→15% ethyl acetate in cyclohexane).

Clear colorless oil; ^{1}H NMR (600 MHz, 300 K, $CDCl_3$): *δ* = 3.90 (s, 3H), 3.72 (s, 3H), 3.33 (dd, *J* = 19.5, 10.4 Hz, 1H), 2.94−2.89 (m, 2H), 1.70−1.64 (m, 1H), 1.59−1.53 (m, 1H), 1.24−1.15 (m, 2H), 0.89 ppm (s, 9H); ^{13}C NMR (150 MHz, 300 K, $CDCl_3$): *δ* = 192.3, 175.1, 161.0, 53.0, 51.9, 40.9, 40.6, 40.4, 30.2, 29.2, 26.8 ppm; IR (film): ṽ = 2955, 2905, 2869, 1732, 1437, 1365, 1252, 1169, 1072 cm^{-1}; HRMS (ESI): *m/z* calculated for $C_{13}H_{22}O_5Na$ [M+Na]$^+$: 281.1359, found: 281.1361.

2-(3,3-Dimethylbutyl)-4-oxopentanedioic acid (30).

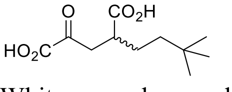

Dicarboxylic acid **30** (91 mg, 70%) was obtained from dimethyl 2-(3,3-dimethylbutyl)-4-oxopentanedioate **S42** (129 mg, 0.5 mmol) according to General Procedure H.

White amorphous solid; ^{1}H NMR (600 MHz, 300 K, D_2O): *δ* = 2.85 (brs, 2H), 2.73 (brs, 1H), 1.60 (brs, 1H), 1.56−1.50 (m, 1H), 1.26−1.18 (m, 2H), 0.89 ppm (s, 9H); ^{13}C NMR (150 MHz, 300 K, D_2O): *δ* = 182.9, 171.0 (br), 42.7 (br), 41.2 (br), 40.7, 29.2, 28.5, 26.8 (br) ppm (the ketone ^{13}C-signal was not observed even after prolonged measurement times); IR (film): ṽ = 3401, 2955, 1706, 1636, 1576, 1417, 1363, 1307, 1246, 1105, 1058,

1036 cm^{-1}; HRMS (ESI): *m/z* calculated for $C_{11}H_{17}O_5$ $[M-H]^-$: 229.1071, found: 229.1078.

Methyl 5-cyano-4-oxo-2-(3-phenylpropyl)-5-(tetrahydro-1-λ^4-thiophen-1-ylidene)pentanoate (S43).

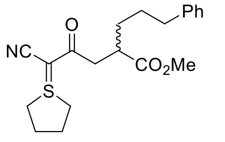

According to General Procedure D, 1-benzyl 4-methyl 3-(3-phenylpropylidene)succinate (1.17 g, 56%) was obtained from 4-benzyl 1-methyl 2-(diethoxyphosphoryl)succinate **S7**[11] (2.69 g, 7.5 mmol) and hydrocinnamaldehyde, following column chromatography (100 g KP-Sil; 60 mL/min; 100% cyclohexane (2 CV), followed by a linear gradient (10 CV): 0%→7% ethyl acetate in cyclohexane). The clear pale yellow oil was a mixture of alkene diastereomers (1.7:1) which was used in the next reaction without further purification. ^{1}H NMR (600 MHz, 300 K, $CDCl_3$): major diastereomer: δ = 7.39−7.29 (m, 6H), 7.23−7.16 (m, 4H), 7.03 (t, J = 7.5 Hz, 1H), 5.13 (s, 2H), 3.72 (s, 3H), 3.36 (s, 2H), 2.81−2.75 (m, 2H), 2.51 ppm (apparent q, J = 7.7 Hz, 2H); minor diastereomer: δ = 7.39−7.29 (m, 6H), 7.23−7.16 (m, 4H), 6.12 (t, J = 7.3 Hz, 1H), 5.15 (s, 2H), 3.66 (s, 3H), 3.32 (s, 2H), 2.91 (apparent q, J = 7.6 Hz, 2H), 2.81−2.75 ppm (m, 2H).

1-Benzyl 4-methyl 3-(3-phenylpropylidene)succinate (1.17 g, 4.18 mmol) was reduced according to General Procedure E to afford the corresponding crude mono-methyl dicarboxylic acid half-ester (quant.), which was used in the next reaction without further purification. According to General Procedure F, cyanosulfur ylid **S43** (1.14 g, 76% over two steps) was obtained, following column chromatography (50 g KP-Sil; 50 mL/min; 100% ethyl acetate (7 CV), followed by a linear gradient (7 CV): 0%→100% acetone in ethyl acetate).

Clear yellow/orange oil; ^{1}H NMR (600 MHz, 300 K, $CDCl_3$): δ = 7.29−7.27 (m, 2H), 7.20−7.17 (m, 3H), 3.69 (s, 3H), 3.33−3.28 (m, 4H), 2.93−2.88 (m, 2H), 2.68−2.57 (m, 5H), 2.09−2.02 (m, 2H), 1.72−1.61 (m, 3H), 1.56−1.54 ppm (m, 1H); ^{13}C NMR (150 MHz, 300 K, $CDCl_3$): δ = 189.5, 175.9, 142.1, 128.4, 128.3, 125.7, 120.5, 53.6, 51.6, 44.8 (2C), 41.2, 40.6, 35.6, 31.6, 28.9, 28.4, 28.3(9) ppm; IR (film): $\tilde{v}$ = 3510, 3025, 2946, 2166, 1730, 1595, 1496, 1453, 1373, 1297, 1256, 1227, 1156, 1085 cm^{-1}; HRMS (ESI): *m/z* calculated for $C_{20}H_{26}O_3NS$ $[M+H]^+$: 360.1628, found: 360.1627.

Dimethyl 2-oxo-4-(3-phenylpropyl)pentanedioate (S44).

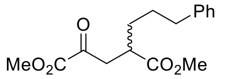

According to General Procedure G, dimethyl dicarboxylate **S44** (654 mg, 71%) was obtained from cyanosulfur ylid **S43** (1.14 g, 3.16 mmol), following column chromatography (50 g KP-Sil; 50 mL/min; 100% cyclohexane (3 CV), followed by a linear gradient (7 CV): 0%→17% ethyl acetate in cyclohexane).

Clear colorless oil; ^{1}H NMR (600 MHz, 300 K, $CDCl_3$): δ = 7.31−7.28 (m, 2H), 7.22−7.19 (m, 1H), 7.17 (d, J = 7.3 Hz, 2H), 3.89 (s, 3H), 3.69 (s, 3H), 3.32 (dd, J = 18.6, 9.3 Hz, 1H), 3.00−2.96 (m, 1H), 2.92 (dd, J = 18.6, 4.5 Hz, 1H), 2.64 (t, J = 7.4 Hz, 2H), 1.77−1.71 (m, 1H), 1.70−1.58 ppm (m, 3H); ^{13}C NMR (150 MHz, 300 K, $CDCl_3$): δ = 192.2, 174.9, 160.9, 141.7, 128.4, 128.3, 125.9, 53.0, 52.0, 40.8, 39.7, 35.5, 31.3, 28.7 ppm; IR (film): $\tilde{v}$ = 3027, 2952, 1729, 1603, 1496, 1454, 1437, 1392, 1259, 1168, 1068, 969 cm^{-1}; HRMS (ESI): *m/z* calculated for $C_{16}H_{20}O_5Na$ $[M+Na]^+$: 315.1203, found: 315.1200.

2-Oxo-4-(3-phenylpropyl)pentanedioic acid (31).

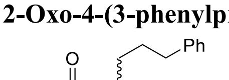

Dicarboxylic acid **31** (119 mg, 90%) was obtained from dimethyl 2-oxo-4-(3-phenylpropyl)pentanedioate **S44** (146 mg, 0.5 mmol) according to General Procedure H.

White solid, m.p.: >220 °C (decomposition); ^{1}H NMR (600 MHz, 300 K, D_2O): δ = 7.40−7.38 (m, 2H), 7.33−7.32 (m, 2H), 7.29−7.27 (m, 1H), 3.02 (dd, J = 18.2, 8.3 Hz, 1H), 2.85 (dd, J = 18.1, 5.9 Hz, 1H), 2.72−2.63 (m, 3H), 1.68−1.61 (m, 2H), 1.60−1.48 ppm (m, 2H); ^{13}C NMR (150 MHz, 300 K, D_2O): δ = 205.1 (br), 183.7, 170.2, 143.0, 128.6, 128.5(6), 125.9, 43.0, 42.3, 34.9, 31.8, 28.6 ppm; IR (film): $\tilde{v}$ = 3393, 2024, 2972, 2938, 1702, 1636, 1572, 1496, 1419, 1222, 1109, 1075, 1060 cm^{-1}; HRMS (ESI): *m/z* calculated for $C_{14}H_{15}O_5$ $[M-H]^-$: 263.0925, found: 263.0924.

Methyl 2-benzyl-5-cyano-4-oxo-5-(tetrahydro-1-λ^4-thiophen-1-ylidene)pentanoate (S45).

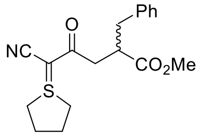

According to General Procedure D, 1-benzyl 4-methyl 3-benzylidenesuccinate (874 mg, 38%) was obtained as a single alkene diastereomer from 4-benzyl 1-methyl 2-(diethoxyphosphoryl)succinate **S7**[11] (2.69 g, 7.5 mmol) and benzaldehyde, following column chromatography (100 g KP-Sil; 60 mL/min; 100% cyclohexane (2 CV), followed by a linear gradient (10 CV): 0%→8% ethyl acetate in cyclohexane). ^{1}H NMR (400 MHz, 300 K, $CDCl_3$): δ = 7.93 (s, 1H), 7.41−7.32 (m, 10H), 5.20 (s, 2H), 3.79 (s, 3H), 3.61 ppm (s, 2H).

1-Benzyl 4-methyl 3-benzylidenesuccinate (983 mg, 3.17 mmol) was reduced according to General Procedure E to afford the corresponding crude mono-methyl dicarboxylic acid half-ester (quant.), which was used in the next reaction without further purification. According to General Procedure F, cyanosulfur ylid **S45** (1.03 g, 98% over two steps) was obtained, following column chromatography (50 g KP-Sil; 50 mL/min; 100% ethyl acetate (7 CV), followed by a linear gradient (7 CV): 0%→100% acetone in ethyl acetate).

Clear pale yellow oil; ^{1}H NMR (600 MHz, 300 K, $CDCl_3$): δ = 7.30−7.27 (m, 2H), 7.22−7.19 (m, 3H), 3.65 (s, 3H), 3.35−3.27 (m, 4H), 3.23−3.18 (m, 1H), 3.04 (dd, J = 13.6, 6.9 Hz, 1H), 2.92 (dd, J = 16.2, 8.9 Hz, 1H), 2.76 (dd, J = 13.6, 8.1 Hz, 1H), 2.64 (dd, J = 16.2, 5.1 Hz, 1H), 2.61−2.56 (m, 2H), 2.08−2.01 ppm (m, 2H); ^{13}C NMR (150 MHz, 300 K, $CDCl_3$): δ = 189.2, 175.3, 138.8, 129.1, 128.4, 126.4, 120.4, 53.7, 51.7, 44.8(4), 44.8, 43.2, 39.9, 38.0, 28.4, 28.3(7) ppm; HRMS (ESI): m/z calculated for $C_{18}H_{22}O_3NS$ $[M+H]^+$: 332.1315, found: 332.1315.

Dimethyl 2-benzyl-4-oxopentanedioate (S46).

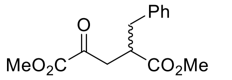

According to General Procedure G, dimethyl dicarboxylate **S46** (484 mg, 59%) was obtained from cyanosulfur ylid **S45** (1.03 g, 3.1 mmol), following column chromatography (50 g KP-Sil; 50 mL/min; 100% cyclohexane (3 CV), followed by a linear gradient (7 CV): 0%→15% ethyl acetate in cyclohexane). The analytical data are in agreement with those reported.[20,22]

Clear colorless oil; ^{1}H NMR (600 MHz, 300 K, $CDCl_3$): δ = 7.33−7.31 (m, 2H), 7.26−7.24 (m, 1H), 7.18 (d, J = 7.1 Hz, 2H), 3.86 (s, 3H), 3.69 (s, 3H), 3.30−3.23 (m, 2H), 3.14 (dd, J = 13.7, 5.4 Hz, 1H), 2.88−2.77 ppm (m, 2H); ^{13}C NMR (150 MHz, 300 K, $CDCl_3$): δ = 192.1, 174.3, 160.8, 137.9, 129.0, 128.7, 126.9, 53.0, 52.1, 41.9, 39.7, 37.5 ppm; IR (film): ṽ = 3029, 2954, 1730, 1604, 1497, 1455, 1437, 1258, 1227, 1195, 1170, 1095, 1065, 970 cm^{-1}; HRMS (ESI): m/z calculated for $C_{14}H_{16}O_5Na$ $[M+Na]^+$: 287.0890, found: 287.0891.

2-Benzyl-4-oxopentanedioic acid (32).

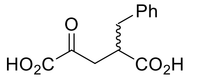

Dicarboxylic acid **32** (99 mg, 84%) was obtained from dimethyl 2-benzyl-4-oxopentanedioate **S46** (132 mg, 0.5 mmol) according to General Procedure H.

White solid, m.p.: >240 °C (decomposition); ^{1}H NMR (600 MHz, 300 K, D_2O): δ = 7.40−7.37 (m, 2H), 7.31−7.29 (m, 3H), 3.05 (dd, J = 18.0, 7.9 Hz, 1H), 2.99−2.91 (m, 2H), 2.86 (dd, J = 18.0, 5.2 Hz, 1H), 2.77 ppm (dd, J = 12.9, 6.7 Hz, 1H); ^{13}C NMR (150 MHz, 300 K, D_2O): δ = 204.6, 182.7, 169.8, 139.8, 129.1, 128.5, 126.4, 45.2, 41.6, 38.2 ppm; IR (film): ṽ = 3388, 2981, 1702, 1635, 1568, 1497, 1417, 1340, 1286, 1189, 1105 cm^{-1}; HRMS (ESI): m/z calculated for $C_{12}H_{11}O_5$ $[M-H]^-$: 235.0612, found: 235.0611.

Methyl 5-cyano-2-(naphthalen-2-ylmethyl)-4-oxo-5-(tetrahydro-1-λ^4-thiophen-1-ylidene)pentanoate (S47).

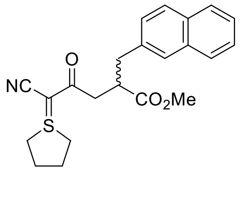

According to General Procedure D, 1-benzyl 4-methyl 3-(naphthalen-2-ylmethylene)succinate (962 mg, 36%) was obtained as a single alkene diastereomer from 4-benzyl 1-methyl 2-(diethoxyphosphoryl)succinate **S7**[11] (2.69 g, 7.5 mmol) and 2-naphthaldehyde, following column chromatography (100 g KP-Sil; 60 mL/min; 100% cyclohexane (2 CV), followed by a linear gradient (10 CV): 0%→13% ethyl acetate in cyclohexane). ^{1}H NMR (400 MHz, 300 K, $CDCl_3$): δ = 8.08 (s, 1H), 7.86−7.81 (m, 3H), 7.75−7.73 (m, 1H), 7.55−7.48 (m, 2H), 7.44−7.34 (m, 6H), 5.23 (s, 2H), 3.82 (s, 3H), 3.69 ppm (s, 2H).

1-Benzyl 4-methyl 3-(naphthalen-2-ylmethylene)succinate (1.17 g, 3.26 mmol) was reduced according to General Procedure E (twice submitted to reaction conditions to achieve full conversion) to afford the corresponding crude mono-methyl dicarboxylic acid half-ester (quant.), which was used in the next reaction without further purification. According to General Procedure F, cyanosulfur ylid **S47** (800 mg, 64% over two steps) was obtained, following column chromatography (50 g KP-Sil; 50 mL/min; 100% ethyl acetate (7 CV), followed by a linear gradient (7 CV): 0%→100% acetone in ethyl acetate).

Clear pale orange oil; ^{1}H NMR (600 MHz, 300 K, $CDCl_3$): δ = 7.82−7.80 (m, 2H), 7.78 (d, J = 8.4 Hz, 1H), 7.68 (s, 1H), 7.48−7.42 (m, 2H), 7.36 (dd, J = 8.4, 1.7 Hz, 1H), 3.65 (s, 3H), 3.38−3.30 (m, 1H), 3.25−3.16 (m, 4H), 3.14−3.09 (m, 1H), 2.95 (dd, J = 16.1, 8.1 Hz, 1H), 2.91 (dd, J = 13.5, 8.4 Hz, 1H), 2.71 (dd, J = 16.1, 5.6 Hz, 1H), 2.55−2.47 (m, 2H), 2.02−1.95 ppm (m, 2H); ^{13}C NMR (150 MHz, 300 K, $CDCl_3$): δ = 189.1, 175.3, 136.3, 133.5, 132.3, 128.0, 127.7, 127.6, 127.5 (2C), 125.9, 125.4, 120.4, 53.9, 51.7, 44.8, 44.6, 43.0, 40.0, 38.3, 28.3 (2C) ppm; IR (film): ṽ = 3535, 3051, 2950, 2166, 1731, 1597, 1508, 1436, 1372, 1270, 1228, 1155, 1088, 1037 cm^{-1}; HRMS (ESI): *m/z* calculated for $C_{22}H_{24}O_3NS$ $[M+H]^+$: 382.1471, found: 382.1471.

Dimethyl 2-(naphthalen-2-ylmethyl)-4-oxopentanedioate (S48).

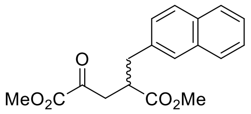

According to General Procedure G, dimethyl dicarboxylate **S48** (411 mg, 62%) was obtained from cyanosulfur ylid **S47** (800 mg, 2.1 mmol), following column chromatography (25 g KP-Sil; 45 mL/min; 100% cyclohexane (3 CV), followed by a linear gradient (8 CV): 0%→17% ethyl acetate in cyclohexane).

Clear colorless oil; ^{1}H NMR (600 MHz, 300 K, $CDCl_3$): δ = 7.84−7.80 (m, 3H), 7.63 (s, 1H), 7.51−7.46 (m, 2H), 7.32 (dd, J = 8.4, 1.6 Hz, 1H), 3.82 (s, 3H), 3.70 (s, 3H), 3.39−3.34 (m, 1H), 3.33−3.28 (m, 2H), 2.95 (dd, J = 13.6, 8.8 Hz, 1H), 2.89 ppm (dd, J = 18.3, 4.0 Hz, 1H); ^{13}C NMR (150 MHz, 300 K, $CDCl_3$): δ = 192.1, 174.3, 160.8, 135.4, 133.5, 132.4, 128.5, 127.7 (2C), 127.5, 127.0, 126.2, 125.7, 53.0, 52.1, 41.9, 39.8, 37.7 ppm; IR (film): ṽ = 3052, 2954, 1731, 1601, 1509, 1437, 1374, 1272, 1229, 1170, 1092, 1066, 968 cm^{-1}; HRMS (ESI): *m/z* calculated for $C_{18}H_{18}O_5Na$ $[M+Na]^+$: 337.1046, found: 337.1043.

2-(Naphthalen-2-ylmethyl)-4-oxopentanedioic acid (33).

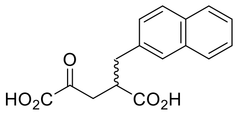

Dicarboxylic acid **33** (139 mg, 97%) was obtained from dimethyl 2-(naphthalen-2-ylmethyl)-4-oxopentanedioate **S48** (157 mg, 0.5 mmol) according to General Procedure H.

White solid, m.p.: >220 °C (decomposition); ^{1}H NMR (600 MHz, 300 K, D_2O): δ = 7.95−7.91 (m, 3H), 7.79 (s, 1H), 7.59−7.54 (m, 2H), 7.49 (d, J = 8.3 Hz, 1H), 3.14−3.07 (m, 3H), 2.96 (dd, J = 12.3, 5.9 Hz, 1H), 2.92−2.87 ppm (m, 1H); ^{13}C NMR (150 MHz, 300 K, D_2O): δ = 204.5 (br), 182.6, 169.8, 137.6, 133.2, 131.8, 127.9, 127.8, 127.6, 127.5, 127.2, 126.3, 125.7, 45.1, 41.6, 38.3 ppm; IR (film): ṽ = 3370, 3056, 3046, 2981, 1702, 1636, 1569, 1418, 1349, 1291, 1101 cm^{-1}; HRMS (ESI): *m/z* calculated for $C_{16}H_{13}O_5$ $[M-H]^-$: 285.0757, found: 285.0769.

Methyl 5-cyano-2,2-dimethyl-4-oxo-5-(tetrahydro-1-λ^4-thiophen-1-ylidene)pentanoate (S49).

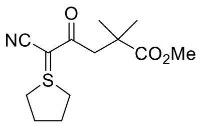

According to General Procedure F, cyanosulfur ylid **S49** (815 mg, 50%) was obtained from 4-methoxy-3,3-dimethyl-4-oxobutanoic acid (960 mg, 6.0 mmol), following column chromatography (50 g KP-Sil; 40 mL/min; 100% ethyl acetate (7 CV), followed by a linear gradient (7 CV): 0%→100% acetone in ethyl acetate).

White solid, m.p.: 90−92 °C; ^{1}H NMR (600 MHz, 300 K, $CDCl_3$): δ = 3.70 (s, 2H), 3.38−3.31 (m, 4H), 2.81 (s, 2H), 2.63−2.56 (m, 2H), 2.10−2.03 (m, 2H), 1.25 ppm (s, 6H); ^{13}C NMR (150 MHz, 300 K, $CDCl_3$): δ = 189.2, 178.0, 120.8, 54.6, 51.8, 48.4, 44.9, 40.9, 28.4, 25.4 ppm; IR (film): ṽ = 3531, 2972, 2950, 2874, 2164, 1725, 1590, 1473, 1433, 1364, 1306, 1277, 1251, 1199, 1160, 1027 cm^{-1}; HRMS (ESI): *m/z* calculated for $C_{13}H_{19}O_3NSNa$ $[M+Na]^+$: 292.0978, found: 292.0977.

Dimethyl 2,2-dimethyl-4-oxopentanedioate (S50).

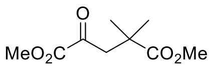

According to General Procedure G, dimethyl dicarboxylate **S50** (426 mg, 70%) was obtained from cyanosulfur ylid **S49** (815 mg, 3.03 mmol), following column chromatography (25 g KP-Sil; 45 mL/min; 100% cyclohexane (3 CV), followed by a linear gradient (8 CV): 0%→20% ethyl acetate in cyclohexane). The analytical data are in agreement with those reported.[23]

Clear colorless oil; ^{1}H NMR (600 MHz, 300 K, $CDCl_3$): δ = 3.88 (s, 3H), 3.68 (s, 3H), 3.13 (s, 2H), 1.31 ppm (s, 6H); ^{13}C NMR (150 MHz, 300 K, $CDCl_3$): δ = 191.8, 177.0, 161.2, 53.0, 52.1, 48.1, 40.6, 25.5 ppm; IR (film): $\tilde{\nu}$ = 2979, 2956, 1731, 1475, 1437, 1388, 1367, 1301, 1280, 1201, 1154, 1129, 1062, 977 cm^{-1}; HRMS (ESI): *m/z* calculated for $C_9H_{14}O_5Na$ [M+Na]$^+$: 225.0733, found: 225.0733.

2,2-Dimethyl-4-oxopentanedioic acid (34).

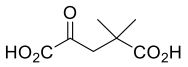

Dicarboxylic acid **34** (169 mg, 97%) was obtained from dimethyl 2,2-dimethyl-4-oxopentanedioate **S50** (202 mg, 1.0 mmol) according to General Procedure H. The analytical data are in the range of those reported for the corresponding dilithium salt.[23]

White amorphous solid; ^{1}H NMR (600 MHz, 300 K, D_2O): δ = 2.93 (brs, 2H), 1.20 ppm (s, 6H); ^{13}C NMR (150 MHz, 300 K, D_2O): δ = 204.7 (br), 186.1, 171.3 (br), 49.0 (br), 41.3, 25.5 ppm; IR (film): $\tilde{\nu}$ = 3391, 2980, 1761, 1699, 1636, 1570, 1475, 1418, 1376, 1338, 1268, 1226, 1178, 1077 cm^{-1}; HRMS (ESI): *m/z* calculated for $C_7H_9O_5$ [M−H]$^-$: 173.0456, found: 173.0453.

***cis*-Methyl 3-(2-cyano-2-(tetrahydro-1-λ^4-thiophen-1-ylidene)acetyl)-2,2-dimethylcyclopropane-1-carboxylate (S51).**

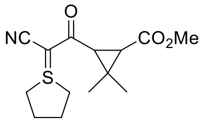

According to General Procedure A, *cis*-3-(methoxycarbonyl)-2,2-dimethylcyclopropane-1-carboxylic acid **13a** (3.08 g, 89%) was obtained from 6,6-dimethyl-3-oxabicyclo[3.1.0]hexane-2,4-dione (2.80 g, 20 mmol) and used in the next reaction without further purification. Characteristic NMR signals: ^{1}H NMR (600 MHz, 300 K, CD_3OD): δ = 3.65 (s, 3H), 1.95 (d, J = 9.4 Hz, 1H), 1.93−1.91 (m, 1H), 1.38 (s, 3H), 1.21 ppm (s, 3H); ^{13}C NMR (150 MHz, 300 K, CD_3OD): δ = 172.7, 171.6, 52.1, 33.1, 33.0, 28.0, 26.9, 15.9 ppm.

According to General Procedure F, cyanosulfur ylid **S51** (2.50 g, 50%) was obtained as a single racemic *cis*-diastereomer from 3-(methoxycarbonyl)-2,2-dimethylcyclopropane-1-carboxylic acid (3.08 g, 17.9 mmol), following column chromatography (100 g KP-Sil; 50 mL/min; 100% ethyl acetate (8 CV), followed by a linear gradient (7 CV): 0%→100% acetone in ethyl acetate).

Gray solid, m.p.: 92−94 °C; ^{1}H NMR (600 MHz, 300 K, $CDCl_3$): δ = 3.70 (s, 3H), 3.44−3.39 (m, 2H), 3.35−3.30 (m, 2H), 2.66−2.58 (m, 2H), 2.21 (d, J = 9.3 Hz, 1H), 2.08−2.01 (m, 2H), 1.87 (d, J = 9.3 Hz, 1H), 1.40 (s, 3H), 1.25 ppm (s, 3H); ^{13}C NMR (150 MHz, 300 K, $CDCl_3$): δ = 186.4, 170.6, 120.9, 53.8, 51.4, 44.6, 44.4, 37.2, 31.7, 28.4, 28.3(9), 28.1, 26.6, 15.5 ppm; IR (film): $\tilde{\nu}$ = 3496, 3003, 2952, 2874, 2166, 1728, 1586, 1438, 1418, 1375, 1286, 1220, 1183, 1157, 1120 cm^{-1}; HRMS (ESI): *m/z* calculated for $C_{14}H_{19}O_3NSNa$ [M+Na]$^+$: 304.0978, found: 304.0977.

***cis*-Methyl 3-(2-methoxy-2-oxoacetyl)-2,2-dimethylcyclopropane-1-carboxylate (S52).**

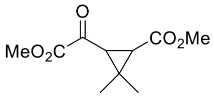

According to General Procedure G, dimethyl dicarboxylate **S52** (1.38 g, 72%) was obtained as a single racemic *cis*-diastereomer from cyanosulfur ylid **S51** (2.5 g, 8.89 mmol), following column chromatography (50 g KP-Sil; 50 mL/min; 100% cyclohexane (3 CV), followed by a linear gradient (6 CV): 0%→20% ethyl acetate in cyclohexane).

Clear pale yellow oil; ^{1}H NMR (600 MHz, 300 K, $CDCl_3$): δ = 3.88 (s, 3H), 3.69 (s, 3H), 2.62 (d, J = 9.1 Hz, 1H), 2.19 (d, J = 9.1 Hz, 1H), 1.34 (s, 3H), 1.33 ppm (s, 3H); ^{13}C NMR (150 MHz, 300 K, $CDCl_3$): δ = 188.7, 169.5, 161.1, 53.0, 52.0, 35.3, 35.1, 29.7, 27.8, 15.2 ppm; IR (film): $\tilde{\nu}$ = 2958, 1732, 1440, 1416, 1362, 1282, 1228, 1180,

1116, 1060, 1023 cm^{-1}; HRMS (ESI): *m/z* calculated for $C_{10}H_{14}O_5Na$ $[M+Na]^+$: 237.0733, found: 237.0734.

3-(Carboxycarbonyl)-2,2-dimethylcyclopropane-1-carboxylic acid (35).

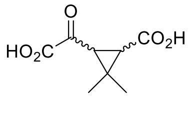

Dicarboxylic acid **35** (130 mg, 70%) was obtained from methyl 3-(2-methoxy-2-oxoacetyl)-2,2-dimethylcyclopropane-1-carboxylate **S52** (214 mg, 1.0 mmol) according to General Procedure H. Epimerization occurred during the reaction and the product was isolated as was a mixture of racemic diastereomers (2.5:1, *cis*:*trans*) which was used without further purification in biochemical assays.

White amorphous solid; ^{1}H NMR (600 MHz, 300 K, D_2O): major diastereomer: δ = 2.54 (d, J = 8.9 Hz, 1H), 2.24 (d, J = 8.9 Hz, 1H), 1.29 (s, 3H), 1.26 ppm (s, 3H); minor diastereomer: δ = 2.79 (d, J = 6.0 Hz, 1H), 2.37 (d, J = 6.0 Hz, 1H), 1.33 (s, 3H), 1.19 ppm (s, 3H); ^{13}C NMR (150 MHz, 300 K, D_2O): major diastereomer: δ = 202.1 (br), 176.6, 171.0, 41.1, 35.6, 31.6, 27.3, 14.7 ppm; minor diastereomer: δ = 203.2, 177.5, 171.2, 40.1, 38.3, 34.3, 20.8, 19.1 ppm; HRMS (ESI): *m/z* calculated for $C_8H_9O_5$ $[M-H]^-$: 185.0456, found: 185.0454.

(±)-(*2-exo,3-exo*)-Methyl 3-(2-cyano-2-(tetrahydro-1-λ^4-thiophen-1-ylidene)acetyl)bicyclo[2.2.1]hept-5-ene-2-carboxylate (S53).

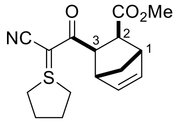

According to General Procedure F, cyanosulfur ylid **S53** (0.99 g, 15%) was obtained as a single racemic (*2-exo,3-exo*)-diastereomer from 3-(methoxycarbonyl)bicyclo[2.2.1]hept-5-ene-2-carboxylic acid (4.19 g, 21.4 mmol), following column chromatography (100 g KP-Sil; 50 mL/min; 100% ethyl acetate (7 CV), followed by a linear gradient (7 CV): 0%→100% acetone in ethyl acetate). The stereochemistry of **S53** was tentatively assigned based on **S54**.

White solid, m.p.: 127−128 °C; ^{1}H NMR (600 MHz, 300 K, $CDCl_3$): δ = 6.38 (dd, J = 5.6, 3.0 Hz, 1H), 6.13 (dd, J = 5.5, 3.0 Hz, 1H), 3.80 (dd, J = 9.9, 3.4 Hz, 1H), 3.58 (s, 3H), 3.46 (dt, J = 11.9, 7.7 Hz, 1H), 3.41−3.37 (m, 1H), 3.29−3.25 (m, 3H), 3.23−3.21 (m, 1H), 3.16−3.14 (m, 1H), 2.68−2.57 (m, 2H), 2.07−1.95 (m, 2H), 1.44 (dt, J = 8.5, 1.8 Hz, 1H), 1.38 ppm (d, J = 8.5 Hz, 1H); ^{13}C NMR (150 MHz, 300 K, $CDCl_3$): δ = 190.5, 173.7, 135.3, 134.0, 121.5, 51.6, 51.5, 51.1, 48.8, 48.6, 47.3, 46.3, 43.6, 43.4, 28.4, 28.3 ppm; IR (film): ṽ = 3504, 2978, 2948, 2872, 2163, 1731, 1601, 1434, 1377, 1350, 1335, 1265, 1184, 1157, 1070, 1043 cm^{-1}; HRMS (ESI): *m/z* calculated for $C_{16}H_{19}O_3NSNa$ $[M+Na]^+$: 328.0980, found: 328.0979.

(±)-(*2-exo,3-exo*)-Methyl 3-(2-cyano-2-(tetrahydro-1-λ^4-thiophen-1-ylidene)acetyl)bicyclo[2.2.1]hept-5-ene-2-carboxylate (S54).

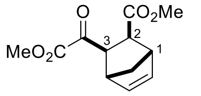

According to General Procedure G, dimethyl dicarboxylate **S54** (654 mg, 66%) was obtained as a single racemic diastereomer from cyanosulfur ylid **S53** (1.27 g, 4.16 mmol), following column chromatography (50 g KP-Sil; 50 mL/min; 100% cyclohexane (3 CV), followed by a linear gradient (8 CV): 0%→20% ethyl acetate in cyclohexane). Analysis of the $^3J_{HH}$ coupling constants of C_2-H and C_3-H in combination with nOe NMR experiments indicates the exclusive presence of **S54** as the (*2-exo,3-exo*)-diastereomer.

White solid, m.p.: 42−44 °C; ^{1}H NMR (600 MHz, 300 K, $CDCl_3$): δ = 6.51 (dd, J = 5.6, 3.0 Hz, 1H), 5.95 (dd, J = 5.6, 2.9 Hz, 1H), 3.83 (s, 3H), 3.77 (dd, J = 9.7, 3.8 Hz, 1H), 3.72 (dd, J = 9.7, 2.9 Hz, 1H), 3.60 (s, 3H), 3.27 (s, 1H), 3.21−3.19 (m, 1H), 1.53 (dt, J = 8.7, 1.8 Hz, 1H), 1.42 ppm (d, J = 8.7 Hz, 1H); ^{13}C NMR (150 MHz, 300 K, $CDCl_3$): δ = 192.0, 173.5, 161.8, 137.4, 132.9, 52.7, 52.1, 51.6, 50.1, 48.1, 47.3, 44.8 ppm; IR (film): ṽ = 2985, 2954, 1727, 1438, 1339, 1275, 1248, 1204, 1143, 1078, 988 cm^{-1}; HRMS (ESI): *m/z* calculated for $C_{12}H_{14}O_5Na$ $[M+Na]^+$: 261.0733, found: 261.0732.

(±)-(*2-exo,3-endo*)-3-(Carboxycarbonyl)bicyclo[2.2.1]hept-5-ene-2-carboxylic acid (36).

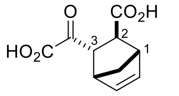

Dicarboxylic acid **36** (96 mg, 91%) was obtained as a single racemic diastereomer from methyl 3-(2-cyano-2-(tetrahydro-1-λ^4-thiophen-1-ylidene)acetyl)bicyclo[2.2.1]hept-5-ene-2-carboxylate **S54** (119 mg, 0.5 mmol) according to General Procedure H. Analysis of the $^3J_{HH}$ coupling constants of C_2-H and C_3-H in combination with nOe NMR experiments indicates the exclusive presence of **36** as the (*2-exo,3-endo*)-diastereomer.

Pale orange amorphous solid; ^{1}H NMR (600 MHz, 300 K, D_2O): δ = 6.40 (dd, J = 5.5, 3.2 Hz, 1H), 6.20 (dd, J = 5.6, 2.8 Hz, 1H), 3.35 (t, J = 4.2 Hz, 1H), 3.28 (s, 1H), 3.19 (d, J = 1.2 Hz, 1H), 3.16 (dd, J = 4.7, 1.5 Hz, 1H), 1.45 (dd, J = 8.9, 1.6 Hz, 1H), 1.37 ppm (d, J = 8.9 Hz, 1H); ^{13}C NMR (150 MHz, 300 K, D_2O): δ = 206.3, 180.1, 171.3, 137.4, 135.6, 52.2, 47.4, 46.7, 46.4, 46.0 ppm; IR (film): ṽ = 3405, 3065, 2981, 1699, 1684, 1636, 1570, 1418, 1333, 1268, 1235, 1094 cm^{-1}; HRMS (ESI): *m/z* calculated for $C_{10}H_9O_5$ [M−H]$^-$: 209.0444, found: 209.0451.

Methyl 4-(2-cyano-2-(tetrahydro-1-λ^4-thiophen-1-ylidene)acetyl)thiophene-3-carboxylate (S55).

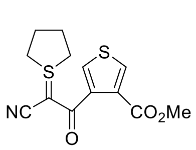

According to General Procedure A, 4-(methoxycarbonyl)thiophene-3-carboxylic acid **13b** (4.23 g, 95%) was obtained from 1*H*,3*H*-thieno[3,4-c]furan-1,3-dione[24] (3.70 g, 24 mmol) and used without further purification for the synthesis of cyanosulfur ylid **S55**. ^{1}H NMR (600 MHz, 300 K, CD_3OD): δ = 8.14 (d, J = 3.3 Hz, 1H), 8.10 (d, J = 3.3 Hz, 1H), 3.88 ppm (s, 3H).

According to General Procedure F, cyanosulfur ylid **S55** (2.56 g, 38%) was obtained from 4-(methoxycarbonyl)thiophene-3-carboxylic acid **13b** (4.23 g, 22.6 mmol), following column chromatography (100 g KP-Sil; 50 mL/min; 100% ethyl acetate (7 CV), followed by a linear gradient (7 CV): 0%→100% acetone in ethyl acetate).

Yellow solid, m.p.: 158−160 °C; ^{1}H NMR (600 MHz, 300 K, $CDCl_3$): δ = 8.02 (d, J = 3.2 Hz, 1H), 7.47 (d, J = 3.2 Hz, 1H), 3.85 (s, 3H), 3.65−3.59 (m, 2H), 3.48−3.43 (m, 2H), 2.72−2.66 (m, 2H), 2.15−2.08 ppm (m, 2H); ^{13}C NMR (150 MHz, 300 K, $CDCl_3$): δ = 182.7, 162.5, 141.7, 133.4, 131.6, 126.1, 120.3, 55.5, 51.8, 44.3, 28.6 ppm; IR (film): ṽ = 3474, 3101, 3000, 2952, 2170, 1715, 1566, 1511, 1454, 1425, 1328, 1251, 1188, 1118, 1040, 954 cm^{-1}; HRMS (ESI): *m/z* calculated for $C_{13}H_{13}O_3NS_2Na$ [M+Na]$^+$: 318.0229, found: 318.0230.

Methyl 4-(2-methoxy-2-oxoacetyl)thiophene-3-carboxylate (S56).

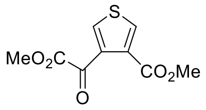

According to General Procedure G, dimethyl dicarboxylate **S56** (1.60 g, 81%) was obtained from cyanosulfur ylid **S55** (2.56 g, 8.7 mmol), following column chromatography (50 g KP-Sil; 50 mL/min; 100% cyclohexane (3 CV), followed by a linear gradient (6 CV): 0%→25% ethyl acetate in cyclohexane).

White solid, m.p.: 65−67 °C; ^{1}H NMR (600 MHz, 300 K, $CDCl_3$): δ = 8.08 (d, J = 3.3 Hz, 1H), 8.02 (d, J = 3.3 Hz, 1H), 3.92 (s, 3H), 3.86 ppm (s, 3H); ^{13}C NMR (150 MHz, 300 K, $CDCl_3$): δ = 181.5, 162.7, 162.1, 138.3, 134.2, 133.7, 132.6, 53.0, 52.3 ppm; IR (film): ṽ = 3109, 3004, 2956, 1710, 1511, 1455, 1399, 1312, 1259, 1195, 1170, 1090, 1003 cm^{-1}; HRMS (ESI): *m/z* calculated for $C_9H_9O_5SNa$ [M+Na]$^+$: 250.9996, found: 250.9986.

4-(Carboxycarbonyl)thiophene-3-carboxylic acid (37).

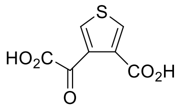

Dicarboxylic acid **37** (200 mg, quant.) was obtained from methyl 4-(2-methoxy-2-oxoacetyl)thiophene-3-carboxylate **S56** (228 mg, 1.0 mmol) according to General Procedure H.

White amorphous solid; ^{1}H NMR (600 MHz, 300 K, D_2O): δ = 8.17 (d, J = 3.0 Hz, 1H), 7.57−7.56 ppm (m, 1H); ^{13}C NMR (150 MHz, 300 K, D_2O): δ = 190.5, 173.1, 170.9, 140.6, 136.8, 136.0, 126.2 ppm; IR (film): ṽ = 3348, 3103, 2981, 1623, 1573, 1509, 1455, 1395, 1353, 1202, 1109 cm^{-1}; HRMS (ESI): *m/z* calculated for $C_7H_3O_5S$

[M−H]⁻: 198.9707, found: 198.9706.

Methyl 5-(2-cyano-2-(tetrahydro-1-λ^4-thiophen-1-ylidene)acetyl)thiophene-2-carboxylate (S57).

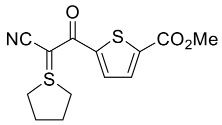

According to General Procedure F, cyanosulfur ylid **S57** (1.40 g, 87%) was obtained from 5-(methoxycarbonyl)thiophene-2-carboxylic acid (1.0 g, 5.5 mmol), following column chromatography (50 g KP-Sil; 45 mL/min; 100% ethyl acetate (7 CV), followed by a linear gradient (7 CV): 0%→100% acetone in ethyl acetate).

Yellow solid, m.p.: 143−145 °C; ^{1}H NMR (600 MHz, 300 K, $CDCl_3$): δ = 7.90 (d, J = 4.0 Hz, 1H), 7.72 (d, J = 4.1 Hz, 1H), 3.90 (s, 3H), 3.54−3.45 (m, 4H), 2.68−2.61 (m, 2H), 2.18−2.11 ppm (m, 2H); ^{13}C NMR (150 MHz, 300 K, $CDCl_3$): δ = 175.7, 162.4, 149.2, 136.6, 133.4, 129.4, 120.2, 55.2, 52.3, 44.8, 28.5 ppm; IR (film): ṽ = 3498, 2951, 2165, 1711, 1546, 1523, 1452, 1431, 1337, 1283, 1257, 1187, 1100, 1073, 1033 cm^{-1}; HRMS (ESI): *m/z* calculated for $C_{13}H_{13}O_3NS_2$ $[M+Na]^+$: 318.0229, found: 318.0229.

Methyl 5-(2-methoxy-2-oxoacetyl)thiophene-2-carboxylate (S58).

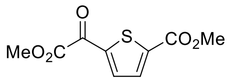

According to General Procedure G, dimethyl dicarboxylate **S58** (644 mg, 73%) was obtained from cyanosulfur ylid **S57** (1.11 g, 3.9 mmol), following column chromatography (50 g KP-Sil; 50 mL/min; 100% cyclohexane (3 CV), followed by a linear gradient (8 CV): 0%→20% ethyl acetate in cyclohexane).

Pale yellow solid, m.p.: 121−123 °C; ^{1}H NMR (600 MHz, 300 K, $CDCl_3$): δ = 8.10 (d, J = 4.0 Hz, 1H), 7.81 (d, J = 4.1 Hz, 1H), 4.01 (s, 3H), 3.96 ppm (s, 3H); ^{13}C NMR (150 MHz, 300 K, $CDCl_3$): δ = 175.9, 161.8, 161.2, 142.9, 142.4, 136.5, 133.3, 53.5, 52.8 ppm; IR (film): ṽ = 3098, 2966, 1741, 1726, 1682, 1516, 1442, 1428, 1363, 1319, 1257, 1241, 1223, 1203, 1183, 1103, 1044, 1003 cm^{-1}; HRMS (ESI): *m/z* calculated for $C_9H_9O_5S$ $[M+H]^+$: 229.0165, found: 229.0167.

5-(Carboxycarbonyl)thiophene-2-carboxylic acid (38).

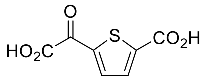

Dicarboxylic acid **38** (94 mg, 94%) was obtained from methyl 5-(2-methoxy-2-oxoacetyl)thiophene-2-carboxylate **S58** (114 mg, 0.5 mmol) according to General Procedure H.

Pale yellow solid, m.p.: >285 °C (decomposition); ^{1}H NMR (600 MHz, 300 K, D_2O): δ = 7.89 (d, J = 4.0 Hz, 1H), 7.62 ppm (d, J = 4.0 Hz, 1H); ^{13}C NMR (150 MHz, 300 K, D_2O): δ = 188.8, 170.6, 168.5, 150.9, 141.0, 137.1, 131.2 ppm; IR (film): ṽ = 3323, 3126, 3112, 2982, 1657, 1632, 1557, 1514, 1456, 1379, 1325, 1246, 1051 cm^{-1}; HRMS (ESI): *m/z* calculated for $C_7H_3O_5S$ $[M-H]^-$: 198.9707, found: 198.9705.

Methyl 2-(2-cyano-2-(tetrahydro-1-λ^4-thiophen-1-ylidene)acetyl)benzoate (S59).

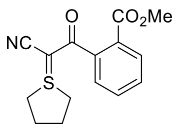

According to General Procedure F, cyanosulfur ylid **S59** (2.33 g, 81%) was obtained from 2-(methoxycarbonyl)benzoic acid (1.8 g, 10.0 mmol), following column chromatography (100 g KP-Sil; 50 mL/min; 100% ethyl acetate (8 CV), followed by a linear gradient (7 CV): 0%→100% acetone in ethyl acetate).

Yellow solid, m.p.: 90−91 °C; ^{1}H NMR (600 MHz, 300 K, $CDCl_3$): δ = 7.96 (d, J = 7.7 Hz, 1H), 7.55 (td, J = 7.5, 1.2 Hz, 1H), 7.44 (td, J = 7.6, 1.3 Hz, 1H), 7.43 (d, J = 7.4 Hz, 1H), 3.88 (s, 3H), 3.70−3.64 (m, 2H), 3.48−3.43 (m, 2H), 2.73−2.67 (m, 2H), 2.16−2.09 ppm (m, 2H); ^{13}C NMR (150 MHz, 300 K, $CDCl_3$): δ = 188.1, 166.4, 142.3, 132.2, 130.3, 129.0, 128.2, 127.4, 120.4, 54.3, 52.1, 44.0, 28.6 ppm; IR (film): ṽ = 3486, 2998, 2951, 2169, 1721, 1580, 1483, 1434, 1341, 1293, 1273, 1202, 1120, 1074, 1038, 959 cm^{-1}; HRMS (ESI): *m/z* calculated for $C_{15}H_{15}O_3NSNa$ $[M+Na]^+$: 312.0665, found: 312.0663.

Methyl 2-(2-methoxy-2-oxoacetyl)benzoate (S60).

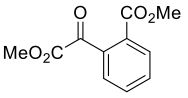

According to General Procedure G, dimethyl dicarboxylate **S60** (1.48 g, 83%) was obtained from cyanosulfur ylid **S59** (2.33 g, 8.1 mmol), following column chromatography (50 g KP-Sil; 50 mL/min; 100% cyclohexane (3 CV), followed by a linear gradient (8 CV): 0%→20% ethyl acetate in cyclohexane). The analytical data are in agreement with those reported.[25]

White solid, m.p.: 65−67 °C; ^{1}H NMR (600 MHz, 300 K, $CDCl_3$): δ = 8.04 (dd, J = 7.7, 0.9 Hz, 1H), 7.69 (td, J = 7.5, 1.2 Hz, 1H), 7.63 (td, J = 7.6, 1.3 Hz, 1H), 7.58 (dd, J = 7.5, 1.1 Hz, 1H), 3.91 (s, 3H), 3.90 ppm (s, 3H); ^{13}C NMR (150 MHz, 300 K, $CDCl_3$): δ = 187.3, 166.7, 161.3, 138.6, 133.0, 131.5, 129.7, 129.6, 129.0, 53.0, 52.7 ppm; IR (film): ṽ = 3002, 2956, 1760, 1735, 1710, 1596, 1577, 1437, 1292, 1256, 1207, 1140, 1089, 1045, 1014, 993, 961 cm^{-1}; HRMS (ESI): *m/z* calculated for $C_{11}H_{10}O_5Na$ [M+Na]$^+$: 245.0420, found: 245.0419.

2-(Carboxycarbonyl)benzoic acid (39).

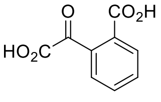

Dicarboxylic acid **39** (204 mg, quant.) was obtained from methyl 2-(2-methoxy-2-oxoacetyl)benzoate **S60** (222 mg, 1.0 mmol) according to General Procedure H.

White amorphous solid; ^{1}H NMR (600 MHz, 300 K, D_2O): δ = 7.68−7.64 (m, 2H), 7.61−7.57 ppm (m, 2H); ^{13}C NMR (150 MHz, 300 K, D_2O): δ = 197.5, 175.9, 169.7, 138.9, 134.7 (br), 132.3, 129.6, 129.1, 127.6 ppm; IR (film): ṽ = 3302, 3068, 2963, 2937, 17040, 1634, 1604, 1583, 1561, 1445, 1394, 1272, 1225 cm^{-1}; HRMS (ESI): *m/z* calculated for $C_9H_5O_5$ [M−H]$^-$: 193.0143, found: 193.0140.

Methyl 3-(2-cyano-2-(tetrahydro-1-λ^4-thiophen-1-ylidene)acetyl)benzoate (S61).

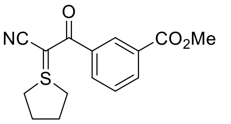

According to General Procedure F, cyanosulfur ylid **S61** (1.30 g, 81%) was obtained from 3-(methoxycarbonyl)benzoic acid (1.0 g, 5.6 mmol), following column chromatography (50 g KP-Sil; 30 mL/min; 100% ethyl acetate (8 CV), followed by a linear gradient (10 CV): 0%→100% acetone in ethyl acetate).

Beige solid, m.p.: 102−104 °C; ^{1}H NMR (500 MHz, 300 K, $CDCl_3$): δ = 8.47 (t, J = 1.6 Hz, 1H), 8.12 (dt, J = 7.8, 1.4 Hz, 1H), 8.01 (dt, J = 7.7, 1.4 Hz, 1H), 7.49 (t, J = 7.8 Hz, 1H), 3.92 (s, 3H), 3.52−3.44 (m, 4H), 2.66−2.58 (m, 2H), 2.17−2.09 ppm (m, 2H); ^{13}C NMR (125 MHz, 300 K, $CDCl_3$): δ = 184.8, 166.5, 138.7, 131.9, 131.8, 130.1, 128.9, 128.2, 120.5, 56.3, 52.3, 45.0, 28.4 ppm; IR (film): ṽ = 3515, 3069, 3002, 2951, 2165, 1719, 1604, 1588, 1557, 1438, 1347, 1293, 1272, 1201, 1122, 1080, 978 cm^{-1}; HRMS (ESI): *m/z* calculated for $C_{15}H_{16}O_3NS$ [M+H]$^+$: 290.0845, found: 290.0841.

Methyl 3-(2-methoxy-2-oxoacetyl)benzoate (S62).

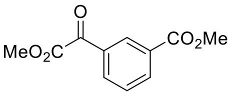

According to General Procedure G, dimethyl dicarboxylate **S62** (802 mg, 80%) was obtained from cyanosulfur ylid **S61** (1.30 g, 4.5 mmol), following column chromatography (25 g KP-Sil; 45 mL/min; 100% cyclohexane (3 CV), followed by a linear gradient (8 CV): 0%→20% ethyl acetate in cyclohexane).

White solid, m.p.: 54−56 °C; ^{1}H NMR (500 MHz, 300 K, $CDCl_3$): δ = 8.69 (t, J = 1.6 Hz, 1H), 8.35 (dt, J = 7.7, 1.4 Hz, 1H), 8.25 (dt, J = 7.8, 1.5 Hz, 1H), 7.64 (t, J = 7.8 Hz, 1H), 4.03 (s, 3H), 3.98 ppm (s, 3H); ^{13}C NMR (150 MHz, 300 K, $CDCl_3$): δ = 184.9, 165.8, 163.4, 135.6, 134.0, 132.8, 131.2, 131.1, 129.2, 53.0, 52.5 ppm; IR (film): ṽ = 3003, 2957, 1728, 1693, 1603, 1434, 1304, 1275, 1202, 1169, 1116, 1021, 974 cm^{-1}; HRMS (ESI): *m/z* calculated for $C_{11}H_{11}O_5$ [M+H]$^+$: 223.0601, found: 223.0603.

3-(Carboxycarbonyl)benzoic acid (40).

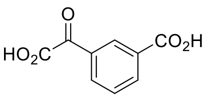

Dicarboxylic acid **40** (207 mg, quant.) was obtained from methyl 3-(2-methoxy-2-oxoacetyl)benzoate **S62** (222 mg, 1.0 mmol) according to General Procedure H.

White amorphous solid; ^{1}H NMR (600 MHz, 300 K, D_2O): δ = 8.41 (t, J = 1.7 Hz, 1H),

8.20−8.19 (m, 1H), 8.08−8.06 (m, 1H), 7.68 ppm (t, J = 7.7 Hz, 1H); ^{13}C NMR (150 MHz, 300 K, D_2O): δ = 196.5, 174.2, 172.8, 137.4, 135.0, 132.2 (2C), 129.5, 129.1 ppm; IR (film): ṽ = 3387, 2981, 1601, 1560, 1428, 1391, 1236, 1126 cm^{-1}; HRMS (ESI): *m/z* calculated for $C_9H_5O_5$ [M−H]$^-$: 193.0143, found: 193.0140.

Methyl 4-(2-cyano-2-(tetrahydro-1-λ^4-thiophen-1-ylidene)acetyl)benzoate (S63).

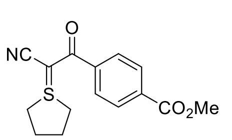

According to General Procedure F, sulfur ylid **S63** (2.61 g, 90%) was obtained from 4-(methoxycarbonyl)benzoic acid (1.8 g, 10.0 mmol), following column chromatography (100 g KP-Sil; 50 mL/min; 100% ethyl acetate (7 CV), followed by a linear gradient (7 CV): 0%→100% acetone in ethyl acetate).

White solid, m.p.: 149−151 °C; ^{1}H NMR (600 MHz, 300 K, $CDCl_3$): δ = 8.09−8.07 (m, 2H), 7.89−7.87 (m, 2H), 3.94 (s, 3H), 3.51−3.48 (m, 4H), 2.67−2.61 (m, 2H), 2.19−2.12 ppm (m, 2H); ^{13}C NMR (150 MHz, 300 K, $CDCl_3$): δ = 184.9, 166.5, 142.3, 132.0, 129.4, 127.7, 120.4, 56.7, 52.2, 45.0, 28.5 ppm; IR (film): ṽ = 3485, 2981, 2886, 2166, 1719, 1580, 1546, 1436, 1405, 1348, 1279, 1202, 1108, 1017, 957 cm^{-1}; HRMS (ESI): *m/z* calculated for $C_{15}H_{15}O_3NSNa$ [M+Na]$^+$: 312.0676, found: 312.0661.

Methyl 4-(2-methoxy-2-oxoacetyl)benzoate (S64).

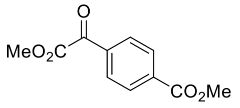

According to General Procedure G, dimethyl dicarboxylate **S64** (769 mg, 81%) was obtained from cyanosulfur ylid **S63** (1.24 g, 4.3 mmol), following column chromatography (25 g KP-Sil; 40 mL/min; 100% cyclohexane (3 CV), followed by a linear gradient (8 CV): 0%→20% ethyl acetate in cyclohexane). The analytical data are in agreement with those reported.[26]

White solid, m.p.: 102−103 °C; ^{1}H NMR (600 MHz, 300 K, $CDCl_3$): δ = 8.19−8.17 (m, 2H), 8.13−8.11 (m, 2H), 4.02 (s, 3H), 3.98 ppm (s, 3H); ^{13}C NMR (150 MHz, 300 K, $CDCl_3$): δ = 185.2, 165.8, 163.3, 135.6, 135.4, 130.0, 129.9, 53.0, 52.6 ppm; IR (film): ṽ = 2966, 1732, 1720, 1689, 1440, 1430, 1406, 1325, 1281, 1212, 1189, 1108, 1006, 915 cm^{-1}; HRMS (ESI): *m/z* calculated for $C_{11}H_{10}O_5Na$ [M+Na]$^+$: 245.0420, found: 245.0422.

4-(Carboxycarbonyl)benzoic acid (41).

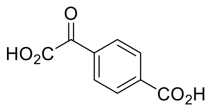

Dicarboxylic acid **41** (204 mg, quant.) was obtained from methyl 4-(2-methoxy-2-oxoacetyl)benzoate **S64** (222 mg, 1.0 mmol) according to General Procedure H.

White solid, m.p.: >350 °C (decomposition); ^{1}H NMR (600 MHz, 300 K, D_2O): δ = 8.02−8.01 (m, 2H), 8.00−7.99 ppm (m, 2H); ^{13}C NMR (150 MHz, 300 K, D_2O): δ = 196.5, 174.6, 172.8, 142.3, 133.7, 129.6, 129.1 ppm; IR (film): ṽ = 3395, 3066, 2981, 2884, 1684, 1593, 1549, 1400, 1377, 1236, 1217, 1131, 1100, 1069 cm^{-1}; HRMS (ESI): *m/z* calculated for $C_9H_5O_5$ [M−H]$^-$: 193.0143, found: 193.0140.

Methyl 4-(2-cyano-2-(tetrahydro-1-λ^4-thiophen-1-ylidene)acetyl)-3-fluorobenzoate (S65).

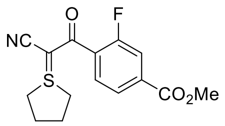

According to General Procedure B, 2-fluoro-4-(methoxycarbonyl)benzoic acid **13c** (1.26 g, 71%) was obtained from methyl 3-fluoro-4-iodobenzoate (2.50 g, 8.9 mmol) and used in the next reaction without further purification. Pale orange solid, m.p.: 151−153 °C; ^{1}H NMR (600 MHz, 300 K, DMSO-d_6): δ = 13.62 (brs, 1H), 7.98 (t, J = 7.7 Hz, 1H), 7.84 (dd, J = 8.1, 1.5 Hz, 1H), 7.76 (dd, J = 11.0, 1.4 Hz, 1H), 3.88 ppm (s, 3H); ^{13}C NMR (150 MHz, 300 K, DMSO-d_6): δ = 164.5 (d, J = 1.9 Hz), 164.3 (d, J = 2.9 Hz), 160.6 (d, J = 257.8 Hz), 134.9 (d, J = 7.8 Hz), 132.4, 124.9 (d, J = 4.0 Hz), 123.6 (d, J = 10.8 Hz), 117.3 (d, J = 24.3 Hz), 52.7 ppm; ^{19}F NMR (565 MHz, 300 K, DMSO-d_6): δ = −109.9 ppm (m, 1F); IR (film): ṽ = 3085, 2960, 2927, 1735, 1700, 1626, 1576, 1500, 1441, 1421, 1290, 1225, 1148, 1115, 1084, 992 cm^{-1}; HRMS (ESI): *m/z* calculated for $C_9H_6O_4F$ [M−H]$^-$: 197.0256, found: 197.0253.

According to General Procedure F, cyanosulfur ylid **S65** (1.79 g, 54%) was obtained from 2-fluoro-4-(methoxycarbonyl)benzoic acid **13c** (2.11 g, 10.9 mmol), following column chromatography (100 g KP-Sil; 60

mL/min; 100% ethyl acetate (8 CV), followed by a linear gradient (7 CV): 0%→100% acetone in ethyl acetate).
White solid, m.p.: 159−161 °C; ^{1}H NMR (600 MHz, 300 K, $CDCl_3$): δ = 7.86 (dd, J = 8.0, 1.4 Hz, 1H), 7.78 (dd, J = 10.1, 1.3 Hz, 1H), 7.57−7.55 (m, 1H), 3.94 (s, 3H), 3.54−3.52 (m, 4H), 2.70−2.65 (m, 2H), 2.19−2.14 ppm (m, 2H); ^{13}C NMR (150 MHz, 300 K, $CDCl_3$): δ = 181.8, 165.5 (d, J = 2.1 Hz), 158.9 (d, J = 251.0 Hz), 133.3 (d, J = 7.7 Hz), 131.9 (d, J = 16.5 Hz), 129.5 (d, J = 2.7 Hz), 125.2, 119.2, 117.4 (d, J = 23.6 Hz), 58.0, 52.5, 45.2, 28.6 ppm; ^{19}F NMR (565 MHz, 300 K, $CDCl_3$): δ = −116.2 ppm (m, 1F); IR (film): ṽ = 3510, 2954, 2173, 1723, 1581, 1497, 1437, 1413, 1348, 1291, 1251, 1210, 1107, 985 cm^{-1}; HRMS (ESI): *m/z* calculated for $C_{15}H_{15}O_3NFS$ $[M+H]^+$: 308.0751, found: 308.0750.

Methyl 3-fluoro-4-(2-methoxy-2-oxoacetyl)benzoate (S66).

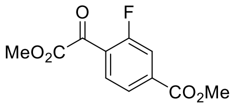

According to General Procedure G, dimethyl dicarboxylate **S66** (1.36 g, 98%) was obtained from cyanosulfur ylid **S65** (1.79 g, 5.8 mmol), following column chromatography (50 g KP-Sil; 50 mL/min; 100% cyclohexane (3 CV), followed by a linear gradient (8 CV): 0%→15% ethyl acetate in cyclohexane).
Pale yellow solid, m.p.: 39−40 °C; ^{1}H NMR (600 MHz, 300 K, $CDCl_3$): δ = 8.00−7.96 (m, 2H), 7.84 (d, J = 11.1 Hz, 1H), 4.00 (s, 3H), 3.99 ppm (s, 3H); ^{13}C NMR (150 MHz, 300 K, $CDCl_3$): δ = 183.2, 164.7 (d, J = 2.1 Hz), 163.6, 162.1 (d, J = 258.0 Hz), 137.5 (d, J = 8.1 Hz), 131.0, 125.6 (d, J = 3.1 Hz), 125.1 (d, J = 11.0 Hz), 117.8 (d, J = 23.2 Hz), 53.2, 52.9 ppm; ^{19}F NMR (565 MHz, 300 K, $CDCl_3$): δ = −113.6 ppm (m, 1F); IR (film): ṽ = 2959, 1730, 1697, 1621, 1575, 1493, 1438, 1418, 1286, 1243, 1202, 1180, 1120, 1089, 1005 cm^{-1}; HRMS (ESI): *m/z* calculated for $C_{11}H_{10}O_5F$ $[M+H]^+$: 241.0507, found: 241.0508.

4-(Carboxycarbonyl)-3-fluorobenzoic acid (42).

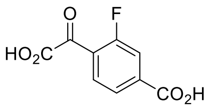

Dicarboxylic acid **42** (212 mg, quant.) was obtained from methyl 3-fluoro-4-(2-methoxy-2-oxoacetyl)benzoate **S66** (240 mg, 1.0 mmol) according to General Procedure H.
White solid, m.p.: >310 °C (decomposition); ^{1}H NMR (600 MHz, 300 K, D_2O): δ = 7.97 (t, J = 7.6 Hz, 1H), 7.83 (dd, J = 8.1, 1.3 Hz, 1H), 7.73 ppm (dd, J = 11.5, 1.2 Hz, 1H); ^{13}C NMR (150 MHz, 300 K, D_2O): δ = 192.8, 172.4, 171.9, 162.2 (d, J = 257.0 Hz), 143.0 (d, J = 7.8 Hz), 130.9, 125.0 (d, J = 3.2 Hz), 123.5 (d, J = 10.7 Hz), 116.9 ppm (d, J = 22.6 Hz); ^{19}F NMR (565 MHz, 300 K, D_2O): δ = −112.4 ppm (m, 1F); IR (film): ṽ = 3348, 2981, 1683, 1636, 1603, 1566, 1490, 1408, 1386, 1310, 1257, 1222, 1200, 1096, 1066, 1001 cm^{-1}; HRMS (ESI): *m/z* calculated for $C_9H_4O_5F$ $[M-H]^-$: 211.0048, found: 211.0047.

Methyl 4-(2-cyano-2-(tetrahydro-1-λ^4-thiophen-1-ylidene)acetyl)-2-fluorobenzoate (S67).

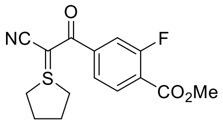

According to General Procedure B, 3-fluoro-4-(methoxycarbonyl)benzoic acid **13d** (1.95 g, 55%) was obtained from methyl 2-fluoro-4-iodobenzoate (5.0 g, 17.8 mmol) and used in the next reaction without further purification. ^{1}H NMR (600 MHz, 300 K, DMSO-d_6): δ = 13.61 (brs, 1H), 7.99 (t, J = 7.6 Hz, 1H), 7.84 (dd, J = 8.1, 1.5 Hz, 1H), 7.76 (dd, J = 11.1, 1.4 Hz, 1H), 3.87 ppm (s, 3H); ^{13}C NMR (150 MHz, 300 K, DMSO-d_6): δ = 165.4, 163.4 (d, J = 3.2 Hz), 160.5 (d, J = 258.5 Hz), 136.9 (d, J = 7.8 Hz), 132.2, 125.2 (d, J = 3.3 Hz), 121.8 (d, J = 10.7 Hz), 117.5 (d, J = 23.5 Hz), 52.7 ppm.
According to General Procedure F, cyanosulfur ylid **S67** (2.46 g, 82%) was obtained from 3-fluoro-4-(methoxycarbonyl)benzoic acid **13d** (1.95 g, 9.8 mmol), following column chromatography (100 g KP-Sil; 60 mL/min; 100% ethyl acetate (8 CV), followed by a linear gradient (8 CV): 0%→100% acetone in ethyl acetate).
White solid, m.p.: 106−108 °C; ^{1}H NMR (600 MHz, 300 K, $CDCl_3$): δ = 8.00−7.97 (m, 1H), 7.69 (dd, J = 8.0, 1.6 Hz, 1H), 7.59 (dd, J = 11.2, 1.5 Hz, 1H), 3.96 (s, 3H), 3.52−3.50 (m, 4H), 2.68−2.62 (m, 2H), 2.21−2.14 ppm (m, 2H); ^{13}C NMR (150 MHz, 300 K, $CDCl_3$): δ = 183.0, 164.4 (d, J = 3.5 Hz), 161.5 (d, J = 261.5 Hz), 144.1 (d, J = 7.5 Hz), 132.1, 123.1 (d, J = 4.0 Hz), 120.4 (d, J = 10.1 Hz), 120.0, 116.4 (d, J = 24.2 Hz), 57.0, 52.4, 45.0,

28.5 ppm; ^{19}F NMR (565 MHz, 300 K, $CDCl_3$): δ = −108.8 ppm (m, 1F); IR (film): ṽ = 3510, 2953, 2168, 1727, 1620, 1582, 1551, 1436, 1415, 1349, 1293, 1277, 1252, 1177, 1139, 1105, 938 cm^{-1}; HRMS (ESI): *m/z* calculated for $C_{15}H_{15}O_3NFS$ [M+H]$^+$: 308.0751, found: 308.0750.

Methyl 2-fluoro-4-(2-methoxy-2-oxoacetyl)benzoate (S68).

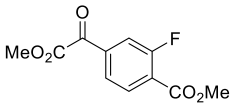

According to General Procedure G, dimethyl dicarboxylate **S68** (1.22 g, 63%) was obtained from cyanosulfur ylid **S67** (2.46 g, 8.0 mmol), following column chromatography (100 g KP-Sil; 60 mL/min; 100% cyclohexane (2 CV), followed by a linear gradient (6 CV): 0%→18% ethyl acetate in cyclohexane).

Pale yellow solid, m.p.: 63−65 °C; ^{1}H NMR (600 MHz, 300 K, $CDCl_3$): δ = 8.03 (d, *J* = 8.0, 7.0 Hz, 1H), 7.86 (dd, *J* = 8.1, 1.5 Hz, 1H), 7.81 (dd, *J* = 10.7, 1.5 Hz, 1H), 3.99 (s, 3H), 3.95 ppm (s, 3H); ^{13}C NMR (150 MHz, 300 K, $CDCl_3$): δ = 183.5, 163.7 (d, *J* = 4.1 Hz), 162.5, 161.4 (d, *J* = 262.0 Hz), 137.1 (d, *J* = 7.5 Hz), 132.6, 125.2 (d, *J* = 4.2 Hz), 123.9 (d, *J* = 10.9 Hz), 118.2 (d, *J* = 24.3 Hz), 53.1, 52.7 ppm; ^{19}F NMR (565 MHz, 300 K, $CDCl_3$): δ = −107.6 ppm (m, 1F); IR (film): ṽ = 3077, 2964, 1721, 1691, 1619, 1567, 1493, 1433, 1410, 1296, 1274, 1253, 1235, 1194, 1148, 1085, 1023, 963 cm^{-1}; HRMS (ESI): *m/z* calculated for $C_{11}H_{10}O_5F$ [M+H]$^+$: 241.0507, found: 241.0506.

4-(Carboxycarbonyl)-2-fluorobenzoic acid (43).

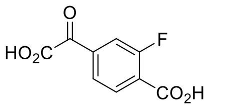

Dicarboxylic acid **43** (211 mg, quant.) was obtained from methyl 2-fluoro-4-(2-methoxy-2-oxoacetyl)benzoate **S68** (240 mg, 1.0 mmol) according to General Procedure H.

White solid, m.p.: >260 °C (decomposition); ^{1}H NMR (600 MHz, 300 K, D_2O): δ = 7.79 (dd, *J* = 7.9, 1.4 Hz, 1H), 7.77−7.74 ppm (m, 2H); ^{13}C NMR (150 MHz, 300 K, D_2O): δ = 194.9, 172.1, 171.9, 159.4 (d, *J* = 250.0 Hz), 134.8 (d, *J* = 7.0 Hz), 131.7 (d, *J* = 16.2 Hz), 130.2 (d, *J* = 1.9 Hz), 125.7, 116.7 ppm (apparent dd, *J* = 24.2, 5.3 Hz); ^{19}F NMR (565 MHz, 300 K, D_2O): δ = −114.9 ppm (m, 1F); IR (film): ṽ = 3365, 3066, 3027, 2980, 1592, 1497, 1404, 1270, 1240, 1168, 1114, 1062 cm^{-1}; HRMS (ESI): *m/z* calculated for $C_9H_4O_5F$ [M−H]$^-$: 211.0048, found: 211.0047.

Methyl 3-bromo-4-(2-cyano-2-(tetrahydro-1-λ^4-thiophen-1-ylidene)acetyl)benzoate (S69).

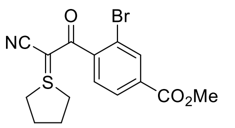

According to General Procedure B, 2-bromo-4-(methoxycarbonyl)benzoic acid **13e** (1.48 g, 72%) was obtained from methyl 3-bromo-4-iodobenzoate (2.70 g, 7.92 mmol) and used in the next reaction without further purification. ^{1}H NMR (600 MHz, 300 K, $CDCl_3$): δ = 8.38 (d, *J* = 1.5 Hz, 1H), 8.06 (dd, *J* = 8.1, 1.6 Hz, 1H), 8.02 (d, *J* = 8.1 Hz, 1H), 3.98 ppm (s, 3H); ^{13}C NMR (150 MHz, 300 K, $CDCl_3$): δ = 168.5, 164.9, 135.6, 134.5, 134.2, 132.0, 128.2, 122.3, 52.8 ppm; IR (film): ṽ = 3075, 2963, 2892, 1737, 1716, 1685, 1557, 1485, 1437, 1407, 1379, 1300, 1267, 1127, 1046, 964 cm^{-1}; HRMS (ESI): *m/z* calculated for $C_9H_6O_4Br$ [M−H]$^-$: 256.9455, found: 256.9455.

According to General Procedure F, cyanosulfur ylid **S69** (1.23 g, 57%) was obtained from 2-bromo-4-(methoxycarbonyl)benzoic acid **13e** (1.53 g, 5.9 mmol), following column chromatography (50 g KP-Sil; 55 mL/min; 100% ethyl acetate (9 CV), followed by a linear gradient (7 CV): 0%→100% acetone in ethyl acetate).

Brown solid, m.p.: 145−147 °C; ^{1}H NMR (600 MHz, 300 K, $CDCl_3$): δ = 8.26 (d, *J* = 1.1 Hz, 1H), 8.01 (dd, *J* = 7.9, 1.4 Hz, 1H), 7.43 (d, *J* = 7.9 Hz, 1H), 3.94 (s, 3H), 3.60−3.55 (m, 2H), 3.55−3.50 (m, 2H), 2.74−2.67 (m, 2H), 2.20−2.14 ppm (m, 2H); ^{13}C NMR (150 MHz, 300 K, $CDCl_3$): δ = 185.3, 165.3, 145.1, 134.2, 132.1, 128.5, 128.1, 119.5, 119.1, 56.9, 52.5, 45.0, 28.7 ppm; IR (film): ṽ = 3498, 2996, 2952, 2172, 1724, 1579, 1480, 1435, 1381, 1344, 1288, 1248, 1201, 1113, 1035, 968 cm^{-1}; HRMS (ESI): *m/z* calculated for $C_{15}H_{15}O_3NBrS$ [M+H]$^+$: 367.9951, found: 367.9950.

Methyl 3-bromo-4-(2-methoxy-2-oxoacetyl)benzoate (S70).

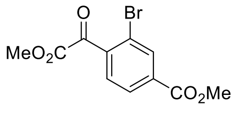

According to General Procedure G, dimethyl dicarboxylate **S70** (811 mg, 86%) was obtained from cyanosulfur ylid **S69** (1.23 g, 3.3 mmol), following column chromatography (50 g KP-Sil; 50 mL/min; 100% cyclohexane (3 CV), followed by a linear gradient (8 CV): 0%→15% ethyl acetate in cyclohexane).

White solid, m.p.: 78−80 °C; ^{1}H NMR (600 MHz, 300 K, $CDCl_3$): δ = 8.31 (d, J = 1.4 Hz, 1H), 8.11 (dd, J = 8.0, 1.5 Hz, 1H), 7.72 (d, J = 8.0 Hz, 1H), 3.99 ppm (s, 6H); ^{13}C NMR (150 MHz, 300 K, $CDCl_3$): δ = 186.4, 164.7, 161.9, 139.5, 134.9, 134.5, 131.3, 128.6, 121.1, 53.5, 52.8 ppm; IR (film): ṽ = 2955, 1756, 1729, 1555, 1436, 1383, 1283, 1250, 1206, 1116, 1054, 1005 cm^{-1}; HRMS (ESI): *m/z* calculated for $C_{11}H_9O_5BrNa$ $[M+Na]^+$: 322.9526, found: 322.9526.

3-Bromo-4-(carboxycarbonyl)benzoic acid (44).

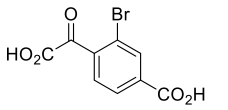

Dicarboxylic acid **44** (127 mg, 93%) was obtained from methyl 3-bromo-4-(2-methoxy-2-oxoacetyl)benzoate **S70** (144 mg, 0.5 mmol) according to General Procedure H.

White solid, m.p.: >300 °C (decomposition); ^{1}H NMR (600 MHz, 300 K, D_2O): δ = 8.20 (d, J = 1.4 Hz, 1H), 7.99 (dd, J = 7.9, 1.5 Hz, 1H), 7.73 ppm (d, J = 8.0 Hz, 1H); ^{13}C NMR (150 MHz, 300 K, D_2O): δ = 196.7, 171.7, 169.6, 139.5, 139.1, 133.9, 130.8, 128.2, 120.0 ppm; IR (film): ṽ = 3405, 3293, 3062, 2981, 1693, 1617, 1590, 1543, 1481, 1398, 1371, 1294, 1277, 1248, 1222, 1148, 1056 cm^{-1}; HRMS (ESI): *m/z* calculated for $C_9H_4O_5Br$ $[M-H]^-$: 270.9248, found: 270.9247.

Methyl 2-bromo-4-(2-cyano-2-(tetrahydro-1-λ^4-thiophen-1-ylidene)acetyl)benzoate (S71).

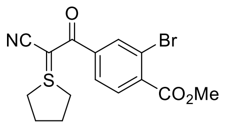

According to General Procedure B, 3-bromo-4-(methoxycarbonyl)benzoic acid **13f** (1.41 g, 31%) was obtained from methyl 2-bromo-4-iodobenzoate (5.87 g, 17.2 mmol) and used in the next reaction without further purification. ^{1}H NMR (600 MHz, 300 K, DMSO-d_6): δ = 13.60 (brs, 1H), 8.16 (d, J = 1.4 Hz, 1H), 8.00 (dd, J = 8.0, 1.6 Hz, 1H), 7.85 (d, J = 8.0 Hz, 1H), 3.88 ppm (s, 3H); ^{13}C NMR (150 MHz, 300 K, DMSO-d_6): δ = 165.8, 165.3, 136.1, 134.7, 134.0, 130.9, 128.5, 119.9, 52.8 ppm.

According to General Procedure F, cyanosulfur ylid **S71** (1.41 g, 70%) was obtained from 3-bromo-4-(methoxycarbonyl)benzoic acid **13f** (1.41 g, 5.4 mmol), following column chromatography (50 g KP-Sil; 50 mL/min; 100% ethyl acetate (7 CV), followed by a linear gradient (7 CV): 0%→100% acetone in ethyl acetate).

Beige solid, m.p.: 112−114 °C; ^{1}H NMR (600 MHz, 300 K, $CDCl_3$): δ = 8.09 (d, J = 1.2 Hz, 1H), 7.84 (dd, J = 8.0, 1.4 Hz, 1H), 7.82 (d, J = 7.9 Hz, 1H), 3.95 (s, 3H), 3.51−3.49 (m, 4H), 2.68−2.61 (m, 2H), 2.20−2.15 ppm (m, 2H); ^{13}C NMR (150 MHz, 300 K, $CDCl_3$): δ = 182.9, 166.2, 142.0, 133.9, 133.6, 131.0, 126.3, 121.6, 120.0, 56.9, 52.6, 45.0, 28.5 ppm; IR (film): ṽ = 3503, 2984, 2946, 2911, 2167, 1731, 1576, 1538, 1446, 1380, 1331, 1292, 1253, 1198, 1172, 1120, 1039, 916 cm^{-1}; HRMS (ESI): *m/z* calculated for $C_{15}H_{15}O_3NBrS$ $[M+H]^+$: 367.9951, found: 367.9953.

Methyl 2-bromo-4-(2-methoxy-2-oxoacetyl)benzoate (S72).

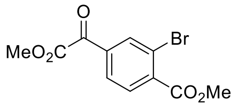

According to General Procedure G, dimethyl dicarboxylate **S72** (735 mg, 67%) was obtained from cyanosulfur ylid **S71** (1.41 g, 3.8 mmol), following column chromatography (50 g KP-Sil; 50 mL/min; 100% cyclohexane (3 CV), followed by a linear gradient (7 CV): 0%→15% ethyl acetate in cyclohexane).

Pale yellow solid, m.p.: 67−69 °C; ^{1}H NMR (600 MHz, 300 K, $CDCl_3$): δ = 8.34 (d, J = 1.4 Hz, 1H), 8.06 (dd, J = 8.0, 1.5 Hz, 1H), 7.88 (d, J = 8.0 Hz, 1H), 4.02 (s, 3H), 4.00 ppm (s, 3H); ^{13}C NMR (150 MHz, 300 K, $CDCl_3$): δ = 183.5, 165.8, 162.6, 137.8, 135.5, 135.4, 131.3, 128.5, 121.9, 53.2, 52.9 ppm; IR (film): ṽ = 2956, 1736, 1697, 1551, 1435, 1382, 1296, 1256, 1201, 1177, 1122, 1041, 1019 cm^{-1}; HRMS (ESI): *m/z* calculated for $C_{11}H_{10}O_5Br$

$[M+H]^+$: 300.9706, found: 300.9707.

2-Bromo-4-(carboxycarbonyl)benzoic acid (45).

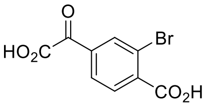

Dicarboxylic acid **45** (132 mg, 97%) was obtained from methyl 2-bromo-4-(2-methoxy-2-oxoacetyl)benzoate **S72** (144 mg, 0.5 mmol) according to General Procedure H.
Pale yellow amorphous solid; ^{1}H NMR (600 MHz, 300 K, D_2O): δ = 8.19 (d, *J* = 1.5 Hz, 1H), 7.93 (dd, *J* = 7.9, 1.5 Hz, 1H), 7.51 ppm (d, *J* = 7.9 Hz, 1H); ^{13}C NMR (150 MHz, 300 K, D_2O): δ = 194.8, 175.3, 172.1, 147.3, 133.5, 133.0, 129.1, 127.2, 117.6 ppm; IR (film): ṽ = 3364, 3065, 2980, 1580, 1509, 1496, 1405, 1366, 1267, 1219, 1161, 1111 cm^{-1}; HRMS (ESI): *m/z* calculated for $C_9H_4O_5Br$ $[M-H]^-$: 270.9237, found: 270.9250.

Methyl 4-(2-cyano-2-(tetrahydro-1-λ⁴-thiophen-1-ylidene)acetyl)-3-methylbenzoate (S73).

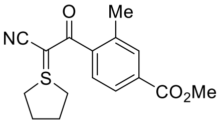

According to General Procedure B, 4-(methoxycarbonyl)-2-methylbenzoic acid **13g** (373 mg, 11%) was obtained from methyl 4-iodo-3-methylbenzoate (5.0 g, 18.1 mmol) and used in the next reaction without further purification. According to General Procedure F, cyanosulfur ylid **S73** (393 mg, 68%) was obtained from 4-(methoxycarbonyl)-2-methylbenzoic acid **13g** (373 mg, 1.9 mmol), following column chromatography (25 g Ultra; 45 mL/min; 100% ethyl acetate (7 CV), followed by a linear gradient (7 CV): 0%→100% acetone in ethyl acetate).
Beige solid, m.p.: 133−135 °C; ^{1}H NMR (600 MHz, 300 K, $CDCl_3$): *δ* = 7.90 (s, 1H), 7.88 (d, *J* = 7.9 Hz, 1H), 7.42 (d, *J* = 7.9 Hz, 1H), 3.92 (s, 3H), 3.55−3.48 (m, 4H), 2.70−2.64 (m, 2H), 2.44 (s, 3H), 2.20−2.13 ppm (m, 2H); ^{13}C NMR (150 MHz, 300 K, $CDCl_3$): *δ* = 187.9, 166.8, 143.7, 135.3, 131.8, 130.7, 127.0, 126.9, 119.6, 57.3, 52.1, 45.3, 28.6, 19.1 ppm; IR (film): ṽ = 3530, 2981, 2952, 2170, 1718, 1576, 1492, 1437, 1403, 1342, 1295, 1259, 1207, 1115, 1078 cm^{-1}; HRMS (ESI): *m/z* calculated for $C_{16}H_{18}O_3NS$ $[M+H]^+$: 304.1002, found: 304.1003.

Methyl 4-(2-methoxy-2-oxoacetyl)-3-methylbenzoate (S74).

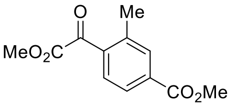

According to General Procedure G, dimethyl dicarboxylate **S74** (268 mg, 87%) was obtained from cyanosulfur ylid **S73** (393 mg, 1.3 mmol), following column chromatography (10 g Ultra; 30 mL/min; 100% cyclohexane (3 CV), followed by a linear gradient (8 CV): 0%→15% ethyl acetate in cyclohexane).
Pale yellow solid, m.p.: 77−79 °C; ^{1}H NMR (600 MHz, 300 K, $CDCl_3$): *δ* = 7.99 (s, 1H), 7.97 (d, *J* = 8.4 Hz, 1H), 7.76 (d, *J* = 8.0 Hz, 1H), 4.00 (s, 3H), 3.97 (s, 3H), 2.64 ppm (s, 3H); ^{13}C NMR (150 MHz, 300 K, $CDCl_3$): *δ* = 187.9, 166.0, 164.0, 140.9, 135.1, 134.0, 133.0, 131.6, 126.9, 53.0, 52.5, 21.1 ppm; IR (film): ṽ = 2980, 1727, 1694, 1568, 1436, 1400, 1299, 1265, 1201, 1108, 999 cm^{-1}; HRMS (ESI): *m/z* calculated for $C_{12}H_{13}O_5$ $[M+H]^+$: 237.0758, found: 237.0760.

4-(Carboxycarbonyl)-3-methylbenzoic acid (46).

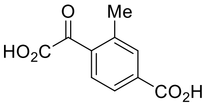

Dicarboxylic acid **46** (56 mg, quant.) was obtained from methyl 4-(2-methoxy-2-oxoacetyl)-3-methylbenzoate **S74** (59 mg, 0.25 mmol) according to General Procedure H.
White solid, m.p.: >320 °C (decomposition); ^{1}H NMR (600 MHz, 300 K, D_2O): *δ* = 7.83−7.81 (m, 2H), 7.78 (d, *J* = 7.9 Hz, 1H), 2.60 ppm (s, 3H); ^{13}C NMR (150 MHz, 300 K, D_2O): *δ* = 198.9, 173.8, 172.8, 140.1, 139.3, 134.6, 132.1, 131.2, 126.2, 20.1 ppm; IR (film): ṽ = 3399, 3063, 2980, 1698, 1673, 1623, 1590, 1548, 1496, 1407, 1296, 1276, 1223, 993 cm^{-1}; HRMS (ESI): *m/z* calculated for $C_{10}H_7O_5$ $[M-H]^-$: 207.0299, found: 207.0298.

Methyl 4-(2-cyano-2-(tetrahydro-1-λ^4-thiophen-1-ylidene)acetyl)bicyclo[2.2.2]octane-1-carboxylate (S75).

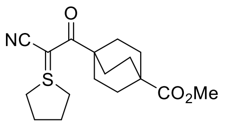

According to General Procedure F, cyanosulfur ylid **S75** (701 mg, 39%) was obtained from 4-(methoxycarbonyl)bicyclo[2.2.2]octane-1-carboxylic acid (1.19 g, 5.6 mmol), following column chromatography (50 g KP-Sil; 35 mL/min; 100% ethyl acetate (7 CV), followed by a linear gradient (5 CV): 0%→100% acetone in ethyl acetate).

Beige solid, m.p.: 202−204 °C; ^{1}H NMR (500 MHz, 300 K, $CDCl_3$): δ = 3.64 (s, 3H), 3.37−3.25 (m, 4H), 2.57−2.49 (m, 2H), 2.11−2.03 (m, 2H), 1.93−1.90 (m, 6H), 1.83−1.80 (m, 6H); ^{13}C NMR (125 MHz, 300 K, $CDCl_3$): δ = 196.4, 178.1, 121.1, 54.8, 51.6, 44.4 (2C), 42.5, 38.8, 28.2 (2C), 28.0 (3C), 27.5 (3C) ppm; IR (film): ṽ = 2951, 2922, 2871, 2156, 1721, 1556, 1453, 1417, 1355, 1330, 1308, 1233, 1180, 1160, 1069 cm^{-1}; HRMS (ESI): *m/z* calculated for $C_{17}H_{24}O_3NS$ $[M+H]^+$: 322.1471, found: 322.1471.

Methyl 4-(2-methoxy-2-oxoacetyl)bicyclo[2.2.2]octane-1-carboxylate (S76).

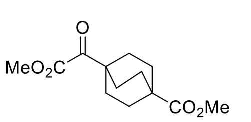

According to General Procedure G, dimethyl dicarboxylate **S76** (460 mg, 83%) was obtained from cyanosulfur ylid **S75** (701 mg, 2.18 mmol), following column chromatography (25 g KP-Sil; 40 mL/min; 100% cyclohexane (3 CV), followed by a linear gradient (8 CV): 0%→20% ethyl acetate in cyclohexane).

White solid, m.p.: 56−58 °C; ^{1}H NMR (600 MHz, 300 K, $CDCl_3$): δ = 3.85 (s, 3H), 3.67 (s, 3H), 1.90−1.84 ppm (m, 12H); ^{13}C NMR (150 MHz, 300 K, $CDCl_3$): δ = 200.6, 177.4, 163.7, 52.3, 51.8, 43.3, 38.8, 27.4, 26.4 ppm; IR (film): ṽ = 2980, 2874, 1731, 1712, 1458, 1435, 1382, 1238, 1173, 1137, 1068, 1024, 960 cm^{-1}; HRMS (ESI): *m/z* calculated for $C_{13}H_{19}O_5$ $[M+H]^+$: 255.1227, found: 255.1228.

4-(Carboxycarbonyl)bicyclo[2.2.2]octane-1-carboxylic acid (47).

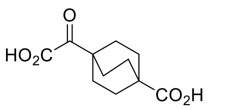

Dicarboxylic acid **47** (100 mg, 88%) was obtained from methyl 4-(2-methoxy-2-oxoacetyl)bicyclo[2.2.2]octane-1-carboxylate **S76** (127 mg, 0.5 mmol) according to General Procedure H.

White solid, m.p.: >300 °C (decomposition); ^{1}H NMR (600 MHz, 300 K, D_2O): δ = 1.82−1.77 ppm (m, 12H); ^{13}C NMR (150 MHz, 300 K, D_2O): δ = 214.3, 186.7, 173.3, 41.7, 40.1, 27.9, 27.1 ppm; IR (film): ṽ = 3421, 2955, 2870, 1686, 1609, 1583, 1417, 1340, 1290, 1270, 1186, 1163, 1080, 1024 cm^{-1}; HRMS (ESI): *m/z* calculated for $C_{11}H_{13}O_5$ $[M-H]^-$: 225.0768, found: 225.0766.

Methyl 1-(2-cyano-2-(tetrahydro-1-λ^4-thiophen-1-ylidene)acetyl)cyclopropane-1-carboxylate (S77).

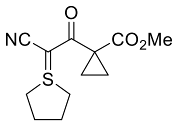

According to General Procedure F, cyanosulfur ylid **S77** (2.16 g, 85%) was obtained from 1-(methoxycarbonyl)cyclopropane-1-carboxylic acid (1.44 g, 10.0 mmol), following column chromatography (100 g KP-Sil; 50 mL/min; 100% ethyl acetate (7 CV), followed by a linear gradient (7 CV): 0%→100% acetone in ethyl acetate).

Beige solid, m.p.: 107−109 °C; ^{1}H NMR (600 MHz, 300 K, $CDCl_3$): δ = 3.75 (s, 3H), 3.42−3.38 (m, 4H), 2.66−2.60 (m, 2H), 2.12−2.06 (m, 2H), 1.49−1.47 (m, 2H), 1.33−1.31 ppm (m, 2H); ^{13}C NMR (150 MHz, 300 K, $CDCl_3$): δ = 185.9, 172.1, 119.7, 55.2, 52.5, 44.7, 32.7, 28.5, 15.9 ppm; IR (film): ṽ = 3500, 3004, 2952, 2169, 1719, 1580, 1436, 1355, 1308, 1253, 1199, 1172, 1126, 1038, 955 cm^{-1}; HRMS (ESI): *m/z* calculated for $C_{12}H_{15}O_3NSNa$ $[M+Na]^+$: 276.0665, found: 276.0664.

Methyl 1-(2-methoxy-2-oxoacetyl)cyclopropane-1-carboxylate (S78).

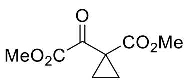

According to General Procedure G, dimethyl dicarboxylate **S78** (1.12 g, 70%) was obtained from cyanosulfur ylid **S77** (2.16 g, 8.53 mmol), following column chromatography (50 g KP-Sil; 50 mL/min; 100% cyclohexane (3 CV), followed by a linear gradient (8 CV): 0%→20% ethyl acetate in cyclohexane).

White solid, m.p.: 38−40 °C; ^{1}H NMR (600 MHz, 300 K, $CDCl_3$): δ = 3.93 (s, 3H), 3.75 (s, 3H), 1.75−1.71 (m, 2H), 1.71−1.67 ppm (m, 2H); ^{13}C NMR (150 MHz, 300 K, $CDCl_3$): δ = 191.3, 170.3, 163.4, 52.8, 52.5, 32.9, 20.6 ppm; IR (film): ṽ = 3012, 2959, 1738, 1719, 1440, 1320, 1255, 1201, 1165, 1061 cm^{-1}; HRMS (ESI): *m/z* calculated for $C_8H_{10}O_5Na$ $[M+Na]^+$: 209.0420, found: 209.0424.

1-(Carboxycarbonyl)cyclopropane-1-carboxylic acid (48).

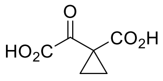

Dicarboxylic acid **48** (158 mg, quant.) was obtained from methyl 1-(2-methoxy-2-oxoacetyl)cyclopropane-1-carboxylate **S78** (186 mg, 1.0 mmol) according to General Procedure H.

White amorphous solid; ^{1}H NMR (600 MHz, 300 K, D_2O): δ = 1.52−1.50 (m, 2H), 1.45−1.43 ppm (m, 2H); ^{13}C NMR (150 MHz, 300 K, D_2O): δ = 205.3, 177.2, 171.9, 34.8, 19.3 ppm; IR (film): ṽ = 3370, 1575, 1406, 1291, 1234, 1075 cm^{-1}; HRMS (ESI): *m/z* calculated for $C_6H_5O_5$ $[M-H]^-$: 157.0143, found: 157.0141.

Methyl 2-(2-methoxy-2-oxoacetamido)-2-methylpropanoate (S79).

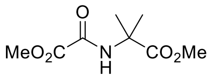

To a solution of 2-aminoisobutyric acid methyl ester hydrochloride (9.7 g, 63.1 mmol, 1.0 equiv.) in anhydrous dichloromethane (120 mL) were added sequentially *N*,*N*-diisopropylethylamine (24.2 mL, 139 mmol, 2.2 equiv.) and methyl chlorooxoacetate (6.4 mL, 69.5 mmol, 1.1 equiv.) at 0° C. The reaction mixture was stirred for 15 min at 0° C and for 2 h at ambient temperature before it was washed with aqueous HCl solution (1.0 M) followed by saturated aqueous $NaHCO_3$ solution. The organic layer was dried over anhydrous Na_2SO_4, filtered, and evaporated to afford the desired dimethyl dicarboxylate **S79** (8.7 g, 68%), following column chromatography (340 g KP-Sil; 100 mL/min; 100% cyclohexane (1 CV), followed by a linear gradient (8 CV): 0%→35% ethyl acetate in cyclohexane).

Clear colorless oil; ^{1}H NMR (600 MHz, 300 K, $CDCl_3$): δ = 7.71 (brs, 1H), 3.91 (s, 3H), 3.78 (s, 3H), 1.63 ppm (s, 6H); ^{13}C NMR (150 MHz, 300 K, $CDCl_3$): δ = 174.0, 160.9, 155.2, 57.1, 53.6, 52.9, 24.1 ppm; IR (film): ṽ = 3325, 2994, 2956, 1739, 1694, 1525, 1438, 1387, 1367, 1311, 1278, 1226, 1195, 1153, 1003 cm^{-1}; HRMS (ESI): *m/z* calculated for $C_8H_{13}O_5NNa$ $[M+Na]^+$: 226.0686, found: 226.0687.

2-(Carboxyformamido)-2-methylpropanoic acid (*N*-oxalyl-α-methylalanine, 49).

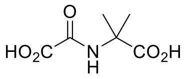

Dicarboxylic acid **49** (160 mg, 91%) was obtained from methyl 2-(2-methoxy-2-oxoacetamido)-2-methylpropanoate **S79** (203 mg, 1.0 mmol) according to General Procedure H.

White amorphous solid; ^{1}H NMR (600 MHz, 300 K, D_2O): δ = 1.50 ppm (s, 6H); ^{13}C NMR (150 MHz, 300 K, D_2O): δ = 180.9, 166.6, 163.5, 57.6, 24.1 ppm; IR (film): ṽ = 3355, 2992, 2938, 1644, 1596, 1532, 1470, 1403, 1363, 1254, 1199 cm^{-1}; HRMS (ESI): *m/z* calculated for $C_6H_8O_5N$ $[M-H]^-$: 174.0408, found: 174.0405.

6. Recombinant AspH production and purification

N-Terminally His_6-tagged human $AspH_{315-758}$ (His_6-$AspH_{315-758}$) was produced in *Escherichia coli* BL21 (DE3) cells using a pET-28a(+) vector and purified by Ni(II)-affinity chromatography (HisTrap HP column, GE Healthcare; 1 mL/min flow rate) and size-exclusion chromatography (HiLoad 26/60 Superdex 75 pg 300 mL column; 1 mL/min) using an ÄKTA pure machine (GE Healthcare) as previously reported.[1,4] AspH was >95% pure by SDS-PAGE and MS analysis and had the anticipated mass (calculated for His_6-$AspH_{315-758}$: 54519 Da, found: 54519 Da).[1] Purified AspH was stored in 50 mM HEPES buffer (pH 7.5, 150 mM NaCl) at a concentration of 125 µM at -78 °C; fresh aliquots were used for all AspH assays.

7. AspH substrate synthesis

AspH substrates were designed based on the sequence of EGFD1 of human coagulation factor X (hFX amino acids 86-124), which is a reported cellular AspH substrate.[2] All substrates were prepared with a C-terminal amide. The hFX-$EGFD1_{86-124}$-4Ser peptide (hFX amino acids 86-124; Supporting Figure S1c),[1] used for crystallography experiments, was synthesized by solid phase peptide synthesis (SPPS) and purified by GL Biochem (Shanghai) Ltd (Shanghai, China). The thioether linked cyclic peptide hFX-$CP_{101-119}$ (hFX amino acids 101-119; Supporting Figure S1d), which was used as substrate in AspH SPE-MS assays,[4] was synthesized by an intramolecular thioetherification cyclization reaction from the corresponding linear peptide (D-Ala replacing $Cys101_{hFX}$ and Ser replacing $Cys112_{hFX}$) which was obtained by microwave-assisted solid phase peptide synthesis using the Fmoc-protection strategy as described.[4]

8. AspH inhibition assays

AspH inhibition assays were performed at 2OG and Fe(II) concentrations close to their AspH K_m-values as previously described ($5 \cdot K_m$ of 2OG and $1.4 \cdot K_m$ of Fe(II), respectively).[3] Cosubstrate/cofactor stock solutions (L-ascorbic acid, LAA: 50 mM in Milli-Q® Ultrapure water; 2-oxoglutarate, 2OG: 10 mM in Milli-Q® Ultrapure water; ammonium iron(II) sulfate hexahydrate, FAS, $(NH_4)_2Fe(SO_4)_2 \cdot 6H_2O$: 400 mM in 20 mM HCl diluted to 1 mM in Milli-Q® Ultrapure water) were freshly prepared each day from commercially sourced solids (Sigma Aldrich).

Solutions of the 2OG mimetics (100% DMSO) were dry dispensed across 384-well polypropylene assay plates (Greiner) in an approximately three-fold and 11-point dilution series (100 µM top concentration) using an ECHO 550 acoustic dispenser (Labcyte). DMSO and 2,4-PDCA were used as negative and positive controls, respectively. The final DMSO concentration was kept constant at $0.5\%_{v/v}$ throughout all experiments (using the DMSO backfill option of the acoustic dispenser). Each reaction was performed in technical duplicates in adjacent wells of the assay plates; additionally, assays were performed in three independent technical duplicates on different days using different DMSO inhibitor solutions.

The Enzyme Mixture (25 µL/well), containing 0.1 µM His_6-$AspH_{315-758}$ in 50 mM HEPES buffer (pH 7.5), was dispensed across the inhibitor-containing 384-well assay plates with a multidrop dispenser (ThermoFischer Scientific) at 20°C under an ambient atmosphere. The plates were subsequently centrifuged (1000 rpm, 30 s) and incubated for 15 minutes at 20°C. The Substrate Mixture (25 µL/well), containing 2.0 µM hFX-$CP_{101-119}$, 200 µM LAA, 6.0 µM 2OG, and 4.0 µM FAS in 50 mM HEPES buffer (pH 7.5), was added using the multidrop dispenser. Note: The multidrop dispenser ensured proper mixing of the Enzyme and the Substrate Mixtures which was essential for assay reproducibility. The plates were centrifuged (1000 rpm, 30 s) and after incubating for 7 minutes, the enzyme reaction was stopped by addition of $10\%_{v/v}$ aqueous formic acid (5 µL/well). The plates were then centrifuged (1000 rpm, 60 s) and analysed by MS.

In a similar manner, AspH inhibition assays were performed at higher 2OG concentrations (using 200, 400, and

600 µM final 2OG assay concentrations), likely reflecting more physiologically relevant 2OG concentrations in healthy tissue. AspH inhibition assays were incubated for 6 rather than 7 minutes before the enzyme reaction was stopped by the addition of 10%$_{v/v}$ aqueous formic acid.
MS-analyses were performed using a RapidFire RF 365 high-throughput sampling robot (Agilent) attached to an iFunnel Agilent 6550 accurate mass quadrupole time-of-flight (Q-TOF) mass spectrometer operated in the positive ionization mode. Assay samples were aspirated under vacuum for 0.4 s and loaded onto a C4 solid phase extraction (SPE) cartridge. After loading, the C4 SPE cartridge was washed with 0.1%$_{v/v}$ aqueous formic acid to remove non-volatile buffer salts (5 s, 1.5 mL/min). The peptide was eluted from the SPE cartridge with 0.1%$_{v/v}$ aqueous formic acid in 85/15$_{v/v}$ acetonitrile/water into the mass spectrometer (5 s, 1.25 mL/min) and the SPE cartridge re-equilibrated with 0.1%$_{v/v}$ aqueous formic acid (1 s, 1.25 mL/min). The mass spectrometer parameters were: capillary voltage (4000 V), nozzle voltage (1000 V), fragmentor voltage (365 V), gas temperature (280°C), gas flow (13 L/min), sheath gas temperature (350°C), sheath gas flow (12 L/min). The m/z +2 charge states of the cyclic peptide (substrate) and the hydroxylated cyclic peptide (product) were used to extract ion chromatogram data, peak areas were integrated using RapidFire Integrator software (Agilent). Data were exported into Microsoft Excel and used to calculate the % conversion of the hydroxylation reaction using the equation: % conversion = 100 x (integral product cyclic peptide) / (integral substrate cyclic peptide + integral product cyclic peptide). Normalized dose-response curves (2,4-PDCA and DMSO controls) were obtained from the raw data by non-linear regression (GraphPad Prism 5) and used to determine IC_{50}-values. The standard deviation (SD) of three independent IC_{50} determinations (n = 3) was calculated using GraphPad Prism 5. Z'-factors were calculated according to the cited literature using Microsoft Excel (Supporting Figure S3).[14]

9. Determination of kinetic parameters

Maximum velocities (v_{max}^{app} monitoring hFX-CP$_{101\text{-}119}$ turnover) and Michaelis constants (K_{m}^{app} monitoring hFX-CP$_{101\text{-}119}$ turnover) of AspH were determined in independent triplicates for 2OG and 2OG derivatives by SPE-MS as previously described.[4] An Enzyme Mixture (0.1 mL) containing 0.6 µM His$_6$-AspH$_{315\text{-}758}$ in 50 mM HEPES buffer (pH 7.5) was added at 20°C to a Substrate Mixture (0.5 mL) containing 2.4 µM hFX-CP$_{101\text{-}119}$ peptide substrate (Supporting Figure S1d), 120 µM LAA, 24 µM FAS, and 2OG/2OG derivative (1.2 x final concentration) in 50 mM HEPES buffer (pH 7.5). Final 2OG/2OG derivative concentrations are given in Supporting Figure S6. Reactions were monitored with a rate of 1 sample/25 s using the same SPE-MS configuration as described above. Data were analyzed as described and the slopes of the initial reaction rates (Supporting Figure S6) fitted to a Michaelis-Menten plot using non-linear regression (GraphPad Prism 5). The total concentration of active His$_6$-AspH$_{315\text{-}758}$ was determined by an active site titration, using pyridine-2,4-dicarboxylic acid (2,4-PDCA) as tight binding AspH inhibitor, to be 90.8±13.7 nM[4] with an original estimated AspH assay concentration of 100 nM. The concentration of active His$_6$-AspH$_{315\text{-}758}$ was used to calculate turnover numbers (k_{cat}) of AspH with 2OG or the 2OG derivatives.

10. Crystallography and structure solutions

High-throughput crystallization experiments were performed in 96-well, 3-subwell, low profile Intelliplates (Art Robbins Instruments) using a Phoenix RE liquid dispensing robot (Art Robbins Instruments) and Hampton Research (PEG/Ion, Crystal Screen) or Molecular Dimensions (PACT Premier) crystallization screens. *N*-Terminally His$_6$-tagged AspH$_{315\text{-}758}$ (18 mg/mL in 50 mM HEPES buffer, pH 7.5) was mixed with 1 mM $MnCl_2$, 2 mM 2OG or 2OG derivative, and, when appropriate, the hFX-EGFD1$_{86\text{-}124}$-4Ser peptide (Supporting Figure S1c) as AspH substrate. Crystals were grown using the vapor diffusion method at 4°C in 200 nL or 300 nL sitting drops with 2:1, 1:1 or 1:2 sample:well solution ratios; precipitants are listed in the Supporting Tables S3 and S4.

Crystals were cryo-protected using mother liquor supplemented with 25%$_{v/v}$ glycerol before cryo-cooling in liquid N_2. Data were collected at 100 K using synchroton radiation at Diamond Light Source (DLS) beamlines I03 and I04. Data were indexed, integrated, and scaled using the autoPROC[27] and STARANISO[28] strategies of the beamline auto-processing pipeline (Supporting Tables S3 and S4); for the AspH:**17**:hFX-EGFD1$_{86\text{-}124}$-4Ser structure, data were indexed, integrated, and scaled using the Xia2[29] strategy of the beamline auto-processing pipeline (Supporting Table S4).

The AspH crystal structures were determined by molecular replacement (MR) using the AutoMR (PHASER[30]) subroutine in PHENIX[31]. The search model used for MR was based on PDB-ID 5JZA[1] for AspH crystal structures without substrate (AspH:2OG, AspH:**16**) and PDB-ID 5JTC[1] for AspH crystal structures with substrate (AspH:2OG:hFX-EGFD1$_{86\text{-}124}$-4Ser, AspH:**16**:hFX-EGFD1$_{86\text{-}124}$-4Ser, AspH:**34**:hFX-EGFD1$_{86\text{-}124}$-4Ser, AspH:**17**:hFX-EGFD1$_{86\text{-}124}$-4Ser, AspH:**49**:hFX-EGFD1$_{86\text{-}124}$-4Ser). The structural model was improved by iterative cycles of manual re-building in COOT[32] and crystallographic refinement in phenix.refine[33] (refinement details are summarized in Supporting Tables S3 and S4).

Crystal structure data for *N*-terminal His$_6$-tagged AspH$_{315\text{-}758}$ complexed to Mn, 2OG or 2OG derivatives (3-methyl-2OG, **16**; 3-ethyl-2OG, **17**; 4,4-dimethyl-2OG, **34**; *N*-oxalyl-α-methylalanine, **49**), and, in some cases, substrate peptide (hFX-EGFD1$_{86\text{-}124}$-4Ser) are deposited in the protein data bank with PDB accession codes: 6YYU (AspH:2OG), 6YYW (AspH:2OG:hFX-EGFD1$_{86\text{-}124}$-4Ser), 6YYV (AspH:**16**), 6YYX (AspH:**16**:hFX-EGFD1$_{86\text{-}124}$-4Ser), 6YYY (AspH:**34**:hFX-EGFD1$_{86\text{-}124}$-4Ser), 6Z6Q (AspH:**17**:hFX-EGFD1$_{86\text{-}124}$-4Ser), and 6Z6R (AspH:**49**:hFX-EGFD1$_{86\text{-}124}$-4Ser). PyMOL[34] was used for the generation of graphical representations; polder omit maps were calculated using Polder Maps[35] in PHENIX[31].

11. References

1. I. Pfeffer, L. Brewitz, T. Krojer, S. A. Jensen, G. T. Kochan, N. J. Kershaw, K. S. Hewitson, L. A. McNeill, H. Kramer, M. Münzel, R. J. Hopkinson, U. Oppermann, P. A. Handford, M. A. McDonough and C. J. Schofield, *Nat. Commun.*, 2019, **10**, 4910.
2. (a) P. Fernlund and J. Stenflo, *J. Biol. Chem.*, 1983, **258**, 12509-12512; (b) B. A. McMullen, K. Fujikawa, W. Kisiel, T. Sasagawa, W. N. Howald, E. Y. Kwa and B. Weinstein, *Biochemistry*, 1983, **22**, 2875-2884.
3. L. Brewitz, A. Tumber, I. Pfeffer, M. A. McDonough and C. J. Schofield, *Sci. Rep.*, 2020, **10**, 8650.
4. L. Brewitz, A. Tumber and C. J. Schofield, *J. Biol. Chem.*, 2020, **295**, 7826-7838.
5. R. F. Heck, *J. Am. Chem. Soc.*, 1968, **90**, 5518-5526.
6. (a) L. Horner, H. Hoffmann and H. G. Wippel, *Chem. Ber.*, 1958, **91**, 61-63; (b) W. S. Wadsworth and W. D. Emmons, *J. Am. Chem. Soc.*, 1961, **83**, 1733-1738.
7. F.-P. Wu, J.-B. Peng, L.-S. Meng, X. Qi and X.-F. Wu, *ChemCatChem*, 2017, **9**, 3121-3124.
8. E. Sagot, D. S. Pickering, X. Pu, M. Umberti, T. B. Stensbøl, B. Nielsen, M. Chapelet, J. Bolte, T. Gefflaut and L. Bunch, *J. Med. Chem.*, 2008, **51**, 4093-4103.
9. C. Gürtler and S. L. Buchwald, *Chem. Eur. J.*, 1999, **5**, 3107-3112.
10. (a) C. P. Ashcroft, S. Challenger, A. M. Derrick, R. Storey and N. M. Thomson, *Org. Process Res. Dev.*, 2003, **7**, 362-368; (b) D. Carmichael, H. Doucet and J. M. Brown, *Chem. Commun.*, 1999, 261-262; (c) Y. Liu, C. A. Sandoval, Y. Yamaguchi, X. Zhang, Z. Wang, K. Kato and K. Ding, *J. Am. Chem. Soc.*, 2006, **128**, 14212-14213; (d) T. Ireland, G. Grossheimann, C. Wieser-Jeunesse and P. Knochel, *Angew. Chem. Int. Ed.*, 1999, **38**, 3212-3215.
11. J. L. Li, H. W. Shi, Q. Wang, Y. H. Chai and J. Yang, *Org. Lett.*, 2017, **19**, 1497-1499.
12. (a) W. Tang, D. Liu and X. Zhang, *Org. Lett.*, 2003, **5**, 205-207; (b) M. J. Burk, F. Bienewald, M. Harris and A. Zanotti-Gerosa, *Angew. Chem. Int. Ed.*, 1998, **37**, 1931-1933.
13. H. Stobbe, *Liebigs Ann.*, 1899, **308**, 89-114.

14. J.-H. Zhang, T. D. Y. Chung and K. R. Oldenburg, *J. Biomol. Screen.*, 1999, **4**, 67-73.
15. (a) H. M. Hanauske-Abel and V. Günzler, *J. Theor. Biol.*, 1982, **94**, 421-455; (b) S. Martinez and R. P. Hausinger, *J. Biol. Chem.*, 2015, **290**, 20702-20711.
16. L. Ju, A. R. Lippert and J. W. Bode, *J. Am. Chem. Soc.*, 2008, **130**, 4253-4255.
17. P. C. J. Kamer, P. W. N. M. van Leeuwen and J. N. H. Reek, *Acc. Chem. Res.*, 2001, **34**, 895-904.
18. R. R. Gowda and E. Y. X. Chen, *Org. Chem. Front.*, 2014, **1**, 230-234.
19. (a) K. M. Steward and J. S. Johnson, *Org. Lett.*, 2011, **13**, 2426-2429; (b) M. Xian, S. Alaux, E. Sagot and T. Gefflaut, *J. Org. Chem.*, 2007, **72**, 7560-7566.
20. M. Sperrle and G. Consiglio, *J. Organomet. Chem.*, 1996, **506**, 177-180.
21. (a) G. Szöllősi, K. Balázsik and M. Bartók, *Appl. Catal. A Gen.*, 2007, **319**, 193-201; (b) K. Achiwa, P. A. Chaloner and D. Parker, *J. Organomet. Chem.*, 1981, **218**, 249-260.
22. S. Alaux, M. Kusk, E. Sagot, J. Bolte, A. A. Jensen, H. Bräuner-Osborne, T. Gefflaut and L. Bunch, *J. Med. Chem.*, 2005, **48**, 7980-7992.
23. V. Hélaine, J. Rossi, T. Gefflaut, S. Alaux and J. Bolte, *Adv. Synth. Catal.*, 2001, **343**, 692-697.
24. R. Kundu and C. Kulshreshtha, *RSC Adv.*, 2015, **5**, 77460-77468.
25. S. H. Kim, K. H. Kim and J. N. Kim, *Adv. Synth. Catal.*, 2011, **353**, 3335-3339.
26. J. Jiang, *J. Chem. Res.*, 2019, **43**, 235-240.
27. C. Vonrhein, C. Flensburg, P. Keller, A. Sharff, O. Smart, W. Paciorek, T. Womack and G. Bricogne, *Acta Cryst. D*, 2011, **67**, 293-302.
28. I. J. Tickle, O. Smart, C. Flensburg, P. Keller, W. Paciorek, A. Sharff, C. Vonrhein and G. Bricogne, 2018, STARANISO (http://staraniso.globalphasing.org/cgi-bin/staraniso.cgi). Cambridge, United Kingdom: Global Phasing Ltd.
29. G. Winter, *J. Appl. Cryst.*, 2010, **43**, 186-190.
30. A. J. McCoy, R. W. Grosse-Kunstleve, P. D. Adams, M. D. Winn, L. C. Storoni and R. J. Read, *J. Appl. Cryst.*, 2007, **40**, 658-674.
31. P. D. Adams, P. V. Afonine, G. Bunkoczi, V. B. Chen, I. W. Davis, N. Echols, J. J. Headd, L.-W. Hung, G. J. Kapral, R. W. Grosse-Kunstleve, A. J. McCoy, N. W. Moriarty, R. Oeffner, R. J. Read, D. C. Richardson, J. S. Richardson, T. C. Terwilliger and P. H. Zwart, *Acta Cryst. D*, 2010, **66**, 213-221.
32. P. Emsley, B. Lohkamp, W. G. Scott and K. Cowtan, *Acta Cryst. D*, 2010, **66**, 486-501.
33. P. V. Afonine, R. W. Grosse-Kunstleve, N. Echols, J. J. Headd, N. W. Moriarty, M. Mustyakimov, T. C. Terwilliger, A. Urzhumtsev, P. H. Zwart and P. D. Adams, *Acta Cryst. D*, 2012, **68**, 352-367.
34. W. L. DeLano, 2002, De Lano Scientific, San Carlos.
35. D. Liebschner, P. V. Afonine, N. W. Moriarty, B. K. Poon, O. V. Sobolev, T. C. Terwilliger and P. D. Adams, *Acta Cryst. D*, 2017, **73**, 148-157.

12. ^{1}H and ^{13}C NMR spectra of novel compounds prepared for this study

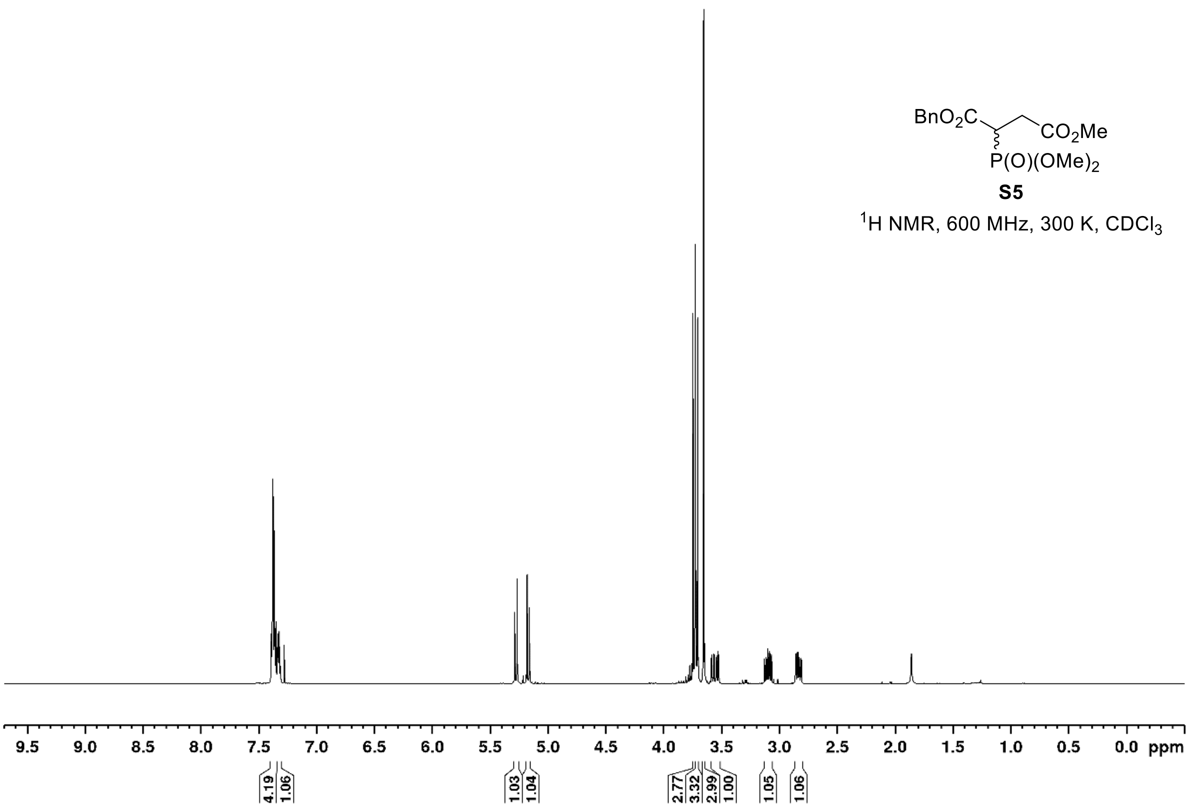

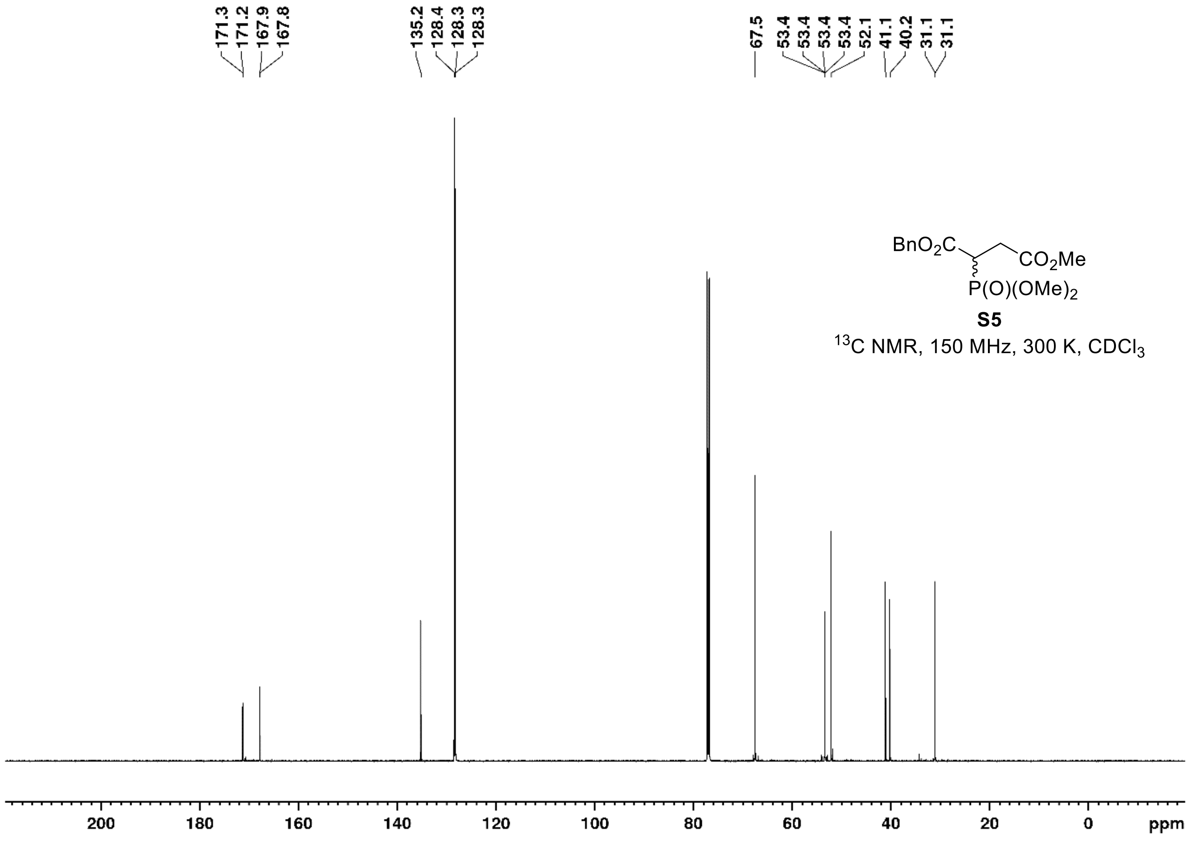

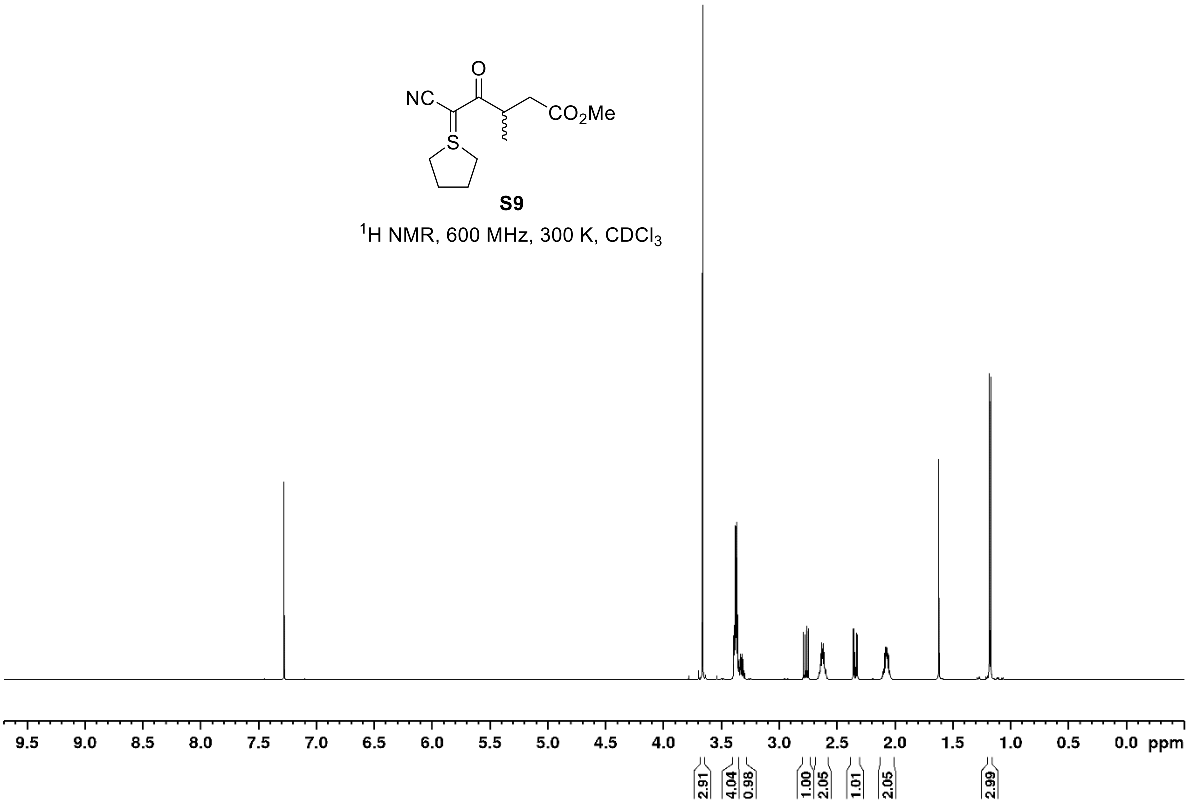
NC
O
CO2Me
S
S9
1H NMR, 600 MHz, 300 K, CDCl3
9.5 9.0 8.5 8.0 7.5 7.0 6.5 6.0 5.5 5.0 4.5 4.0 3.5 3.0 2.5 2.0 1.5 1.0 0.5 0.0 ppm
2.91 4.04 0.98 1.00 2.05 1.01 2.05 2.99

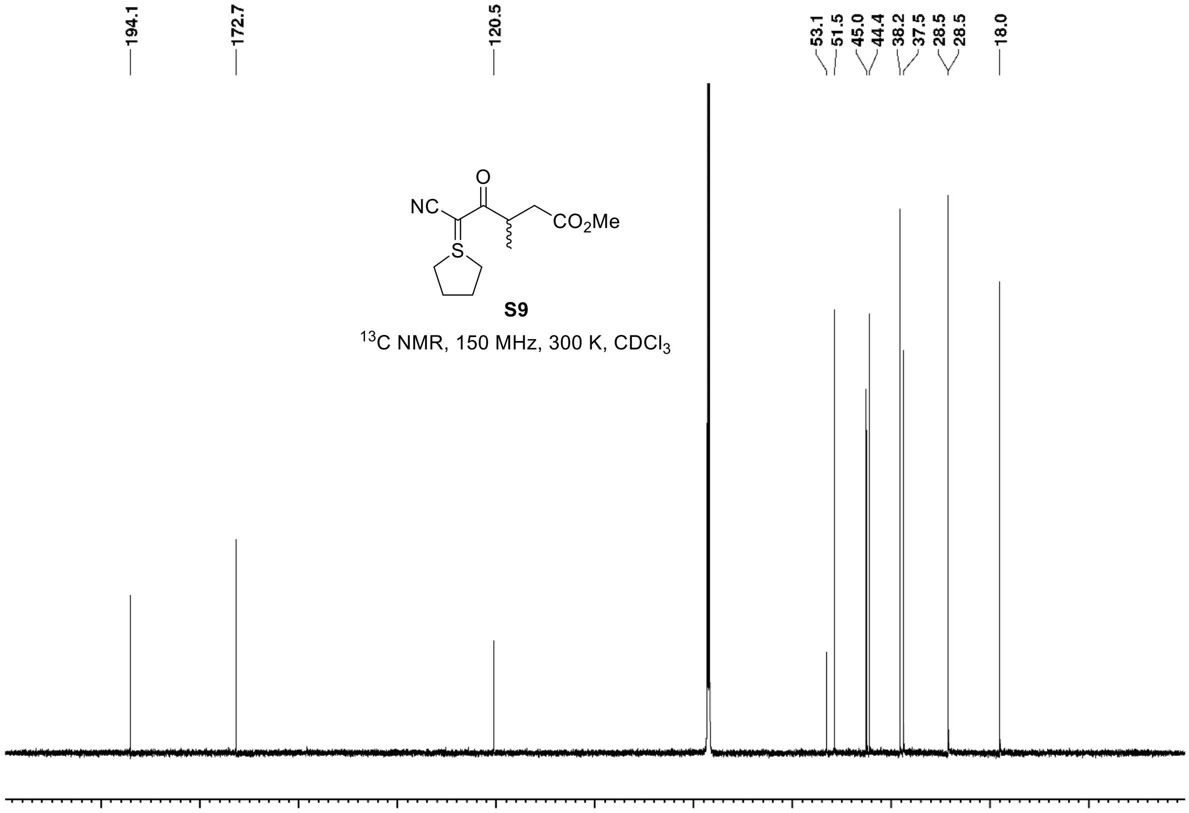
194.1
172.7
120.5
53.1 51.5 45.0 44.4 38.2 37.5 28.5 28.5 18.0
NC
O
CO2Me
S
S9
13C NMR, 150 MHz, 300 K, CDCl3
200 180 160 140 120 100 80 60 40 20 0 ppm

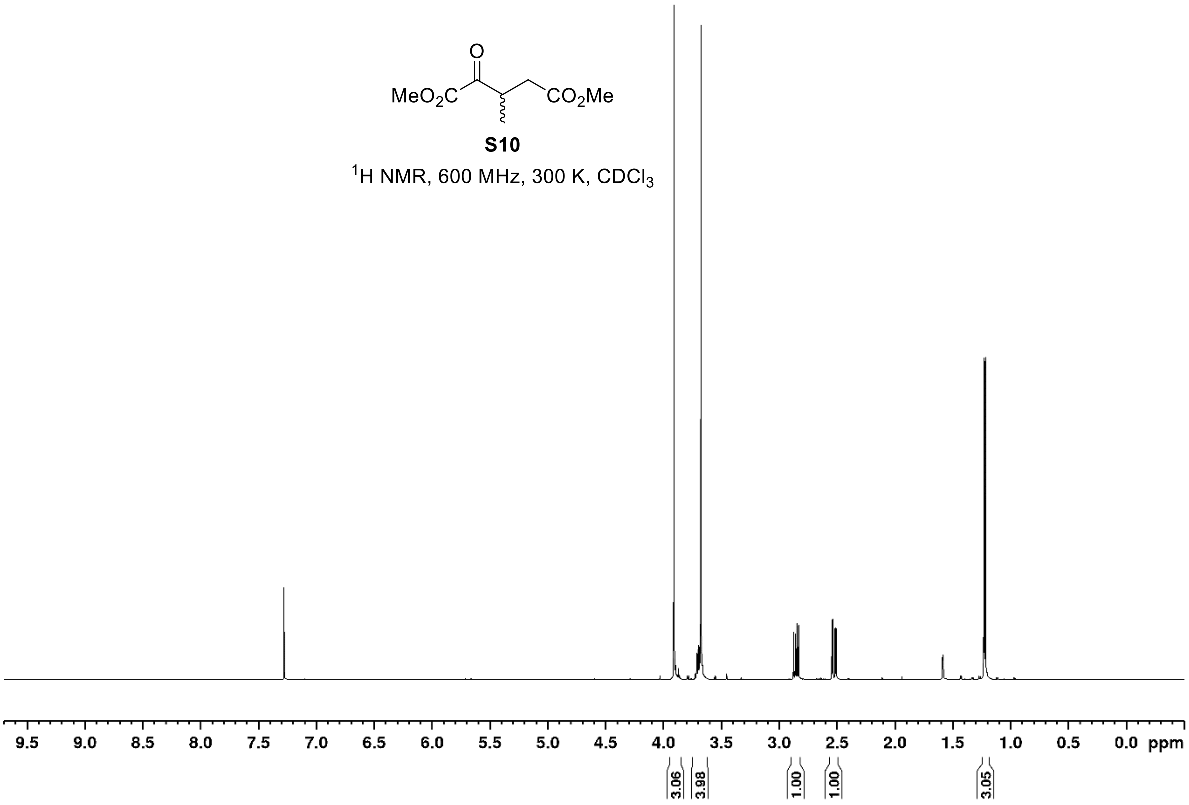
MeO2C
CO2Me
O
S10
1H NMR, 600 MHz, 300 K, CDCl3
9.5 9.0 8.5 8.0 7.5 7.0 6.5 6.0 5.5 5.0 4.5 4.0 3.5 3.0 2.5 2.0 1.5 1.0 0.5 0.0 ppm
3.06
3.98
1.00
1.00
3.05

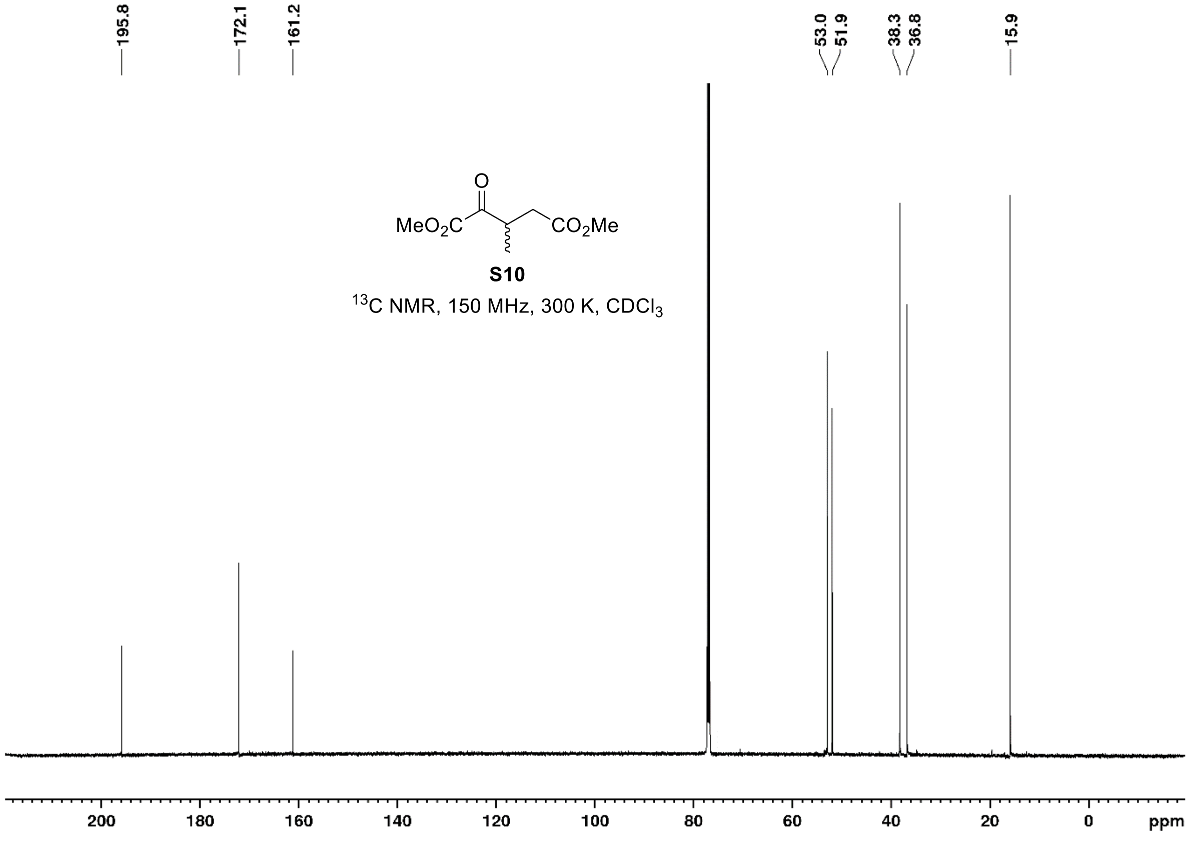
195.8
172.1
161.2
53.0
51.9
38.3
36.8
15.9
MeO2C
CO2Me
O
S10
13C NMR, 150 MHz, 300 K, CDCl3
200 180 160 140 120 100 80 60 40 20 0 ppm

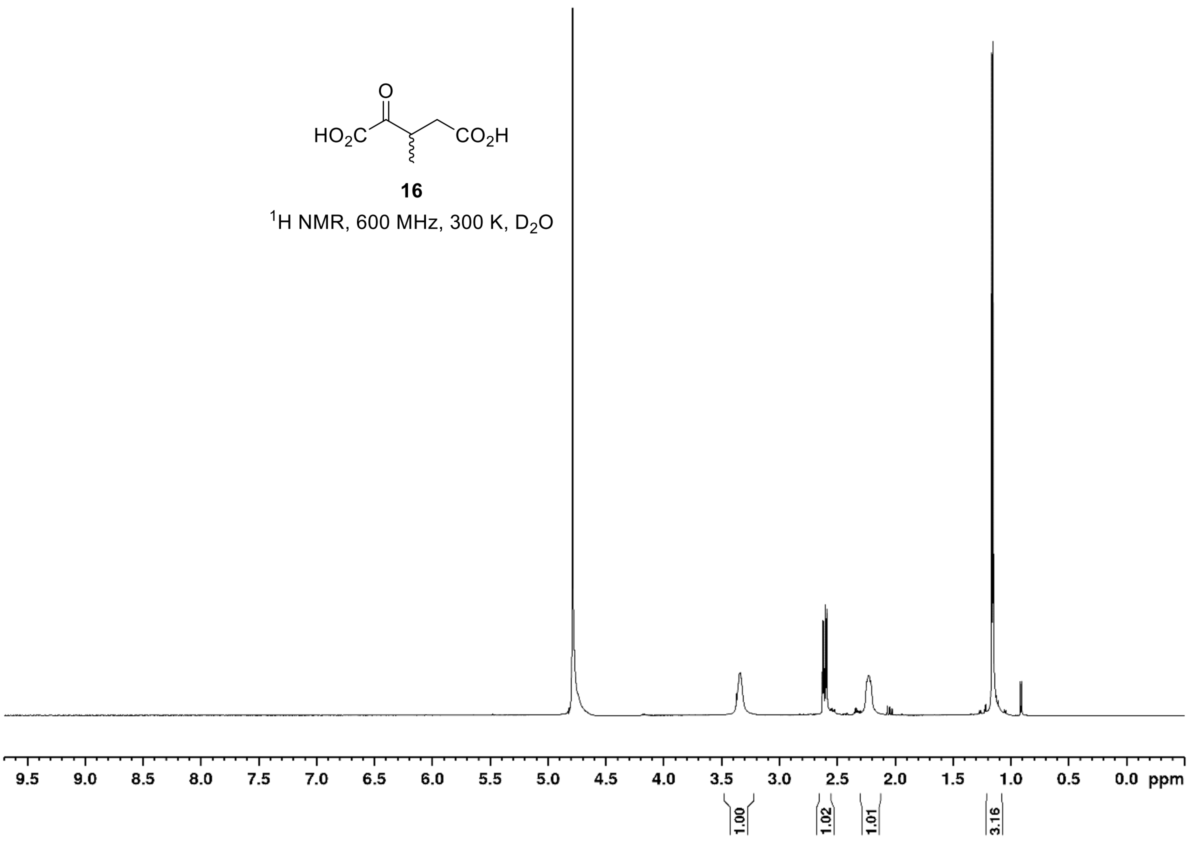
16
^{1}H NMR, 600 MHz, 300 K, D_2O
9.5 9.0 8.5 8.0 7.5 7.0 6.5 6.0 5.5 5.0 4.5 4.0 3.5 3.0 2.5 2.0 1.5 1.0 0.5 0.0 ppm
1.00
1.02
1.01
3.16

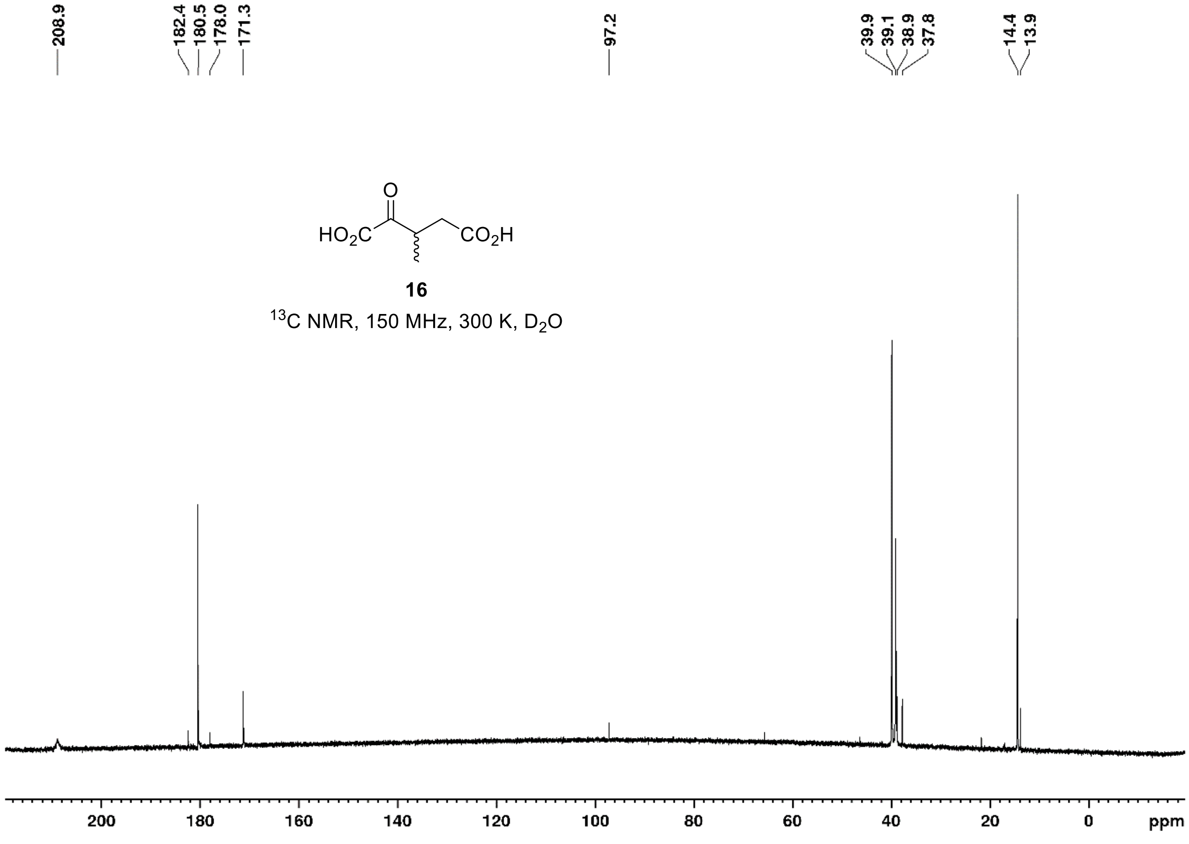
208.9
182.4
180.5
178.0
171.3
97.2
39.9
39.1
38.9
37.8
14.4
13.9
16
^{13}C NMR, 150 MHz, 300 K, D_2O
200 180 160 140 120 100 80 60 40 20 0 ppm

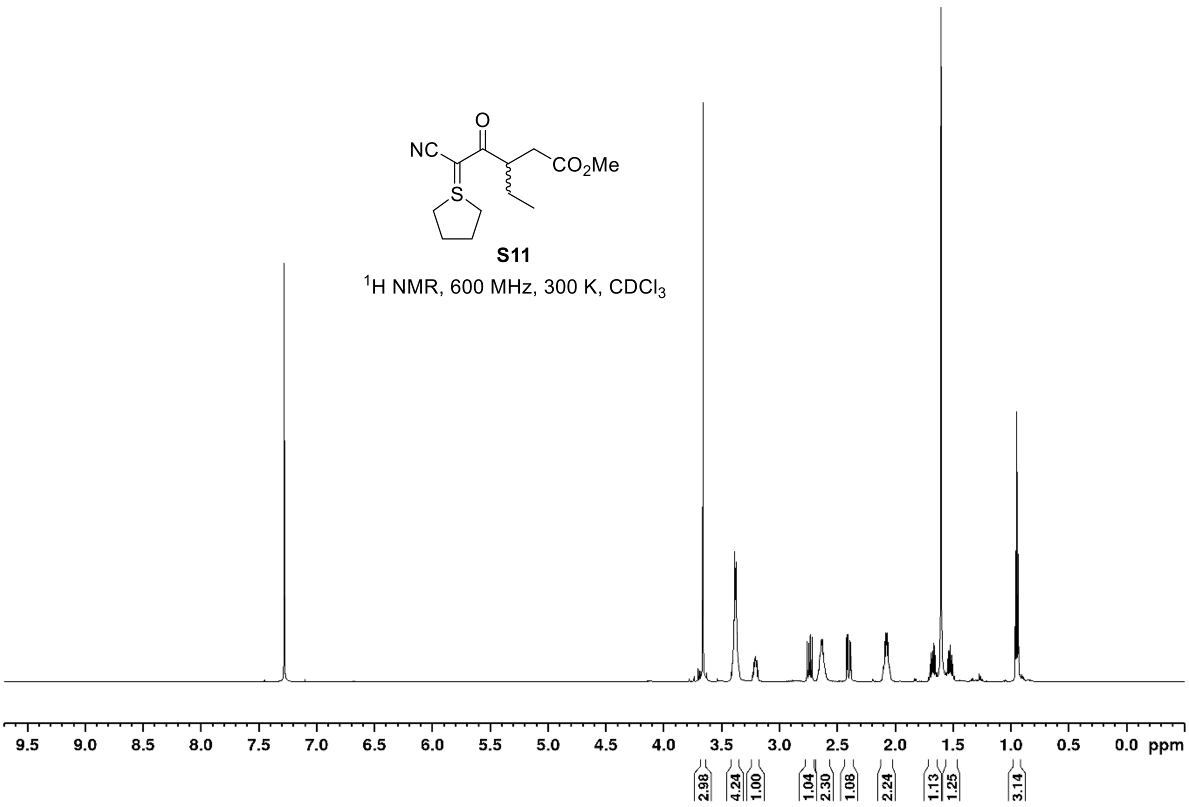
NC
O
CO2Me
S
S11
1H NMR, 600 MHz, 300 K, CDCl3
9.5 9.0 8.5 8.0 7.5 7.0 6.5 6.0 5.5 5.0 4.5 4.0 3.5 3.0 2.5 2.0 1.5 1.0 0.5 0.0 ppm
2.98 4.24 1.00 1.04 2.30 1.08 2.24 1.13 1.25 3.14

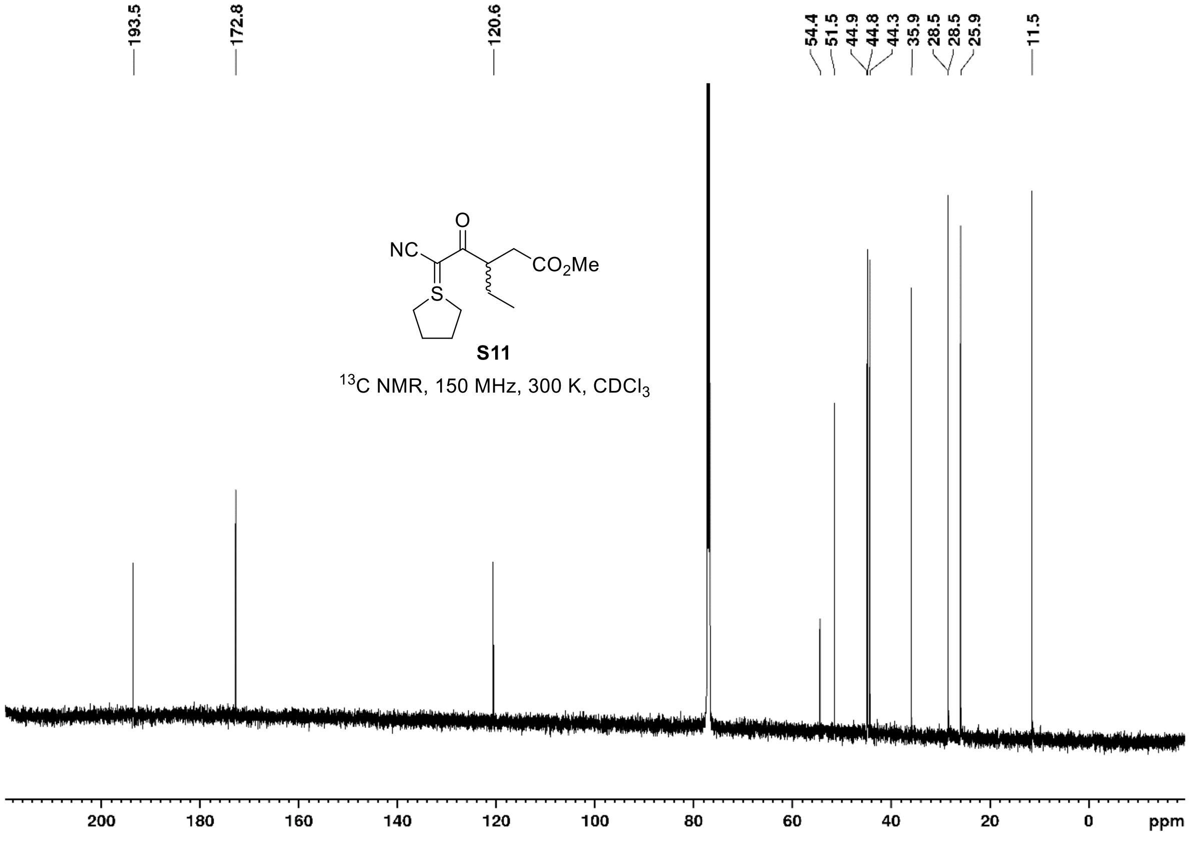
193.5
172.8
120.6
54.4 51.5 44.9 44.8 44.3 35.9 28.5 28.5 25.9
11.5
NC
O
CO2Me
S
S11
13C NMR, 150 MHz, 300 K, CDCl3
200 180 160 140 120 100 80 60 40 20 0 ppm

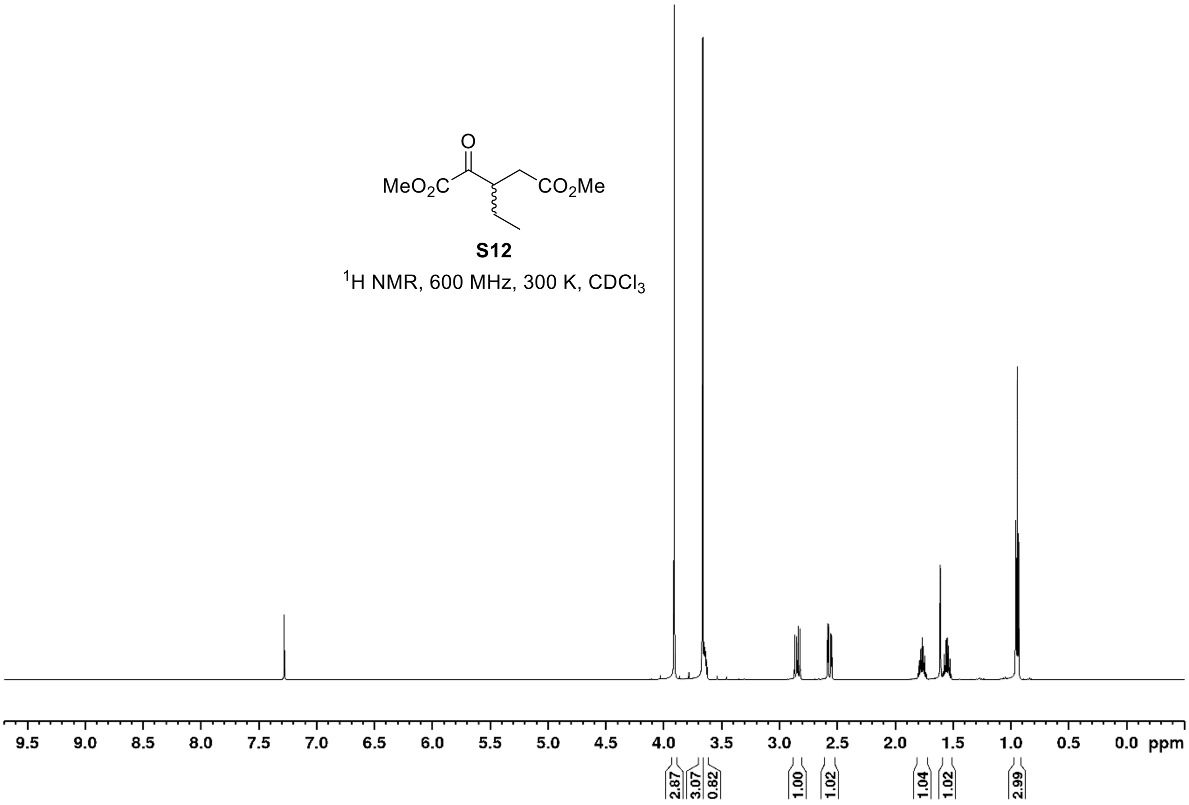

S12

^{1}H NMR, 600 MHz, 300 K, $CDCl_3$

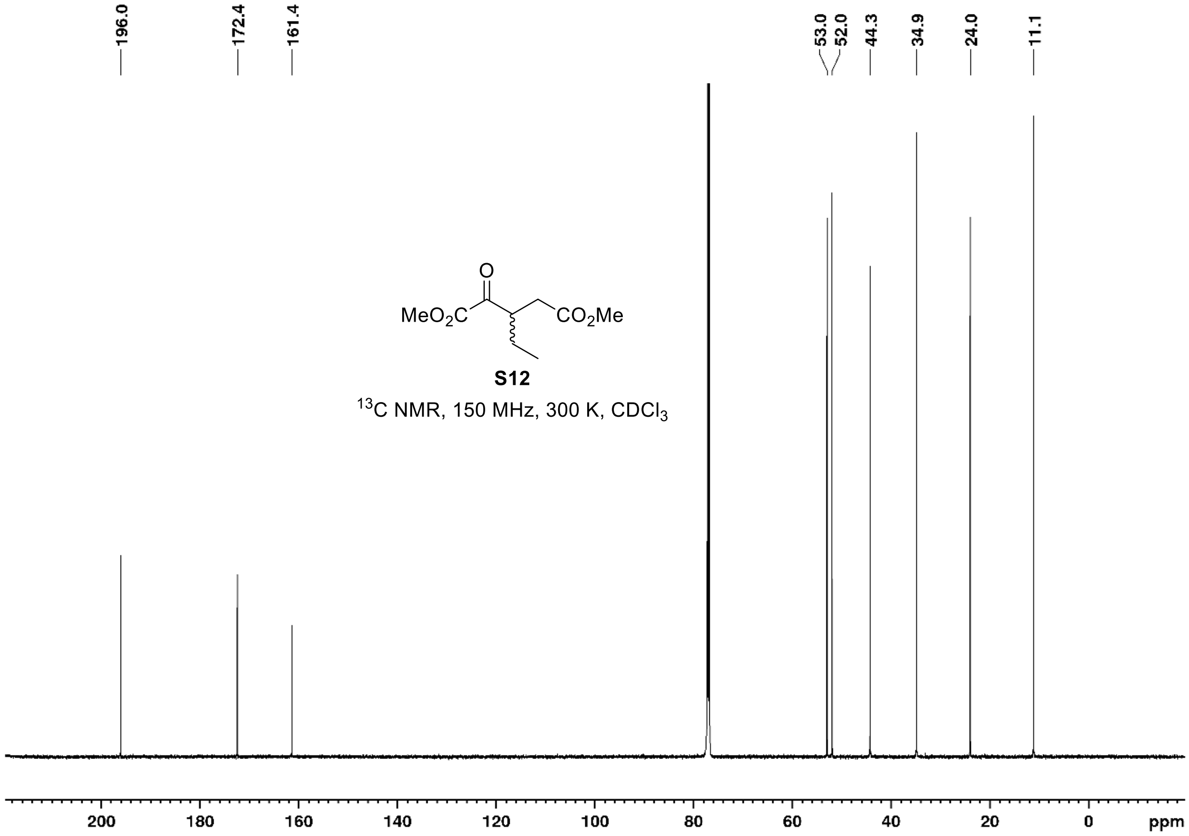

S12

^{13}C NMR, 150 MHz, 300 K, $CDCl_3$

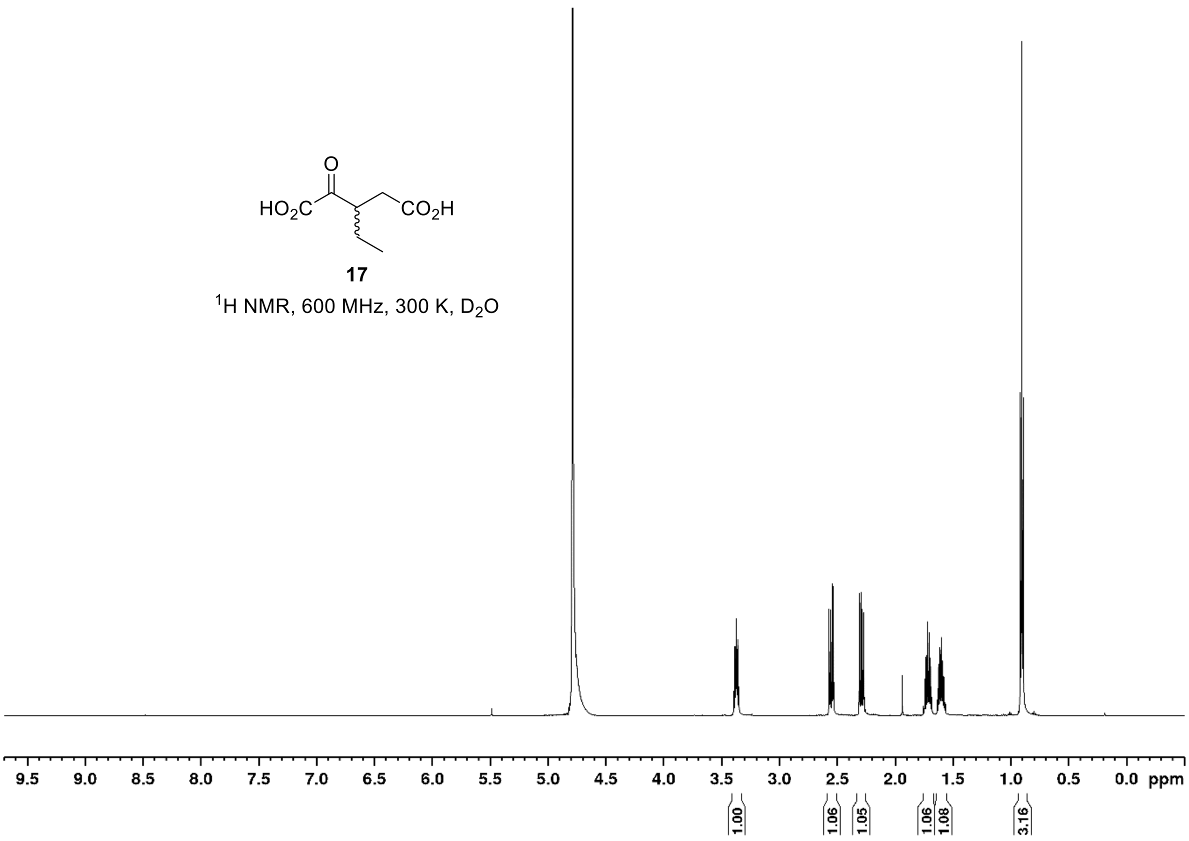
HO2C CO2H
17
1H NMR, 600 MHz, 300 K, D2O
9.5 9.0 8.5 8.0 7.5 7.0 6.5 6.0 5.5 5.0 4.5 4.0 3.5 3.0 2.5 2.0 1.5 1.0 0.5 0.0 ppm
1.00
1.06
1.05
1.06
1.08
3.16

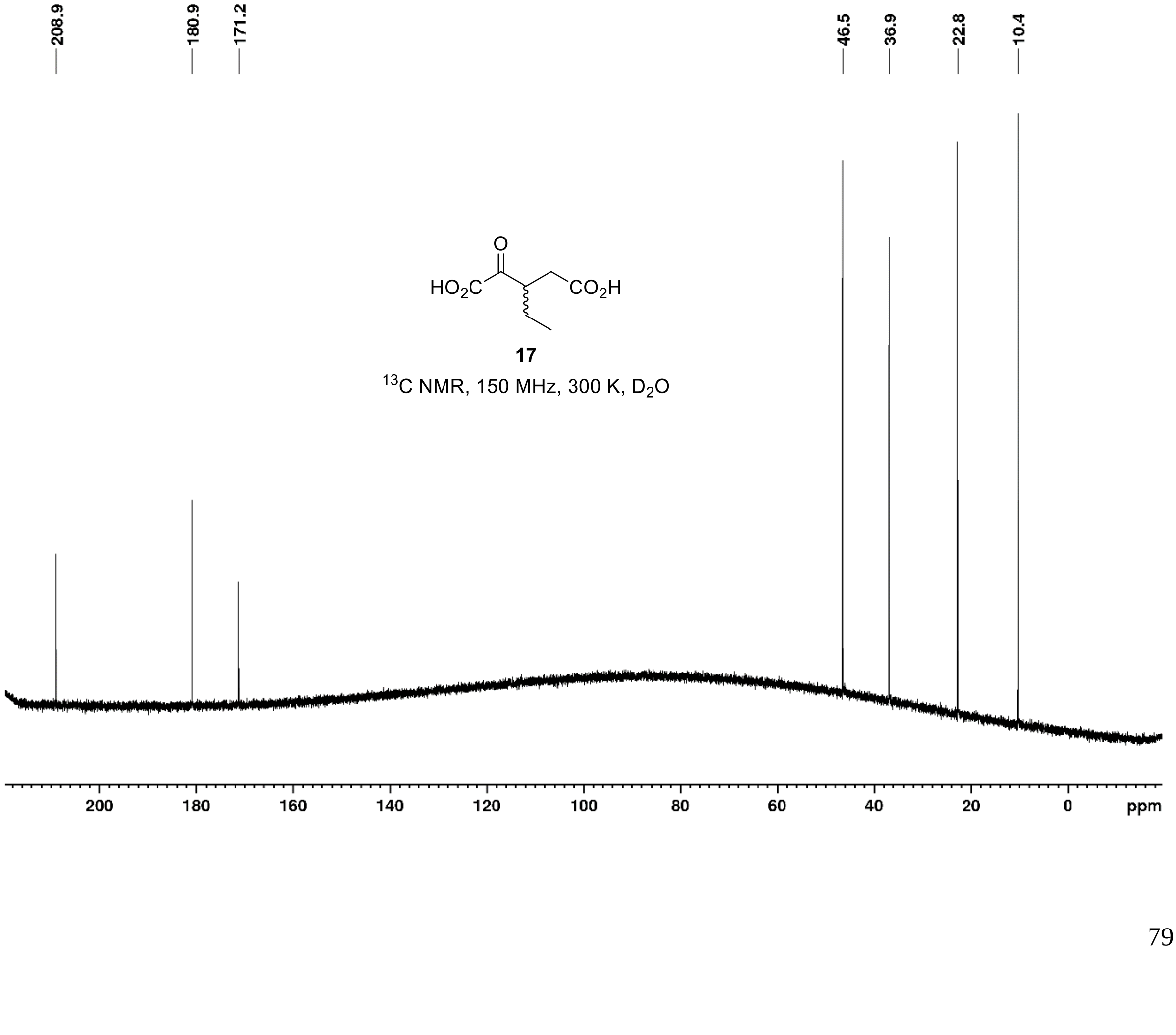
208.9
180.9
171.2
46.5
36.9
22.8
10.4
HO2C CO2H
17
13C NMR, 150 MHz, 300 K, D2O
200 180 160 140 120 100 80 60 40 20 0 ppm

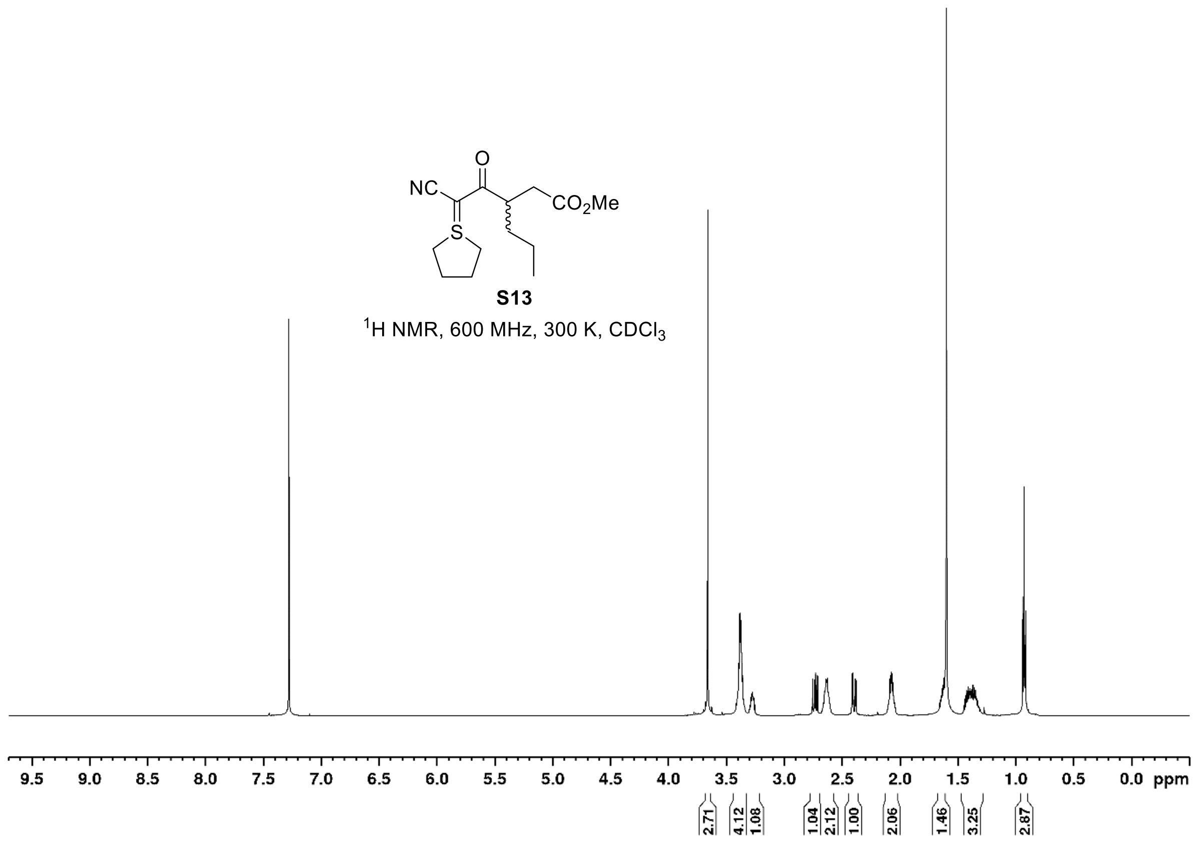
NC
O
CO2Me
S
S13
1H NMR, 600 MHz, 300 K, CDCl3
9.5 9.0 8.5 8.0 7.5 7.0 6.5 6.0 5.5 5.0 4.5 4.0 3.5 3.0 2.5 2.0 1.5 1.0 0.5 0.0 ppm
2.71 4.12 1.08 1.04 2.12 1.00 2.06 1.46 3.25 2.87

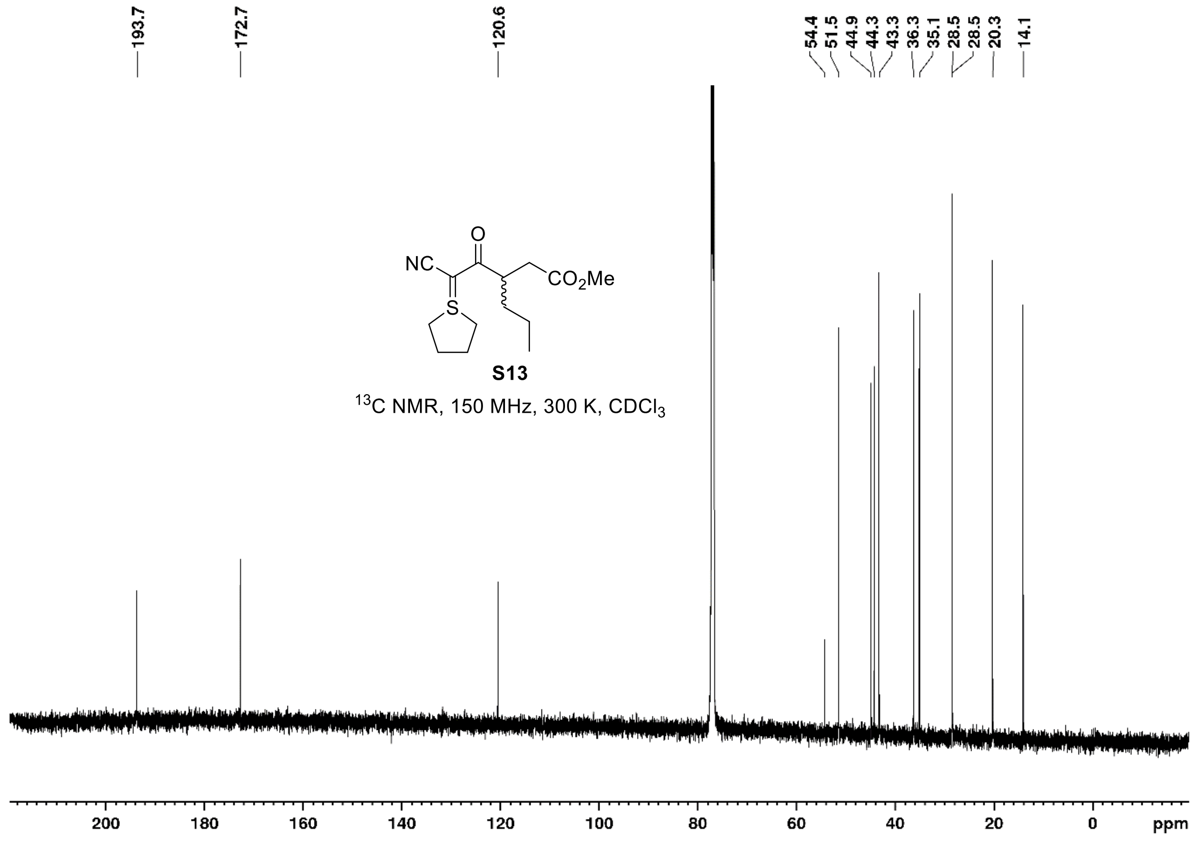
193.7
172.7
120.6
54.4 51.5 44.9 44.3 43.3 36.3 35.1 28.5 28.5 20.3 14.1
NC
O
CO2Me
S
S13
13C NMR, 150 MHz, 300 K, CDCl3
200 180 160 140 120 100 80 60 40 20 0 ppm

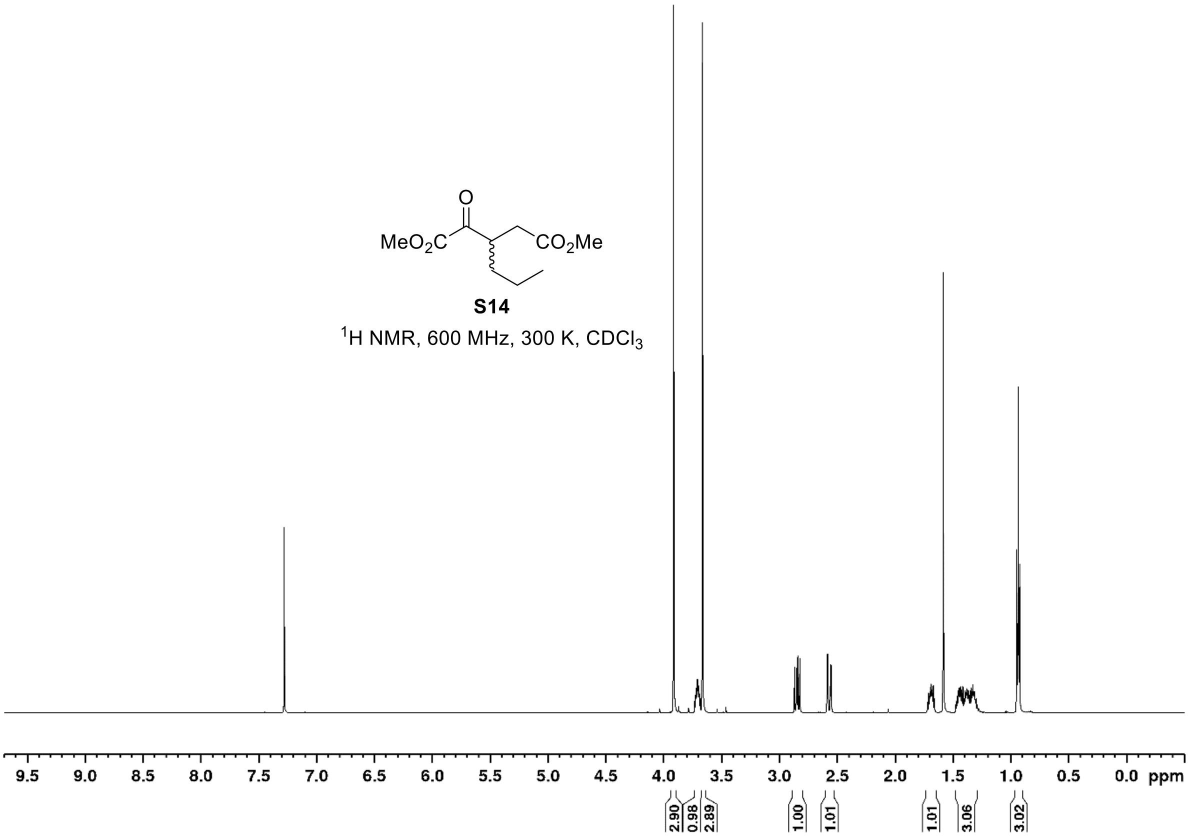
MeO2C
CO2Me
O
S14
1H NMR, 600 MHz, 300 K, CDCl3
9.5 9.0 8.5 8.0 7.5 7.0 6.5 6.0 5.5 5.0 4.5 4.0 3.5 3.0 2.5 2.0 1.5 1.0 0.5 0.0 ppm
2.90 0.98 2.89 1.00 1.01 1.01 3.06 3.02

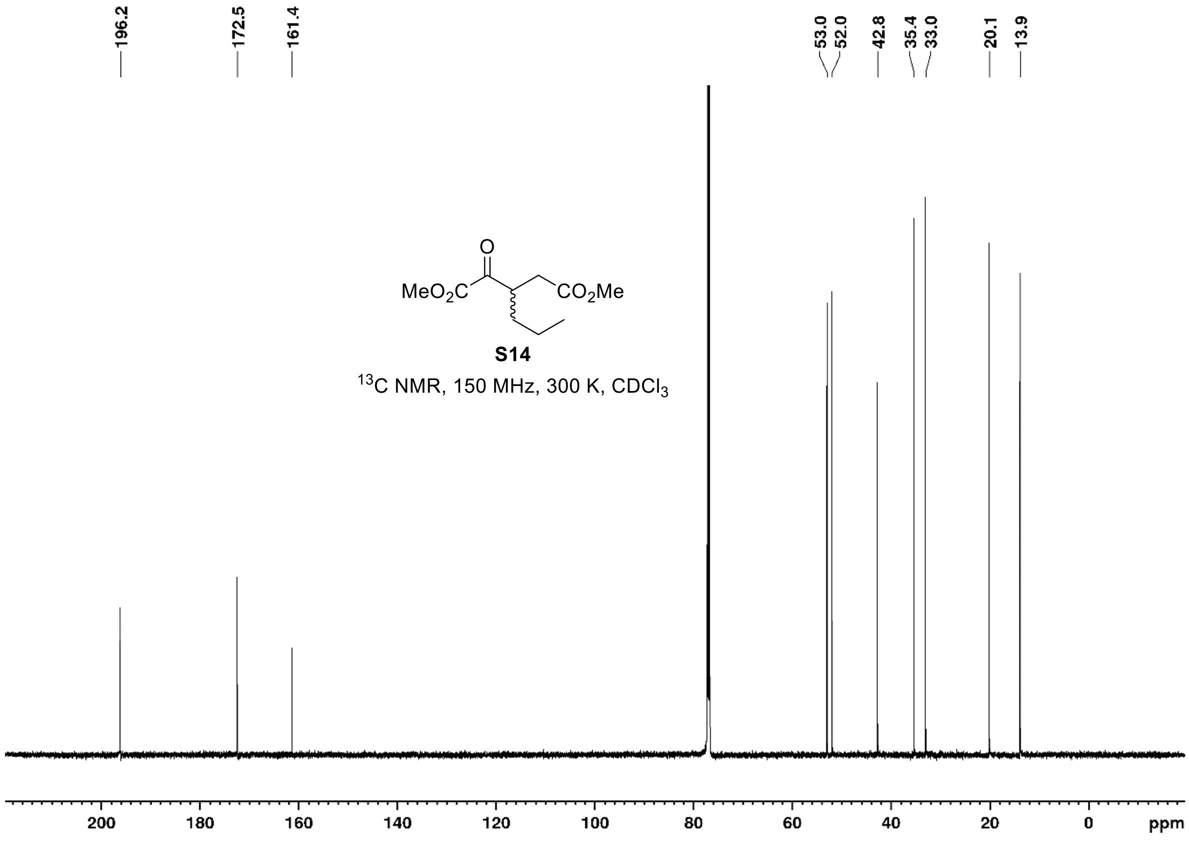
196.2
172.5
161.4
53.0
52.0
42.8
35.4
33.0
20.1
13.9
MeO2C
CO2Me
O
S14
13C NMR, 150 MHz, 300 K, CDCl3
200 180 160 140 120 100 80 60 40 20 0 ppm

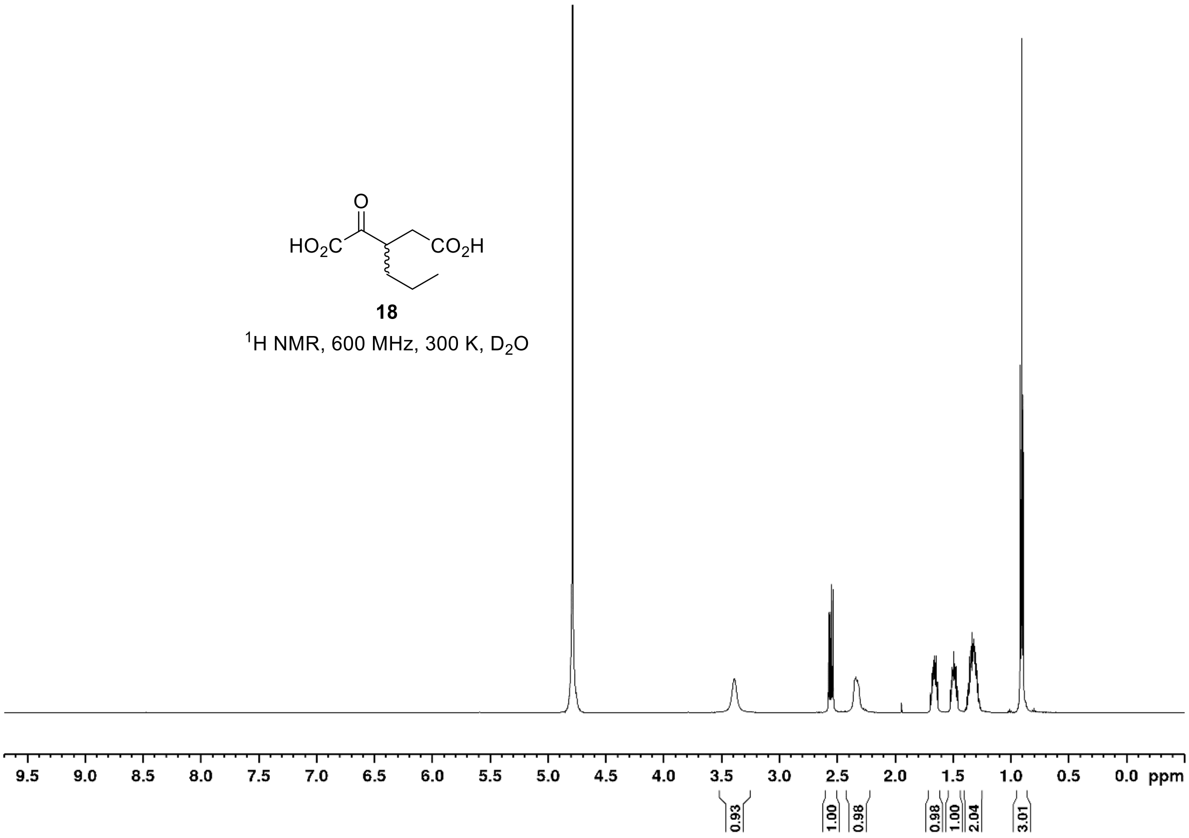

O
HO2C
CO2H
18
1H NMR, 600 MHz, 300 K, D2O
9.5 9.0 8.5 8.0 7.5 7.0 6.5 6.0 5.5 5.0 4.5 4.0 3.5 3.0 2.5 2.0 1.5 1.0 0.5 0.0 ppm
0.93
1.00
0.98
0.98
1.00
2.04
3.01

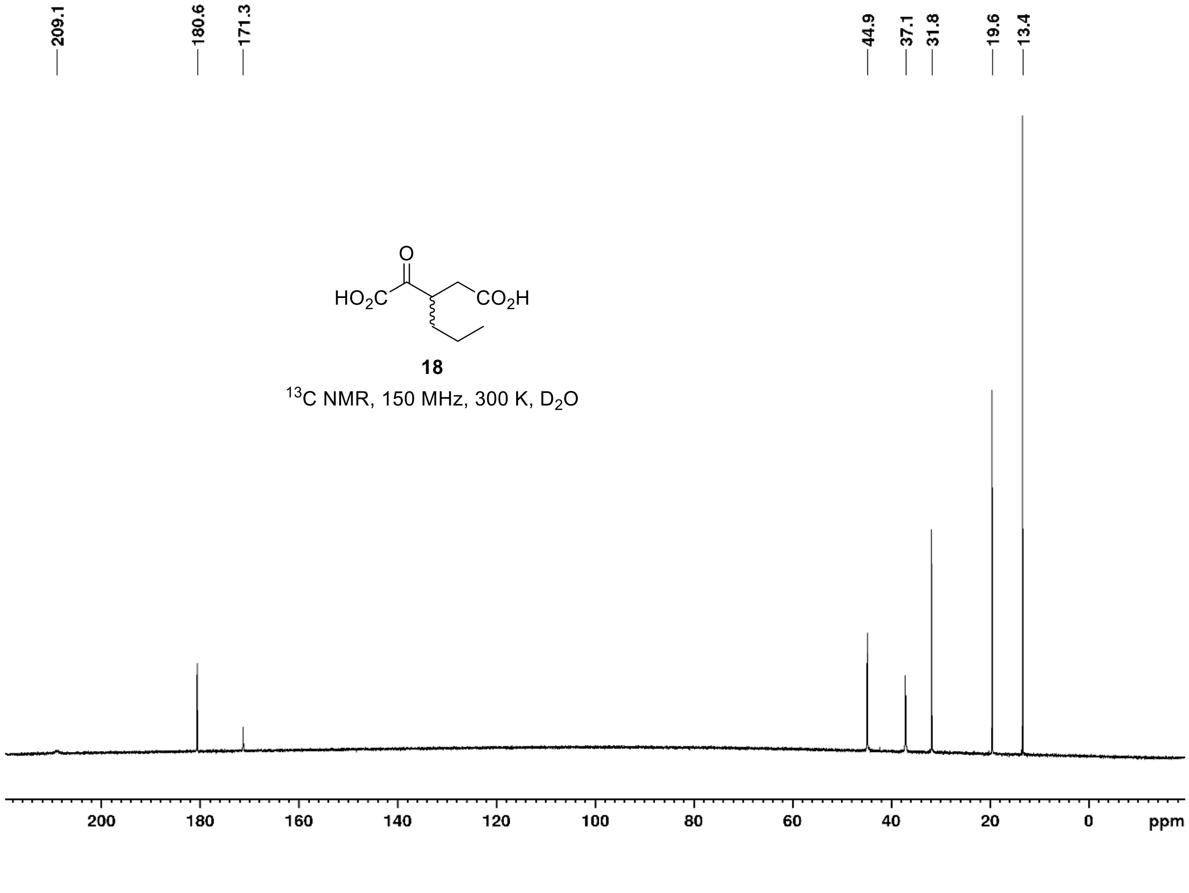

209.1
180.6
171.3
44.9
37.1
31.8
19.6
13.4
O
HO2C
CO2H
18
13C NMR, 150 MHz, 300 K, D2O
200 180 160 140 120 100 80 60 40 20 0 ppm

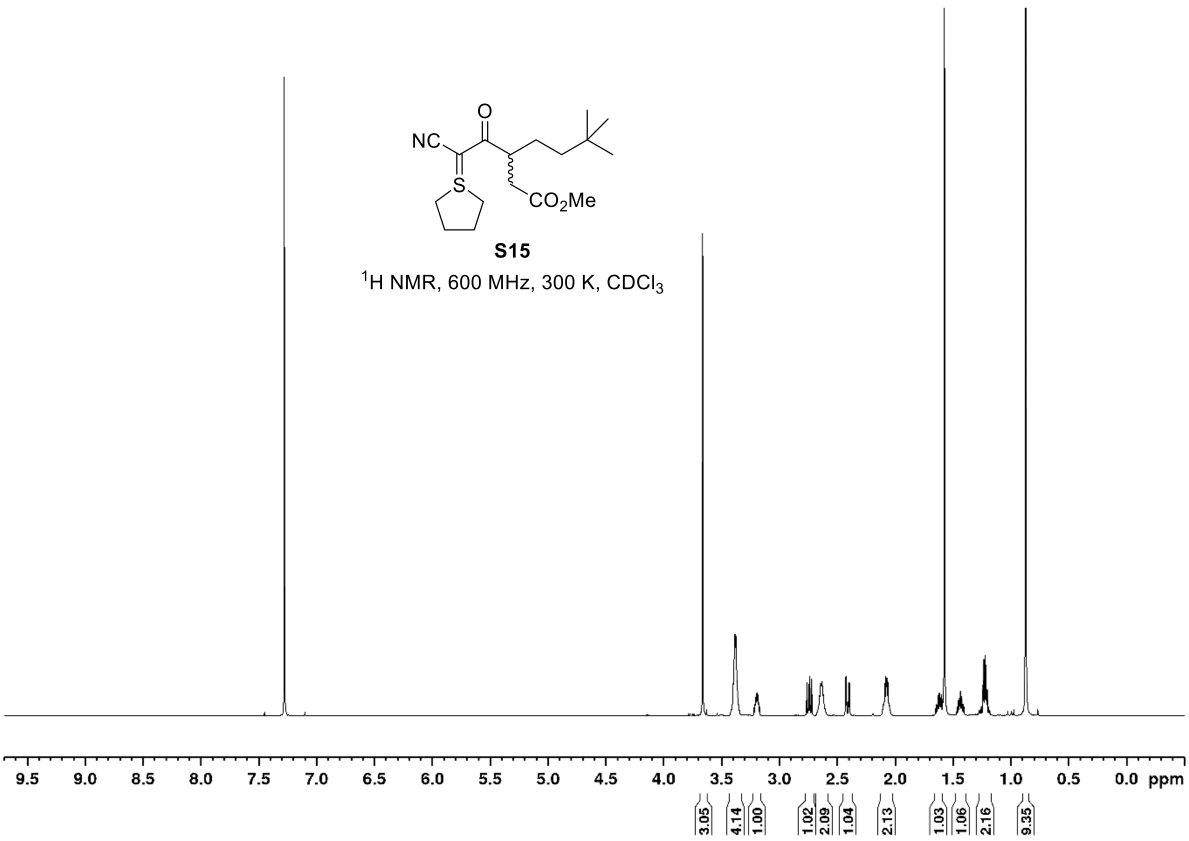
NC
O
S
CO2Me
S15
1H NMR, 600 MHz, 300 K, CDCl3
9.5 9.0 8.5 8.0 7.5 7.0 6.5 6.0 5.5 5.0 4.5 4.0 3.5 3.0 2.5 2.0 1.5 1.0 0.5 0.0 ppm
3.05 4.14 1.00 1.02 2.09 1.04 2.13 1.03 1.06 2.16 9.35

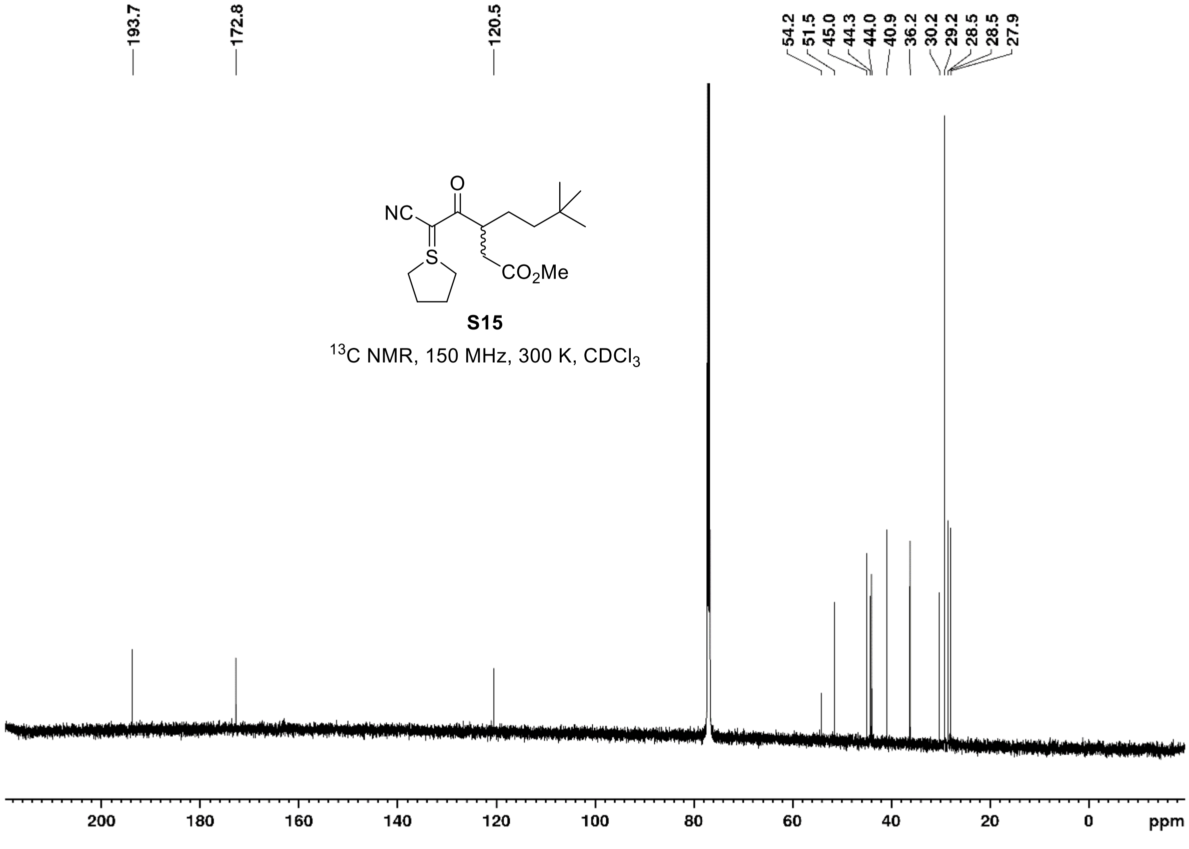
193.7
172.8
120.5
54.2 51.5 45.0 44.3 44.0 40.9 36.2 30.2 29.2 28.5 28.5 27.9
NC
O
S
CO2Me
S15
13C NMR, 150 MHz, 300 K, CDCl3
200 180 160 140 120 100 80 60 40 20 0 ppm

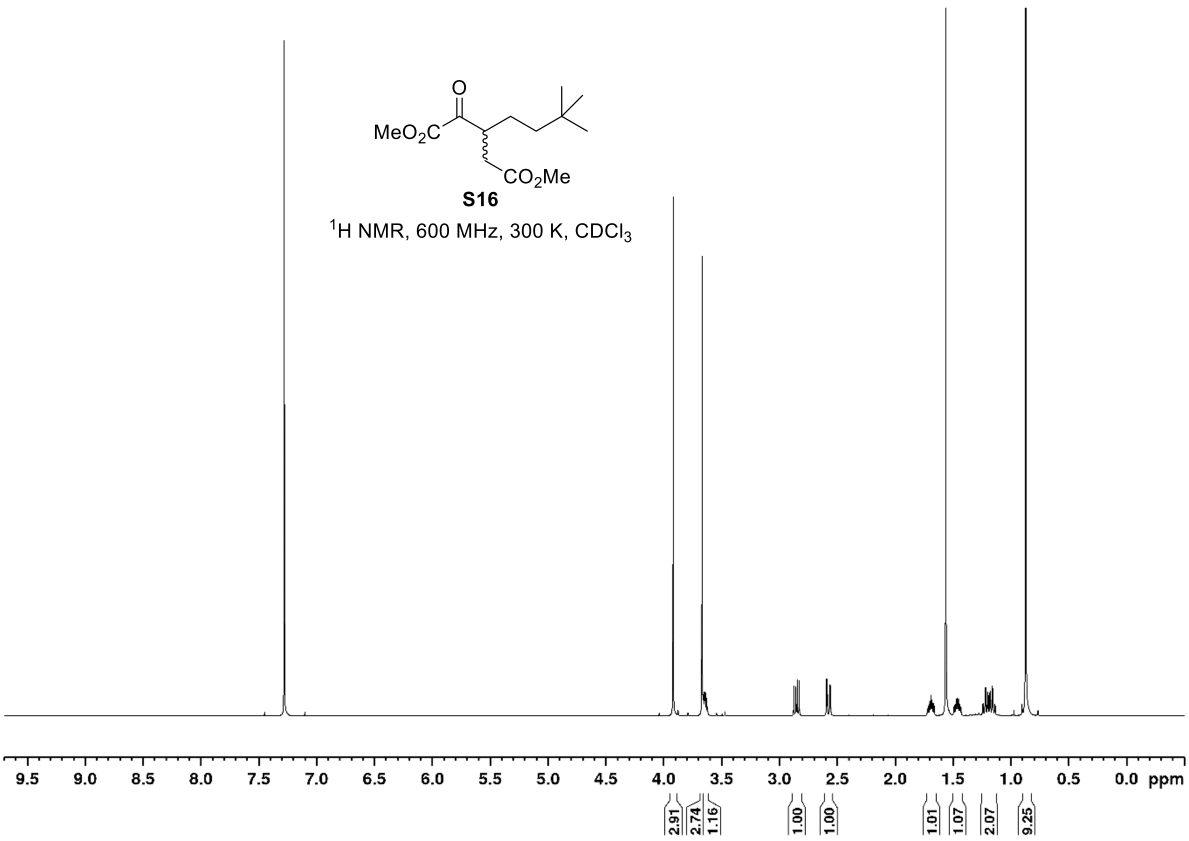
MeO2C
O
CO2Me
S16
1H NMR, 600 MHz, 300 K, CDCl3
9.5 9.0 8.5 8.0 7.5 7.0 6.5 6.0 5.5 5.0 4.5 4.0 3.5 3.0 2.5 2.0 1.5 1.0 0.5 0.0 ppm
2.91 2.74 1.16 1.00 1.00 1.01 1.07 2.07 9.25

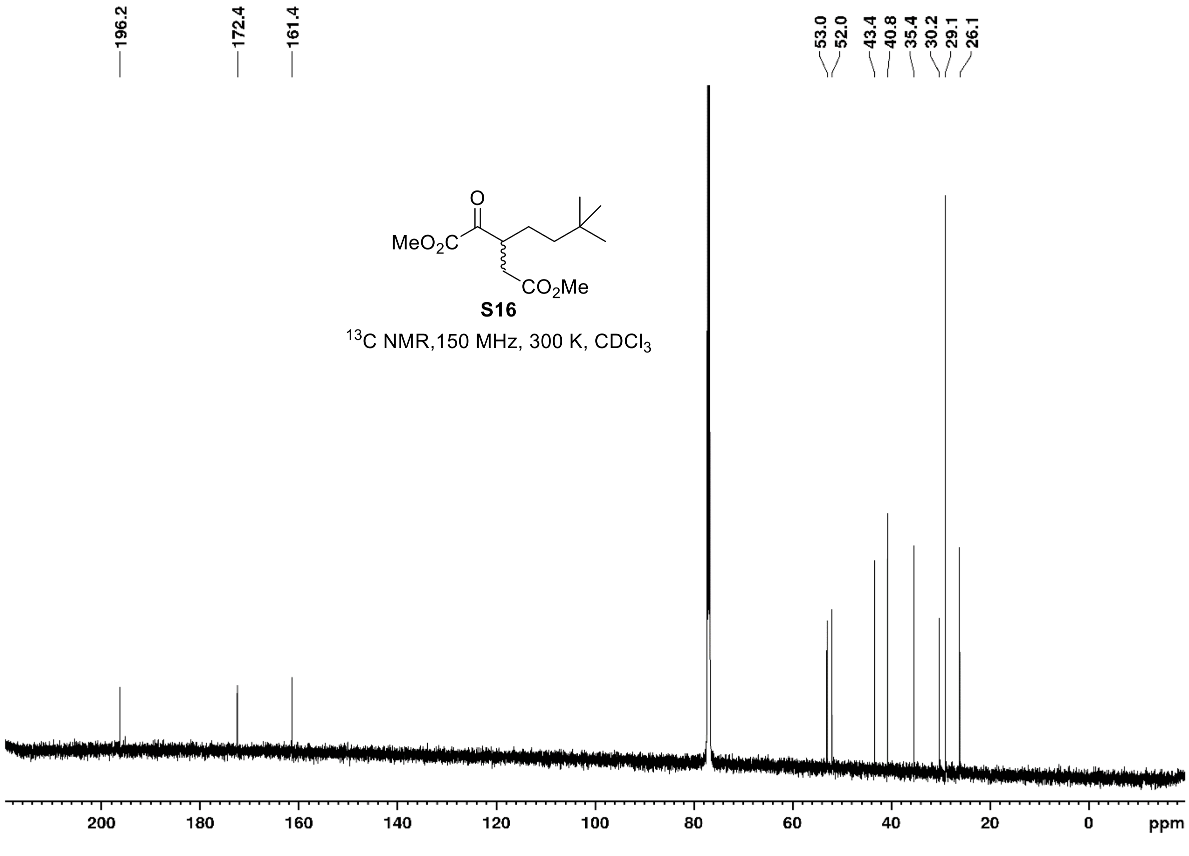
196.2
172.4
161.4
53.0 52.0 43.4 40.8 35.4 30.2 29.1 26.1
MeO2C
O
CO2Me
S16
13C NMR,150 MHz, 300 K, CDCl3
200 180 160 140 120 100 80 60 40 20 0 ppm

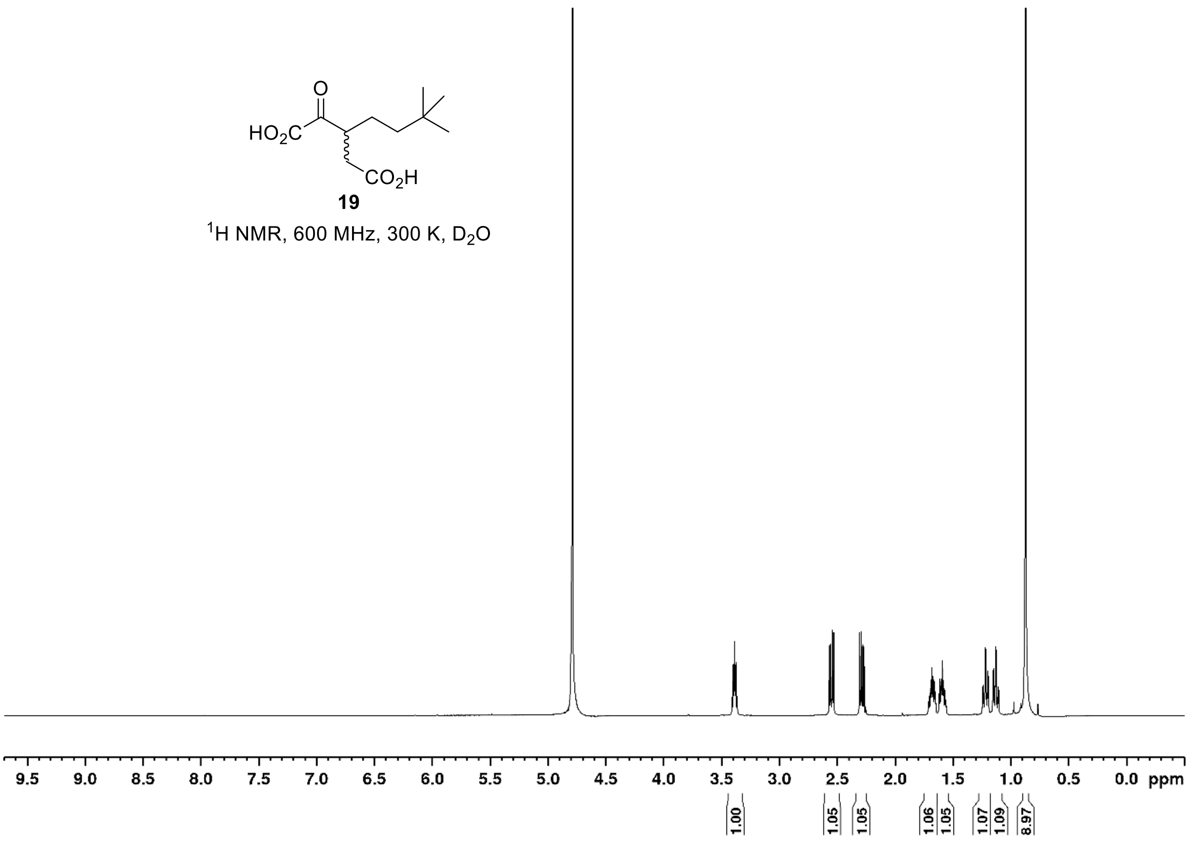
O
HO2C
CO2H
19
1H NMR, 600 MHz, 300 K, D2O
9.5 9.0 8.5 8.0 7.5 7.0 6.5 6.0 5.5 5.0 4.5 4.0 3.5 3.0 2.5 2.0 1.5 1.0 0.5 0.0 ppm
1.00 1.05 1.05 1.06 1.05 1.07 1.09 8.97

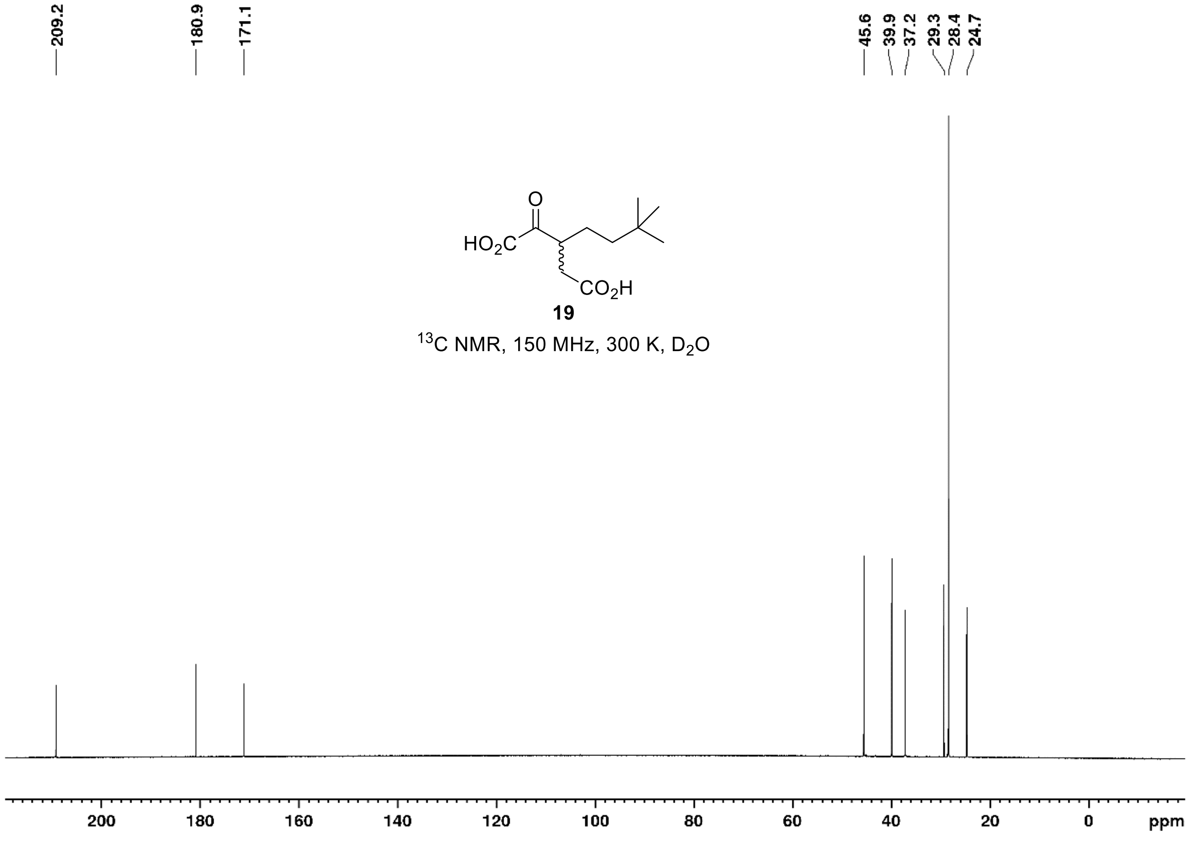
209.2
180.9
171.1
45.6
39.9
37.2
29.3
28.4
24.7
O
HO2C
CO2H
19
13C NMR, 150 MHz, 300 K, D2O
200 180 160 140 120 100 80 60 40 20 0 ppm

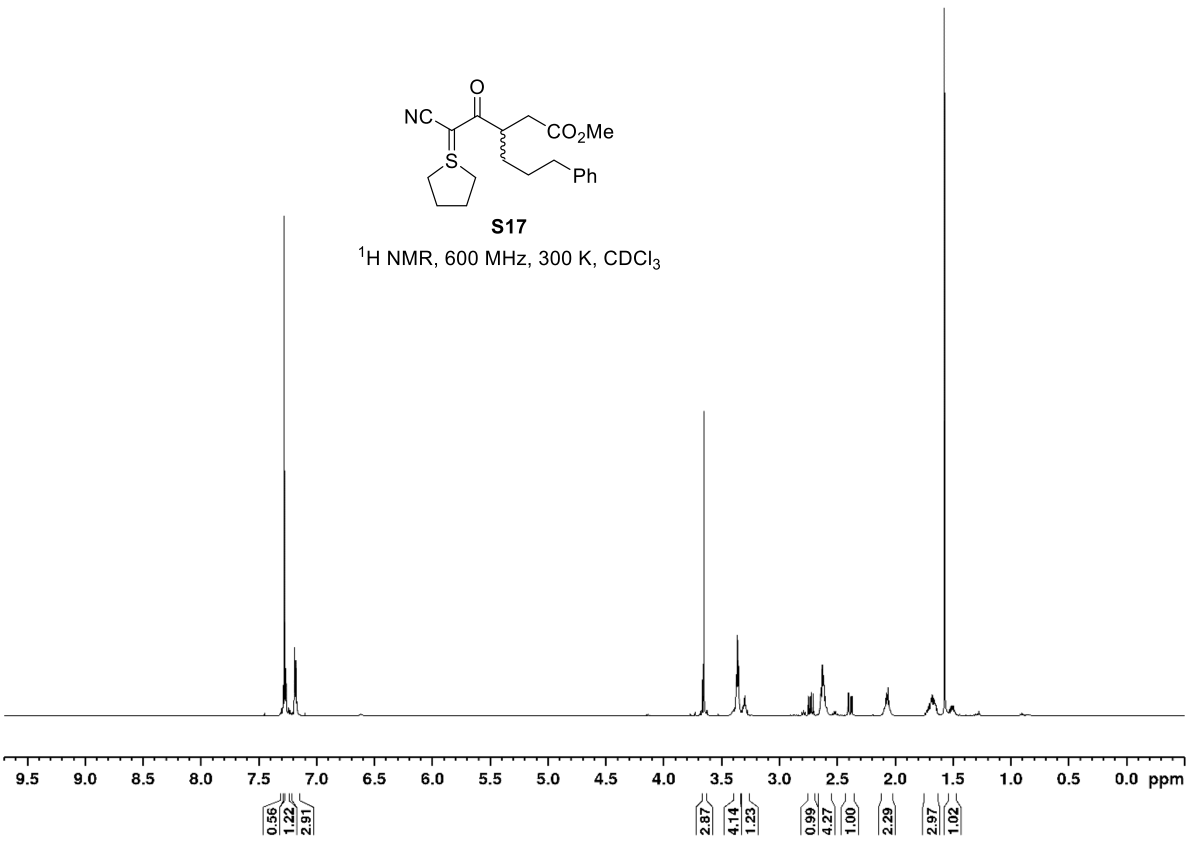
S17
^{1}H NMR, 600 MHz, 300 K, $CDCl_3$
9.5 9.0 8.5 8.0 7.5 7.0 6.5 6.0 5.5 5.0 4.5 4.0 3.5 3.0 2.5 2.0 1.5 1.0 0.5 0.0 ppm
0.56 1.22 2.91 2.87 4.14 1.23 0.99 4.27 1.00 2.29 2.97 1.02

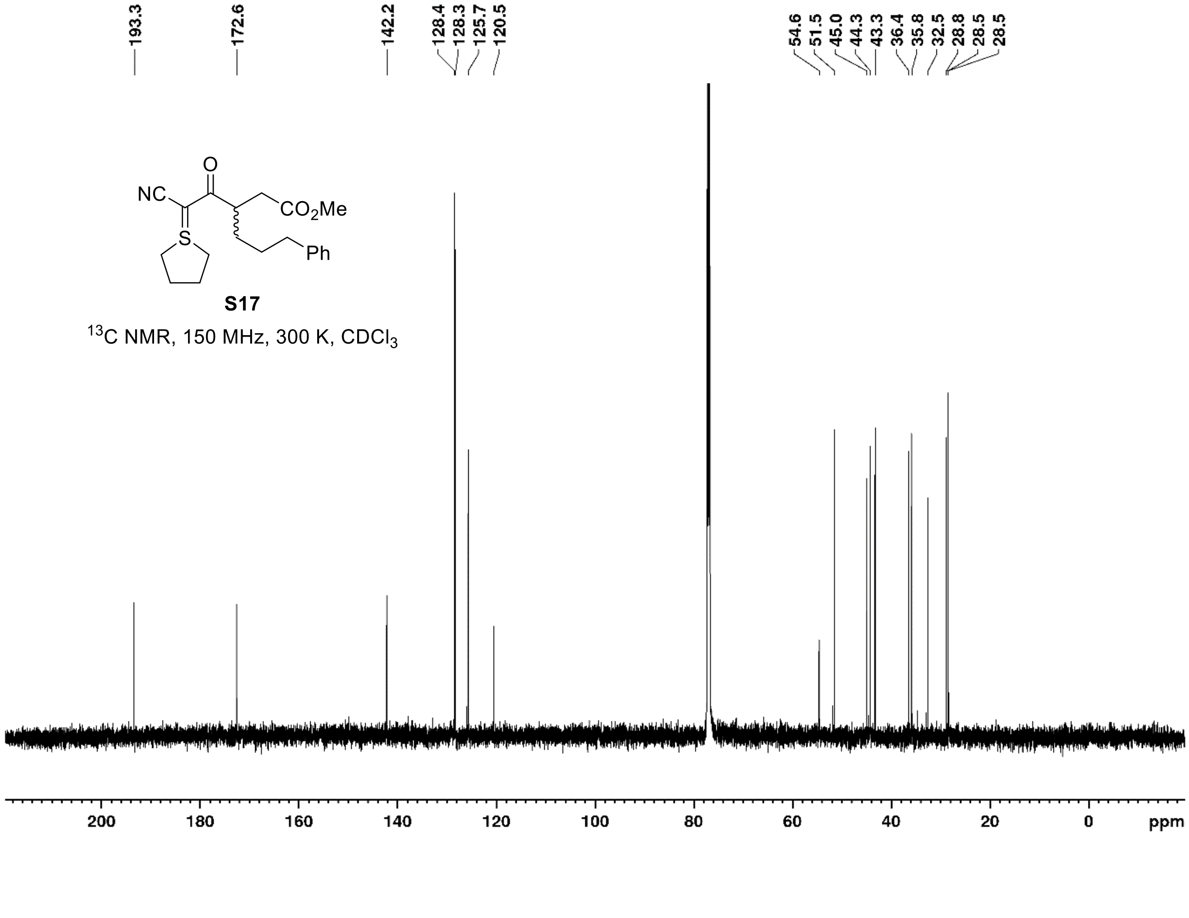
193.3 172.6 142.2 128.4 128.3 125.7 120.5 54.6 51.5 45.0 44.3 43.3 36.4 35.8 32.5 28.8 28.5 28.5
S17
^{13}C NMR, 150 MHz, 300 K, $CDCl_3$
200 180 160 140 120 100 80 60 40 20 0 ppm

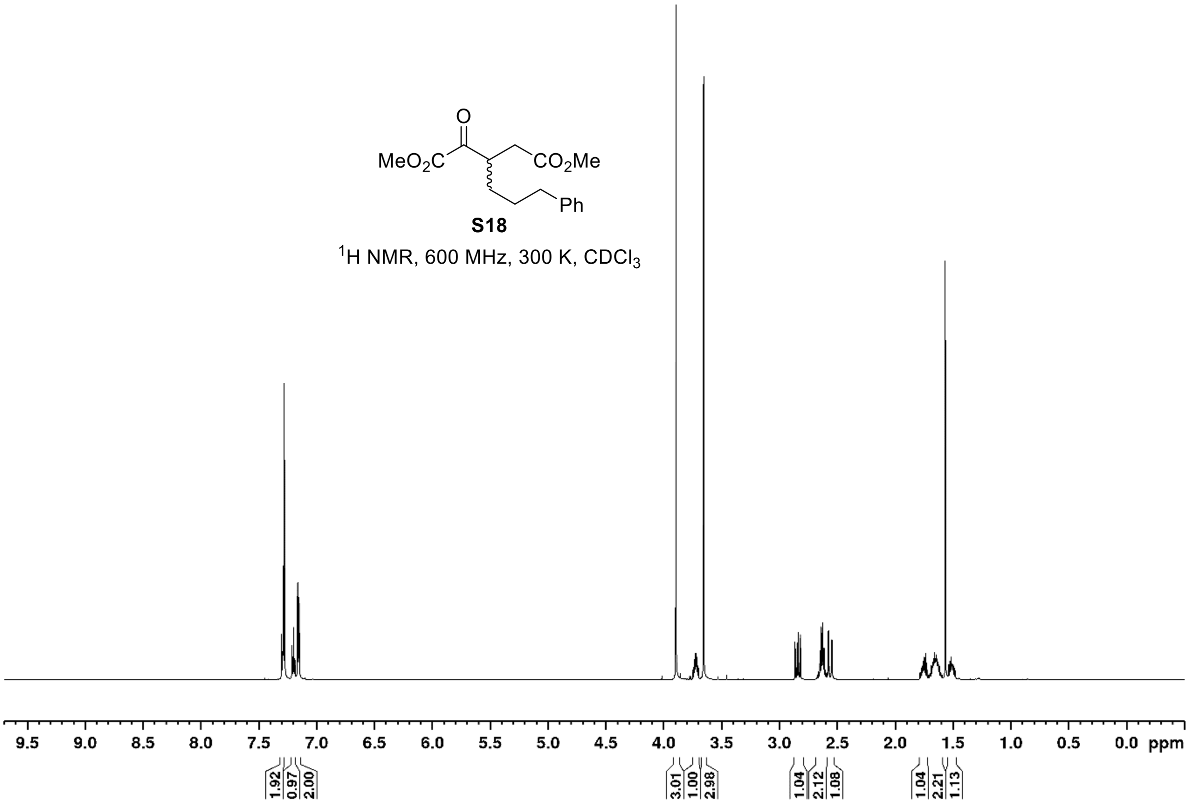
MeO2C
CO2Me
Ph
S18
1H NMR, 600 MHz, 300 K, CDCl3
9.5 9.0 8.5 8.0 7.5 7.0 6.5 6.0 5.5 5.0 4.5 4.0 3.5 3.0 2.5 2.0 1.5 1.0 0.5 0.0 ppm
1.92 0.97 2.00 3.01 1.00 2.98 1.04 2.12 1.08 1.04 2.21 1.13

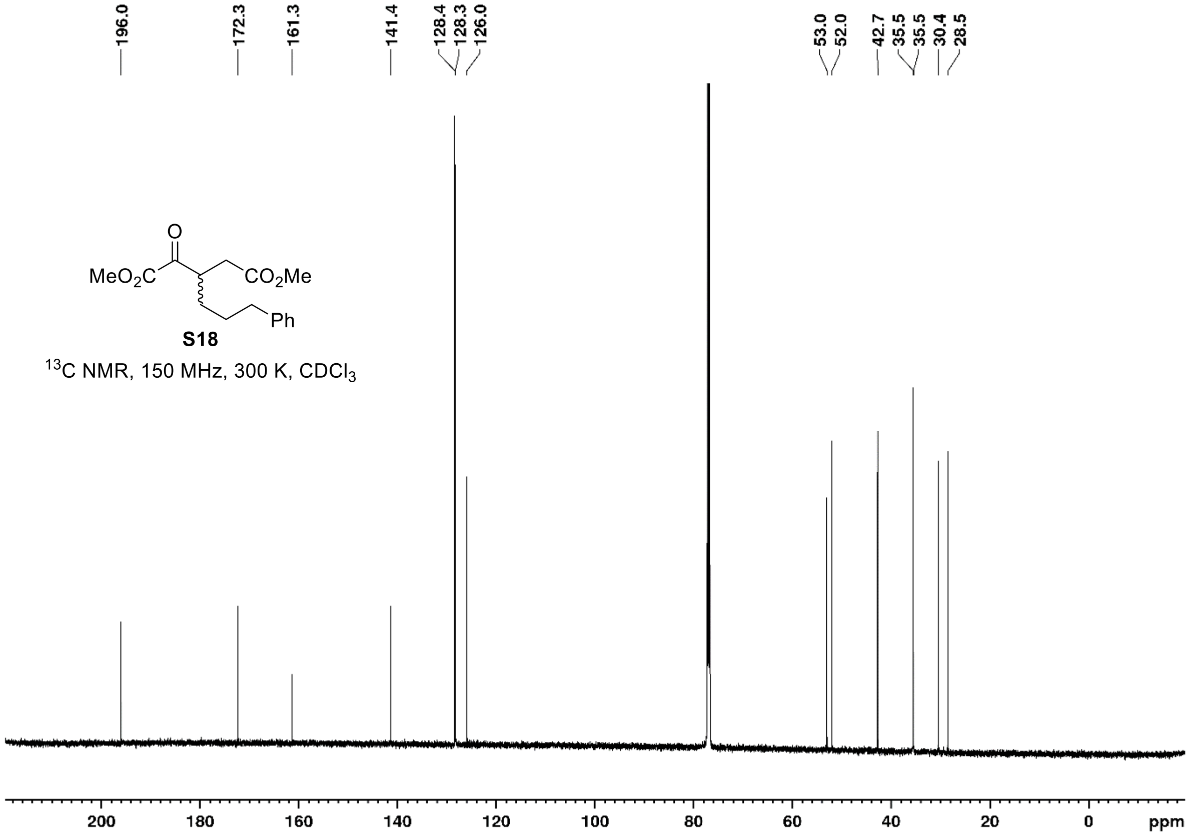
196.0 172.3 161.3 141.4 128.4 128.3 126.0 53.0 52.0 42.7 35.5 35.5 30.4 28.5
MeO2C
CO2Me
Ph
S18
13C NMR, 150 MHz, 300 K, CDCl3
200 180 160 140 120 100 80 60 40 20 0 ppm

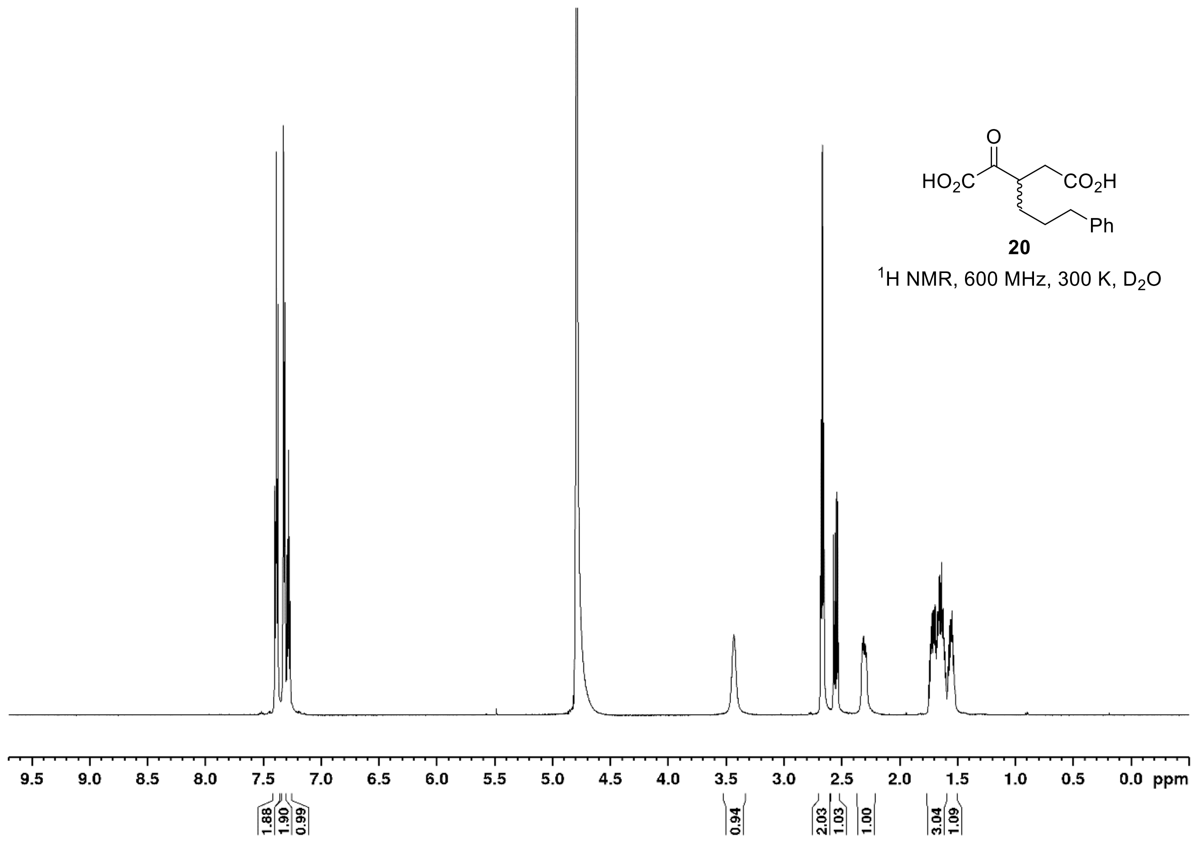
O
HO2C
CO2H
Ph
20
1H NMR, 600 MHz, 300 K, D2O
9.5 9.0 8.5 8.0 7.5 7.0 6.5 6.0 5.5 5.0 4.5 4.0 3.5 3.0 2.5 2.0 1.5 1.0 0.5 0.0 ppm
1.88 1.90 0.99 0.94 2.03 1.03 1.00 3.04 1.09

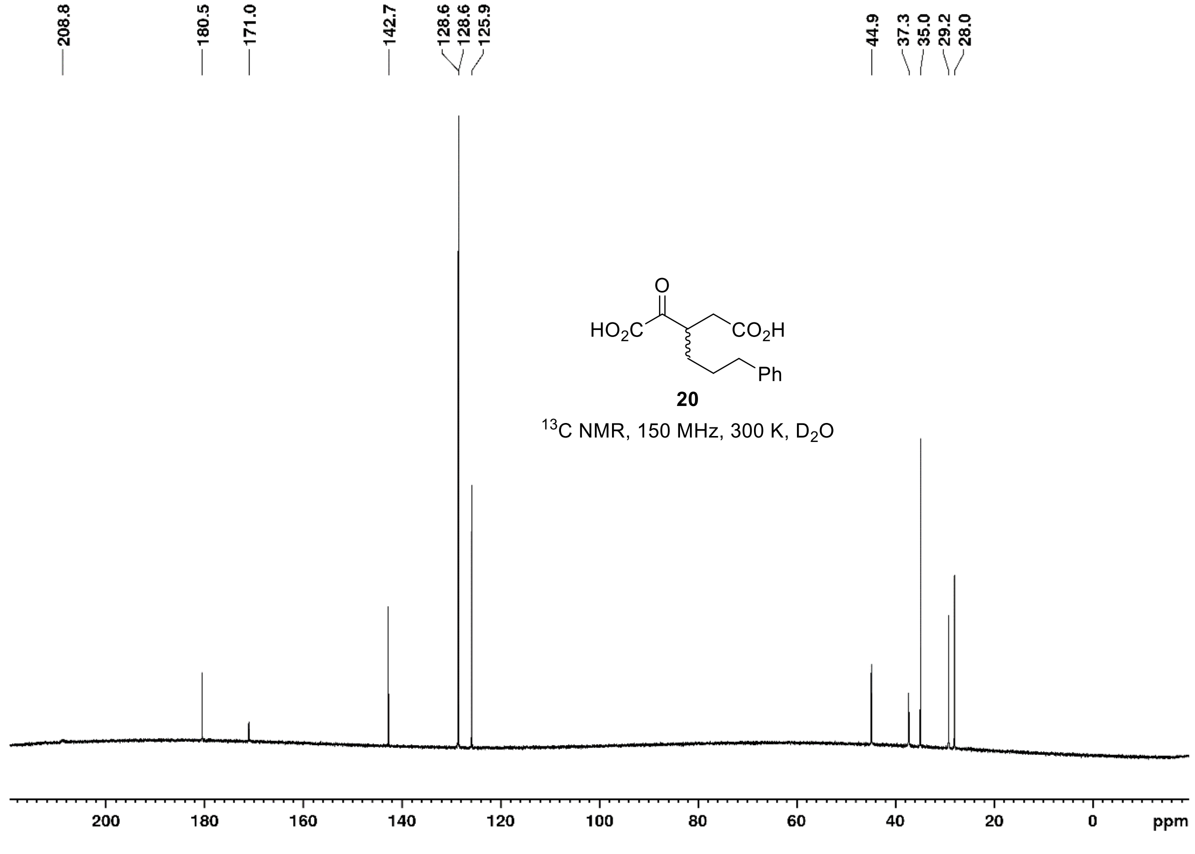
208.8 180.5 171.0 142.7 128.6 128.6 125.9 44.9 37.3 35.0 29.2 28.0
O
HO2C
CO2H
Ph
20
13C NMR, 150 MHz, 300 K, D2O
200 180 160 140 120 100 80 60 40 20 0 ppm

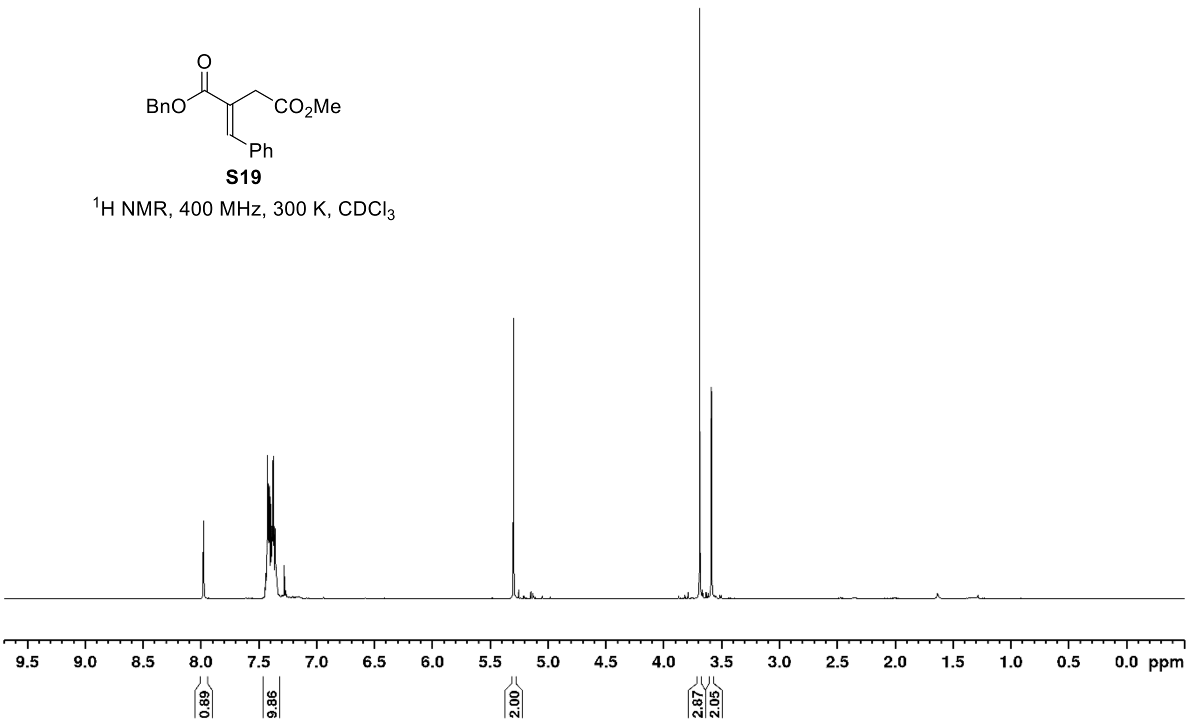
O
BnO
CO2Me
Ph
S19
1H NMR, 400 MHz, 300 K, CDCl3
9.5 9.0 8.5 8.0 7.5 7.0 6.5 6.0 5.5 5.0 4.5 4.0 3.5 3.0 2.5 2.0 1.5 1.0 0.5 0.0 ppm
0.89
9.86
2.00
2.87
2.05

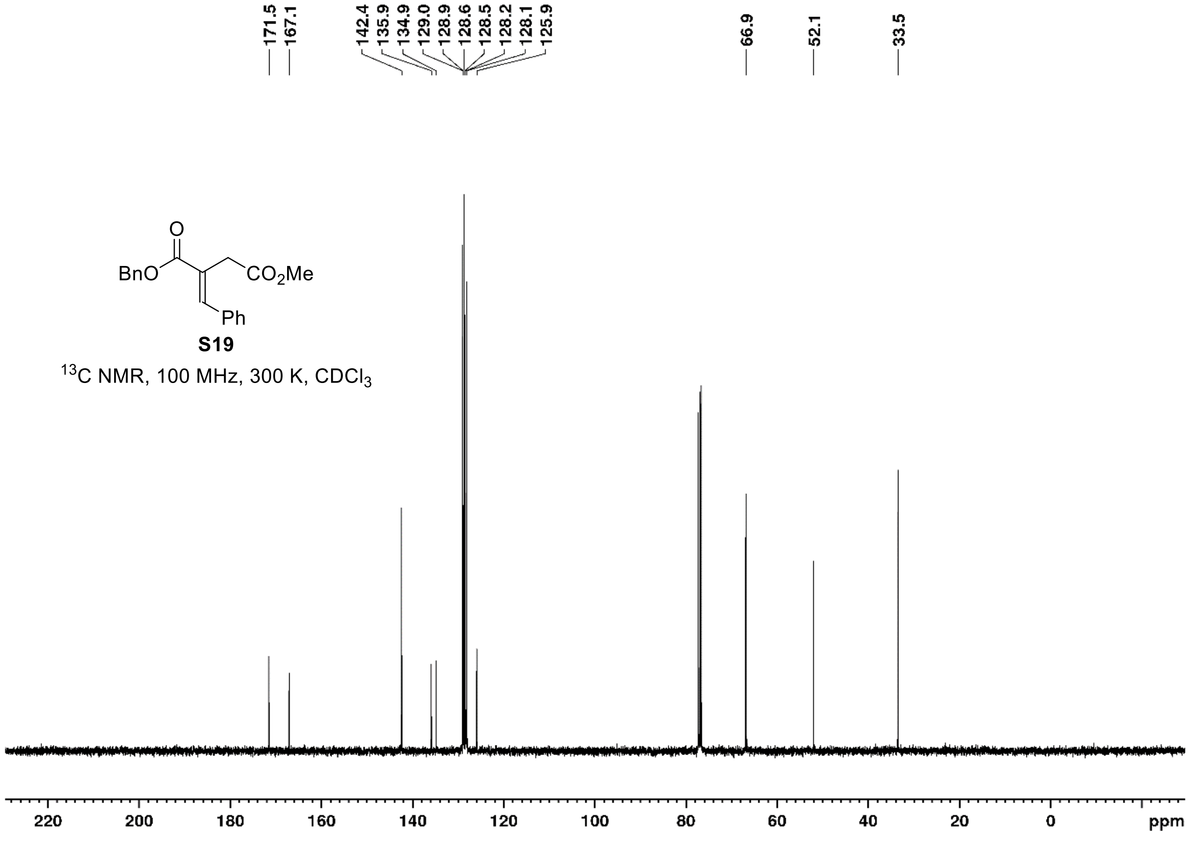
171.5
167.1
142.4
135.9
134.9
129.0
128.9
128.6
128.5
128.2
128.1
125.9
66.9
52.1
33.5
O
BnO
CO2Me
Ph
S19
13C NMR, 100 MHz, 300 K, CDCl3
220 200 180 160 140 120 100 80 60 40 20 0 ppm

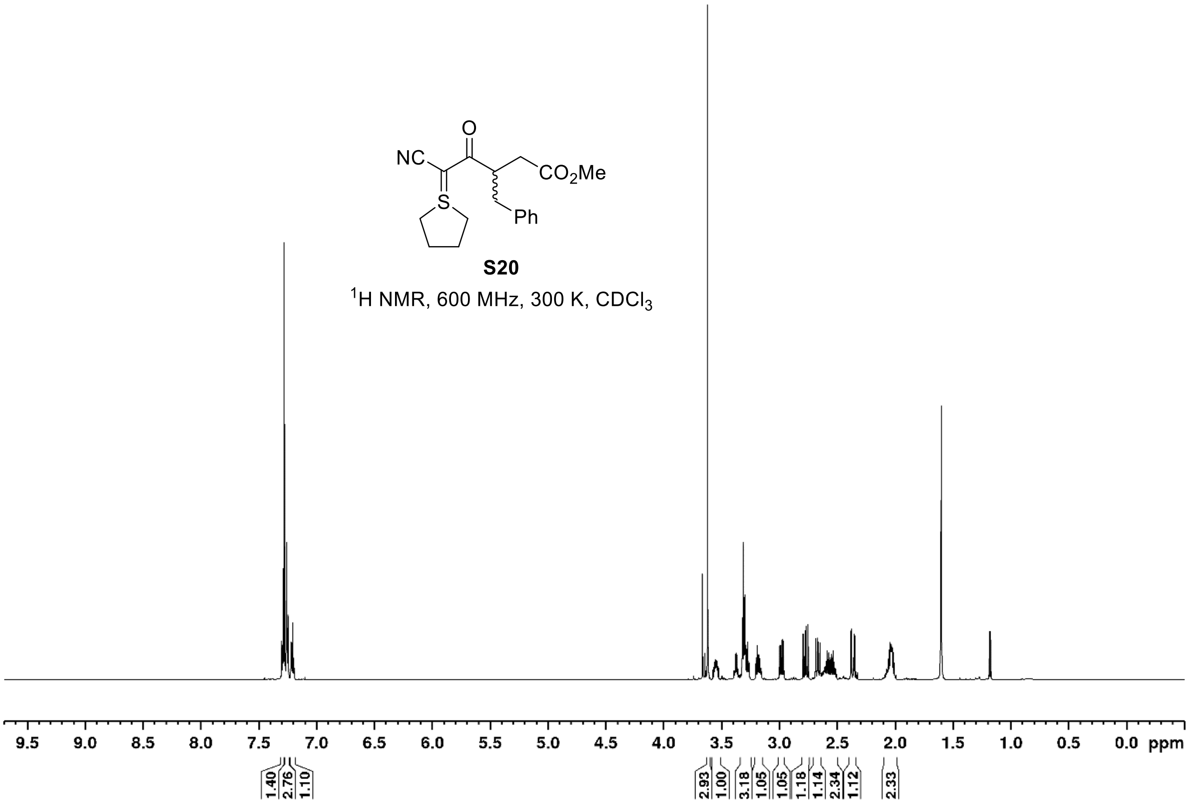
NC
O
CO2Me
S
Ph
S20
1H NMR, 600 MHz, 300 K, CDCl3
9.5 9.0 8.5 8.0 7.5 7.0 6.5 6.0 5.5 5.0 4.5 4.0 3.5 3.0 2.5 2.0 1.5 1.0 0.5 0.0 ppm
1.40 2.76 1.10
2.93 1.00 3.18 1.05 1.18 1.14 2.34 1.12 2.33

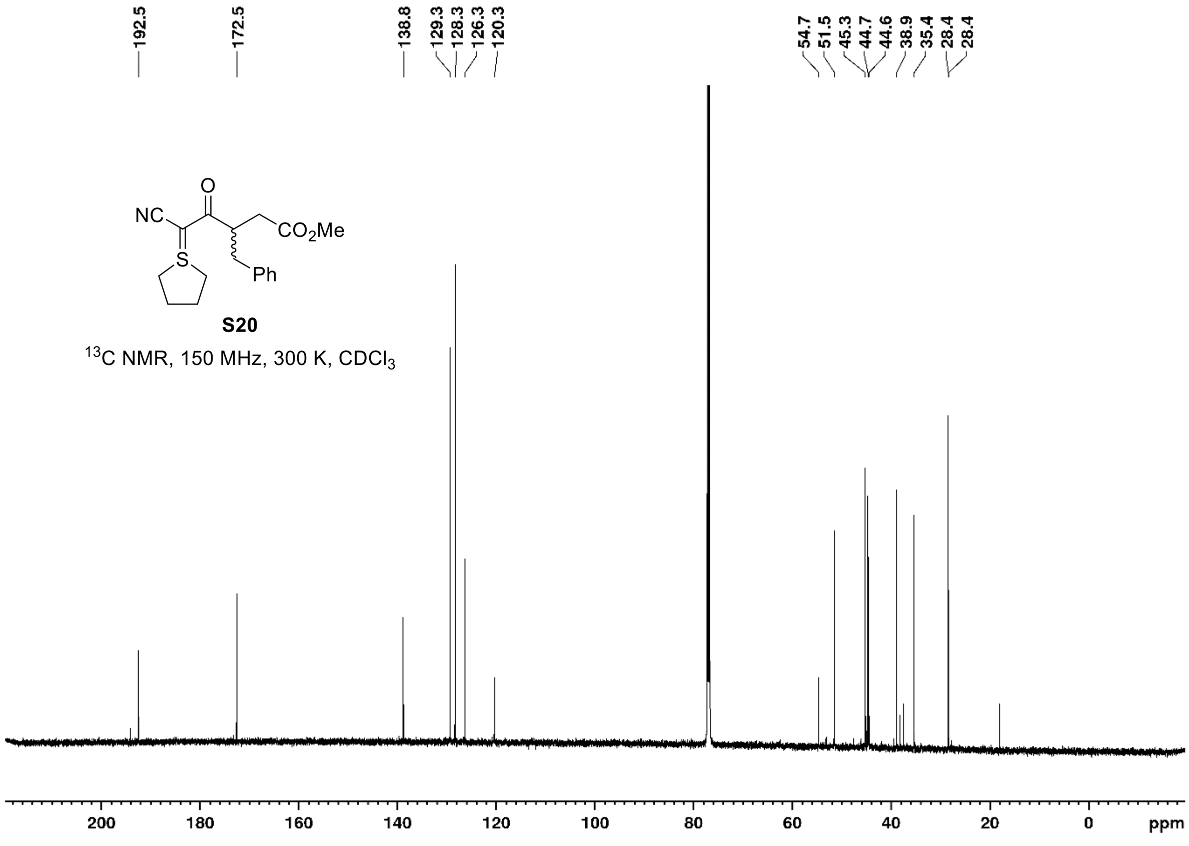
192.5
172.5
138.8 129.3 128.3 126.3 120.3
54.7 51.5 45.3 44.7 44.6 38.9 35.4 28.4 28.4
NC
O
CO2Me
S
Ph
S20
13C NMR, 150 MHz, 300 K, CDCl3
200 180 160 140 120 100 80 60 40 20 0 ppm

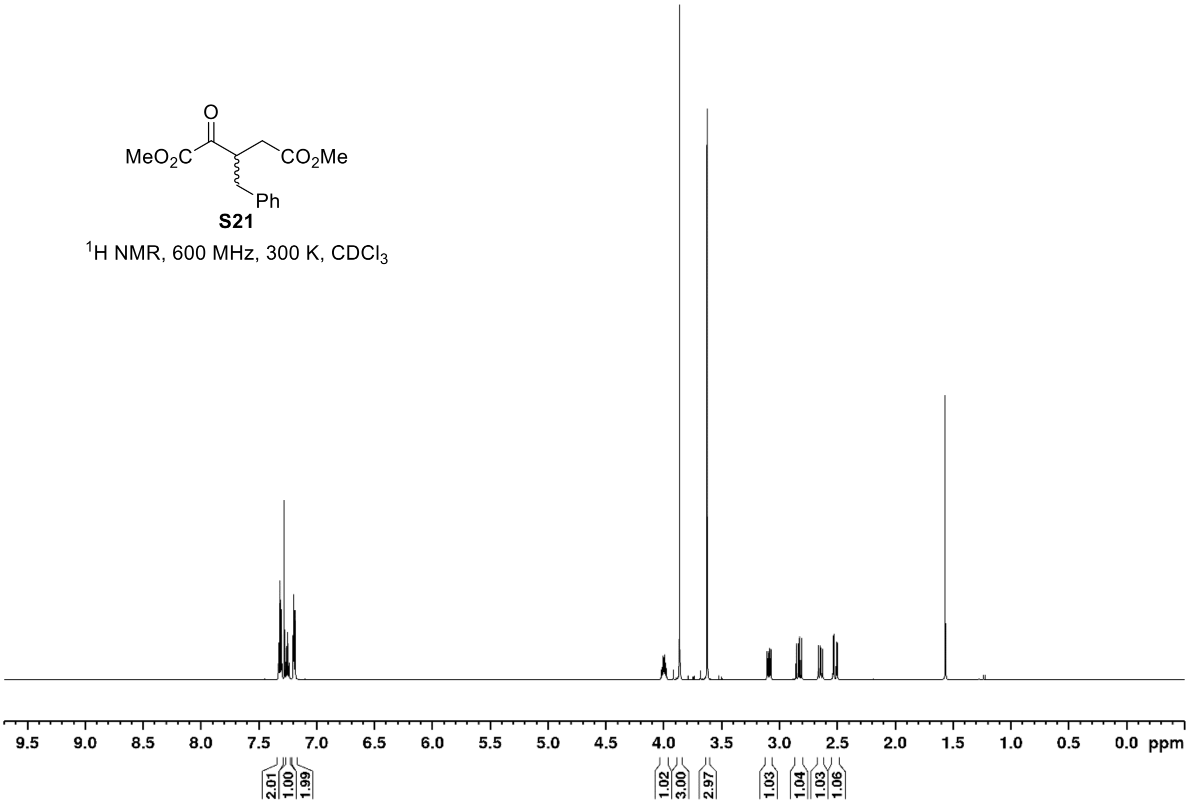
MeO2C
CO2Me
Ph
S21
1H NMR, 600 MHz, 300 K, CDCl3
9.5 9.0 8.5 8.0 7.5 7.0 6.5 6.0 5.5 5.0 4.5 4.0 3.5 3.0 2.5 2.0 1.5 1.0 0.5 0.0 ppm
2.01 1.00 1.99
1.02 3.00 2.97
1.03 1.04 1.03 1.06

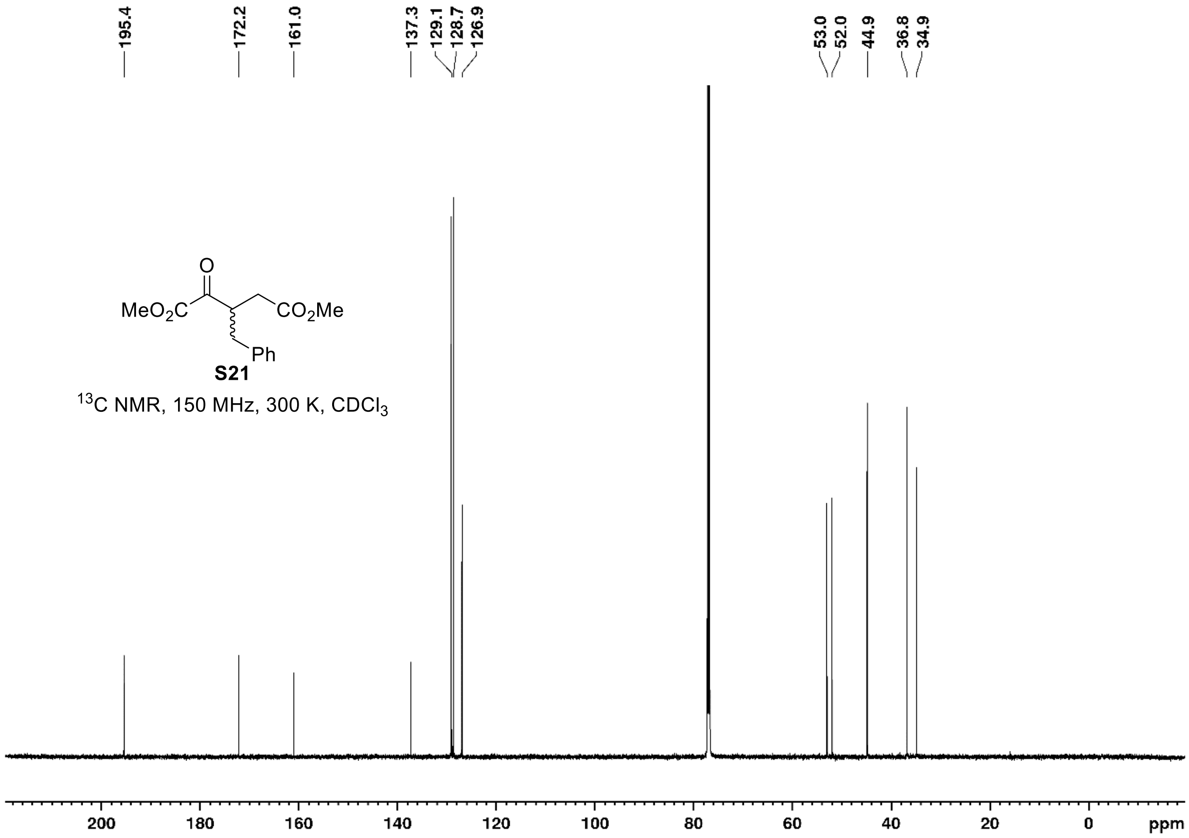
195.4
172.2
161.0
137.3 129.1 128.7 126.9
53.0 52.0
44.9
36.8 34.9
MeO2C
CO2Me
Ph
S21
13C NMR, 150 MHz, 300 K, CDCl3
200 180 160 140 120 100 80 60 40 20 0 ppm

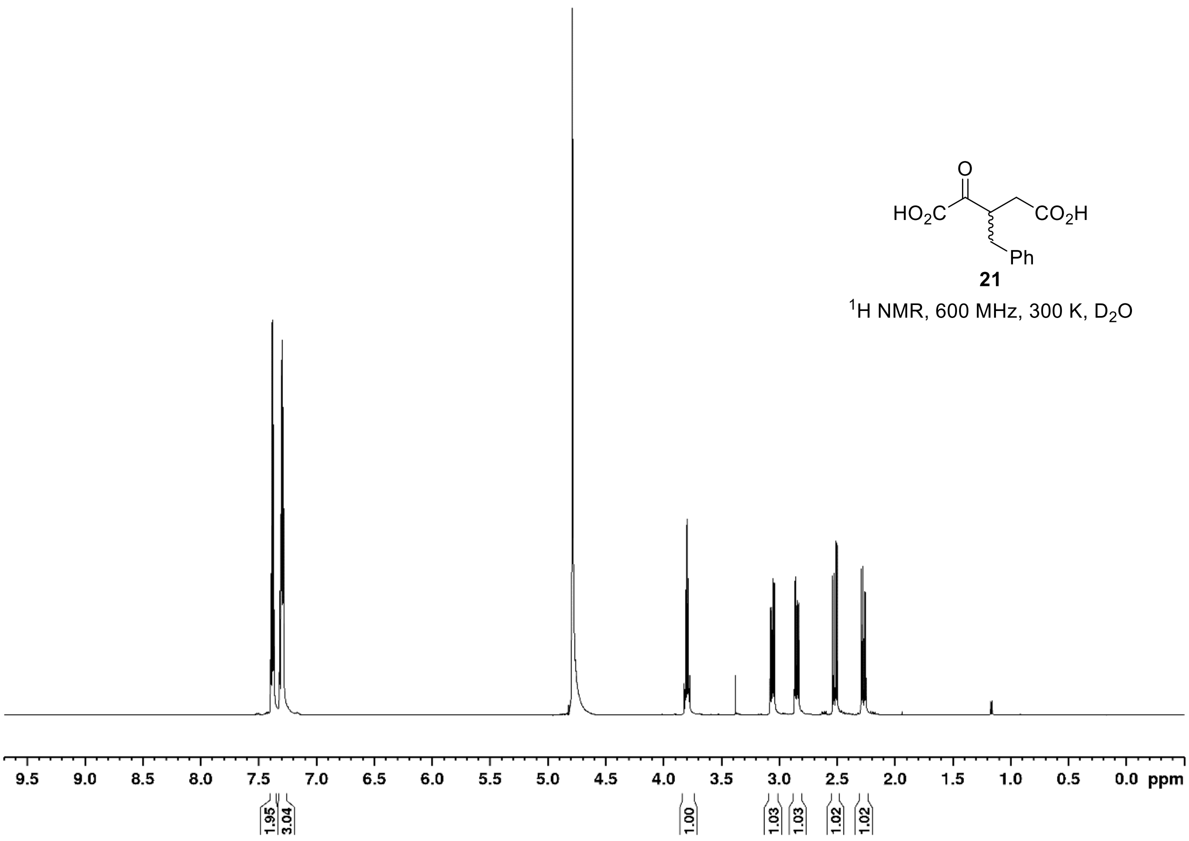
O
HO_2C
CO_2H
Ph
21
^{1}H NMR, 600 MHz, 300 K, D_2O
9.5 9.0 8.5 8.0 7.5 7.0 6.5 6.0 5.5 5.0 4.5 4.0 3.5 3.0 2.5 2.0 1.5 1.0 0.5 0.0 ppm
1.95 3.04 1.00 1.03 1.03 1.02 1.02

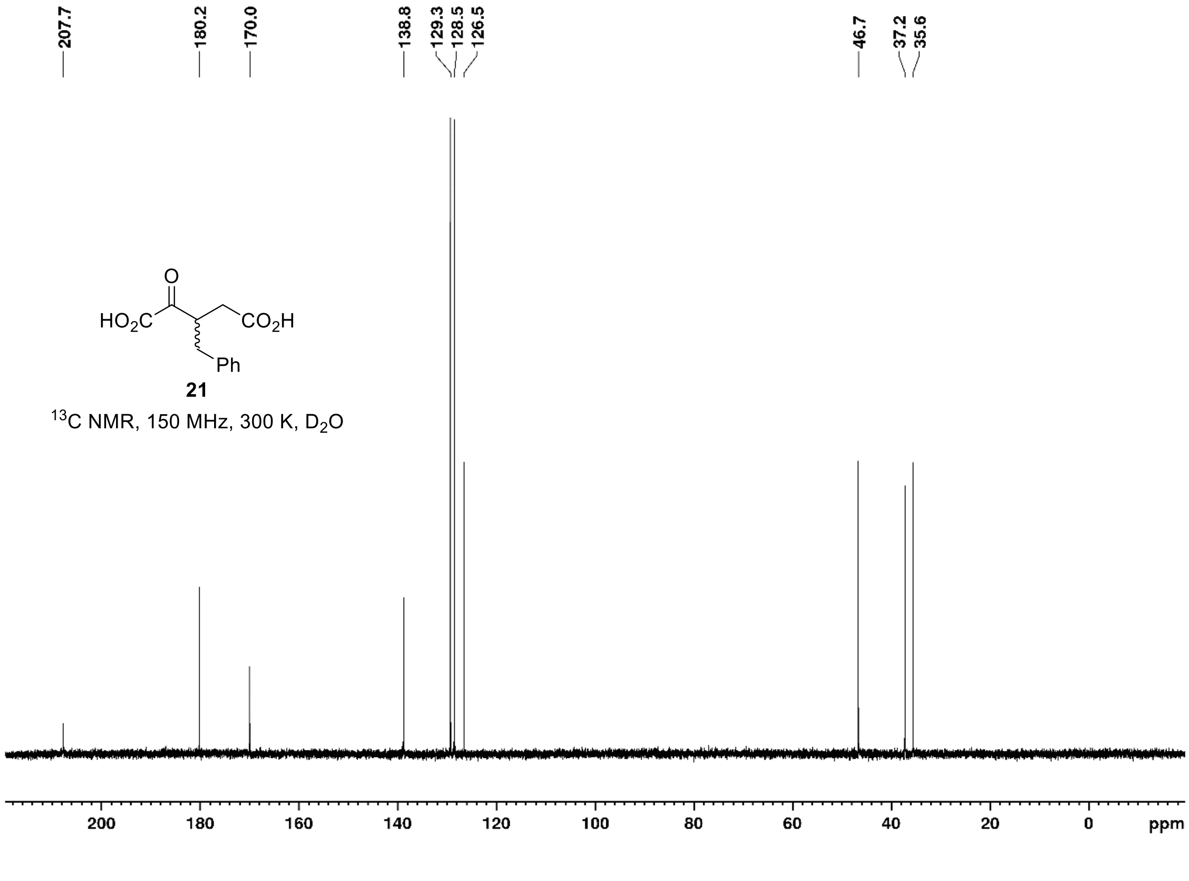
207.7 180.2 170.0 138.8 129.3 128.5 126.5 46.7 37.2 35.6
O
HO_2C
CO_2H
Ph
21
^{13}C NMR, 150 MHz, 300 K, D_2O
200 180 160 140 120 100 80 60 40 20 0 ppm

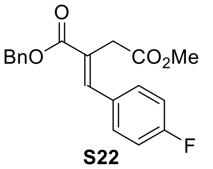

S22

^{1}H NMR, 600 MHz, 300 K, $CDCl_3$

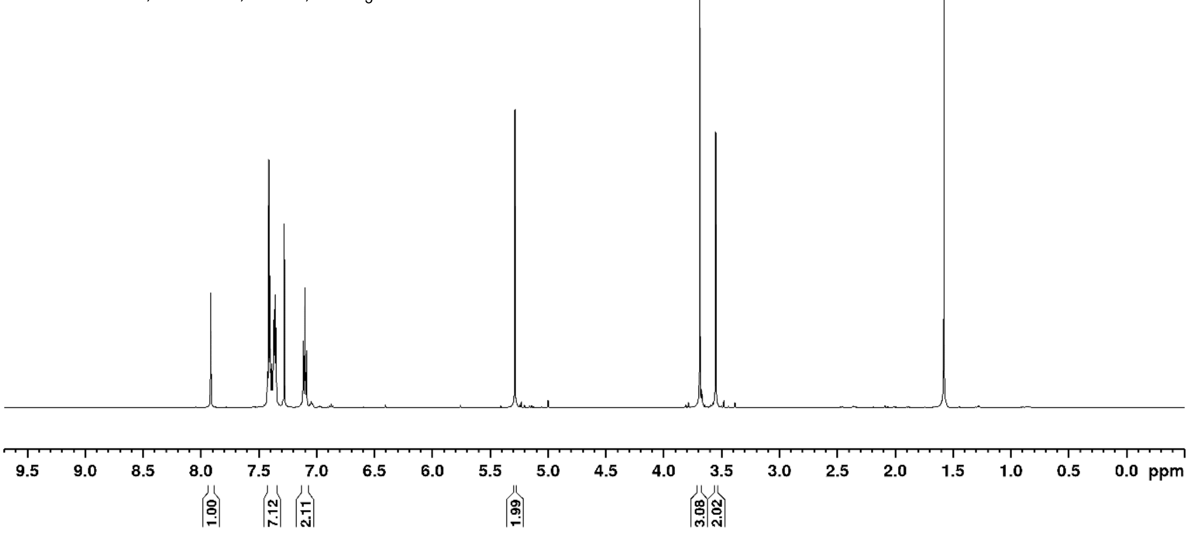

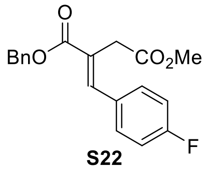

S22

^{13}C NMR, 150 MHz, 300 K, $CDCl_3$

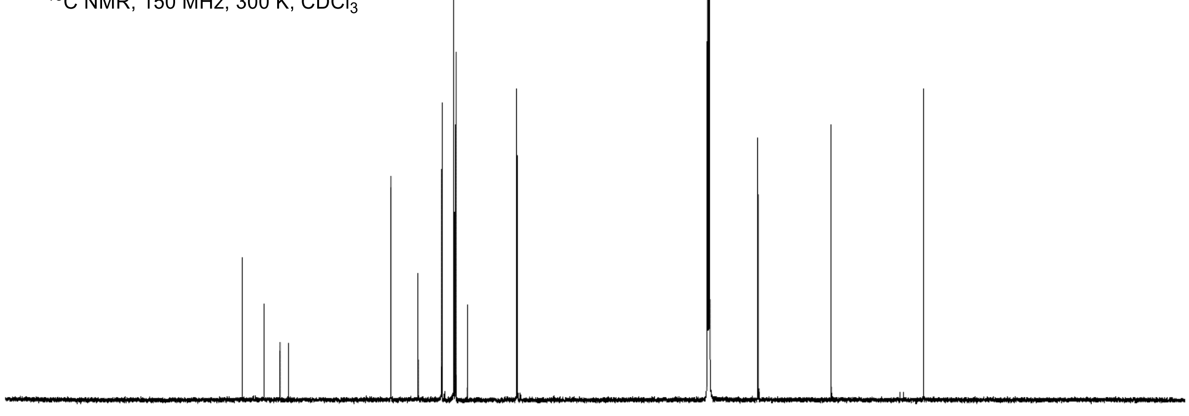

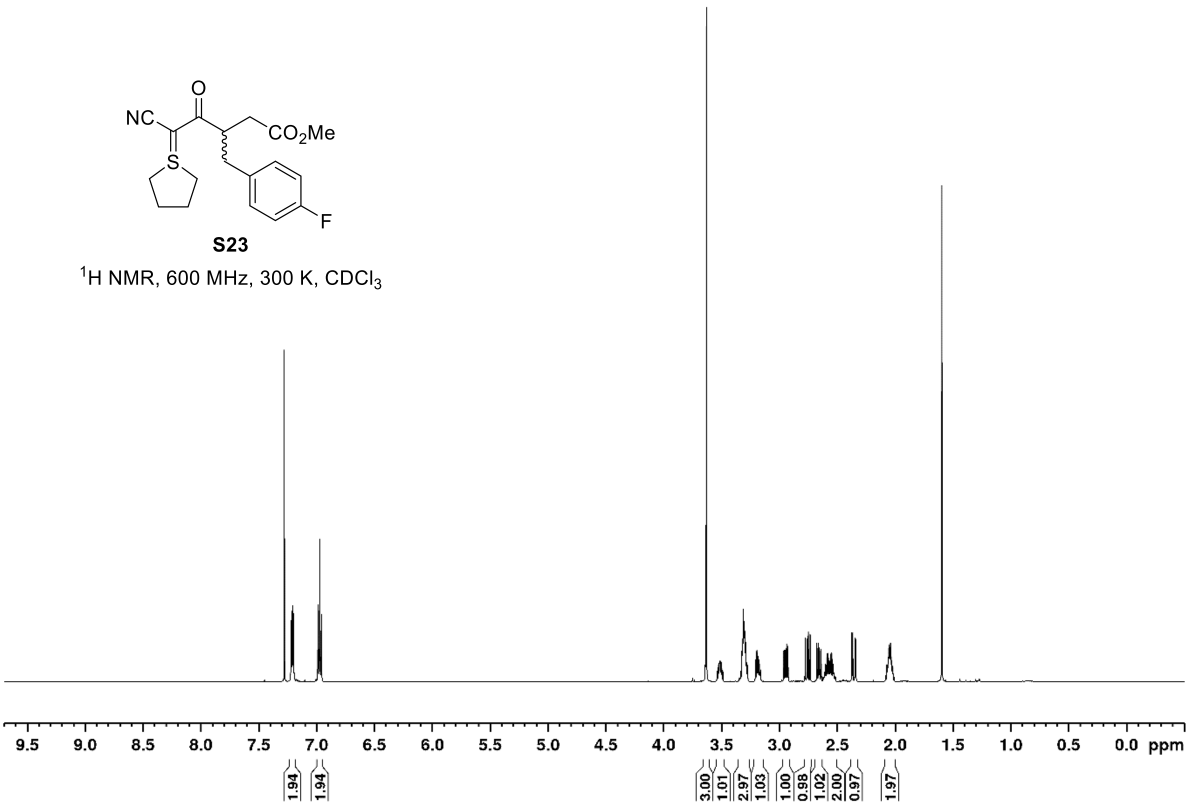

NC
O
CO2Me
S
F
S23
1H NMR, 600 MHz, 300 K, CDCl3
9.5 9.0 8.5 8.0 7.5 7.0 6.5 6.0 5.5 5.0 4.5 4.0 3.5 3.0 2.5 2.0 1.5 1.0 0.5 0.0 ppm
1.94
1.94
3.00
1.01
2.97
1.03
1.00
0.98
1.02
2.00
0.97
1.97

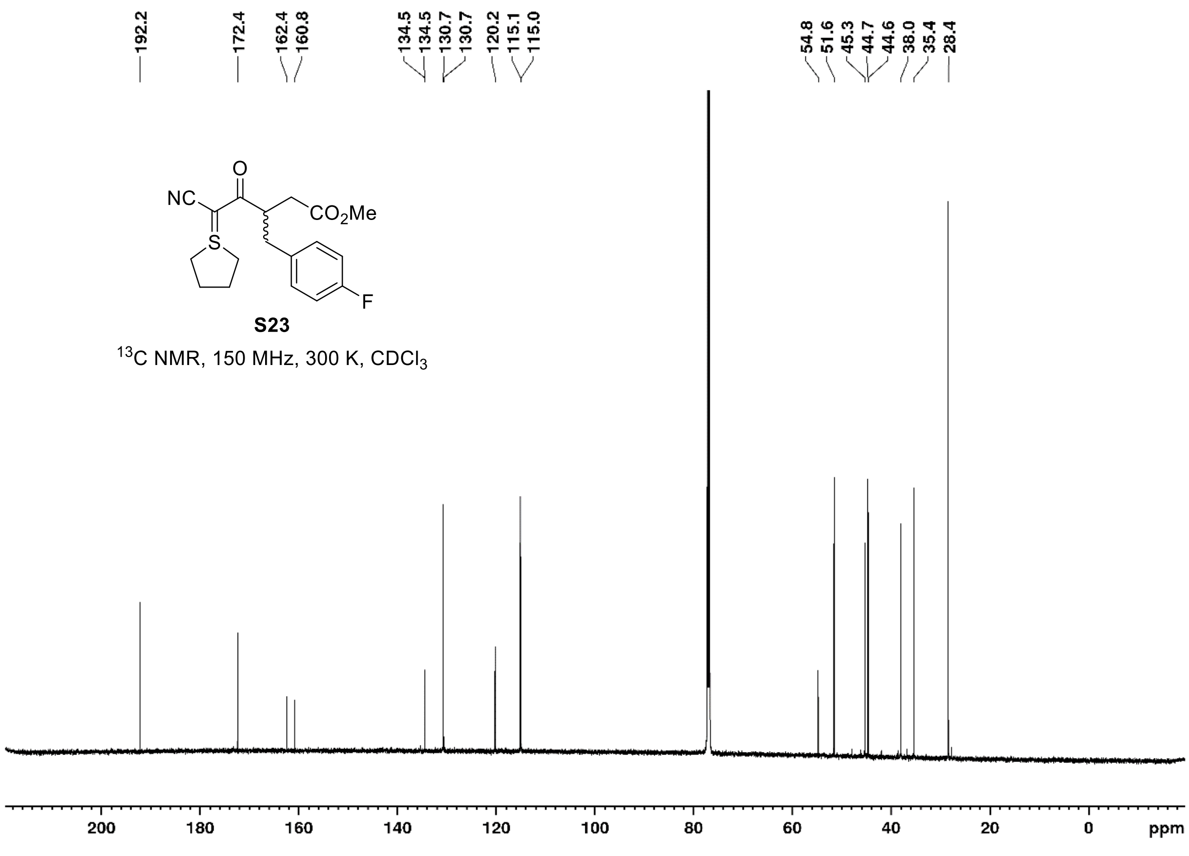

192.2
172.4
162.4
160.8
134.5
134.5
130.7
130.7
120.2
115.1
115.0
54.8
51.6
45.3
44.7
44.6
38.0
35.4
28.4
NC
O
CO2Me
S
F
S23
13C NMR, 150 MHz, 300 K, CDCl3
200 180 160 140 120 100 80 60 40 20 0 ppm

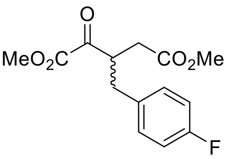

S24

^{1}H NMR, 600 MHz, 300 K, $CDCl_3$

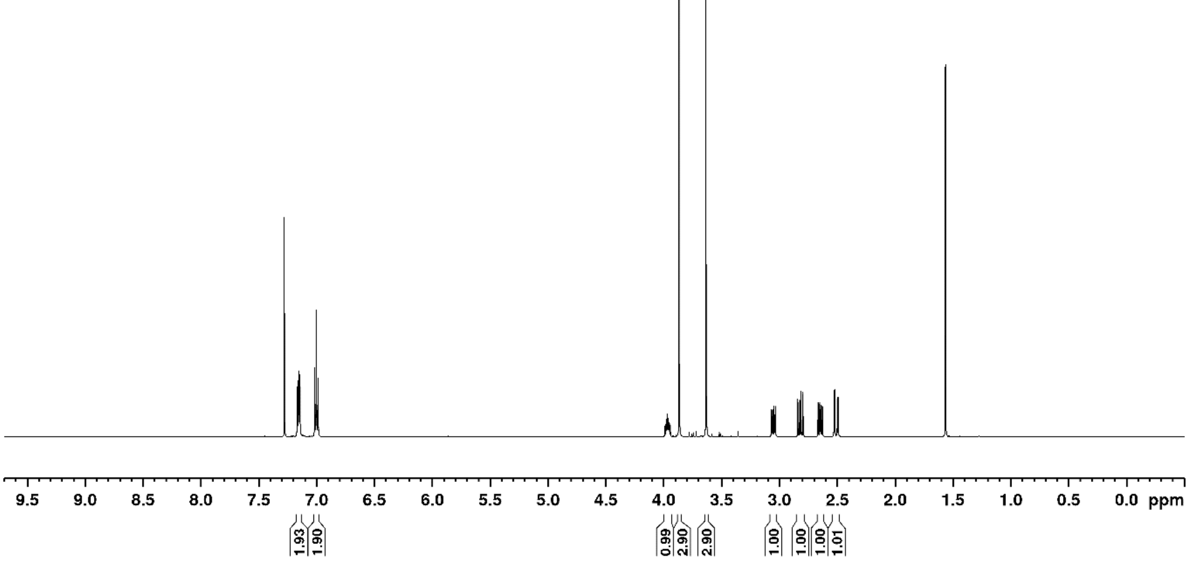

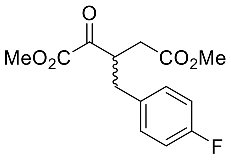

S24

^{13}C NMR, 150 MHz, 300 K, $CDCl_3$

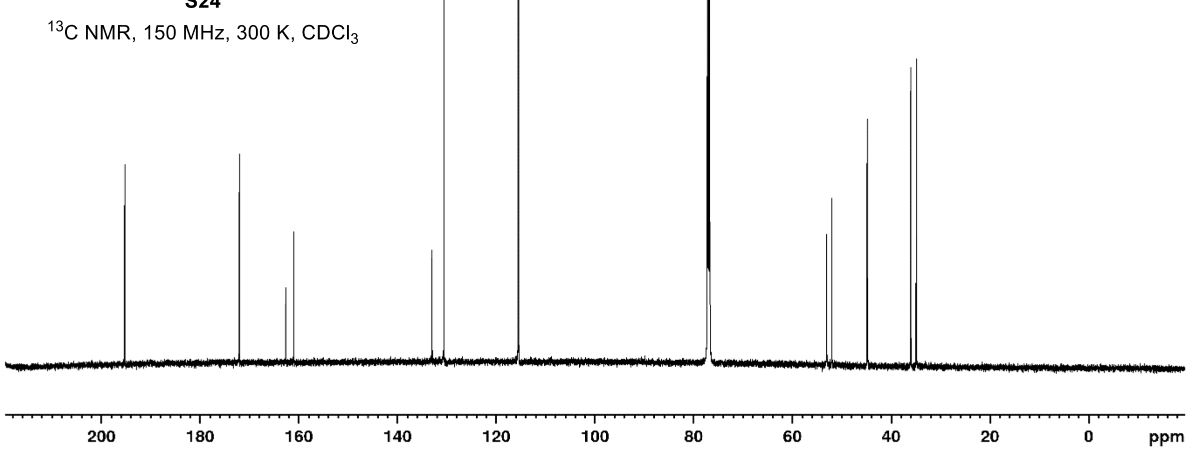

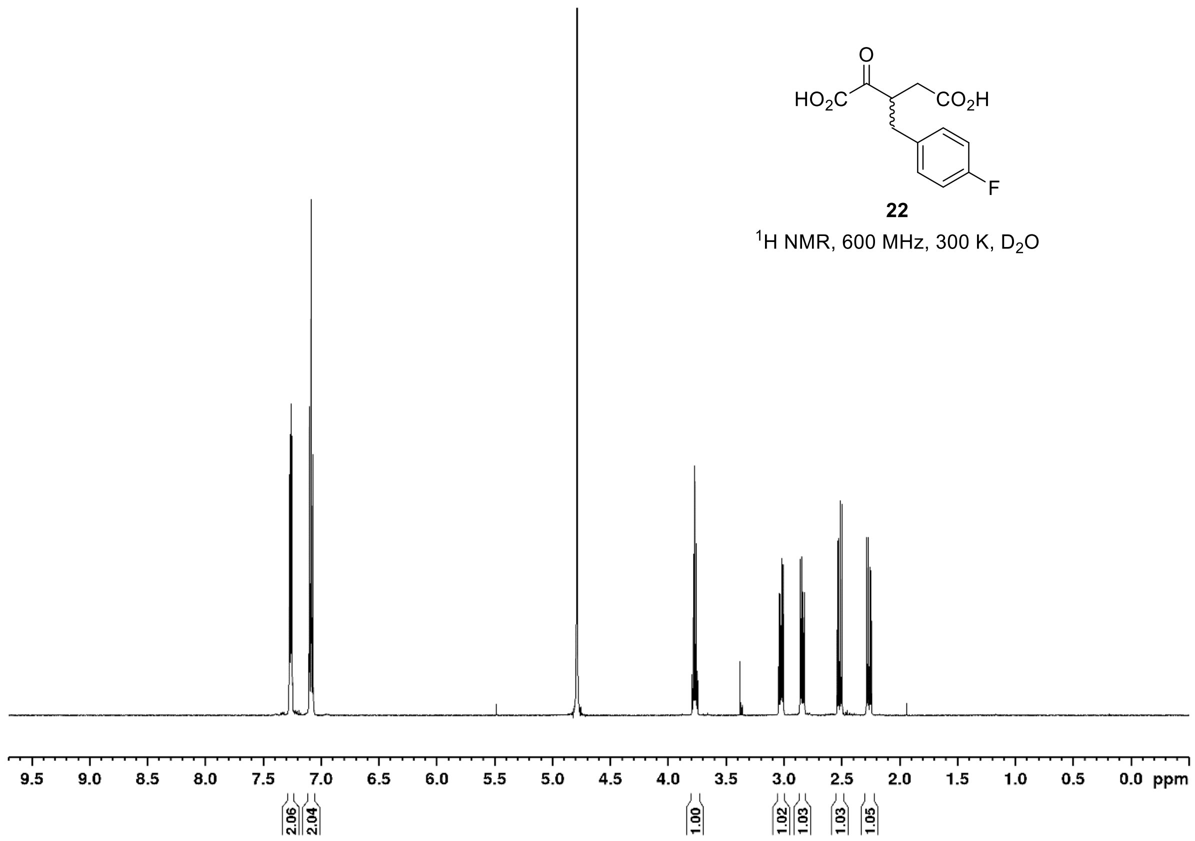
O
HO2C
CO2H
F
22
1H NMR, 600 MHz, 300 K, D2O
9.5 9.0 8.5 8.0 7.5 7.0 6.5 6.0 5.5 5.0 4.5 4.0 3.5 3.0 2.5 2.0 1.5 1.0 0.5 0.0 ppm
2.06 2.04 1.00 1.02 1.03 1.03 1.05

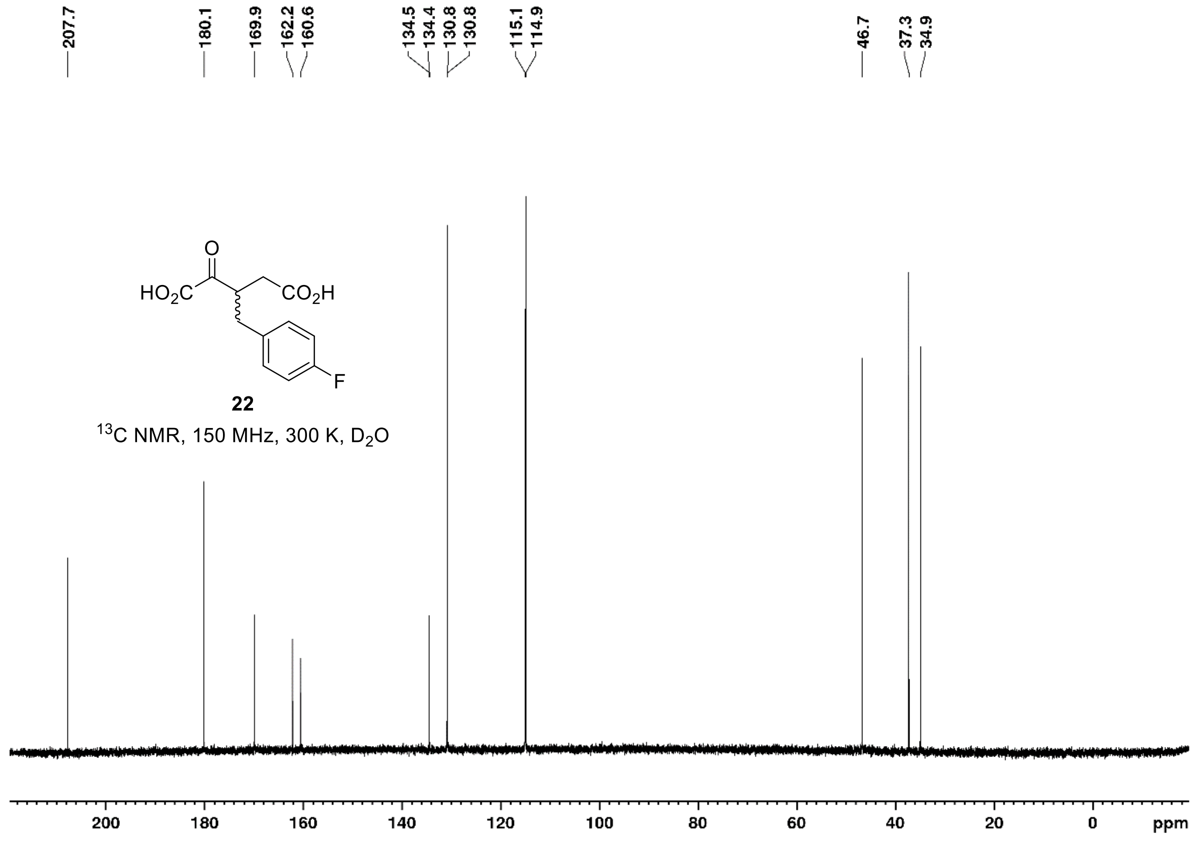
207.7 180.1 169.9 162.2 160.6 134.5 134.4 130.8 130.8 115.1 114.9 46.7 37.3 34.9
O
HO2C
CO2H
F
22
13C NMR, 150 MHz, 300 K, D2O
200 180 160 140 120 100 80 60 40 20 0 ppm

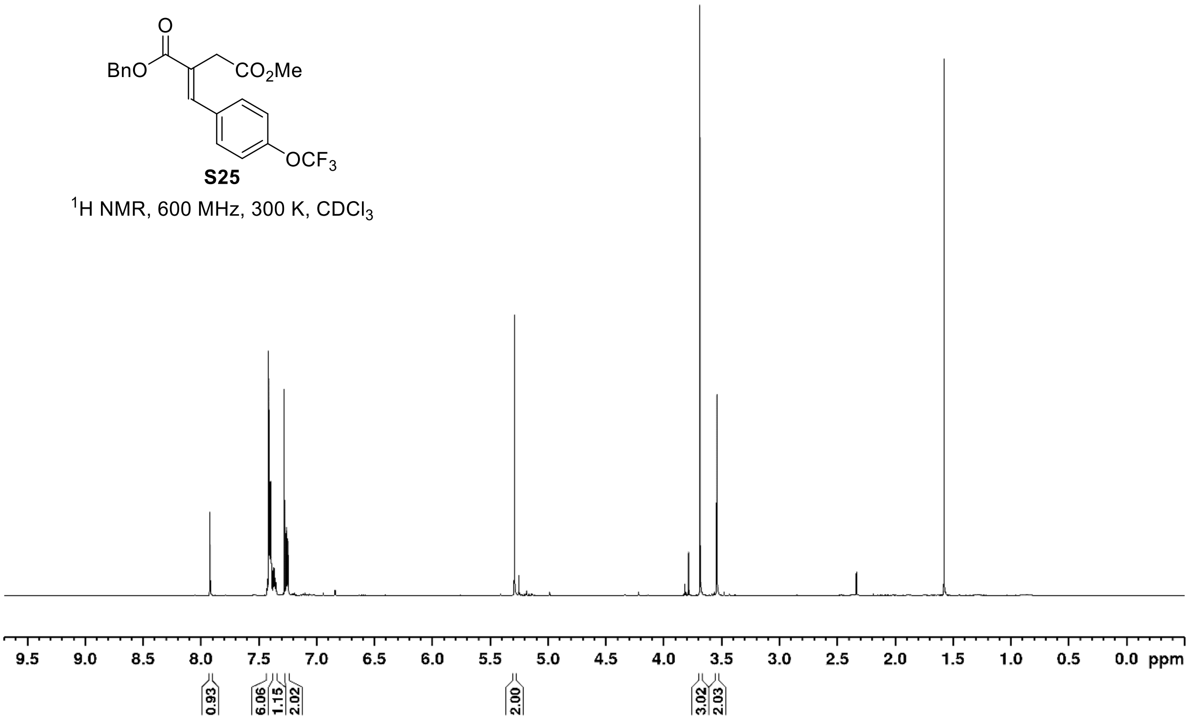
BnO
O
CO2Me
OCF3
S25
1H NMR, 600 MHz, 300 K, CDCl3
9.5 9.0 8.5 8.0 7.5 7.0 6.5 6.0 5.5 5.0 4.5 4.0 3.5 3.0 2.5 2.0 1.5 1.0 0.5 0.0 ppm
0.93
6.06
1.15
2.02
2.00
3.02
2.03

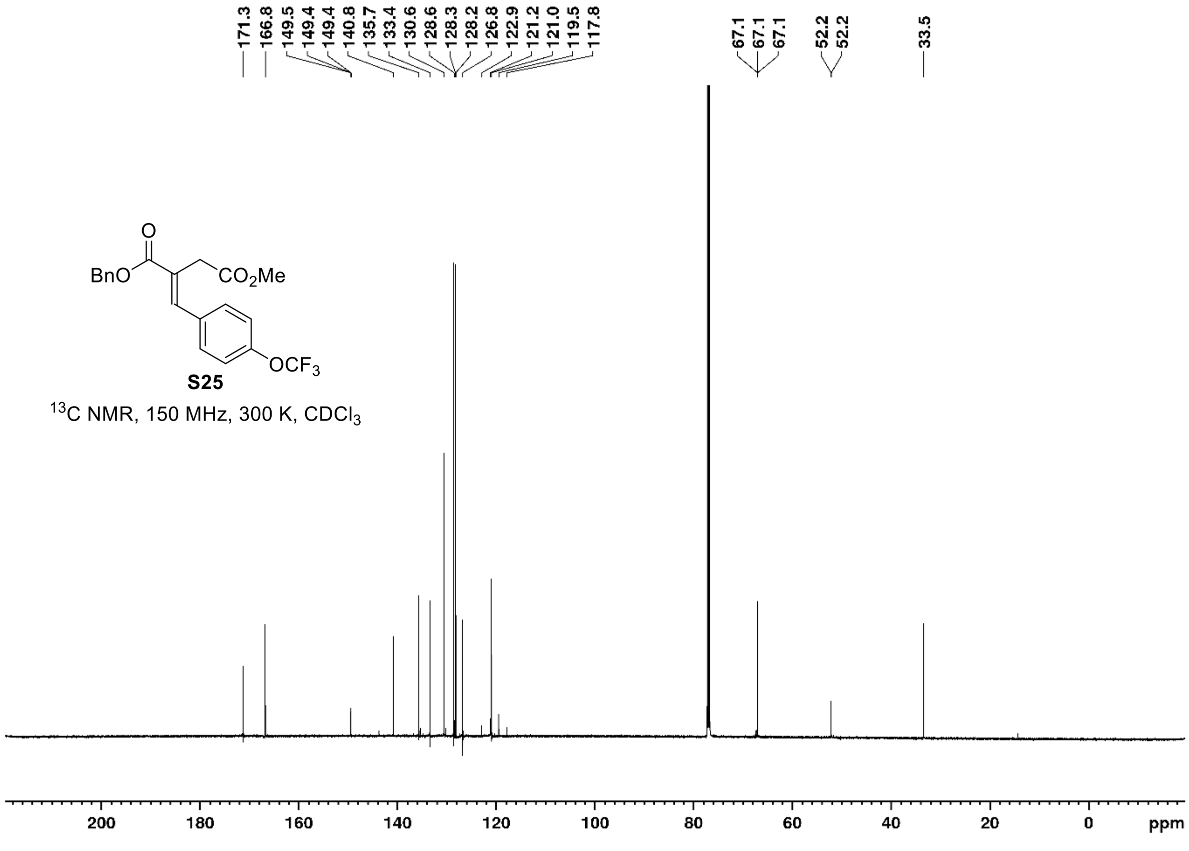
171.3
166.8
149.5
149.4
149.4
140.8
135.7
133.4
130.6
128.6
128.3
128.2
126.8
122.9
121.2
121.0
119.5
117.8
67.1
67.1
67.1
52.2
52.2
33.5
BnO
O
CO2Me
OCF3
S25
13C NMR, 150 MHz, 300 K, CDCl3
200 180 160 140 120 100 80 60 40 20 0 ppm

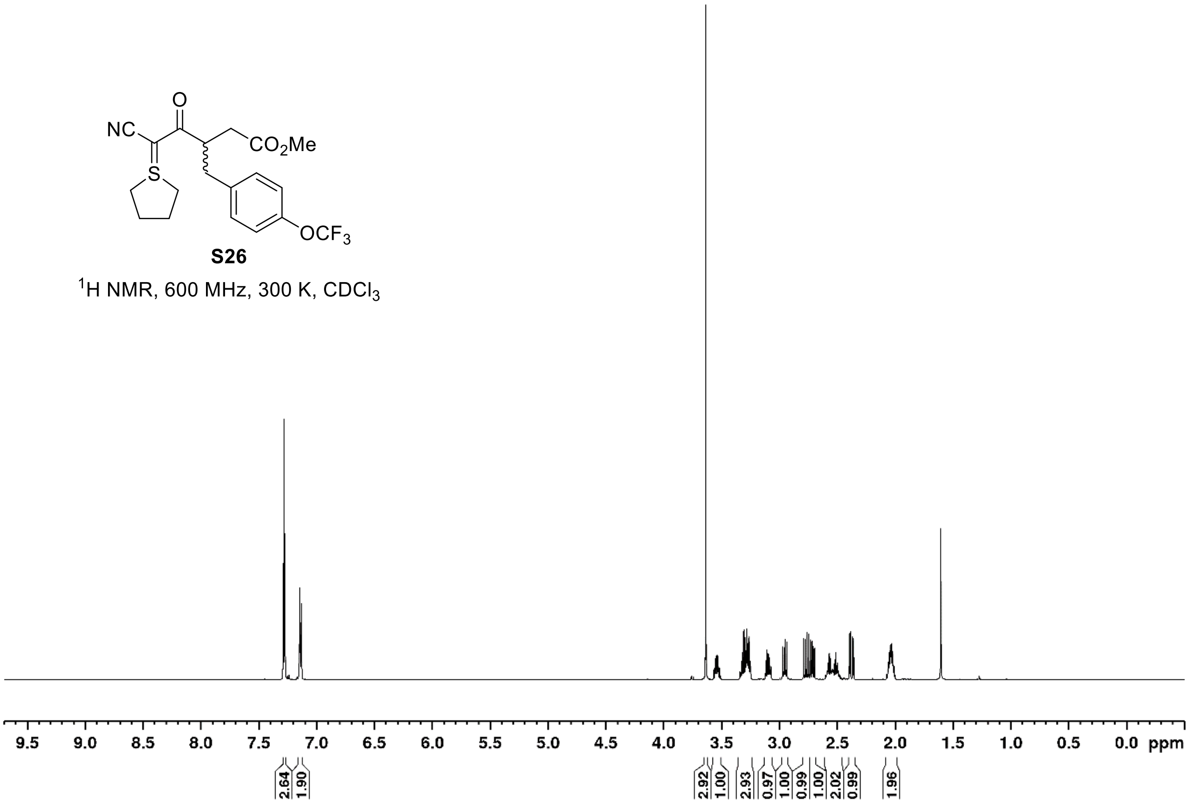
NC
O
CO2Me
S
OCF3
S26
1H NMR, 600 MHz, 300 K, CDCl3
9.5 9.0 8.5 8.0 7.5 7.0 6.5 6.0 5.5 5.0 4.5 4.0 3.5 3.0 2.5 2.0 1.5 1.0 0.5 0.0 ppm
2.64 1.90 2.92 1.00 2.93 0.97 1.00 0.99 1.00 2.02 0.99 1.96

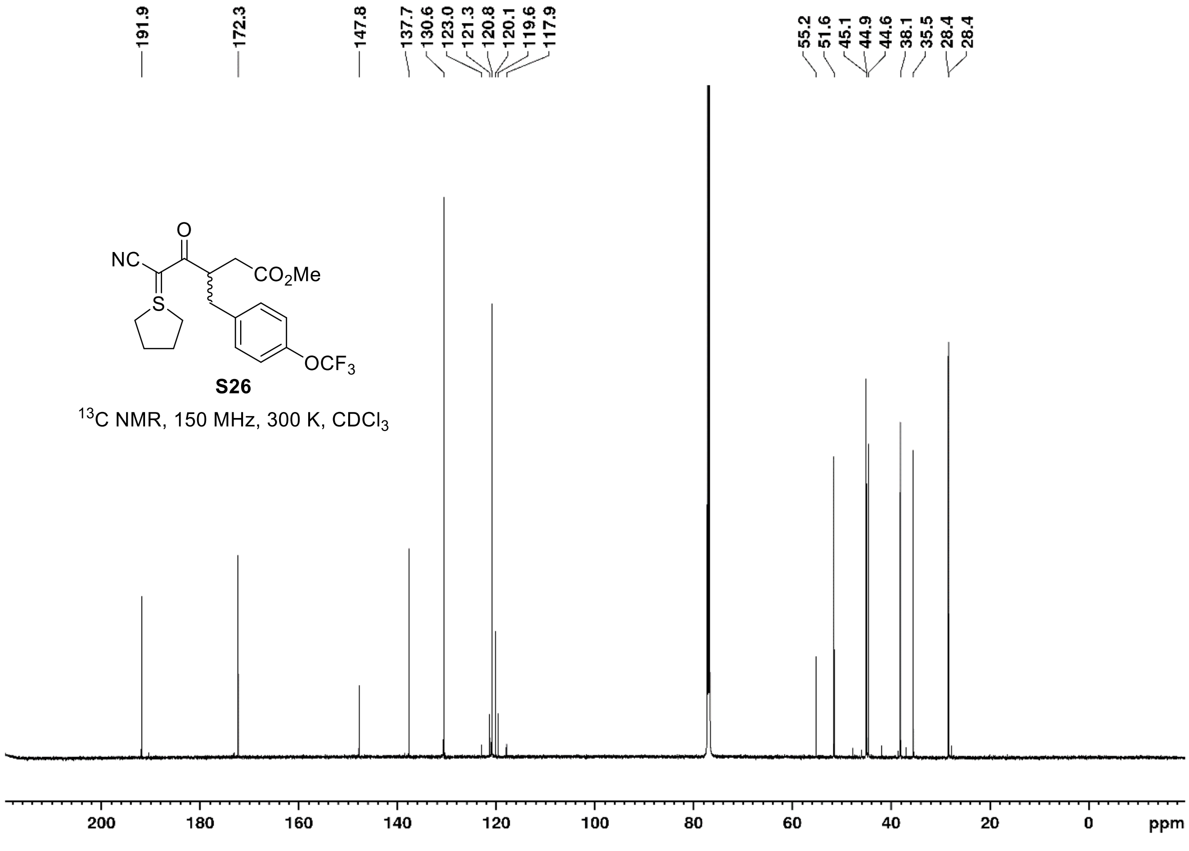
191.9
172.3
147.8
137.7 130.6 123.0 121.3 120.8 120.1 119.6 117.9
55.2 51.6 45.1 44.9 44.6 38.1 35.5 28.4 28.4
NC
O
CO2Me
S
OCF3
S26
13C NMR, 150 MHz, 300 K, CDCl3
200 180 160 140 120 100 80 60 40 20 0 ppm

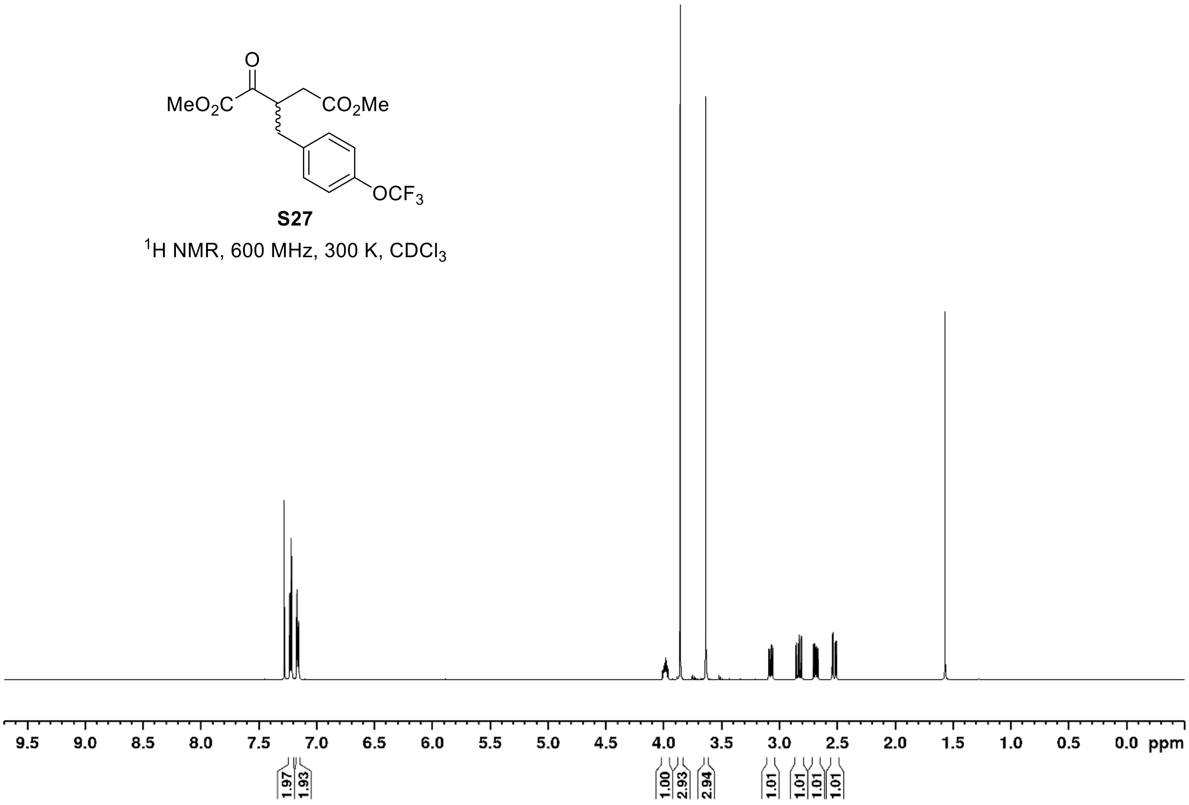
MeO2C
CO2Me
OCF3
S27
1H NMR, 600 MHz, 300 K, CDCl3
9.5 9.0 8.5 8.0 7.5 7.0 6.5 6.0 5.5 5.0 4.5 4.0 3.5 3.0 2.5 2.0 1.5 1.0 0.5 0.0 ppm
1.97 1.93 1.00 2.93 2.94 1.01 1.01 1.01 1.01

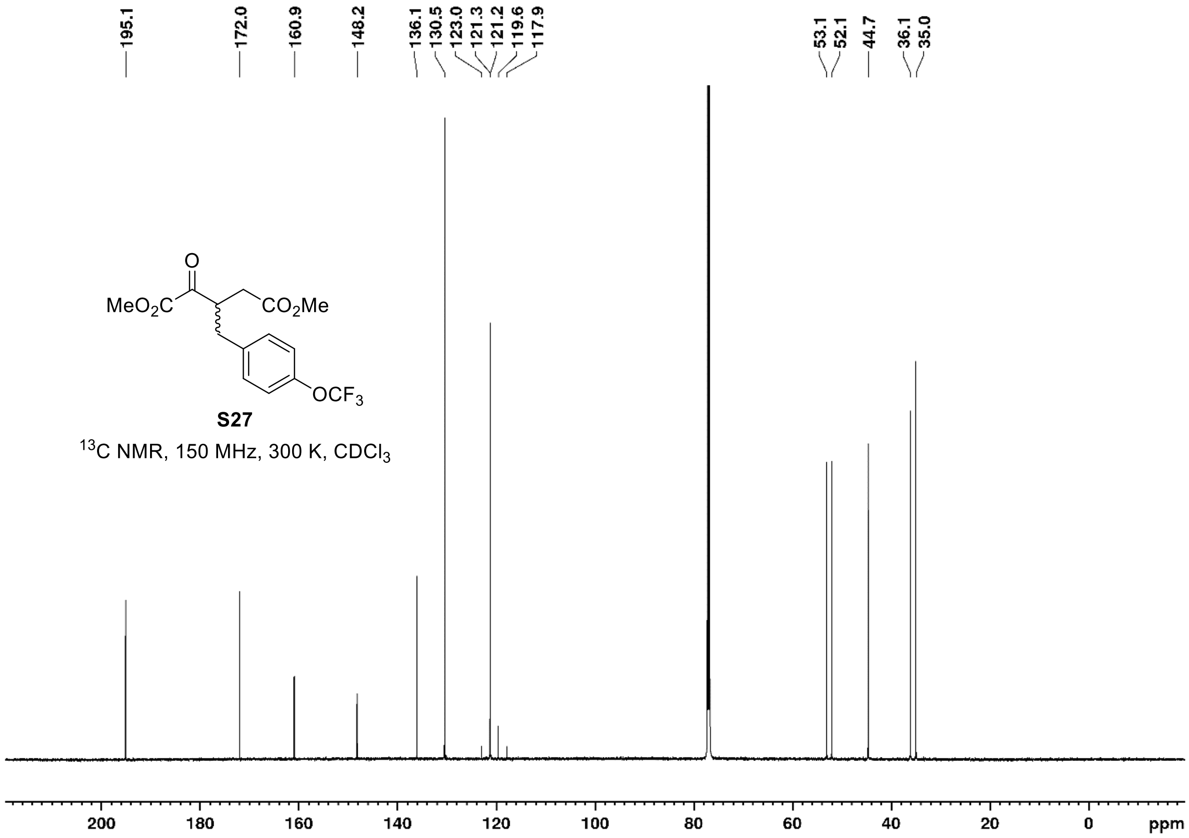
195.1 172.0 160.9 148.2 136.1 130.5 123.0 121.3 121.2 119.6 117.9 53.1 52.1 44.7 36.1 35.0
MeO2C
CO2Me
OCF3
S27
13C NMR, 150 MHz, 300 K, CDCl3
200 180 160 140 120 100 80 60 40 20 0 ppm

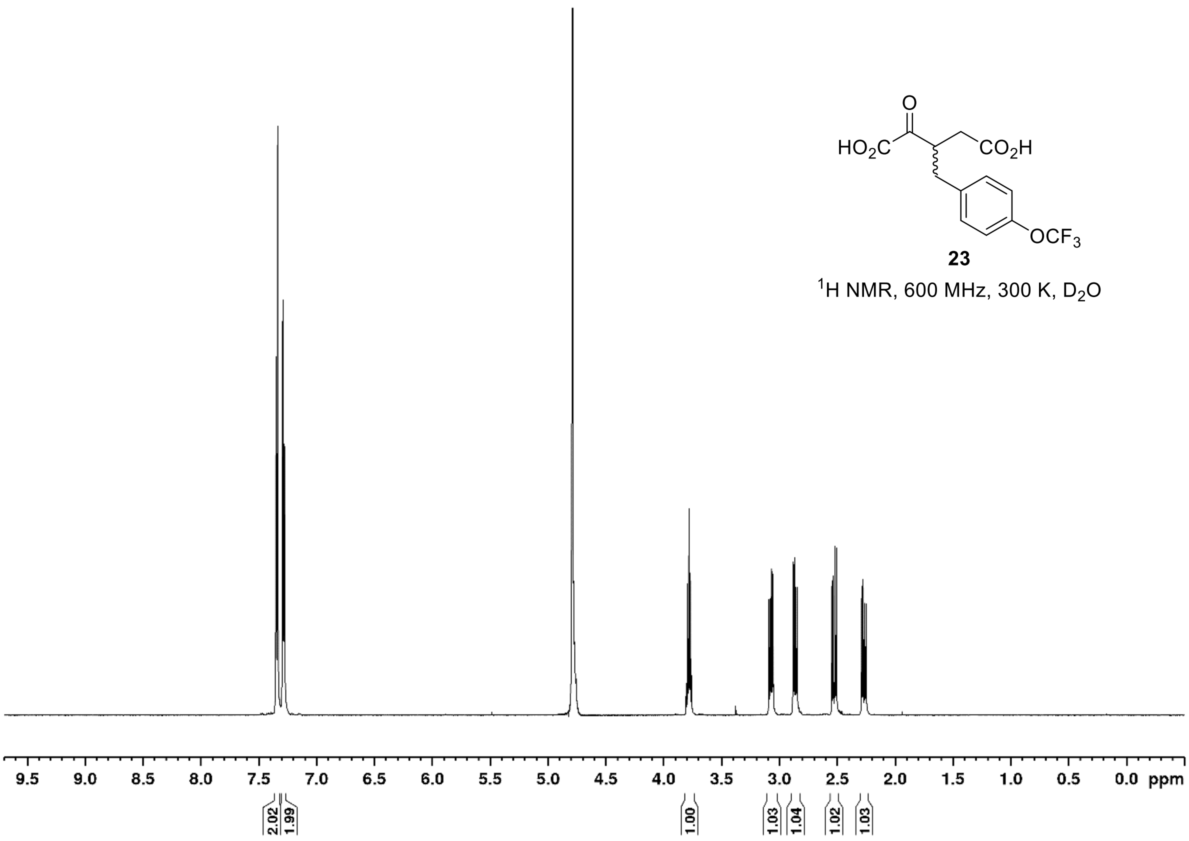
23
^{1}H NMR, 600 MHz, 300 K, D_2O
9.5 9.0 8.5 8.0 7.5 7.0 6.5 6.0 5.5 5.0 4.5 4.0 3.5 3.0 2.5 2.0 1.5 1.0 0.5 0.0 ppm
2.02 1.99 1.00 1.03 1.04 1.02 1.03

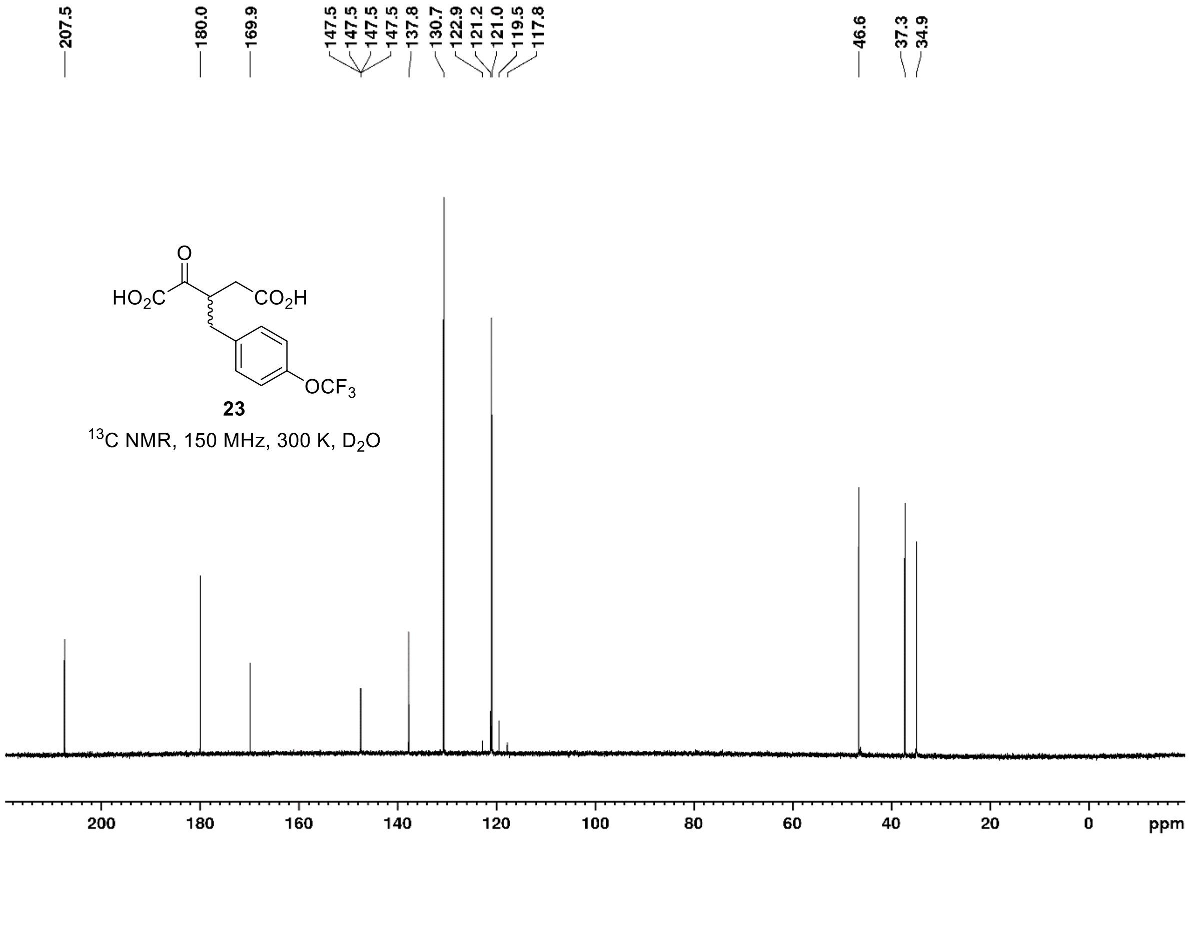
207.5
180.0
169.9
147.5 147.5 147.5 147.5 137.8 130.7 122.9 121.2 121.0 119.5 117.8
46.6
37.3 34.9
23
^{13}C NMR, 150 MHz, 300 K, D_2O
200 180 160 140 120 100 80 60 40 20 0 ppm

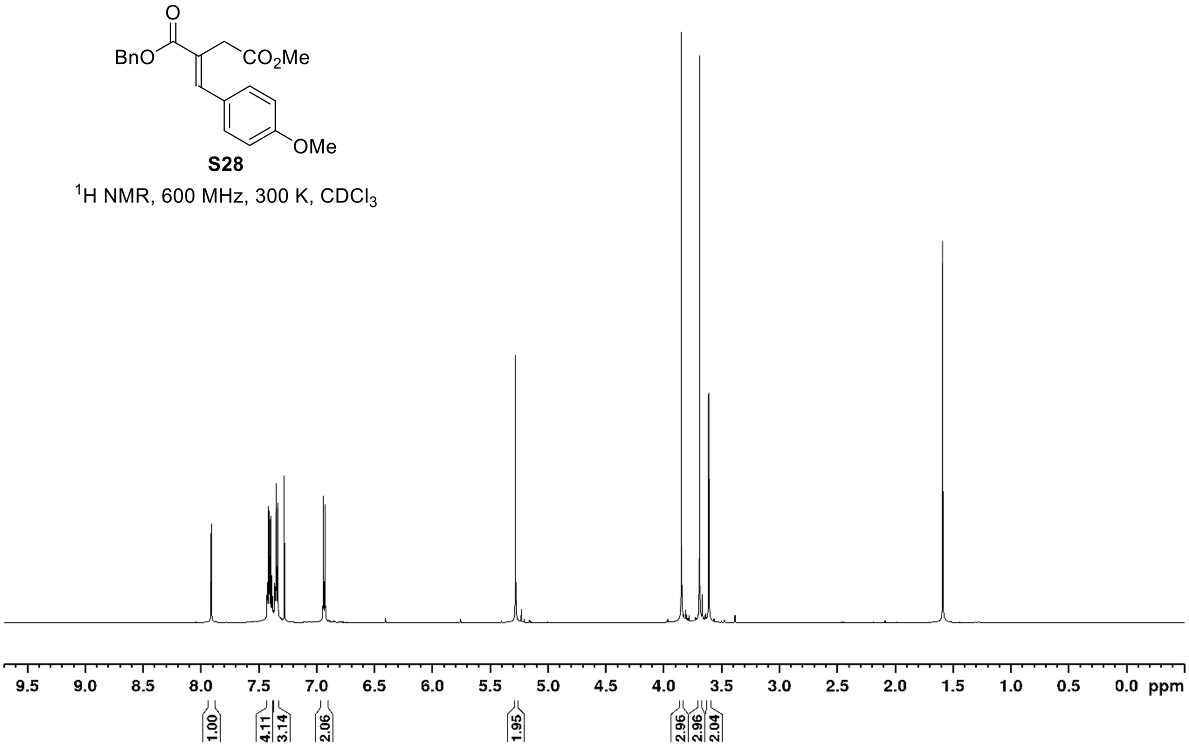

S28

^{1}H NMR, 600 MHz, 300 K, $CDCl_3$

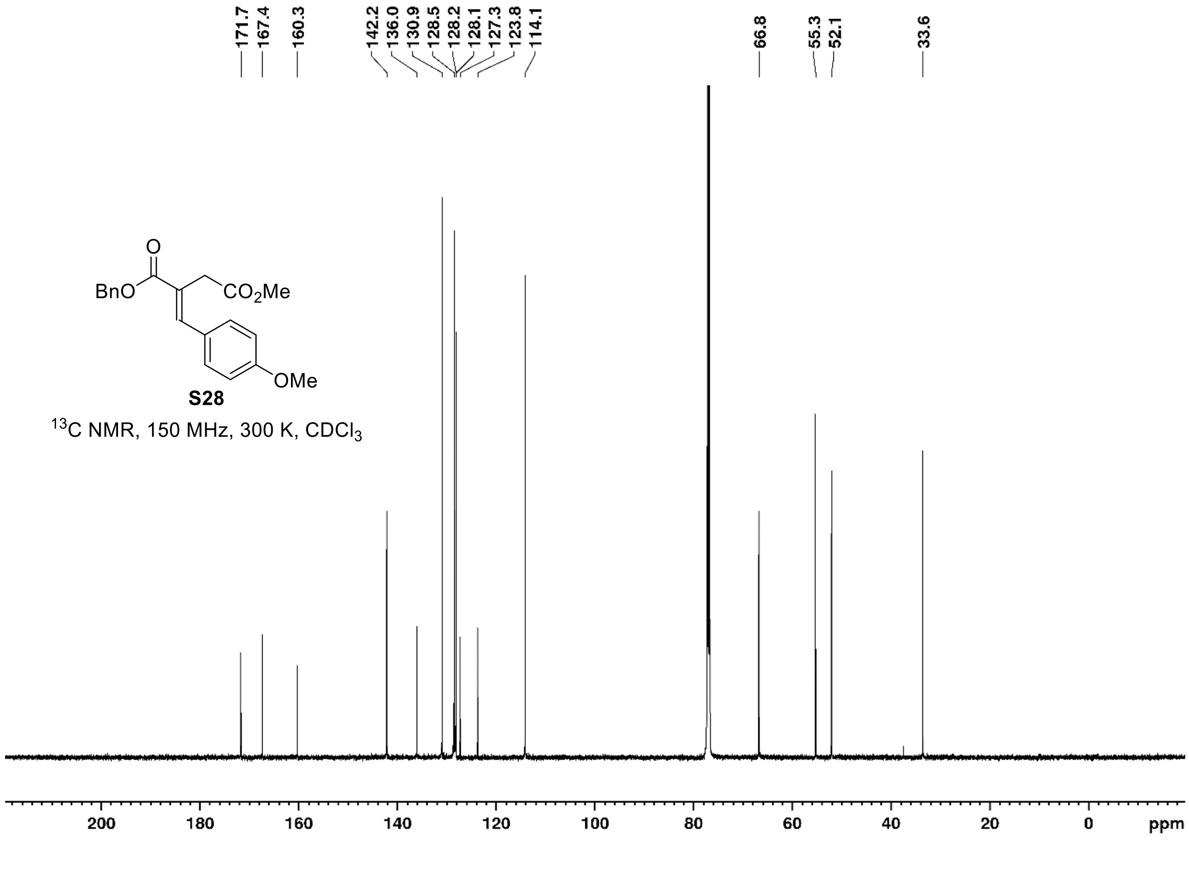

S28

^{13}C NMR, 150 MHz, 300 K, $CDCl_3$

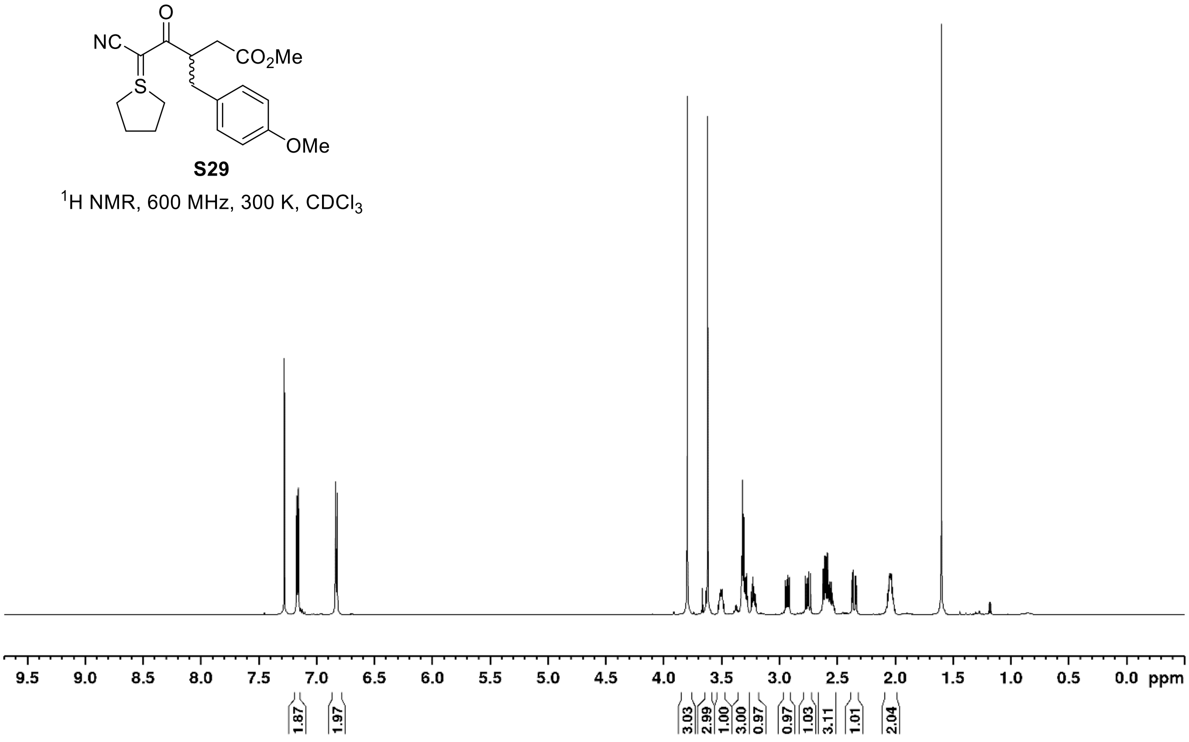
NC
O
CO_2Me
S
OMe
S29
^{1}H NMR, 600 MHz, 300 K, $CDCl_3$
9.5 9.0 8.5 8.0 7.5 7.0 6.5 6.0 5.5 5.0 4.5 4.0 3.5 3.0 2.5 2.0 1.5 1.0 0.5 0.0 ppm
1.87 1.97
3.03 2.99 1.00 3.00 0.97 0.97 1.03 3.11 1.01 2.04

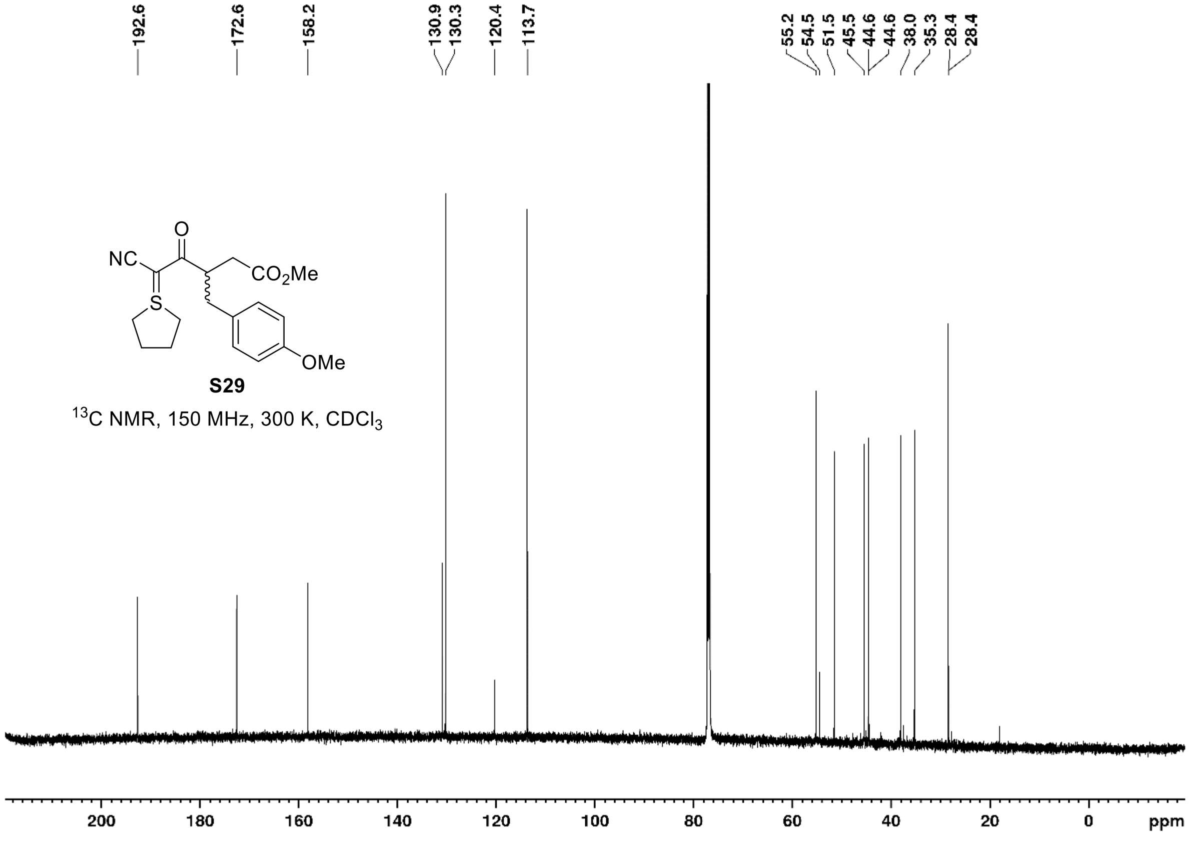
192.6
172.6
158.2
130.9
130.3
120.4
113.7
55.2
54.5
51.5
45.5
44.6
44.6
38.0
35.3
28.4
28.4
NC
O
CO_2Me
S
OMe
S29
^{13}C NMR, 150 MHz, 300 K, $CDCl_3$
200 180 160 140 120 100 80 60 40 20 0 ppm

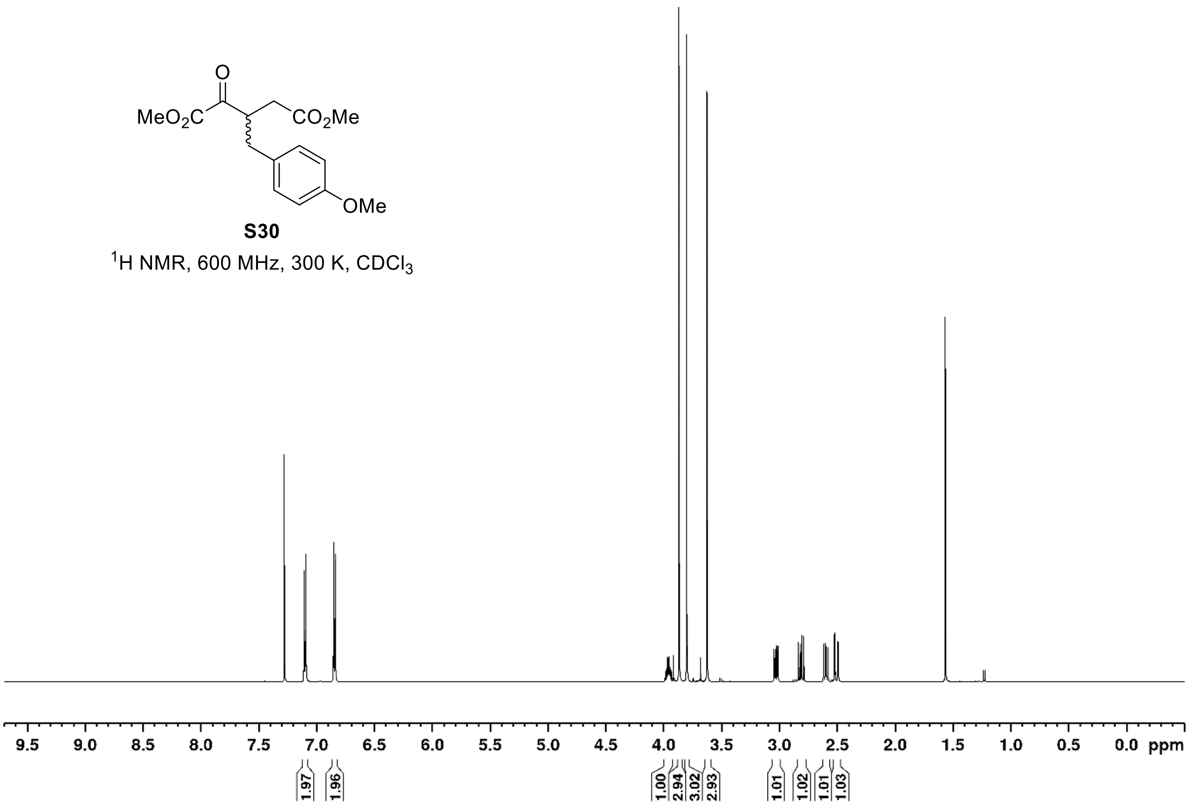
MeO2C
CO2Me
OMe
S30
1H NMR, 600 MHz, 300 K, CDCl3
9.5 9.0 8.5 8.0 7.5 7.0 6.5 6.0 5.5 5.0 4.5 4.0 3.5 3.0 2.5 2.0 1.5 1.0 0.5 0.0 ppm
1.97
1.96
1.00
2.94
3.02
2.93
1.01
1.02
1.01
1.03

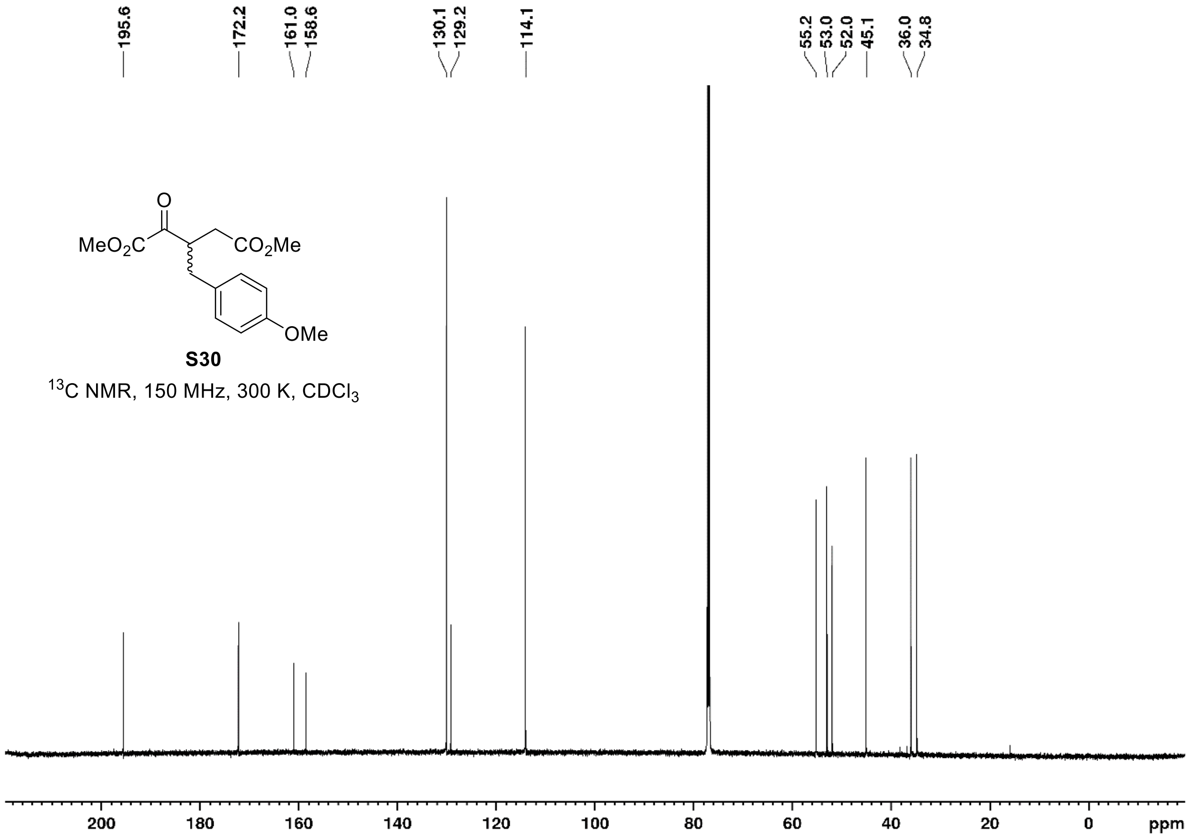
195.6
172.2
161.0
158.6
130.1
129.2
114.1
55.2
53.0
52.0
45.1
36.0
34.8
MeO2C
CO2Me
OMe
S30
13C NMR, 150 MHz, 300 K, CDCl3
200 180 160 140 120 100 80 60 40 20 0 ppm

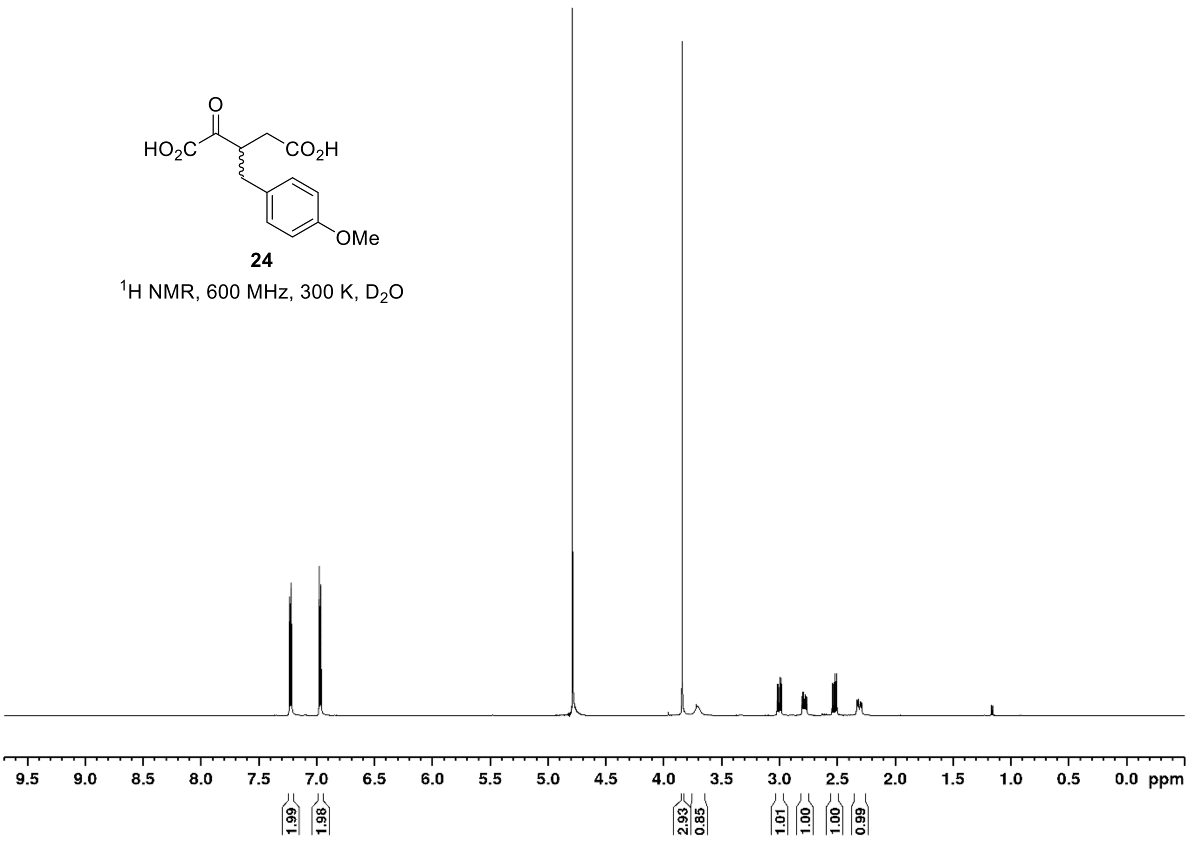
HO2C
CO2H
OMe
24
1H NMR, 600 MHz, 300 K, D2O
9.5 9.0 8.5 8.0 7.5 7.0 6.5 6.0 5.5 5.0 4.5 4.0 3.5 3.0 2.5 2.0 1.5 1.0 0.5 0.0 ppm
1.99 1.98 2.93 0.85 1.01 1.00 1.00 0.99

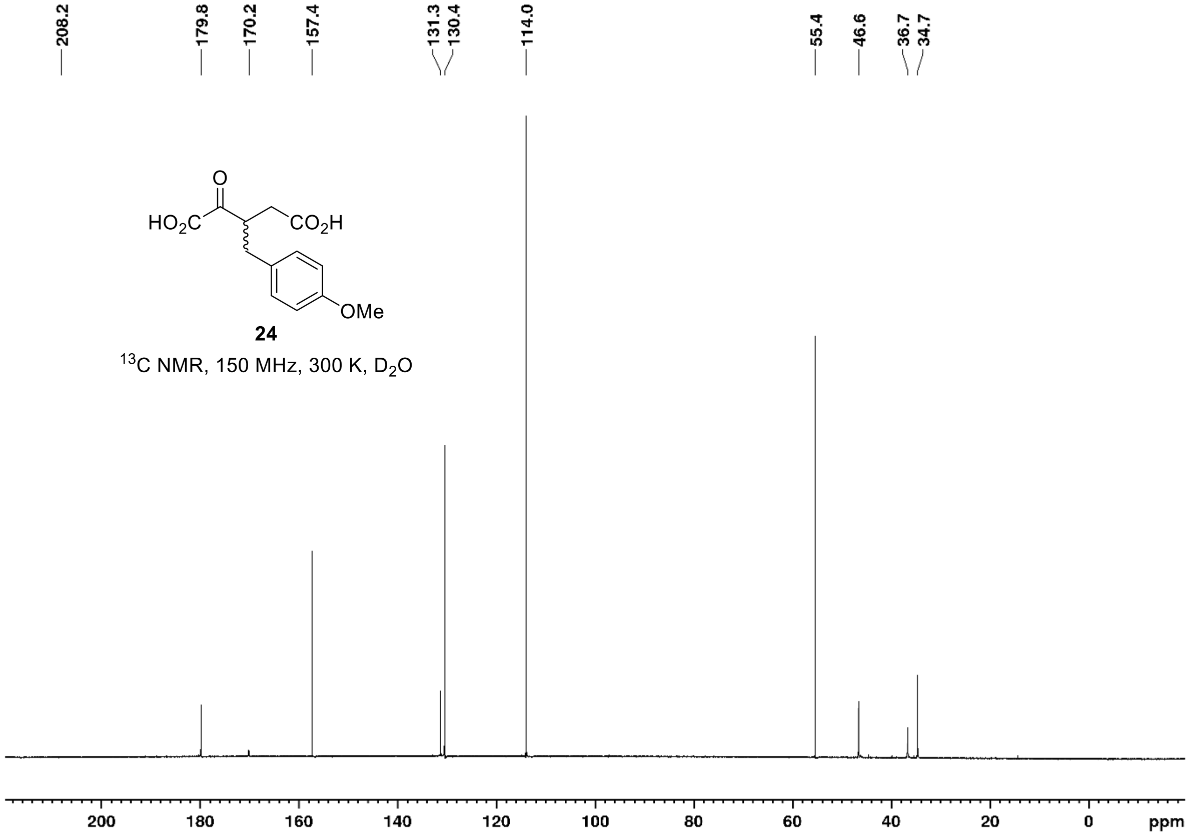
208.2
179.8
170.2
157.4
131.3
130.4
114.0
55.4
46.6
36.7
34.7
HO2C
CO2H
OMe
24
13C NMR, 150 MHz, 300 K, D2O
200 180 160 140 120 100 80 60 40 20 0 ppm

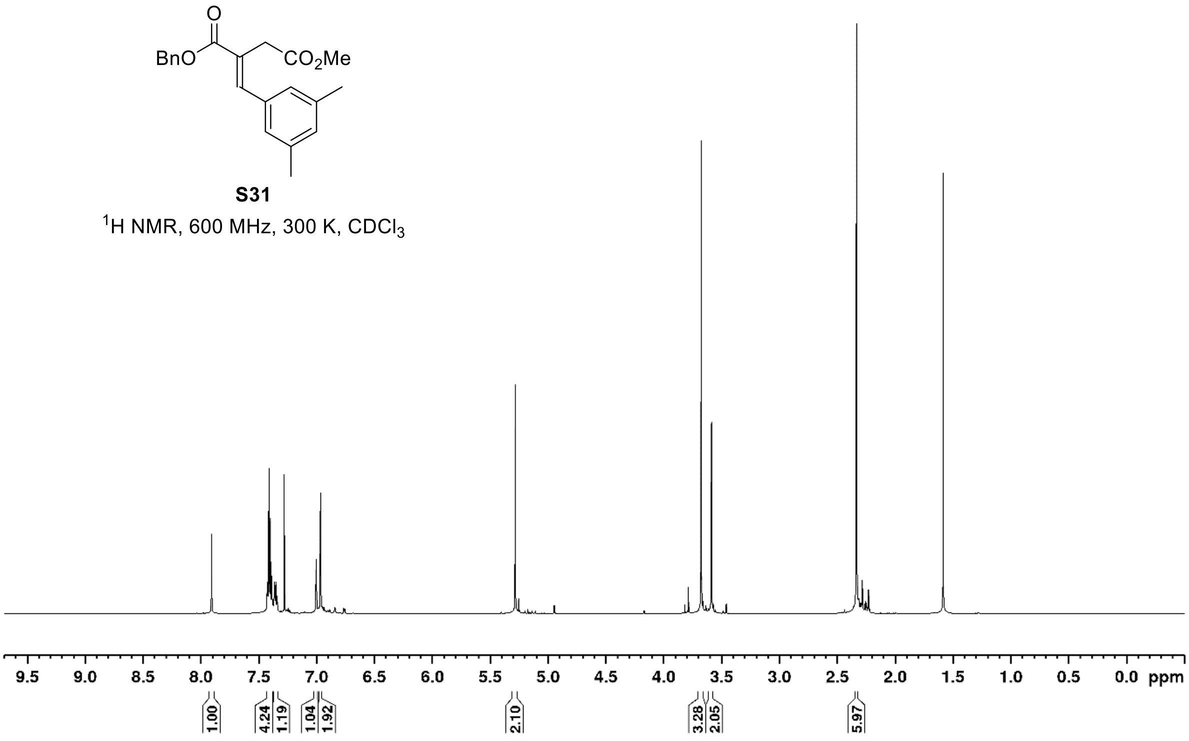
O
BnO
CO2Me
S31
1H NMR, 600 MHz, 300 K, CDCl3
9.5 9.0 8.5 8.0 7.5 7.0 6.5 6.0 5.5 5.0 4.5 4.0 3.5 3.0 2.5 2.0 1.5 1.0 0.5 0.0 ppm
1.00
4.24
1.19
1.04
1.92
2.10
3.28
2.05
5.97

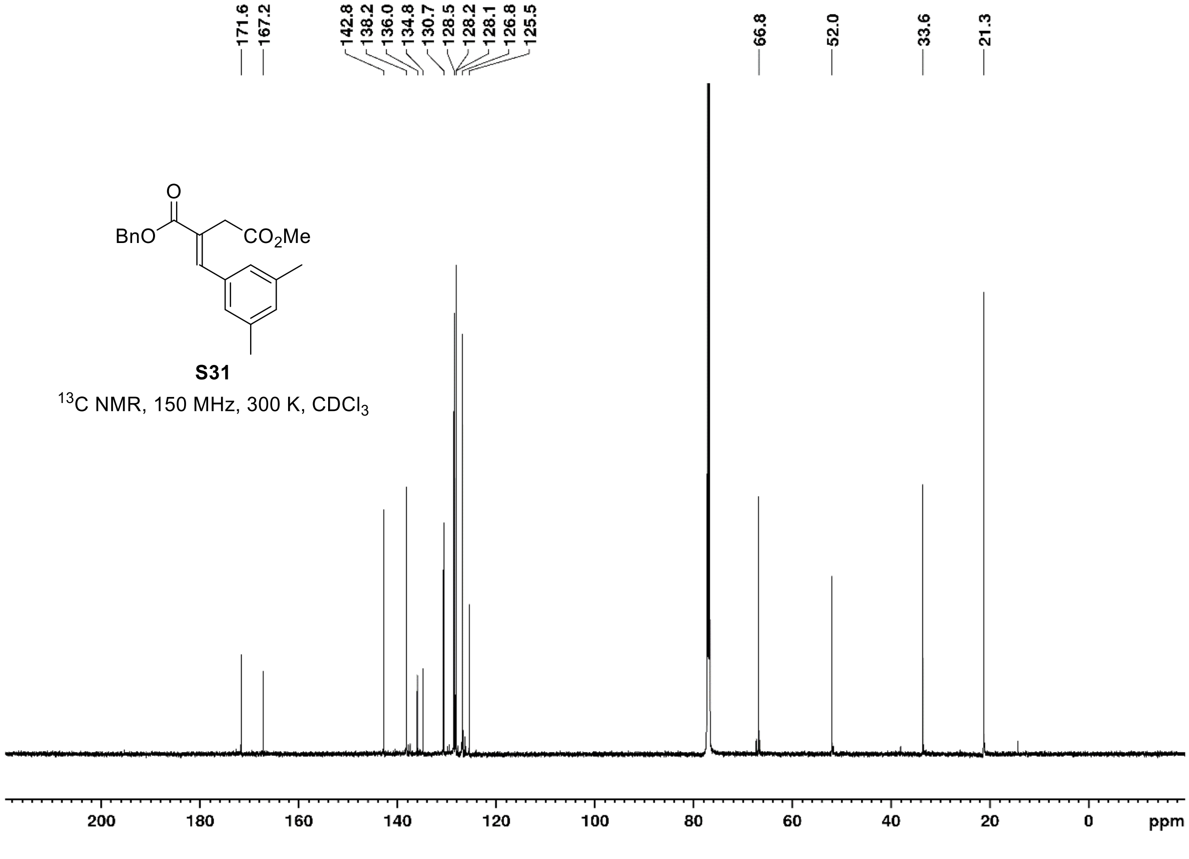
171.6
167.2
142.8
138.2
136.0
134.8
130.7
128.5
128.2
128.1
126.8
125.5
66.8
52.0
33.6
21.3
O
BnO
CO2Me
S31
13C NMR, 150 MHz, 300 K, CDCl3
200 180 160 140 120 100 80 60 40 20 0 ppm

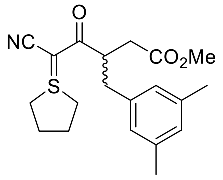

S32

^{1}H NMR, 600 MHz, 300 K, $CDCl_3$

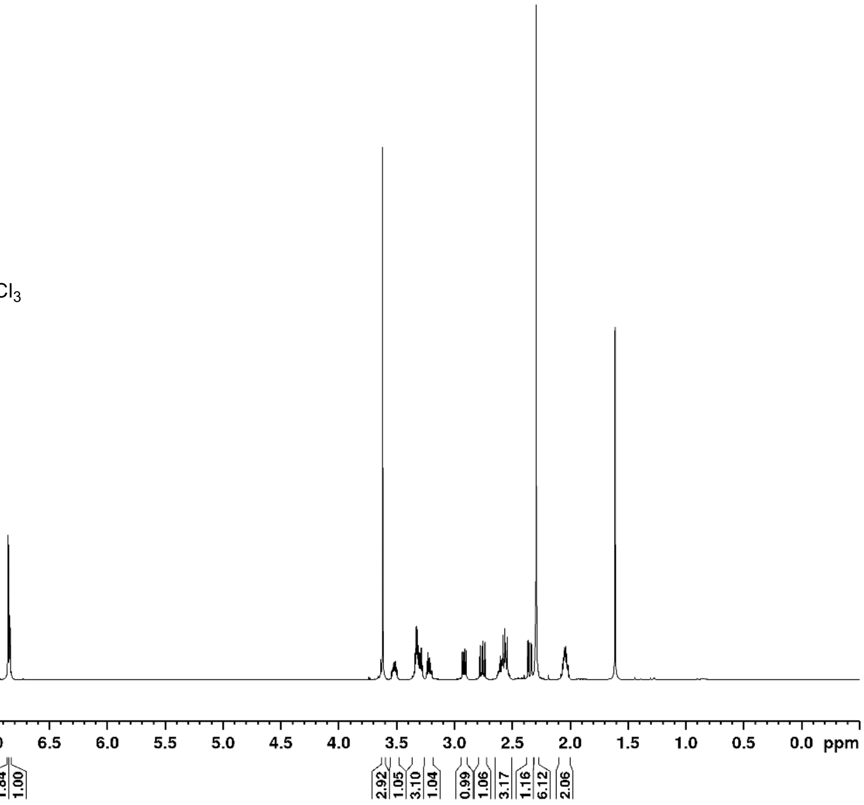

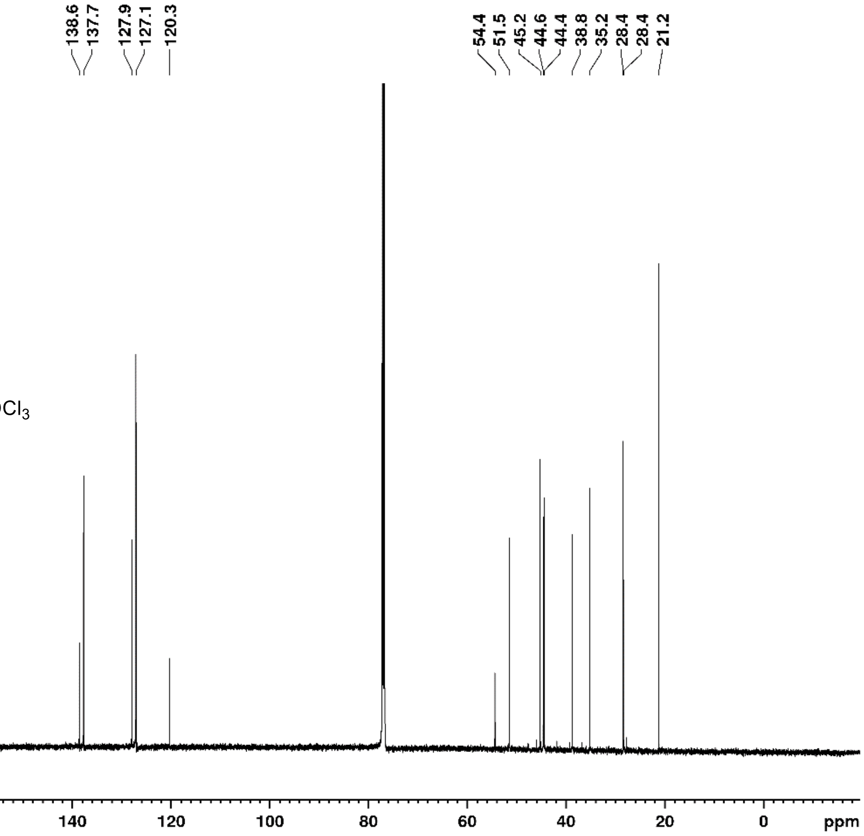

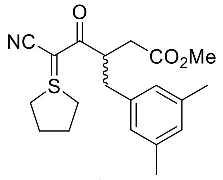

S32

^{13}C NMR, 150 MHz, 300 K, $CDCl_3$

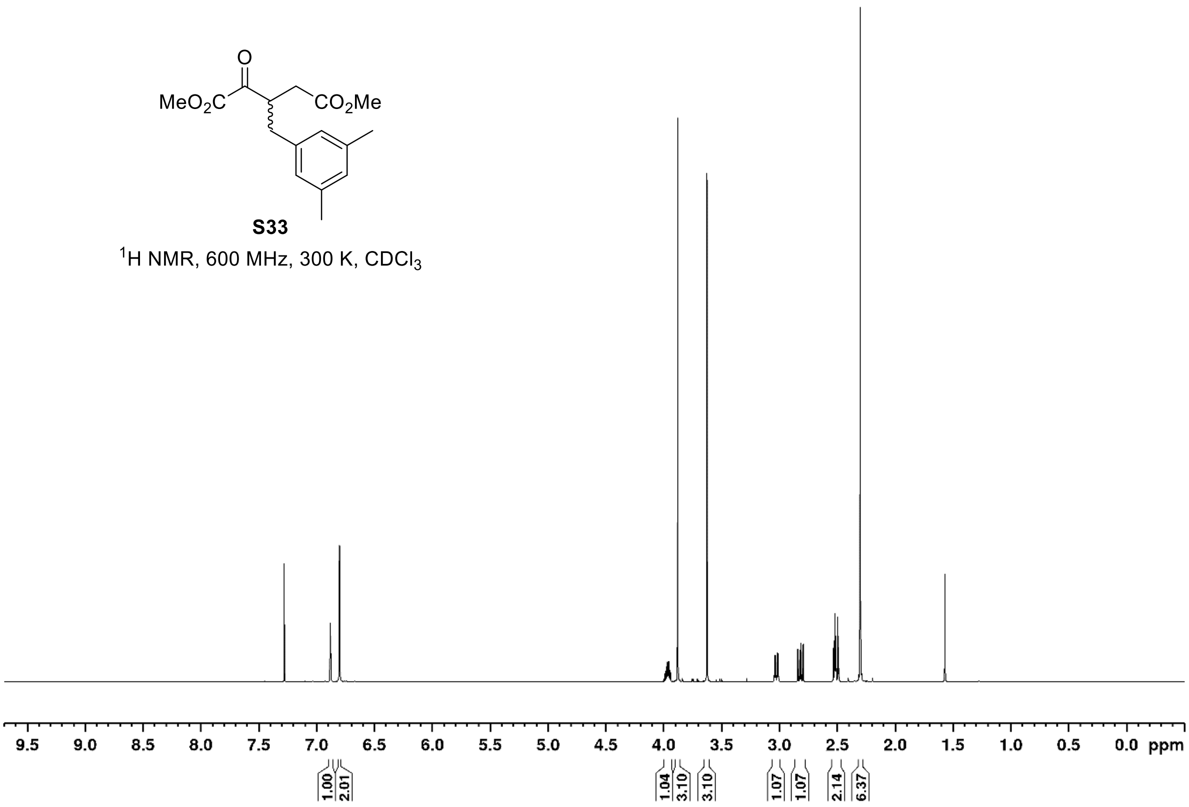

S33

^{1}H NMR, 600 MHz, 300 K, $CDCl_3$

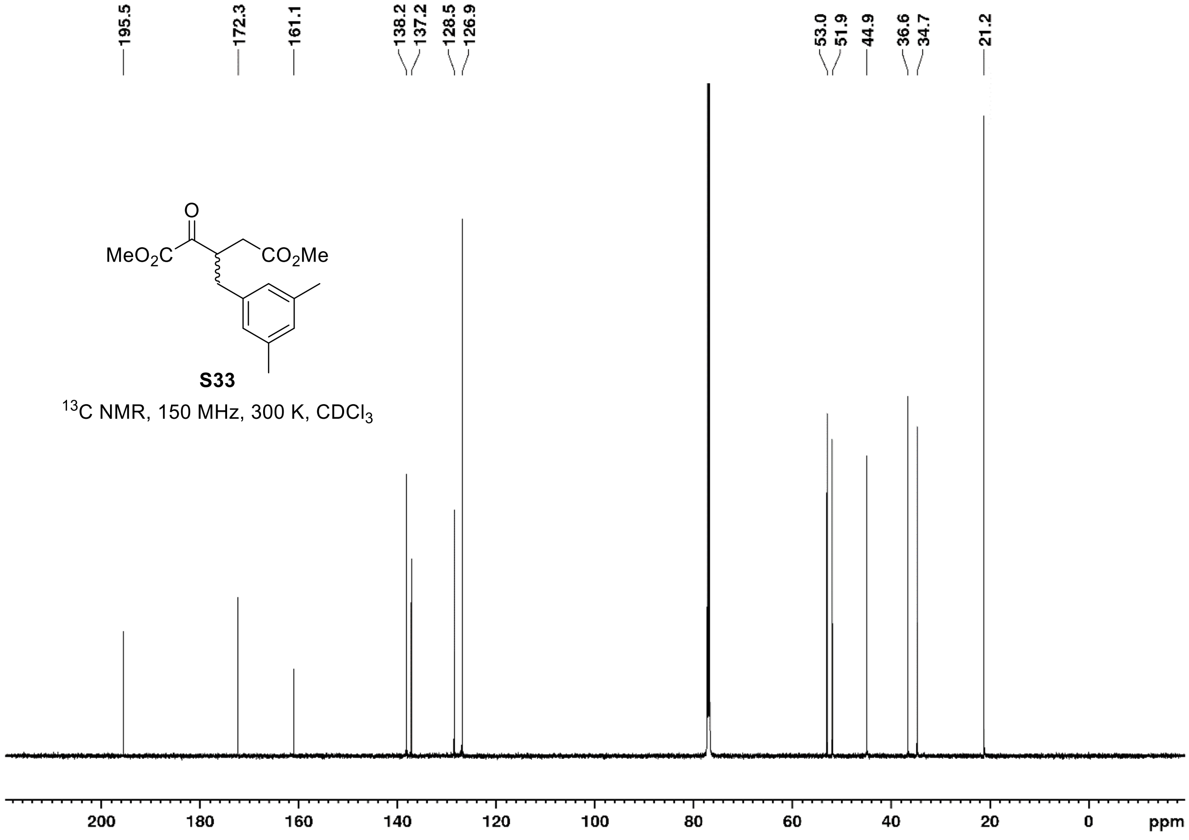

S33

^{13}C NMR, 150 MHz, 300 K, $CDCl_3$

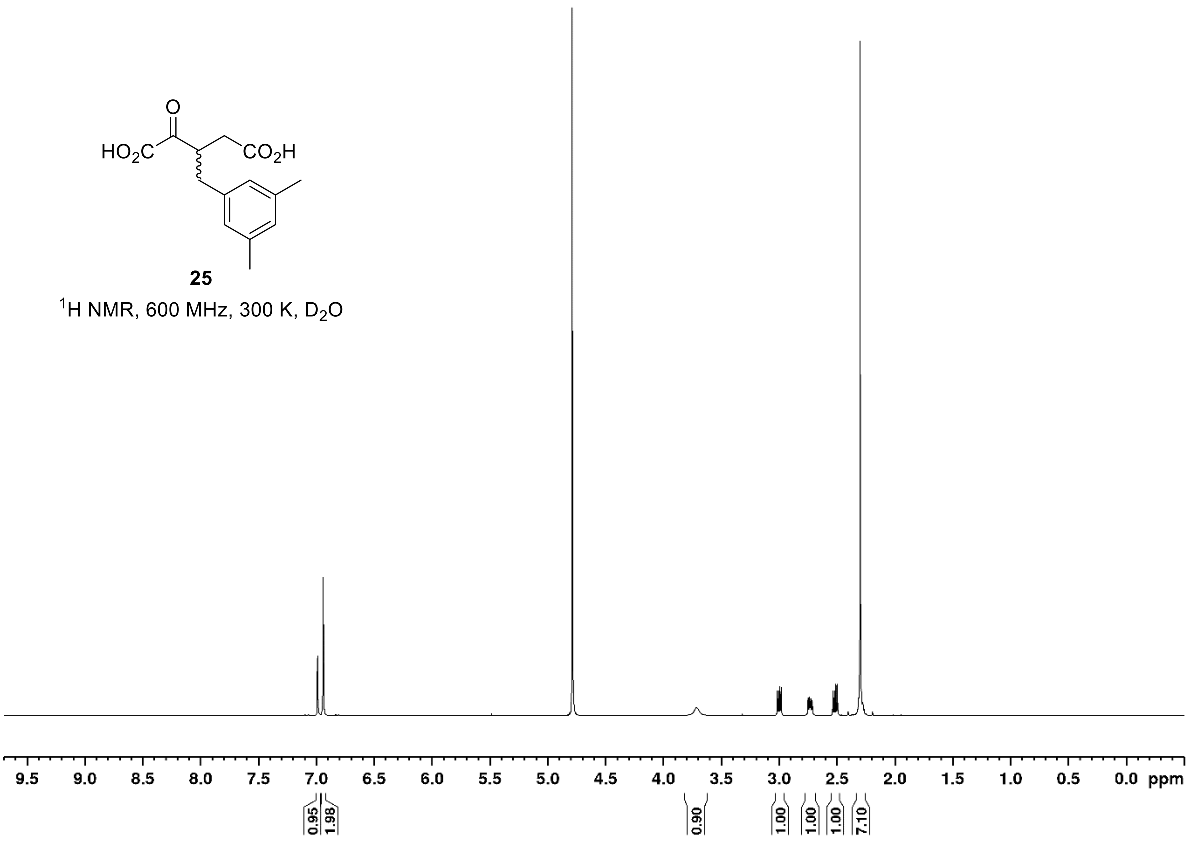

25

^{1}H NMR, 600 MHz, 300 K, D_2O

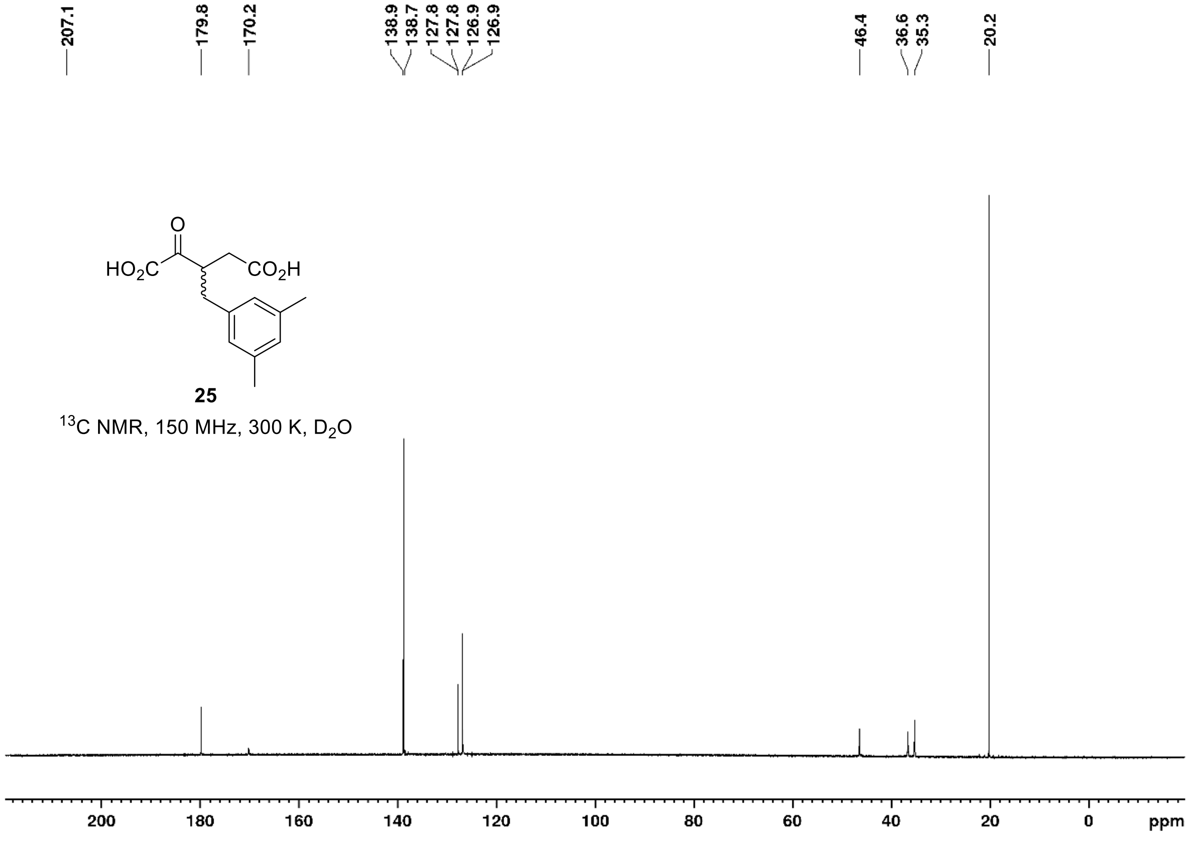

25

^{13}C NMR, 150 MHz, 300 K, D_2O

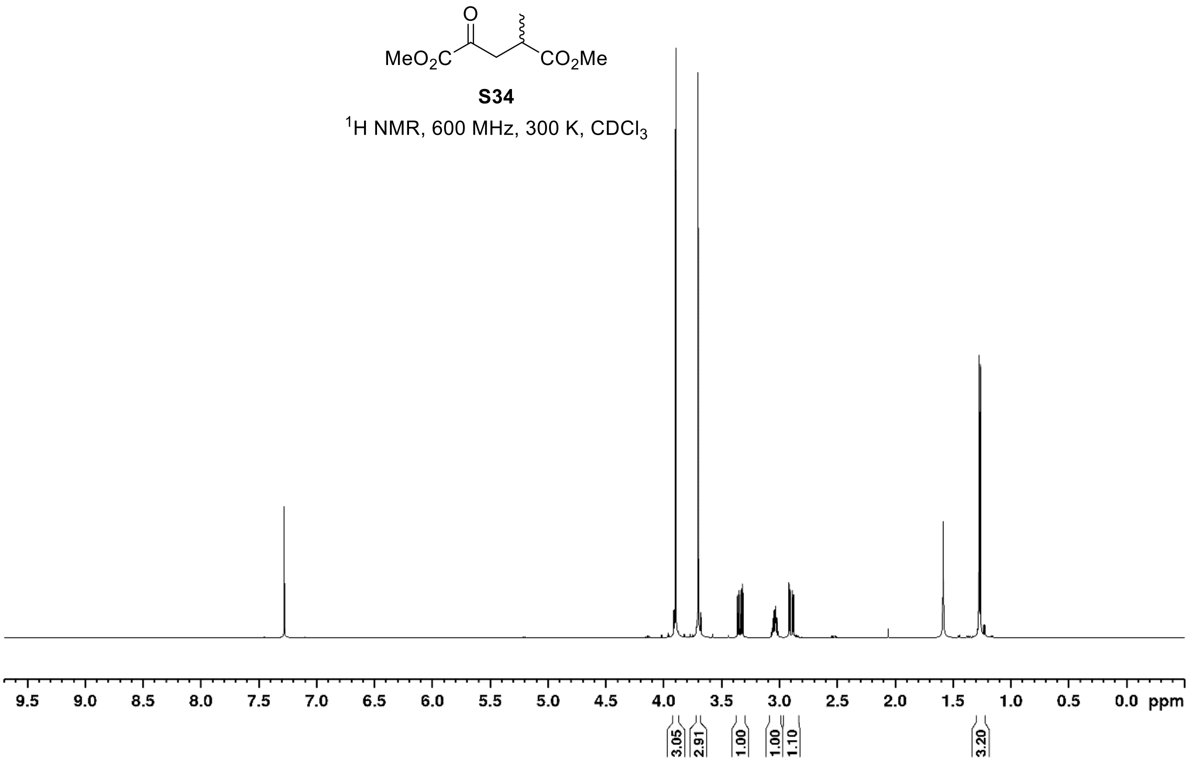
MeO2C
O
CO2Me
S34
1H NMR, 600 MHz, 300 K, CDCl3
9.5 9.0 8.5 8.0 7.5 7.0 6.5 6.0 5.5 5.0 4.5 4.0 3.5 3.0 2.5 2.0 1.5 1.0 0.5 0.0 ppm
3.05 2.91 1.00 1.00 1.10 3.20

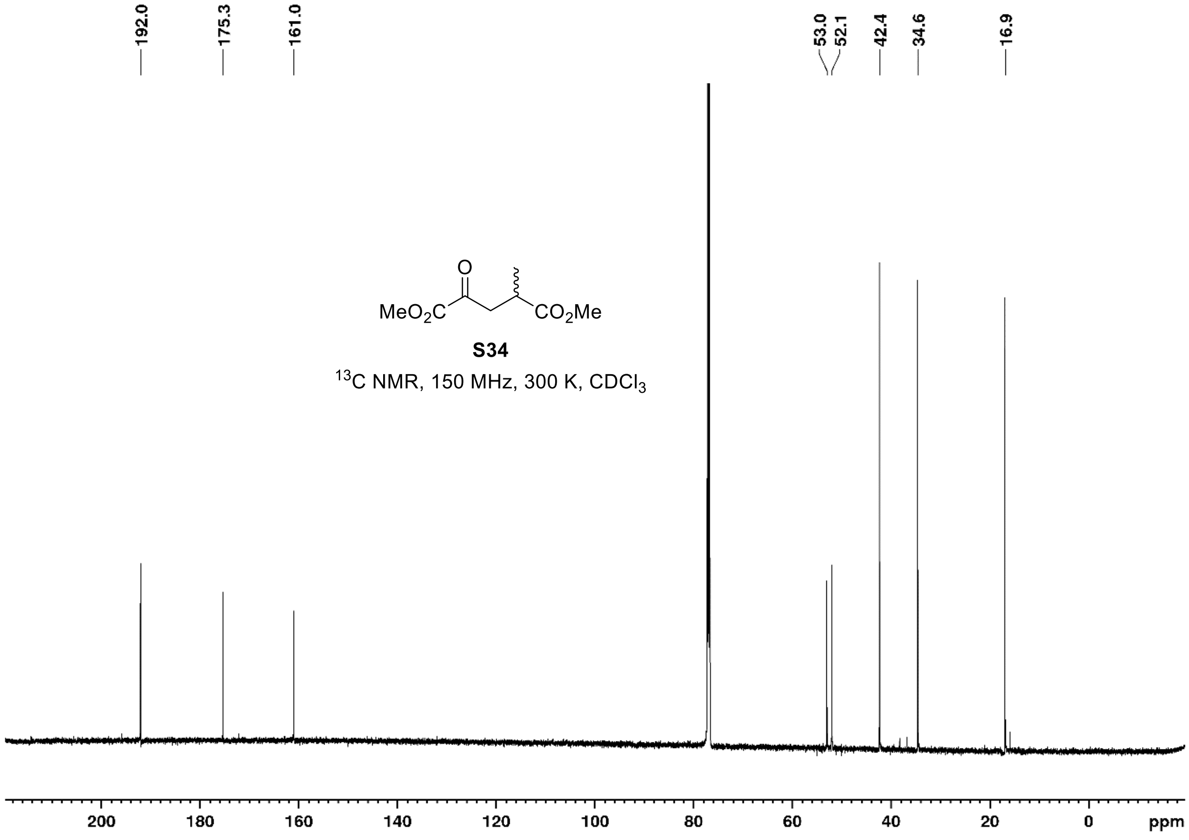
192.0
175.3
161.0
53.0
52.1
42.4
34.6
16.9
MeO2C
O
CO2Me
S34
13C NMR, 150 MHz, 300 K, CDCl3
200 180 160 140 120 100 80 60 40 20 0 ppm

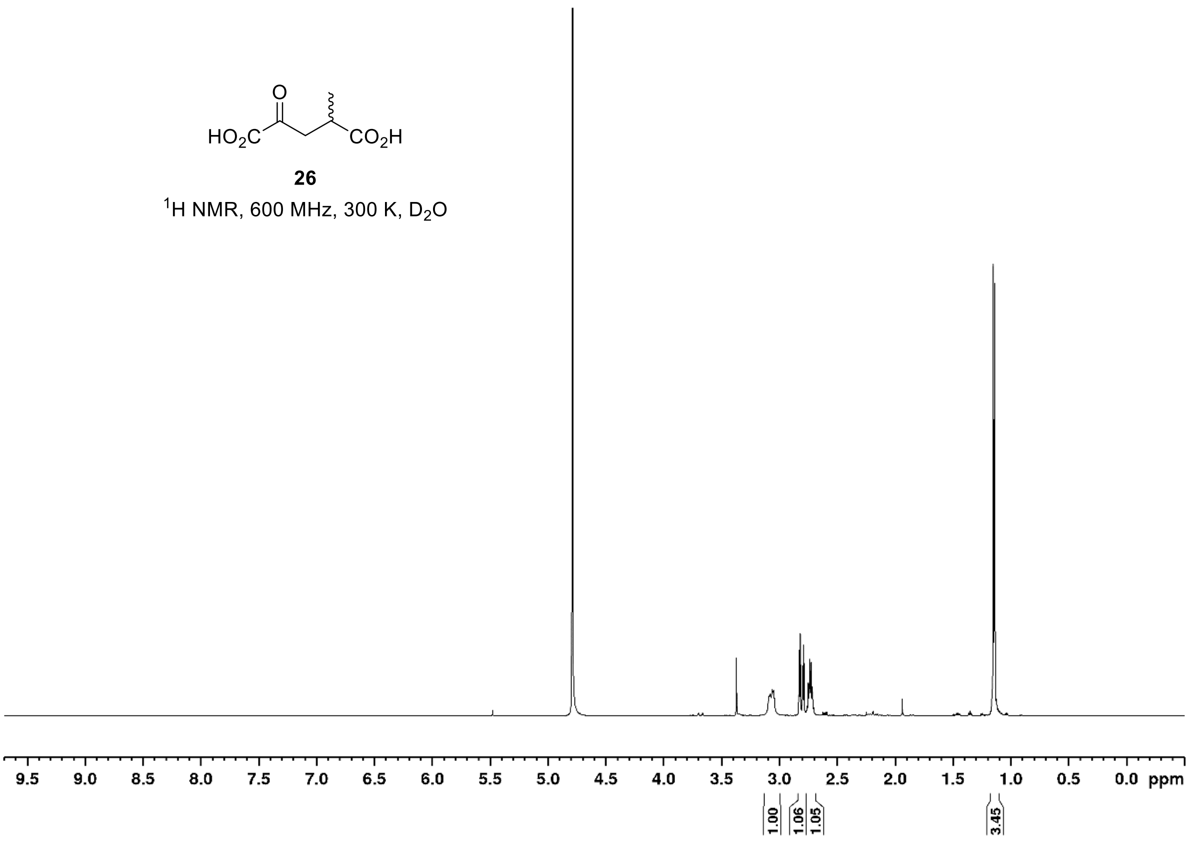

O
HO2C
CO2H
26
1H NMR, 600 MHz, 300 K, D2O
9.5 9.0 8.5 8.0 7.5 7.0 6.5 6.0 5.5 5.0 4.5 4.0 3.5 3.0 2.5 2.0 1.5 1.0 0.5 0.0 ppm
1.00
1.06
1.05
3.45

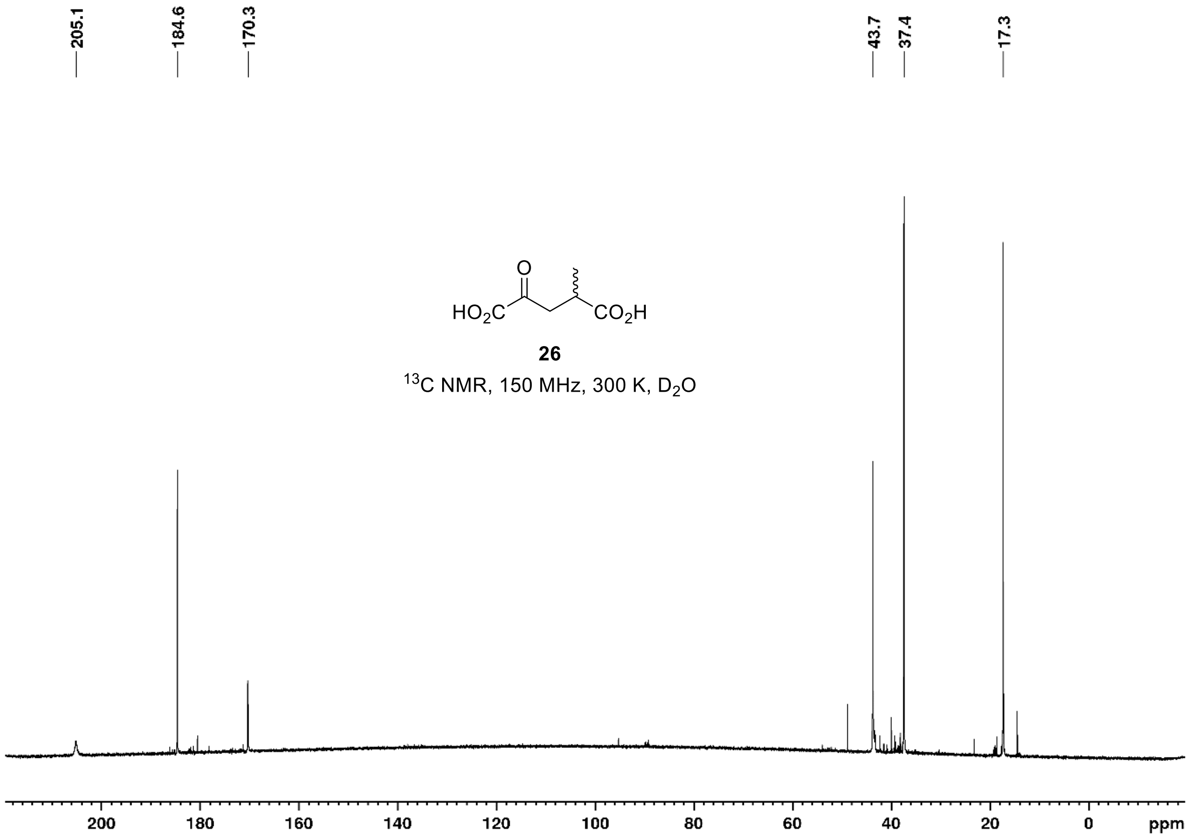

205.1
184.6
170.3
43.7
37.4
17.3
O
HO2C
CO2H
26
13C NMR, 150 MHz, 300 K, D2O
200 180 160 140 120 100 80 60 40 20 0 ppm

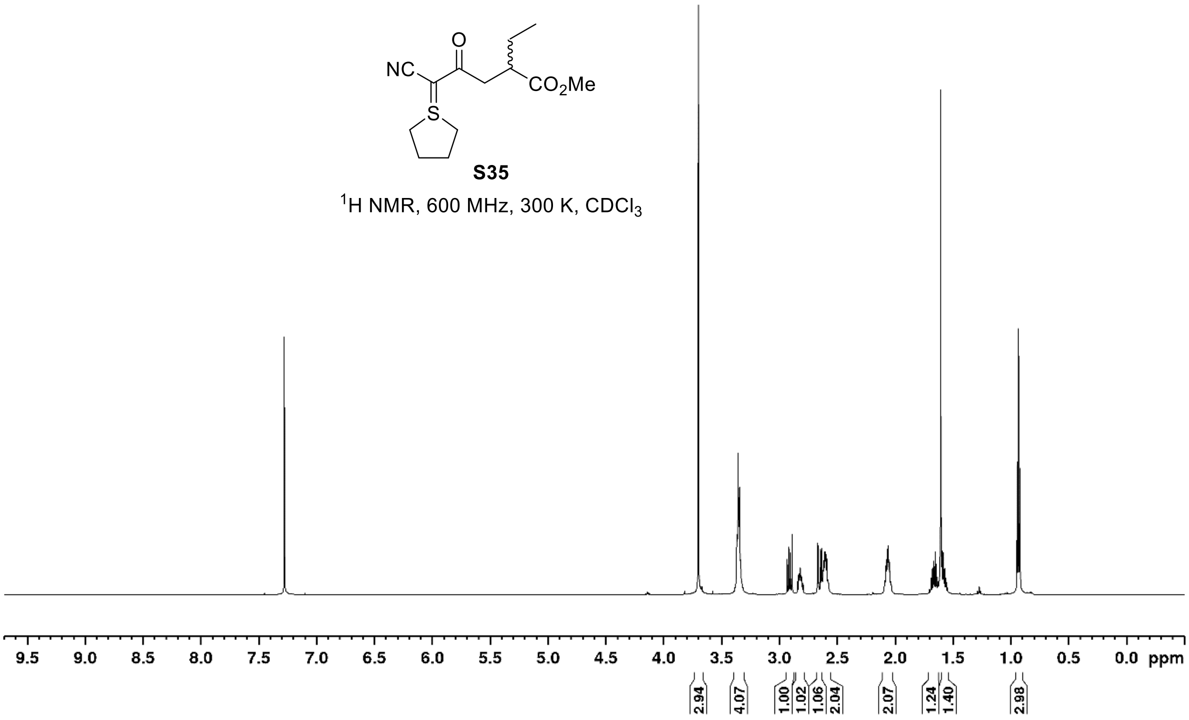
NC
O
CO2Me
S
S35
1H NMR, 600 MHz, 300 K, CDCl3
9.5 9.0 8.5 8.0 7.5 7.0 6.5 6.0 5.5 5.0 4.5 4.0 3.5 3.0 2.5 2.0 1.5 1.0 0.5 0.0 ppm
2.94 4.07 1.00 1.02 1.06 2.04 2.07 1.24 1.40 2.98

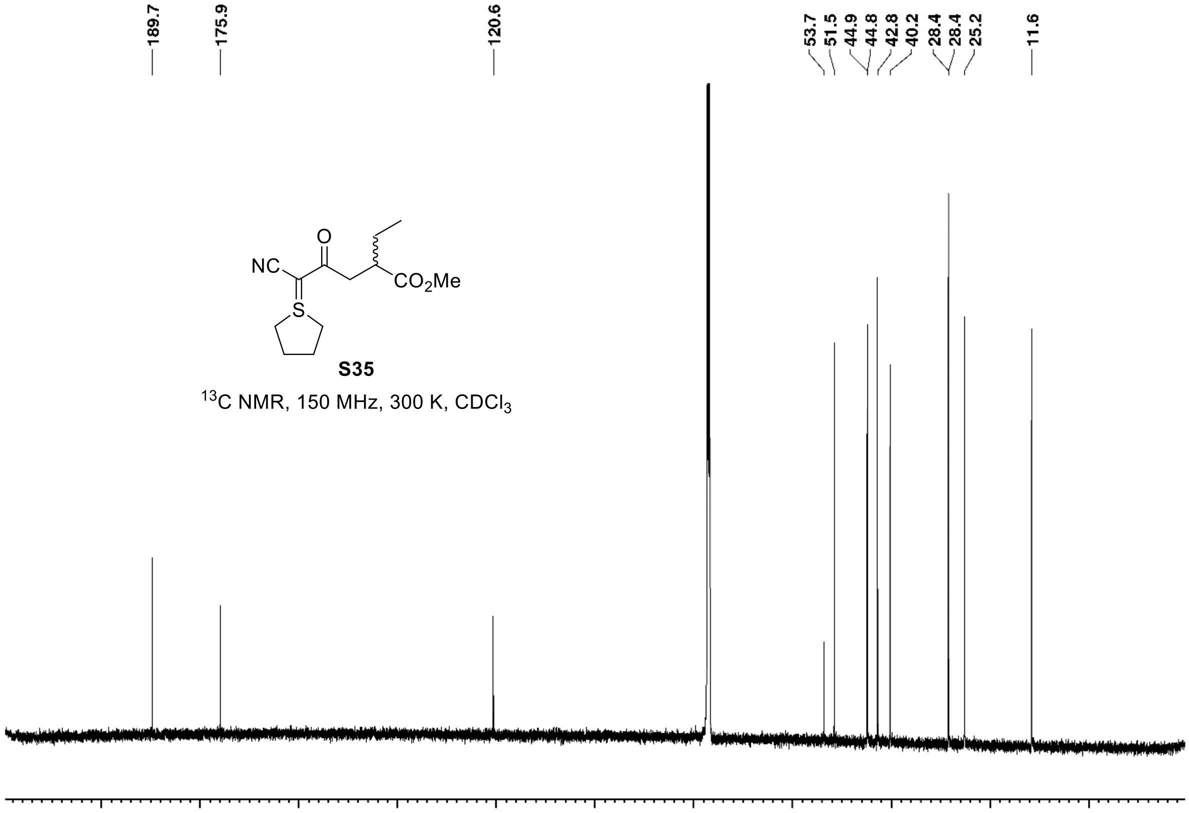
189.7
175.9
120.6
53.7 51.5 44.9 44.8 42.8 40.2 28.4 28.4 25.2
11.6
NC
O
CO2Me
S
S35
13C NMR, 150 MHz, 300 K, CDCl3
200 180 160 140 120 100 80 60 40 20 0 ppm

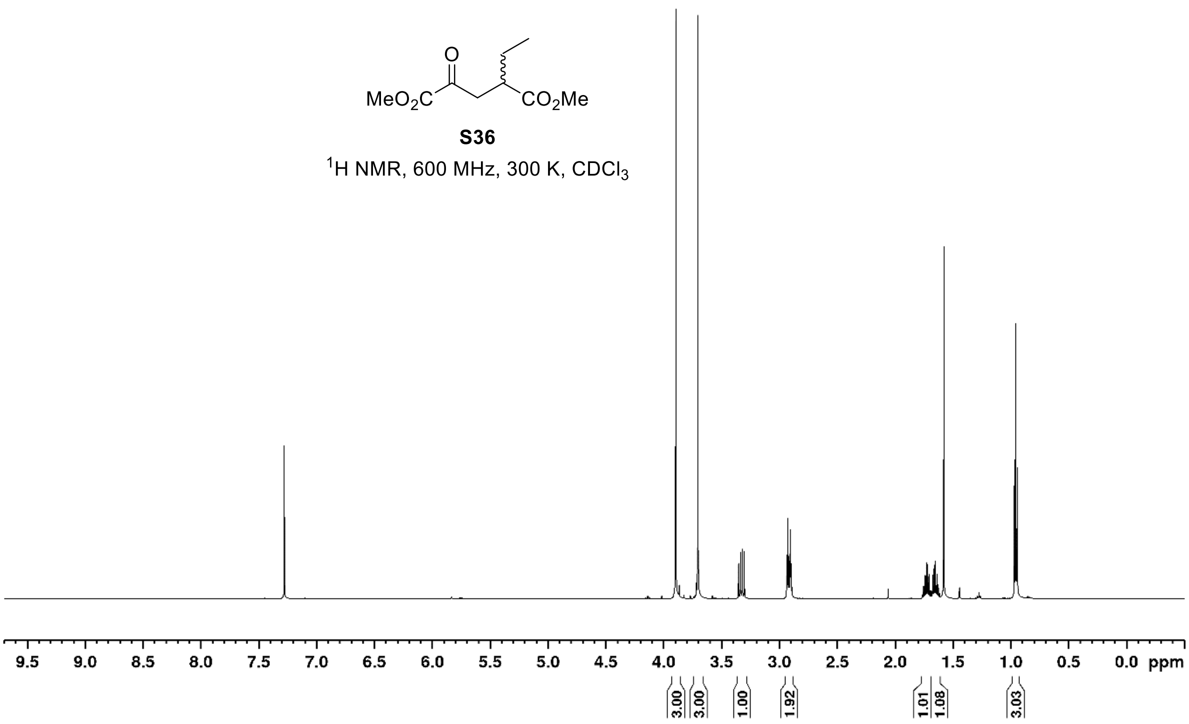

MeO2C
CO2Me
S36
1H NMR, 600 MHz, 300 K, CDCl3
9.5 9.0 8.5 8.0 7.5 7.0 6.5 6.0 5.5 5.0 4.5 4.0 3.5 3.0 2.5 2.0 1.5 1.0 0.5 0.0 ppm
3.00 3.00 1.00 1.92 1.01 1.08 3.03

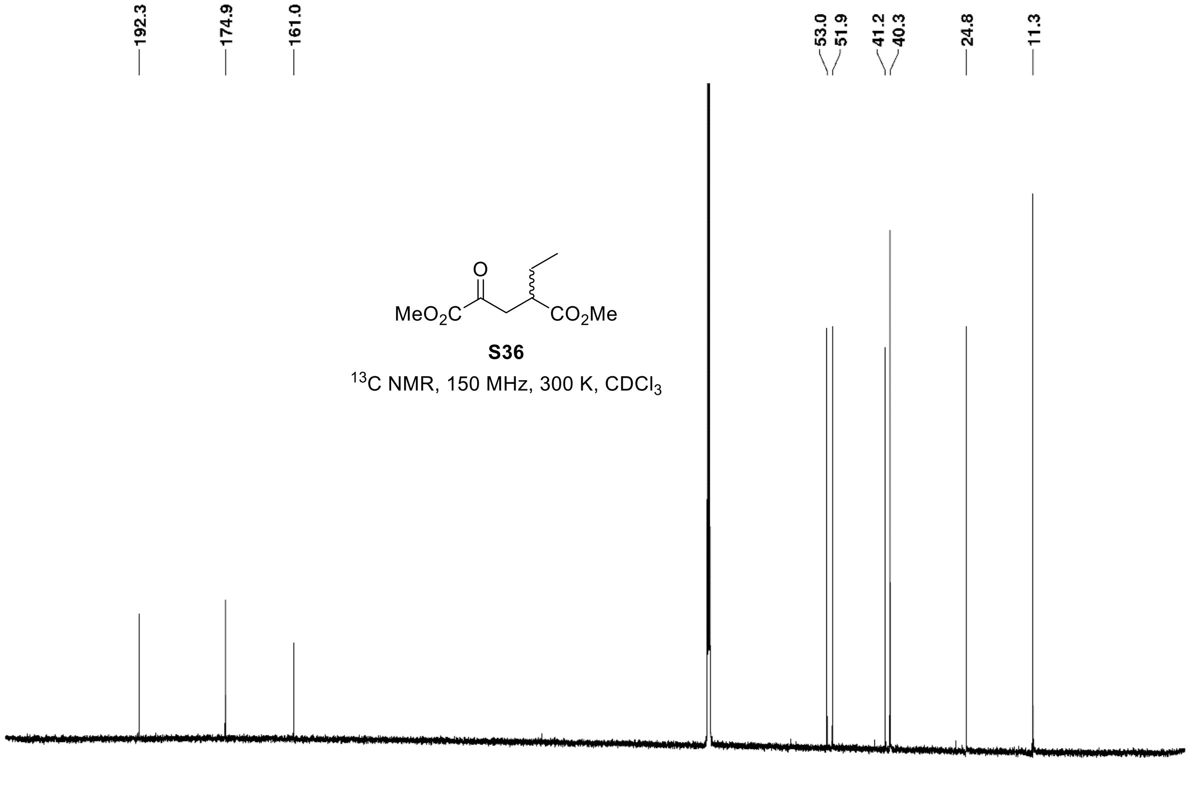

192.3
174.9
161.0
53.0
51.9
41.2
40.3
24.8
11.3
MeO2C
CO2Me
S36
13C NMR, 150 MHz, 300 K, CDCl3
200 180 160 140 120 100 80 60 40 20 0 ppm

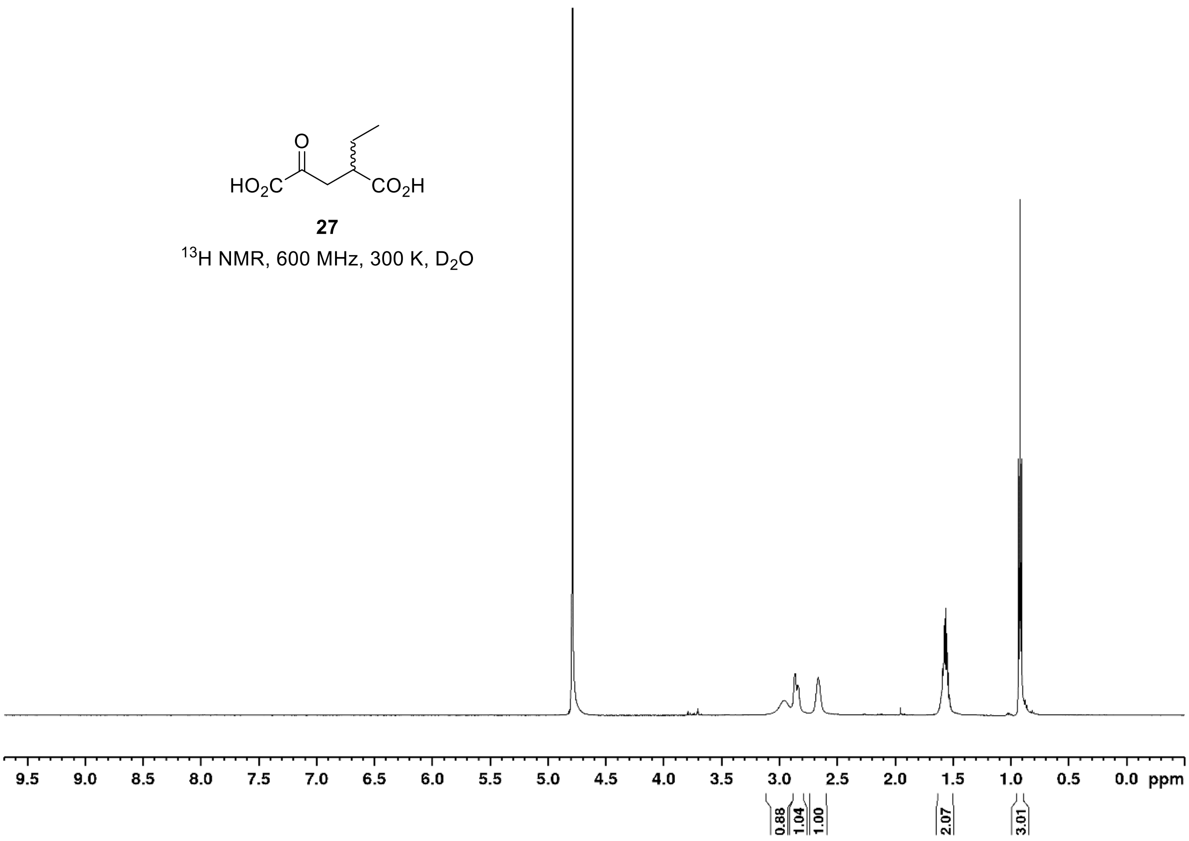
O
HO2C
CO2H
27
13H NMR, 600 MHz, 300 K, D2O
9.5 9.0 8.5 8.0 7.5 7.0 6.5 6.0 5.5 5.0 4.5 4.0 3.5 3.0 2.5 2.0 1.5 1.0 0.5 0.0 ppm
0.88 1.04 1.00 2.07 3.01

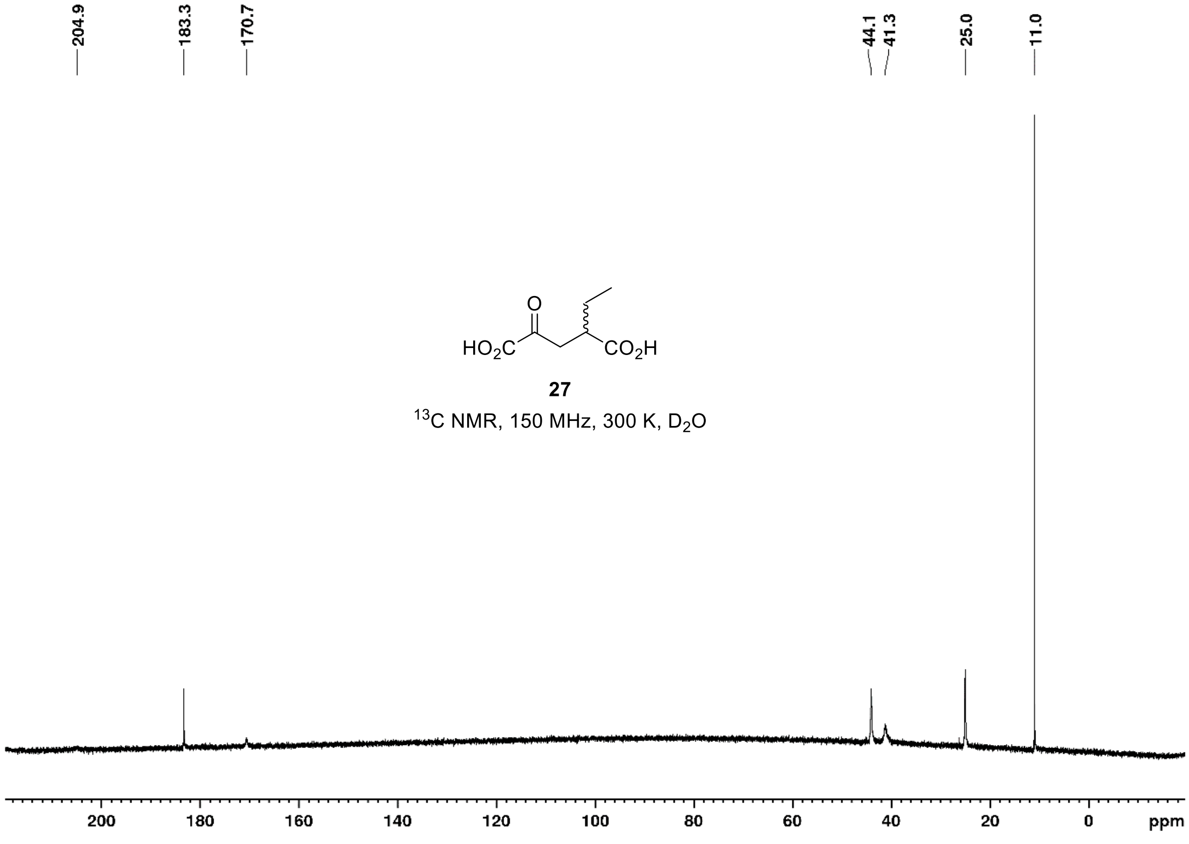
204.9
183.3
170.7
44.1
41.3
25.0
11.0
O
HO2C
CO2H
27
13C NMR, 150 MHz, 300 K, D2O
200 180 160 140 120 100 80 60 40 20 0 ppm

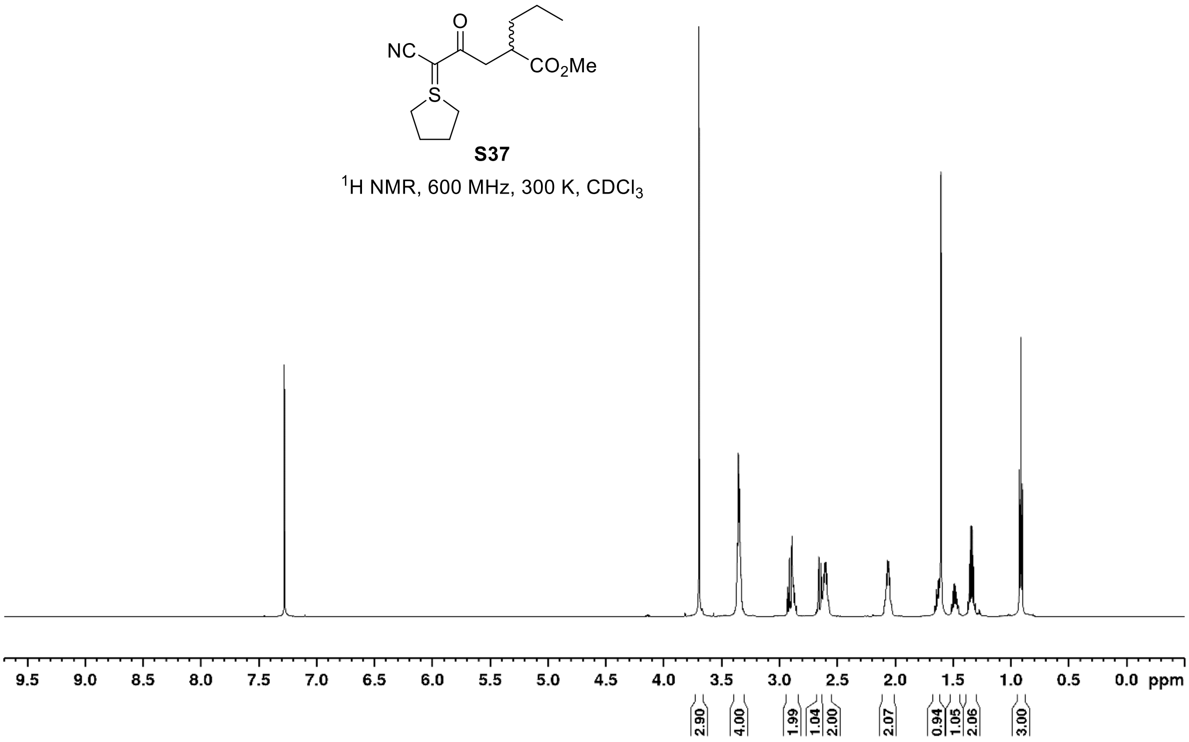
NC
O
CO2Me
S
S37
1H NMR, 600 MHz, 300 K, CDCl3
9.5 9.0 8.5 8.0 7.5 7.0 6.5 6.0 5.5 5.0 4.5 4.0 3.5 3.0 2.5 2.0 1.5 1.0 0.5 0.0 ppm
2.90 4.00 1.99 1.04 2.00 2.07 0.94 1.05 2.06 3.00

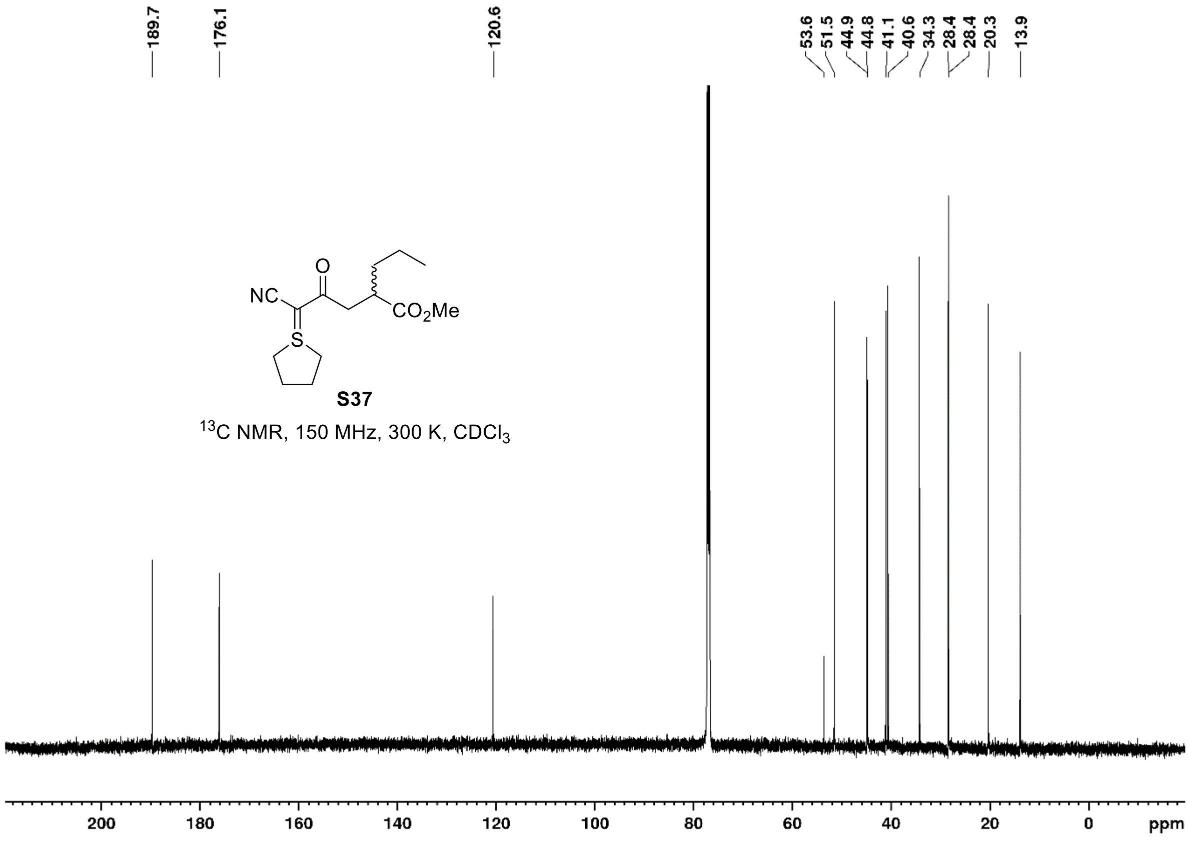
189.7
176.1
120.6
53.6 51.5 44.9 44.8 41.1 40.6 34.3 28.4 28.4 20.3 13.9
NC
O
CO2Me
S
S37
13C NMR, 150 MHz, 300 K, CDCl3
200 180 160 140 120 100 80 60 40 20 0 ppm

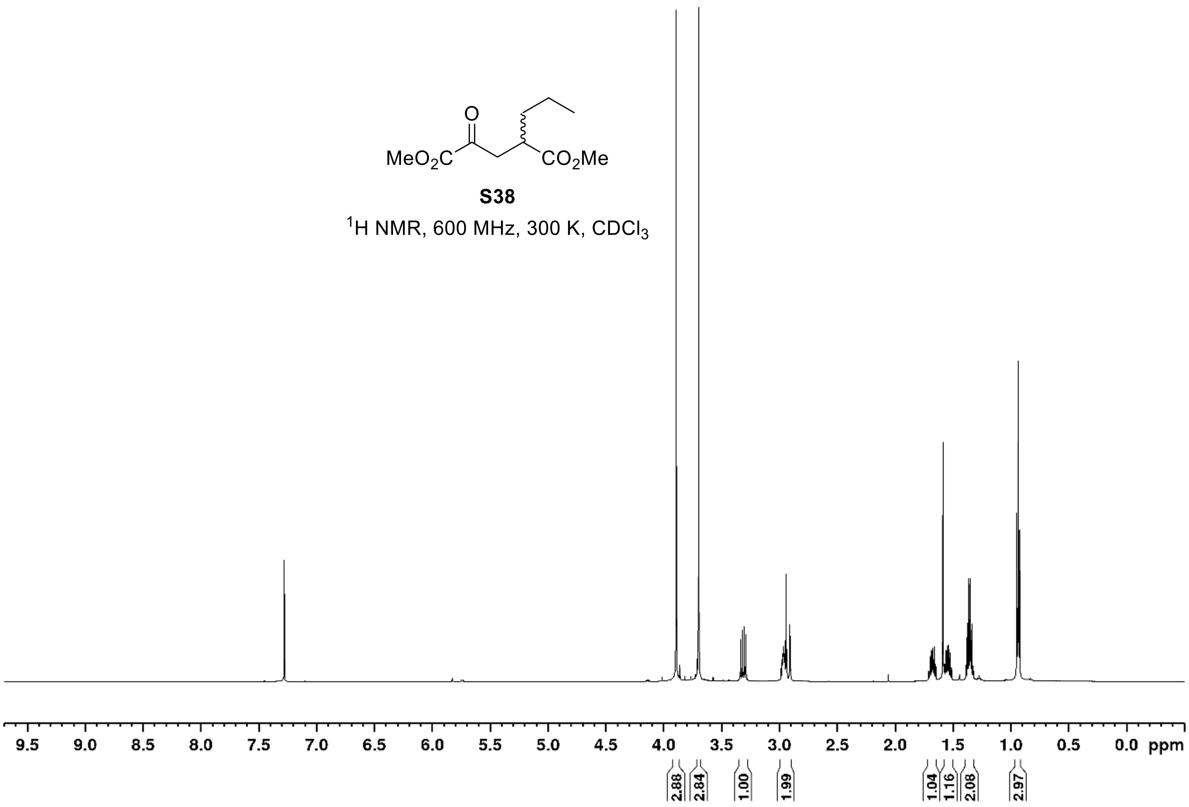
O
MeO2C
CO2Me
S38
1H NMR, 600 MHz, 300 K, CDCl3
9.5 9.0 8.5 8.0 7.5 7.0 6.5 6.0 5.5 5.0 4.5 4.0 3.5 3.0 2.5 2.0 1.5 1.0 0.5 0.0 ppm
2.88 2.84 1.00 1.99 1.04 1.16 2.08 2.97

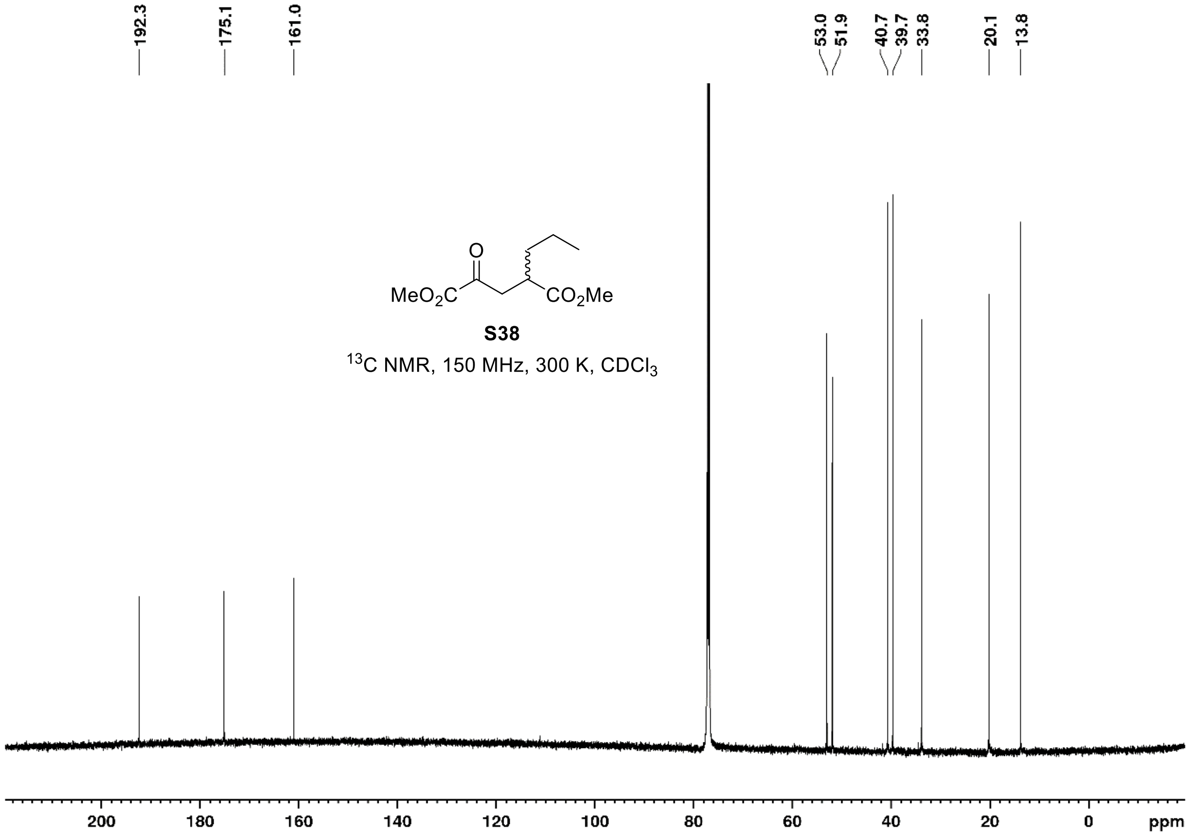
192.3
175.1
161.0
53.0
51.9
40.7
39.7
33.8
20.1
13.8
O
MeO2C
CO2Me
S38
13C NMR, 150 MHz, 300 K, CDCl3
200 180 160 140 120 100 80 60 40 20 0 ppm

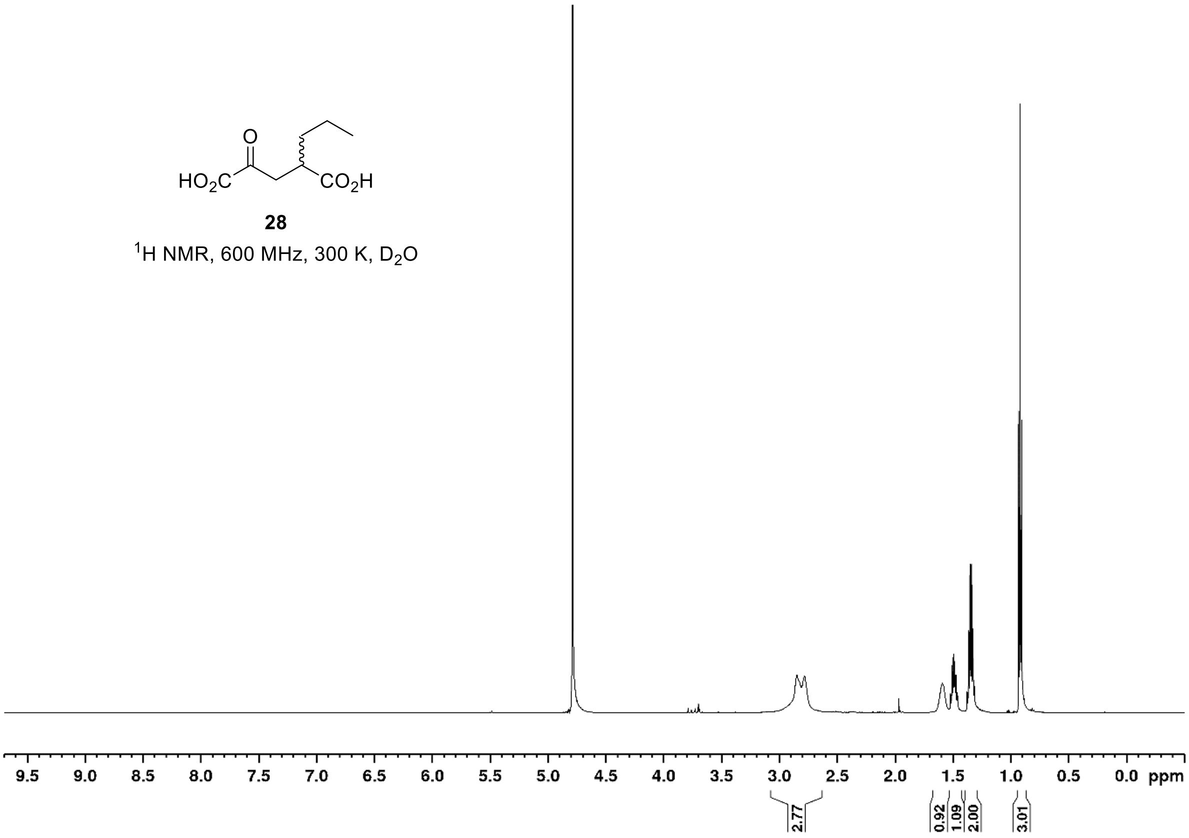

28

^{1}H NMR, 600 MHz, 300 K, D_2O

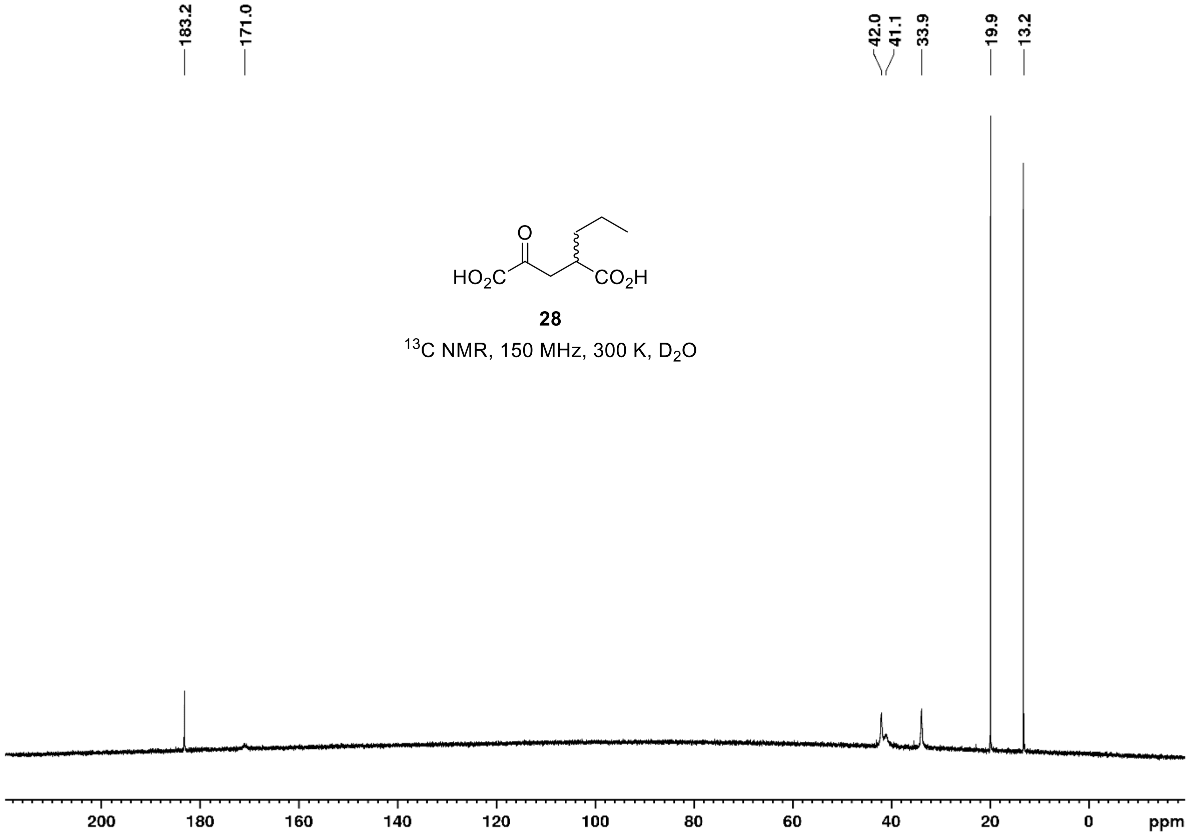

28

^{13}C NMR, 150 MHz, 300 K, D_2O

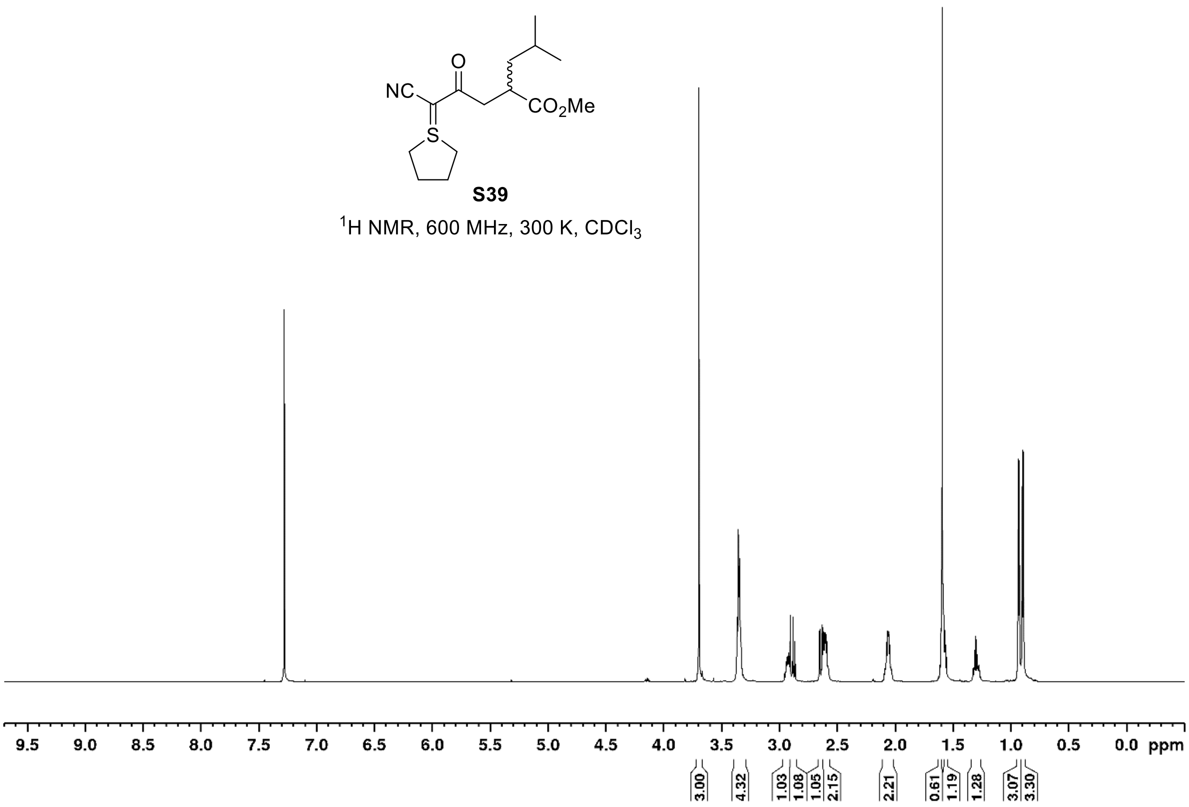
NC
O
S
CO2Me
S39
1H NMR, 600 MHz, 300 K, CDCl3
9.5 9.0 8.5 8.0 7.5 7.0 6.5 6.0 5.5 5.0 4.5 4.0 3.5 3.0 2.5 2.0 1.5 1.0 0.5 0.0 ppm
3.00
4.32
1.03
1.08
1.05
2.15
2.21
0.61
1.19
1.28
3.07
3.30

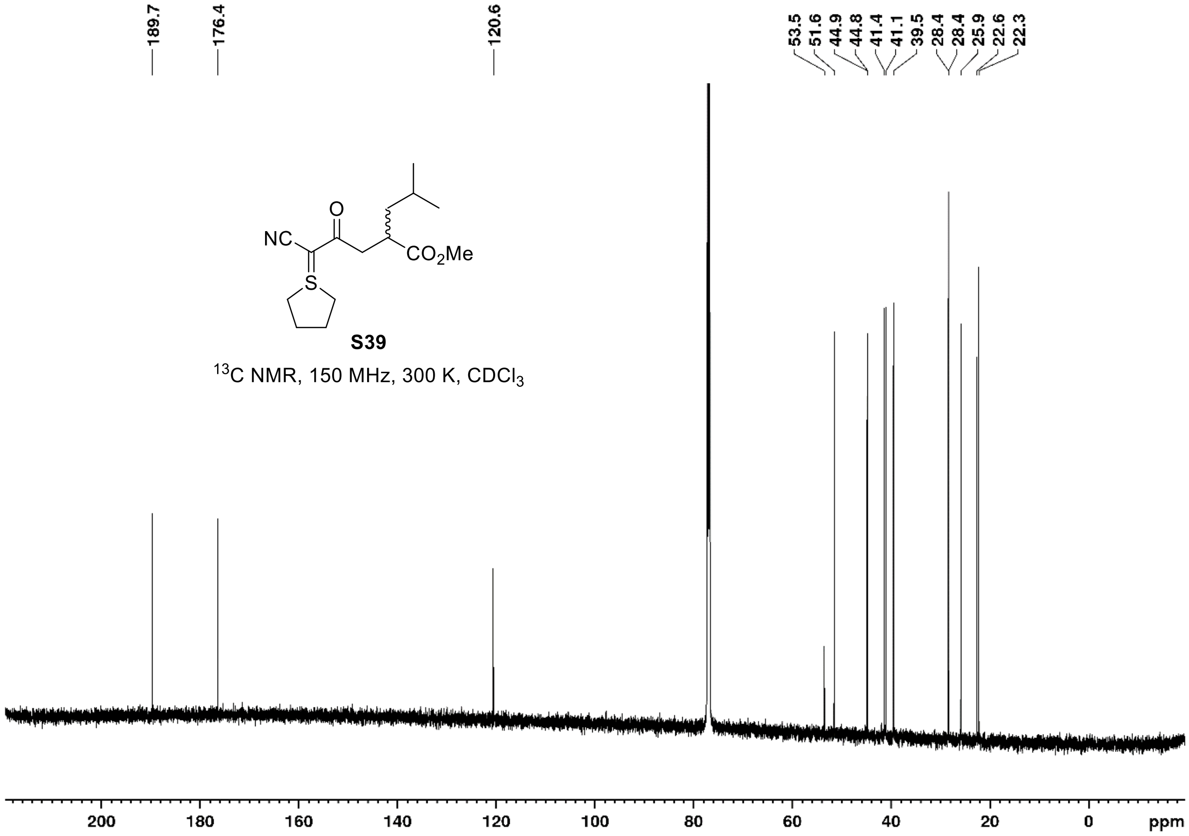
189.7
176.4
120.6
53.5
51.6
44.9
44.8
41.4
41.1
39.5
28.4
28.4
25.9
22.6
22.3
NC
O
S
CO2Me
S39
13C NMR, 150 MHz, 300 K, CDCl3
200 180 160 140 120 100 80 60 40 20 0 ppm

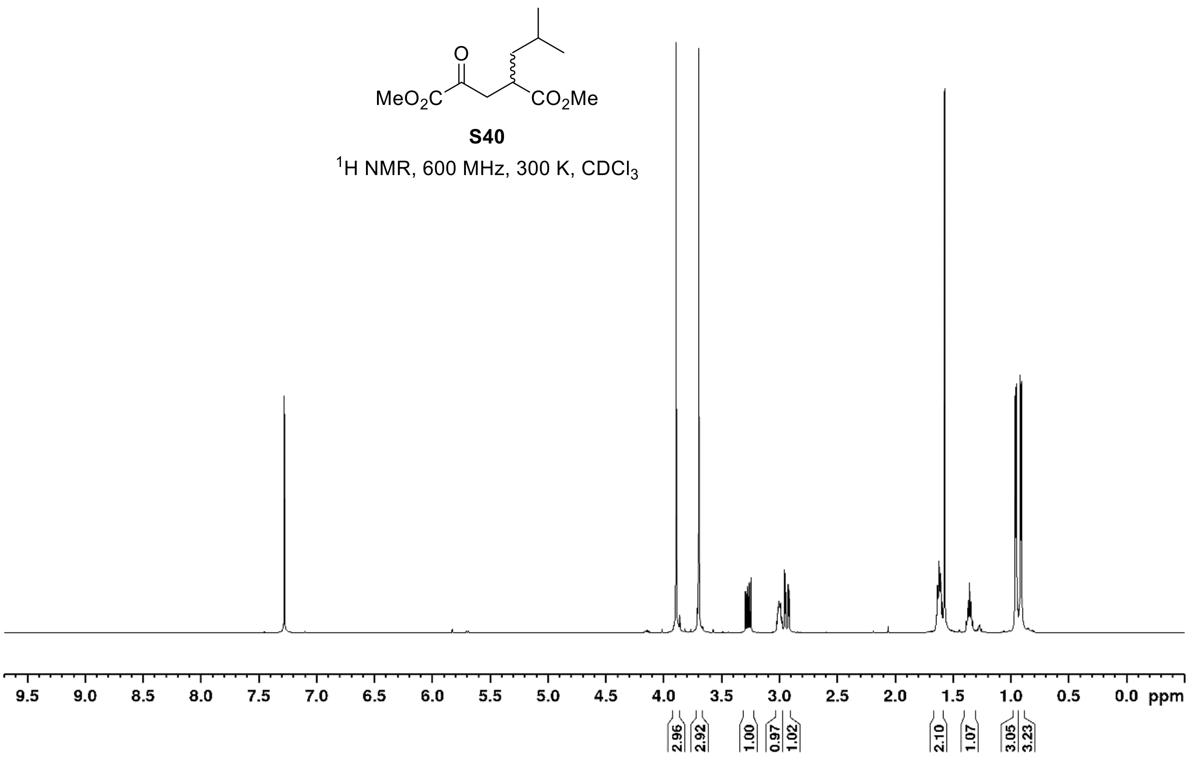
O
MeO_2C
CO_2Me
S40
^{1}H NMR, 600 MHz, 300 K, $CDCl_3$
9.5 9.0 8.5 8.0 7.5 7.0 6.5 6.0 5.5 5.0 4.5 4.0 3.5 3.0 2.5 2.0 1.5 1.0 0.5 0.0 ppm
2.96 2.92 1.00 0.97 1.02 2.10 1.07 3.05 3.23

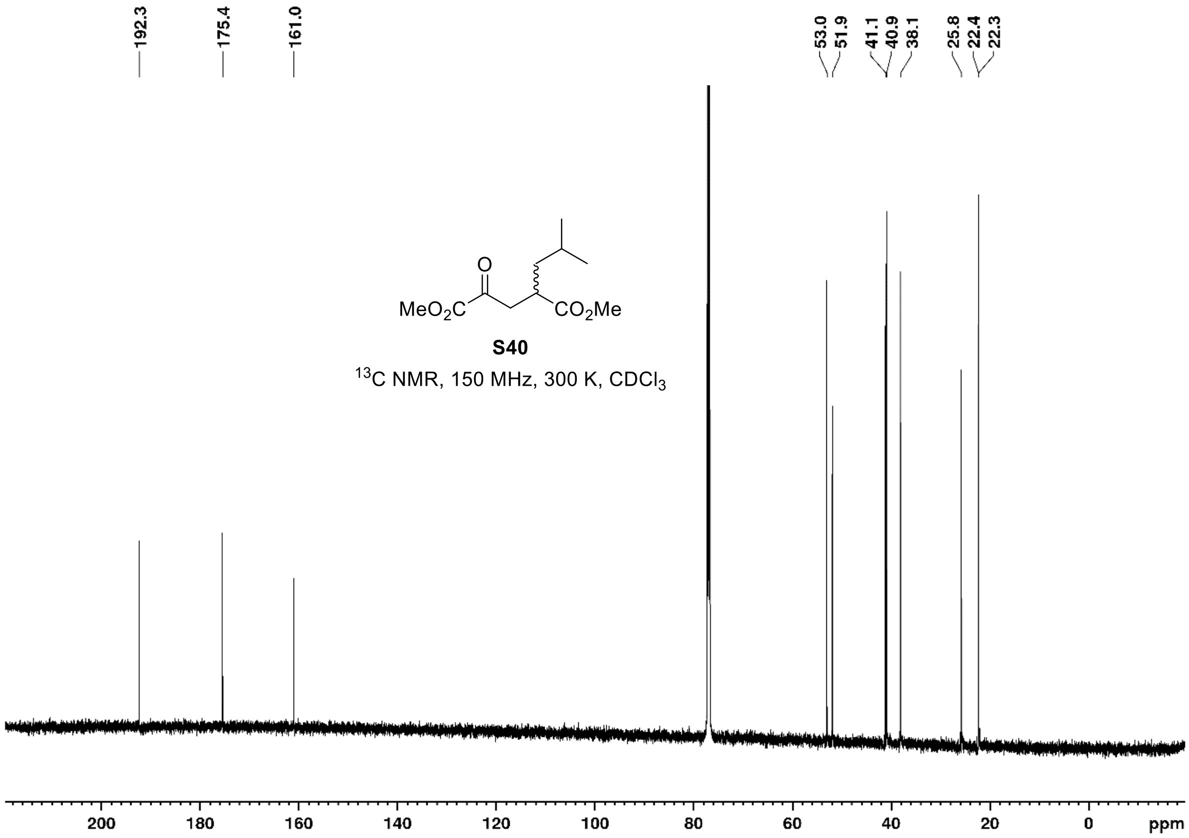
192.3
175.4
161.0
53.0 51.9 41.1 40.9 38.1 25.8 22.4 22.3
O
MeO_2C
CO_2Me
S40
^{13}C NMR, 150 MHz, 300 K, $CDCl_3$
200 180 160 140 120 100 80 60 40 20 0 ppm

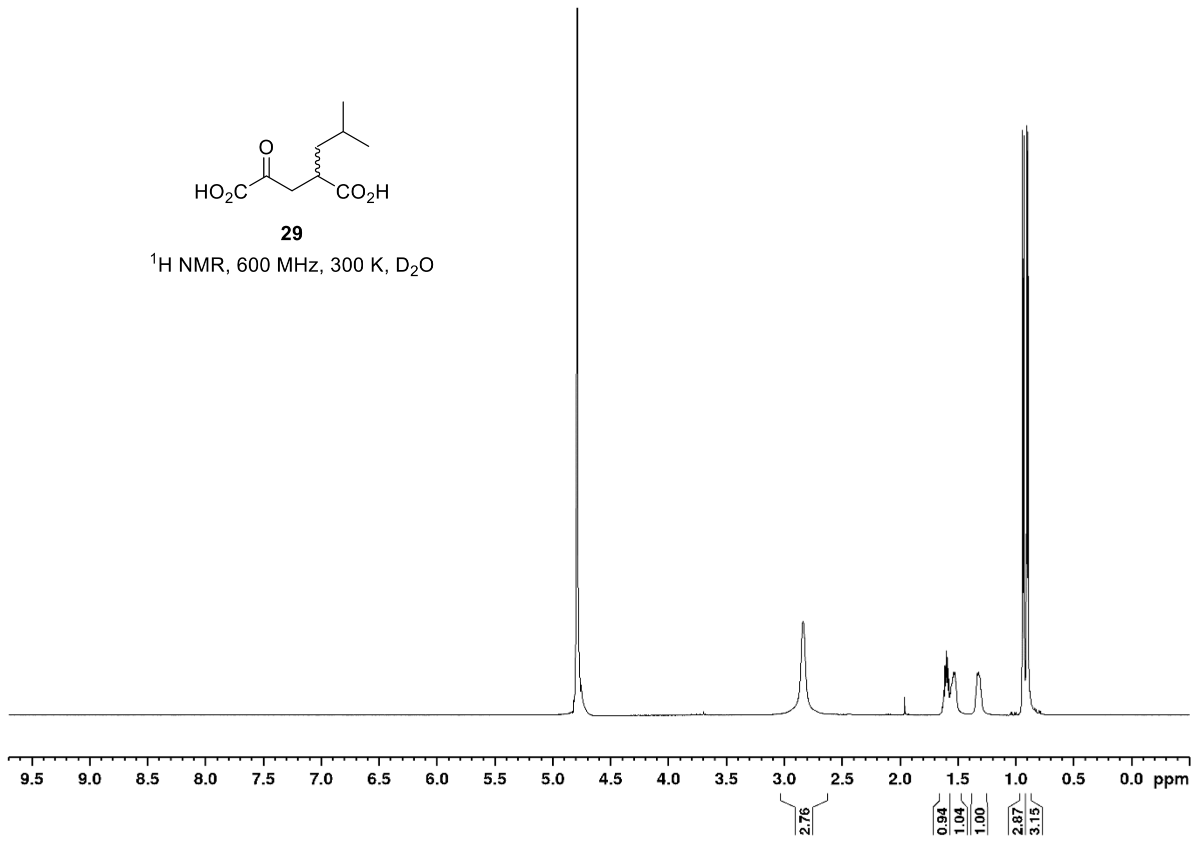

29

^{1}H NMR, 600 MHz, 300 K, D_2O

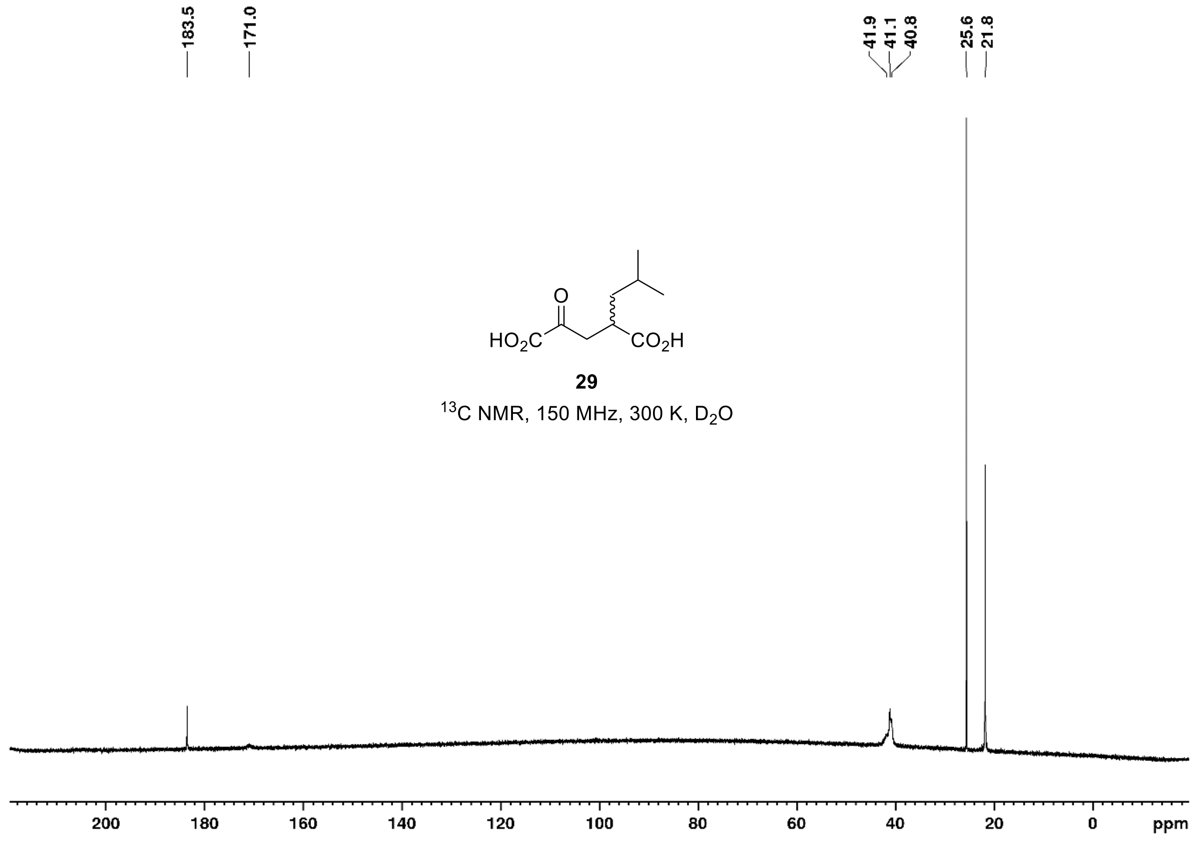

29

^{13}C NMR, 150 MHz, 300 K, D_2O

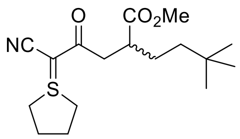

S41

^{1}H NMR, 600 MHz, 300 K, $CDCl_3$

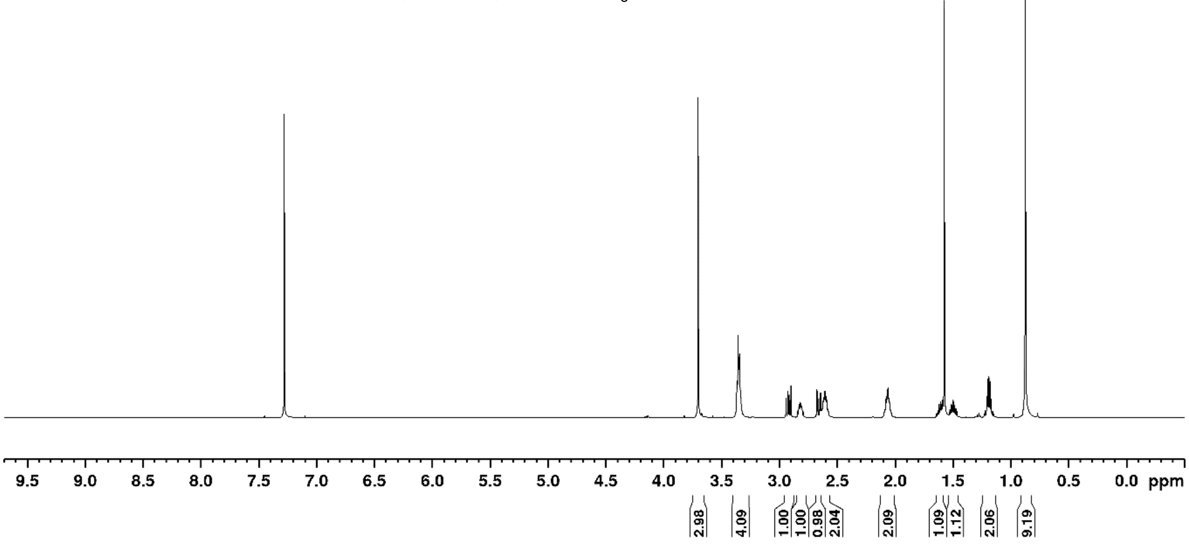

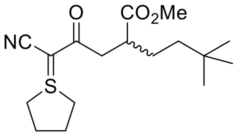

S41

^{13}C NMR, 150 MHz, 300 K, $CDCl_3$

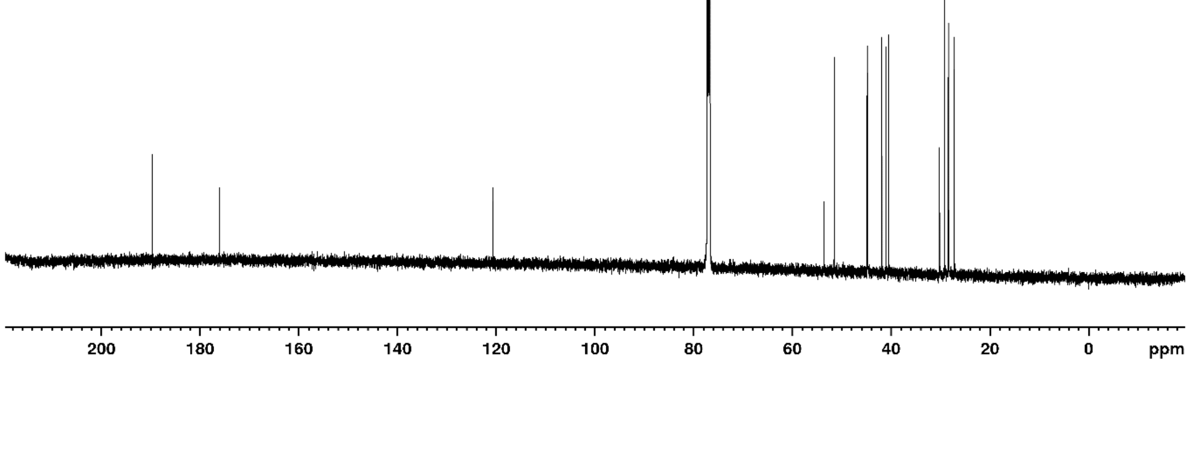

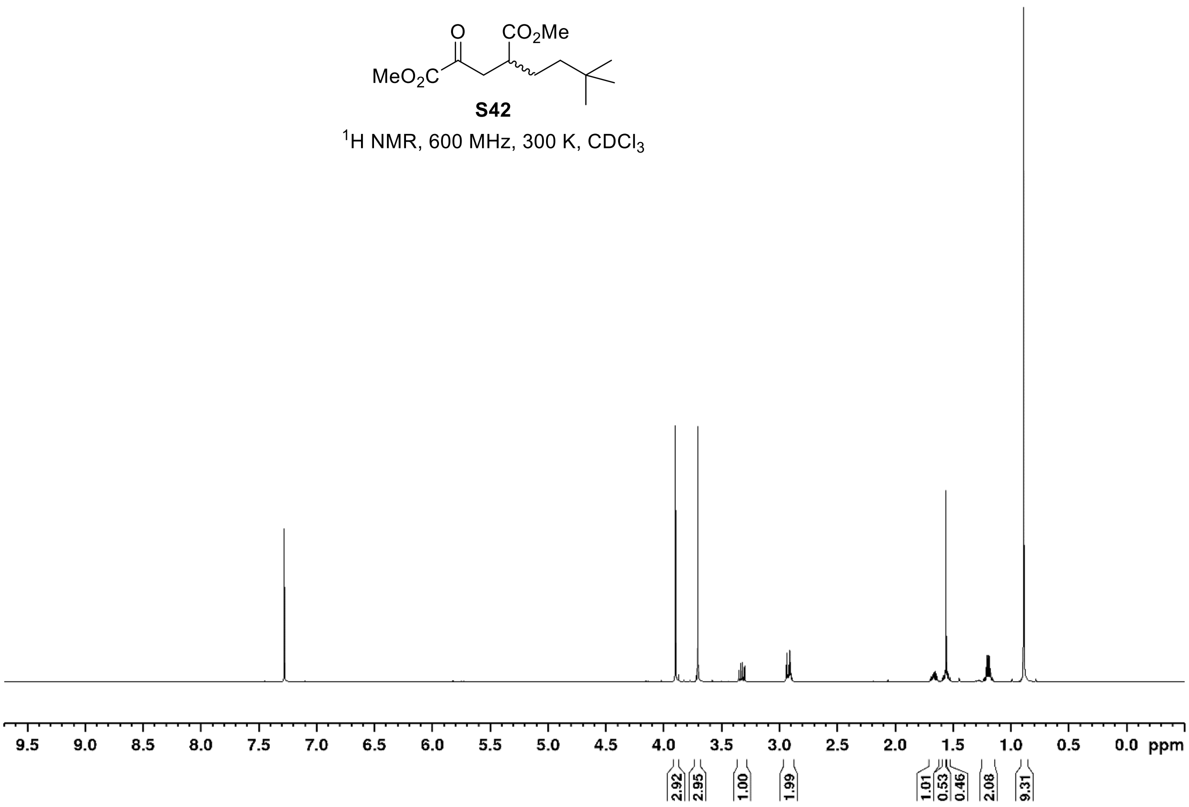
O
CO2Me
MeO2C
S42
1H NMR, 600 MHz, 300 K, CDCl3
9.5 9.0 8.5 8.0 7.5 7.0 6.5 6.0 5.5 5.0 4.5 4.0 3.5 3.0 2.5 2.0 1.5 1.0 0.5 0.0 ppm
2.92 2.95 1.00 1.99 1.01 0.53 0.46 2.08 9.31

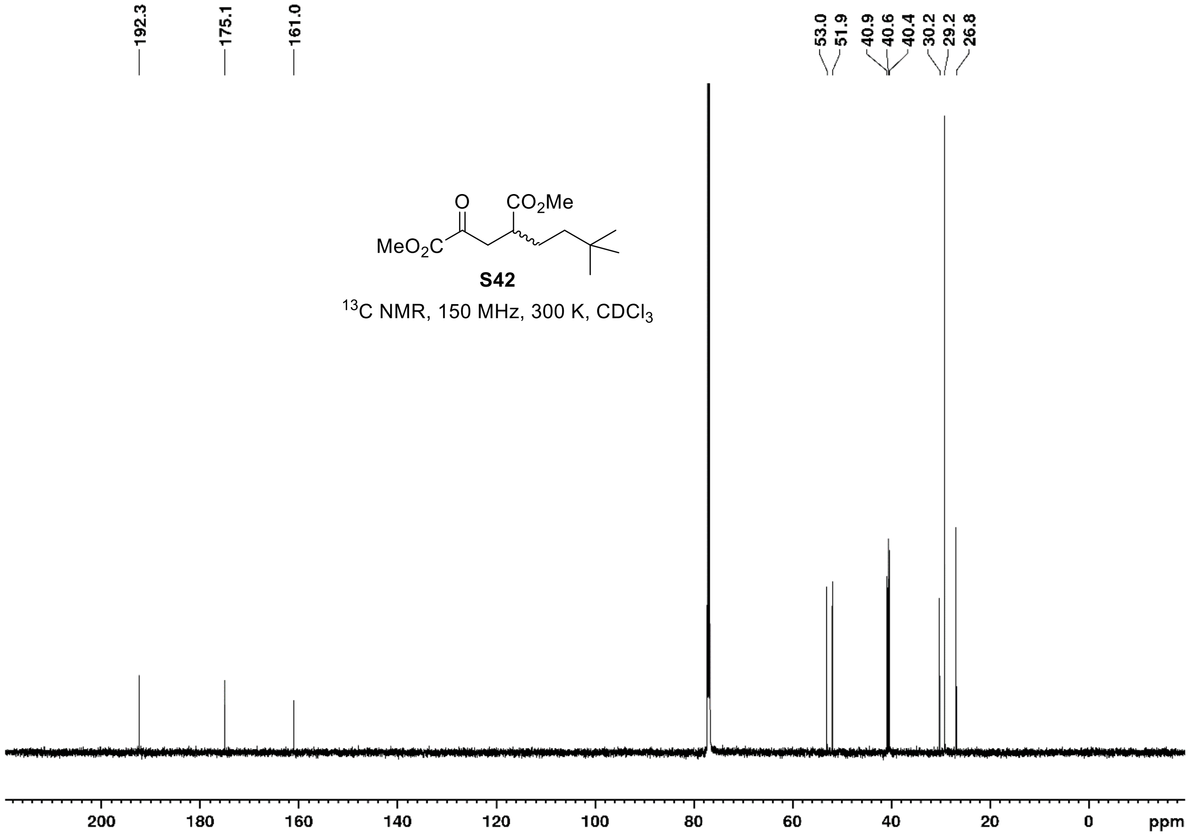
192.3
175.1
161.0
53.0 51.9 40.9 40.6 40.4 30.2 29.2 26.8
O
CO2Me
MeO2C
S42
13C NMR, 150 MHz, 300 K, CDCl3
200 180 160 140 120 100 80 60 40 20 0 ppm

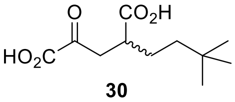

30

^{1}H NMR, 600 MHz, 300 K, D_2O

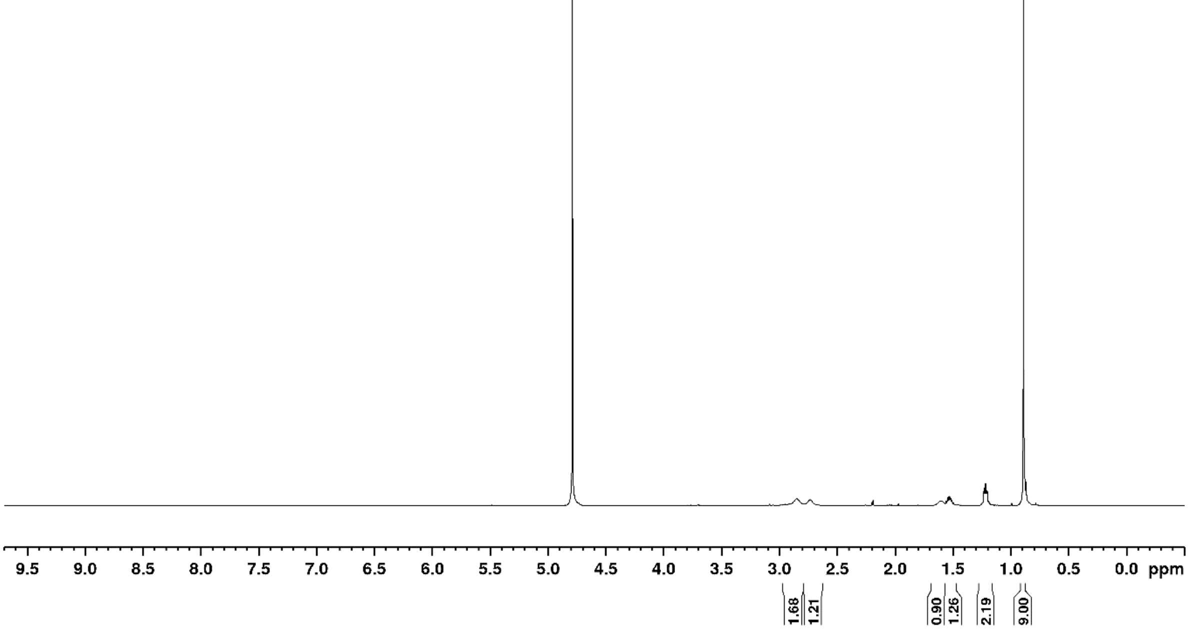

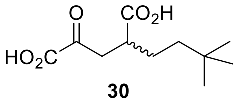

30

^{13}C NMR, 150 MHz, 300 K, D_2O

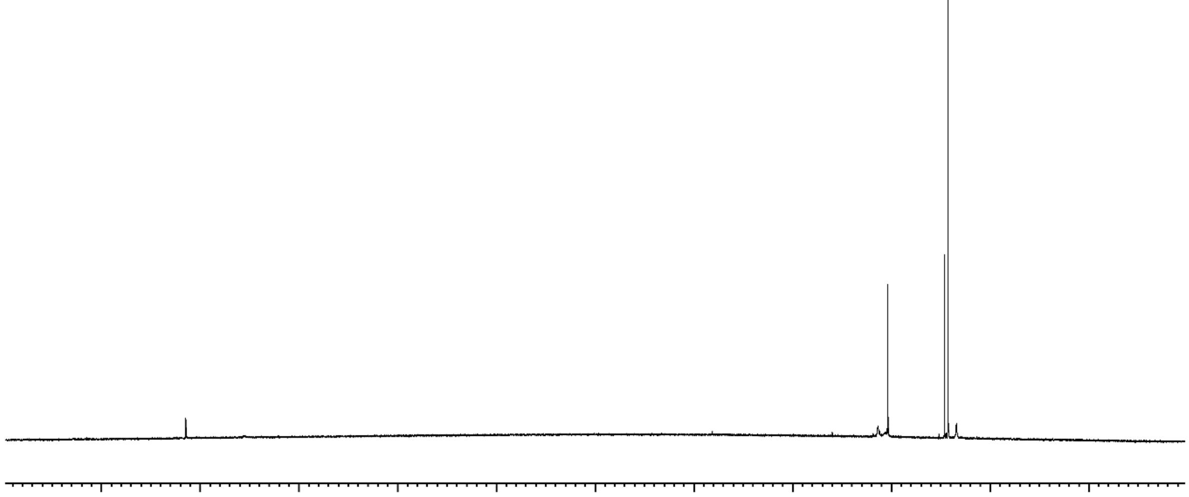

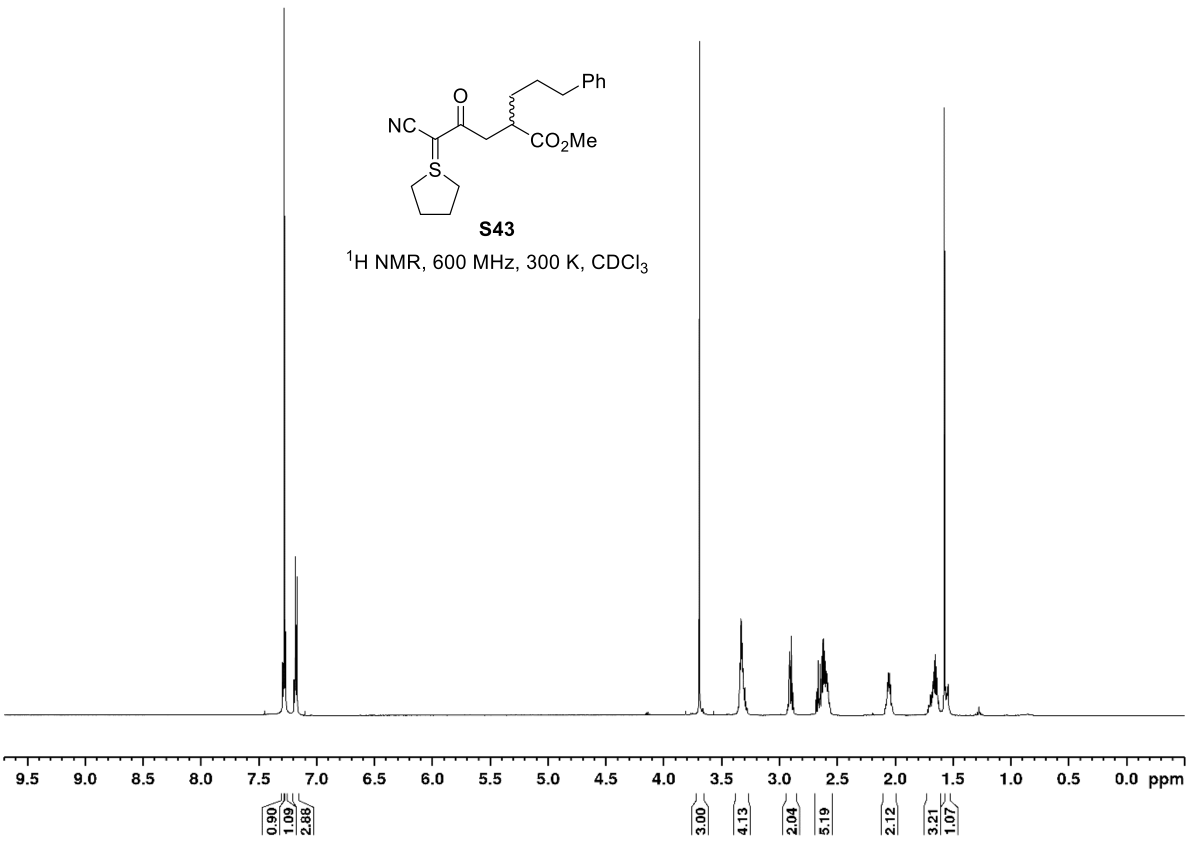
NC
O
Ph
CO2Me
S
S43
1H NMR, 600 MHz, 300 K, CDCl3
9.5 9.0 8.5 8.0 7.5 7.0 6.5 6.0 5.5 5.0 4.5 4.0 3.5 3.0 2.5 2.0 1.5 1.0 0.5 0.0 ppm
0.90
1.09
2.88
3.00
4.13
2.04
5.19
2.12
3.21
1.07

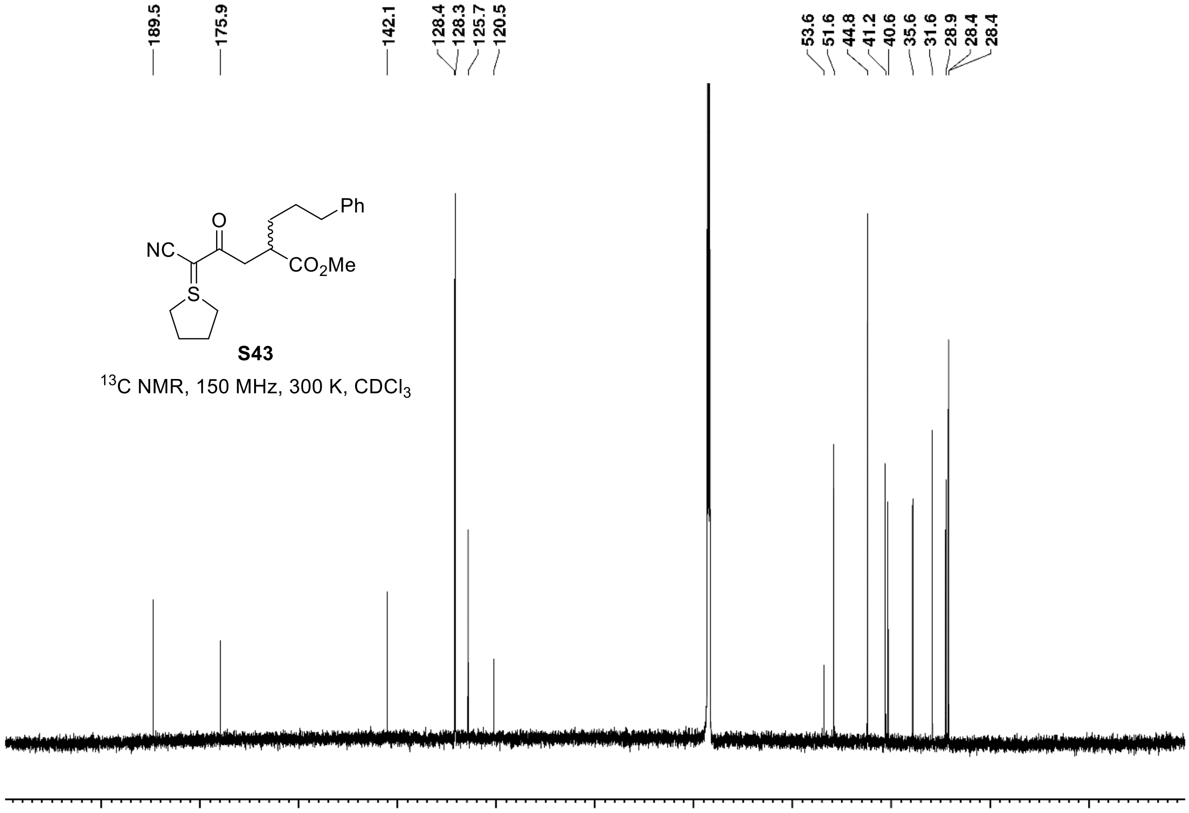
189.5
175.9
142.1
128.4
128.3
125.7
120.5
53.6
51.6
44.8
41.2
40.6
35.6
31.6
28.9
28.4
28.4
NC
O
Ph
CO2Me
S
S43
13C NMR, 150 MHz, 300 K, CDCl3
200 180 160 140 120 100 80 60 40 20 0 ppm

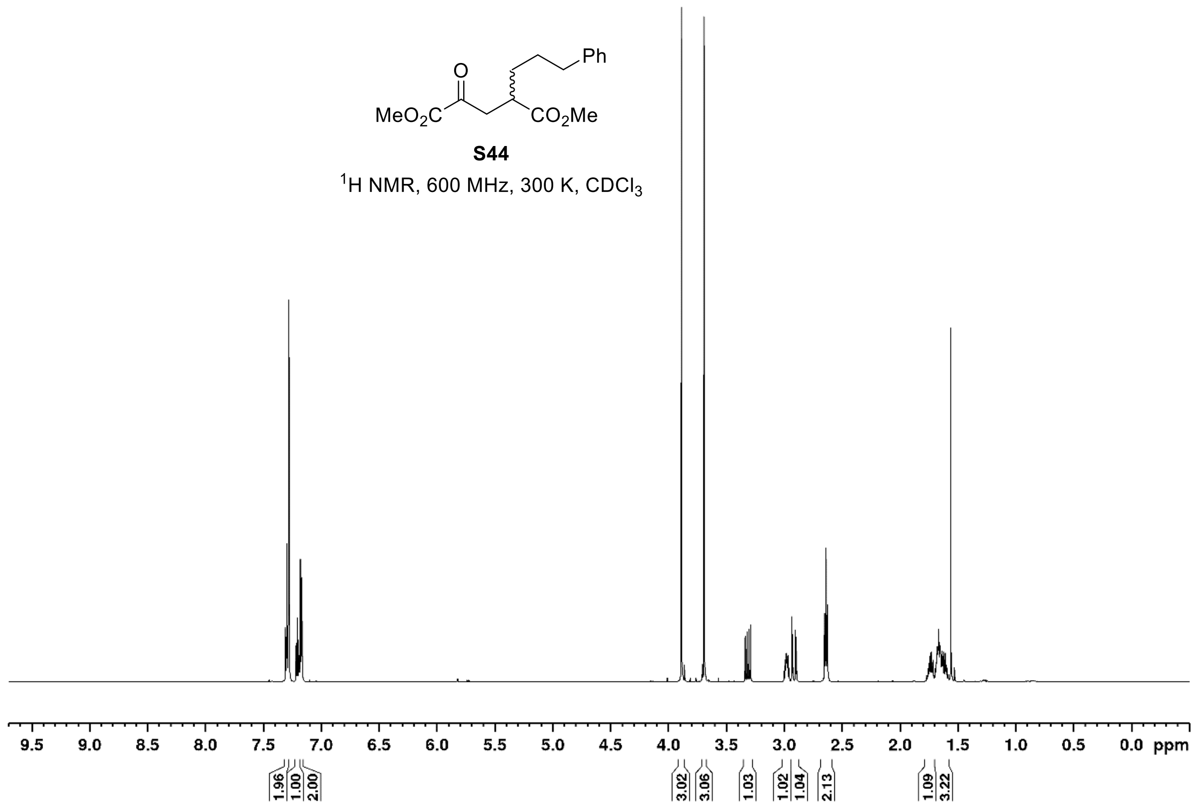

MeO2C
CO2Me
Ph
O
S44
1H NMR, 600 MHz, 300 K, CDCl3
9.5 9.0 8.5 8.0 7.5 7.0 6.5 6.0 5.5 5.0 4.5 4.0 3.5 3.0 2.5 2.0 1.5 1.0 0.5 0.0 ppm
1.96 1.00 2.00 3.02 3.06 1.03 1.02 1.04 2.13 1.09 3.22

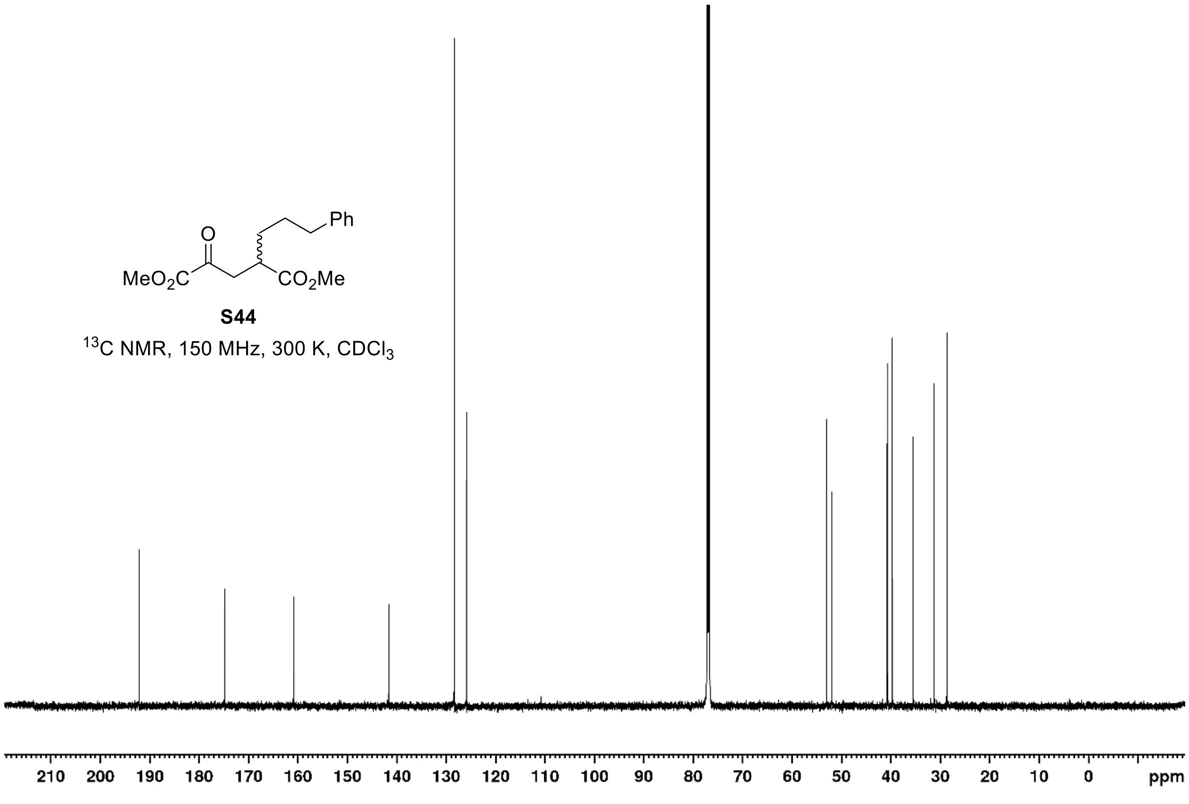

MeO2C
CO2Me
Ph
O
S44
13C NMR, 150 MHz, 300 K, CDCl3
210 200 190 180 170 160 150 140 130 120 110 100 90 80 70 60 50 40 30 20 10 0 ppm

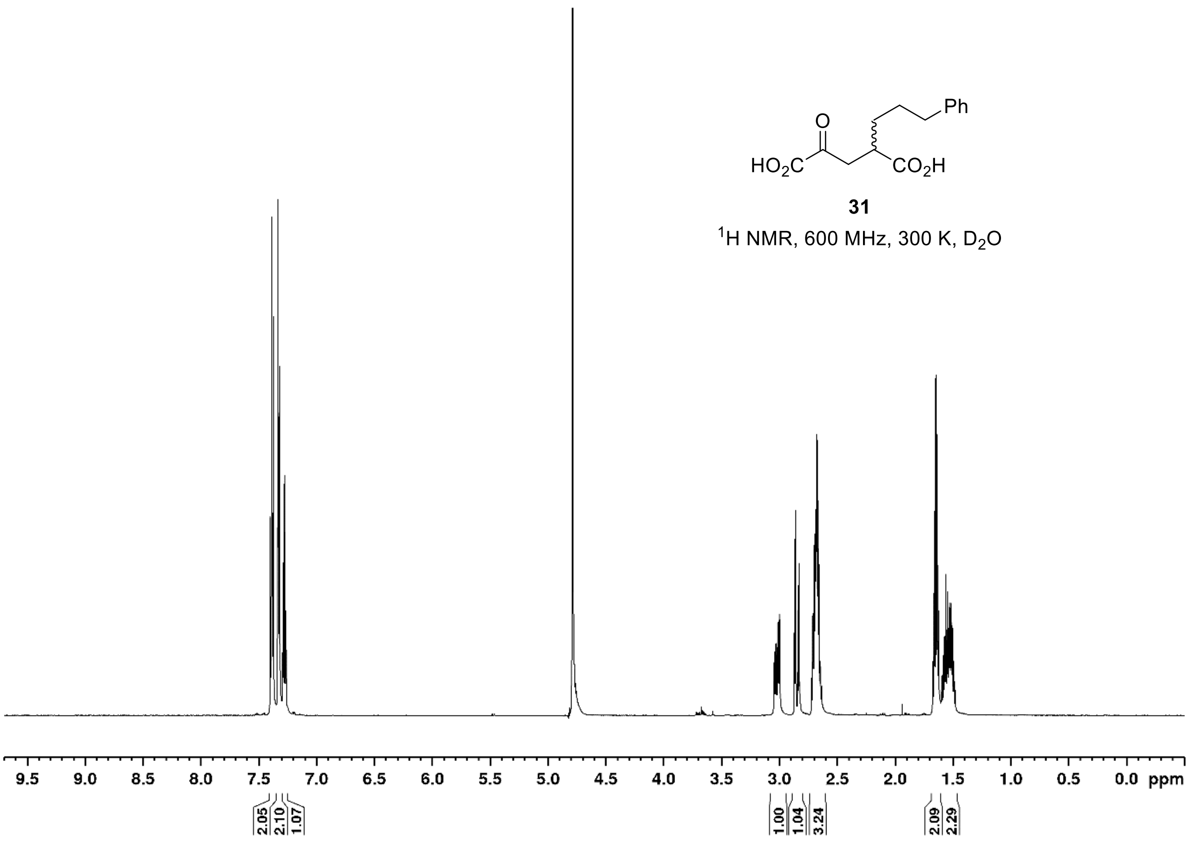

Ph
O
HO2C
CO2H
31
1H NMR, 600 MHz, 300 K, D2O
9.5 9.0 8.5 8.0 7.5 7.0 6.5 6.0 5.5 5.0 4.5 4.0 3.5 3.0 2.5 2.0 1.5 1.0 0.5 0.0 ppm
2.05 2.10 1.07
1.00 1.04 3.24
2.09 2.29

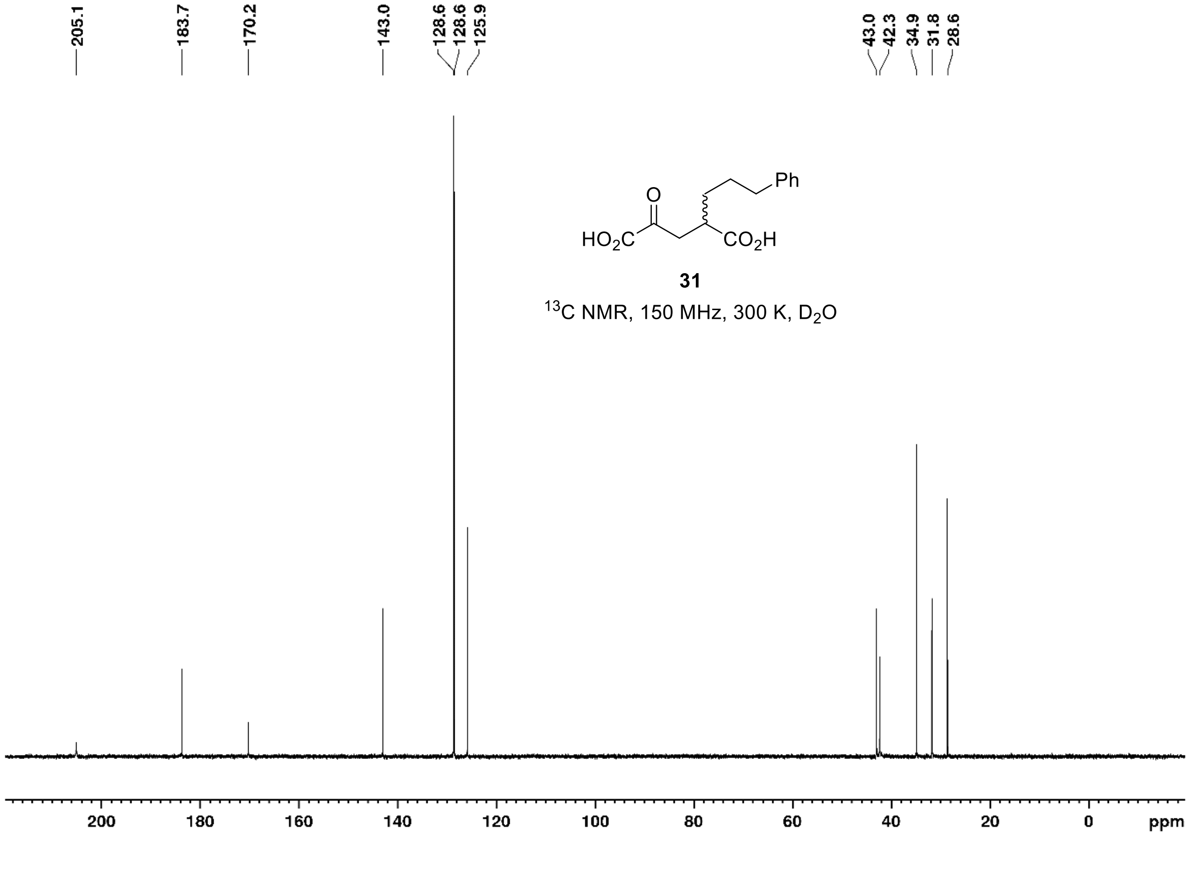

205.1
183.7
170.2
143.0
128.6 128.6 125.9
43.0 42.3 34.9 31.8 28.6
Ph
O
HO2C
CO2H
31
13C NMR, 150 MHz, 300 K, D2O
200 180 160 140 120 100 80 60 40 20 0 ppm

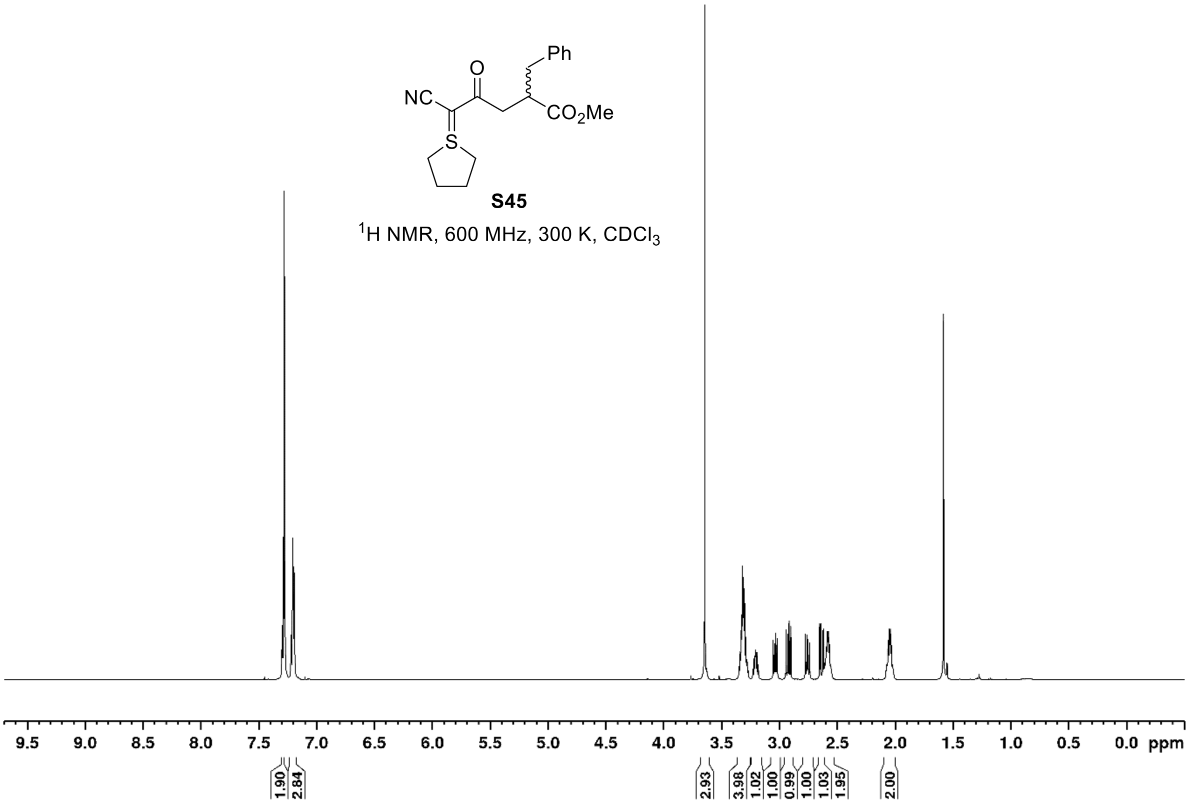
NC
O
Ph
CO2Me
S
S45
1H NMR, 600 MHz, 300 K, CDCl3
9.5 9.0 8.5 8.0 7.5 7.0 6.5 6.0 5.5 5.0 4.5 4.0 3.5 3.0 2.5 2.0 1.5 1.0 0.5 0.0 ppm
1.90 2.84 2.93 3.98 1.02 1.00 0.99 1.00 1.03 1.95 2.00

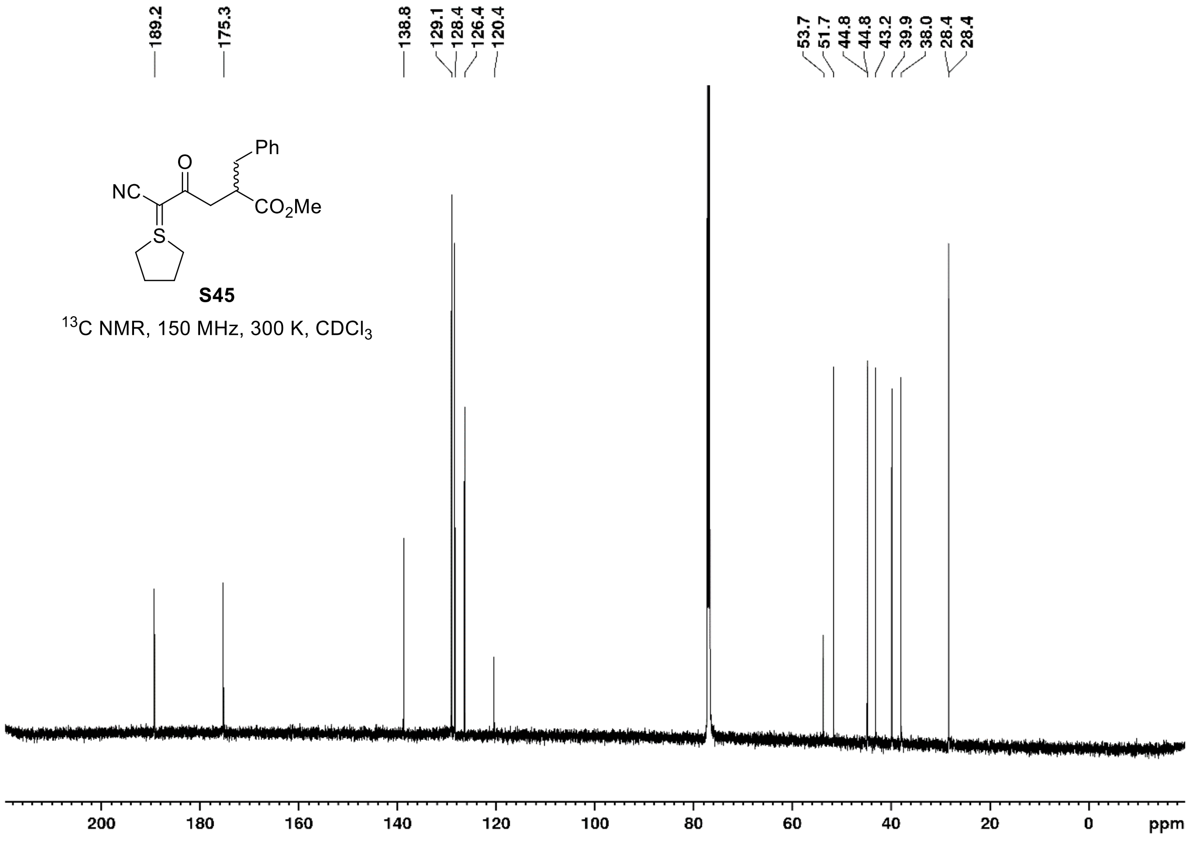
189.2
175.3
138.8
129.1
128.4
126.4
120.4
53.7
51.7
44.8
44.8
43.2
39.9
38.0
28.4
28.4
NC
O
Ph
CO2Me
S
S45
13C NMR, 150 MHz, 300 K, CDCl3
200 180 160 140 120 100 80 60 40 20 0 ppm

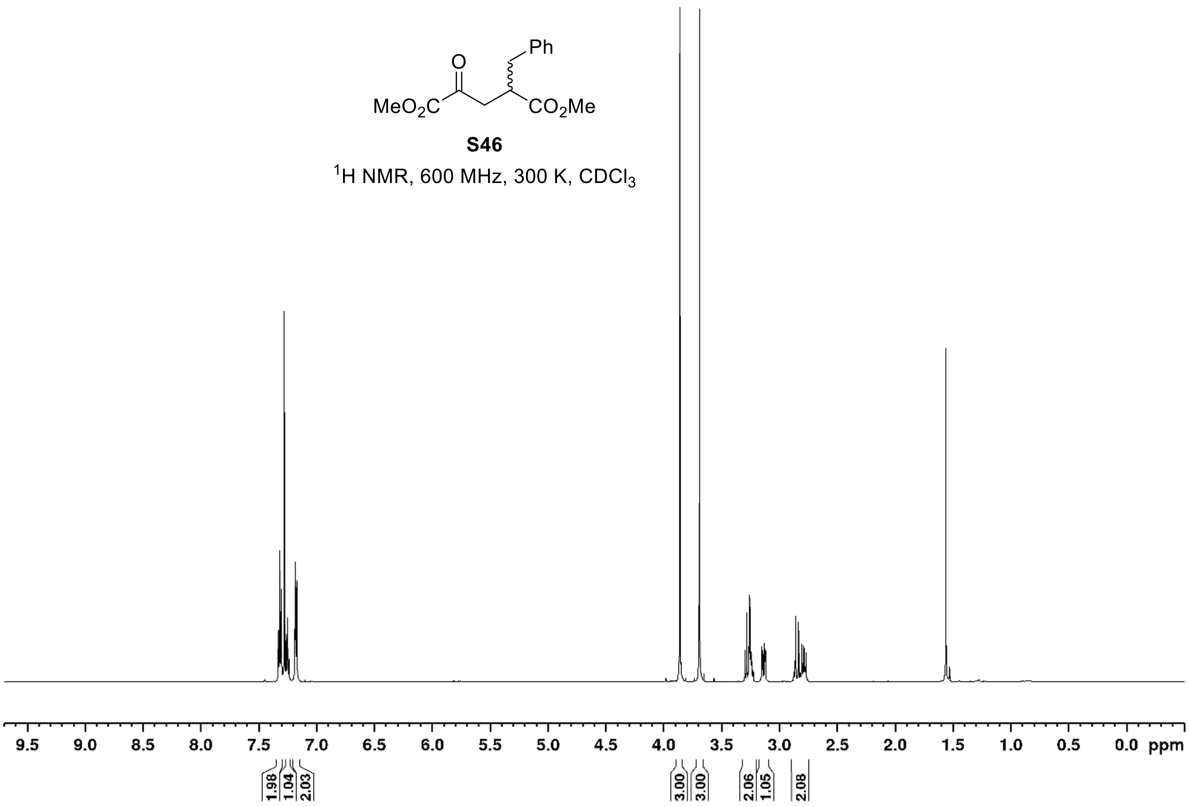

Ph
O
MeO2C
CO2Me
S46
1H NMR, 600 MHz, 300 K, CDCl3
9.5 9.0 8.5 8.0 7.5 7.0 6.5 6.0 5.5 5.0 4.5 4.0 3.5 3.0 2.5 2.0 1.5 1.0 0.5 0.0 ppm
1.98 1.04 2.03
3.00 3.00
2.06 1.05
2.08

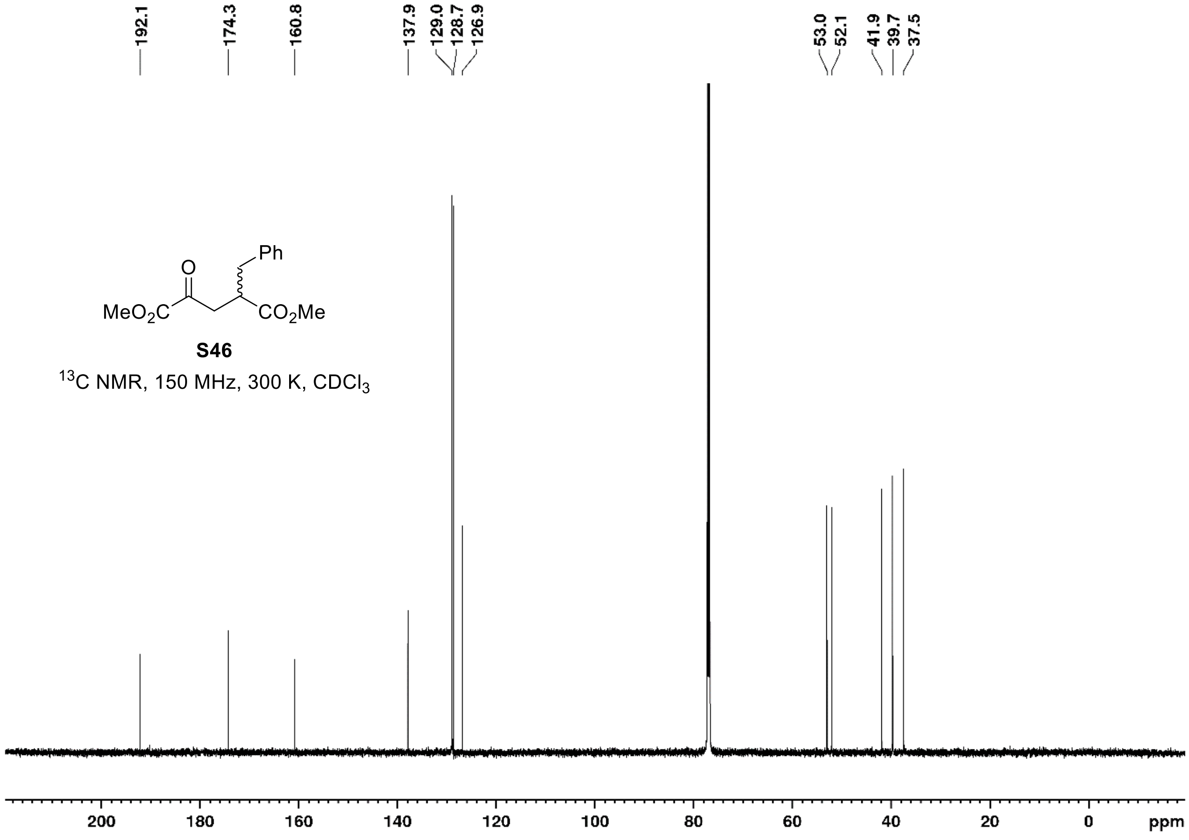

192.1
174.3
160.8
137.9 129.0 128.7 126.9
53.0 52.1
41.9 39.7 37.5
Ph
O
MeO2C
CO2Me
S46
13C NMR, 150 MHz, 300 K, CDCl3
200 180 160 140 120 100 80 60 40 20 0 ppm

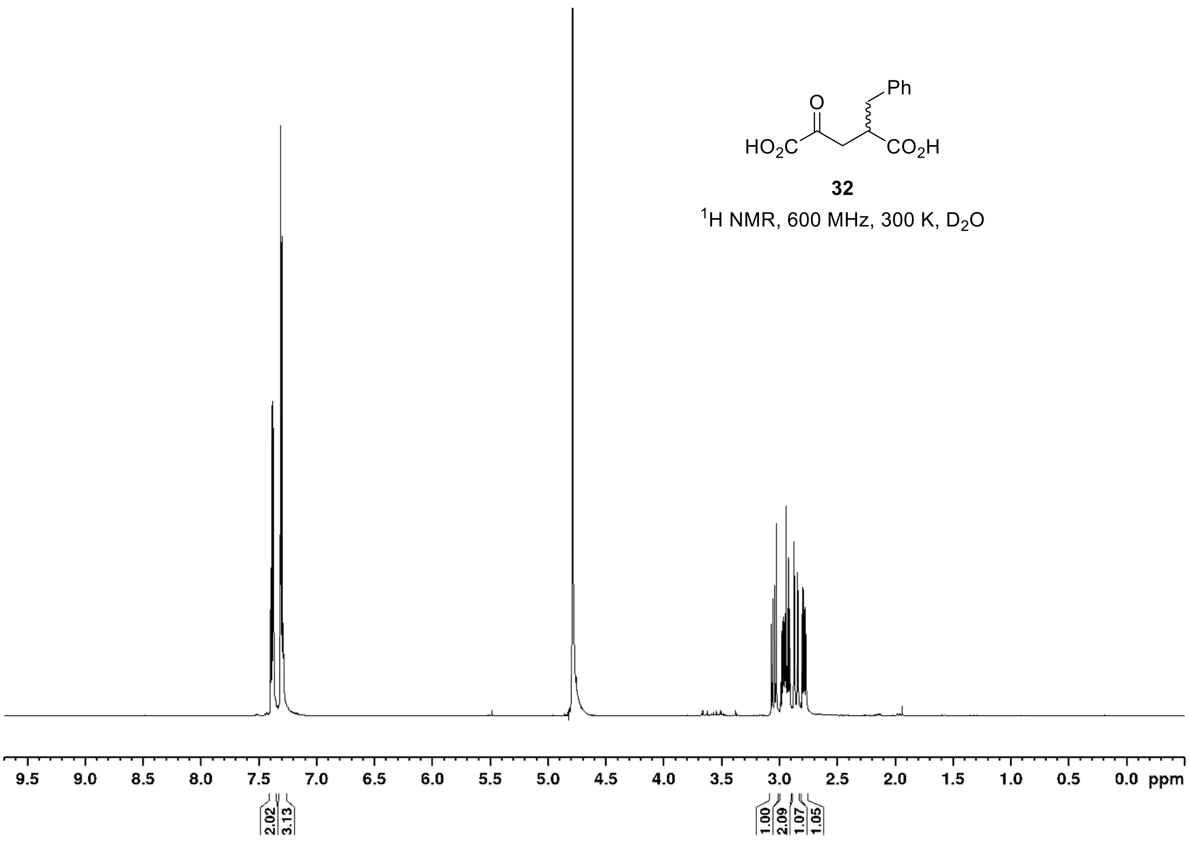
Ph
O
HO_2C
CO_2H
32
^1H NMR, 600 MHz, 300 K, D_2O
9.5 9.0 8.5 8.0 7.5 7.0 6.5 6.0 5.5 5.0 4.5 4.0 3.5 3.0 2.5 2.0 1.5 1.0 0.5 0.0 ppm
2.02 3.13
1.00 2.09 1.07 1.05

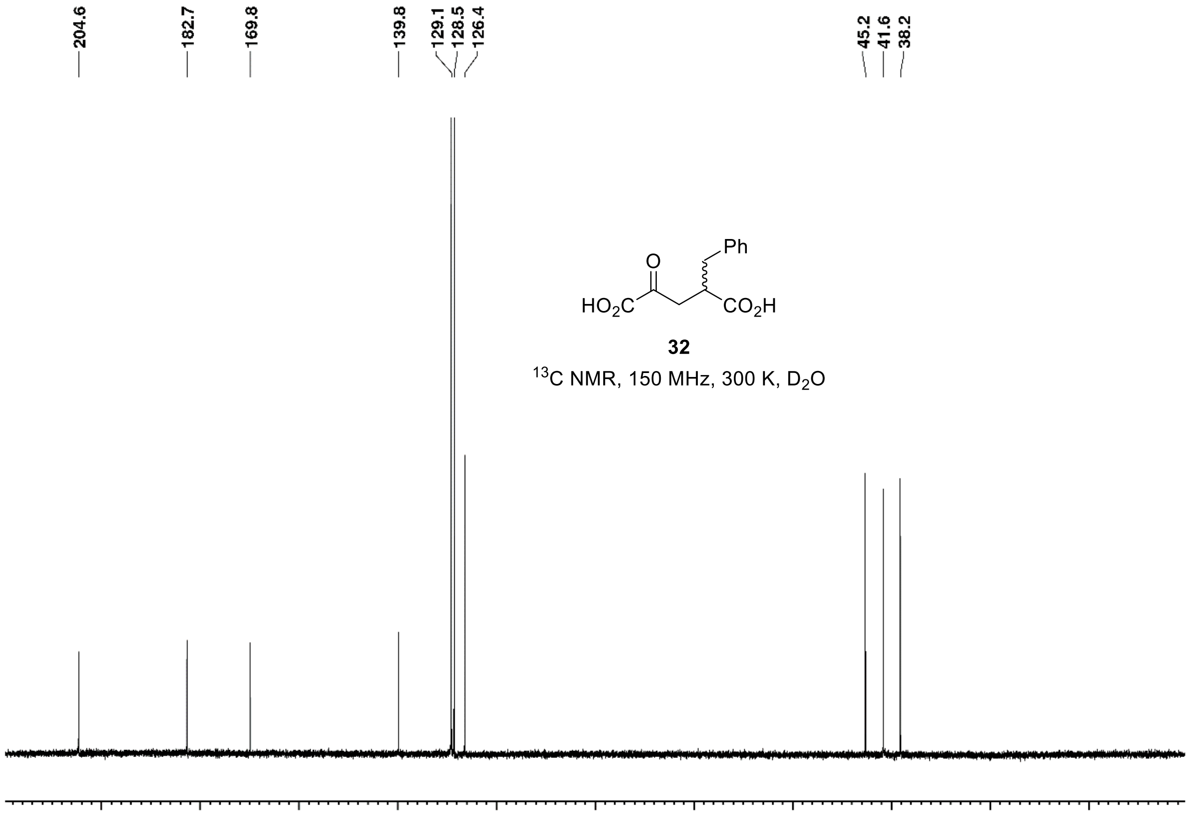
204.6
182.7
169.8
139.8
129.1
128.5
126.4
45.2
41.6
38.2
Ph
O
HO_2C
CO_2H
32
^{13}C NMR, 150 MHz, 300 K, D_2O
200 180 160 140 120 100 80 60 40 20 0 ppm

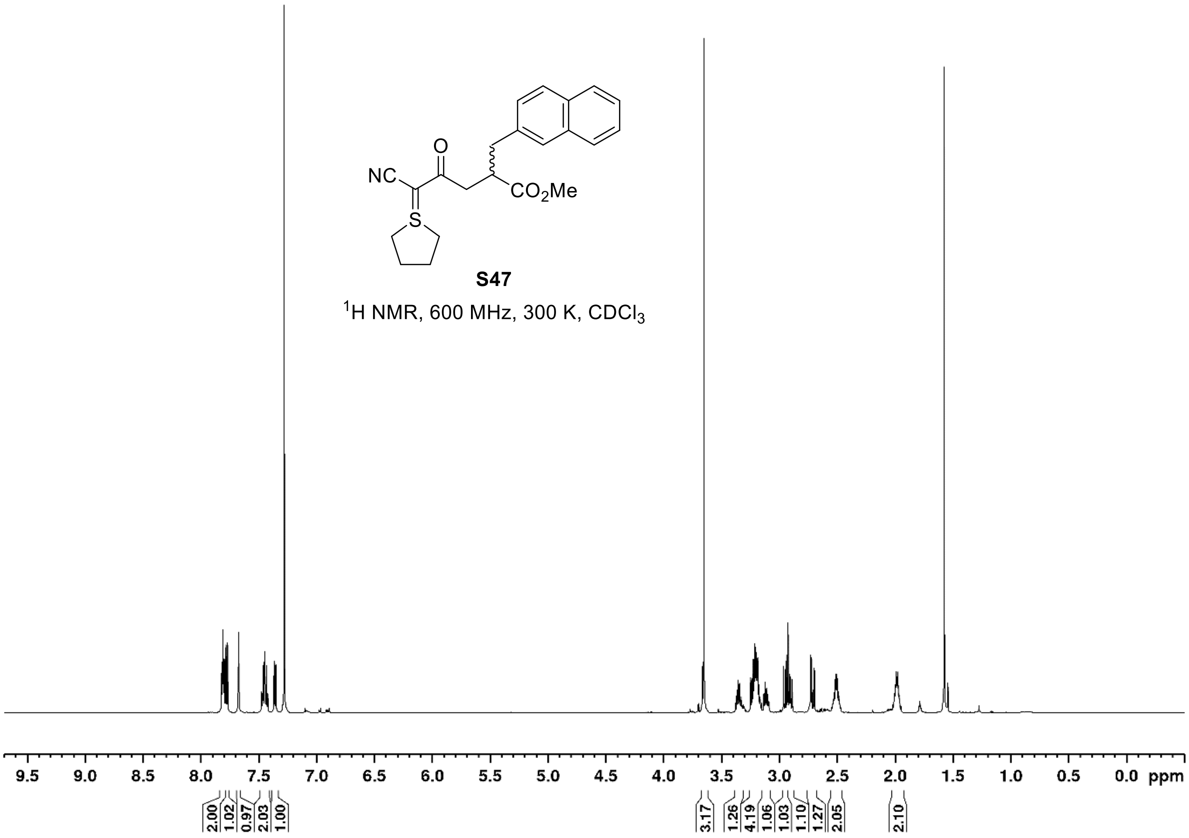
NC
O
CO2Me
S
S47
1H NMR, 600 MHz, 300 K, CDCl3
9.5 9.0 8.5 8.0 7.5 7.0 6.5 6.0 5.5 5.0 4.5 4.0 3.5 3.0 2.5 2.0 1.5 1.0 0.5 0.0 ppm
2.00 1.02 0.97 2.03 1.00
3.17 1.26 4.19 1.06 1.03 1.10 1.27 2.05 2.10

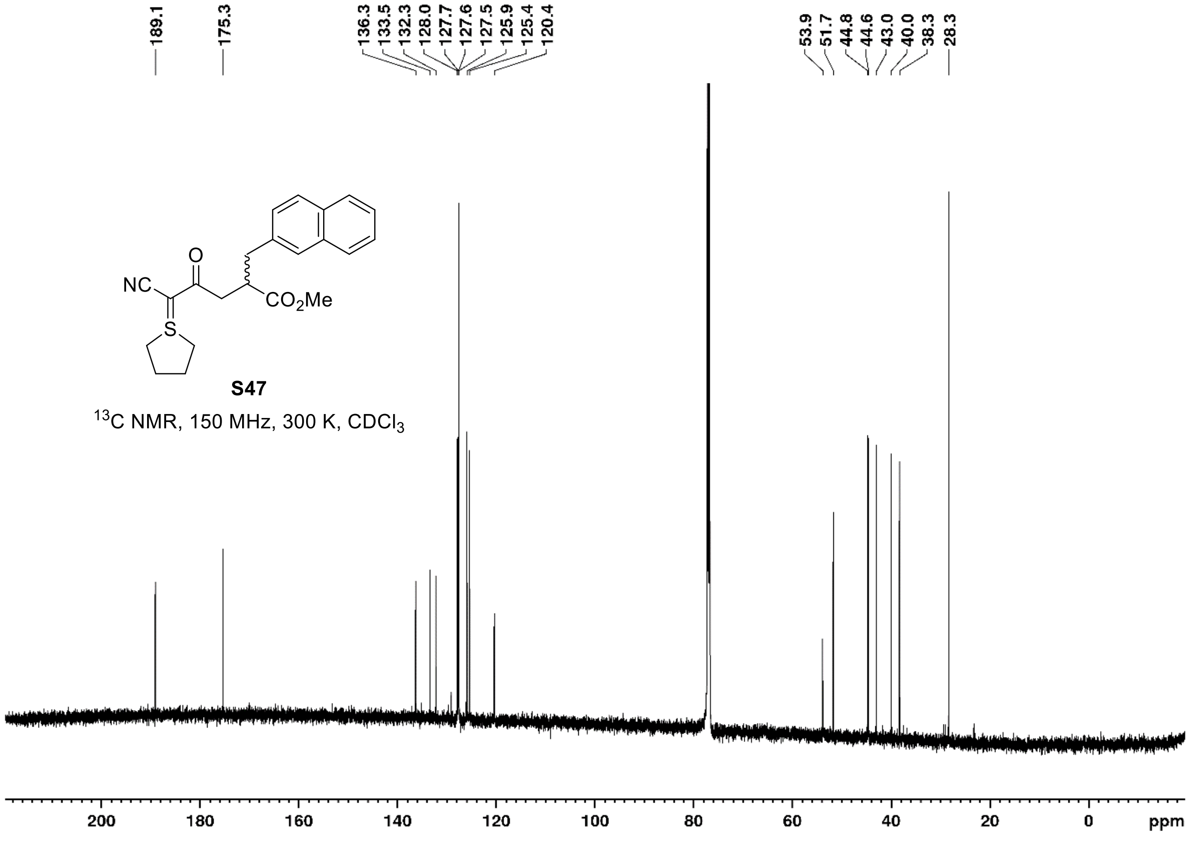
189.1
175.3
136.3 133.5 132.3 128.0 127.7 127.6 127.5 125.9 125.4 120.4
53.9 51.7 44.8 44.6 43.0 40.0 38.3 28.3
NC
O
CO2Me
S
S47
13C NMR, 150 MHz, 300 K, CDCl3
200 180 160 140 120 100 80 60 40 20 0 ppm

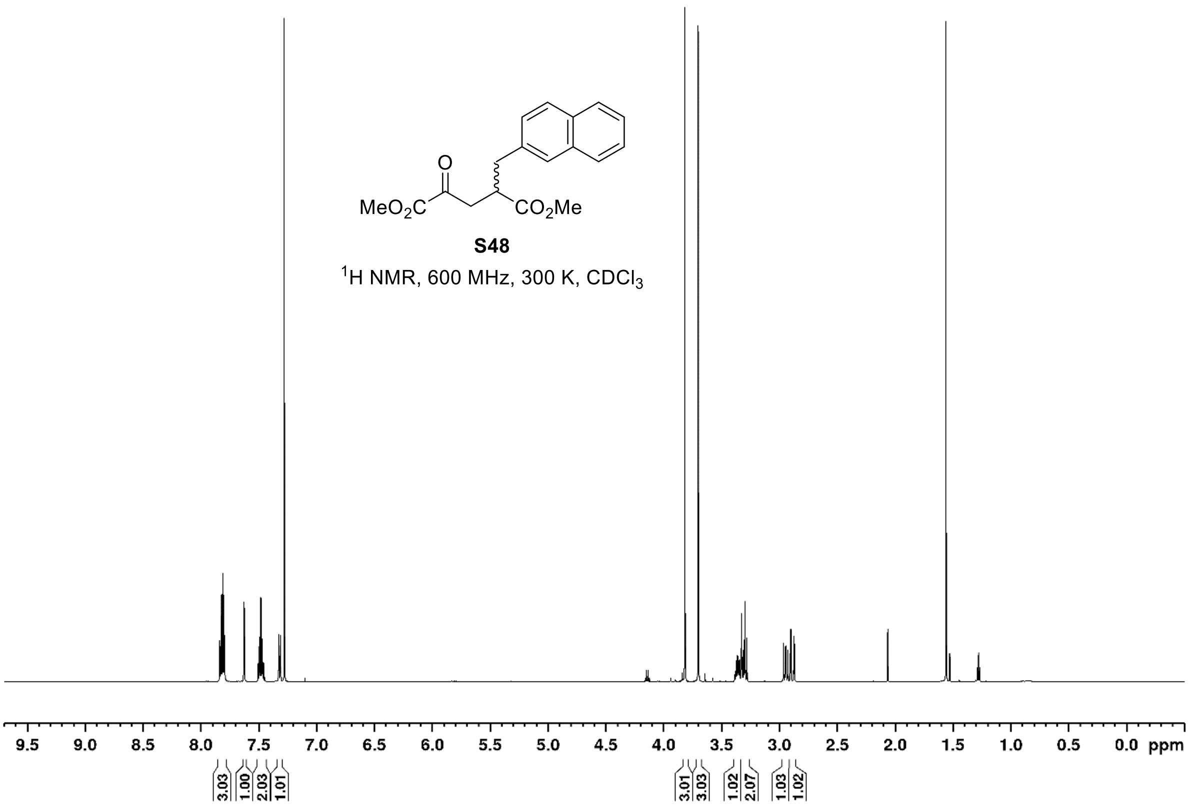
O
MeO2C
CO2Me
S48
1H NMR, 600 MHz, 300 K, CDCl3
9.5 9.0 8.5 8.0 7.5 7.0 6.5 6.0 5.5 5.0 4.5 4.0 3.5 3.0 2.5 2.0 1.5 1.0 0.5 0.0 ppm
3.03 1.00 2.03 1.01 3.01 3.03 1.02 2.07 1.03 1.02

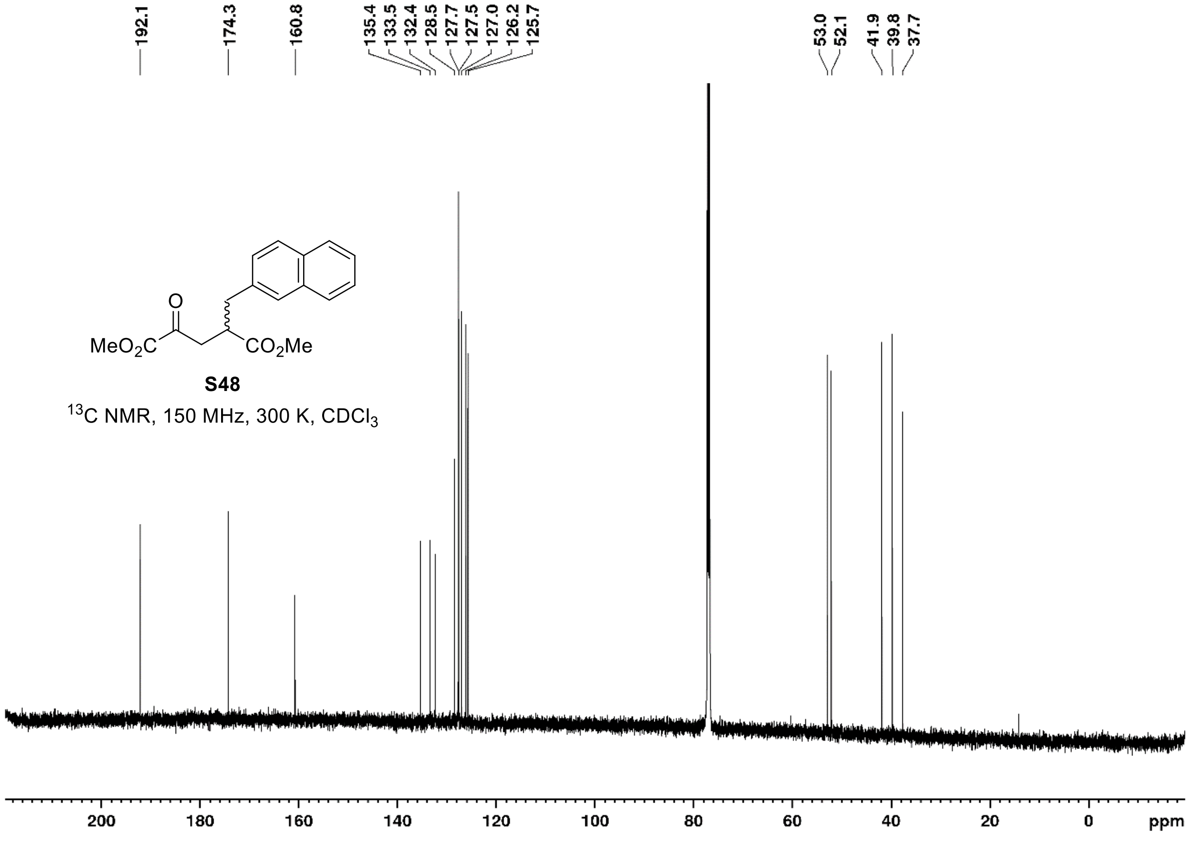
192.1
174.3
160.8
135.4 133.5 132.4 128.5 127.7 127.5 127.0 126.2 125.7
53.0 52.1
41.9 39.8 37.7
O
MeO2C
CO2Me
S48
13C NMR, 150 MHz, 300 K, CDCl3
200 180 160 140 120 100 80 60 40 20 0 ppm

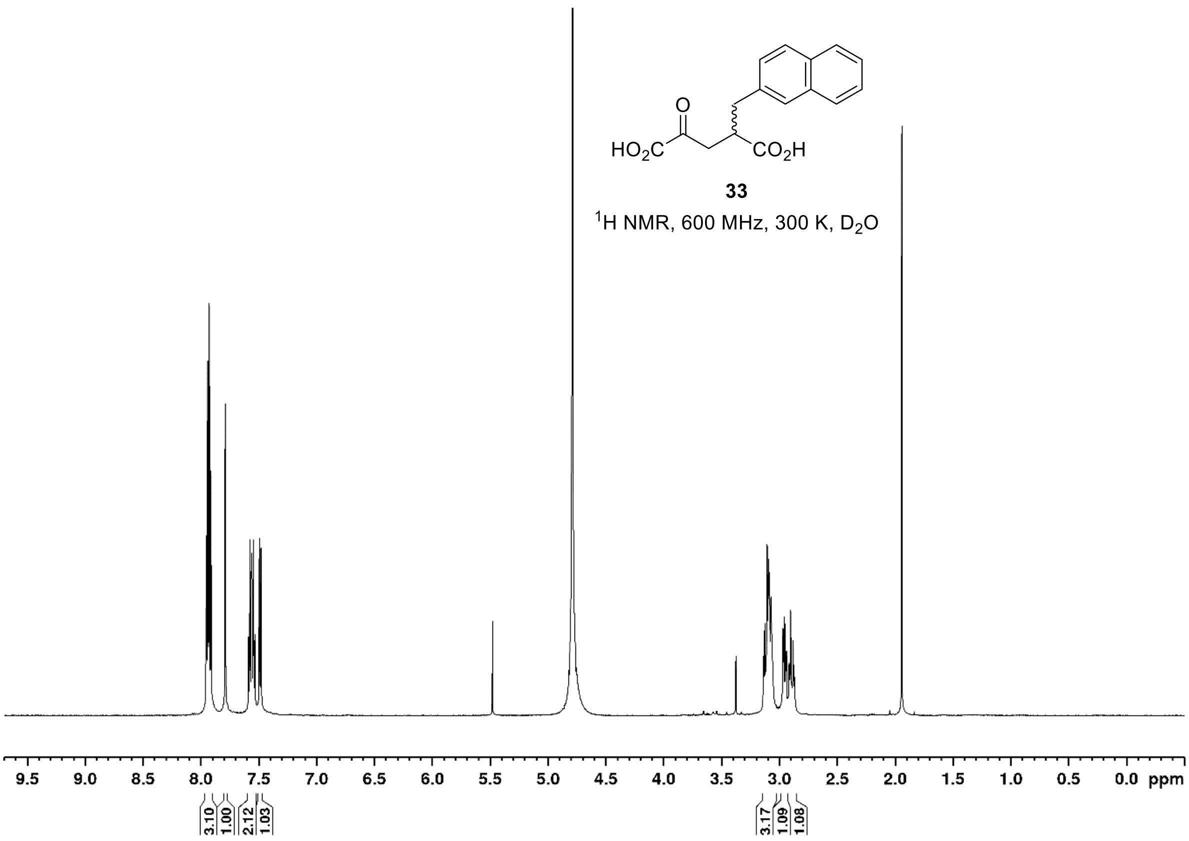

O
HO2C
CO2H
33
1H NMR, 600 MHz, 300 K, D2O
9.5 9.0 8.5 8.0 7.5 7.0 6.5 6.0 5.5 5.0 4.5 4.0 3.5 3.0 2.5 2.0 1.5 1.0 0.5 0.0 ppm
3.10 1.00 2.12 1.03
3.17 1.09 1.08

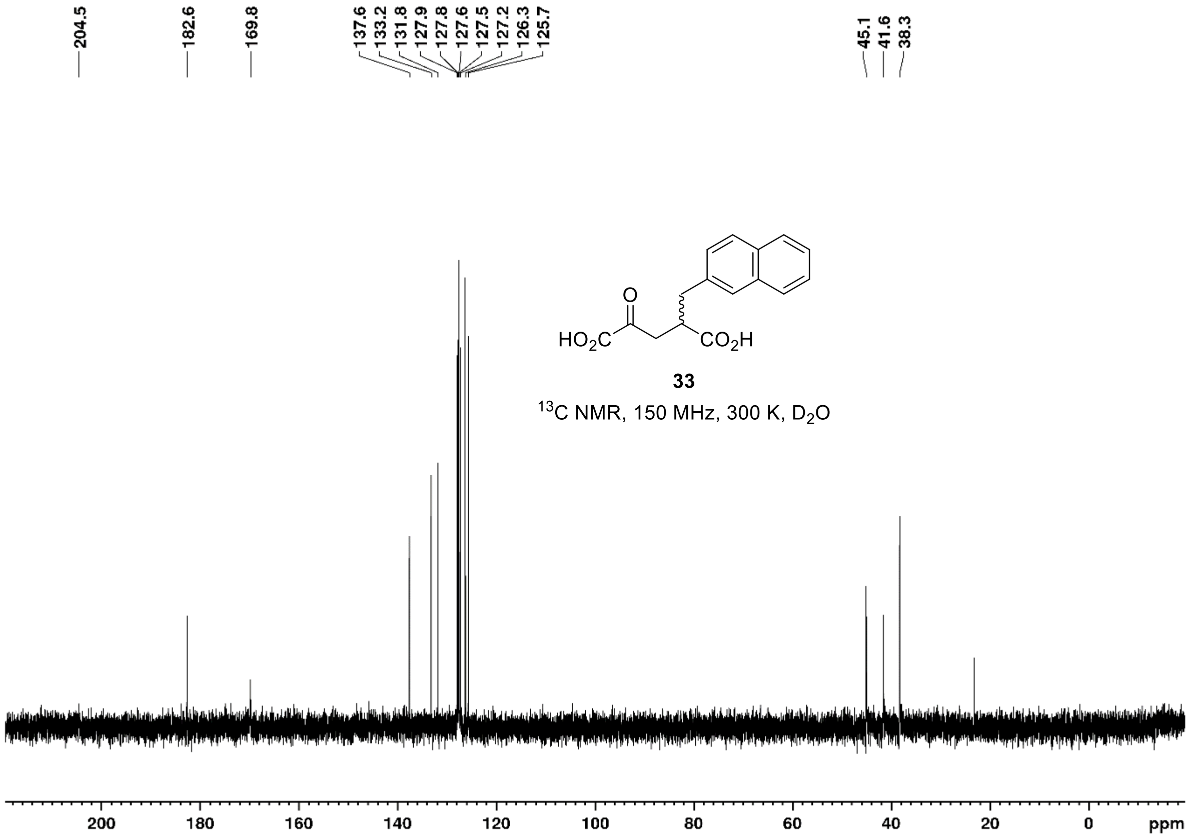

204.5
182.6
169.8
137.6 133.2 131.8 127.9 127.8 127.6 127.5 127.2 126.3 125.7
45.1 41.6 38.3
O
HO2C
CO2H
33
13C NMR, 150 MHz, 300 K, D2O
200 180 160 140 120 100 80 60 40 20 0 ppm

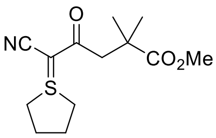

S49

^{1}H NMR, 600 MHz, 300 K, $CDCl_3$

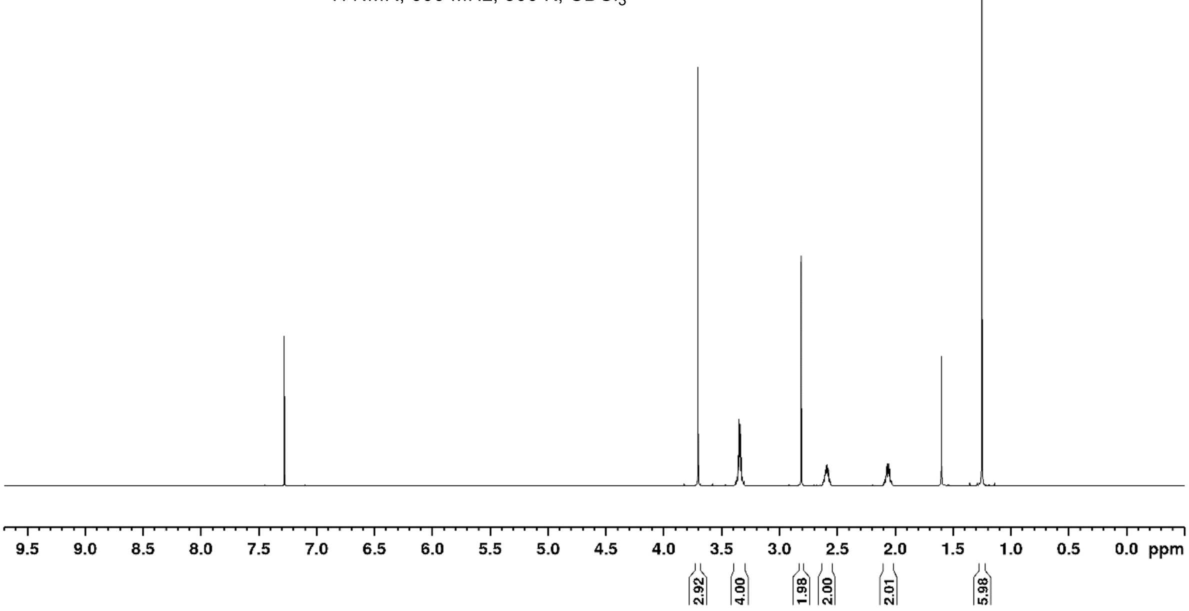

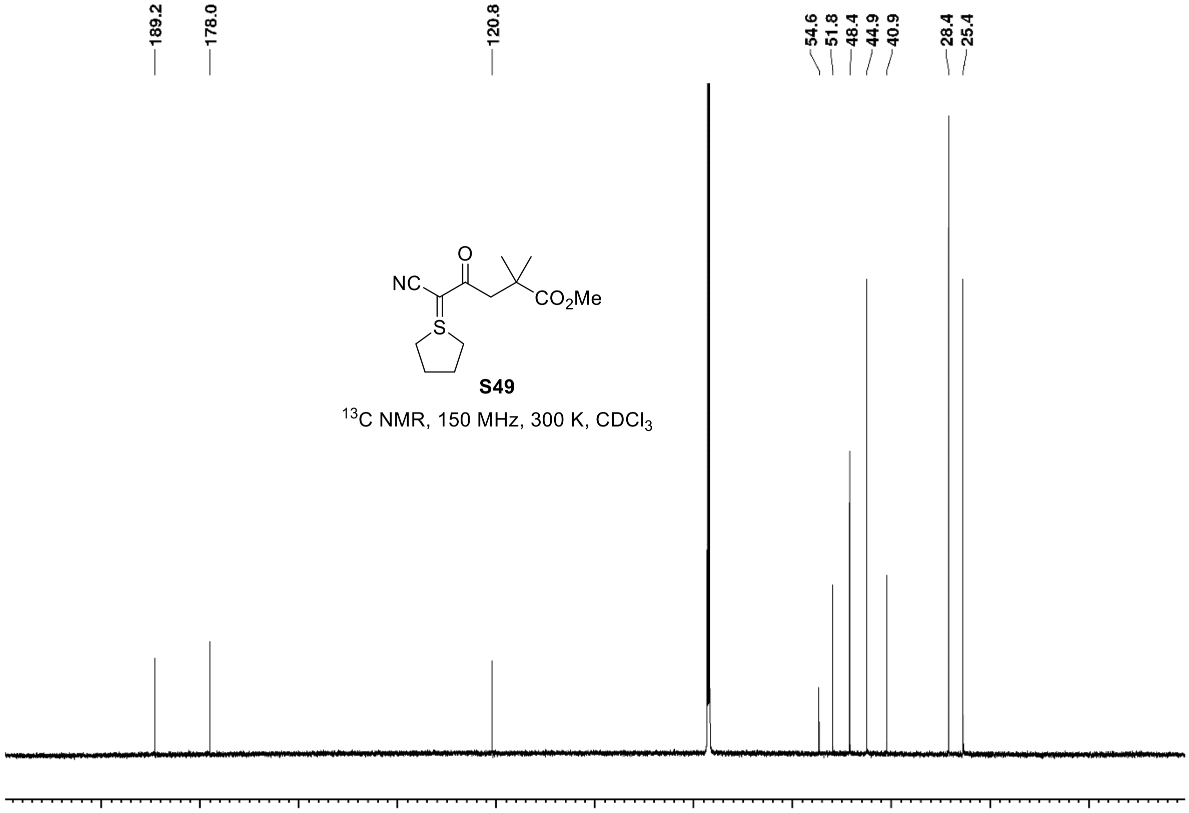

S49

^{13}C NMR, 150 MHz, 300 K, $CDCl_3$

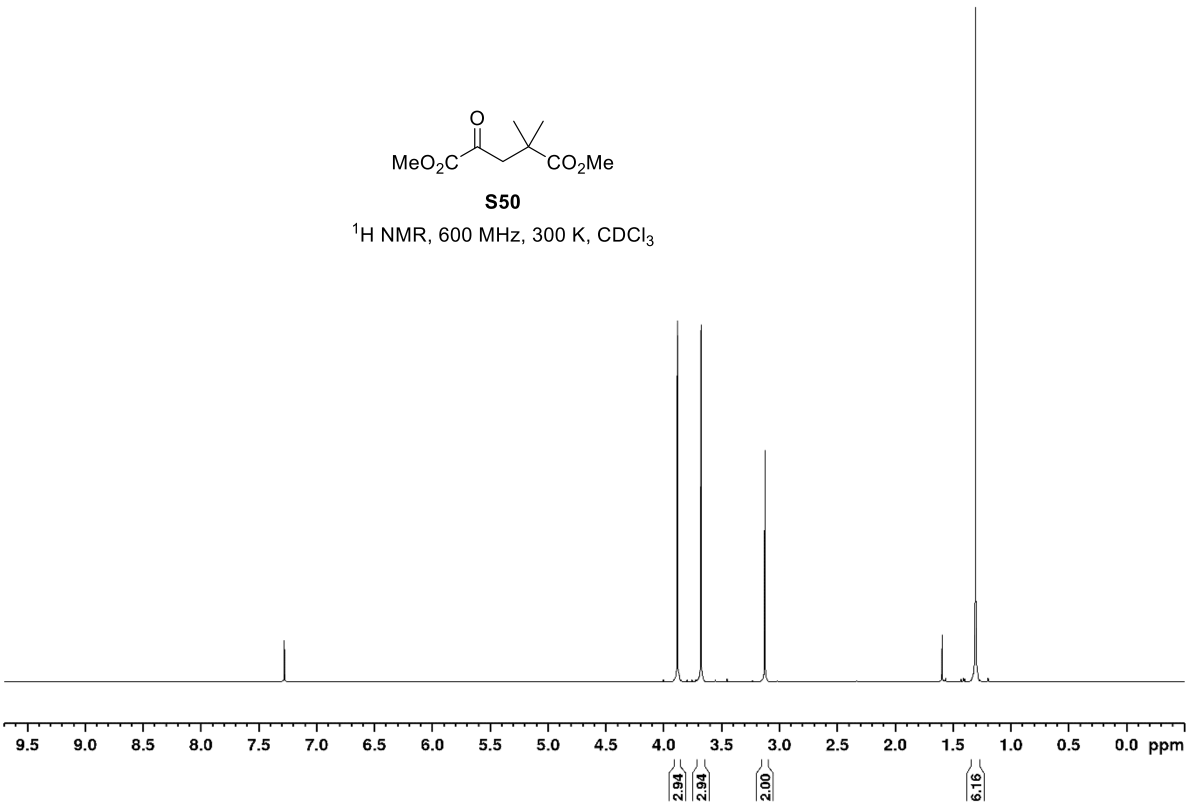
MeO2C CO2Me
S50
1H NMR, 600 MHz, 300 K, CDCl3
9.5 9.0 8.5 8.0 7.5 7.0 6.5 6.0 5.5 5.0 4.5 4.0 3.5 3.0 2.5 2.0 1.5 1.0 0.5 0.0 ppm
2.94 2.94 2.00 6.16

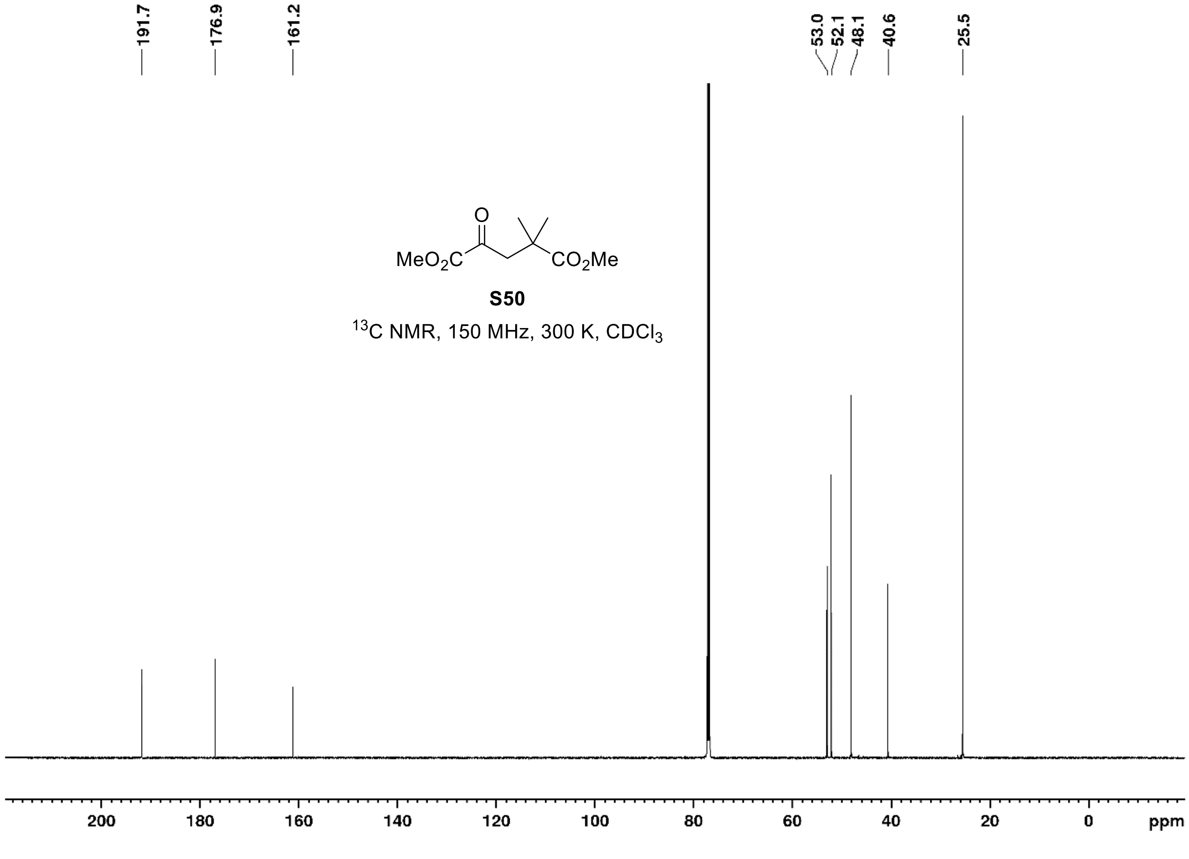
191.7 176.9 161.2 53.0 52.1 48.1 40.6 25.5
MeO2C CO2Me
S50
13C NMR, 150 MHz, 300 K, CDCl3
200 180 160 140 120 100 80 60 40 20 0 ppm

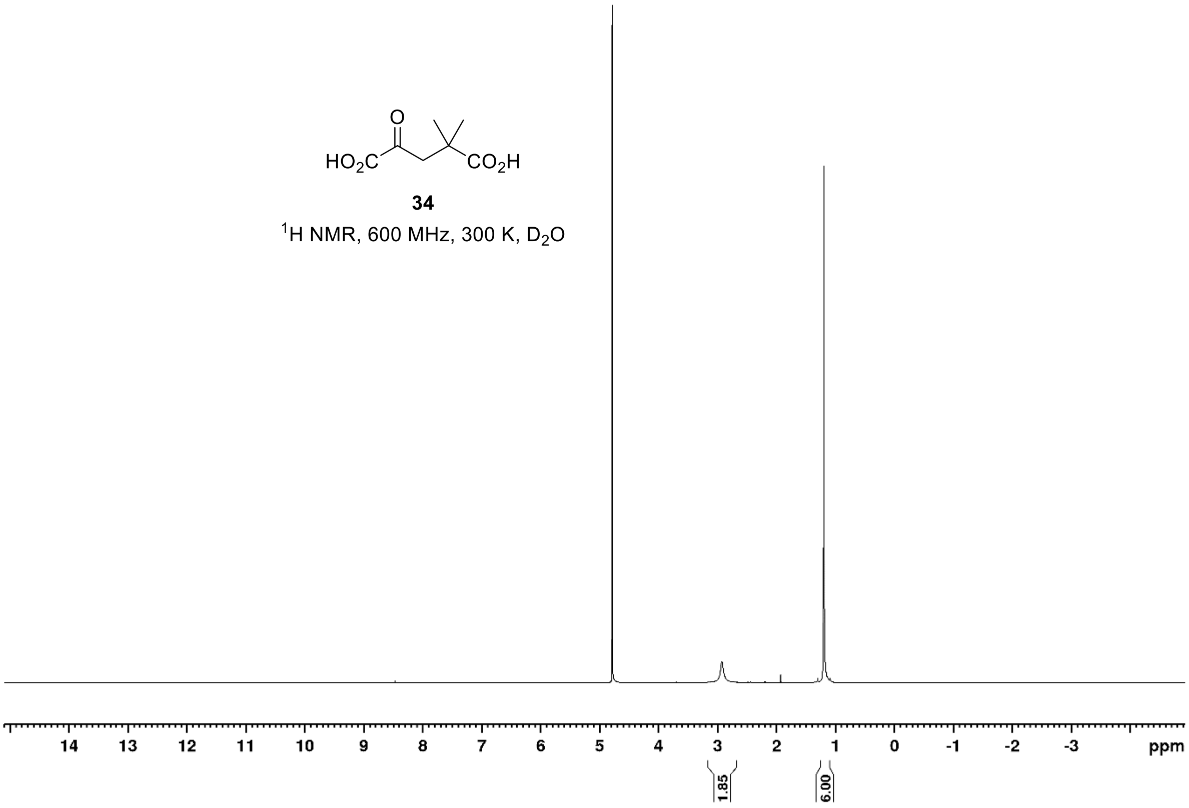

HO2C CO2H
34
1H NMR, 600 MHz, 300 K, D2O
14 13 12 11 10 9 8 7 6 5 4 3 2 1 0 -1 -2 -3 ppm
1.85
6.00

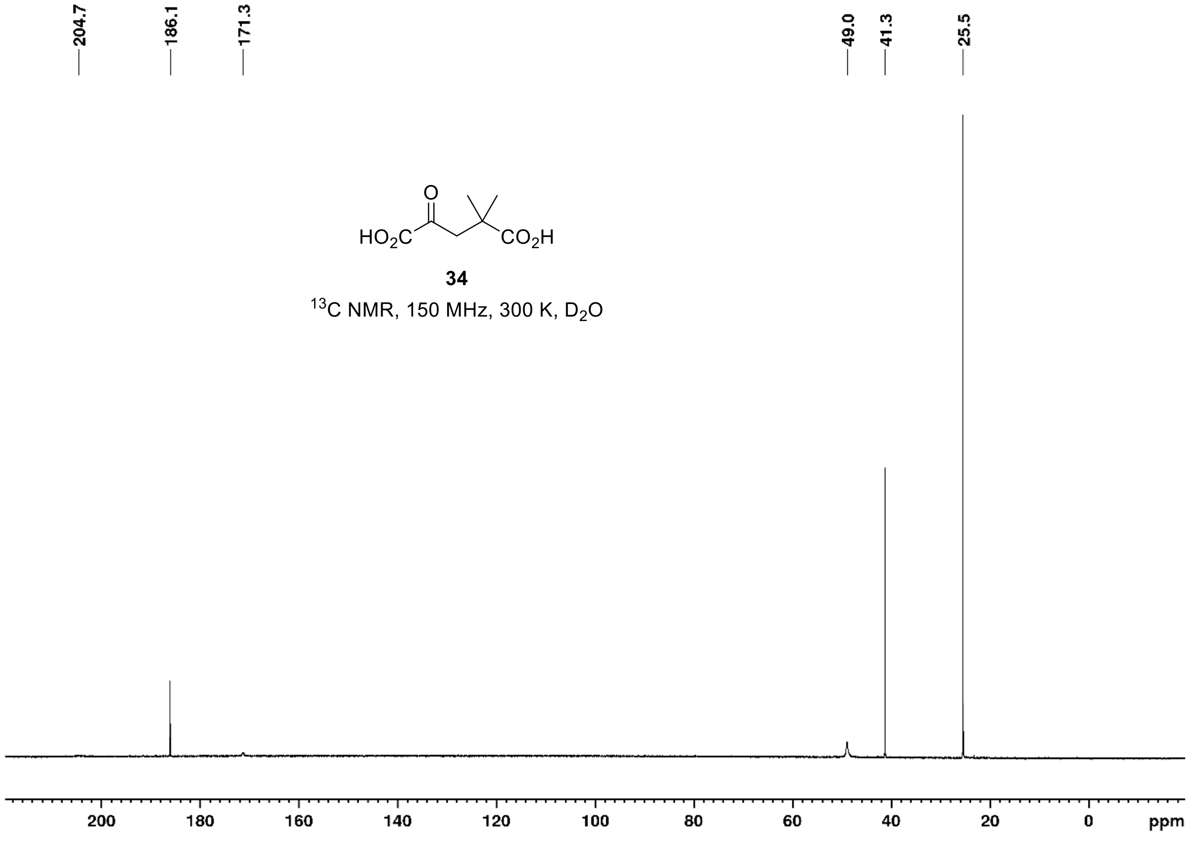

204.7
186.1
171.3
49.0
41.3
25.5
HO2C CO2H
34
13C NMR, 150 MHz, 300 K, D2O
200 180 160 140 120 100 80 60 40 20 0 ppm

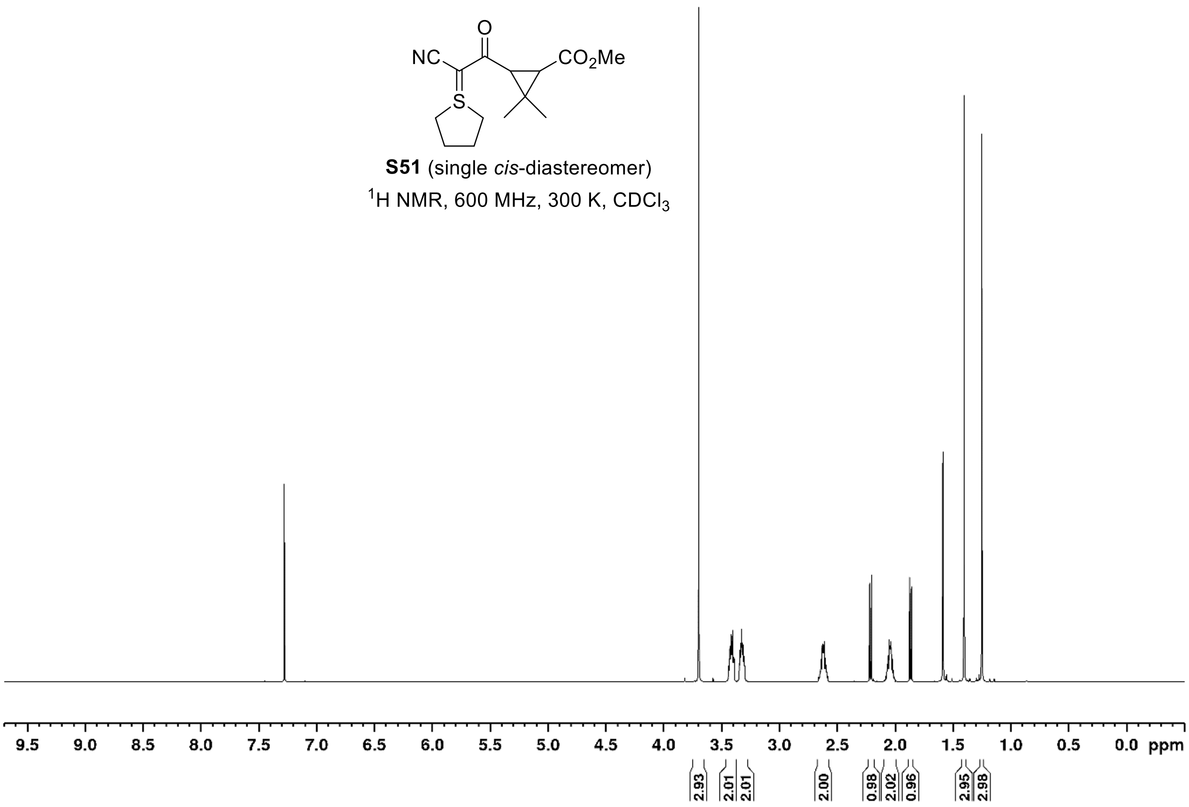
NC
O
CO2Me
S
S51 (single cis-diastereomer)
1H NMR, 600 MHz, 300 K, CDCl3
9.5 9.0 8.5 8.0 7.5 7.0 6.5 6.0 5.5 5.0 4.5 4.0 3.5 3.0 2.5 2.0 1.5 1.0 0.5 0.0 ppm
2.93 2.01 2.01 2.00 0.98 2.02 0.96 2.95 2.98

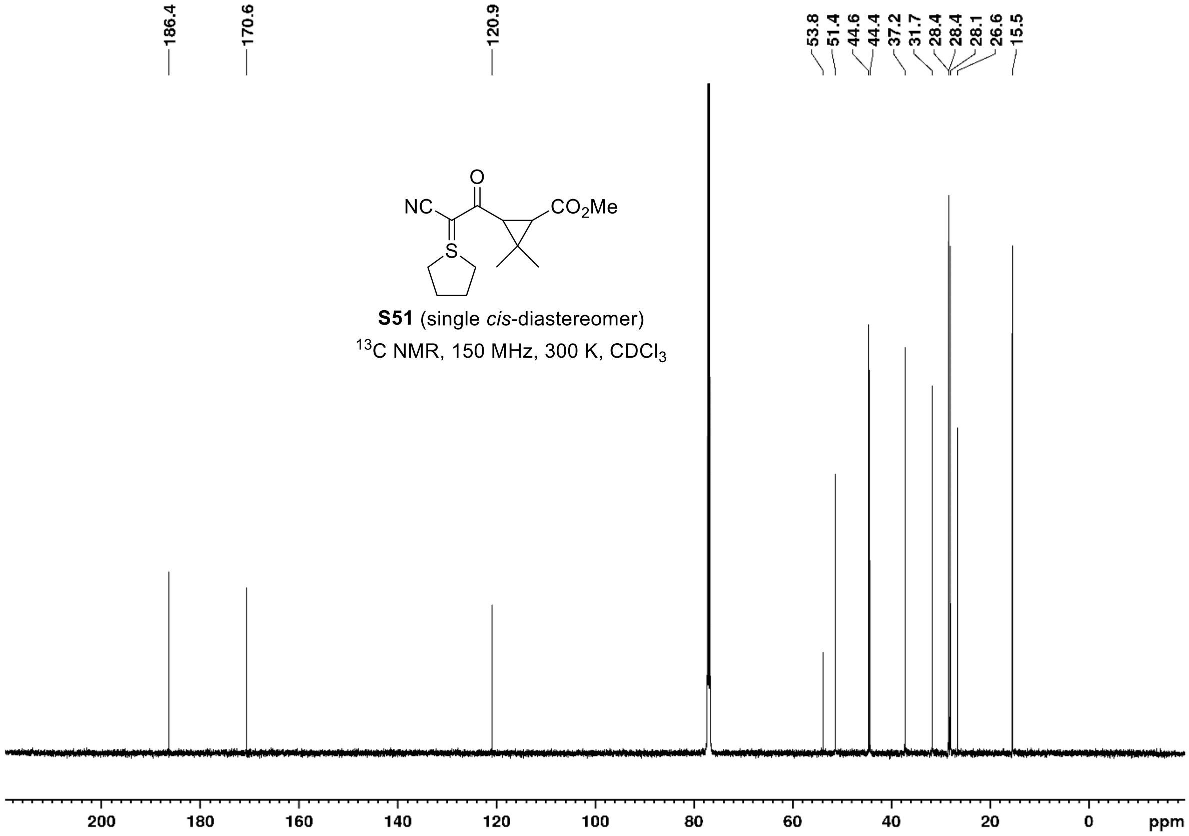
186.4
170.6
120.9
53.8 51.4 44.6 44.4 37.2 31.7 28.4 28.4 28.1 26.6 15.5
NC
O
CO2Me
S
S51 (single cis-diastereomer)
13C NMR, 150 MHz, 300 K, CDCl3
200 180 160 140 120 100 80 60 40 20 0 ppm

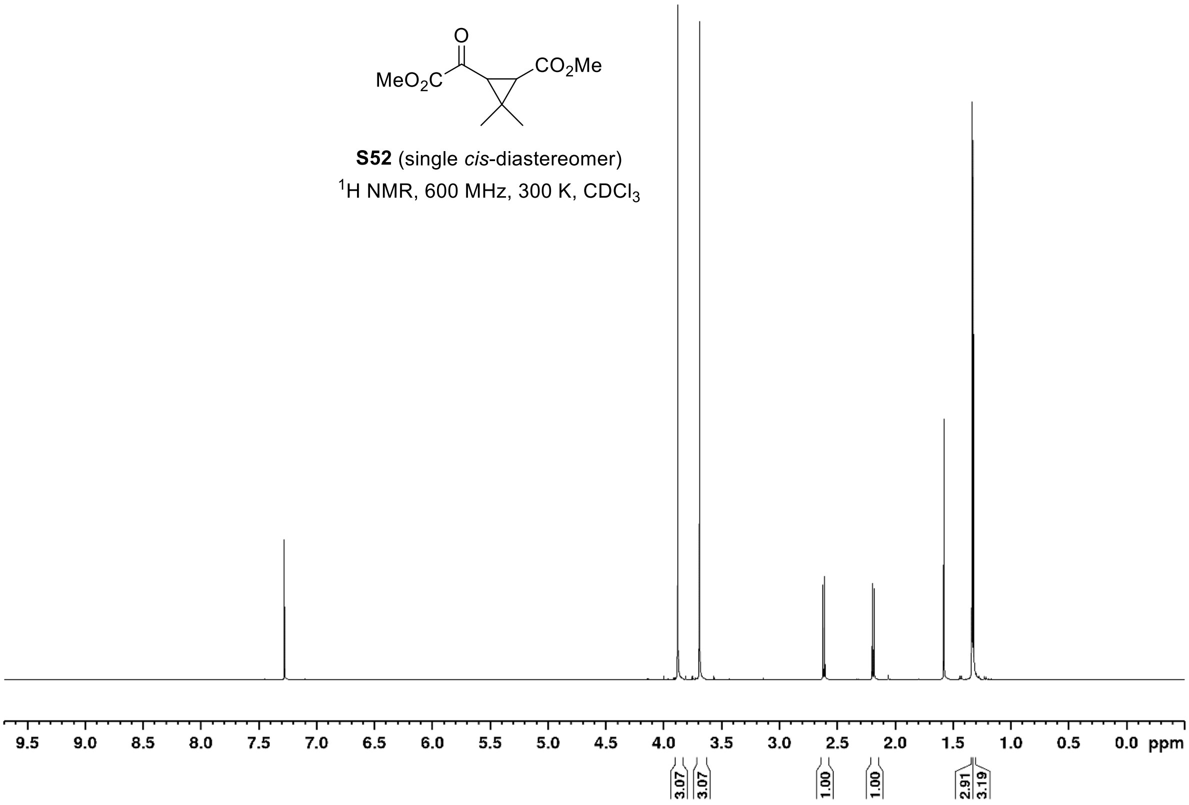
MeO2C
CO2Me
O
S52 (single cis-diastereomer)
1H NMR, 600 MHz, 300 K, CDCl3
9.5 9.0 8.5 8.0 7.5 7.0 6.5 6.0 5.5 5.0 4.5 4.0 3.5 3.0 2.5 2.0 1.5 1.0 0.5 0.0 ppm
3.07
3.07
1.00
1.00
2.91
3.19

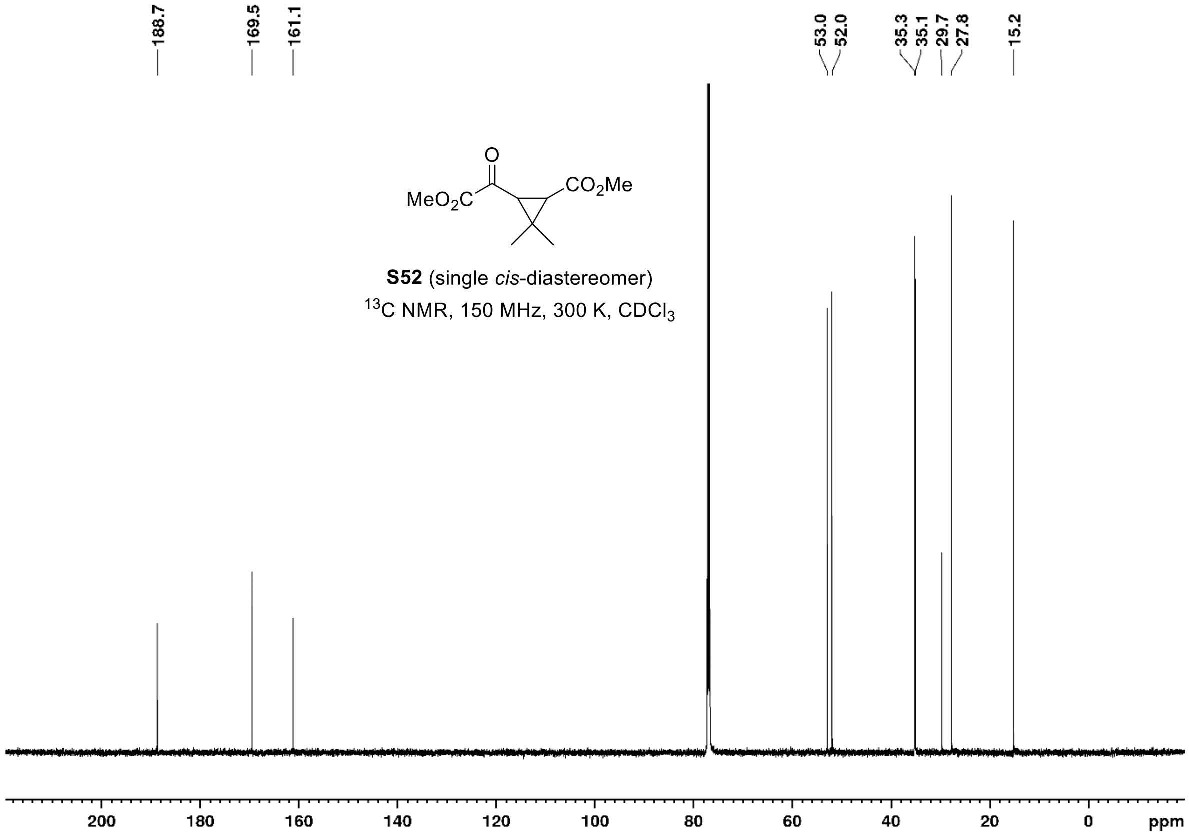
188.7
169.5
161.1
53.0
52.0
35.3
35.1
29.7
27.8
15.2
MeO2C
CO2Me
O
S52 (single cis-diastereomer)
13C NMR, 150 MHz, 300 K, CDCl3
200 180 160 140 120 100 80 60 40 20 0 ppm

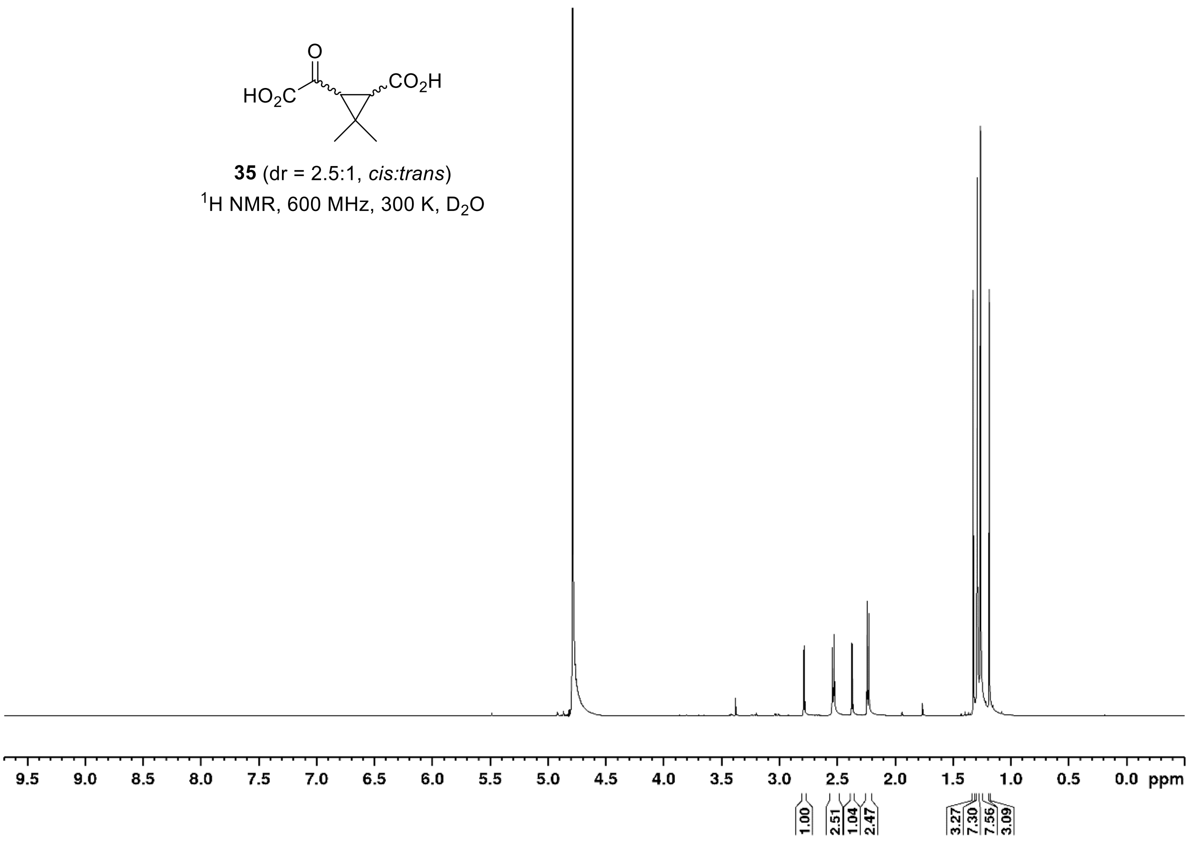

35 (dr = 2.5:1, *cis:trans*)
^{1}H NMR, 600 MHz, 300 K, D_2O

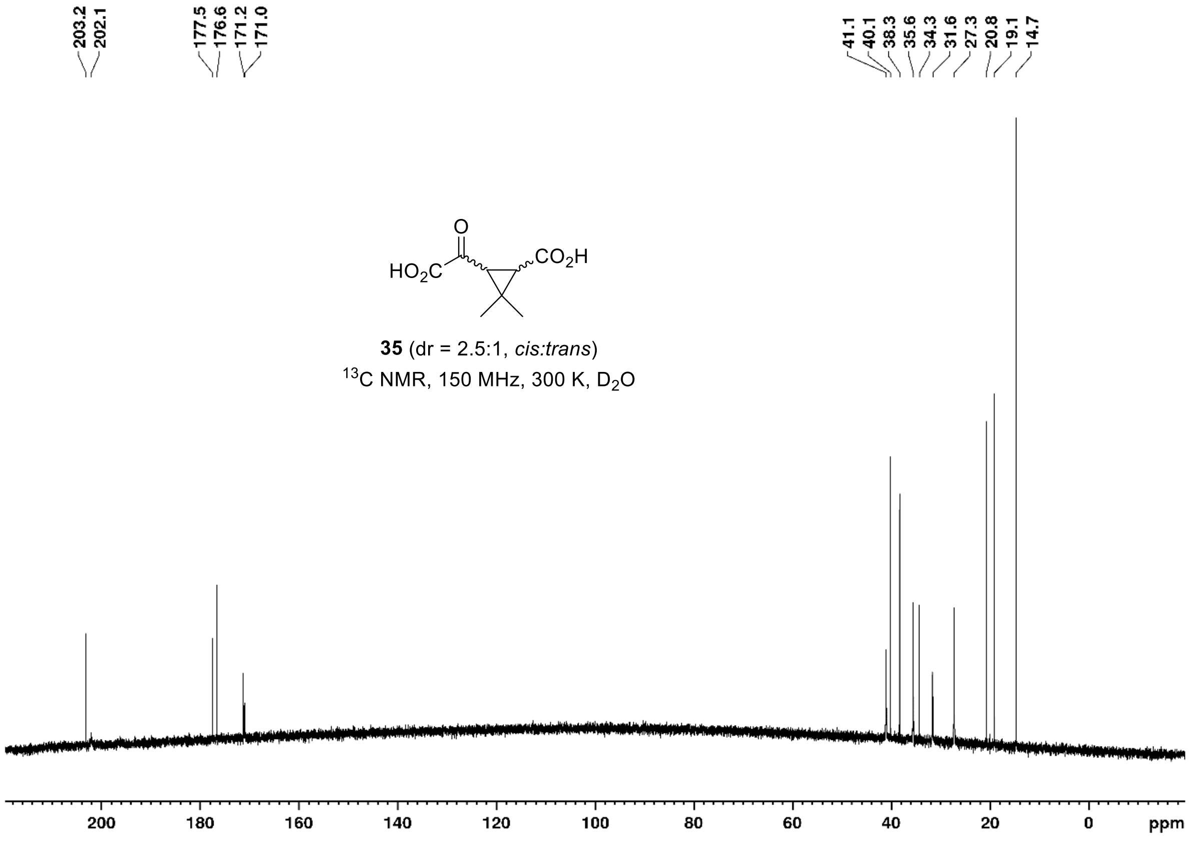

35 (dr = 2.5:1, *cis:trans*)
^{13}C NMR, 150 MHz, 300 K, D_2O

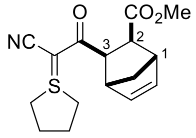

S53 (single (±)-(*2-exo,3-exo*)-diastereomer)
^{1}H NMR, 600 MHz, 300 K, $CDCl_3$

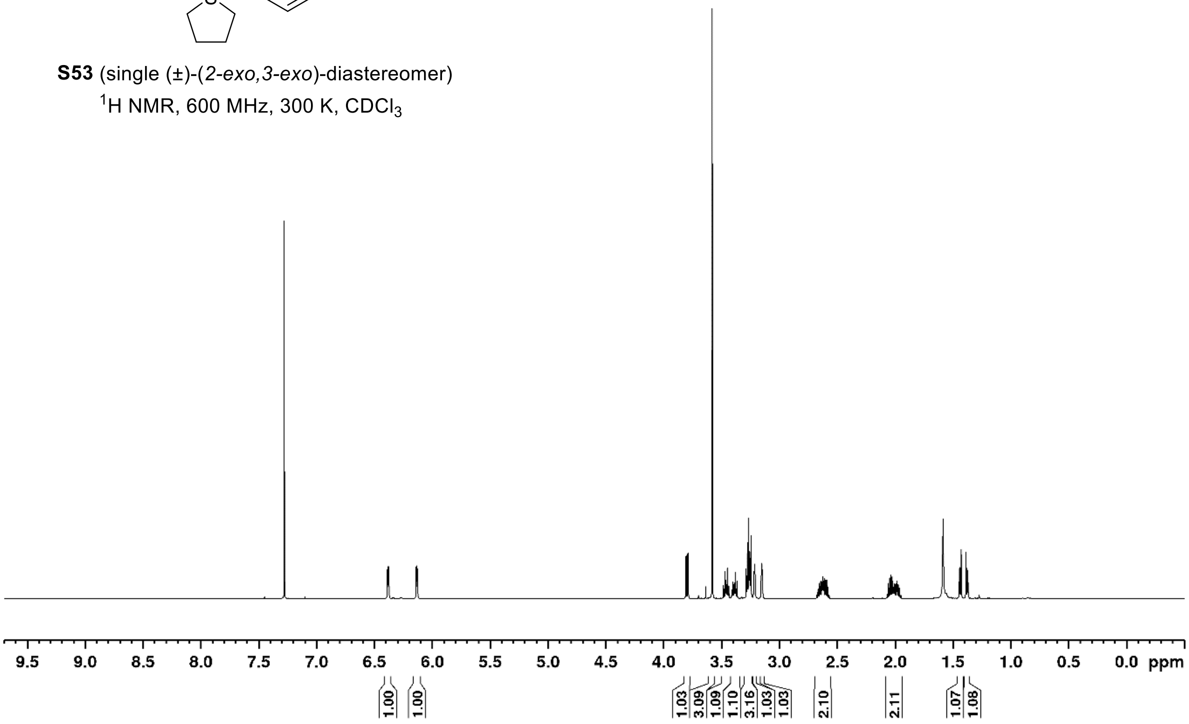

S53 (single (±)-(*2-exo,3-exo*)-diastereomer)
^{13}C NMR, 150 MHz, 300 K, $CDCl_3$

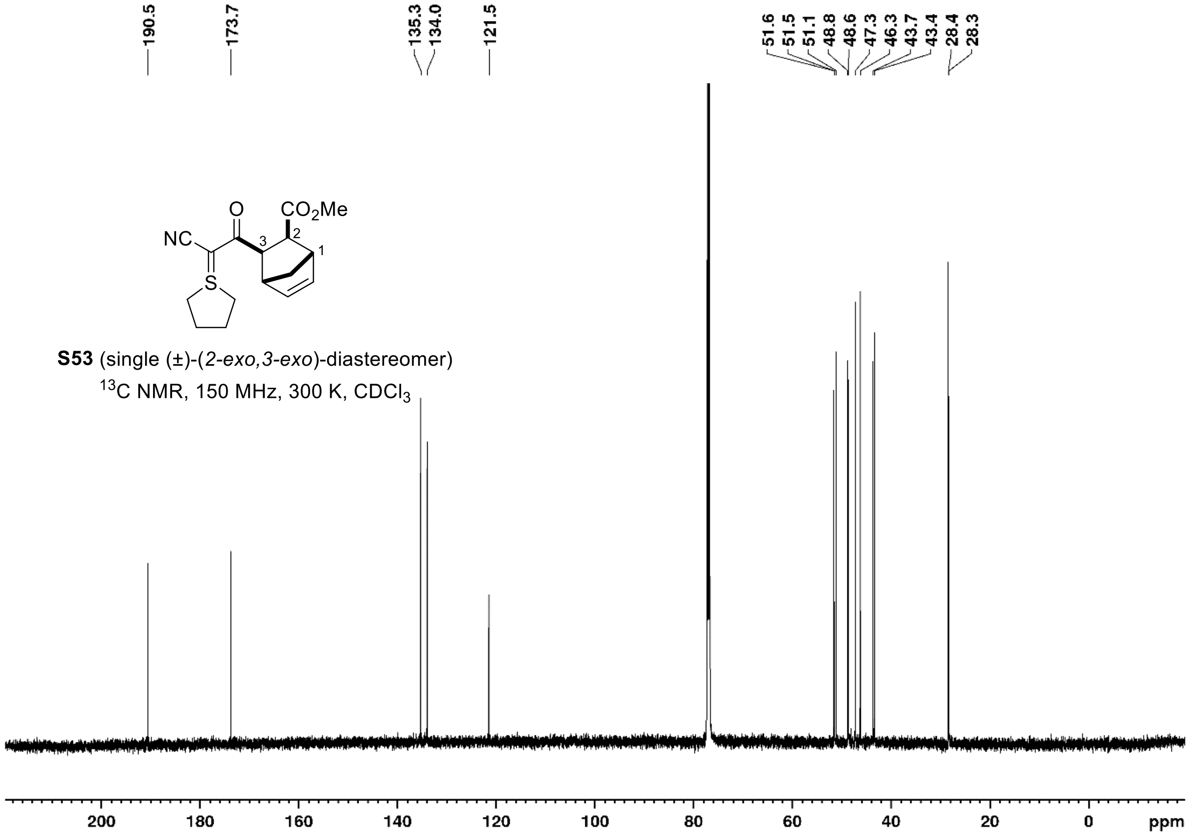

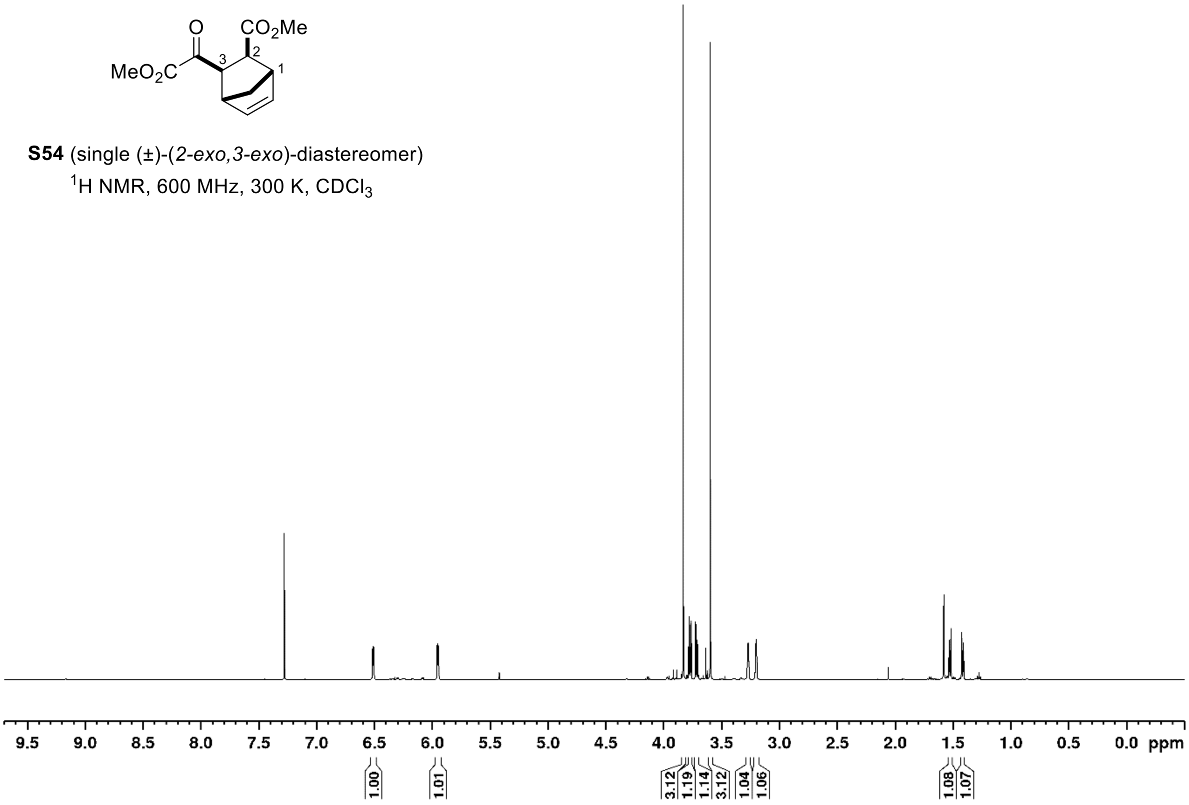

S54 (single (±)-(*2-exo,3-exo*)-diastereomer)
^{1}H NMR, 600 MHz, 300 K, $CDCl_3$

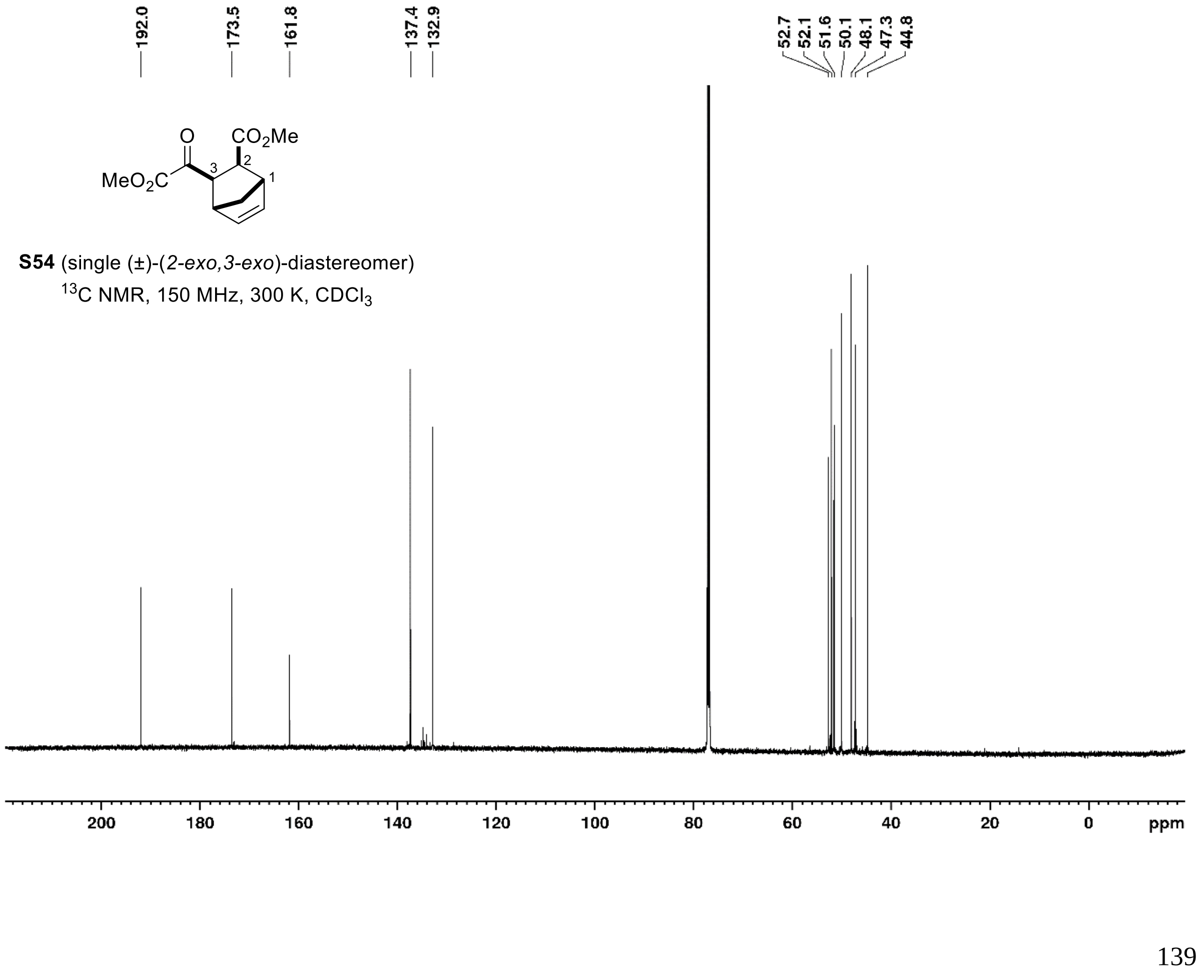

S54 (single (±)-(*2-exo,3-exo*)-diastereomer)
^{13}C NMR, 150 MHz, 300 K, $CDCl_3$

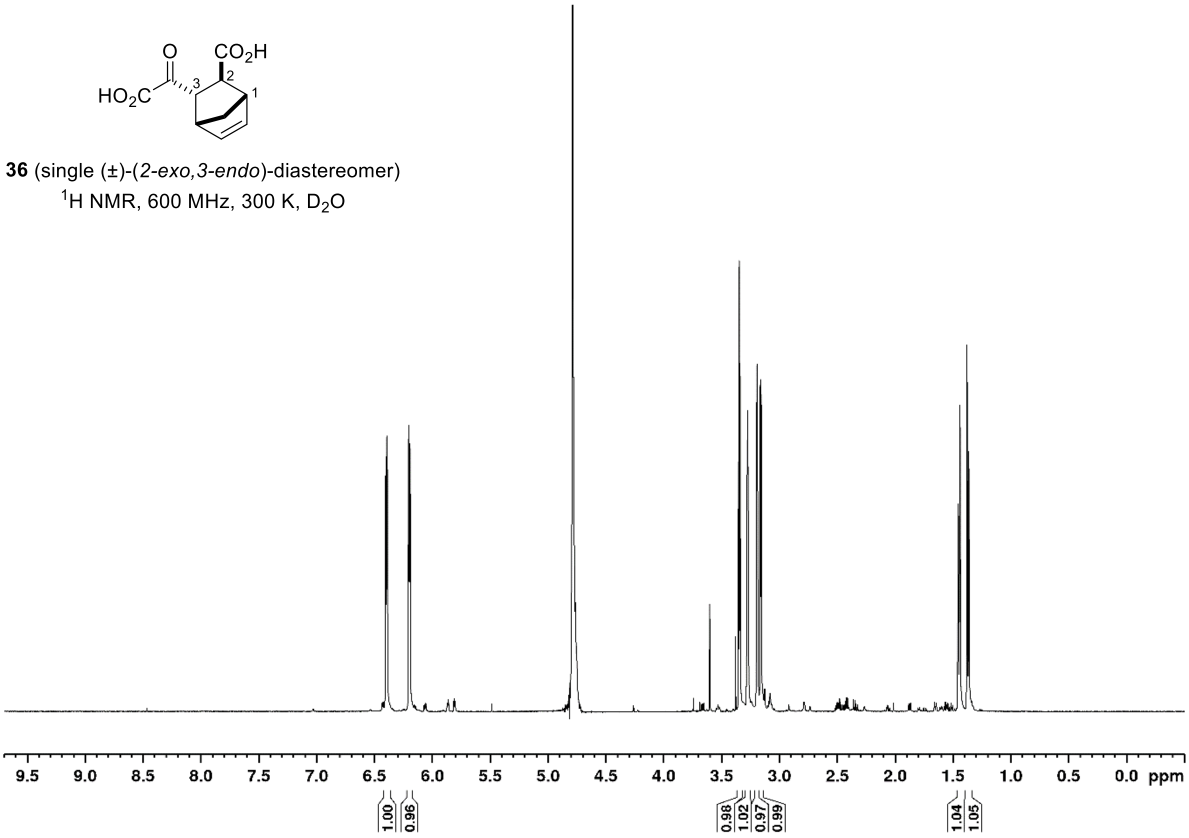
36 (single (±)-(2-exo,3-endo)-diastereomer)
^{1}H NMR, 600 MHz, 300 K, D_2O
9.5 9.0 8.5 8.0 7.5 7.0 6.5 6.0 5.5 5.0 4.5 4.0 3.5 3.0 2.5 2.0 1.5 1.0 0.5 0.0 ppm
1.00 0.96 0.98 1.02 0.97 0.99 1.04 1.05

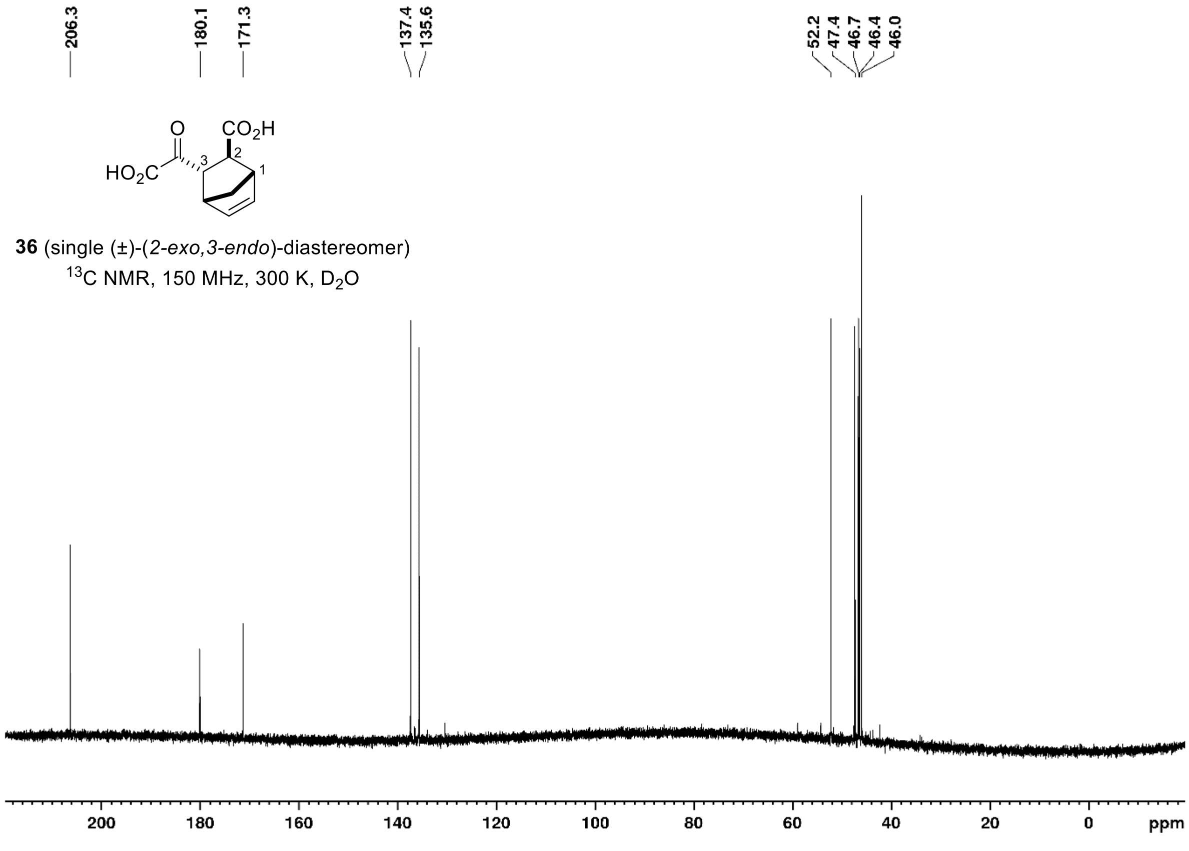
206.3
180.1
171.3
137.4
135.6
52.2
47.4
46.7
46.4
46.0
36 (single (±)-(2-exo,3-endo)-diastereomer)
^{13}C NMR, 150 MHz, 300 K, D_2O
200 180 160 140 120 100 80 60 40 20 0 ppm

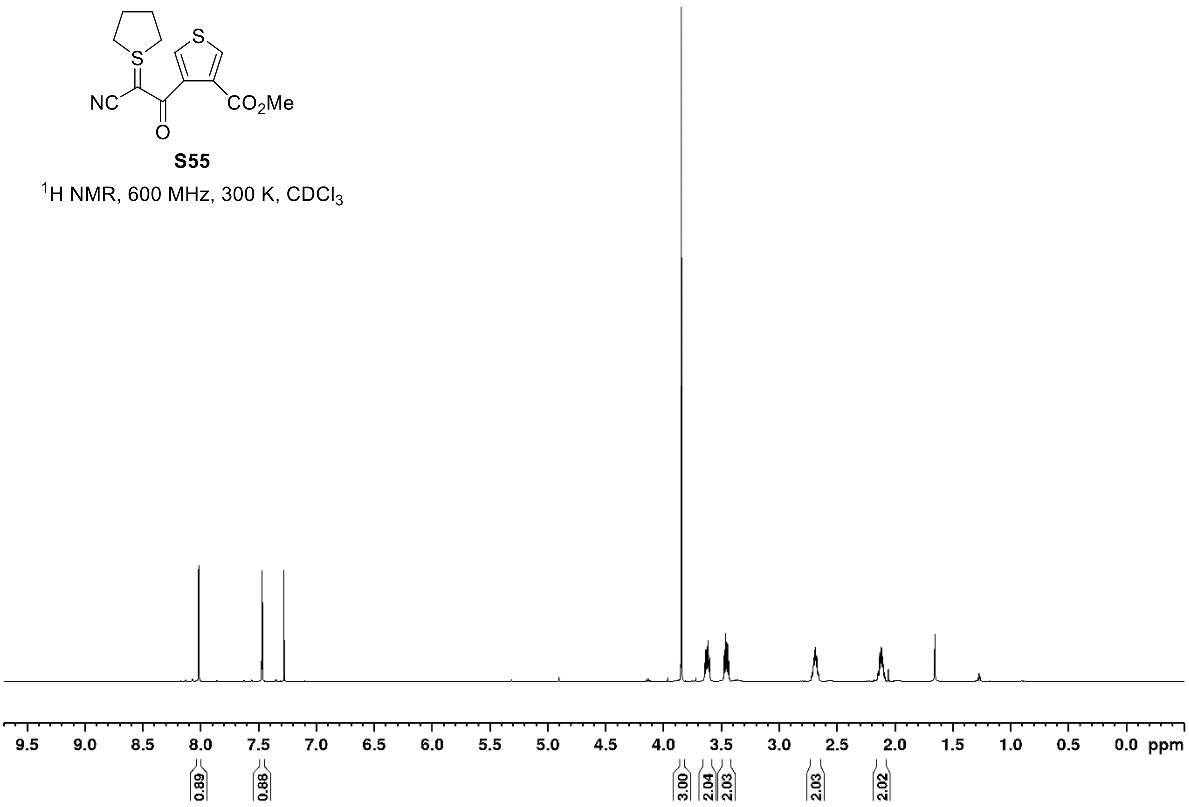
S
NC
CO2Me
O
S55
1H NMR, 600 MHz, 300 K, CDCl3
9.5 9.0 8.5 8.0 7.5 7.0 6.5 6.0 5.5 5.0 4.5 4.0 3.5 3.0 2.5 2.0 1.5 1.0 0.5 0.0 ppm
0.89
0.88
3.00
2.04
2.03
2.03
2.02

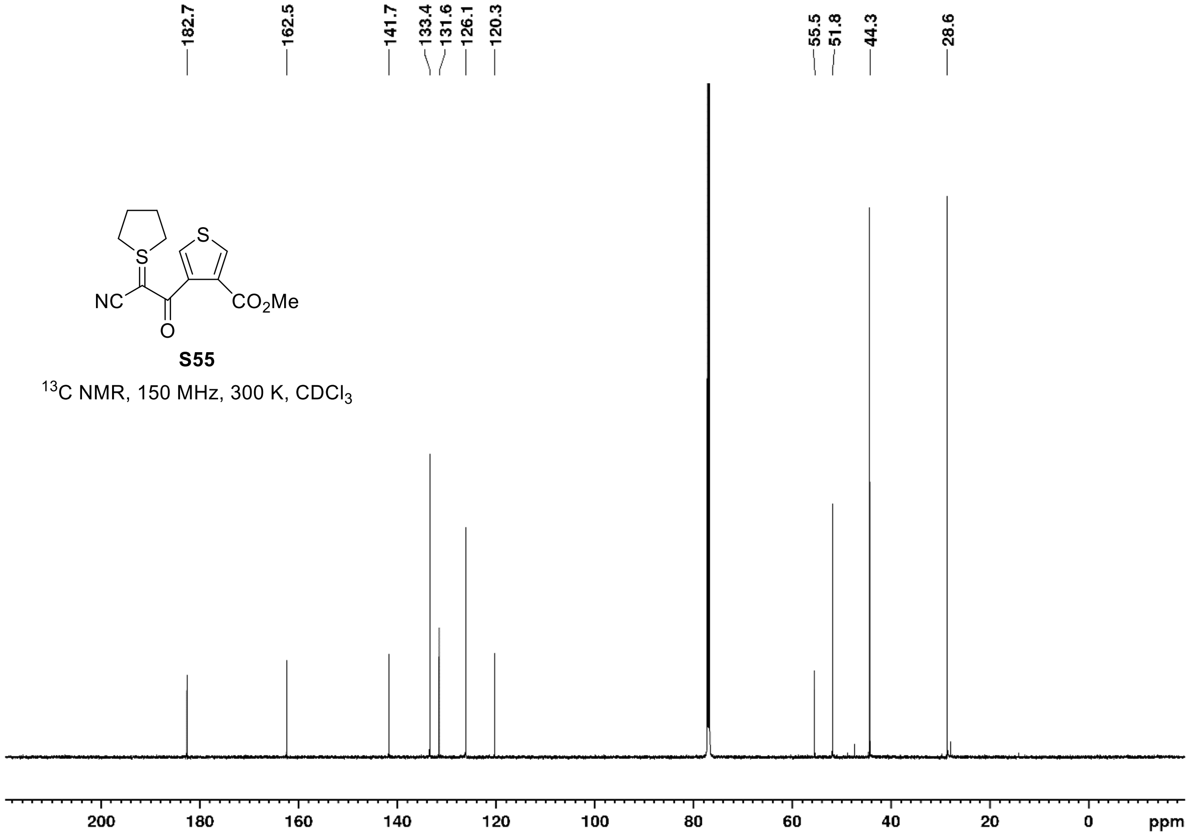
182.7
162.5
141.7
133.4
131.6
126.1
120.3
55.5
51.8
44.3
28.6
S
NC
CO2Me
O
S55
13C NMR, 150 MHz, 300 K, CDCl3
200 180 160 140 120 100 80 60 40 20 0 ppm

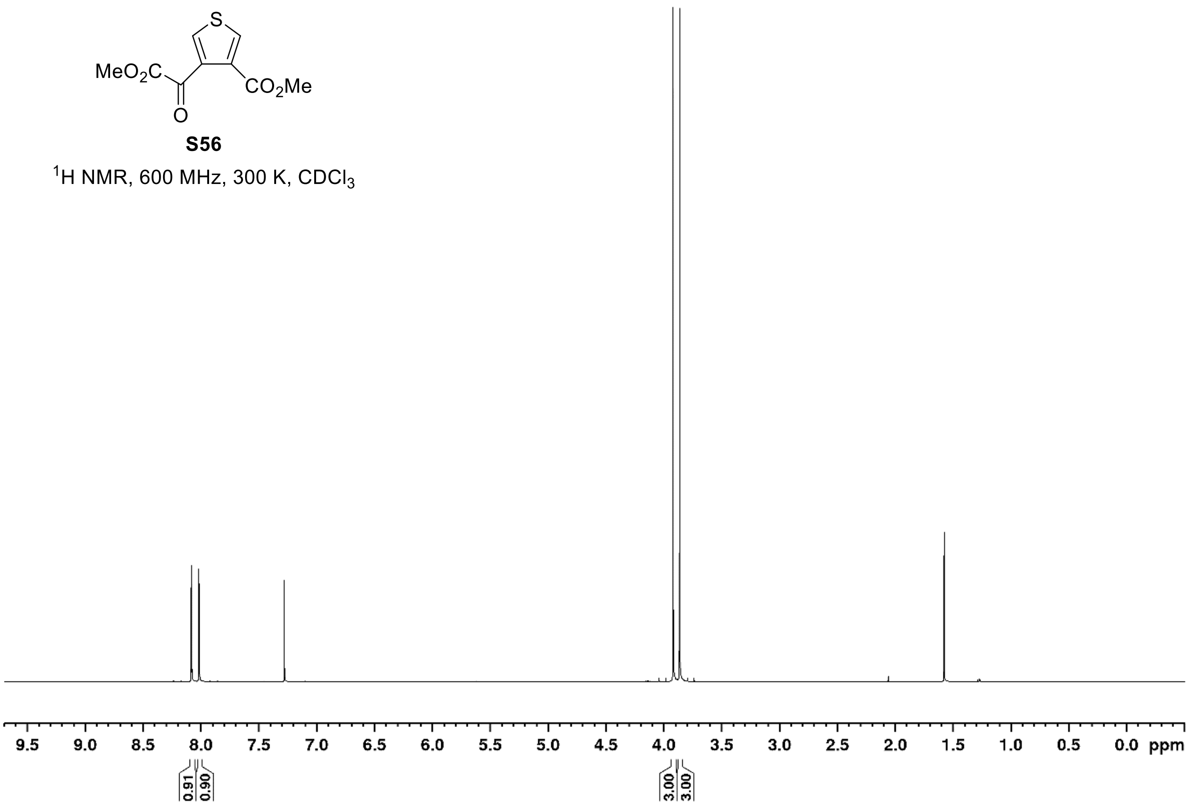

S56

^{1}H NMR, 600 MHz, 300 K, $CDCl_3$

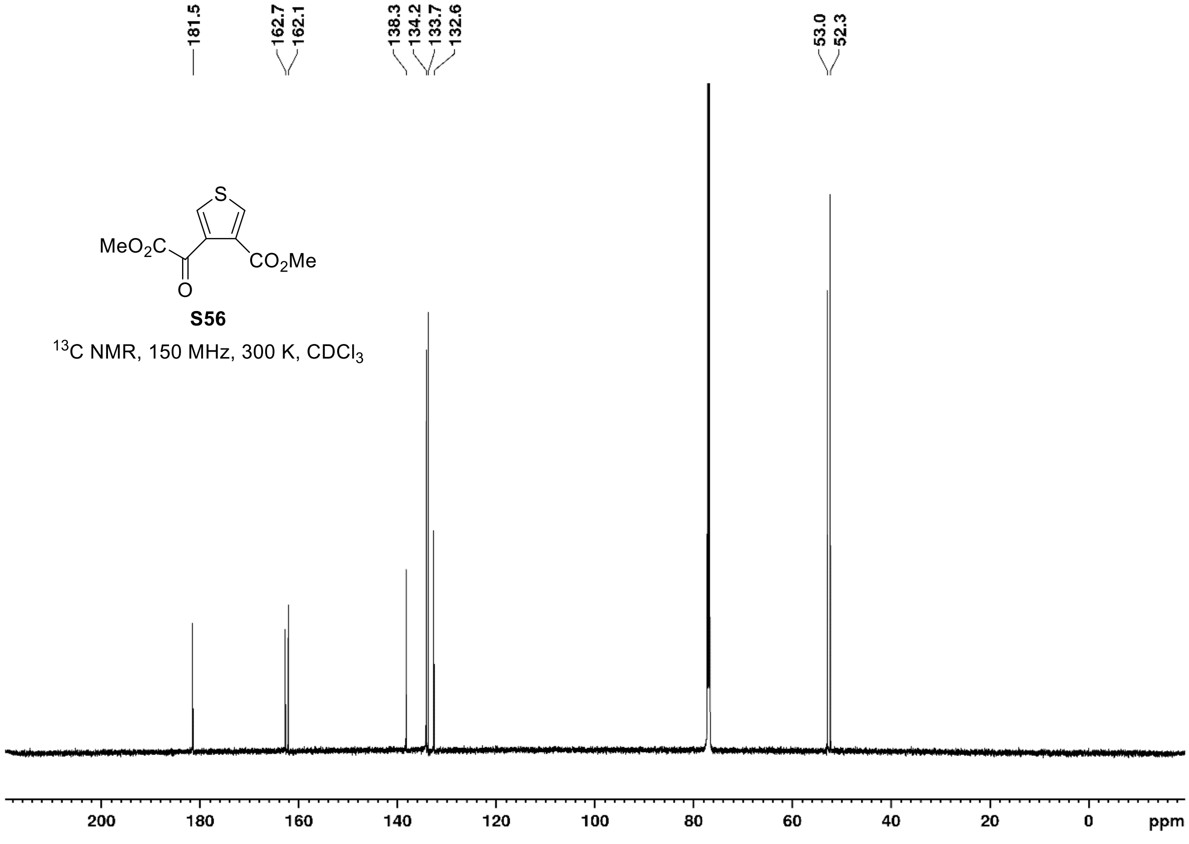

S56

^{13}C NMR, 150 MHz, 300 K, $CDCl_3$

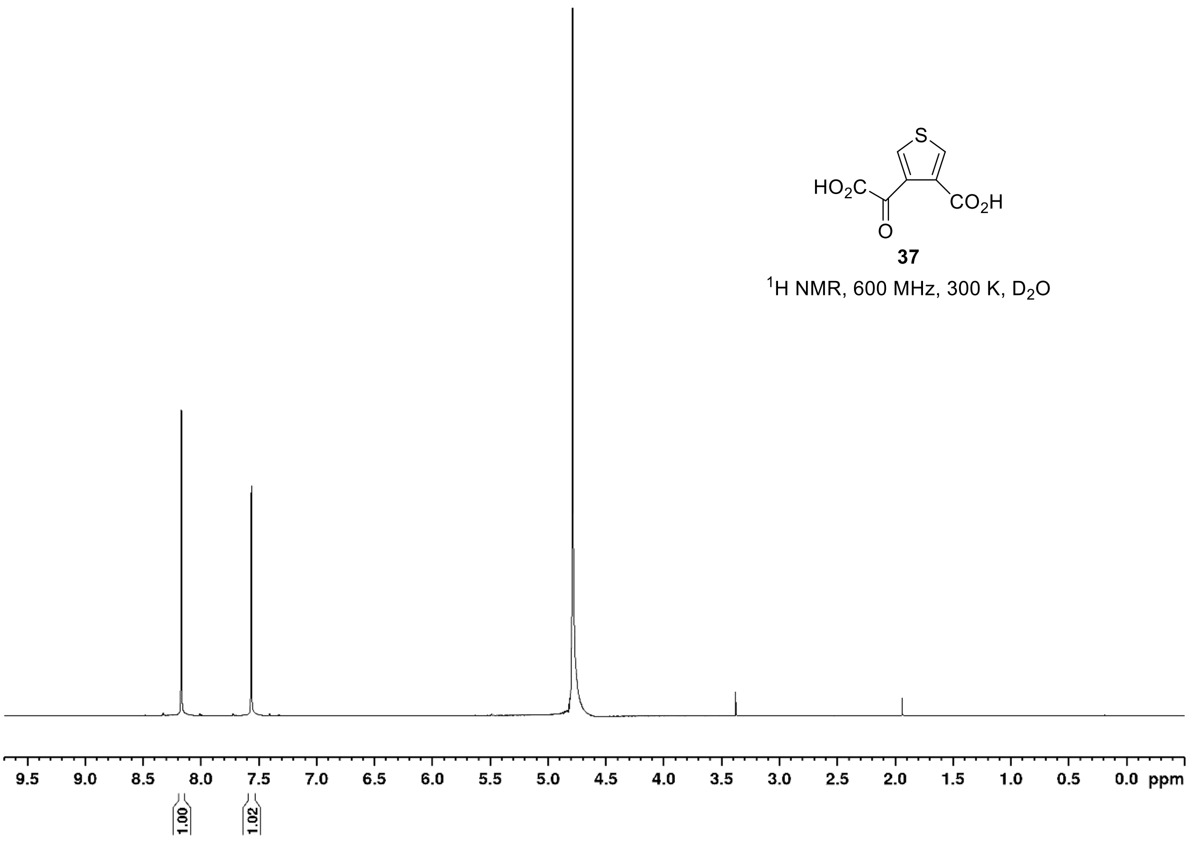
HO2C
CO2H
O
S
37
1H NMR, 600 MHz, 300 K, D2O
9.5 9.0 8.5 8.0 7.5 7.0 6.5 6.0 5.5 5.0 4.5 4.0 3.5 3.0 2.5 2.0 1.5 1.0 0.5 0.0 ppm
1.00
1.02

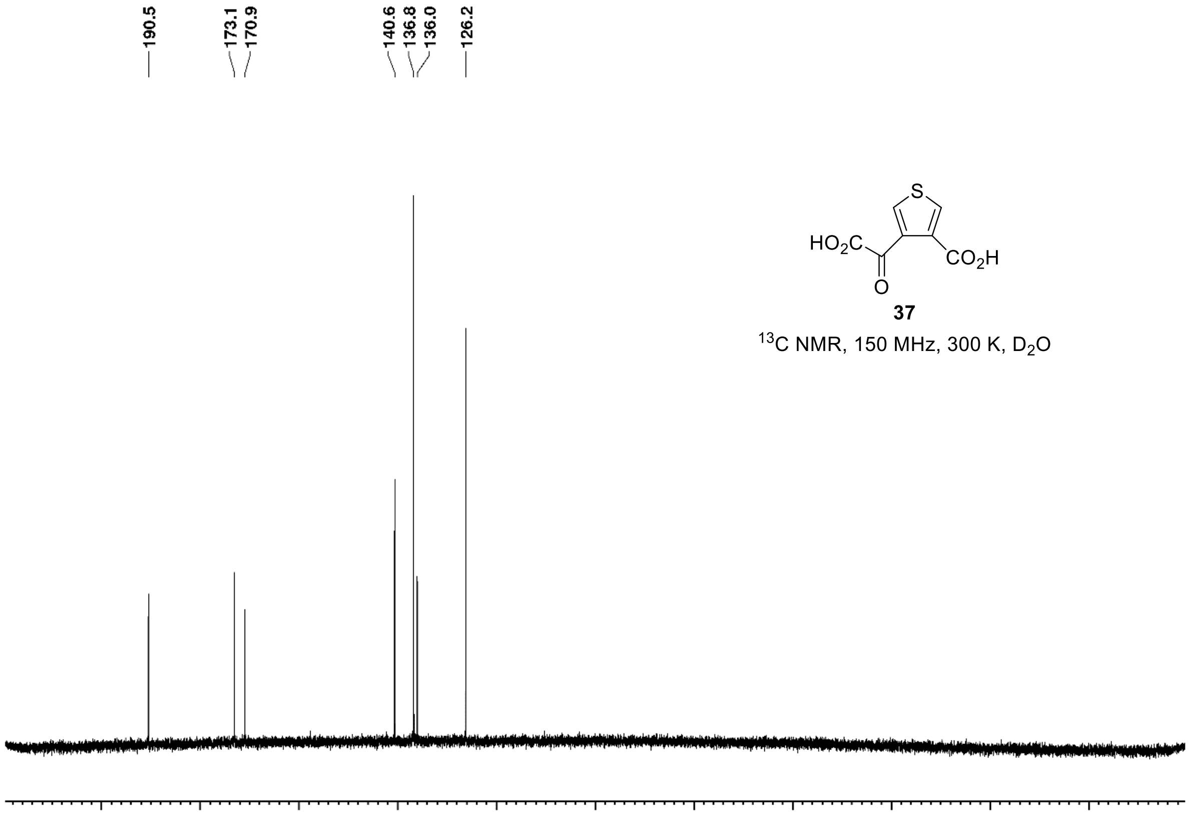
190.5
173.1
170.9
140.6
136.8
136.0
126.2
HO2C
CO2H
O
S
37
13C NMR, 150 MHz, 300 K, D2O
200 180 160 140 120 100 80 60 40 20 0 ppm

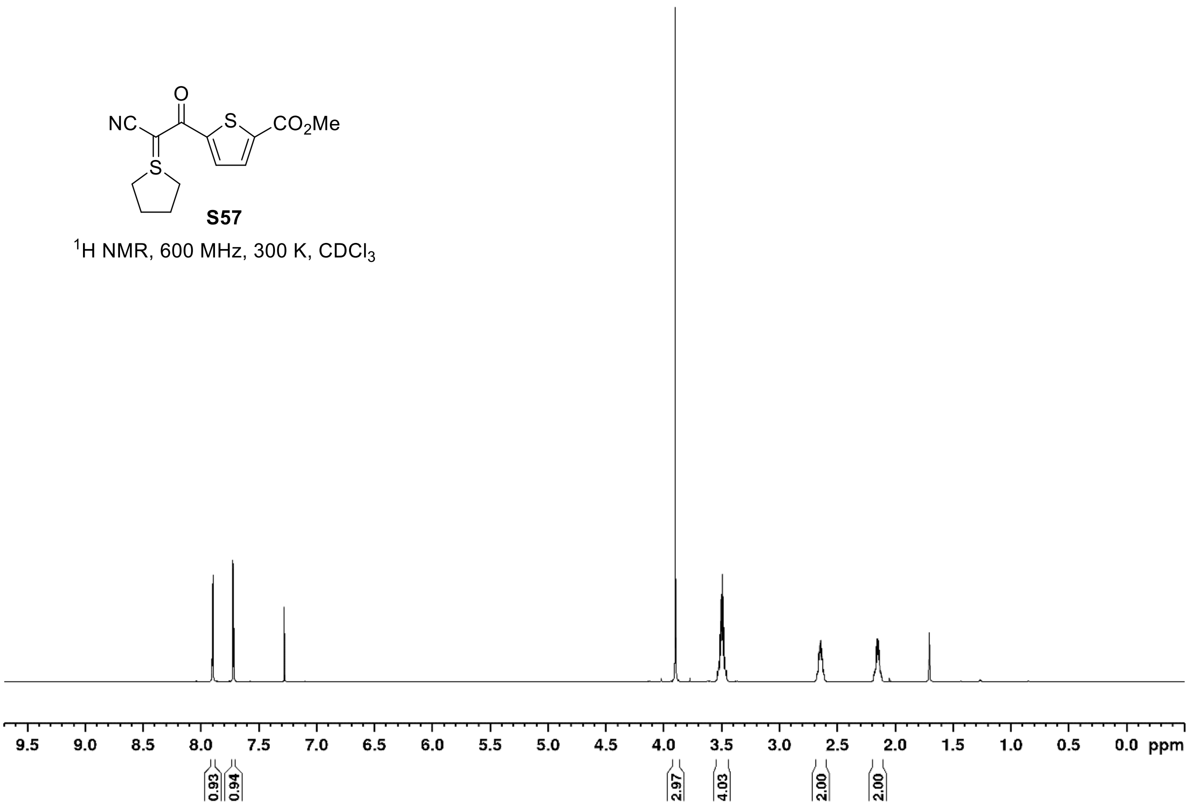
NC
O
S
CO2Me
S
S57
1H NMR, 600 MHz, 300 K, CDCl3
0.93
0.94
2.97
4.03
2.00
2.00
ppm

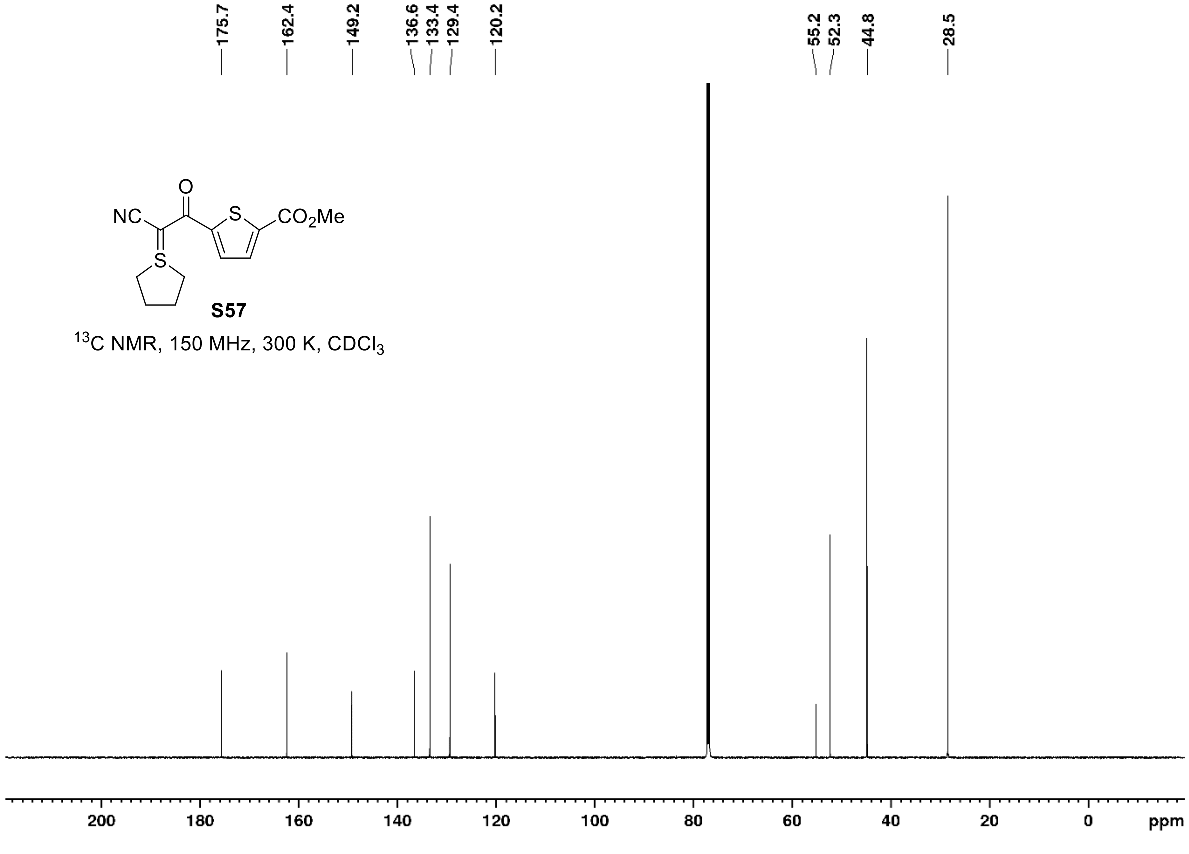
175.7
162.4
149.2
136.6
133.4
129.4
120.2
55.2
52.3
44.8
28.5
NC
O
S
CO2Me
S
S57
13C NMR, 150 MHz, 300 K, CDCl3
ppm

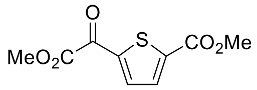

S58

^1H NMR, 600 MHz, 300 K, $CDCl_3$

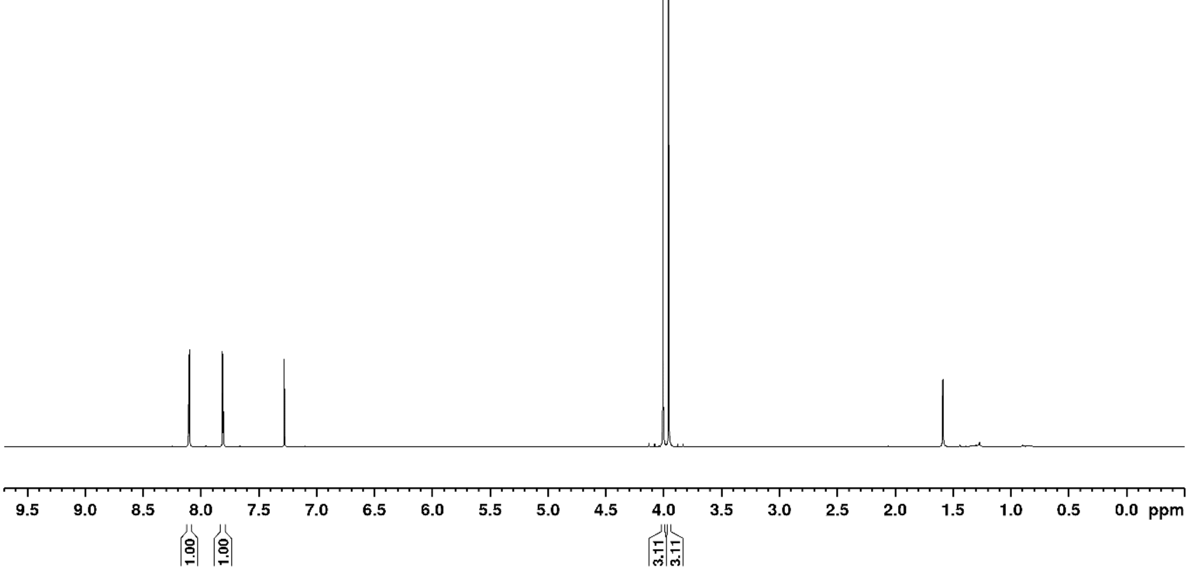

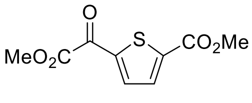

S58

^{13}C NMR, 150 MHz, 300 K, $CDCl_3$

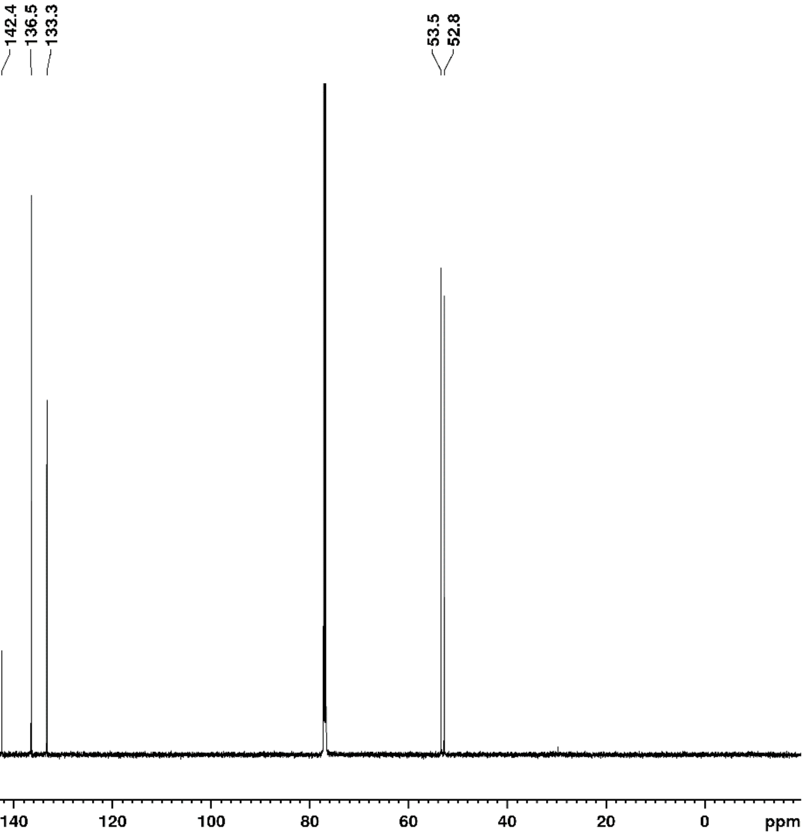

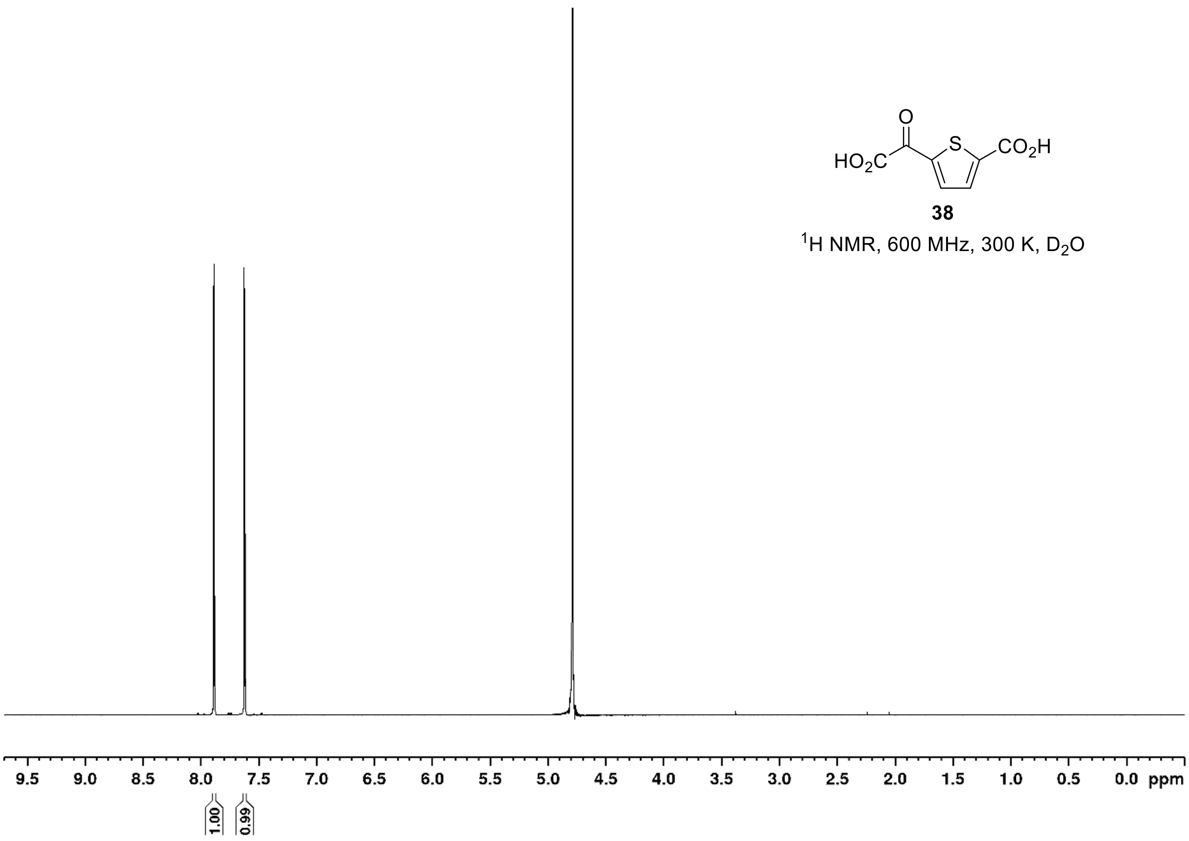
HO2C
CO2H
O
S
38
1H NMR, 600 MHz, 300 K, D2O
9.5 9.0 8.5 8.0 7.5 7.0 6.5 6.0 5.5 5.0 4.5 4.0 3.5 3.0 2.5 2.0 1.5 1.0 0.5 0.0 ppm
1.00
0.99

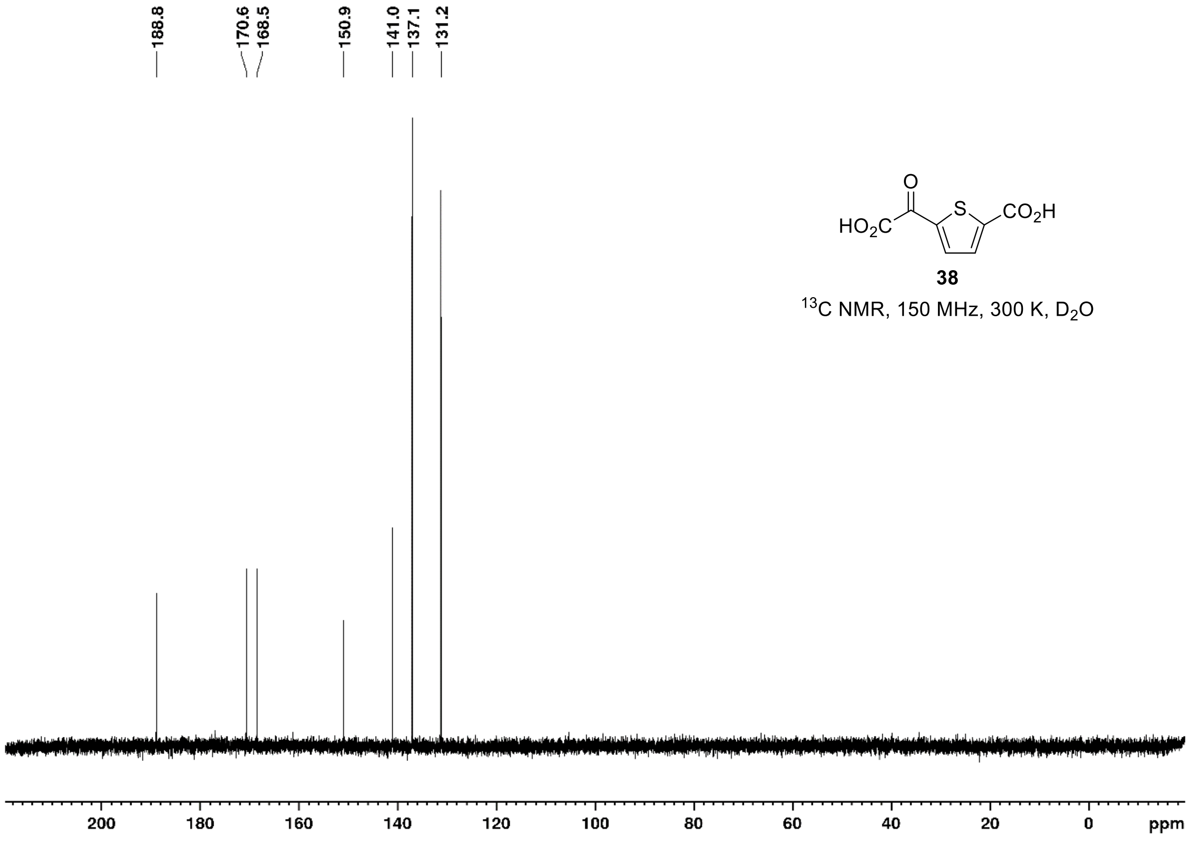
188.8
170.6
168.5
150.9
141.0
137.1
131.2
HO2C
CO2H
O
S
38
13C NMR, 150 MHz, 300 K, D2O
200 180 160 140 120 100 80 60 40 20 0 ppm

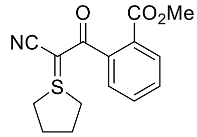

S59

^{1}H NMR, 600 MHz, 300 K, $CDCl_3$

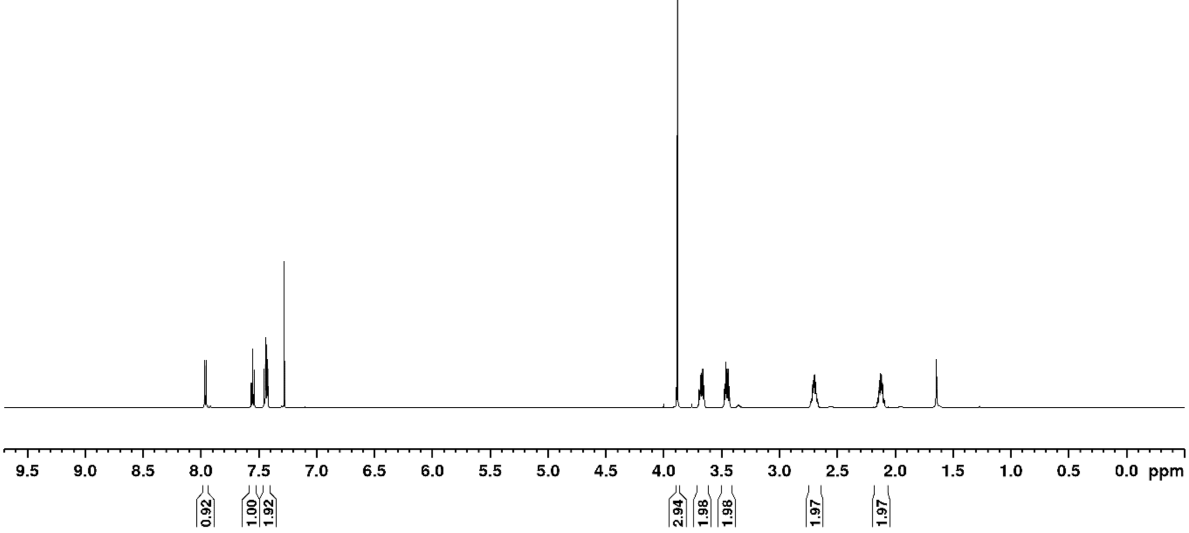

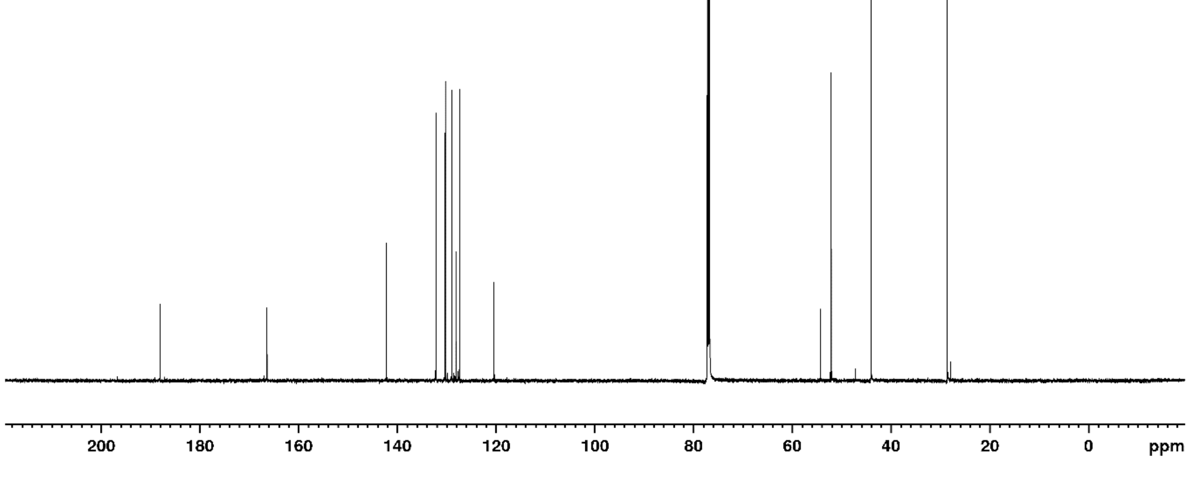

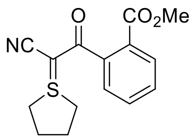

S59

^{13}C NMR, 150 MHz, 300 K, $CDCl_3$

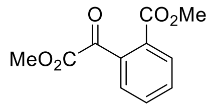

S60

^1H NMR, 600 MHz, 300 K, $CDCl_3$

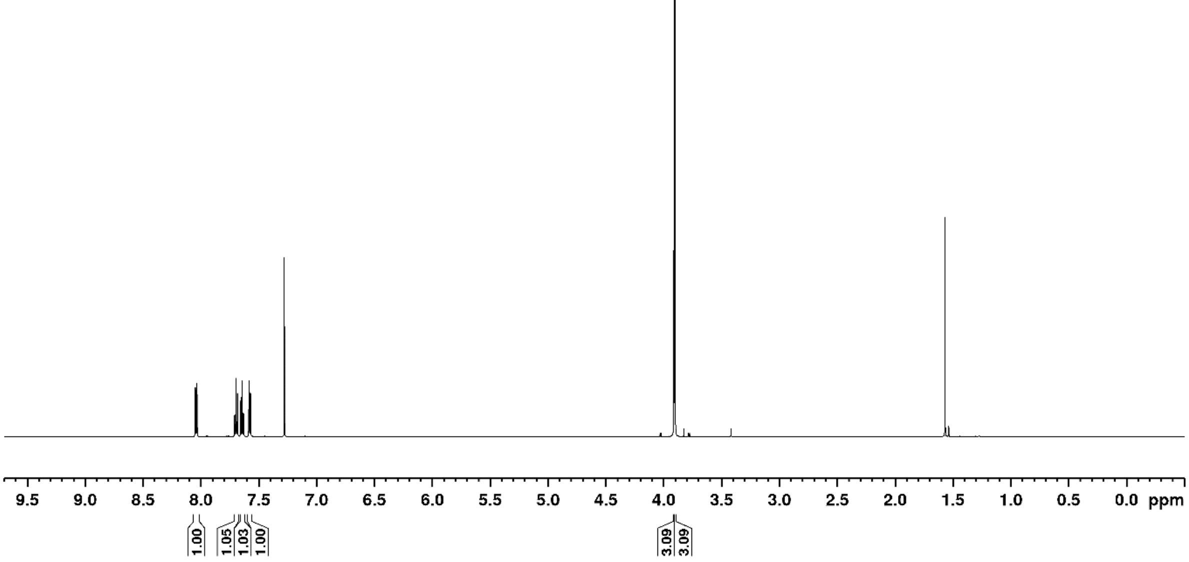

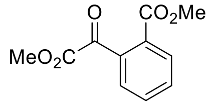

S60

^{13}C NMR, 150 MHz, 300 K, $CDCl_3$

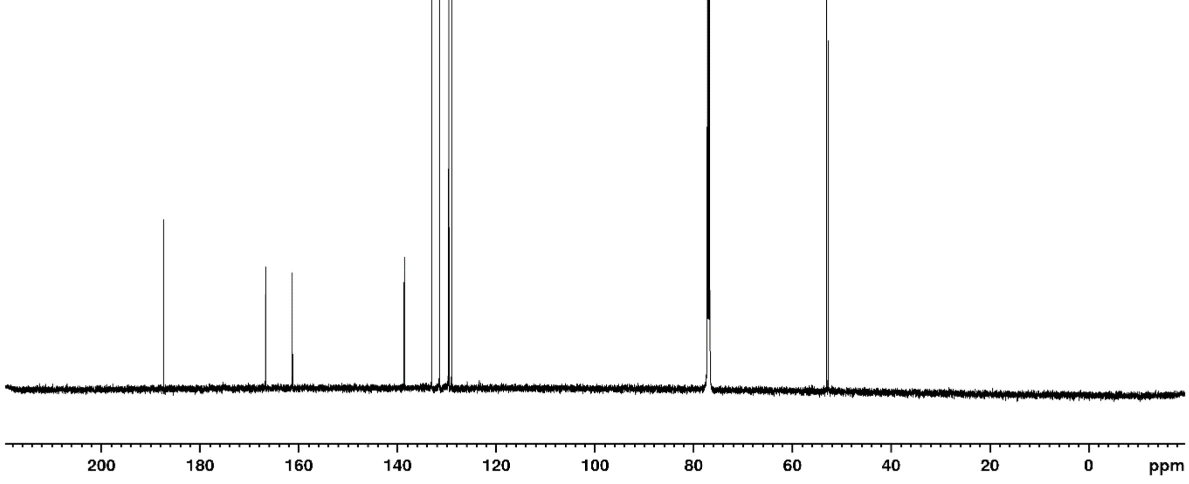

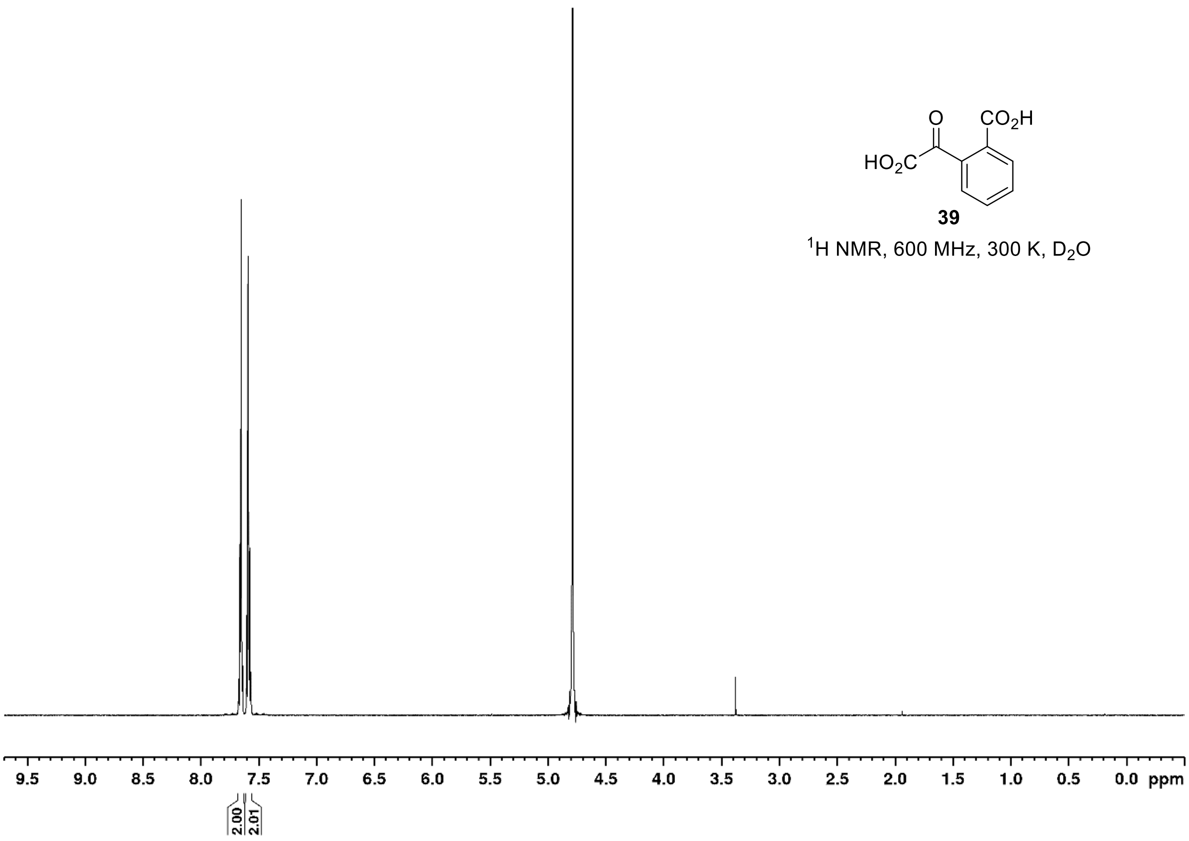
O
CO2H
HO2C
39
1H NMR, 600 MHz, 300 K, D2O
9.5 9.0 8.5 8.0 7.5 7.0 6.5 6.0 5.5 5.0 4.5 4.0 3.5 3.0 2.5 2.0 1.5 1.0 0.5 0.0 ppm
2.00
2.01

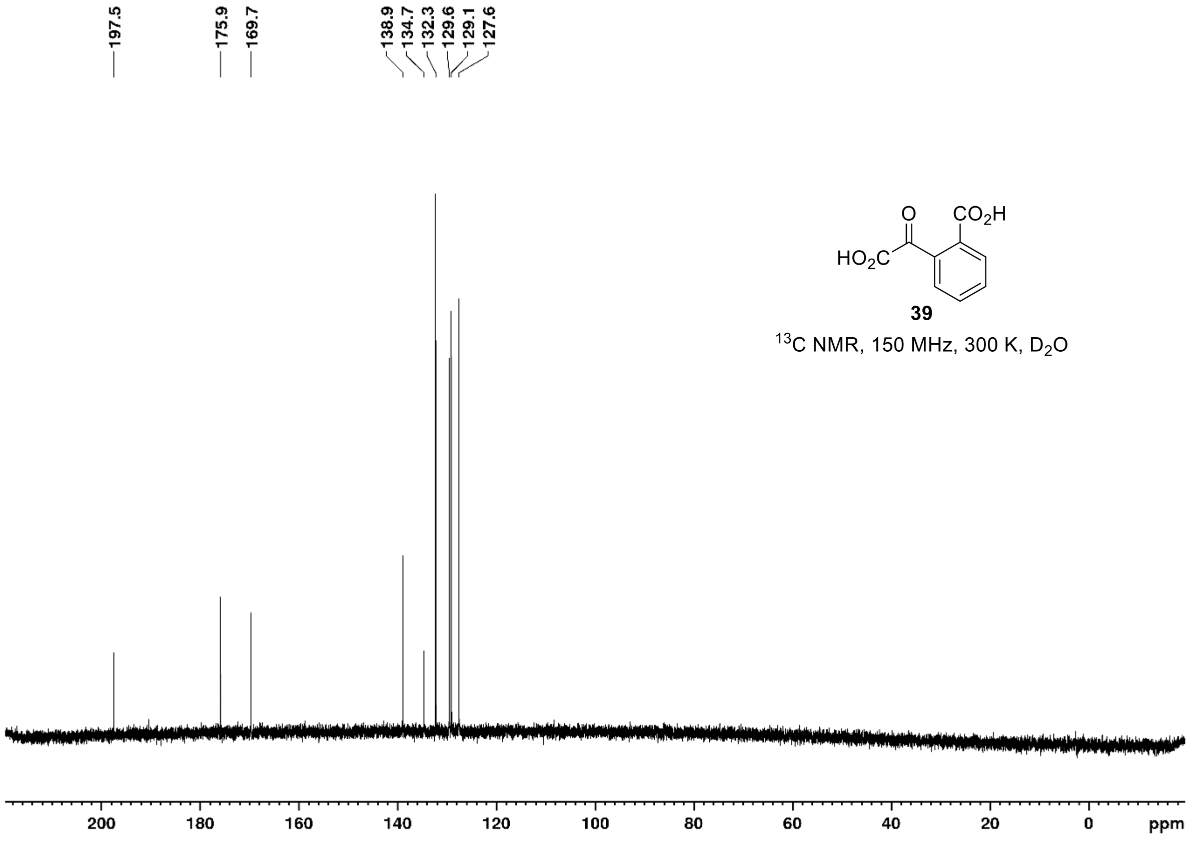
197.5
175.9
169.7
138.9
134.7
132.3
129.6
129.1
127.6
O
CO2H
HO2C
39
13C NMR, 150 MHz, 300 K, D2O
200 180 160 140 120 100 80 60 40 20 0 ppm

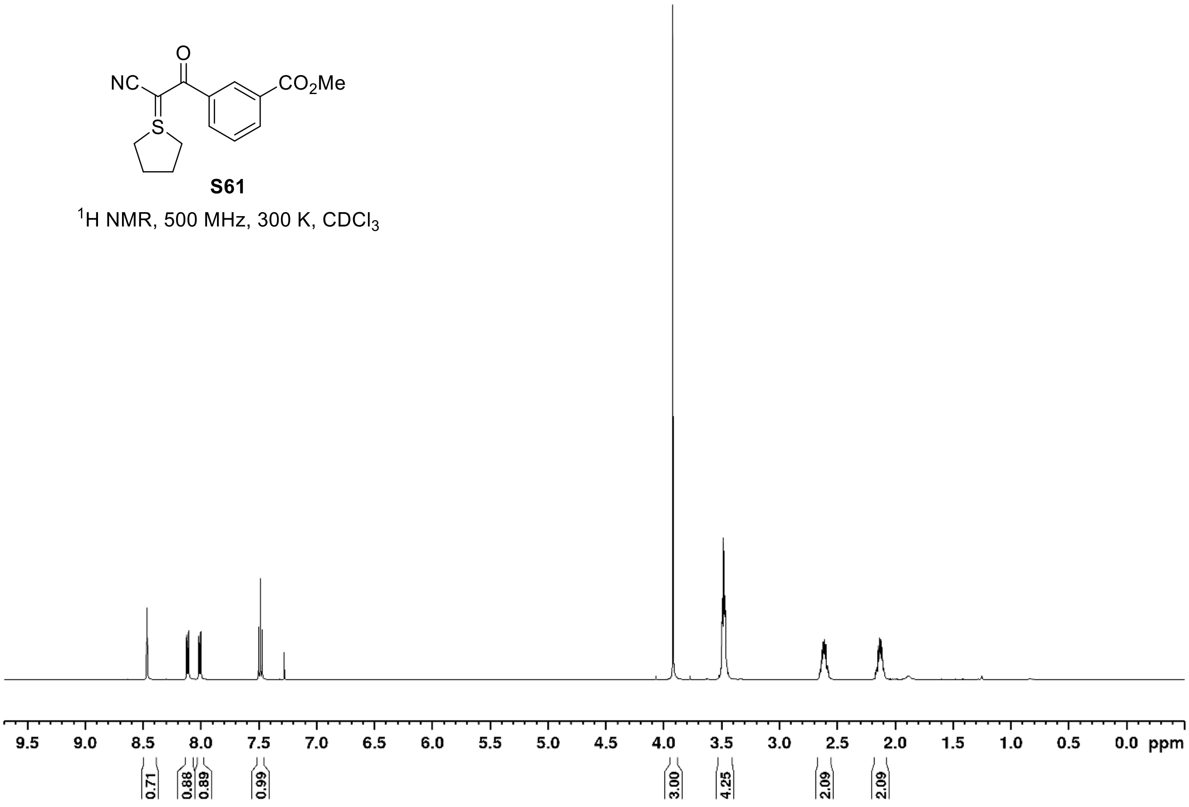
NC
O
CO2Me
S
S61
1H NMR, 500 MHz, 300 K, CDCl3
9.5 9.0 8.5 8.0 7.5 7.0 6.5 6.0 5.5 5.0 4.5 4.0 3.5 3.0 2.5 2.0 1.5 1.0 0.5 0.0 ppm
0.71 0.88 0.89 0.99 3.00 4.25 2.09 2.09

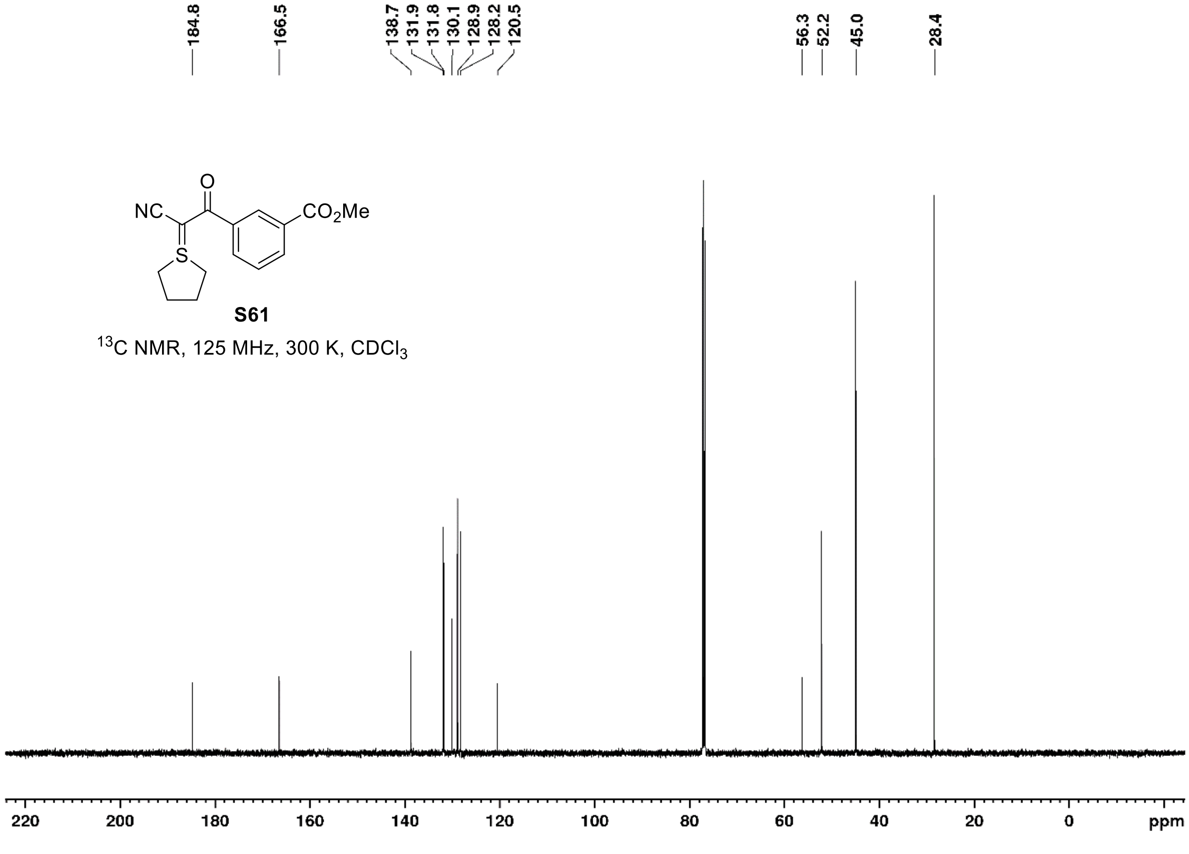
184.8
166.5
138.7
131.9
131.8
130.1
128.9
128.2
120.5
56.3
52.2
45.0
28.4
NC
O
CO2Me
S
S61
13C NMR, 125 MHz, 300 K, CDCl3
220 200 180 160 140 120 100 80 60 40 20 0 ppm

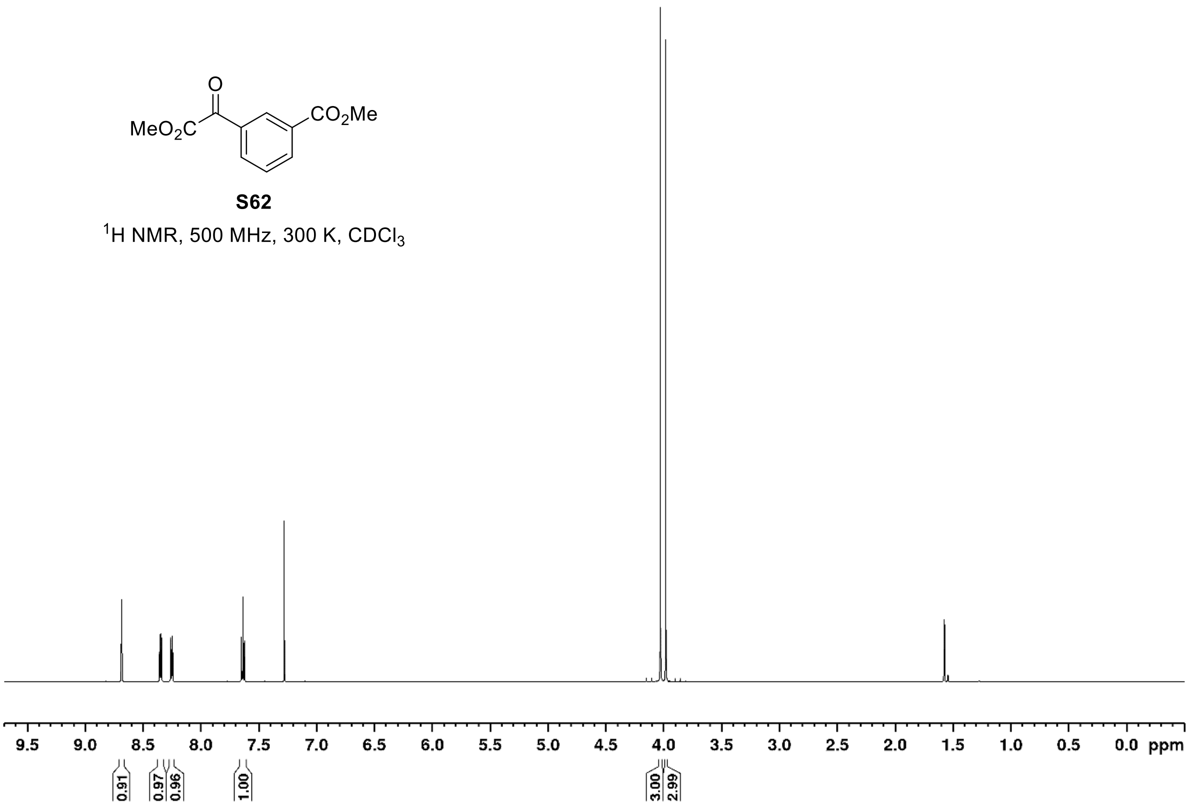
MeO2C
CO2Me
O
S62
1H NMR, 500 MHz, 300 K, CDCl3
9.5 9.0 8.5 8.0 7.5 7.0 6.5 6.0 5.5 5.0 4.5 4.0 3.5 3.0 2.5 2.0 1.5 1.0 0.5 0.0 ppm
0.91
0.97
0.96
1.00
3.00
2.99

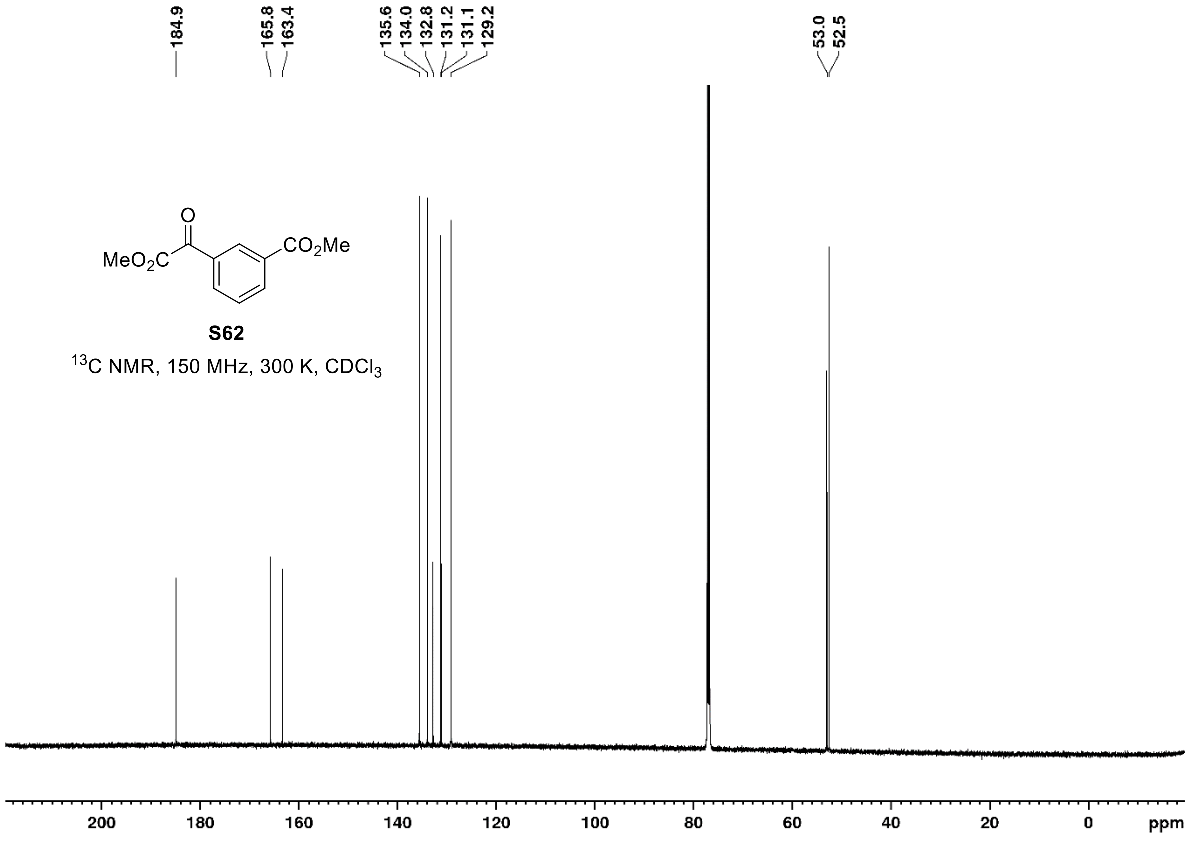
184.9
165.8
163.4
135.6
134.0
132.8
131.2
131.1
129.2
53.0
52.5
MeO2C
CO2Me
O
S62
13C NMR, 150 MHz, 300 K, CDCl3
200 180 160 140 120 100 80 60 40 20 0 ppm

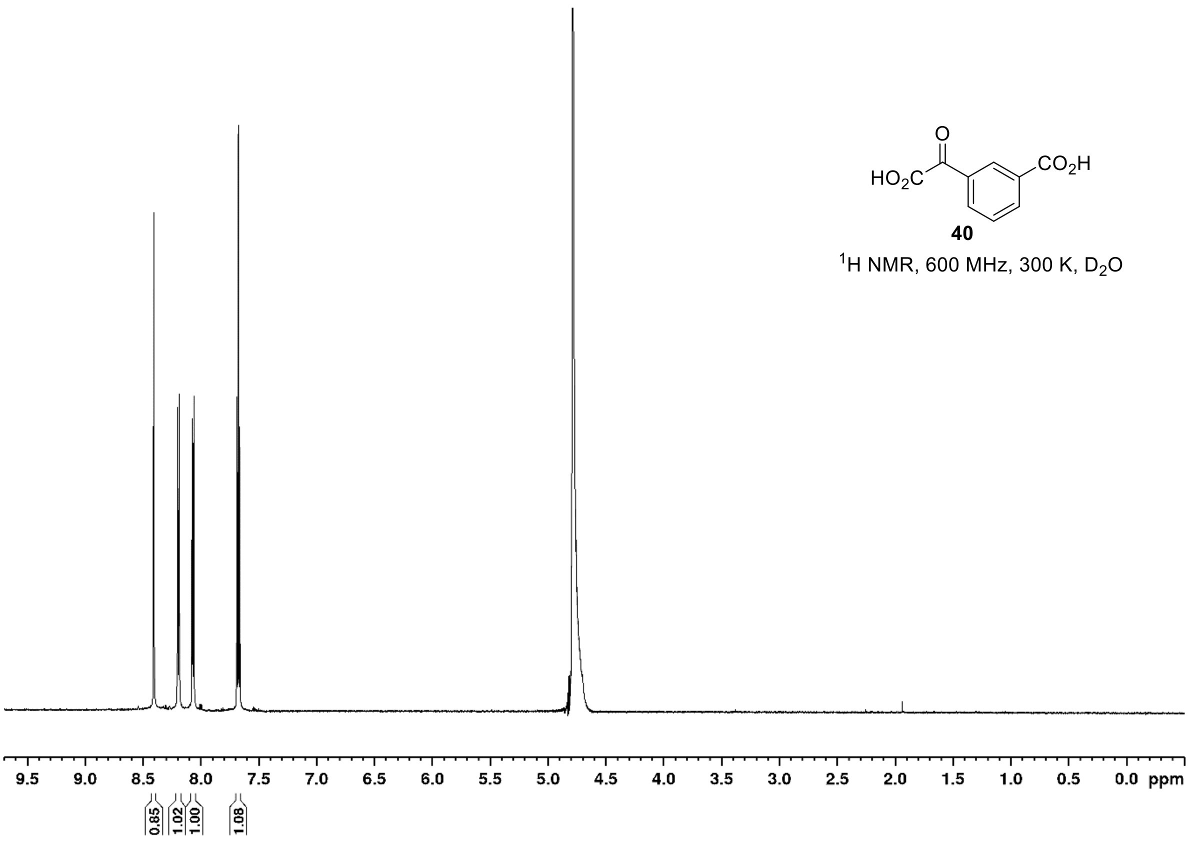
HO2C
CO2H
O
40
1H NMR, 600 MHz, 300 K, D2O
9.5 9.0 8.5 8.0 7.5 7.0 6.5 6.0 5.5 5.0 4.5 4.0 3.5 3.0 2.5 2.0 1.5 1.0 0.5 0.0 ppm
0.85 1.02 1.00 1.08

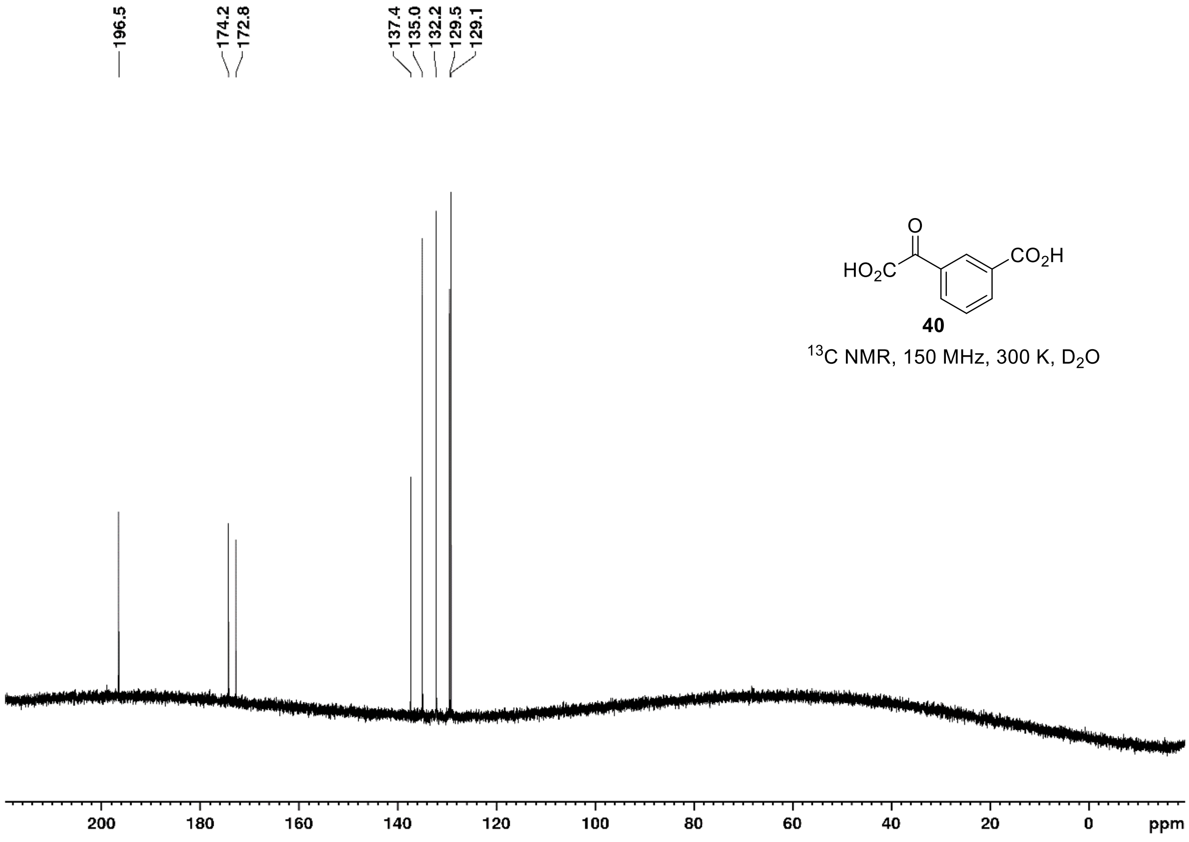
196.5
174.2
172.8
137.4
135.0
132.2
129.5
129.1
HO2C
CO2H
O
40
13C NMR, 150 MHz, 300 K, D2O
200 180 160 140 120 100 80 60 40 20 0 ppm

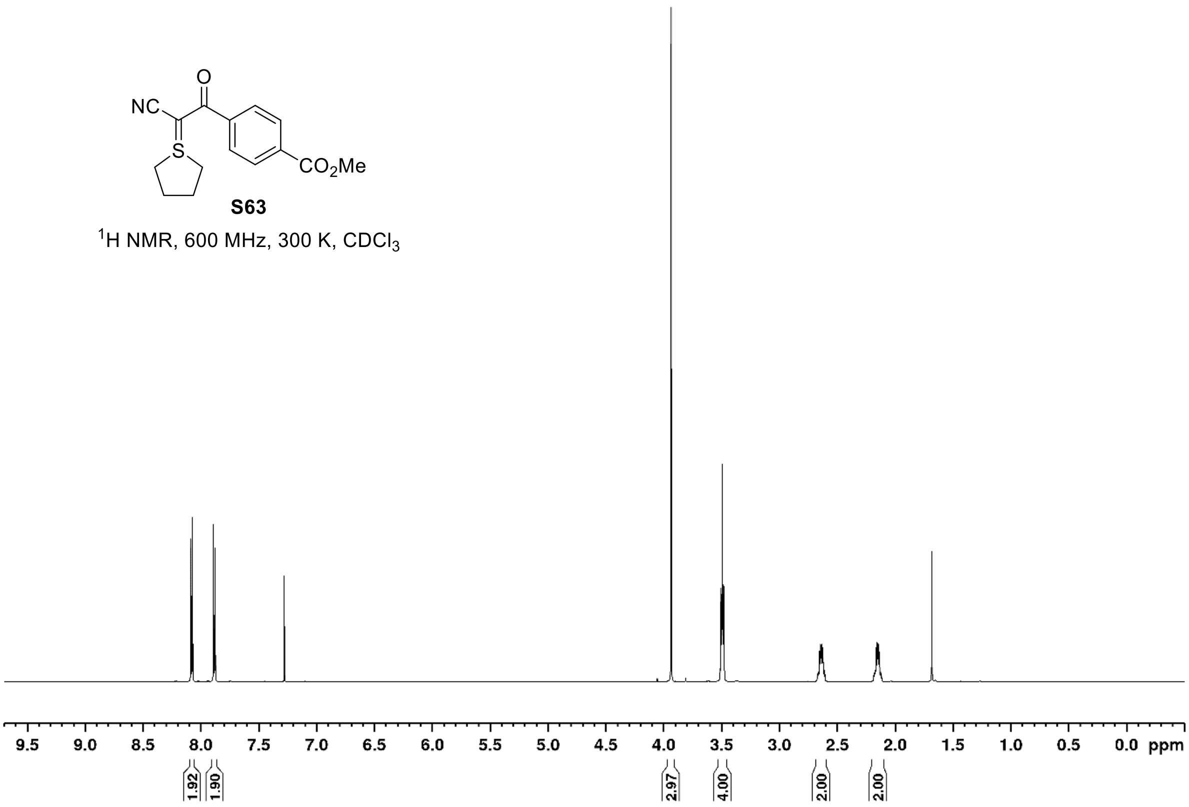

NC
O
S
CO2Me
S63
1H NMR, 600 MHz, 300 K, CDCl3
9.5 9.0 8.5 8.0 7.5 7.0 6.5 6.0 5.5 5.0 4.5 4.0 3.5 3.0 2.5 2.0 1.5 1.0 0.5 0.0 ppm
1.92
1.90
2.97
4.00
2.00
2.00

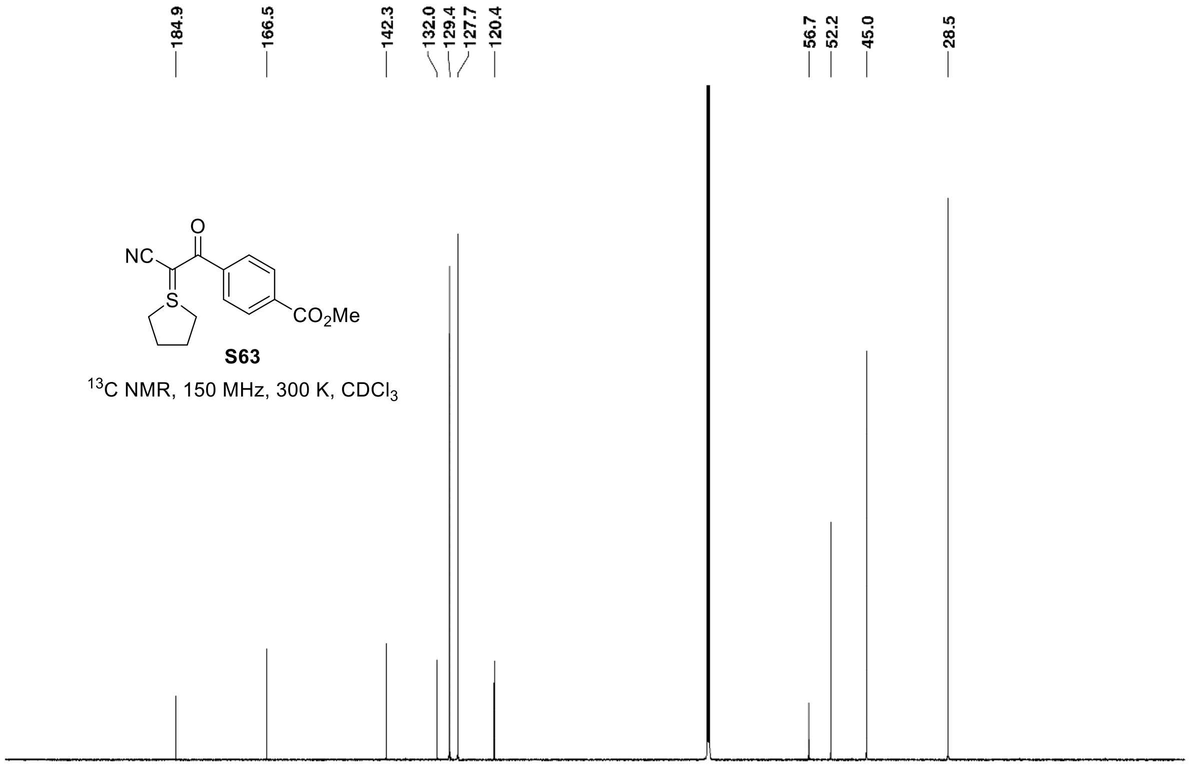

184.9
166.5
142.3
132.0
129.4
127.7
120.4
56.7
52.2
45.0
28.5
NC
O
S
CO2Me
S63
13C NMR, 150 MHz, 300 K, CDCl3
200 180 160 140 120 100 80 60 40 20 0 ppm

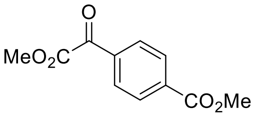

S64

^{1}H NMR, 600 MHz, 300 K, $CDCl_3$

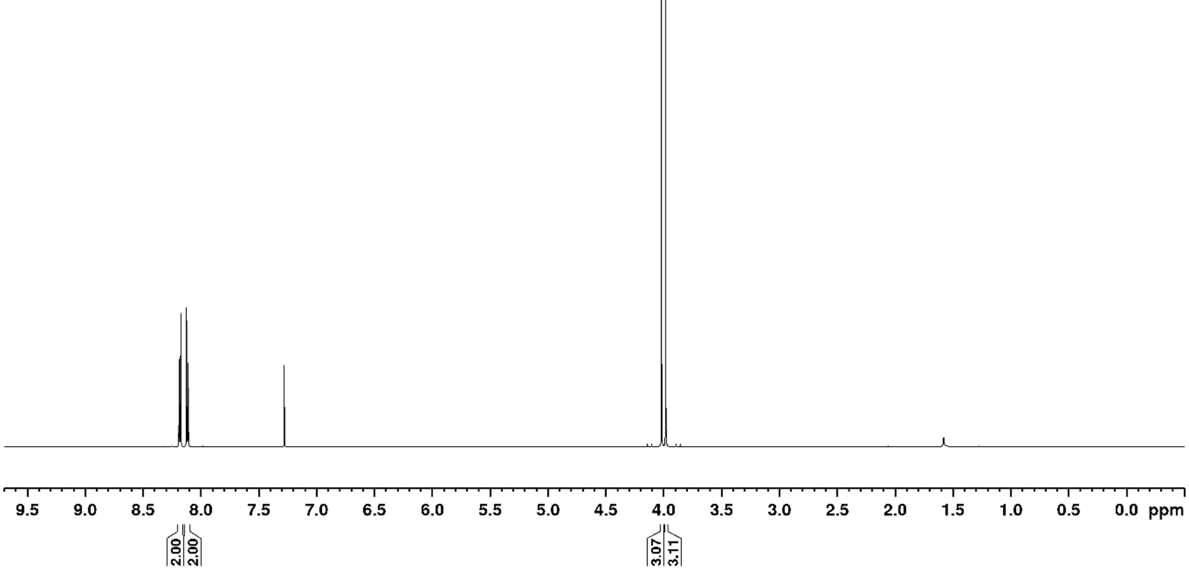

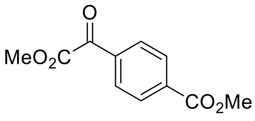

S64

^{13}C NMR, 150 MHz, 300 K, $CDCl_3$

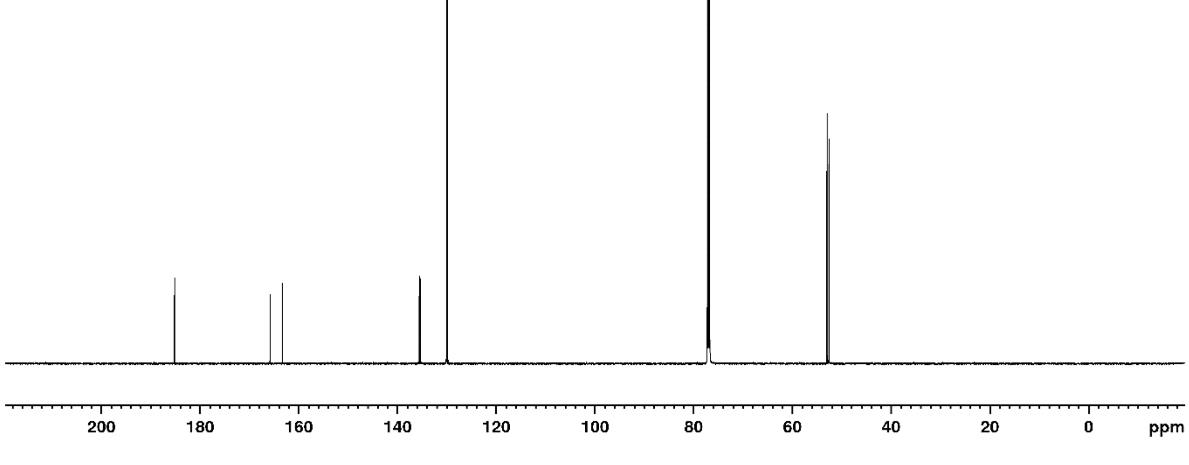

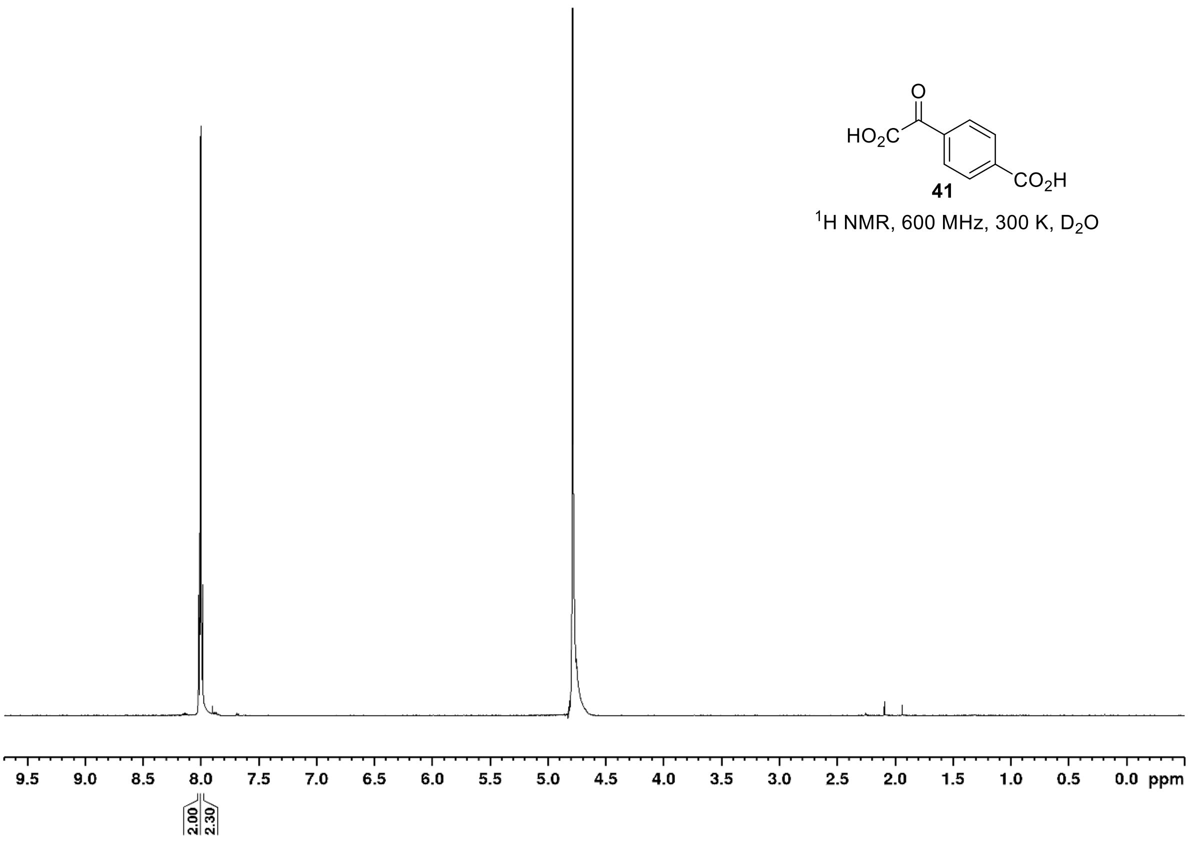
O
HO2C
CO2H
41
1H NMR, 600 MHz, 300 K, D2O
9.5 9.0 8.5 8.0 7.5 7.0 6.5 6.0 5.5 5.0 4.5 4.0 3.5 3.0 2.5 2.0 1.5 1.0 0.5 0.0 ppm
2.00
2.30

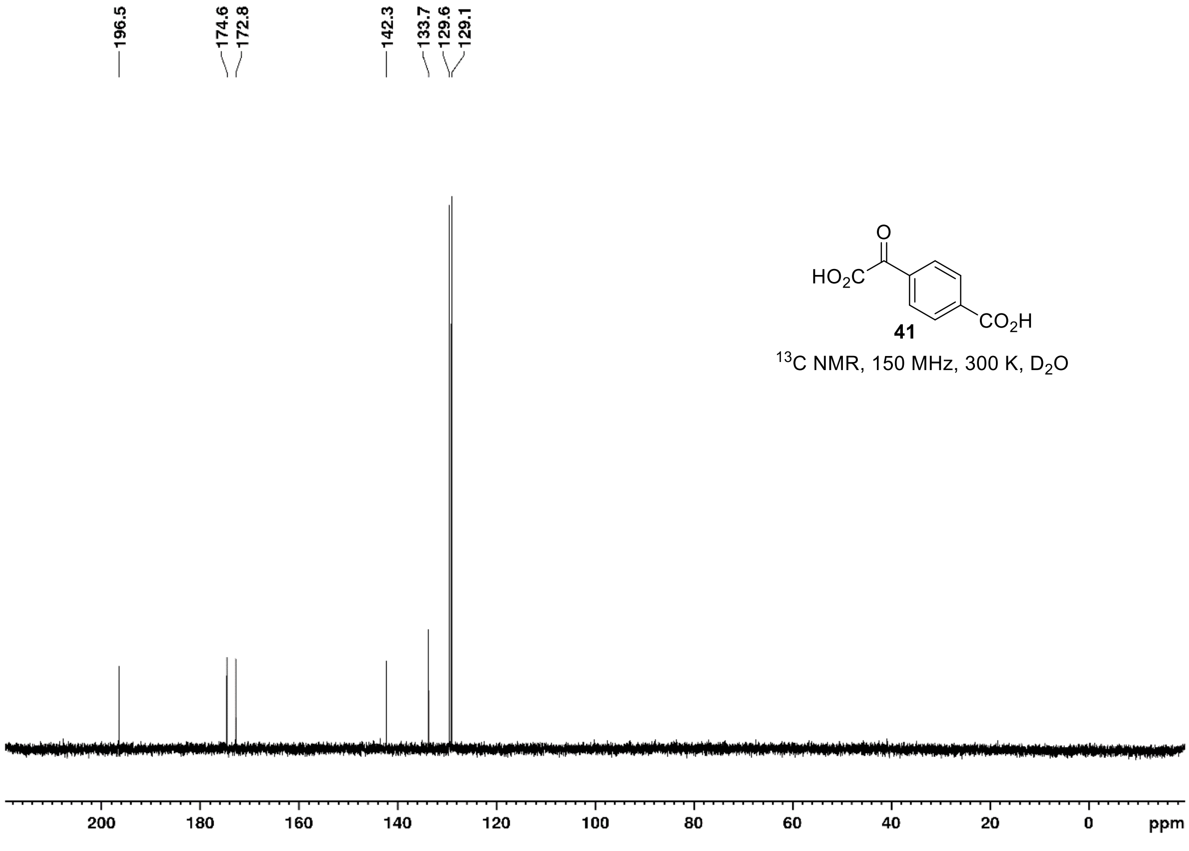
196.5
174.6
172.8
142.3
133.7
129.6
129.1
O
HO2C
CO2H
41
13C NMR, 150 MHz, 300 K, D2O
200 180 160 140 120 100 80 60 40 20 0 ppm

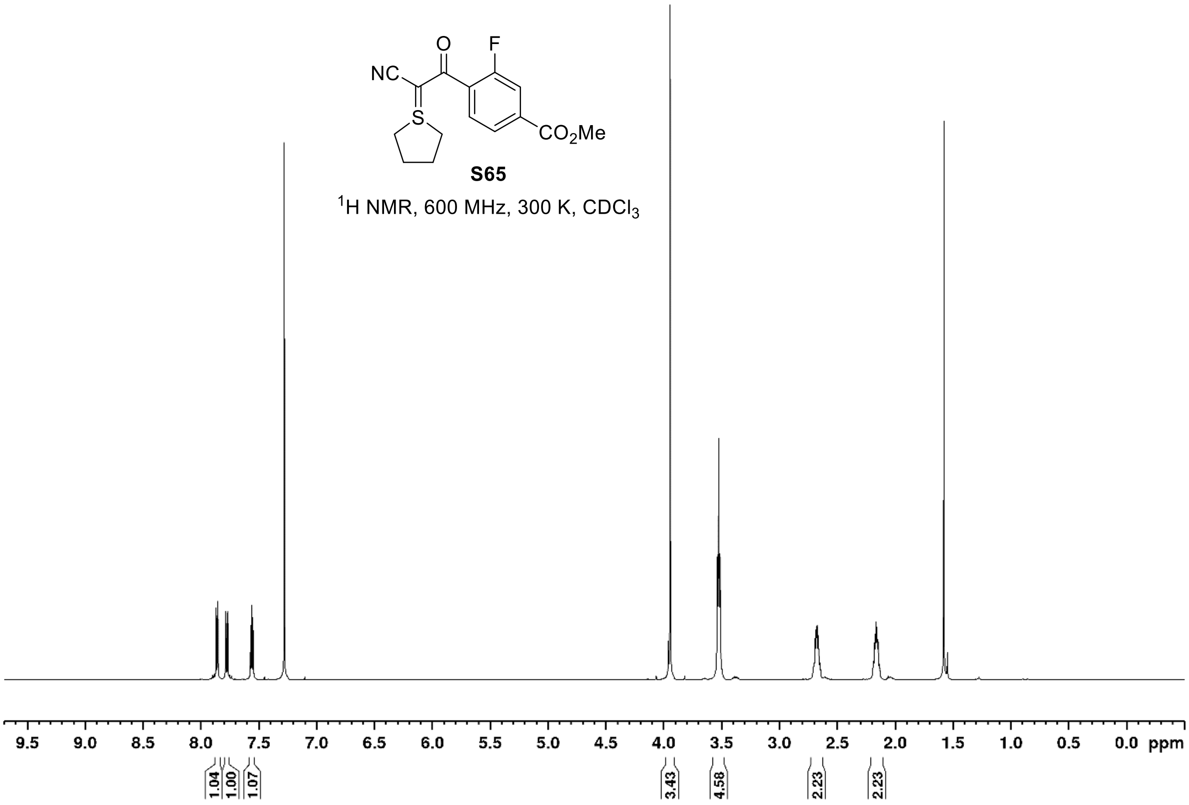
NC
O
F
S
CO2Me
S65
1H NMR, 600 MHz, 300 K, CDCl3
9.5 9.0 8.5 8.0 7.5 7.0 6.5 6.0 5.5 5.0 4.5 4.0 3.5 3.0 2.5 2.0 1.5 1.0 0.5 0.0 ppm
1.04 1.00 1.07 3.43 4.58 2.23 2.23

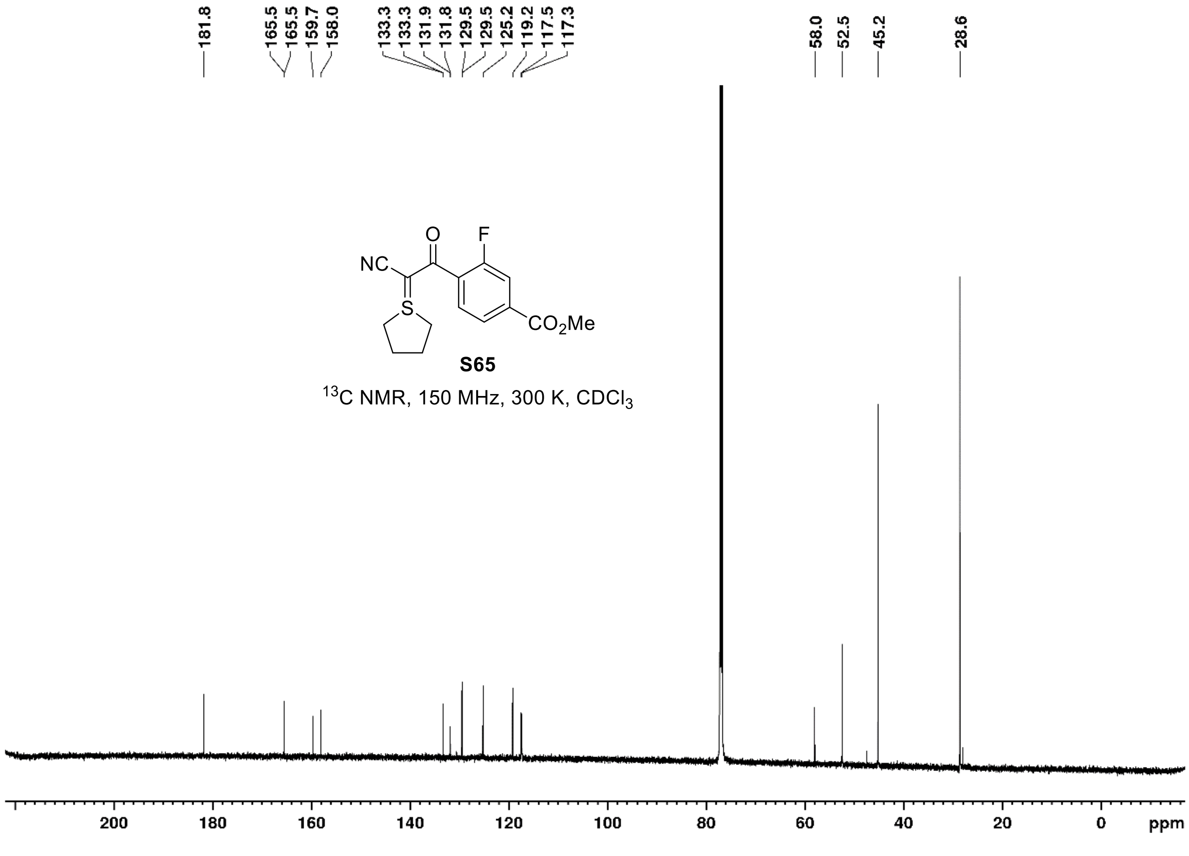
181.8
165.5 165.5 159.7 158.0
133.3 133.3 131.9 131.8 129.5 129.5 125.2 119.2 117.5 117.3
58.0 52.5 45.2 28.6
NC
O
F
S
CO2Me
S65
13C NMR, 150 MHz, 300 K, CDCl3
200 180 160 140 120 100 80 60 40 20 0 ppm

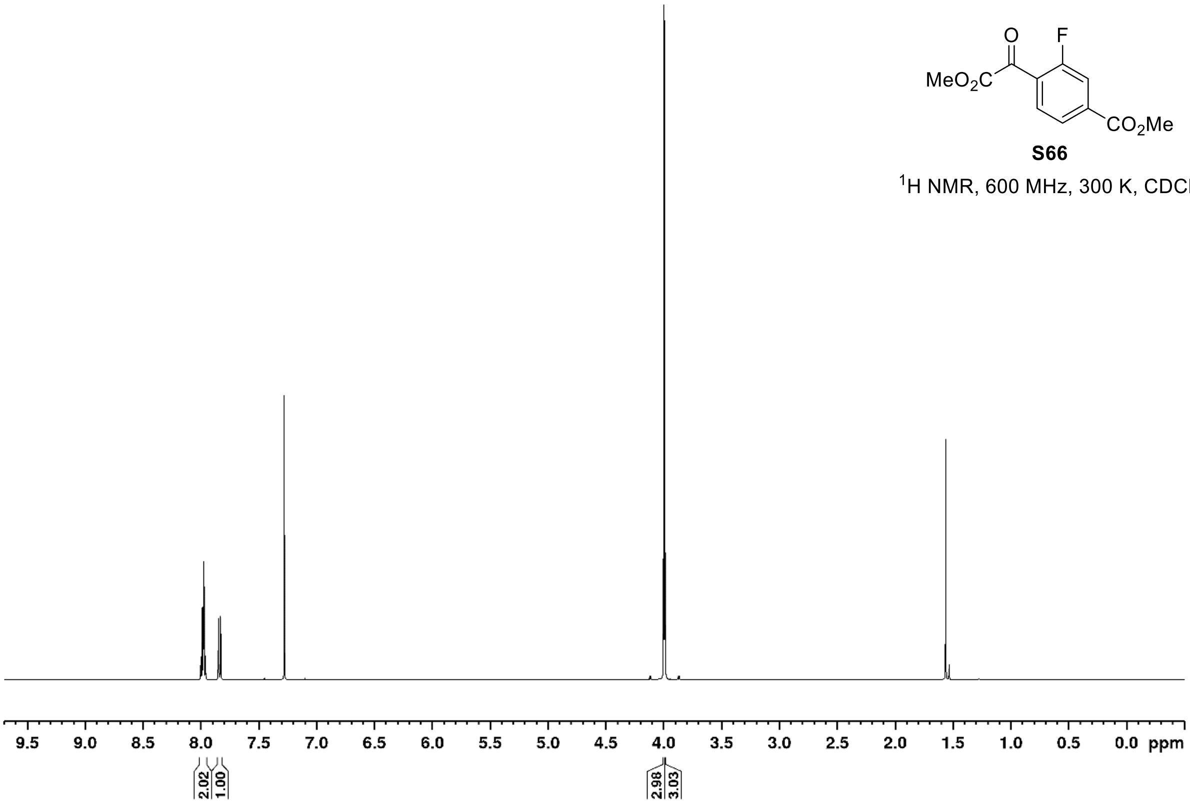

S66

^{1}H NMR, 600 MHz, 300 K, $CDCl_3$

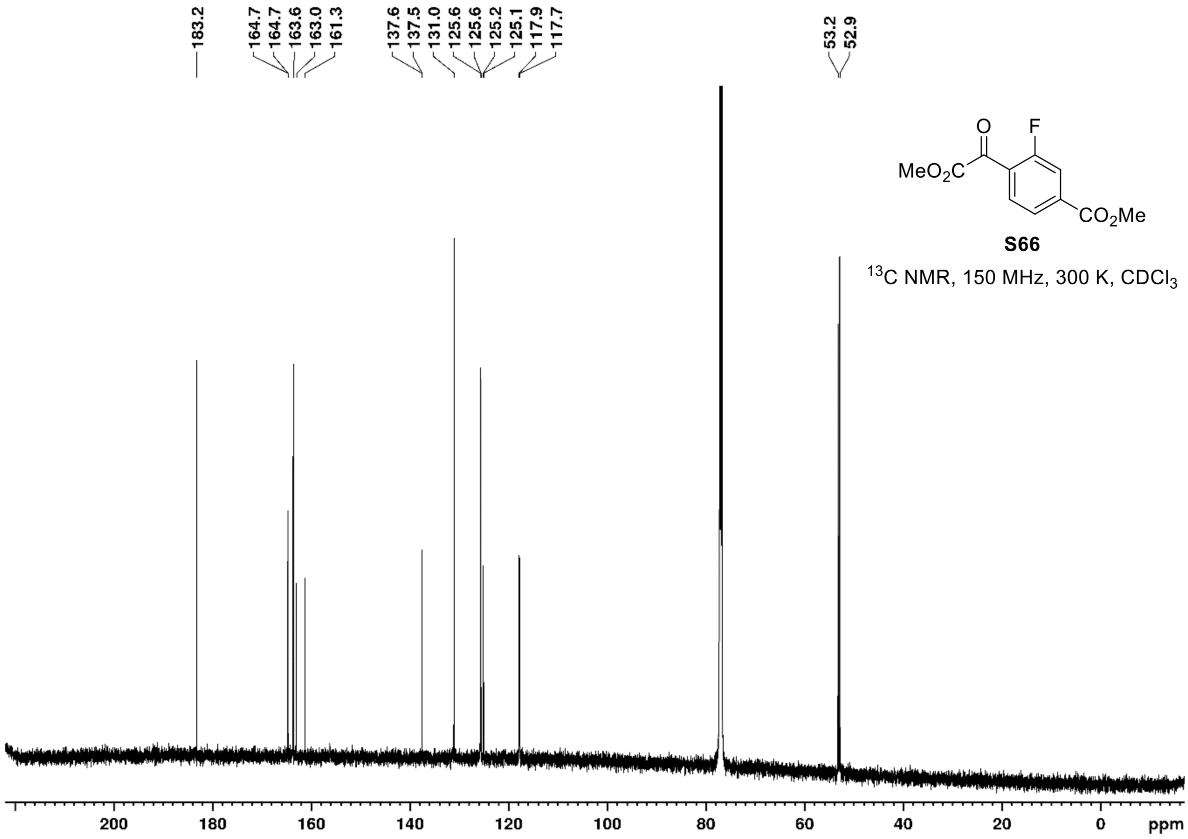

S66

^{13}C NMR, 150 MHz, 300 K, $CDCl_3$

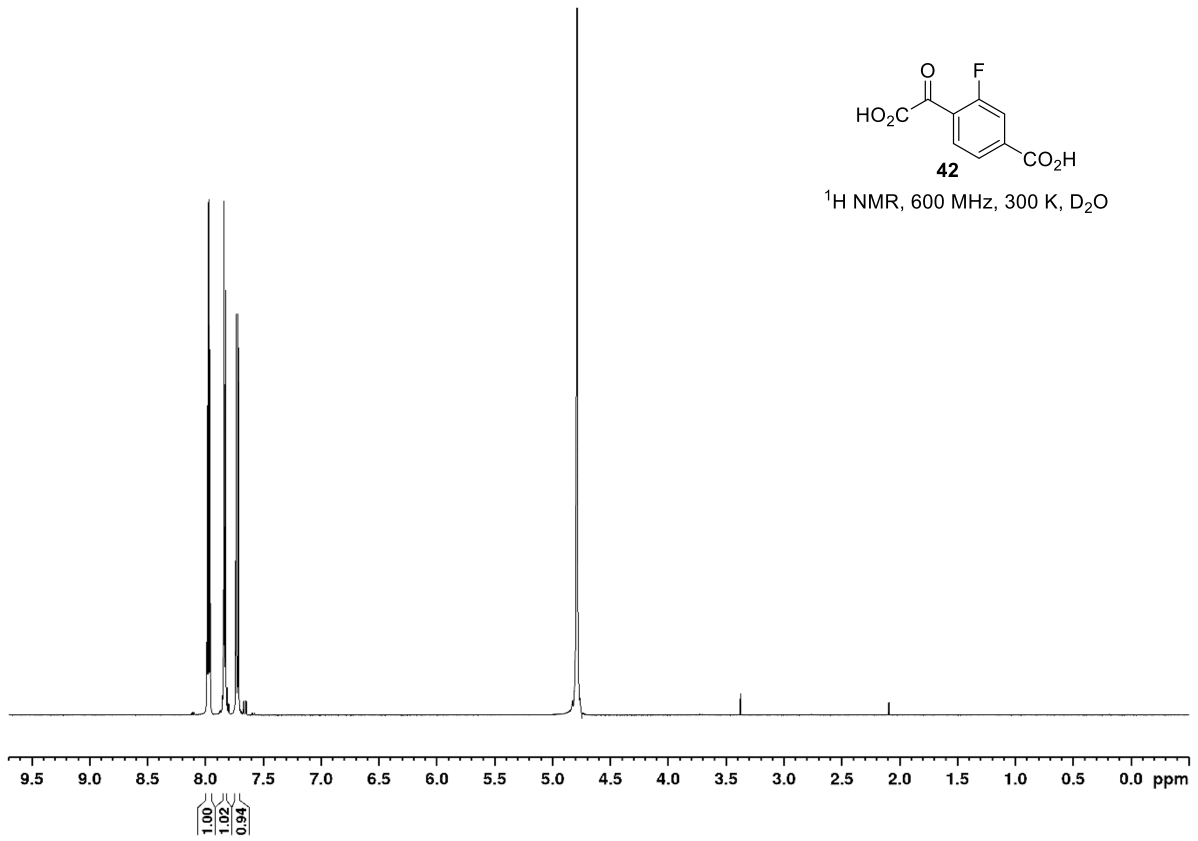
HO2C
O
F
CO2H
42
1H NMR, 600 MHz, 300 K, D2O
9.5 9.0 8.5 8.0 7.5 7.0 6.5 6.0 5.5 5.0 4.5 4.0 3.5 3.0 2.5 2.0 1.5 1.0 0.5 0.0 ppm
1.00 1.02 0.94

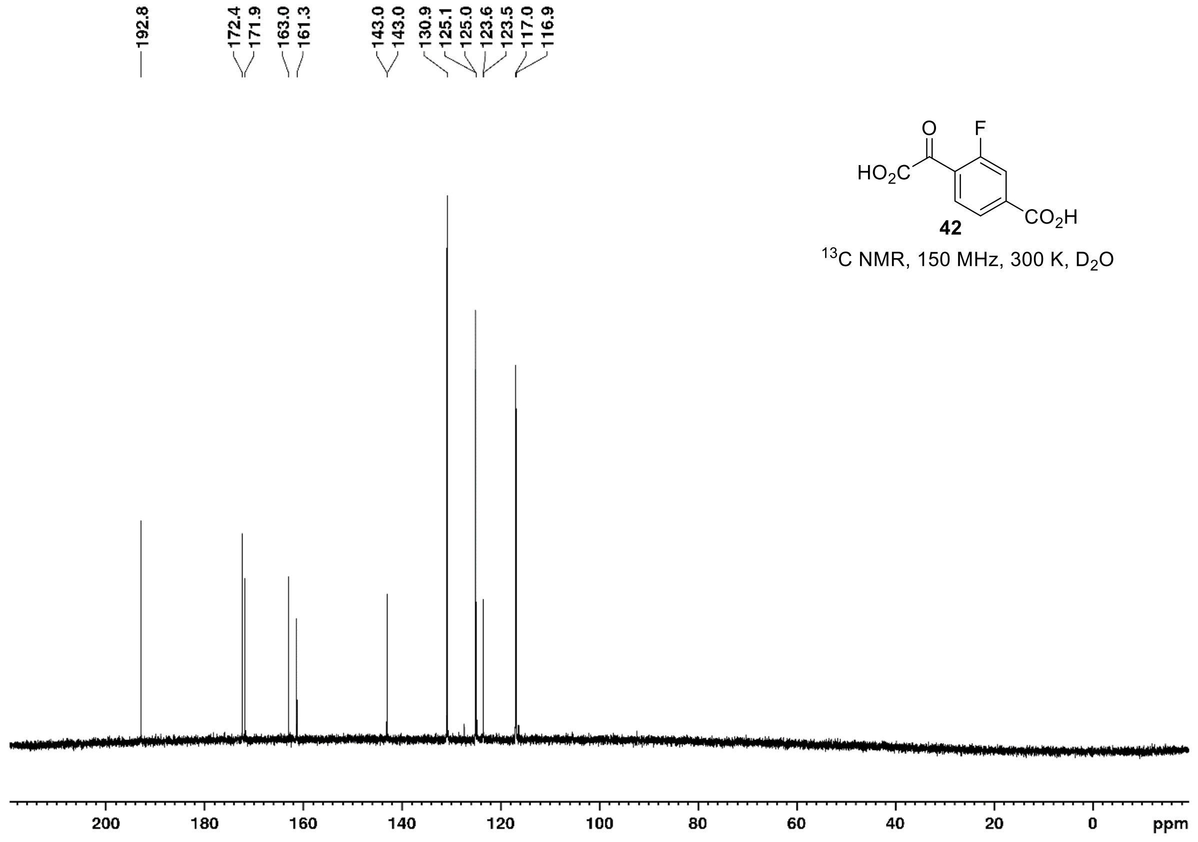
192.8
172.4 171.9 163.0 161.3
143.0 143.0 130.9 125.1 125.0 123.6 123.5 117.0 116.9
HO2C
O
F
CO2H
42
13C NMR, 150 MHz, 300 K, D2O
200 180 160 140 120 100 80 60 40 20 0 ppm

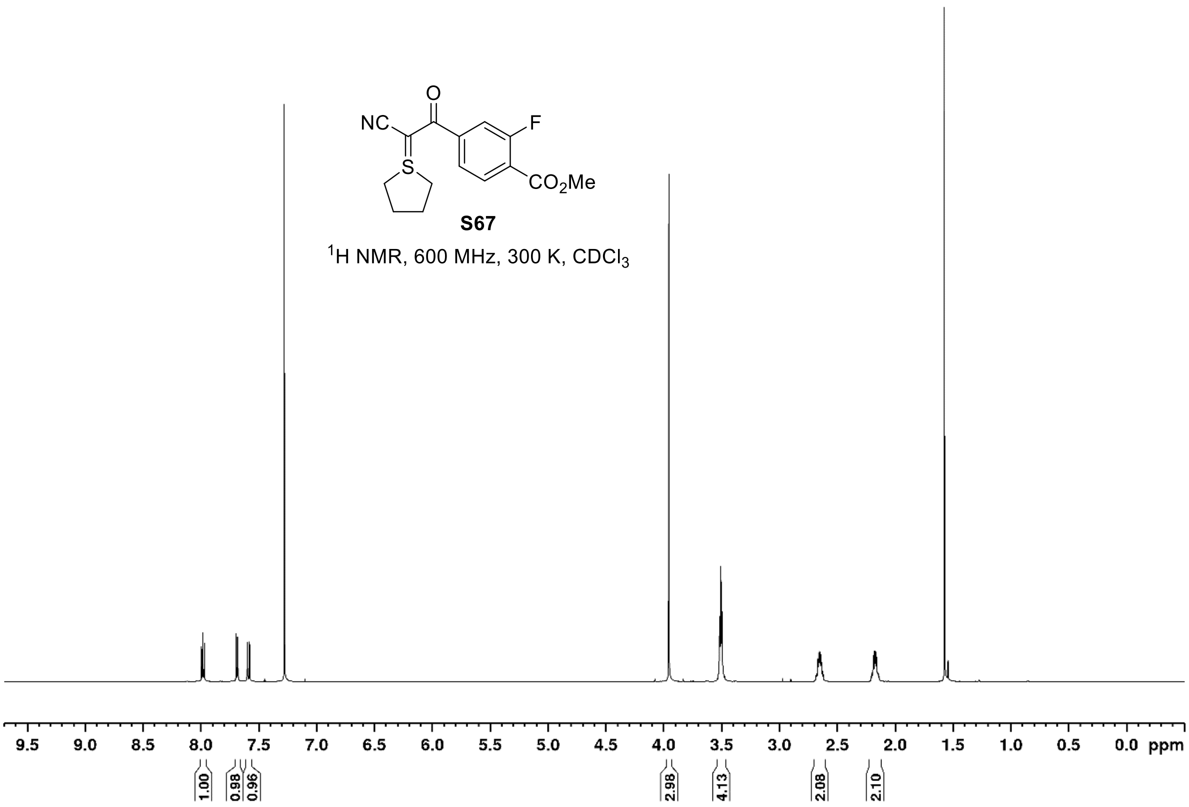
NC
O
F
S
CO2Me
S67
1H NMR, 600 MHz, 300 K, CDCl3
9.5 9.0 8.5 8.0 7.5 7.0 6.5 6.0 5.5 5.0 4.5 4.0 3.5 3.0 2.5 2.0 1.5 1.0 0.5 0.0 ppm
1.00 0.98 0.96 2.98 4.13 2.08 2.10

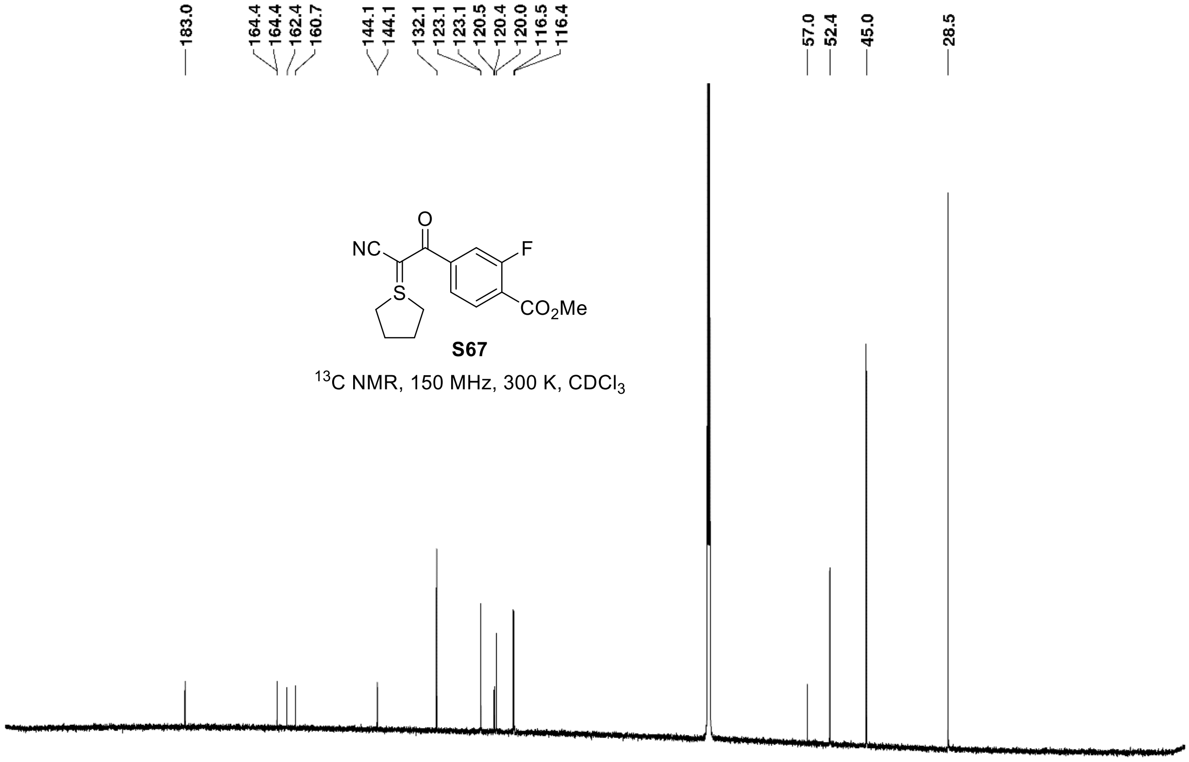
183.0
164.4 164.4 162.4 160.7
144.1 144.1
132.1 123.1 123.1 120.5 120.4 120.0 116.5 116.4
57.0 52.4 45.0
28.5
NC
O
F
S
CO2Me
S67
13C NMR, 150 MHz, 300 K, CDCl3
200 180 160 140 120 100 80 60 40 20 0 ppm

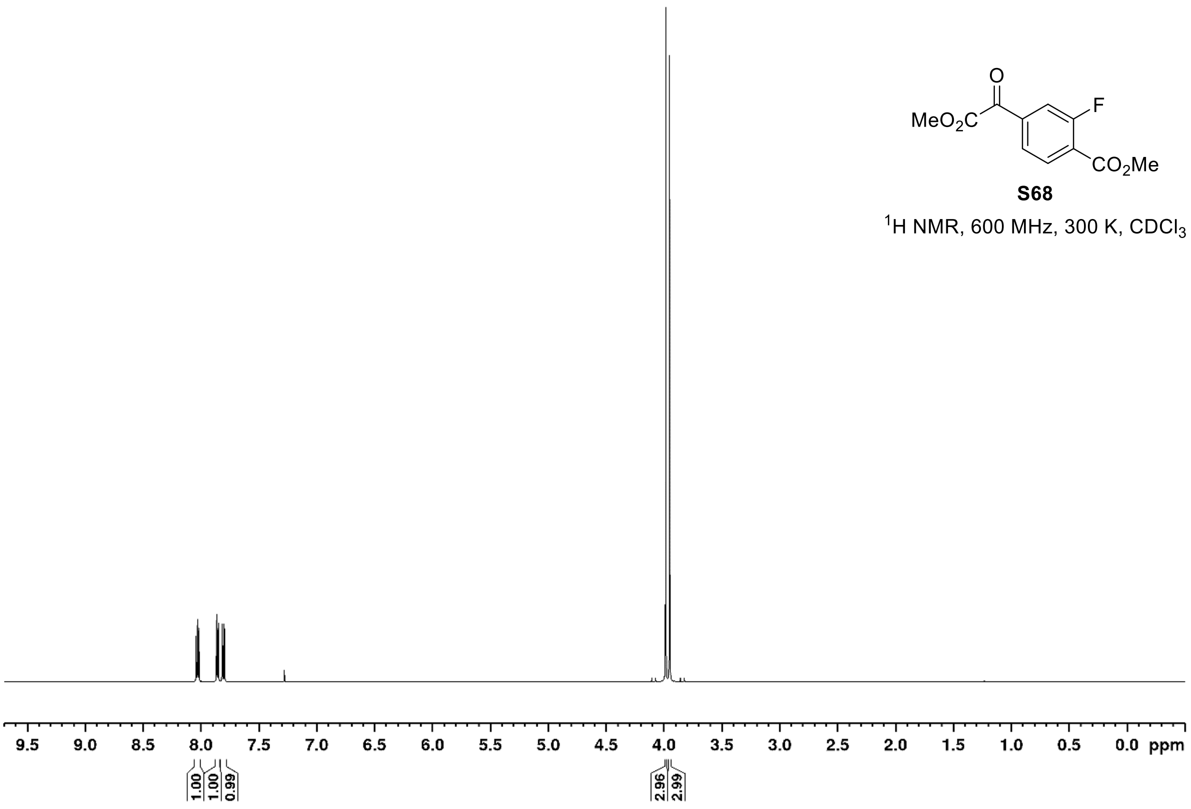
MeO2C
O
F
CO2Me
S68
1H NMR, 600 MHz, 300 K, CDCl3
9.5 9.0 8.5 8.0 7.5 7.0 6.5 6.0 5.5 5.0 4.5 4.0 3.5 3.0 2.5 2.0 1.5 1.0 0.5 0.0 ppm
1.00 1.00 0.99
2.96 2.99

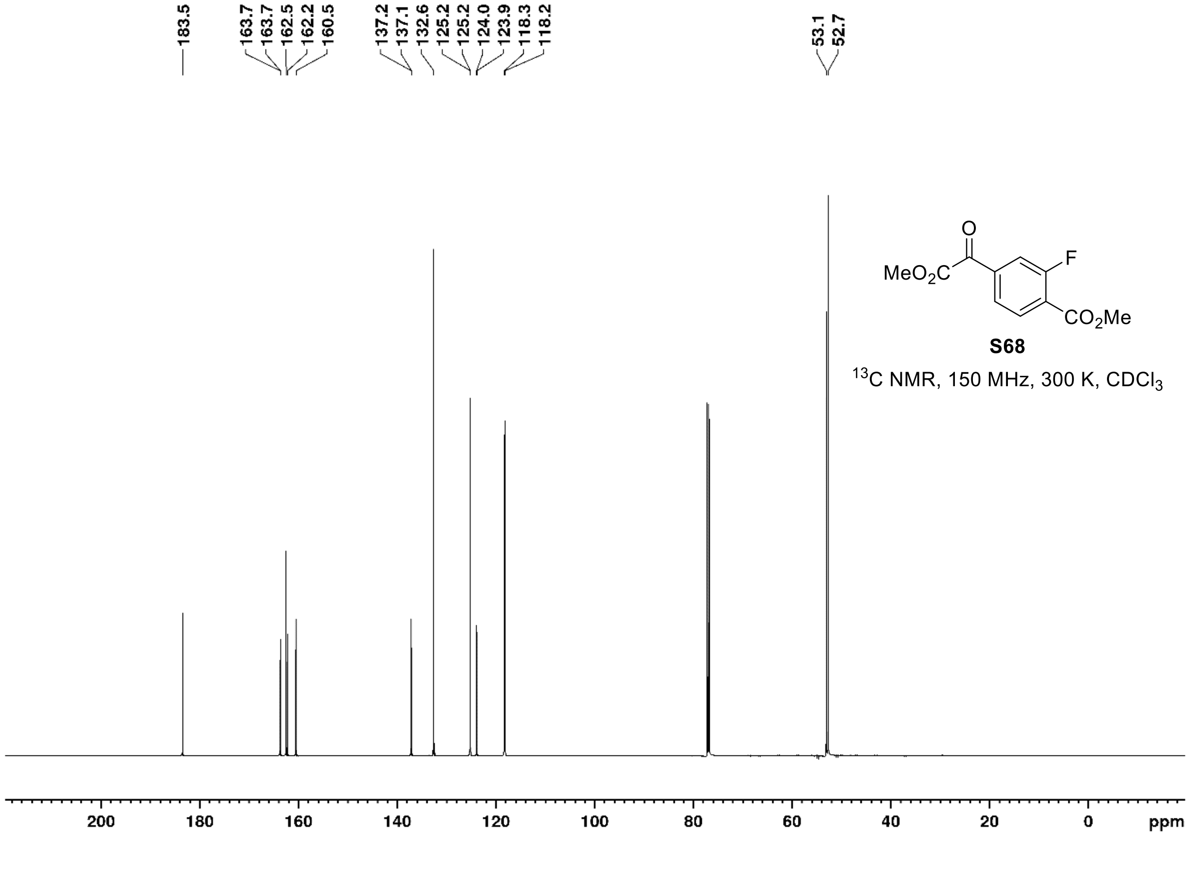
183.5
163.7 163.7 162.5 162.2 160.5
137.2 137.1 132.6 125.2 125.2 124.0 123.9 118.3 118.2
53.1 52.7
MeO2C
O
F
CO2Me
S68
13C NMR, 150 MHz, 300 K, CDCl3
200 180 160 140 120 100 80 60 40 20 0 ppm

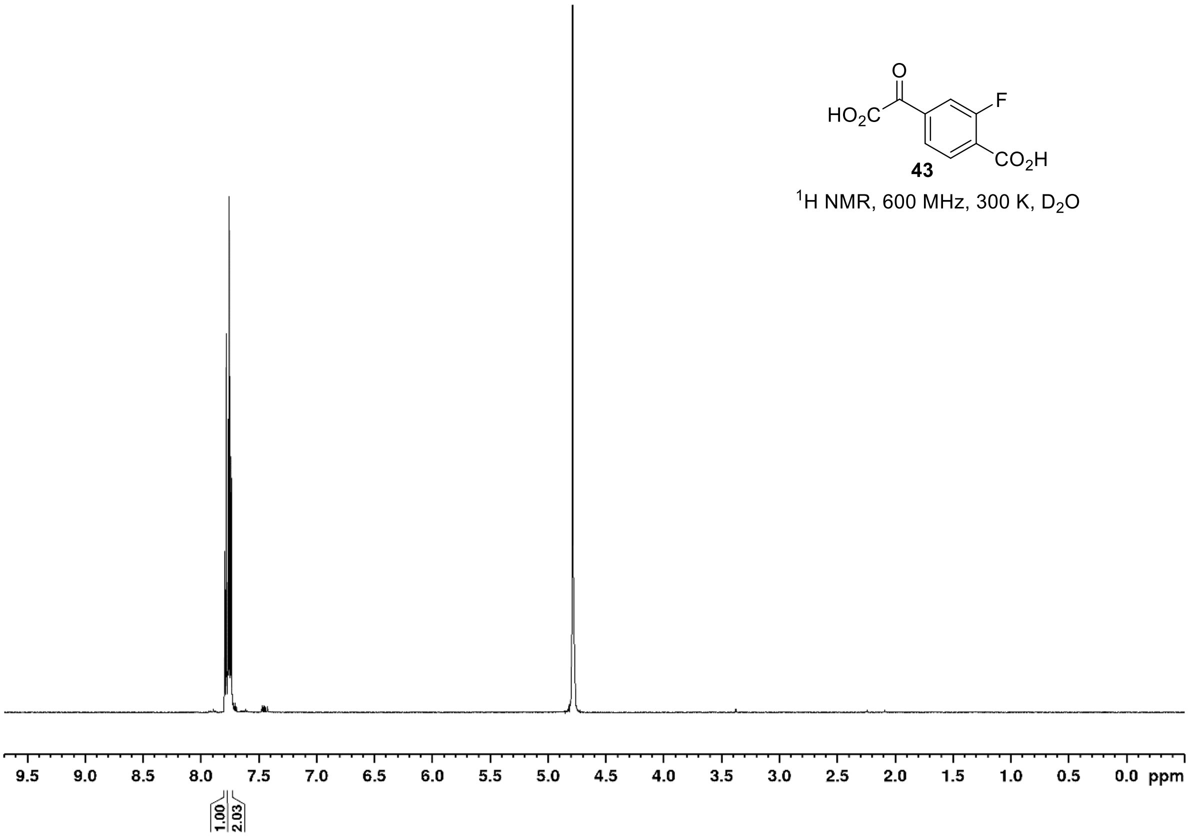
O
HO2C
F
CO2H
43
1H NMR, 600 MHz, 300 K, D2O
9.5 9.0 8.5 8.0 7.5 7.0 6.5 6.0 5.5 5.0 4.5 4.0 3.5 3.0 2.5 2.0 1.5 1.0 0.5 0.0 ppm
1.00
2.03

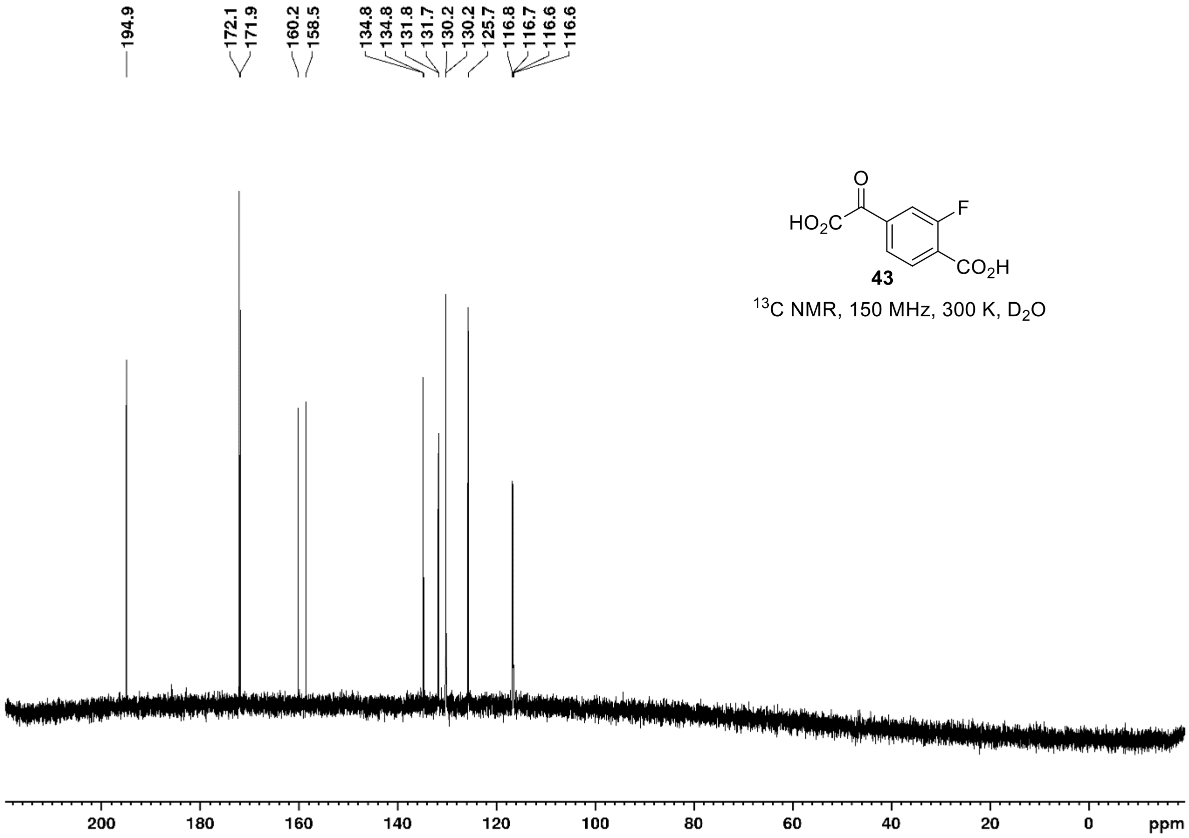
194.9
172.1
171.9
160.2
158.5
134.8
134.8
131.8
131.7
130.2
130.2
125.7
116.8
116.7
116.6
116.6
O
HO2C
F
CO2H
43
13C NMR, 150 MHz, 300 K, D2O
200 180 160 140 120 100 80 60 40 20 0 ppm

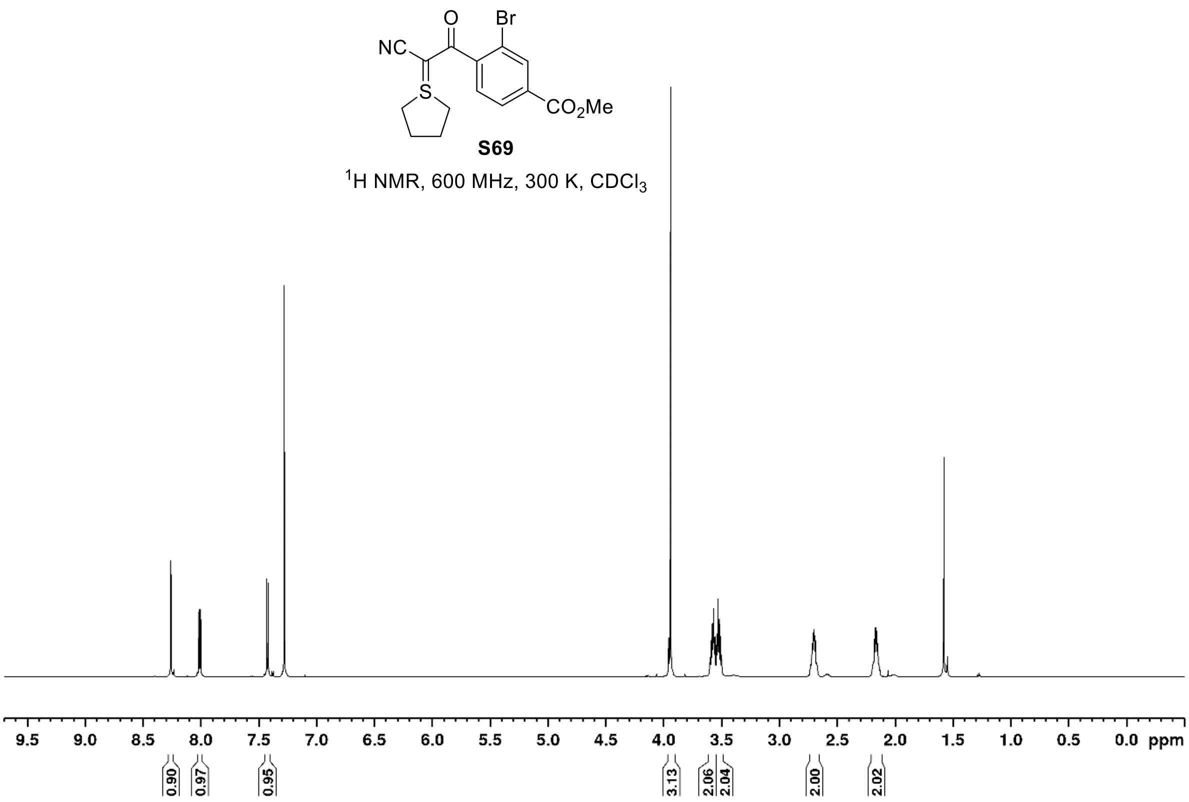
NC
O
Br
S
CO2Me
S69
1H NMR, 600 MHz, 300 K, CDCl3
9.5 9.0 8.5 8.0 7.5 7.0 6.5 6.0 5.5 5.0 4.5 4.0 3.5 3.0 2.5 2.0 1.5 1.0 0.5 0.0 ppm
0.90 0.97 0.95 3.13 2.06 2.04 2.00 2.02

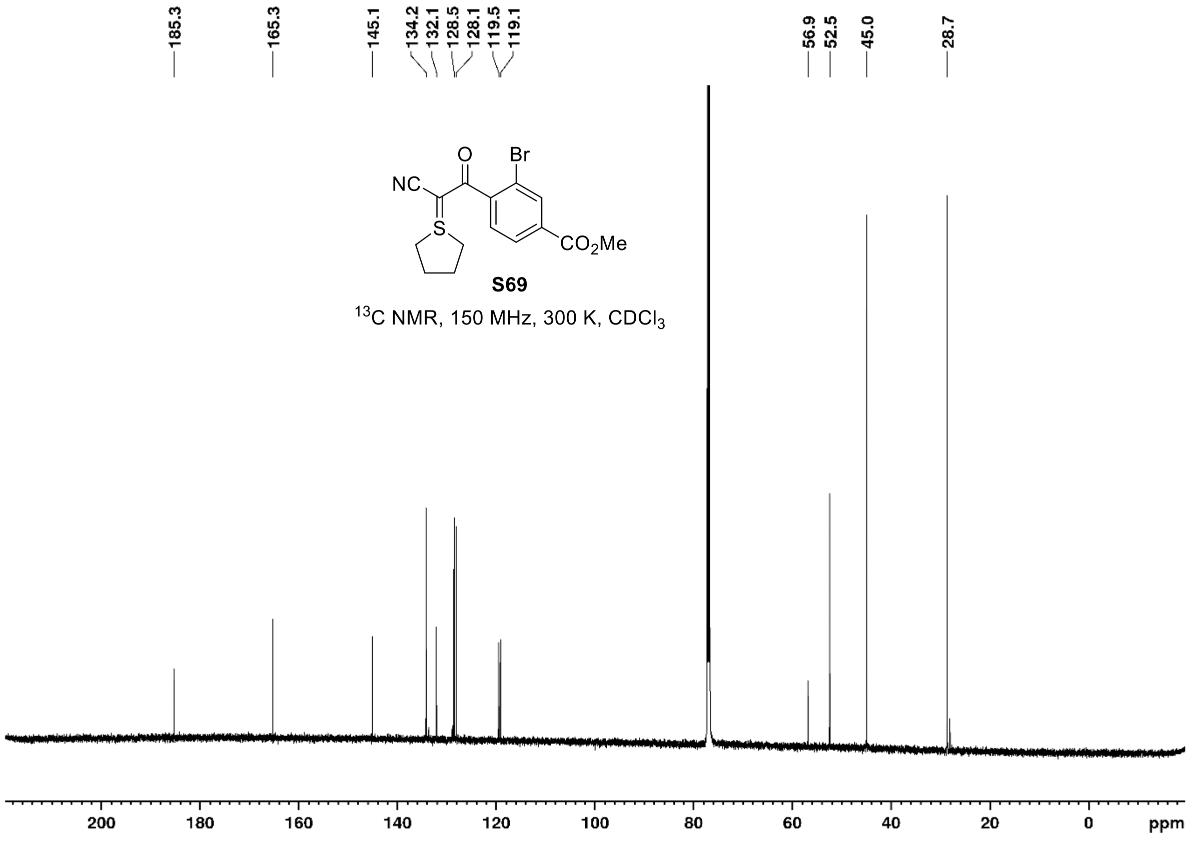
185.3
165.3
145.1
134.2
132.1
128.5
128.1
119.5
119.1
56.9
52.5
45.0
28.7
NC
O
Br
S
CO2Me
S69
13C NMR, 150 MHz, 300 K, CDCl3
200 180 160 140 120 100 80 60 40 20 0 ppm

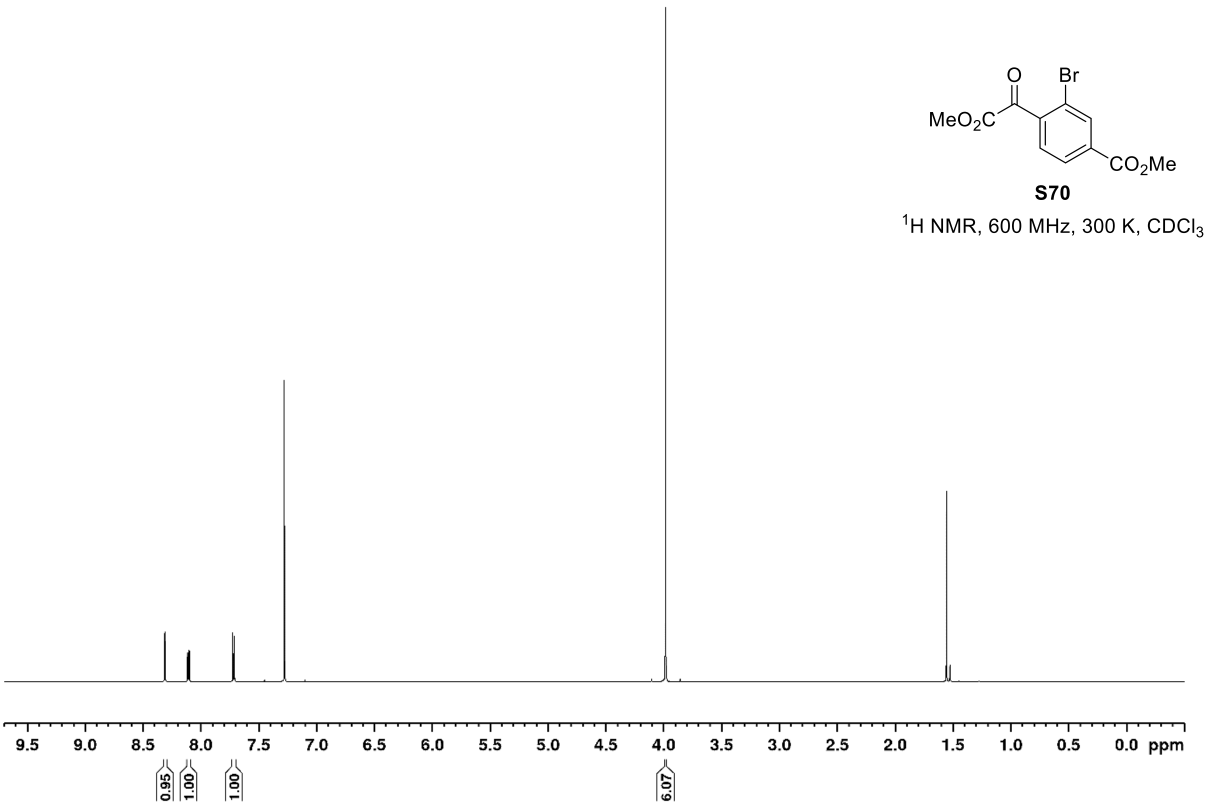

MeO2C
O
Br
CO2Me
S70
1H NMR, 600 MHz, 300 K, CDCl3
9.5 9.0 8.5 8.0 7.5 7.0 6.5 6.0 5.5 5.0 4.5 4.0 3.5 3.0 2.5 2.0 1.5 1.0 0.5 0.0 ppm
0.95
1.00
1.00
6.07

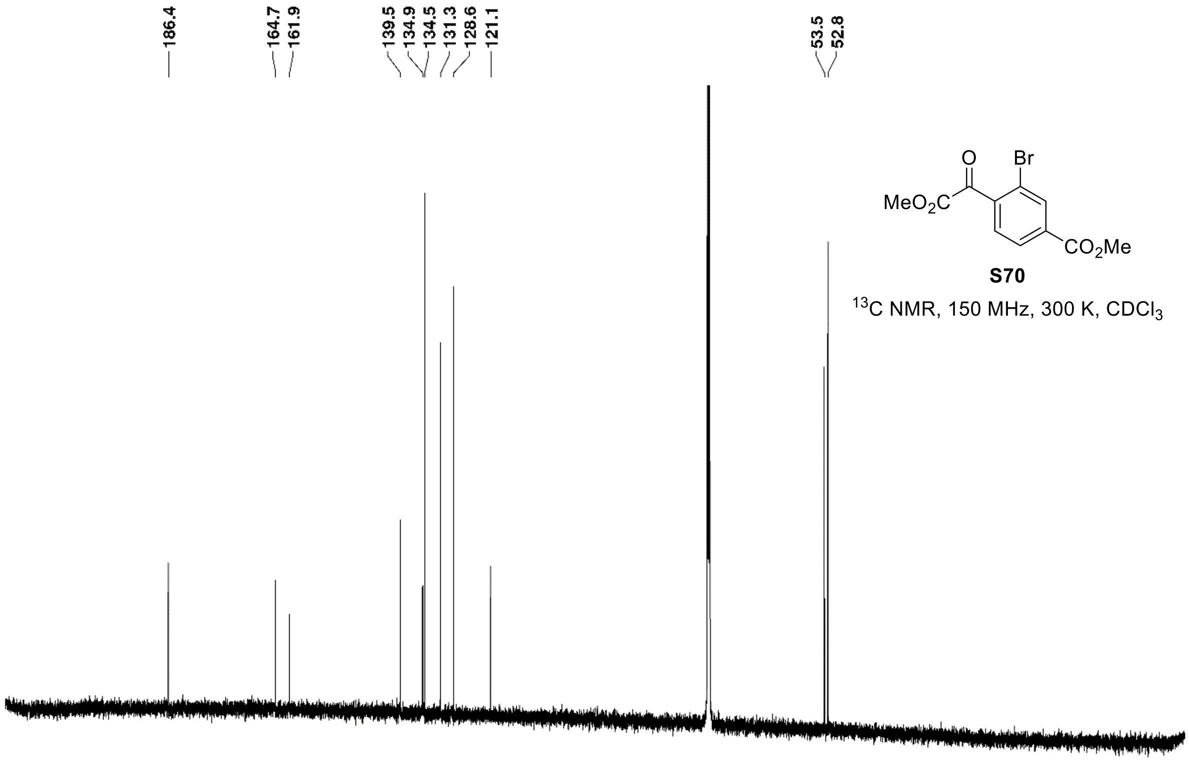

186.4
164.7
161.9
139.5
134.9
134.5
131.3
128.6
121.1
53.5
52.8
MeO2C
O
Br
CO2Me
S70
13C NMR, 150 MHz, 300 K, CDCl3
200 180 160 140 120 100 80 60 40 20 0 ppm

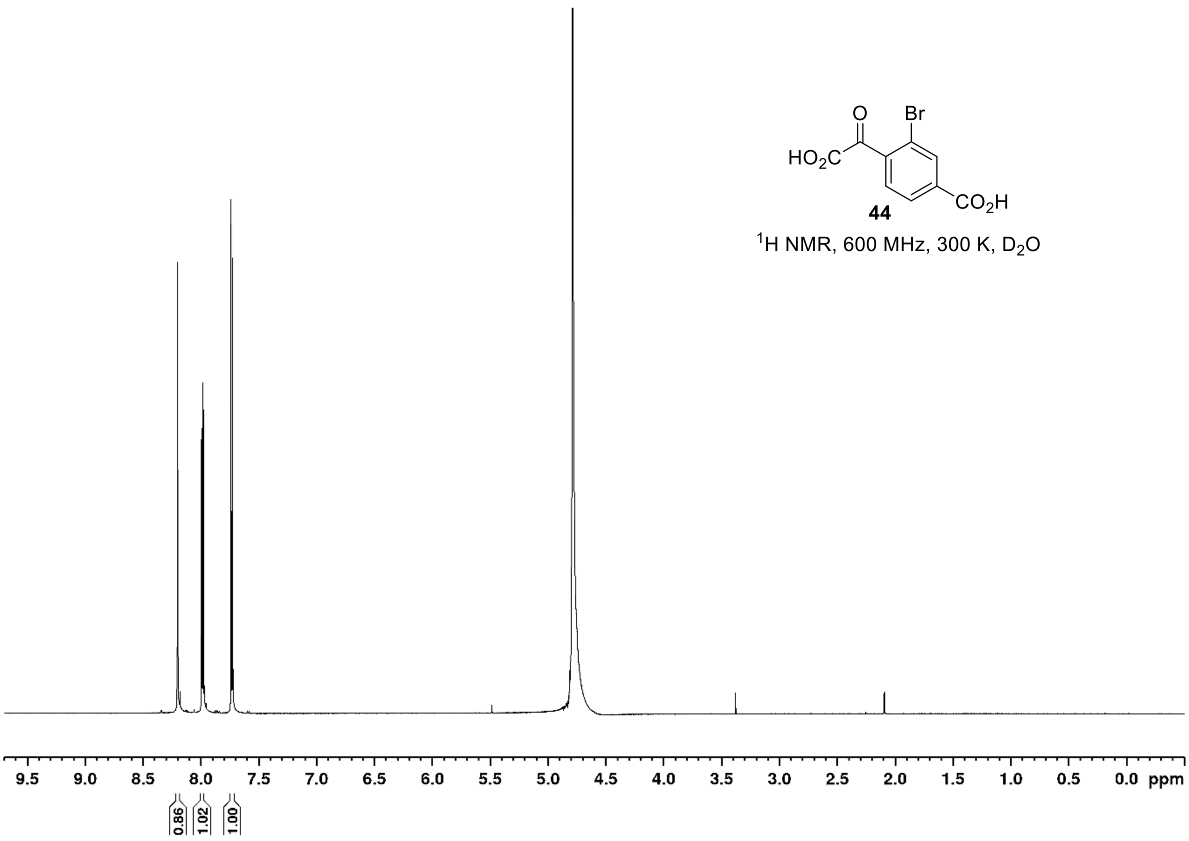
O
Br
HO2C
CO2H
44
1H NMR, 600 MHz, 300 K, D2O
9.5 9.0 8.5 8.0 7.5 7.0 6.5 6.0 5.5 5.0 4.5 4.0 3.5 3.0 2.5 2.0 1.5 1.0 0.5 0.0 ppm
0.86
1.02
1.00

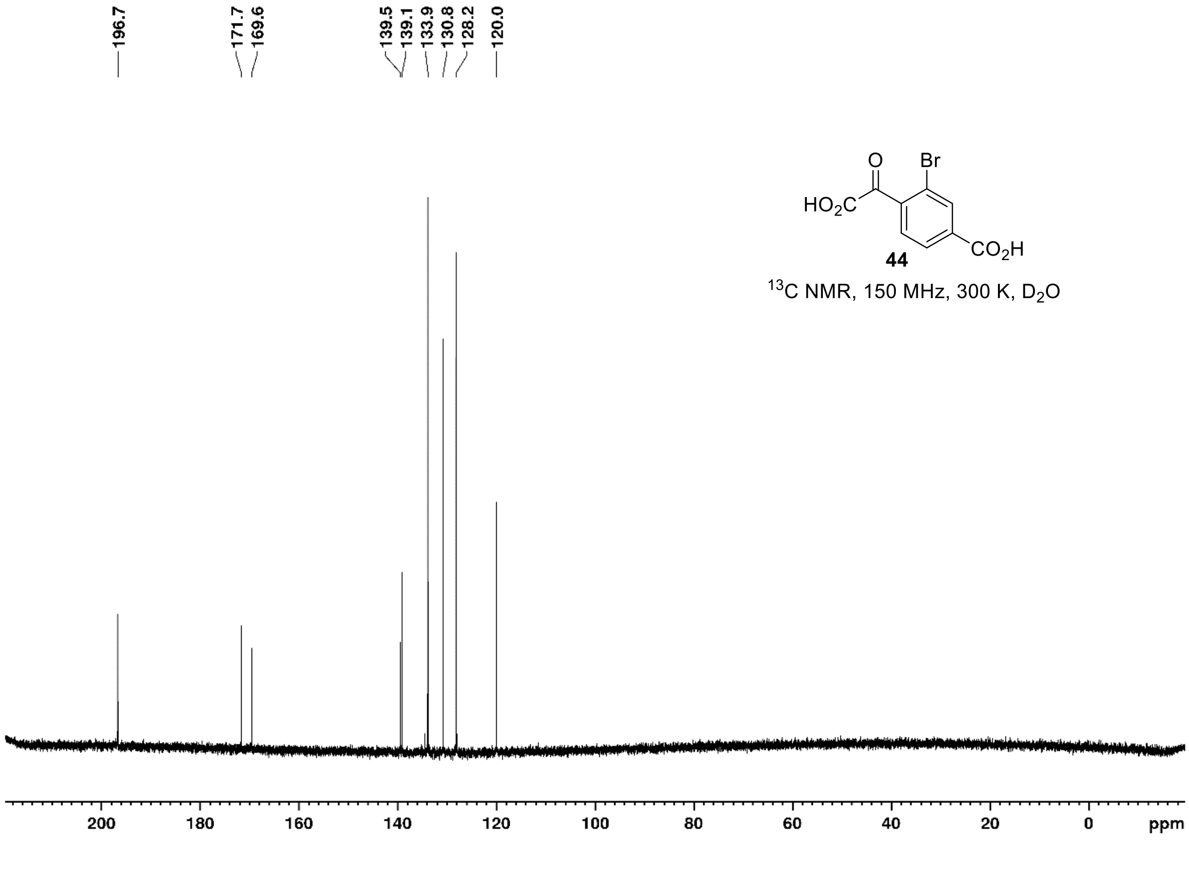
196.7
171.7
169.6
139.5
139.1
133.9
130.8
128.2
120.0
O
Br
HO2C
CO2H
44
13C NMR, 150 MHz, 300 K, D2O
200 180 160 140 120 100 80 60 40 20 0 ppm

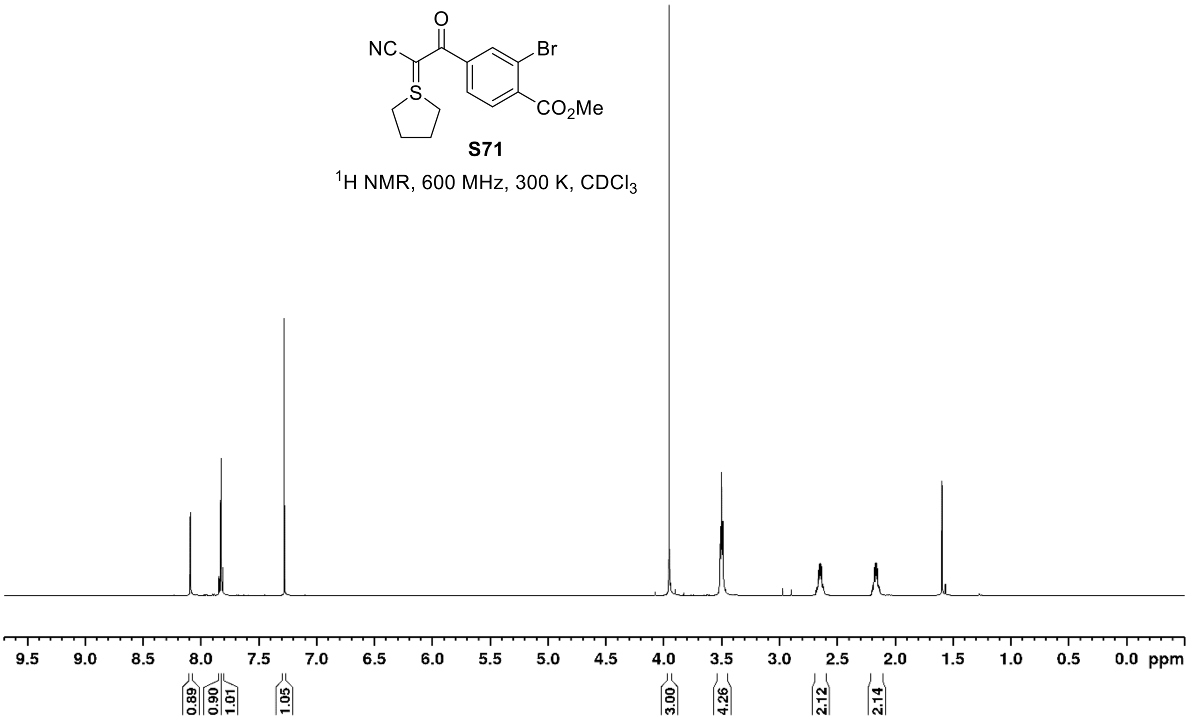
NC
O
Br
S
CO2Me
S71
1H NMR, 600 MHz, 300 K, CDCl3
9.5 9.0 8.5 8.0 7.5 7.0 6.5 6.0 5.5 5.0 4.5 4.0 3.5 3.0 2.5 2.0 1.5 1.0 0.5 0.0 ppm
0.89 0.90 1.01 1.05 3.00 4.26 2.12 2.14

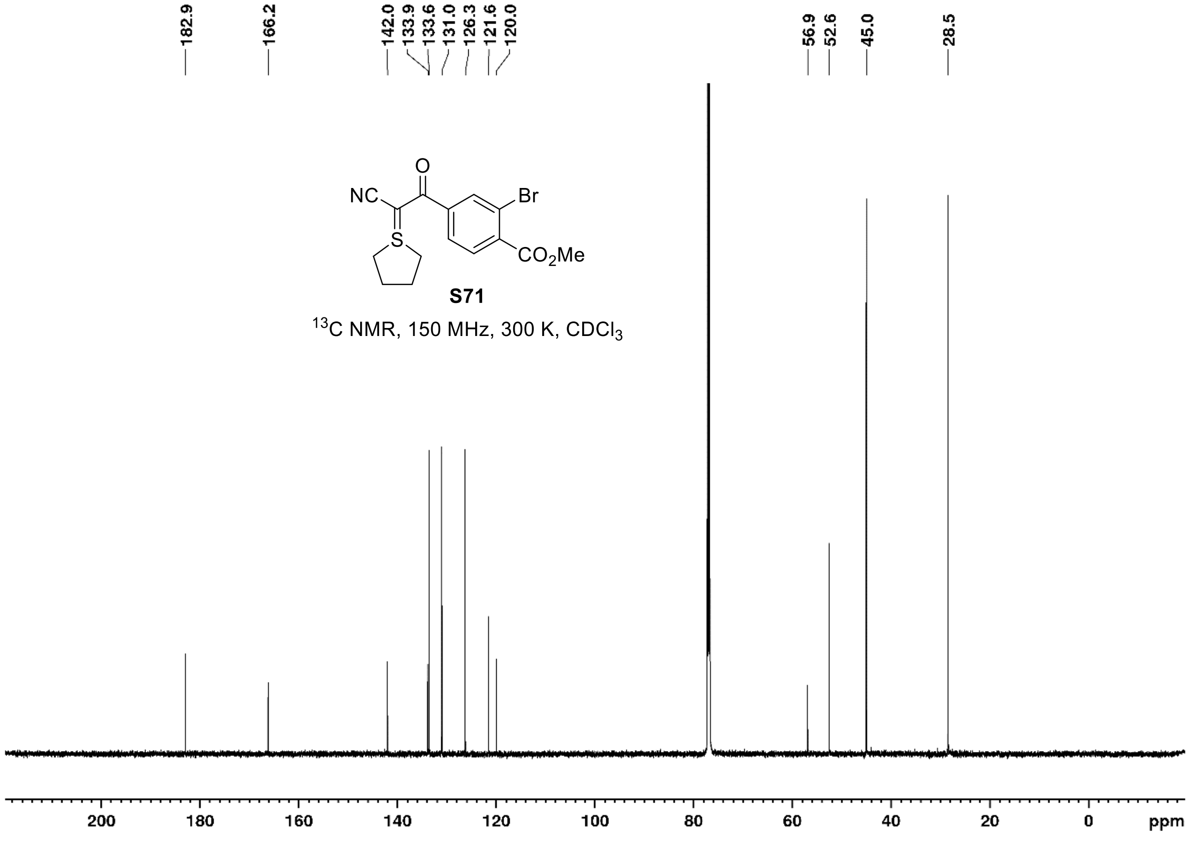
182.9
166.2
142.0
133.9
133.6
131.0
126.3
121.6
120.0
56.9
52.6
45.0
28.5
NC
O
Br
S
CO2Me
S71
13C NMR, 150 MHz, 300 K, CDCl3
200 180 160 140 120 100 80 60 40 20 0 ppm

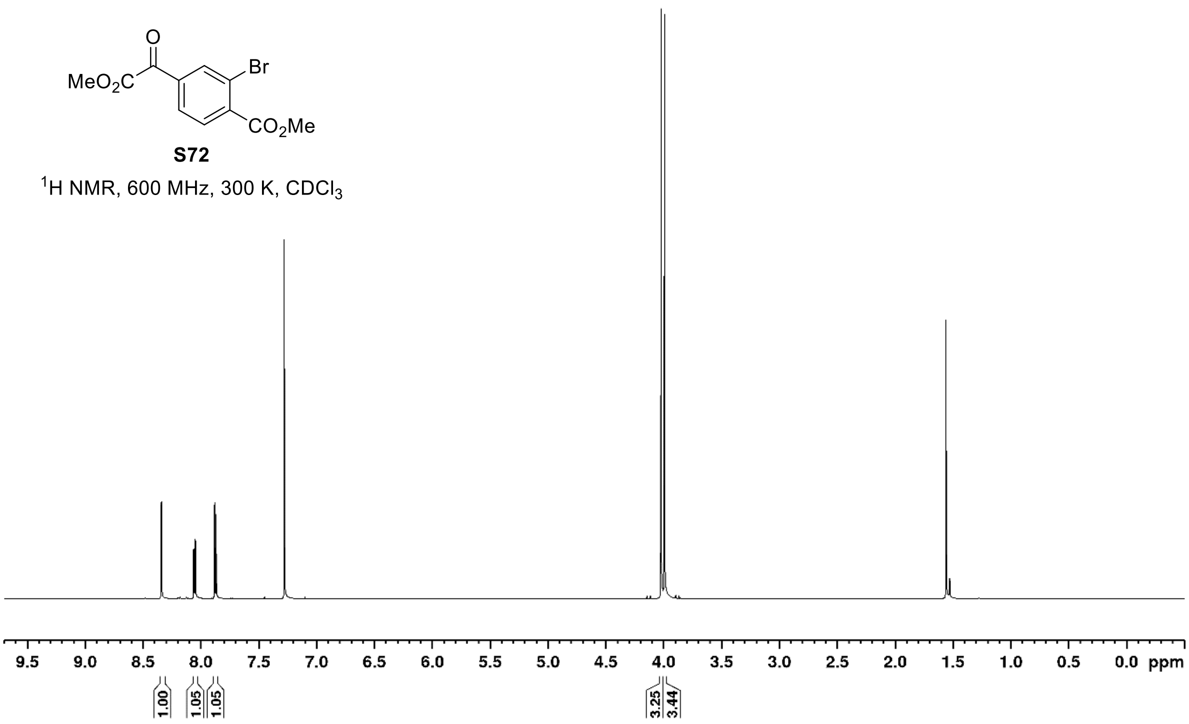
MeO2C
O
Br
CO2Me
S72
1H NMR, 600 MHz, 300 K, CDCl3
9.5 9.0 8.5 8.0 7.5 7.0 6.5 6.0 5.5 5.0 4.5 4.0 3.5 3.0 2.5 2.0 1.5 1.0 0.5 0.0 ppm
1.00
1.05
1.05
3.25
3.44

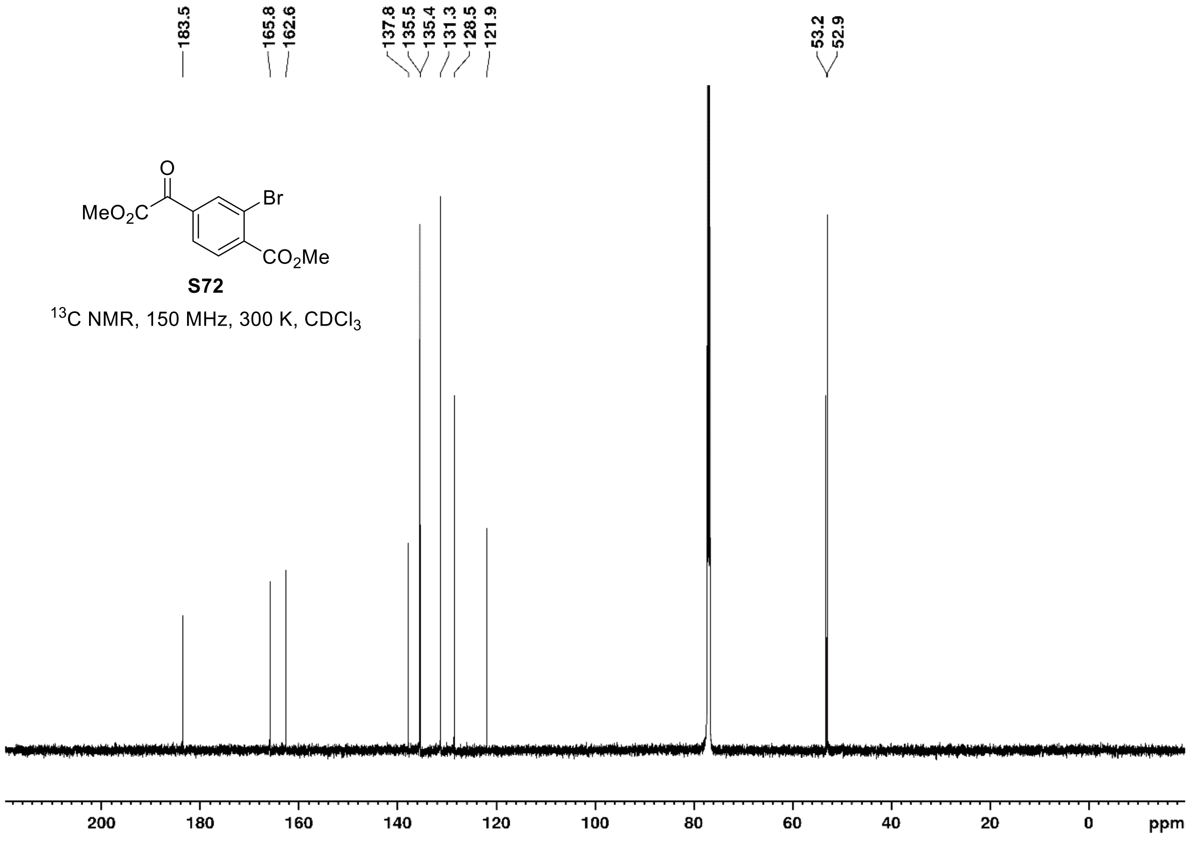
183.5
165.8
162.6
137.8
135.5
135.4
131.3
128.5
121.9
53.2
52.9
MeO2C
O
Br
CO2Me
S72
13C NMR, 150 MHz, 300 K, CDCl3
200 180 160 140 120 100 80 60 40 20 0 ppm

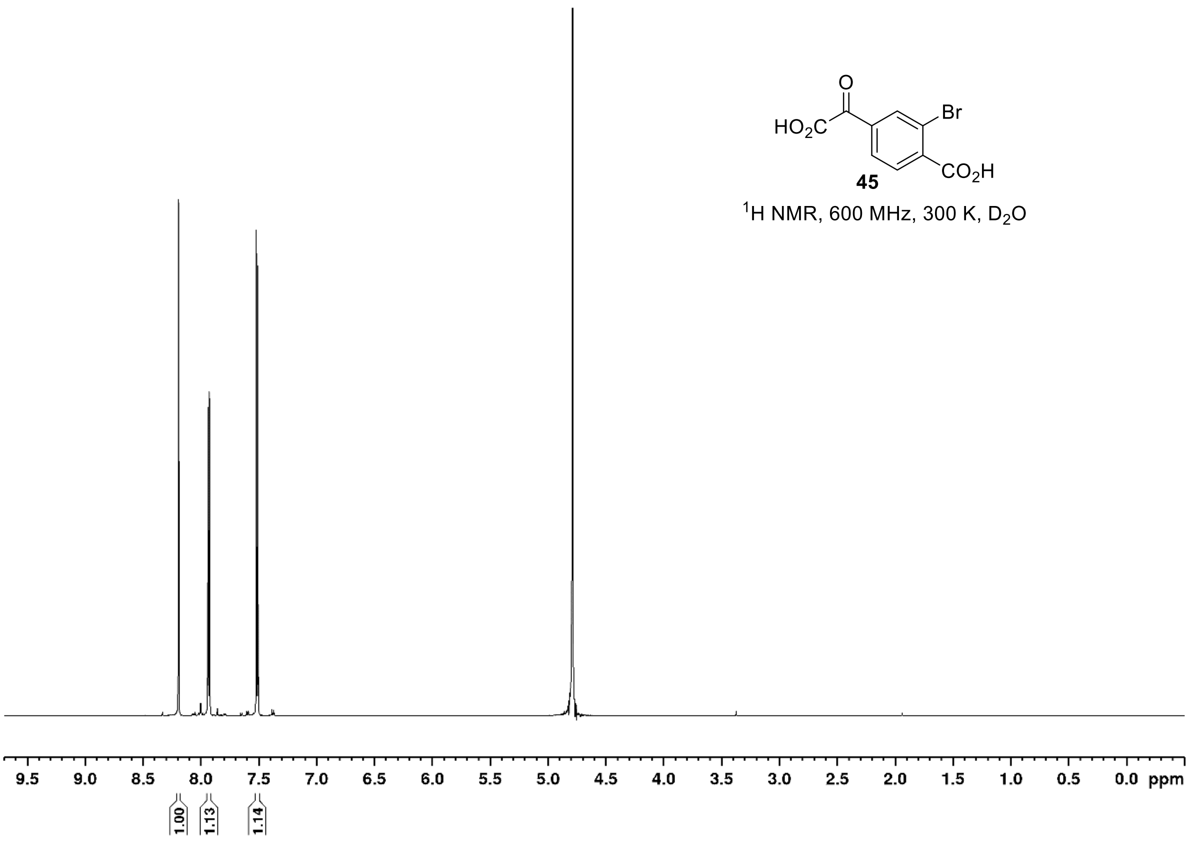
HO2C
O
Br
CO2H
45
1H NMR, 600 MHz, 300 K, D2O
9.5 9.0 8.5 8.0 7.5 7.0 6.5 6.0 5.5 5.0 4.5 4.0 3.5 3.0 2.5 2.0 1.5 1.0 0.5 0.0 ppm
1.00
1.13
1.14

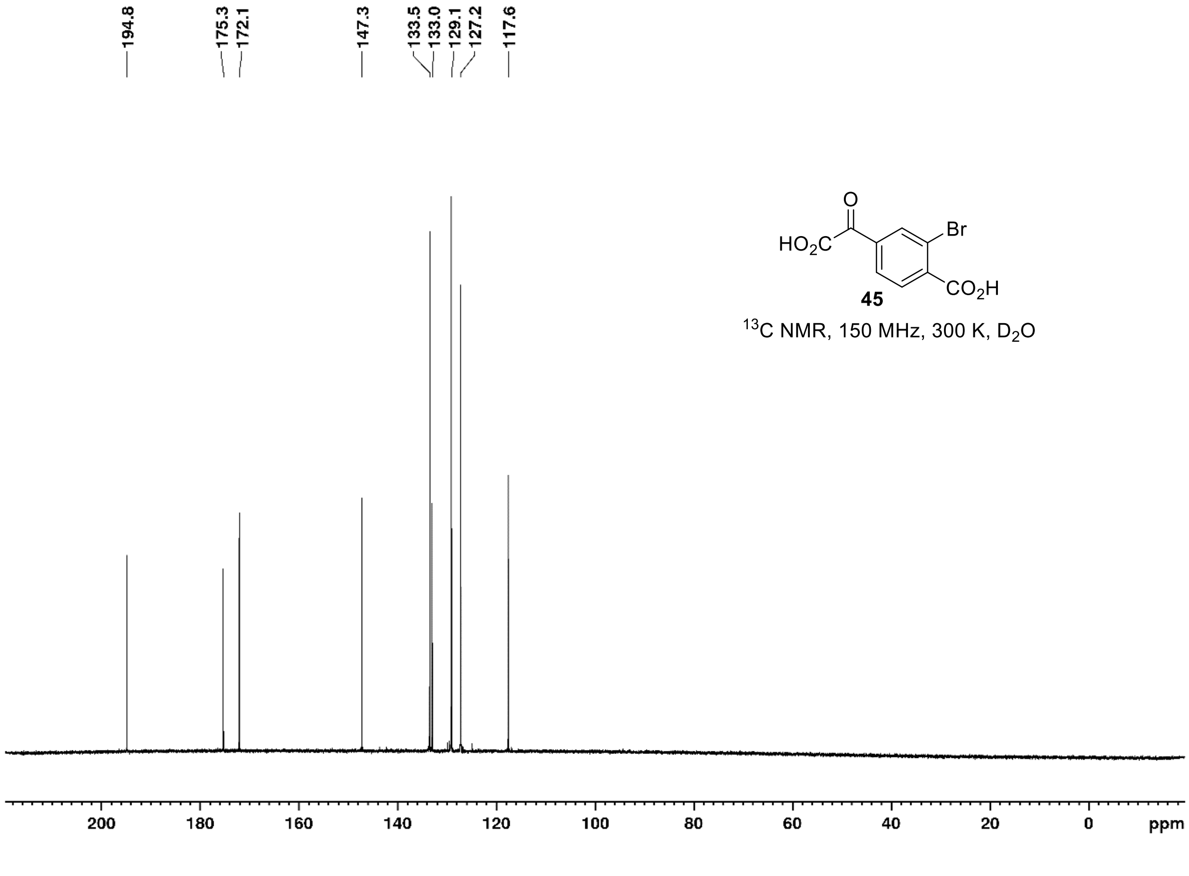
194.8
175.3
172.1
147.3
133.5
133.0
129.1
127.2
117.6
HO2C
O
Br
CO2H
45
13C NMR, 150 MHz, 300 K, D2O
200 180 160 140 120 100 80 60 40 20 0 ppm

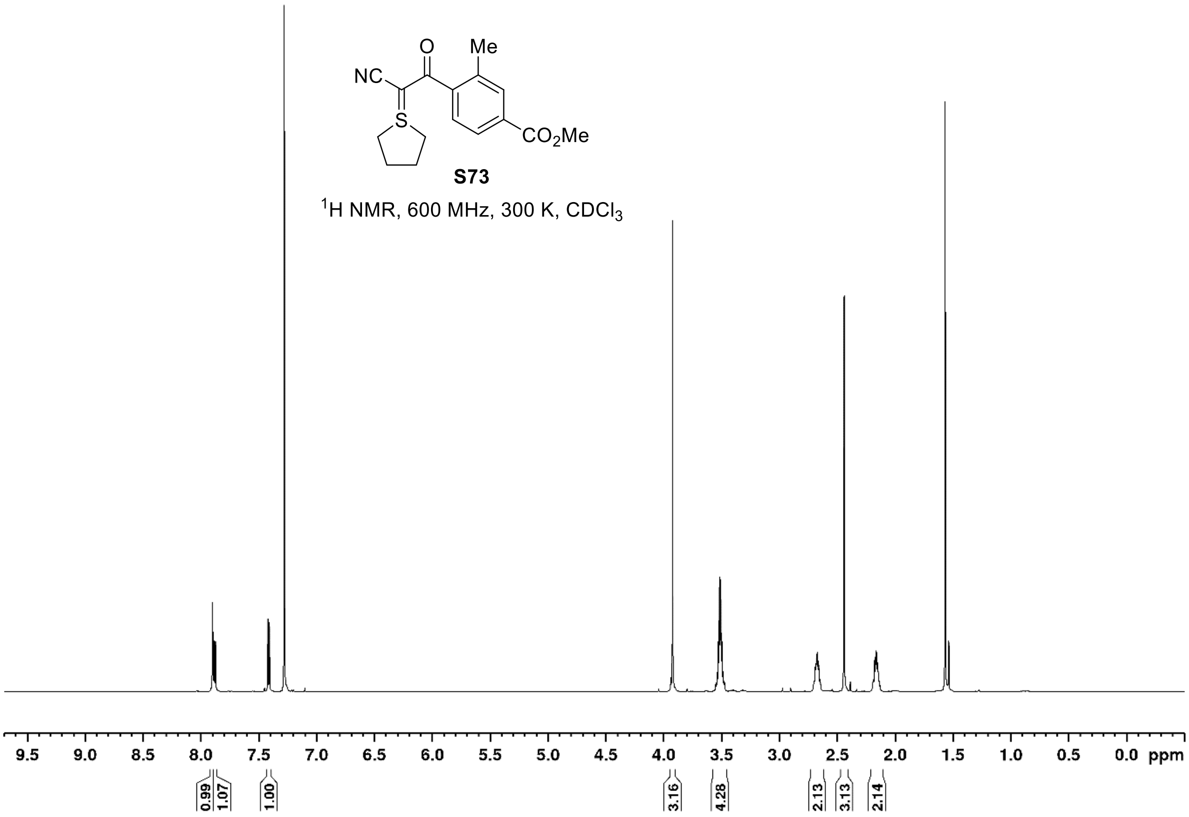
NC
O
Me
S
CO2Me
S73
1H NMR, 600 MHz, 300 K, CDCl3
9.5 9.0 8.5 8.0 7.5 7.0 6.5 6.0 5.5 5.0 4.5 4.0 3.5 3.0 2.5 2.0 1.5 1.0 0.5 0.0 ppm
0.99 1.07 1.00 3.16 4.28 2.13 3.13 2.14

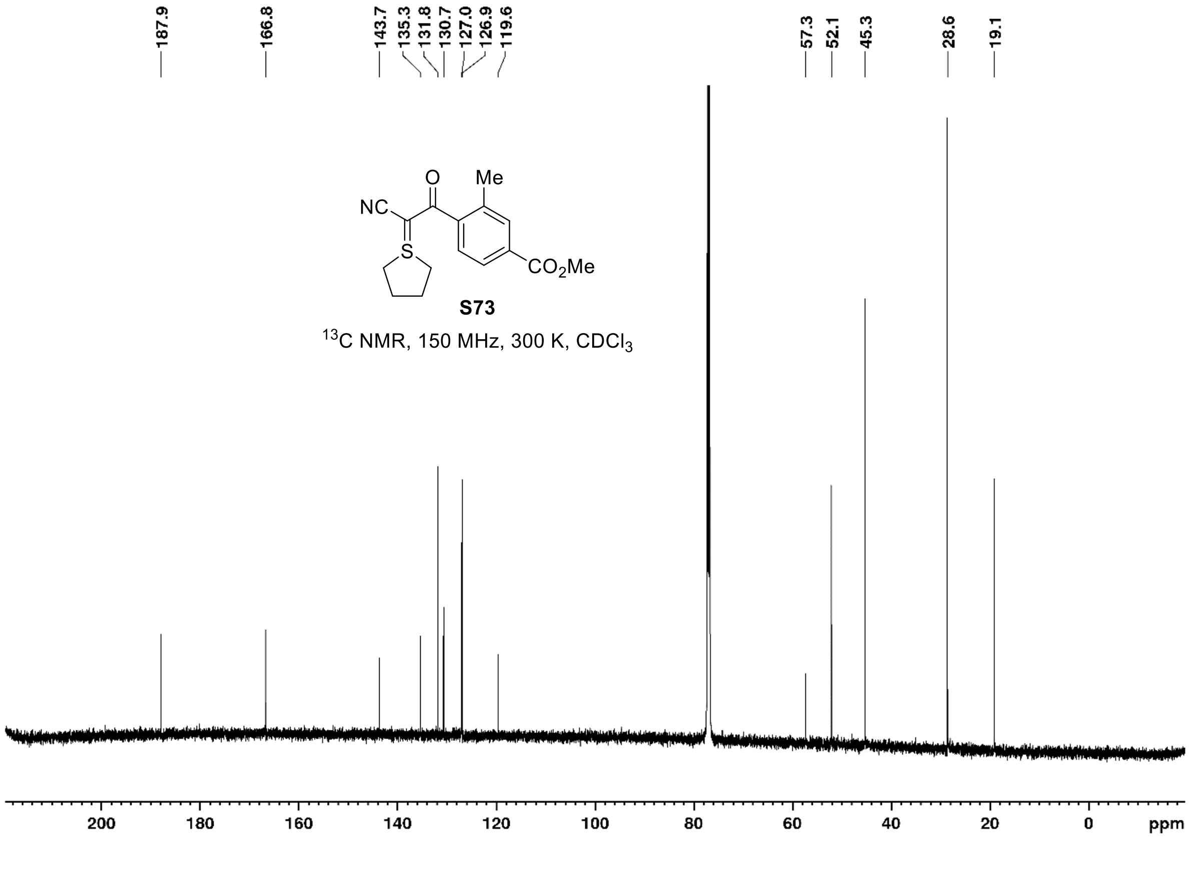
187.9
166.8
143.7 135.3 131.8 130.7 127.0 126.9 119.6
57.3 52.1 45.3
28.6
19.1
NC
O
Me
S
CO2Me
S73
13C NMR, 150 MHz, 300 K, CDCl3
200 180 160 140 120 100 80 60 40 20 0 ppm

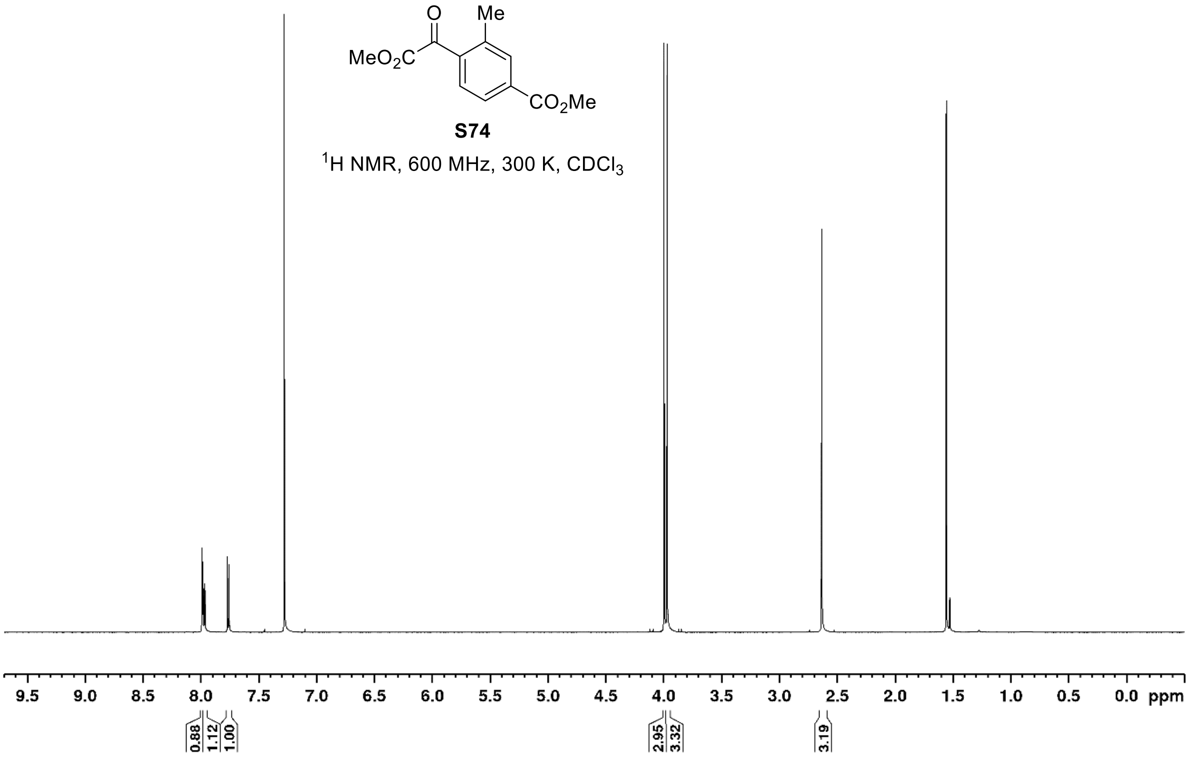

O
Me
MeO2C
CO2Me
S74
1H NMR, 600 MHz, 300 K, CDCl3
9.5
9.0
8.5
8.0
7.5
7.0
6.5
6.0
5.5
5.0
4.5
4.0
3.5
3.0
2.5
2.0
1.5
1.0
0.5
0.0 ppm
0.88
1.12
1.00
2.95
3.32
3.19

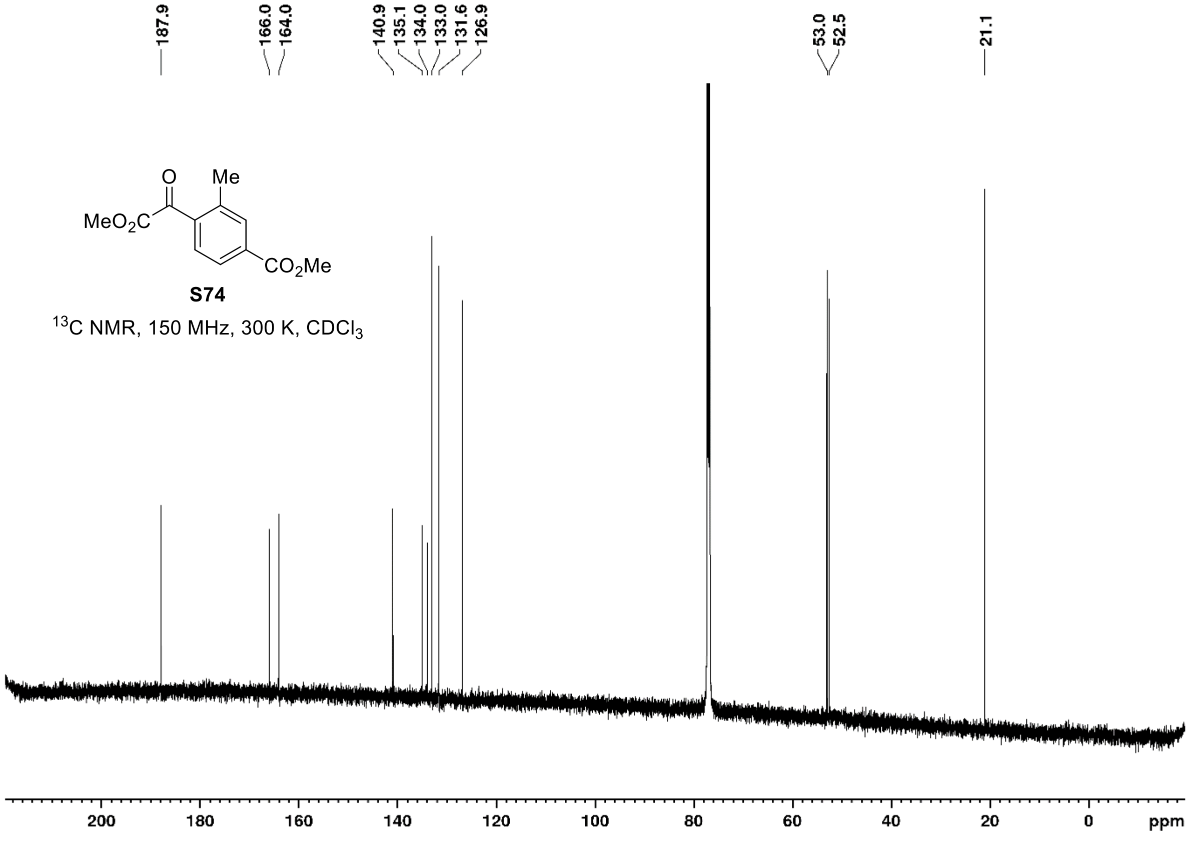

187.9
166.0
164.0
140.9
135.1
134.0
133.0
131.6
126.9
53.0
52.5
21.1
O
Me
MeO2C
CO2Me
S74
13C NMR, 150 MHz, 300 K, CDCl3
200
180
160
140
120
100
80
60
40
20
0
ppm

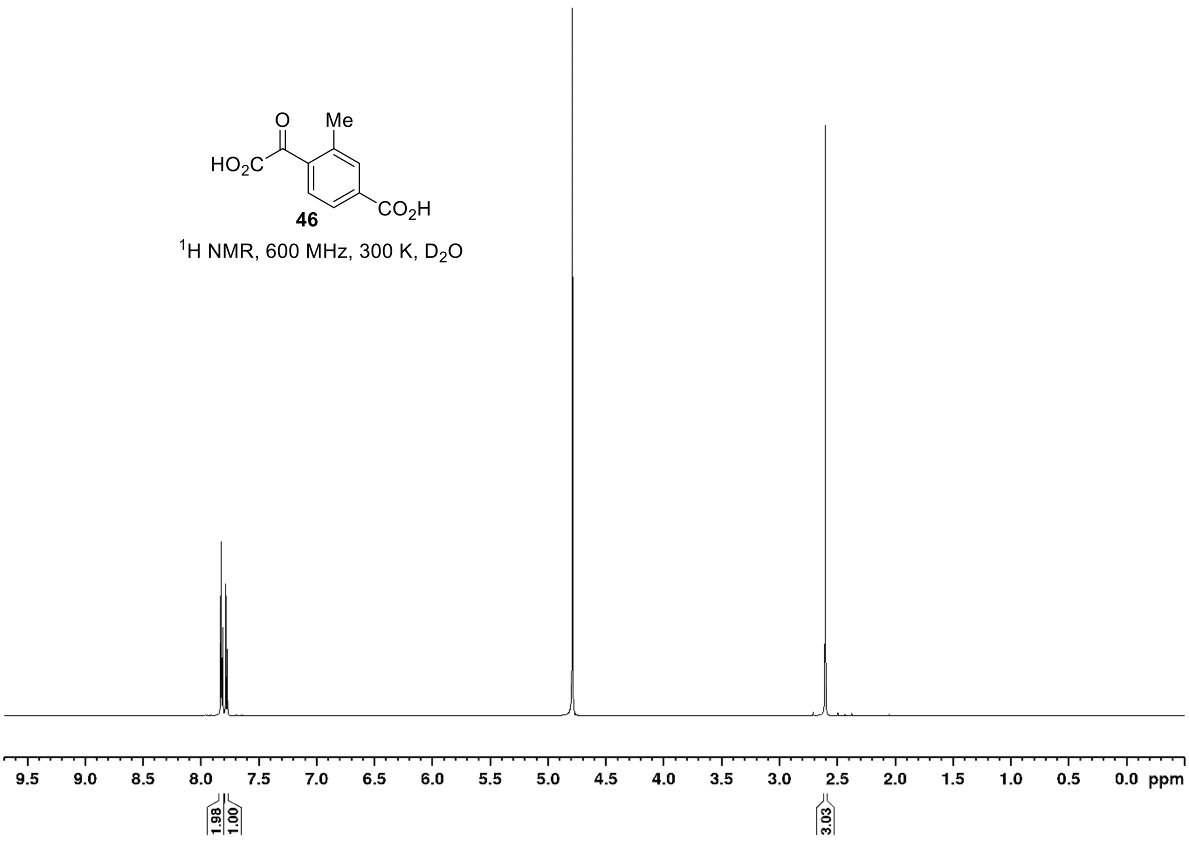
HO2C
O
Me
CO2H
46
1H NMR, 600 MHz, 300 K, D2O
9.5 9.0 8.5 8.0 7.5 7.0 6.5 6.0 5.5 5.0 4.5 4.0 3.5 3.0 2.5 2.0 1.5 1.0 0.5 0.0 ppm
1.98
1.00
3.03

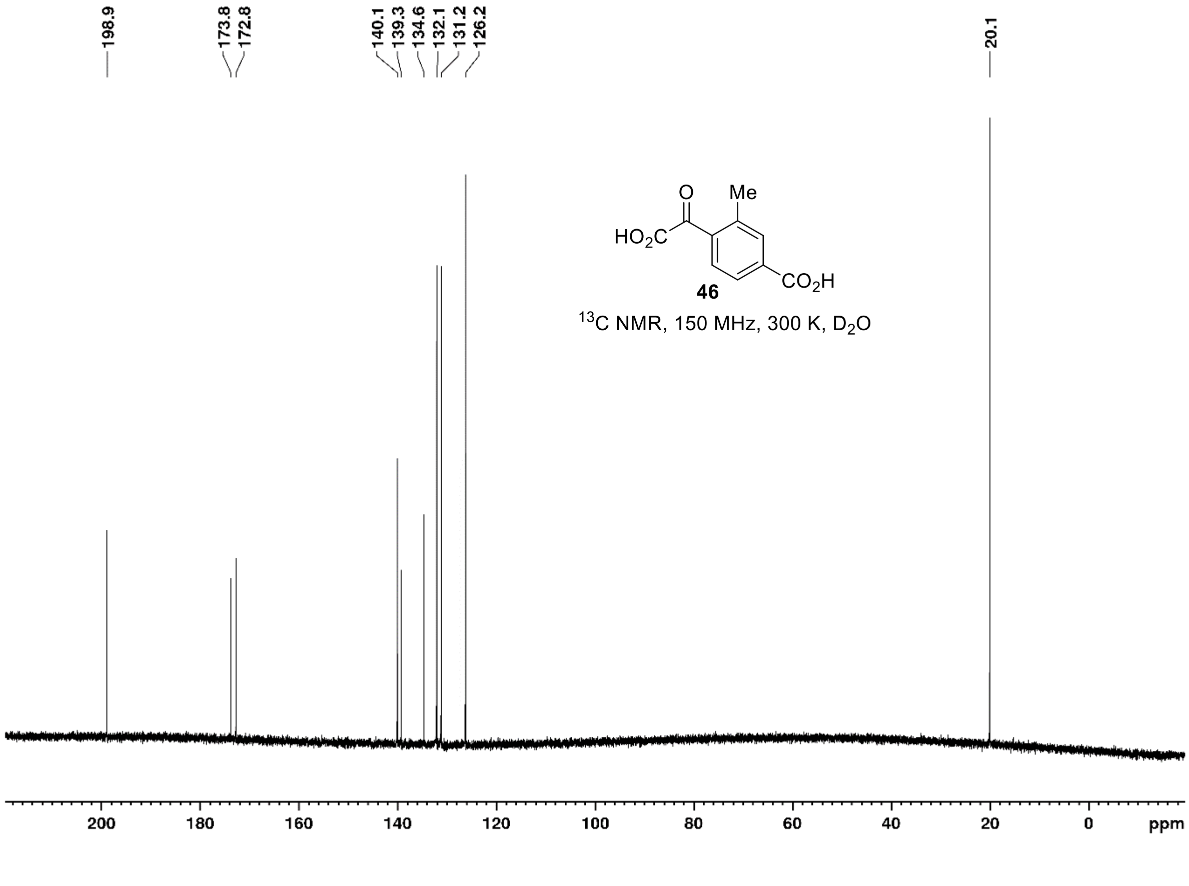
198.9
173.8
172.8
140.1
139.3
134.6
132.1
131.2
126.2
20.1
HO2C
O
Me
CO2H
46
13C NMR, 150 MHz, 300 K, D2O
200 180 160 140 120 100 80 60 40 20 0 ppm

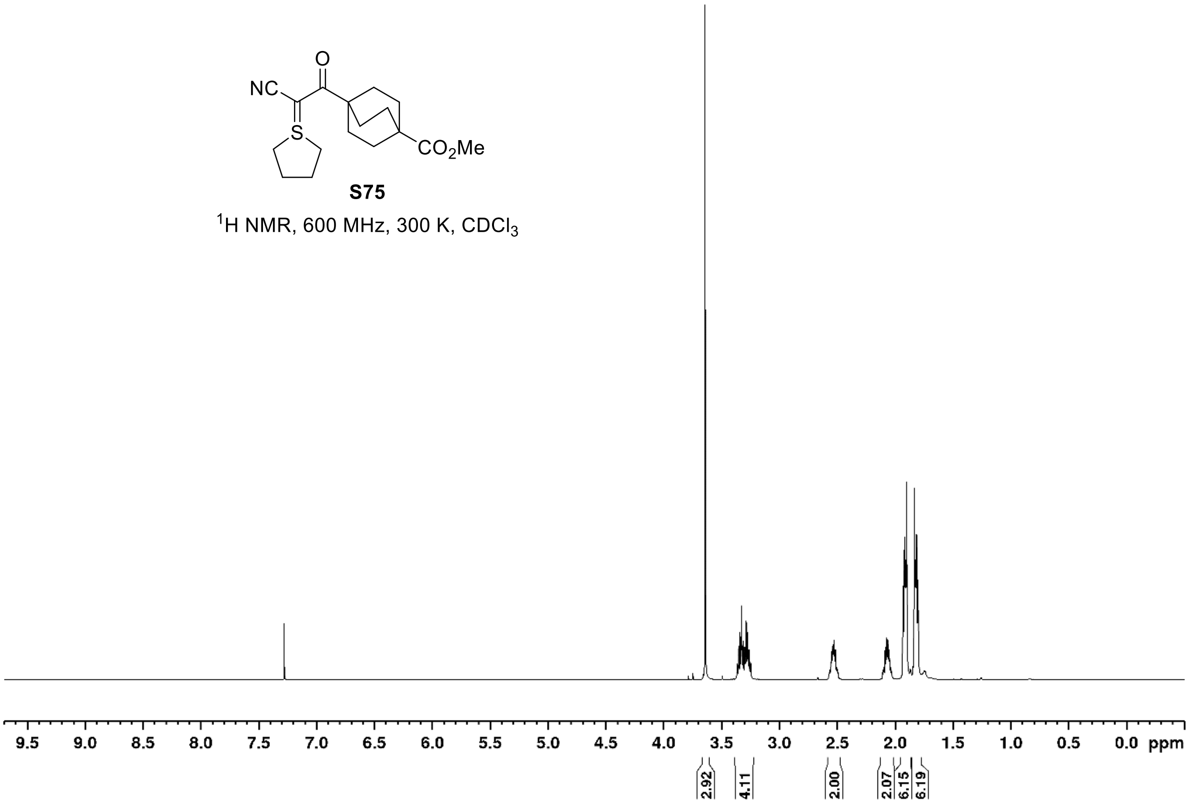
NC
O
S
CO2Me
S75
1H NMR, 600 MHz, 300 K, CDCl3
9.5 9.0 8.5 8.0 7.5 7.0 6.5 6.0 5.5 5.0 4.5 4.0 3.5 3.0 2.5 2.0 1.5 1.0 0.5 0.0 ppm
2.92
4.11
2.00
2.07
6.15
6.19

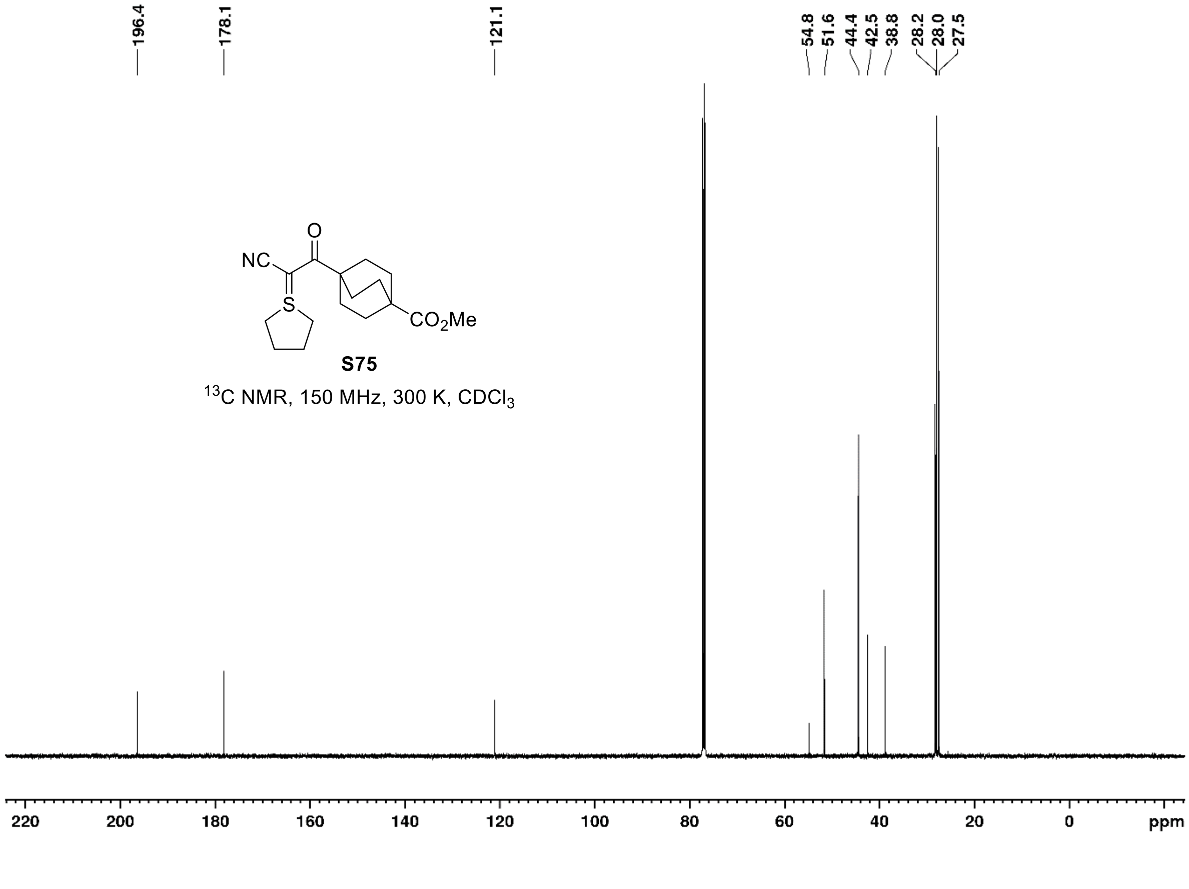
196.4
178.1
121.1
54.8
51.6
44.4
42.5
38.8
28.2
28.0
27.5
NC
O
S
CO2Me
S75
13C NMR, 150 MHz, 300 K, CDCl3
220 200 180 160 140 120 100 80 60 40 20 0 ppm

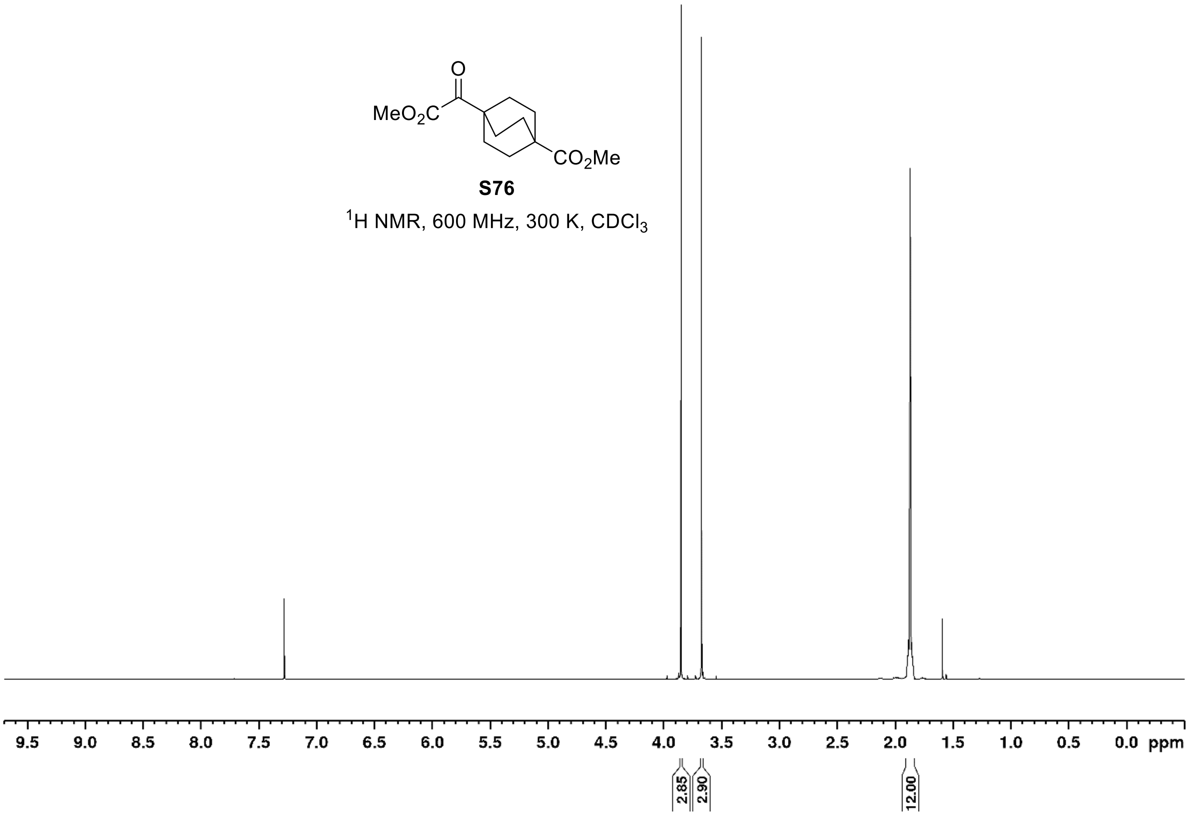
MeO2C
O
CO2Me
S76
1H NMR, 600 MHz, 300 K, CDCl3
9.5 9.0 8.5 8.0 7.5 7.0 6.5 6.0 5.5 5.0 4.5 4.0 3.5 3.0 2.5 2.0 1.5 1.0 0.5 0.0 ppm
2.85
2.90
12.00

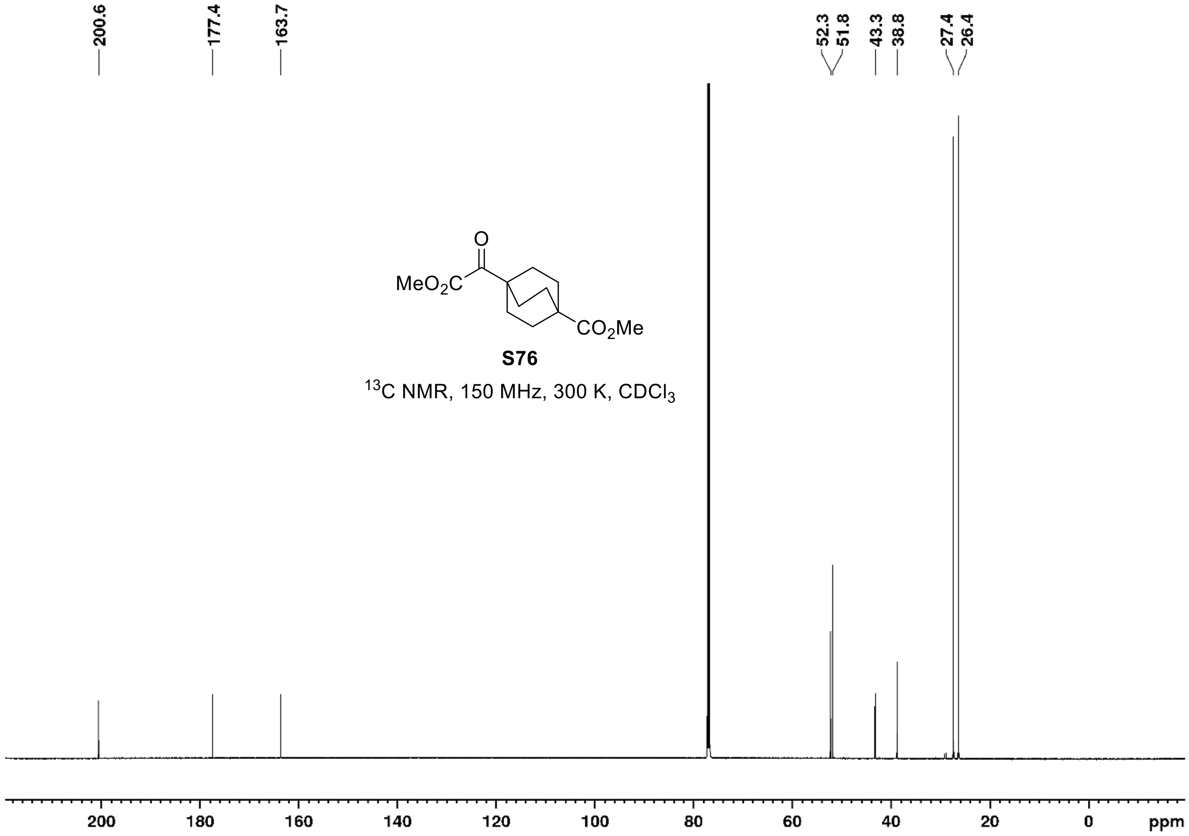
200.6
177.4
163.7
52.3
51.8
43.3
38.8
27.4
26.4
MeO2C
O
CO2Me
S76
13C NMR, 150 MHz, 300 K, CDCl3
200 180 160 140 120 100 80 60 40 20 0 ppm

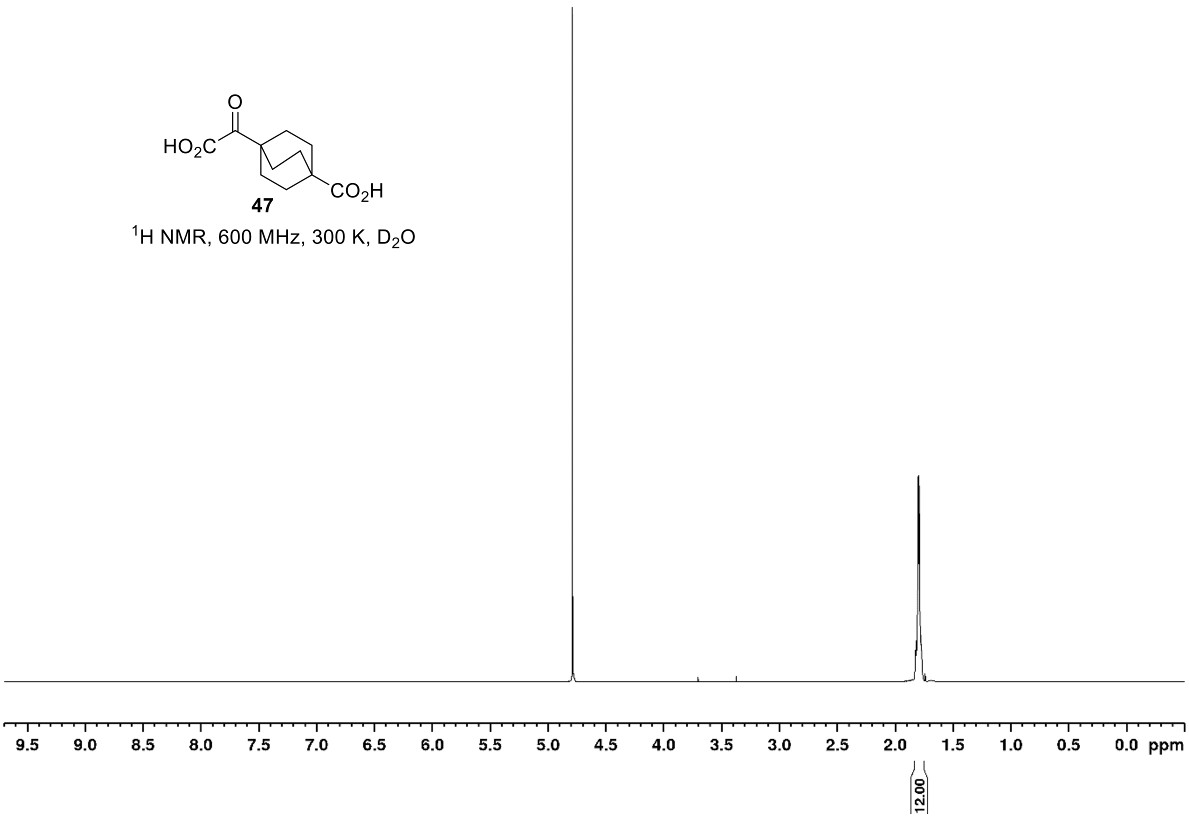
O
HO2C
CO2H
47
1H NMR, 600 MHz, 300 K, D2O
9.5 9.0 8.5 8.0 7.5 7.0 6.5 6.0 5.5 5.0 4.5 4.0 3.5 3.0 2.5 2.0 1.5 1.0 0.5 0.0 ppm
12.00

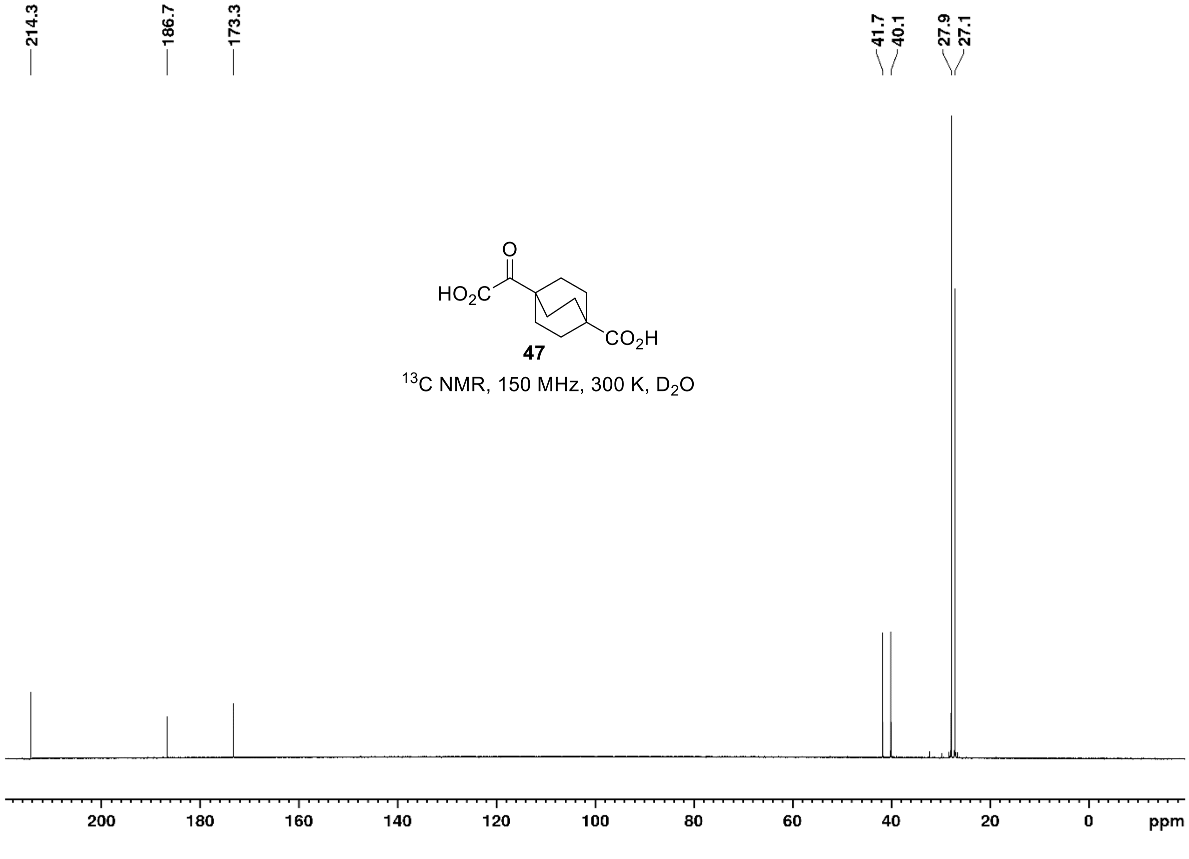
214.3
186.7
173.3
41.7
40.1
27.9
27.1
O
HO2C
CO2H
47
13C NMR, 150 MHz, 300 K, D2O
200 180 160 140 120 100 80 60 40 20 0 ppm

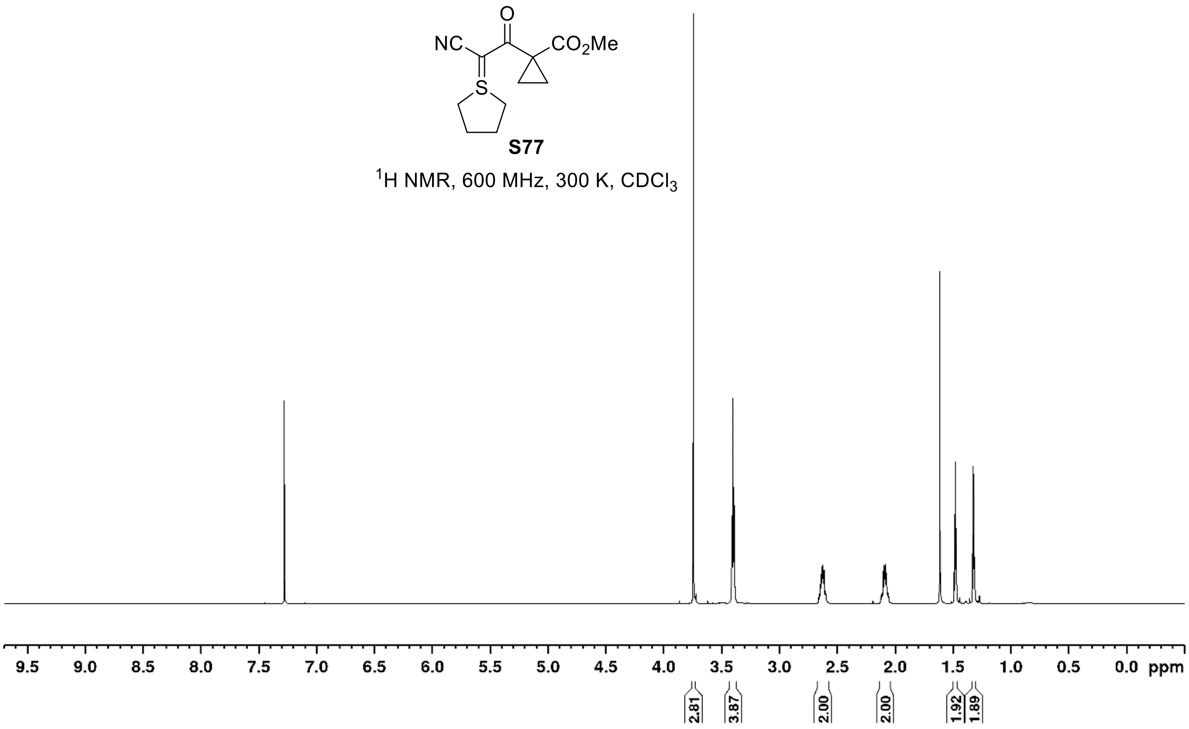
NC
O
CO2Me
S
S77
1H NMR, 600 MHz, 300 K, CDCl3
9.5 9.0 8.5 8.0 7.5 7.0 6.5 6.0 5.5 5.0 4.5 4.0 3.5 3.0 2.5 2.0 1.5 1.0 0.5 0.0 ppm
2.81
3.87
2.00
2.00
1.92
1.89

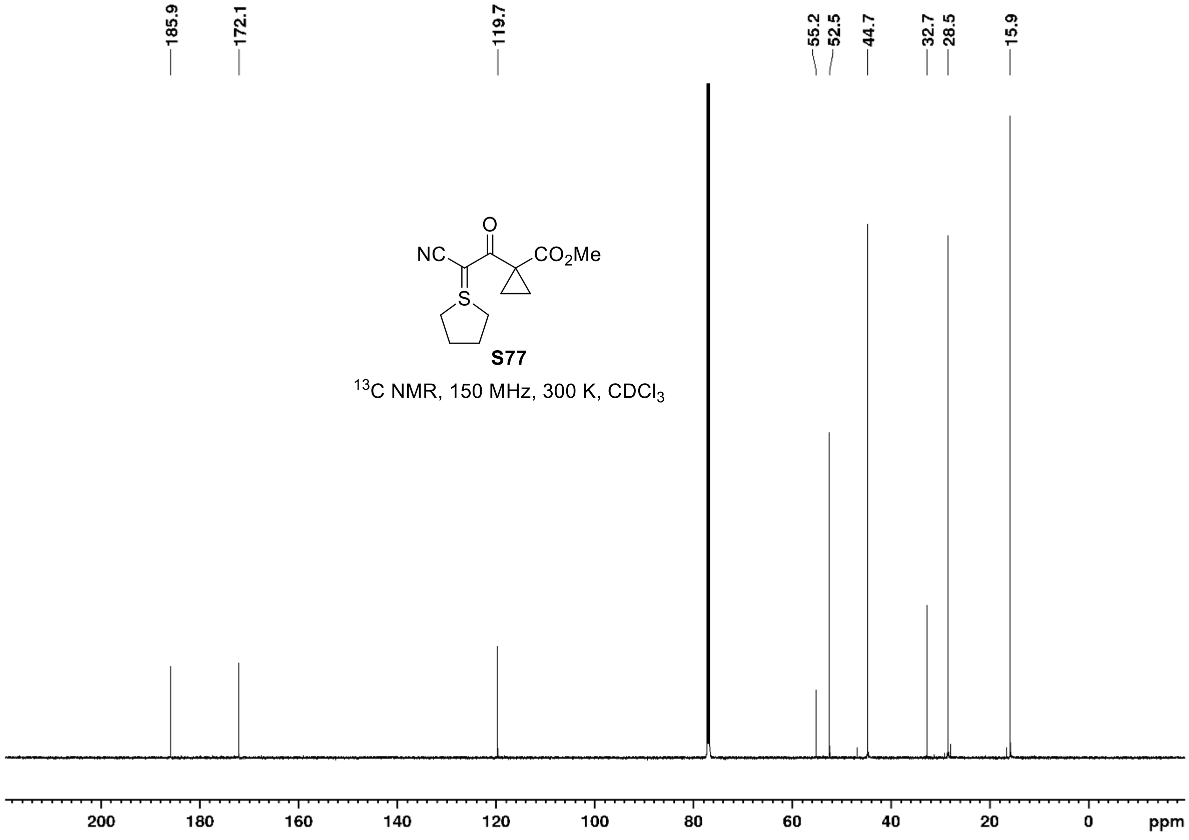
185.9
172.1
119.7
55.2
52.5
44.7
32.7
28.5
15.9
NC
O
CO2Me
S
S77
13C NMR, 150 MHz, 300 K, CDCl3
200 180 160 140 120 100 80 60 40 20 0 ppm

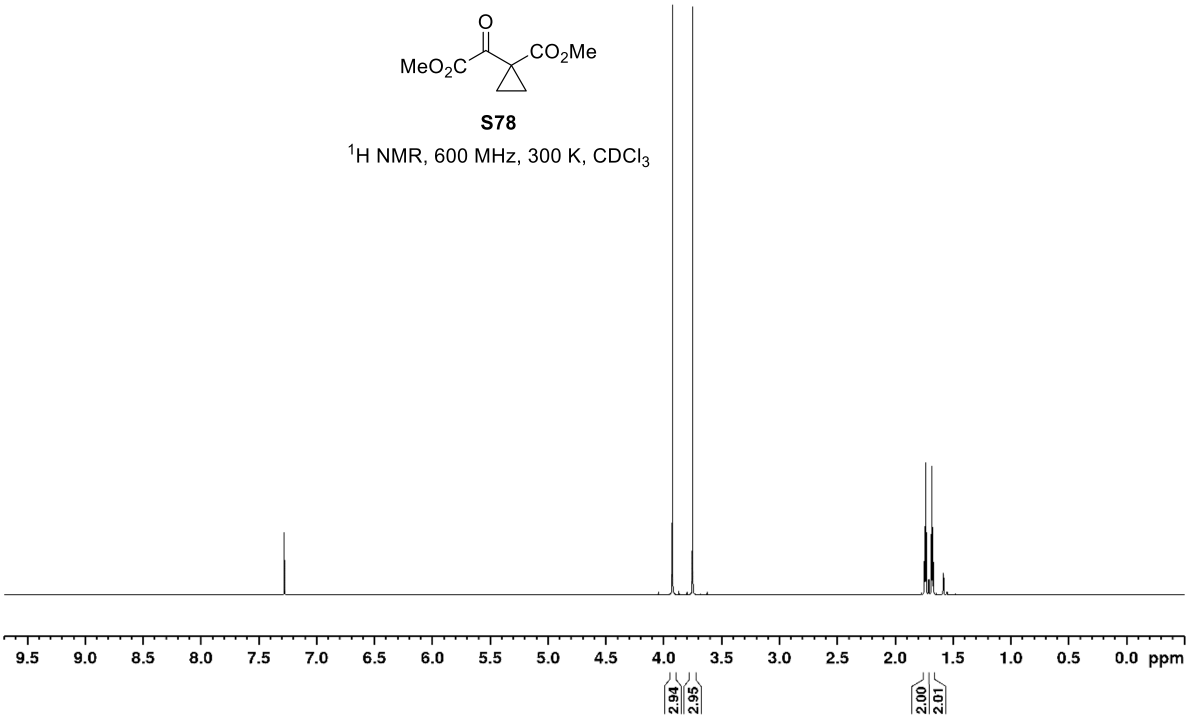
MeO2C
CO2Me
S78
1H NMR, 600 MHz, 300 K, CDCl3
9.5 9.0 8.5 8.0 7.5 7.0 6.5 6.0 5.5 5.0 4.5 4.0 3.5 3.0 2.5 2.0 1.5 1.0 0.5 0.0 ppm
2.94
2.95
2.00
2.01

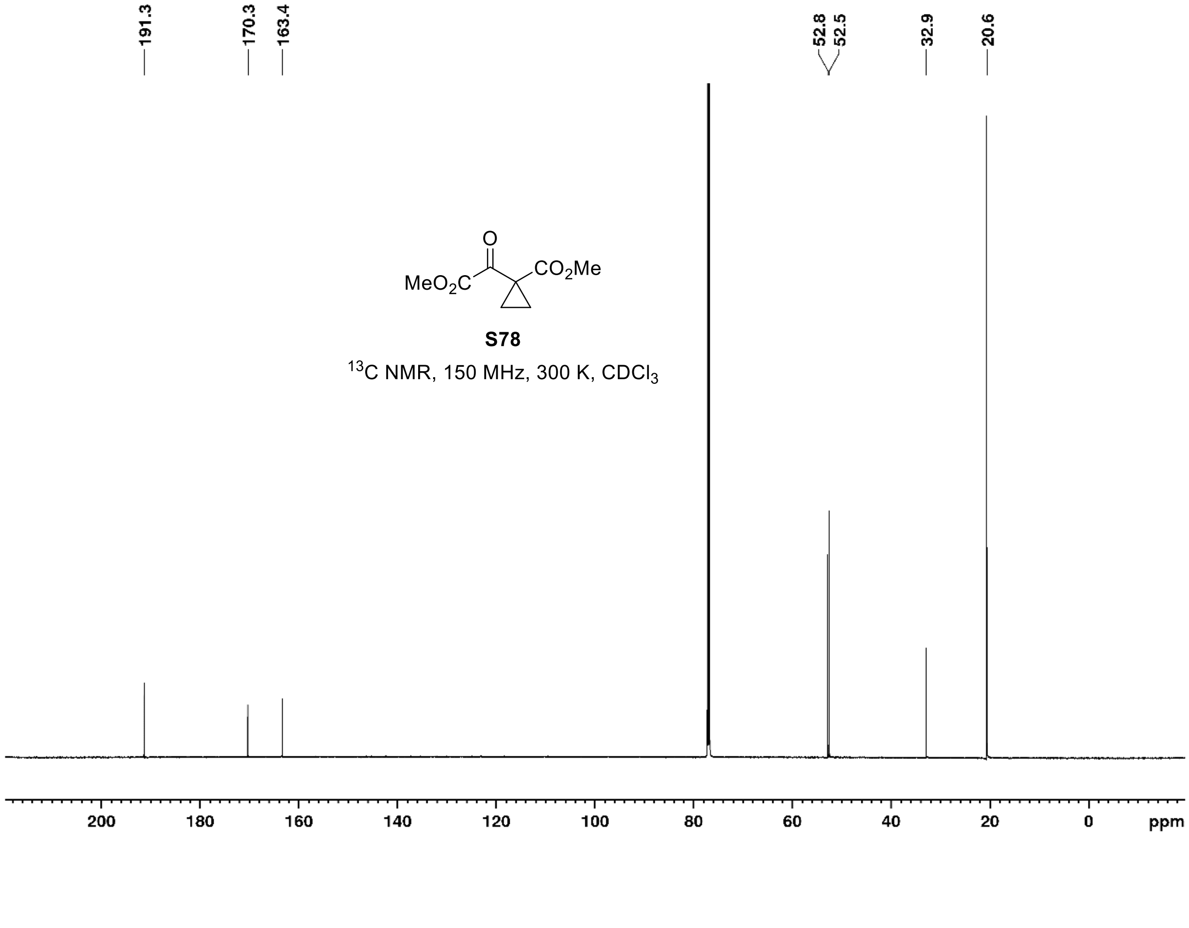
191.3
170.3
163.4
52.8
52.5
32.9
20.6
MeO2C
CO2Me
S78
13C NMR, 150 MHz, 300 K, CDCl3
200 180 160 140 120 100 80 60 40 20 0 ppm

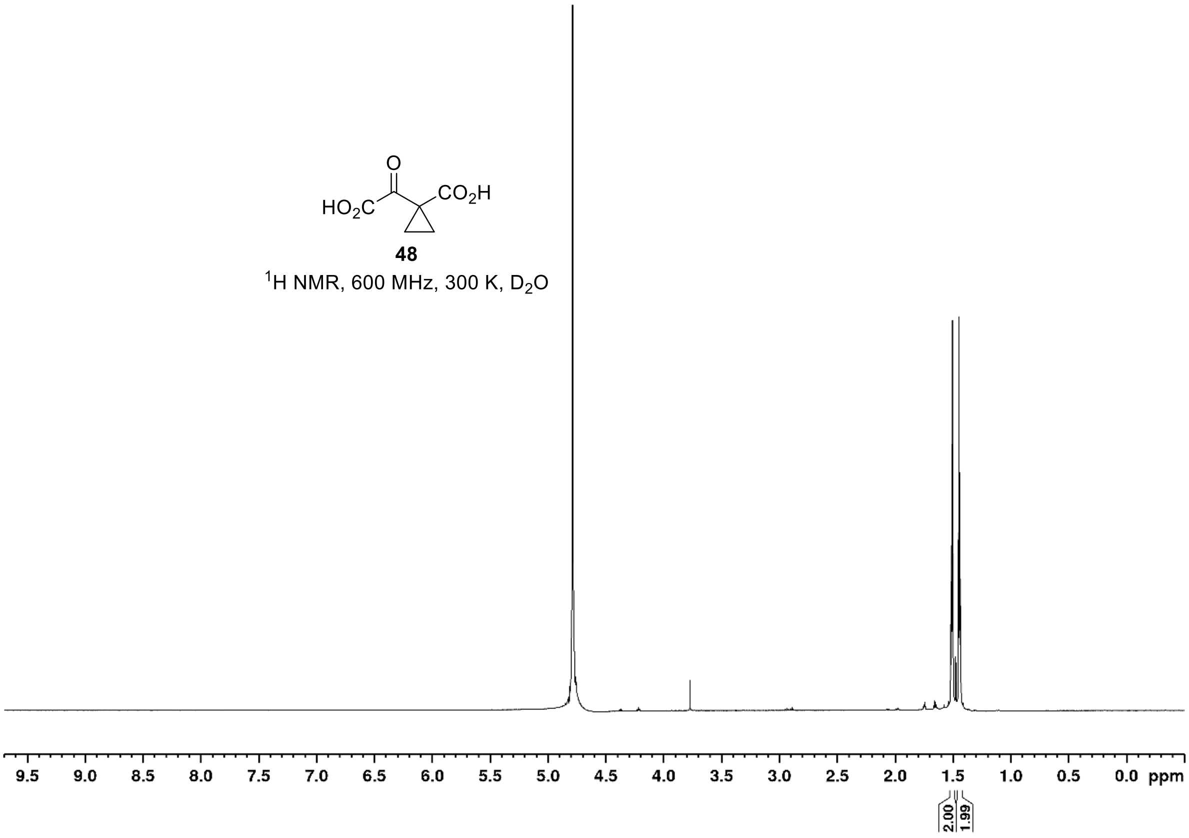
O
HO_2C
CO_2H
48
^1H NMR, 600 MHz, 300 K, D_2O
9.5 9.0 8.5 8.0 7.5 7.0 6.5 6.0 5.5 5.0 4.5 4.0 3.5 3.0 2.5 2.0 1.5 1.0 0.5 0.0 ppm
2.00
1.99

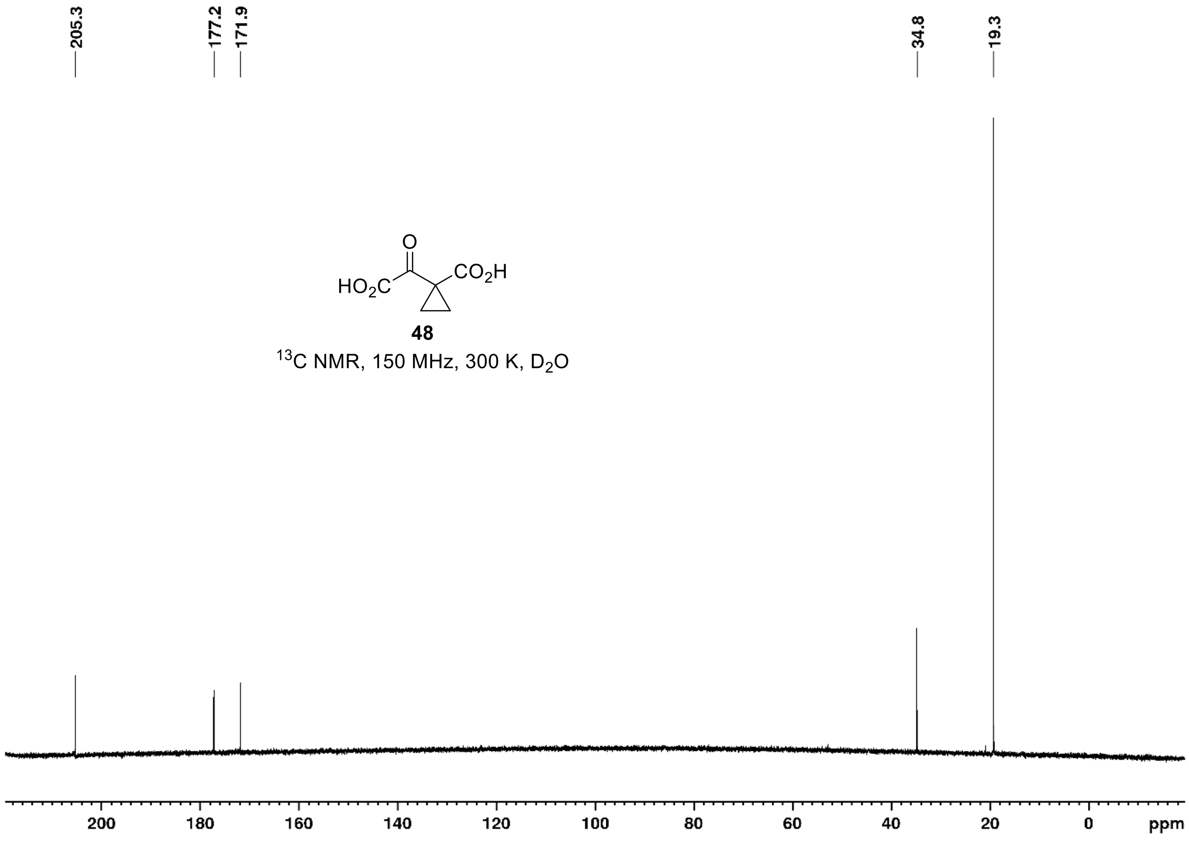
205.3
177.2
171.9
34.8
19.3
O
HO_2C
CO_2H
48
^{13}C NMR, 150 MHz, 300 K, D_2O
200 180 160 140 120 100 80 60 40 20 0 ppm

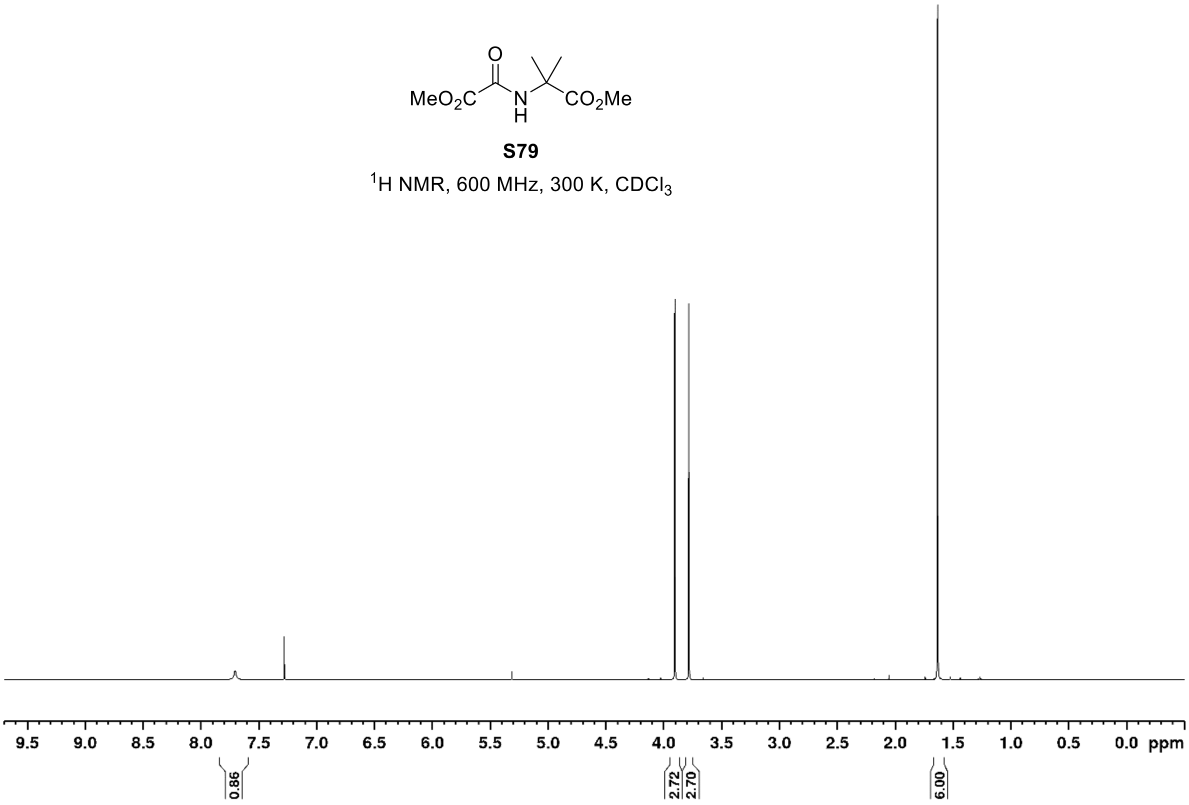
MeO2C
O
N
H
CO2Me
S79
1H NMR, 600 MHz, 300 K, CDCl3
9.5 9.0 8.5 8.0 7.5 7.0 6.5 6.0 5.5 5.0 4.5 4.0 3.5 3.0 2.5 2.0 1.5 1.0 0.5 0.0 ppm
0.86
2.72
2.70
6.00

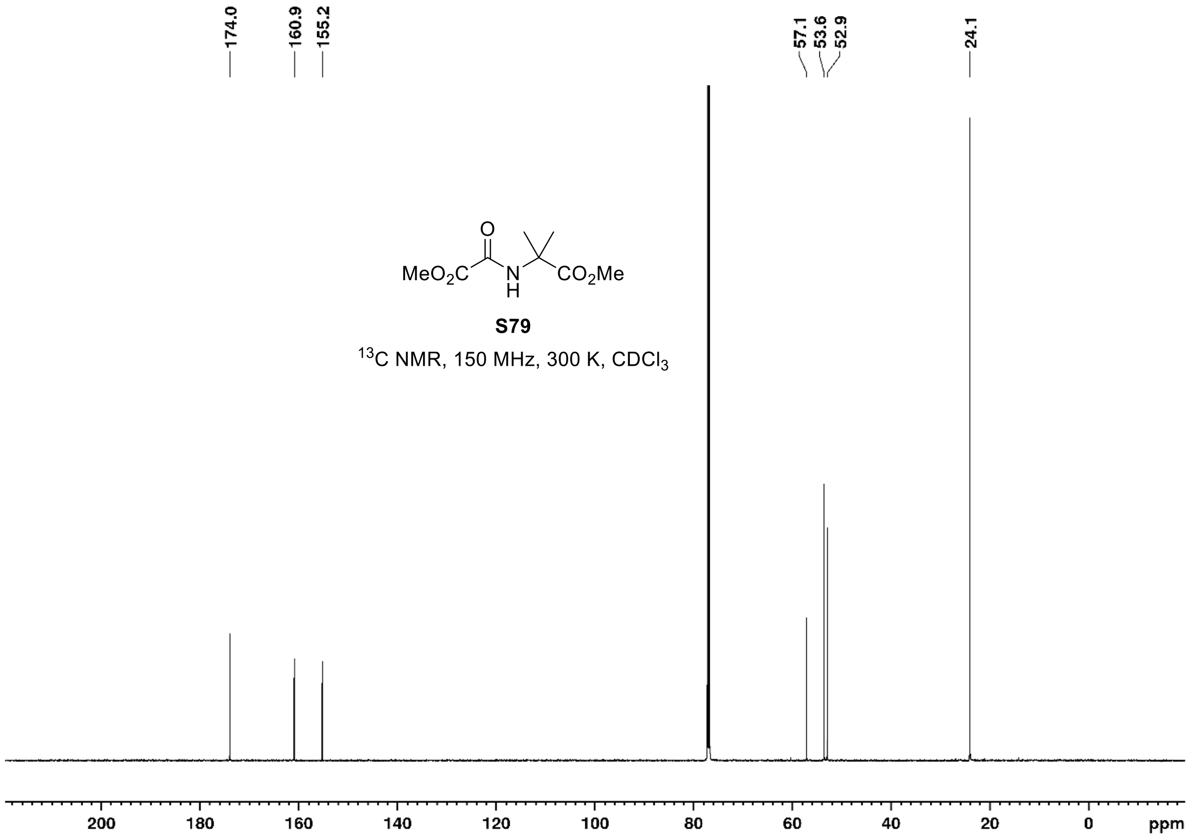
174.0
160.9
155.2
57.1
53.6
52.9
24.1
MeO2C
O
N
H
CO2Me
S79
13C NMR, 150 MHz, 300 K, CDCl3
200 180 160 140 120 100 80 60 40 20 0 ppm

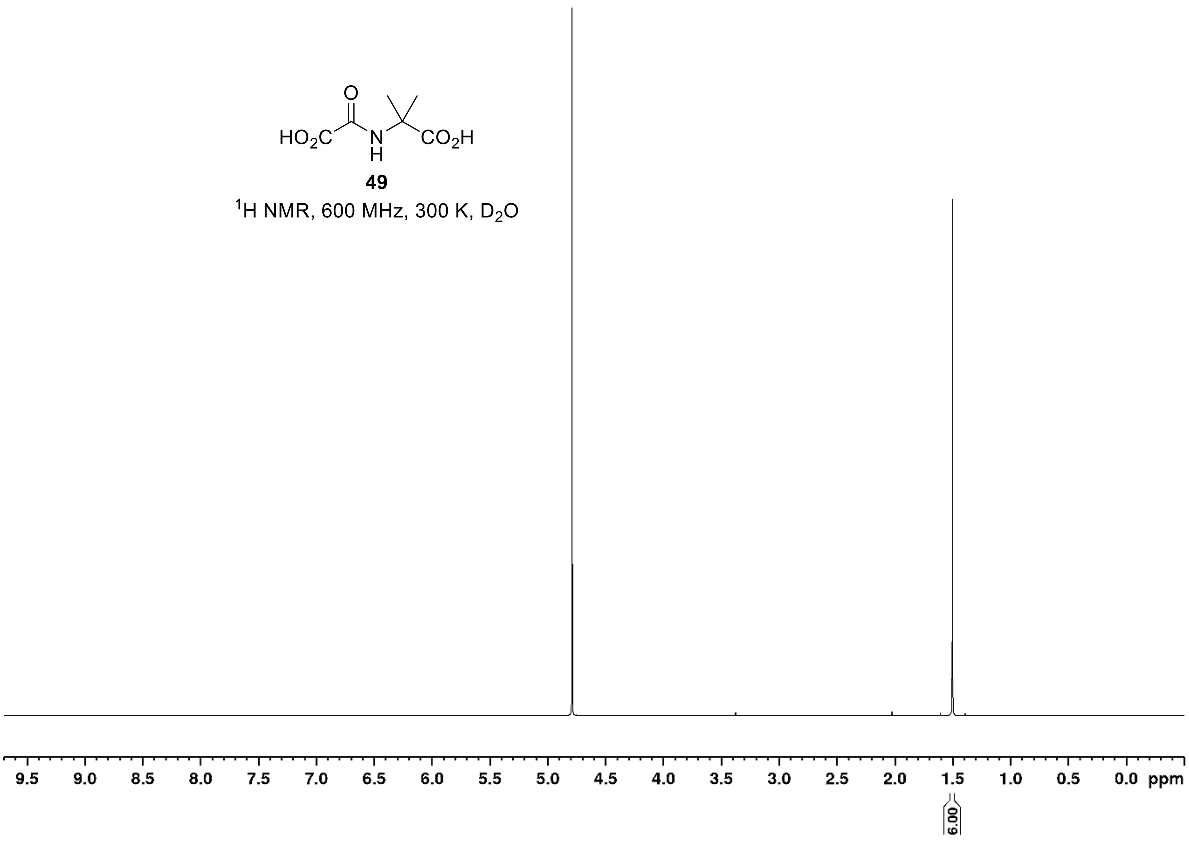
HO2C
O
N
H
CO2H
49
1H NMR, 600 MHz, 300 K, D2O
9.5 9.0 8.5 8.0 7.5 7.0 6.5 6.0 5.5 5.0 4.5 4.0 3.5 3.0 2.5 2.0 1.5 1.0 0.5 0.0 ppm
6.00

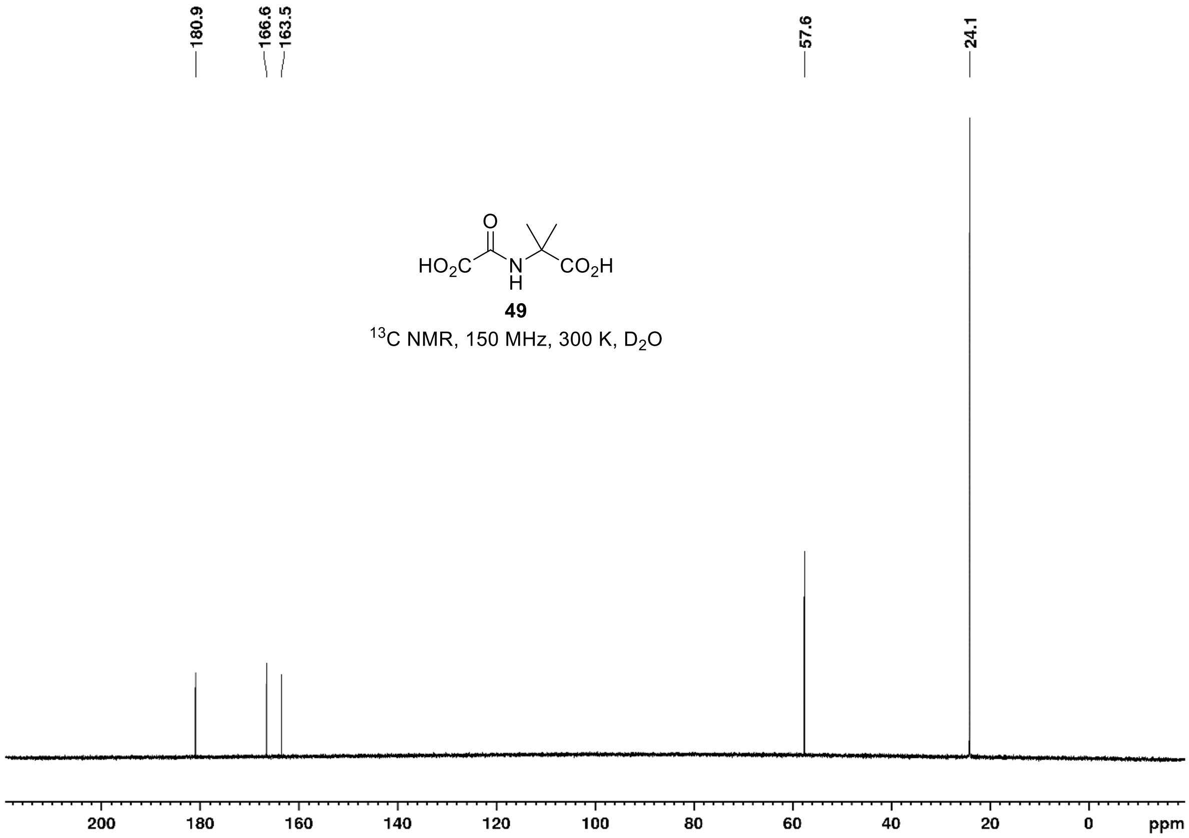
180.9
166.6
163.5
57.6
24.1
HO2C
O
N
H
CO2H
49
13C NMR, 150 MHz, 300 K, D2O
200 180 160 140 120 100 80 60 40 20 0 ppm
